# From Oxygen to Tellurium: The Impact of the Chalcogen on Nucleophilicities and Basicities of Isochalcogenourea Catalysts

**DOI:** 10.1002/anie.202514865

**Published:** 2025-10-23

**Authors:** Lotte Stockhammer, Kevin Kasten, Andreas Eitzinger, Lukas S. Vogl, Magdalena Piringer, David Weinzierl, Armin R. Ofial, Andrew D. Smith, Mario Waser

**Affiliations:** ^1^ Institute of Organic Chemistry Johannes Kepler University Linz Altenbergerstrasse 69 Linz 4040 Austria; ^2^ EaStCHEM School of Chemistry University of St Andrews St Andrews Fife KY169ST UK; ^3^ Department Chemie Ludwig‐Maximilians‐Universität München Butenandtstr. 5‐13 81377 München Germany

**Keywords:** Basicity, Isochalcogenoureas, Nucleophilicity, Organocatalysis

## Abstract

Isochalcogenoureas (IChU) embedded in bi‐ or tricyclic ring systems have proven to be versatile Lewis base/nucleophilic catalysts that activate a wide range of electrophilic substrates for organocatalytic transformations. Ring size, variation of substituents, and the choice of the chalcogen atom affect the efficiency of IChU catalysis in a complex way. To gain a systematic insight into the key parameters that influence reactivity, 14 IChUs covering the fundamental motifs of these structural variations were selected and analyzed by a combination of kinetic, thermodynamic, and quantum‐chemical methods. Two previously unknown tricyclic isotellurourea catalysts were synthesized to facilitate a comparison of all naturally abundant chalcogens (O, S, Se, and Te) in the IChU structure. Furthermore, their reactivity on the Mayr nucleophilicity scale as well as their Brønsted and Lewis basicities were determined in polar organic solvents under standardized conditions. Catalyst performance was assessed in two alcohol acylation reactions and in allenoate activation. The low electronegativity of tellurium gave rise to superior nucleophilicity and Lewis basicity of the isotelluroureas when compared to O‐, S‐, or Se‐containing IChUs. Embedding tellurium in IChU structures thus provides a novel handle to influence and fine‐tune the effectiveness of IChU organocatalysis.

## Graphical abstract

Incorporation of different chalcogen atoms allows for the fine‐tuning of nucleophilicity and basicity of commonly used isochalcogenourea organocatalyst scaffolds. Kinetic and thermodynamic descriptors for the molecular properties of the catalysts are introduced as tools to predict their efficiency and tested in acyl transfer and allenoate activation reactions.

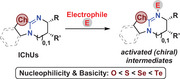

## Introduction

**Scheme 1:**
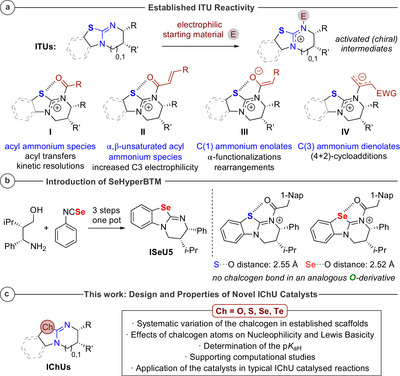
a) Established intermediates in ITU‐catalyzed reactions; b) introduction of SeHyperBTM (**ISeU5**) and reduced Se⋯O distance; c) overview of this contribution.

Bi‐ and tricyclic isochalcogenoureas (IChUs) are a class of versatile nucleophilic/Lewis basic organocatalysts. In particular, chiral isothioureas (ITUs = IChUs with Ch = sulfur) have found widespread use in organic synthesis.^[^ ^1^, ^2^, ^3^, ^4^, ^5^ ^]^ The first use of ITUs as organocatalysts dates back to 2006, when Birman discovered the potential of commercially available tetramisole (**ITU1**, TM) and its benzannulated derivative, benzotetramisole (**ITU2**, BTM) as catalysts for asymmetric acyl transfer reactions (proceeding via in situ formation of acyl ammonium species **I**, Scheme 1a).^[^ ^6^ ^]^ This set the stage for further applications of ITU catalysis for acyl transfer reactions^[^ ^7^, ^8^ ^]^ and (dynamic) kinetic resolutions, including alcohols,^[^ ^6^, ^9^, ^10^, ^11^, ^12^, ^13^, ^14^ ^]^ azlactones,^[^ ^15^, ^16^ ^]^ (thio)lactams,^[^ ^17^, ^18^ ^]^ oxazolidinones,^[^ ^19^ ^]^ azirines,^[^ ^20^ ^]^ and carboxylic acid derivatives.^[^ ^21^, ^22^, ^23^, ^24^, ^25^, ^26^ ^]^ Subsequently, ITUs have proven effective in reactions that involved generation of reactive intermediates, such as chiral α,β‐unsaturated acyl ammonium species **II**,^[^ ^27^, ^28^ ^]^ chiral C(1) ammonium enolates **III**,^[^ ^5^ ^]^ and, more recently, C(3) ammonium dienolates **IV**.^[^ ^29^, ^30^, ^31^, ^32^ ^]^ The acyl ammonium species **I** and **II** and the C(1) ammonium enolates **III** are easily accessible via addition of the ITU to suitable carbonyl precursors, such as ketenes, acid chlorides, (in situ generated) anhydrides and activated esters.^[^ ^1^, ^2^, ^3^, ^4^, ^5^, ^33^, ^34^, ^35^, ^36^, ^37^ ^]^ The acyl ammonium species **I** and **II** represent (chiral) reaction intermediates with increased electrophilicities (in comparison to educts) that allow for numerous (asymmetric) acyl transfer reactions (via **I**) or conjugate addition,^[^ ^34^, ^36^, ^38^, ^39^, ^40^ ^]^ domino/cascade,^[^ ^41^, ^42^ ^]^ and cycloaddition reactions^[^ ^43^, ^44^, ^45^, ^46^, ^47^ ^]^ (utilizing **II**). The C(1) ammonium enolates **III** are well‐suited for asymmetric α‐carbo‐^[^ ^5^, ^48^, ^49^, ^50^, ^51^, ^52^, ^53^, ^54^, ^55^, ^56^, ^57^, ^58^, ^59^, ^60^, ^61^, ^62^, ^63^, ^64^, ^65^, ^66^, ^67^, ^68^, ^69^, ^70^, ^71^ ^]^ and heterofunctionalization reactions of carboxylic acid derivatives (Scheme 1a).^[^ ^5^, ^72^, ^73^, ^74^, ^75^, ^76^, ^77^, ^78^, ^79^, ^80^, ^81^ ^]^ Recent advances described the activation of allenoates using ITUs with the in situ formed chiral dienolates **IV** utilized in (4 + 2)‐cycloaddition reactions.^[^ ^29^, ^30^, ^31^, ^32^ ^]^

Notably, the sulfur atom has a pronounced effect on reactivity and stereoselectivity as it stabilizes the intermediate acyl ammonium species by an intramolecular noncovalent 1,5‐O⋯S chalcogen bonding interaction (*n*_O_→σ*_C‐S_).^[^ ^82^ ^]^ For C(1) ammonium enolates **III**, the enantiocontrol in the subsequent α‐functionalization can thus be rationalized by a conformationally well‐defined chalcogen bond‐stabilized (*Z*)‐enolate,^[^ ^5^, ^8^, ^83^, ^84^ ^]^ where the stereo‐directing phenyl substituent will selectively block either the *Re* or the *Si* face, ensuring electrophile attack from the other, less hindered face.^[^ ^5^, ^85^ ^]^ Similarly well‐defined conformations and spatial arrangements can be observed for the intermediates **I** and **II**, thus allowing for predictable stereoselective transformations.^[^ ^86^ ^]^

Building upon these insights, Smith's group introduced the catalyst **ISeU5** (ISeUs = IChUs with Ch = selenium).^[^ ^87^ ^]^ Computational studies indicated that the chalcogen bonding interaction was stronger in the acylated **ISeU5**‐catalyst than in the **ITU5**‐analogue (Scheme 1b), while this interaction was not detected in the corresponding oxygen‐containing derivative **IU5** (IUs = IChUs with Ch = oxygen), which preferred an *anti*‐coplanar conformation.^[^ ^87^ ^]^ It was also found that **ISeU5** provided enhanced rates of product formation in direct comparison to **ITU5** in several classical benchmarking test reactions. Since this initial demonstration, several examples have shown that **ISeU5** often leads to enhanced product yields and higher selectivity than **ITU5** or facilitates reactions that were previously difficult to achieve.^[^ ^29^, ^30^, ^31^, ^32^, ^55^, ^73^, ^88^ ^]^

**Figure 1:**
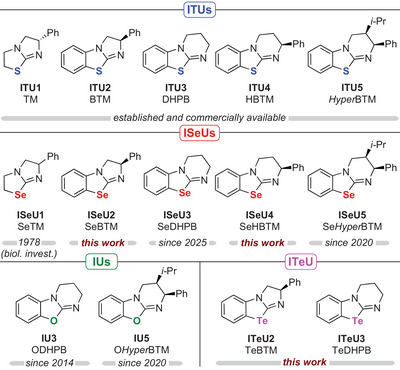
Overview of the IChUs investigated in this study: Established **ITU1**‐**5**, the recently introduced **ISeU5** and **IU5**,^[^ ^87^ ^]^ **ISeU1** (which was investigated for its biological activity in Ref. [90]), **IU3** (which was tested for its acyl‐transfer potential in Ref. [91]), and the herein newly synthesized **ISeU2, ISeU4** and **ITeU2‐3**. During the preparation of this manuscript, **ISeU3** was synthesized and used in catalytic reactions by the Wiskur group, too.^[^ ^89^ ^]^

The experimentally observed higher catalytic activity of **ISeU5** compared to **ITU5** and the fact that the different established ITU scaffolds (**ITU1**–**5**, Figure 1) show varying reactivity and catalytic performance for different target reactions triggered our interest to launch a systematic investigation of the effect of the chalcogen atom on the reactivity of IChU catalysts.^[^ ^89^ ^]^ We, therefore, focused on a comparative evaluation of the oxygen (IU), sulfur (ITU), and selenium analogues (ISeU) that maintained the scaffolds and substitution patterns of the established **ITU1‐5** catalysts. To further challenge the concept of tuning IChU reactivity by varying the chalcogen, expansion to the heavier chalcogen homologs was considered to include the novel isotellurourea derivatives **ITeU2‐3** (ITeU = IChU with Ch = tellurium) as potential nucleophilic/Lewis basic catalysts in our studies (Scheme 1c).

In this work, we characterize the nucleophilicity and the basicity of the IChU catalysts (Figure 1) by experimental and quantum‐chemical techniques. Selected test reactions are used to correlate the thus determined kinetic and thermodynamic descriptors with the activity of the IChU catalysts in organocatalytic reactions. As a consequence, this work provides detailed insight into the properties of benchstable IChU catalysts and outlines synthetic strategies that will facilitate their adaption for further applications. In particular, introducing the tellurium‐derivatives **ITeU2** and **ITeU3** establishes a novel class of organocatalysts, which is shown to be significantly more nucleophilic and (Lewis) basic than the structurally analogous ISeUs. Additionally, going from Se to Te increasingly leverages chalcogen bonding interactions.

## Results and Discussion

### Syntheses of Isochalcogenoureas

**Scheme 2:**
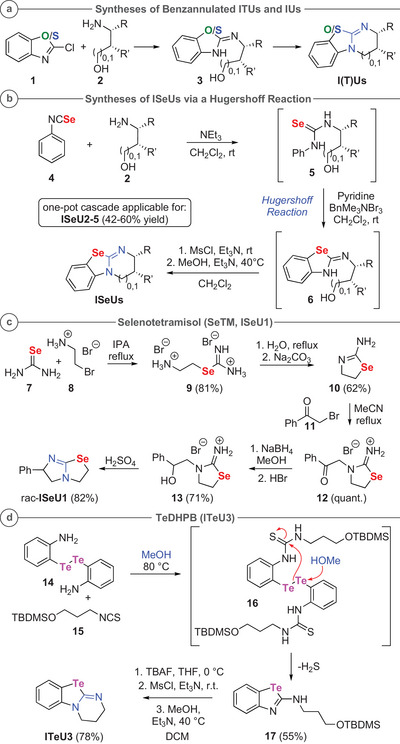
Synthesis methods for different isochalcogenourea catalysts. a) Syntheses starting from compound **1**; b) syntheses of **ISeU2‐5** starting from selenoureas **5**; c) syntheses of **ISeU1**; d) synthesis of **ITeU3** starting from ditelluride **12** (chiral **ITeU2** was accessed by analogy as outlined in the Supporting Information).

A classical approach to access the benzannulated isothioureas **ITU2‐5**, as well as **IU3** and **IU5**, is to react 2‐chlorobenzothiazol (or ‐oxazol) **1** with aminoalcohols **2**. This results in the formation of compounds **3**, which can then be cyclized to the corresponding ITUs and IUs straightforwardly (Scheme 2a).^[^ ^7^, ^8^, ^87^ ^]^ When suitable benzochalcogenazol precursors **1** are not easily accessible, as for instance the Se‐derivatives,^[^ ^92^ ^]^ an alternative Hugershoff strategy to construct such tricyclic isochalcogenoureas can be employed to install the cyclic isochalcogenourea motif.^[^ ^93^, ^94^ ^]^ We previously utilized this methodology to access **ISeU5** in a one‐pot cascade manner by starting from the respective aminoalcohol **2** and isoselenocyanate **4**.^[^ ^87^ ^]^ This strategy was expandable to alternative aminoalcohols **2** giving the corresponding selenoureas **5**, which were directly cyclized to products **6** under “Hugershoff conditions.” Mesylation and final cyclization gave the novel isoselenoureas **ISeU2‐4** in a scalable manner (Scheme 2b).^[^ ^95^ ^]^

SeTM (**ISeU1**) was synthesized by R.N. Hanson et al. in 1978 in order to investigate its potential for the inhibition of alkaline phosphatase isoenzymes compared to the commercially available drug TM (**ITU1**).^[^ ^90^ ^]^ Adapting this reported synthetic sequence (Scheme 2c) gave access to *rac*‐**ISeU1** starting from selenourea **7** and 2‐bromoethylamine **8**.^[^ ^90^, ^96^ ^]^ While *rac*‐**ISeU1** was sufficient for the intended kinetic and thermodynamic investigations, considering its potential for catalytic asymmetric applications enantioenriched **ISeU1** would be necessary. Attempted resolution of *rac*‐**ISeU1** with camphor sulfonic acid^[^ ^90^ ^]^ gave **ISeU1** with an enantiomeric ratio of 90:10.

Furthermore, the synthesis of the achiral **ITeU3** as the first member of the unprecedented isotelluroureas (ITeUs) was investigated (Scheme 2d). Accessing these compounds via a Hugershoff reaction was not feasible due to inaccessibility of the required isotellurocyanate.^[^ ^97^ ^]^ An alternative strategy used amino‐substituted diaryl ditelluride **14** ^[^ ^98^ ^]^ as the starting material. Bonifazi and Junk had utilized such easily accessible ditellurides for the synthesis of 2‐carbo‐ and 2‐hetero‐functionalized benzotellurazoles.^[^ ^98^, ^99^, ^100^, ^101^, ^102^, ^103^ ^]^ We thus planned to react ditelluride **14** with isothiocyanate **15** to access the bis‐thiourea **16**. Reductive conditions were predicted to cleave the ditelluride bond, followed by cyclization to the benzotellurazole **17**. After carrying out the reaction of **14** and **15** in acetone at room temperature without any product formation, we surprisingly found that by using EtOH as solvent at 80 °C the target cyclic compound **17** was formed, accompanied by unidentified side‐ and degradation products. Further optimization revealed that switching to MeOH at 80 °C gave highest selectivity toward product **17**, while using *i*‐PrOH at the same temperature gave significant degradation.^[^ ^95^ ^]^ We speculate that formation of **17** proceeds via initial formation of thiourea **16**, followed by nucleophilic attack of MeOH to promote Te‐Te bond cleavage, followed by cyclization to the thiourea carbon. The fate of the remaining telluroaniline species is unknown at present. However, compound **17** was obtained in more than 50% yield based on the amount of tellurium present in ditelluride **14**. This observation indicates that not only one “half” of compound **16** is converted into product **17**, but also that the postulated addition product of the MeOH to one of the Te‐atoms (the second “half” of the ditelluride) can enter a productive path toward **17**. To our delight, after TBDMS deprotection, the second cyclization step was achieved using reported procedures for the thio‐derivatives.^[^ ^7^, ^8^ ^]^ After trituration with ethyl acetate, isotellurourea **ITeU3** was obtained in satisfying quality and moderate overall yield (Scheme 2d). Noteworthy, single crystal X‐ray analysis of **ITeU3** [CCDC 2452429] revealed short intermolecular Te⋯N distances of 2.8658(52) Å [*r*_vdW_(Te) = 1.99 Å; *r*_vdW_(N) = 1.66 Å],^[^ ^95^, ^104^ ^]^ presumably caused by attractive noncovalent chalcogen bonding interactions, which indicated a significant potential of **ITeU3** to stabilize intermediates of type **I**, **II**, or **III** by leveraging σ‐hole interactions.^[^ ^82^, ^87^ ^]^ Gratifyingly, this synthetic strategy could also successfully be utilized to access TeBTM (**ITeU2**) as first chiral member of this isotellurourea family.^[^ ^95^ ^]^

### Nucleophilicity of IChUs

It has been shown previously that the Mayr–Patz equation (MPE), Equation (1), describes the rates of thousands of polar, σ‐bond‐forming reactions of nucleophile/electrophile combinations, in which at least one of the reaction centers is a carbon atom.^[^ ^105^, ^106^, ^107^, ^108^, ^109^ ^]^

(1) $$\text{Mayr-Patz Equation: lg}\;k_{{20\;}^{\circ }C}=s_{N}\left(N+E\right)$$

The MPE is a linear‐free‐energy relationship that characterizes electrophiles by the electrophilicity parameter *E* and nucleophiles by the solvent‐dependent parameters *N* (nucleophilicity) and *s*_N_ (sensitivity toward changes in the electrophile reactivity). By using the MPE (Equation (1)), the construction of comprehensive nucleophilicity and electrophilicity scales becomes possible, which facilitate the comparison of various classes of Lewis base organocatalysts.^[^ ^110^, ^111^, ^112^, ^113^, ^114^, ^115^, ^116^, ^117^, ^118^, ^119^, ^120^ ^]^

**Figure 2:**
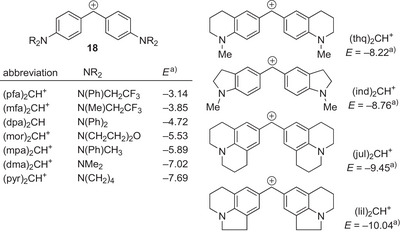
Benzhydrylium ions (Ar_2_CH^+^) used as reference electrophiles to determine the nucleophilic reactivity of the IChU catalysts. a) Electrophilicity parameters *E* are from Ref. [106].

In order to systematically investigate the influence of the chalcogen atom on the nucleophilicity of DHPB‐derived IChUs (Ch = O, S, Se, and Te), we utilized Mayr's benzhydrylium methodology.^[^ ^107^ ^]^ Hence, we followed the kinetics of IChU reactions with a series of benzhydrylium ions of known electrophilicity *E* (reference electrophiles, Figure 2) under standardized conditions, that is, in dichloromethane at 20 °C.

**Figure 3:**
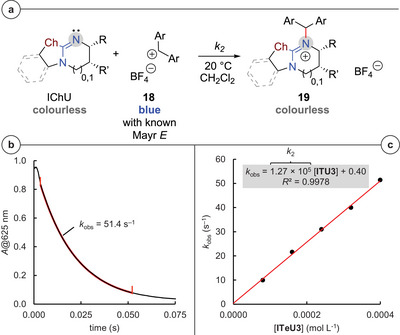
a) Covalent bond formation in reactions of IChUs with benzhydrylium ions **18**. b) Determination of the observed rate constant *k*_obs_ (s^−1^) from the time‐dependent exponential decay of the (ind)_2_CH^+^ absorbance *A* at 625 nm for the reaction of (ind)_2_CH^+^ (*c*_0_ = 8.0 × 10^−6^ mol L^−1^) with **ITeU3** (*c*_0_ = 4.0 × 10^−4^ mol L^−1^, 50 equiv). The section marked in red indicates the range of the decay curve that was used for the *k*_obs_ determination. c) The slope of the linear correlation of *k*_obs_ with [**ITeU3**] corresponds to the second‐order rate constant *k*_2_ (L mol^−1^ s^−1^) for the reaction of **ITeU3** with (ind)_2_CH^+^ (counterion: BF_4_^–^).

In accordance with observations in previous studies on ITU reactivity,^[^ ^119^ ^]^ mixing the strongly colored benzhydrylium tetrafluoroborates **18** with excess IChUs in dichloromethane gave rise to decolorization of the solutions due to the formation of isochalcogenouronium tetrafluoroborates **19**, which are colourless (Figure 3a). Owing to reversibility of the C─N bond formation, generated adducts **19** from the IChU/benzhydrylium reactions were not isolated but characterized by ^1^H, ^13^C, and ^77^Se NMR spectroscopy (the ^77^Se nucleus is remarkably sensitive with observed changes of around 100 ppm when comparing the ^77^Se resonances of free ISeUs with those of the corresponding selenouronium tetrafluoroborates^[^ ^95^ ^]^). The kinetics of the IChU reactions with the reference electrophiles **18** were traced by photometric methods using standard stopped‐flow techniques. To establish pseudo first‐order conditions and to secure a sufficient decay of the benzhydrylium's absorbance during the course of the reactions, the IChUs were employed at higher concentrations than the benzhydrylium ions. First‐order rate constants *k*_obs_ (s^−1^) were determined by fitting the mono‐exponential decay function in Equation (2) to the decreasing absorbance (*A*) of **18** during their reaction with the IChUs as exemplified for the reaction of **ITeU3** with (ind)_2_CH^+^ in Figure 3b.

(2) $$A=A_{0}\;e^{-k_{\mathrm{obs}}t}+C$$

First‐order rate constants *k*_obs_ correlated linearly with the IChU concentration. Therefore, second‐order rate constants *k*_2_ (L mol^−1^ s^−1^) were determined from the slope of the linear regression curves (Figure 3c ^[^ ^95^ ^]^). Second‐order rate constants *k*_2_ for all kinetically investigated reactions of DHPB‐derived IChUs (Ch = O, S, Se, and Te) with benzhydrylium ions **18** are gathered in Table 1.

**Table 1:** Second‐order rate constants *k*_2_^exptl^ for the reactions of DHPB‐derived IChUs (Ch = O, S, Se, and Te) with benzhydrylium ions (Ar_2_CH^+^) in dichloromethane at 20 °C used for the determination of the nucleophilicity parameters *N* (and *s*_N_). For comparison Brønsted basicities p*K*_aH_ (in MeCN, DMSO, and aqueous solution) and benzhydrylium‐derived Lewis basicities (*LB*) (in dichloromethane) are also given.

		*k*_2_^exptl^ (L mol^−1^ s^−1^)			
Ar_2_CH^+^	*E*	Isourea ODHPB (**IU3**)	Isothiourea DHPB (**ITU3**)	Isoselenourea SeDHPB (**ISeU3**)	Isotellurourea TeDHPB (**ITeU3**)
(lil)_2_CH^+^	−10.04	–	–	1.98 × 10^3^	1.94 × 10^4^
(ind)_2_CH^+^	−8.76	1.20 × 10^3^	1.11 × 10^4^ ^a)^	1.81 × 10^4^	1.27 × 10^5^
(thq)_2_CH^+^	−8.22	–	2.07 × 10^4^ ^a)^	3.81 × 10^4^	–
(pyr)_2_CH^+^	−7.69	7.93 × 10^3^	7.49 × 10^4^ ^a)^	1.07 × 10^5^	7.31 × 10^5^
(dma)_2_CH^+^	−7.02	2.79 × 10^4^	2.29 × 10^5^ ^a)^	1.94 × 10^5^	1.67 × 10^6^
(mpa)_2_CH^+^	−5.89	1.29 × 10^5^	–	1.16 × 10^6^	–
(mor)_2_CH^+^	−5.53	1.85 × 10^5^	–	–	–
(dpa)_2_CH^+^	−4.72	8.96 × 10^5^	–	–	–
*N* (*s*_N_)		13.31 (0.69)	13.86 (0.78)	15.16 (0.66)	16.63 (0.65)
p*K*_aH_(MeCN)		16.9	17.5^b)^	17.9	19.7
p*K*_aH_(DMSO)		6.5	7.8^b)^	8.3	11.0
p*K*_aH_(H_2_O)^c)^		8.8^c)^	9.9^c)^	>11^c)^	
*LB*		13.66	15.39	15.54	17.13

^a)^ From Ref. [119].

^b)^ From Ref. [129].

^c)^ In methanolic aqueous solution (1:5‐mixture), from Ref. [89].

Comparing the reactivity data for IChUs in the entries for each reference electrophile Ar_2_CH^+^ (horizontal direction in Table 1) shows that the second‐order rate constants *k*_2_ generally increase in the order **IU3** < **ITU3** < **ISeU3** < **ITeU3** [except for the reactions of (dma)_2_CH^+^ with **ITU3** and **ISeU3**].

**Figure 4:**
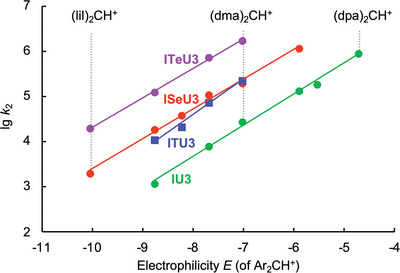
Linear correlations of second‐order rate constants (lg *k*_2_) for the reactions of **IU3**, **ITU3**, **ISeU3**, and **ITeU3** with Ar_2_CH^+^ **18** (in dichloromethane at 20 °C) with the electrophilicities *E* of **18**. The slopes of the linear correlations equal the nucleophile‐dependent sensitivity parameter *s*_N_. Intercepts on the abscissa reflect the nucleophilicity parameter *N* (*N* = –*E* at lg *k*_2_ = 0).

Figure 4 illustrates the linear correlations of the second‐order rate constants (lg *k*_2_) with the electrophilicities *E* of Ar_2_CH^+^ (**18**), which makes it possible to determine the Mayr nucleophilicity parameters *N* (and *s*_N_) for the DHPB‐derived IChUs **IU3**, **ITU3**, **ISeU3**, and **ITeU3**. Though the *s*_N_ = 0.78 of **ITU3** is slightly higher than the *s*_N_ values for the other IChUs (0.65 < *s*_N_ < 0.69), also the nucleophilicities *N* reflect the steadily increasing reactivity of IChUs when going from oxygen to heavier chalcogen atoms (Table 1), clearly indicating the dependence of nucleophilicity at the nitrogen on the vicinal chalcogen.

The ordering of IChU reactivities can be understood by the decrease in the electronegativity of the chalcogen (Pauling scale) when moving from oxygen (3.44) via sulfur (2.58) and selenium (2.55) to tellurium (2.1). Tellurium, the least electronegative element among the naturally abundant chalcogens, gives rise to the highest electron density at the neighboring nucleophilic nitrogen.

**Figure 5:**
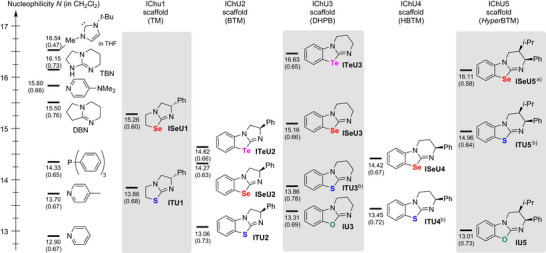
Nucleophilicity scale for IChUs and selected Lewis base catalysts (in dichloromethane if not mentioned otherwise, with *N* and *s*_N_ parameters from Ref. [120]). a) The reactivity of **ISeU5** was also studied in acetonitrile (*N* = 15.84, *s*_N_ = 0.57) and THF (*N* = 14.19, *s*_N_ = 0.77).^[^ ^95^ ^]^ b) From Ref. [119].

Analogous kinetic studies^[^ ^95^ ^]^ for tetramisole TM (**ITU1**) and its seleno homologue **ISeU1** as well as for benzotetramisole BTM (**ITU2**), SeBTM (**ISeU2**), and TeBTM (**ITeU2**) were carried out to gain insight in the effect of changing the ring size of the IChU. The same ring scaffold as in **IChU3** was retained in **ITU4** and **ISeU4** but supplemented by an additional phenyl group in the vicinity of the reactive nitrogen, which generates a chiral center at the IChUs but slightly lowers their reactivity. Finally, the so‐called *Hyper*BTM family (**IU5**, **ITU5**) was investigated kinetically, too, as this scaffold had previously proven to be among the most efficient IChU organocatalysts, e.g., for cycloadditions of allenoates.^[^ ^29^, ^30^, ^31^, ^32^ ^]^ Figure 5 provides an overview of the reactivity trends by embedding these nucleophiles in Mayr's nucleophilicity scale. With nucleophilicities *N* in the range 13 < *N* < 17, IChUs are located in an area of the *N* scale occupied by other typically used Lewis base catalysts, such as pyridines, triarylphosphines, DBN, TBN, or *N*‐heterocyclic carbenes. Though each change at the IChU scaffold induces a change in the absolute reactivities of these nucleophiles, the relative reactivity order within the IChU subclasses is general and follows the sequence O < S < Se < Te. Recognizing this general pattern facilitates the fine‐tuning of catalyst efficiency when optimizing IChU catalyzed reactions. For example, the generally higher nucleophilicity of ISeUs than of ITUs is in accord with recent experimental observations when using **ISeU5** as a catalyst.^[^ ^29^, ^30^, ^31^, ^32^, ^55^, ^73^, ^88^ ^]^ Extrapolating the significant increase in nucleophilic reactivity from **ISeU3** to the tellurium‐prototype **ITeU3**, as well as the observed increase from **ISeU2** to **ITeU2** to other catalyst scaffolds, indicates that further investigations into the field of isotellurourea catalysts could be worthwhile.

Success of polar organic reactions is not only dependent on Gibbs activation energies (the kinetic profile of a reaction), but also on the thermodynamics of adduct formation.^[^ ^121^, ^122^ ^]^ Further work, therefore, explored the trends in the thermodynamic driving force of covalent bond‐forming reactions at IChUs by investigating the Brønsted^[^ ^123^, ^124^, ^125^, ^126^ ^]^ and Lewis basicities^[^ ^121^, ^127^ ^]^ through experimental and quantum‐chemical techniques.

### Basicity of IChUs

#### Brønsted Basicity of IChUs

Surprisingly little is known about the Brønsted basicity of catalytically relevant IChUs.^[^ ^123^, ^124^, ^125^, ^126^ ^]^ Recently, Wiskur's group reported potentiometrically determined p*K*_aH_ values (that is, the p*K*_a_ of the conjugate acids) of achiral ITUs based on the BTM and DHPB scaffolds in aqueous media.^[^ ^89^, ^128^ ^]^ Furthermore, some of us have recently measured the p*K*_aH_ values of the isothioureas **ITU1–5** in DMSO and MeCN^[^ ^129^ ^]^ by utilizing the chemical shift imaging NMR method developed by Wallace and coworkers.^[^ ^130^, ^131^, ^132^ ^]^ In this work, we applied this NMR spectroscopic method to determine the p*K*_aH_ values of further IChUs in DMSO and MeCN (Supporting Information).

**Figure 6:**
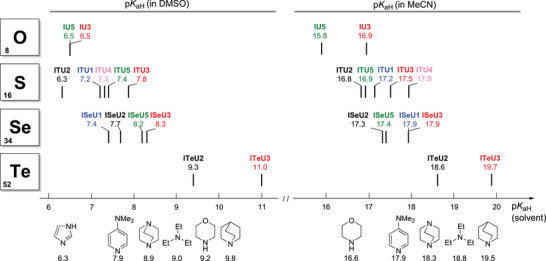
Brønsted basicities p*K*_aH_ (left: in DMSO, right: in acetonitrile) of IChUs (p*K*_aH_ for ITUs were determined in Ref. [129]).

The entries for DHPB‐derived IChU3 in Table 1 show that the chalcogen has a decisive effect on the Brønsted basicity of the IChUs (Ch = O, S, Se, and Te). Figure 6 depicts that the p*K*_aH_ orderings in DMSO as well as in MeCN solution follow the same trend as observed for nucleophilicity (O < S < Se < Te). Analogous p*K*_aH_ measurements showed consistent trends also within each of the **IChU1**–**5** scaffold subclasses. Only in the p*K*_aH_(DMSO) scale (Figure 6, left), the relative order of IChU1 and IChU2 changes when going from the ITU to the ISeU series.

Interestingly, the tellurium‐derivatives **ITeU2** and **ITeU3** were significantly more basic than the other IChUs, while maintaining the general structural trend of BTM derivatives (IChU2) being less Brønsted basic than IChU3. Noteworthy these ITeUs match or even exceed the Brønsted basicities of DABCO and quinuclidine in both acetonitrile and DMSO solution.^[^ ^123^, ^124^, ^125^, ^126^ ^]^ Hence, ITeUs may also be used as Brønsted bases in the future. On the other hand, high Brønsted basicity may deteriorate the performance of **ITeU3** as Lewis base catalyst because it could easily be deactivated by protonation.

#### Lewis Basicity and Methyl Cation Affinity

Since steric bulk affects N─H and N─C bond‐formation to a different extent, we sought to also study the Lewis basicity of IChUs toward C‐centered Lewis acids, which can be derived a) experimentally from equilibrium constants for the Lewis adduct formation with appropriate reference Lewis acids in a selected solvent or b) by quantum‐chemical calculations of carbocation affinities of a Lewis base.

**Figure 7:**
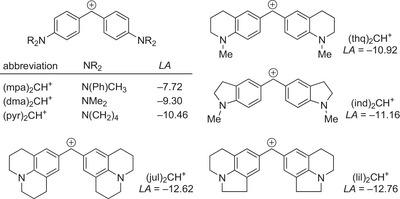
Lewis acidities (*LA*) of benzhydrylium ions Ar_2_CH^+^ used as reference Lewis acids in dichloromethane as reported in Ref. [127]).

(a) *Lewis basicity*. Previously, the Mayr group established a series of benzhydrylium ions as C‐centered Lewis acid references to construct a Lewis basicity scale in dichloromethane. Substituting experimentally determined equilibrium constants *K* (at 20 °C) and reported Lewis acidities *LA* for the benzhydrylium ions (Figure 7) in Equation (3) enables one to calculate the Lewis basicity (*LB*) of a Lewis base.^[^ ^121^, ^127^ ^]^

(3) $$\text{lg}\;K({20\;}^{\circ }C)=\text{LA}+\text{LB}$$

The reversibility of the formation of adducts **19** (Equation (4)), which was also apparent in the kinetics by significant positive intercepts in several of the *k*_obs_ versus [IChU] plots (Supporting Information), provided the opportunity to probe the thermodynamics of the C‐N bond‐forming reactions of IChUs with benzhydrylium ions as the reference Lewis acids in more detail.^[^ ^95^ ^]^

(4) $$\text{IChU}+{\mathrm{Ar}}_{2}{\mathrm{CH}}^{+}\left(18\right)⇌{\mathrm{Ar}}_{2}\text{CH-ICh}U^{+}\;\;\left(19\right)$$

By assuming the validity of the Beer–Lambert law for dilute solutions, equilibrium constants *K* (L mol^−1^) were determined in photometric titrations according to Equation (5) from the initial absorbance (*A*_0_) of the benzhydrylium ions **18** and the absorbance at equilibrium (*A*, Equation 5), that is, after addition of a certain amount of an IChU.^[^ ^109^, ^110^, ^111^, ^112^, ^113^, ^114^, ^115^, ^119^ ^]^

(5) $$K=\frac{\left[19\right]}{\left[18\right]\left[\mathrm{IChU}\right]}=\frac{A_{0}-A}{A\left[\mathrm{IChU}\right]}$$

**Figure 8:**
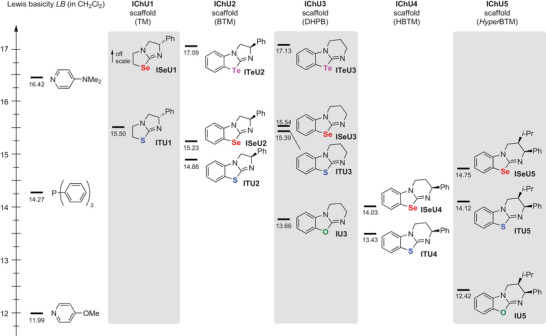
Lewis basicity scale for IChUs and further selected Lewis base catalysts (value for **ITU3** taken from Ref. 119, 127).

Averaged Lewis basicities (*LB*) for IChU3 molecules are gathered in Table 1 and for another eight IChUs in Figure 8. Similar to the observations made in the kinetic studies a clear trend based on the nature of the chalcogen atom is observed. The tellurium‐based catalyst **ITeU3** is more Lewis basic than its selenium counterpart **ISeU3**, followed by the sulfur derivative **ITU3** and the oxygen analogue **IU3** (Table 1), thus following the same trend (O < S < Se < Te) as observed for the nucleophilicity *N* (the same trend was observed for the investigated IChU2 compounds).

However, significant differences in comparison to the trends in the nucleophilicity scale are observed when the scaffold of the IChU catalysts is varied. For both the S‐ and the Se‐family, the most nucleophilic catalysts (**ITU5** and **ISeU5**) were not the most Lewis basic ones (Figure 8). Instead, the TM derivatives **ITU1** and **ISeU1** were the strongest Lewis bases upon comparison to the other scaffolds. Remarkably, the Lewis basicity LB of **ISeU1** could not be determined as the reactions between **ISeU1** and all the benzhydrylium ions **18** listed in Figure 7 proceeded to completion. These experiments indicate a significantly higher Lewis basicity of **ISeU1** than for any of the other catalysts studied in this work. Thus, the LB of **ISeU1** even exceeds that of **ITeU2** and **ITeU3**. The HBTM‐derived catalysts **ITU4** and **ISeU4** are the least Lewis basic members of the IChU families. It is also noteworthy that the highly nucleophilic *Hyper*BTM derivatives showed relatively weak Lewis basicity.

With regard to Lewis base catalysis, reversibility of C─N bond‐formation is required to release the catalyst when closing the catalytic cycle. It is of interest, therefore, that the outstandingly high Brønsted basicity of **ITeU3** is only partially reflected by its Lewis basicity. **ITeU3** is by three orders of magnitude more basic toward protons than **ISeU3** or **ISeU5**, but when equilibria for benzhydrylium adduct formation are compared this difference attenuates to only 1.5 units on the *LB* scale.

**Figure 9:**
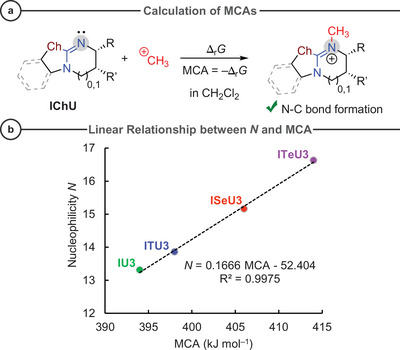
a) Definition equation used to calculate methyl cation affinities MCA of IChUs in dichloromethane at the SMD(DCM)/wB97XD/def2‐TZVP//PBE0‐D3BJ/def2‐TZVP level of theory. b) Linear relationship of IChU3 nucleophilicity (*N*) and MCA.

(b) *Carbocation affinities*. Furthermore, we calculated the methyl cation affinities (MCAs),^[^ ^95^, ^133^ ^]^ defined as the Gibbs free energies of the reaction of an IChU with the methyl cation ^+^CH_3_ in dichloromethane solution using DFT methods at the SMD(DCM)/wB97XD/def2‐TZVP//PBE0‐D3BJ/def2‐TZVP level of theory (Figure 9a; Table 2 gives an overview of the collected data).

**Table 2:** Quantum‐chemically calculated MCA and AIA (in kJ mol^−1^, DFT calculations were performed at the SMD(DCM)/wB97XD/def2‐TZVP//PBE0‐D3BJ/def2‐TZVP level of theory).

IChU	MCA	AIA	MCA–AIA
**IU3**	394	146	248
**IU5**	395	144	251
**ITU1**	402	171	231
**ITU2**	392	169	223
**ITU3**	398	167	231
**ITU4**	396	162	234
**ITU5**	400	166	234
**ISeU1**	412	182	230
**ISeU2**	402	178	224
**ISeU3**	406	177	229
**ISeU4**	403	172	231
**ISeU5**	408	176	232
**ITeU2**	409	192	217
**ITeU3**	414	197	217

For the DHPB‐derived IChU3 species, the nucleophilicity parameters *N*, calculated from the rate constants of IChU reactions with benzhydrylium ions, correlate linearly with MCA with excellent quality as shown in Figure 9b (*R* ^2^ = 0.9975). The quality of the relationships somewhat degrades when all 14 different IChU species from this work are included (Supporting Information, Figure S57).^[^ ^95^ ^]^ Yet, there is still a general trend of increased nucleophilicity with increasing MCA. Thus, the easily calculated MCAs may be used to estimate the reactivity of an unseen IChU when accepting roughly one *N* unit as uncertainty.

**Figure 10:**
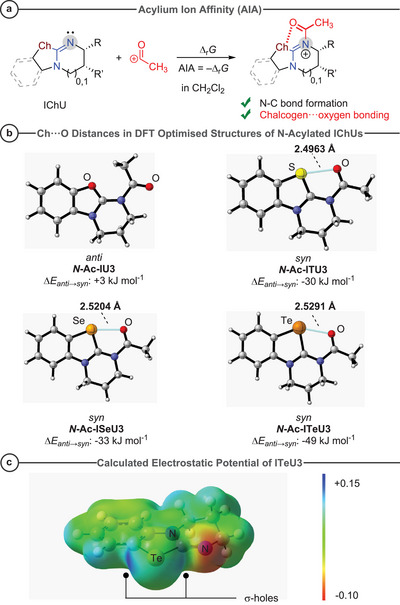
a) Definition equation used to calculate acylium ion affinities AIA of IChUs in dichloromethane at the SMD(DCM)/wB97XD/def2‐TZVP//PBE0‐D3BJ/def2‐TZVP level of theory. b) Optimized structures of *N*‐acyl‐IChU3 ions in the preferred *anti*‐ or *syn*‐conformation with chalcogen–oxygen distances. c) Electrostatic potential surface on the 0.01 au isodensity surface (SMD(DCM)/wB97XD/def2‐TZVP//PBE0‐D3BJ/def2‐TZVP) of **ITeU3** to highlight the σ‐holes (blue areas) at the tellurium atom.

To better account for reactions in which chalcogen–oxygen interactions are considered to stabilize intermediates in catalytic reactions (cf. **I**, **II**, and **III** in Scheme 1), we also analyzed acylium ion affinities (AIA, Table 2) of IChUs as defined in Figure 10a.

Analogous X‐ray analytical and quantum‐chemical studies on *N*‐acylated *Hyper*BTM compounds IChU5 (Ch = O, S, and Se) had revealed the existence of intramolecular 1,5‐O⋯Ch contacts in such ions.^[^ ^87^ ^]^ Accordingly, the optimized geometries of the *N*‐acylated IChU3 ions showed a preference for the *syn*‐conformer for chalcogen atoms with σ‐holes,^[^ ^134^ ^]^ that is, for S (**ITU3**), Se (**ISeU3**), and Te (**ITeU3**) (Figure 10b). Only the oxygen‐derivative **IU3** cannot benefit from 1,5‐O⋯Ch bonding and prefers by 3 kJ mol^−1^ the *anti*‐conformation, as previously observed for the *N*‐acylated **IU5**.^[^ ^87^ ^]^ Atomic distances of 1,5‐O⋯Ch neighbors would be expected to be relatively equal for O⋯S and O⋯Se but significantly larger for O⋯Te based on the van der Waals radii *r*_vdW_ of the chalcogen atoms (O: 1.50 Å; S: 1.89 Å; Se: 1.82 Å; Te: 1.99 Å).^[^ ^104^ ^]^ However, the computed atomic distances in the 1,5‐O⋯Ch bonds vary little when going from the S‐ via Se‐ to the Te‐derivative (Figure 10b).

Differences of MCA and AIA in Table 2 may illustrate the extra thermodynamic benefit of the isochalcogenouronium ions by O⋯Ch bonding. When compared to the MCA/AIA differences of **IU3** and **IU5** (248–251 kJ mol^−1^), which are unaffected by attractive O⋯Ch interactions, the systematically smaller MCA/AIA differences of ITUs and ISeUs (223–234 kJ mol^−1^) indicate a relatively constant favorable effect of 1,5‐O⋯Ch bonding to the stabilization of the *N*‐Ac‐IChU ions. When going from **ISeU3** to **ITeU3**, MCA increases by 8 kJ mol^−1^ but AIA by 12 kJ mol^−1^, which further boosts the *N*‐acyl‐**ITeU3** stabilization compared to S‐ or Se‐based IChU catalysts. The attractive intramolecular O⋯Te interaction in *N*‐acyl‐**ITeU3** can be rationalized by analyzing the electrostatic potential surface of **ITeU3** itself (Figure 10c).^[^ ^135^, ^136^, ^137^, ^138^, ^139^ ^]^ Pertinent positive values of the electrostatic potential at Te (blue color code in Figure 10c) in the ring plane visualize two σ‐holes at Te, one of is oriented to undergo 1,5‐O⋯Te bonding with the carbonyl oxygen of the *N5*‐acyl group.

### Increasing *N* and LB by Installing an Electron‐Donating Backbone Substituent

**Figure 11:**
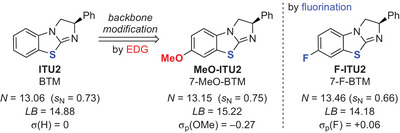
Effect of backbone‐substituents on the nucleophilicity and Lewis basicity of BTM‐derived IChUs.

After quantifying the significant influence of the chalcogen atom and the parent scaffold on the kinetic and thermodynamic properties of isochalcogenoureas, we set out to study the effect of installing an electron‐donating group at the backbone of the catalyst that has been reported to increase IChU reactivity.^[^ ^91^, ^128^, ^140^ ^]^ To gain first quantitative insights, the BTM‐type catalyst was modified by introducing an electron‐donating methoxy group (**MeO‐ITU2**; Figure 11). Interestingly, this revealed a relatively small influence of the substituent on the nucleophilicity (*N*) and the Lewis basicity (LB) as compared to the observed impact of the chalcogen atom and the general catalyst scaffold.^[^ ^95^ ^]^ The introduction of fluorine in the same position (**F‐ITU2**; a catalyst that recently outperformed **ITU2** in α‐fluorination reactions^[^ ^76^ ^]^) led to a slightly increased nucleophilicity combined with reduced Lewis basicity. Upon comparing the almost similar Hammett substituent constants *σ* of p‐F and H^[^ ^141^ ^]^ these observations cannot solely be explained or correlated by simple electronic effects. Nevertheless, these results indicate that such backbone modifications, which have reported effects in catalysis,^[^ ^76^, ^91^, ^128^, ^140^ ^]^ also impact the nucleophilicity and Lewis basicity of these catalysts.

### Acylation Reactions

**Scheme 3:**
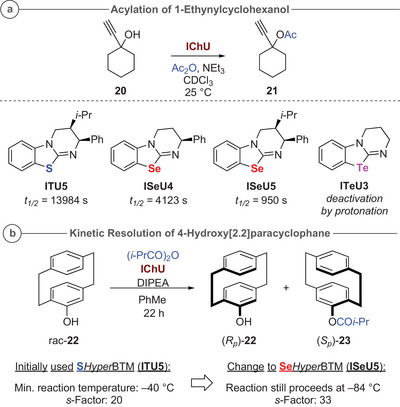
Applications of sulfur‐, selenium‐ and tellurium‐containing catalysts in acylation reactions: a) In situ NMR‐monitoring of catalytic acylation of 1‐ethynylcyclohexanol **20** using acetic anhydride and b) Acylative kinetic resolution of 4‐hydroxy[2.2]paracyclophane **22**.^[^ ^95^ ^].^

In our study introducing Se*Hyper*BTM (**ISeU5**) and O*Hyper*BTM (**IU5**), it was demonstrated that the Se‐derivative allows for significantly faster catalytic asymmetric acyl transfer reactions than the parent S‐based **ITU5**, while **IU5** was much less active.^[^ ^87^ ^]^ To further probe the chalcogen impact on the catalytic acyl transfer potential of IChUs, acylation of 1‐ethynylcyclohexanol **20** using acetic anhydride was utilized as a benchmark test reaction due to the recognized slow rate of reaction of this tertiary alcohol. Online NMR spectroscopic analysis was used to monitor the acylation reactions catalyzed by selected IChUs (Scheme 3a).^[^ ^142^ ^]^

Comparing the Se‐containing catalysts **ISeU4‐5** with HyperBTM (**ITU5**), initial reaction rates were fast, in line with observations with benzhydrylium ions. The most nucleophilic derivative **ISeU5** (*N* = 16.10) gave the shortest time to 50% conversion (*t*_1/2_ = 950 s) and also, when using **ISeU4** (*N* = 14.42) 50% conversion could be reached faster (*t*_1/2_ = 4123 s) compared to HyperBTM (*t*_1/2_ = 13 984 s). Interestingly, when employing the even more nucleophilic **ITeU3** (*N* = 16.63), the reaction never reached any notable conversion. This observation can be explained by the increased Brønsted basicity of **ITeU3** (Figure 6) which, under the reaction conditions depicted in Scheme 3a, precipitates immediately in its protonated form and thus cannot act as a catalyst anymore.

To exploit the higher reactivity of **ISeU5** for asymmetric acyl‐transfer reactions this catalyst was tested in our recently developed acylative kinetic resolution of 4‐hydroxy[2.2]paracyclophane **22** (Scheme 3b).^[^ ^13^ ^]^ Originally, **ITU5** was identified as the optimal catalyst, giving a selectivity factor *s* of 20 when carrying out the reaction at −40 °C. However, utilizing the higher nucleophilicity (*N*) and Lewis basicity (*LB*) of **ISeU5** allowed us to conduct this kinetic resolution at lower temperature (−84 °C) resulting in a significantly higher *s*‐factor of 33.^[^ ^143^ ^]^

### Reactivity Toward Allenoates

**Figure 12:**
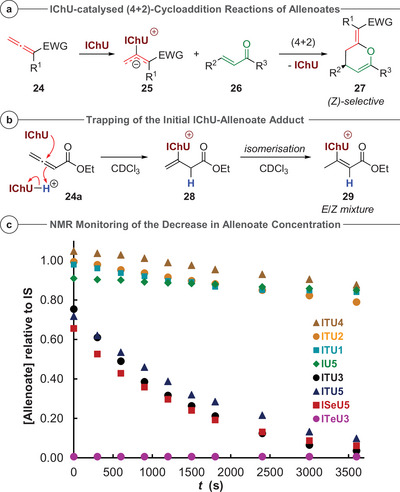
a) Recently developed IChU‐catalyzed reaction between allenoates and Michael acceptors; b) Trapping of the catalyst‐allenoate adducts by immediate protonation and c) NMR monitoring (500 MHz, 298 K, CDCl_3_) of the decrease of allenoate concentration (resonance at 5.64 ppm) relative to mesitylene as internal standard (IS) upon reaction with different catalysts. Spectra were recorded after approx. 5 min needed for mixing and setting up of the NMR experiment (locking and shimming).^[^ ^95^ ^].^

Recent work has shown that isochalcogenourea catalysts can activate allenoates **24**.^[^ ^29^, ^30^, ^31^, ^32^ ^]^ Here, the initially formed catalyst‐allenoate adducts **25** undergo (4 + 2)‐cycloaddition reactions with different Michael acceptors **26** with high levels of stereocontrol (Figure 12a). For these transformations, elevated temperatures (≥ 80 °C) were needed for the reactions to proceed. Extensive computational studies suggested that the initial addition of the catalyst to the allene is a reversible endergonic step with a high activation energy, rationalizing the required higher reaction temperatures.^[^ ^29^, ^30^, ^31^, ^32^ ^]^ During optimization of these reactions **ISeU5** was more catalytically active (giving highest yield and highest e.r.) than **ITU5**. The achiral **ITU3** allowed for comparable yields to **ITU5**, while the other ITUs were clearly less reactive (or did not yield any product at all). To further investigate this first addition step in more detail, the addition of the IChUs to allenoates **24** was monitored by ^1^H‐NMR spectroscopy. Gratifyingly, when providing a proton source instead of a Michael acceptor, the catalyst‐allenoate adducts were trapped even at room temperature. In order to prevent concurrent protonation of the catalyst by Brønsted acids, we employed a 1:1:1 ratio of allenoate, catalyst and the hydrochloride salt of the same catalyst in CDCl_3_ (Figure 12b).^[^ ^144^, ^145^ ^]^

Using this method, the decrease in allenoate concentration (versus an internal standard) during the trapping reaction was monitored by online ^1^H‐NMR spectroscopy.^[^ ^95^ ^]^ In the beginning, the formation of adduct **28** bearing a terminal olefin is observable, followed by isomerization to an *E*/*Z* mixture of adduct **29** and accompanying degradation of the allenoate and allenoate/catalyst adduct. Due to ambiguous kinetics, no reasonable 2^nd^ order rate constants *k* could be determined, but a clear difference in reaction rates was observed (Figure 12c).

The formation of adducts **28** and **29** was fastest with Se*Hyper*BTM (**ISeU5**) followed by *Hyper*BTM (**ITU5**) and DHPB **ITU3**. For all of those catalysts, significant allenoate consumption was observed during the time needed for mixing and setting up the NMR experiment (approx. 5 min). When using TeDHPB (**ITeU3**), the addition to the allenoate was so fast that it could not be followed by these NMR techniques anymore. Already in the first spectrum taken, the allenoate resonance at 5.64 ppm was not observable anymore as all allenoate had converted to adducts **28** and **29**, accompanied by some degradation (testing **ITeU3** in racemic (4 + 2)‐cycloadditions of allenoates^[^ ^30^ ^]^ showed that this novel Lewis base is a potent catalyst for such transformations giving the racemic products in comparable yields as the commonly used (Se)HyperBTM).

All other catalysts reacted significantly slower. Thus, the more nucleophilic and more Lewis basic catalysts perform better in allenoate activation reactions. The nucleophilicity parameter *N* along with the Lewis basicity LB can thus be used to predict whether an isochalcogenourea derivative might be a suitable candidate to catalyze similar reactions. However, the *N* parameter cannot be the sole explanation for the observed reactivity differences as TM (**ITU1**), which is equally nucleophilic as DHPB (**ITU3**), did not perform well in these allenoate reactions.^[^ ^29^ ^]^

## Conclusion

Since the pioneering work of Birman,^[^ ^6^, ^8^, ^9^, ^10^ ^]^ the use of bi‐ and tricyclic IChUs as organocatalysts has been established as a powerful tool to activate electrophiles for subsequent transformations. Yet, predicting the optimum IChU catalyst for a given transformation is almost impossible as various catalyst properties combined with stabilizing or destabilizing interactions in transition states and reaction intermediates impact the overall performance. In this study, we selected 14 IChUs with systematically varied structural key parameters, that is, the ring size, the substituents at the rings, and the chalcogen atom. Studies on the influence of the ring size and substituent variation were based on the established IChU catalyst motifs of TM (IChU1), BTM (IChU2), DHPB (IChU3), HBTM (IChU4), and *Hyper*BTM (IChU5). In addition, the series of chalcogens was extended from the known O‐, S‐, and Se‐members to also include two previously unknown tellurium members (**ITeU2‐3**), for which we developed a synthetic method in this work. With this range of IChU catalysts available, we determined their nucleophilicities on the Mayr scale by kinetic analysis of IChU reactions with benzhydrylium ions (reference electrophiles) in dichloromethane at 20 °C, which were monitored by stopped‐flow photometric methods. Some of these reactions showed reversibility, which prompted us to also investigate the equilibrium reactions of IChU/benzhydrylium reactions to calculate the Lewis basicities of the IChUs. Further quantum‐chemical calculations by using DFT methods provided the methyl cation and acylium ion affinities of IChUs and indicated a favorable geometry of the *N*‐acyl‐**ITeU3** ion that will be used in the future to leverage σ‐hole catalysis to generate geometrically constrained reactive intermediates. In general, the observed trends from the kinetic and thermodynamic property studies correlate with the decreasing electronegativity of the chalcogen atom (O > S = Se > Te) which increasingly enhances the electron density, basicity, or nucleophilicity at the reactive nitrogen of the IChU. First results of organocatalytic benchmark reactions are encouraging and indicate that exploiting chalcogen variation in IChUs will be a powerful strategy to adapt the activating potential of benchstable IChU organocatalysts to the requirements of a given transformation.

## Supporting Information

The authors have cited additional references within the Supporting Information.^[^ ^146^, ^147^, ^148^, ^149^, ^150^, ^151^, ^152^, ^153^, ^154^, ^155^, ^156^, ^157^, ^158^, ^159^, ^160^, ^161^, ^162^, ^163^, ^164^, ^165^, ^166^, ^167^, ^168^, ^169^, ^170^, ^171^, ^172^, ^173^, ^174^, ^175^, ^176^, ^177^, ^178^, ^179^, ^180^ ^]^

## Conflict of Interests

The authors declare no conflict of interest.

## Supporting information



Supporting Information

## Data Availability

The data that support the findings of this study are available in the Supporting Information of this article. The underpinning research data from the University of St Andrews that supports this publication is also available at https://doi.org/10.17630/f1e7f083-2a9e-401c-921a-ecdd68b95ed2.
