# Supplementary material for: From Oxygen to Tellurium: The Impact of the Chalcogen on Nucleophilicities and Basicities of Isochalcogenourea Catalysts
Source: Angew Chem Int Ed Engl. 2025 Oct 23;64(48):e202514865. doi: 10.1002/anie.202514865 (PMC12643354; doi:10.1002/anie.202514865)
Supplement: Supplementary file 1 — Supporting Information [file ANIE-64-e202514865-s001.pdf]

Supplementary Information

From Oxygen to Tellurium: The Impact of the Chalcogen on Nucleophilicities and Basicities of Isochalcogenourea Catalysts

Lotte Stockhammer,[a] Kevin Kasten,[b] Andreas Eitzinger,[a,c] Lukas S. Vogl,[a] Magdalena Piringer,[a] David Weinzierl,[a] Armin R. Ofial,*[c] Andrew D. Smith,*[b] and Mario Waser*[a]

[a] Institute of Organic Chemistry Johannes Kepler University Linz, Altenbergerstrasse 69, 4040 Linz (Austria)

E-Mail: mario.waser@jku.at

[b] EaStCHEM, School of Chemistry, University of St Andrews, KY169ST St Andrews, Fife, (UK)

E-Mail: ads10@st-andrews.ac.uk

[c] Department Chemie, Ludwig-Maximilians-Universität München, Butenandtstr. 5–13, 81377 München (Germany)

E-Mail: ofial@lmu.de

Table of Contents

1. General Information 4

1.1 Linz 4

1.2 St Andrews 5

1.3 Munich 5

2. Synthesis of Isoureas 6

2.1 General Procedure A for the Synthesis of 2-Aminobenzoxazoles 6

2.2 General Procedure B for the Synthesis of Isourea Catalysts 6

2.3 Product Characterization 6

3. Synthesis of Isothioureas 8

4. Synthesis of Benzannulated Isoselenoureas 11

4.1 General Procedure C for the Synthesis of Formamides 11

4.2 General Procedure D for the Synthesis of Isoselenocyanates 11

4.3 General Procedure E for the Synthesis of Isoselenourea Catalysts 12

4.4 Product Characterization 13

5. Synthesis of SeTM 16

6. Synthesis of Isotelluroureas 18

6.1 Synthesis of the Ditelluride Precursor (14) 18

6.2 General Procedure F for the Synthesis of Protected Amino Alcohols 19

6.3 General Procedure G for the Synthesis of Isothiocyanates 19

6.4 General Procedure H for the Synthesis of 2-Aminobenzotellurazoles 20

6.5 General Procedures I for the Deprotection Reaction 20

6.6 General Procedure J for the Synthesis of Isotelluroureas 21

6.7 Characterisation Data 21

6.8 Optimization of the Cyclization Reaction between Ditelluride 14 and Isothiocyanate 15 25

7. Single Crystal X-Ray Diffraction of TeDHPB (ITeU3) 27

8. Reactions between IChUs and Benzhydrylium Ions: Product Studies with Representative Examples 29

9. Reactivity towards Allenoates 31

9.1 General Procedure F for the Synthesis of Catalyst Hydrochloride Salts 31

9.2 General Procedure G for the Synthesis of the Free Base Catalysts 31
9.3 Comparison of NMR data for Free Base Catalysts and their Hydrochloride Salts 31
9.4 Trapping Experiments 38
10. Reactivity in Acylation Reactions 48
10.1 NMR Monitoring the Acylation of 1-ethynylcyclohexan-1-ol 48
10.2 Acylative Kinetic Resolution of 4-Hydroxy[2.2]paracyclophane 69
11. Kinetic Studies 71
11.1 General Information 71
11.2 Kinetic Studies in Dichloromethane 72
11.3 Solvent Effects 101
12. Equilibrium Constants 105
13. Determination of pK_a Values for Isochalcogenoureas 143
13.1 General Information 143
13.2 Datapoint Selection 144
13.3 Sample Preparation 145
13.4 Indicator and Acid Data 146
13.5 Titration Data 147
14. DFT Calculations 148
14.1 General Information 148
14.2 Methyl Cation Affinities and Acyl Ion Affinities 149
14.3 Summary of DFT-Calculated Data 153
15. References 182
16. NMR Spectra for Novel Compounds 185
17. NMR Spectra: CSI Experiments for pKa determination in MeCN 237
18. NMR Spectra: CSI Experiments for pKa determination in DMSO 253

1. General Information

1.1 Linz

NMR spectra were recorded on a Bruker Avance III 300 MHz spectrometer with a broad band observe probe and a sample changer for 16 samples, a Bruker Avance DRX 500 MHz spectrometer or a Bruker Avance III 700 MHz spectrometer with an Ascend magnet and TCI cryoprobe, which are property to the Austro Czech NMR Research Center "RERI uasb". All NMR spectra were referenced on the solvent residual peak ($CDCl_3$: *δ* 7.26 ppm for ^1H NMR and *δ* 77.16 ppm for ^{13}C NMR; DMSO: *δ* 2.50 ppm for ^1H NMR and *δ* 39.52 ppm for ^{13}C NMR; MeOD: *δ* 3.31 ppm for ^1H NMR and *δ* 49.00 ppm for ^{13}C NMR; MeCN: *δ* 1.94 ppm for ^1H NMR and *δ* 118.26 ppm for ^{13}C NMR; D_2O: *δ* 4.79 ppm for ^1H NMR; DCM: *δ* 5.32 ppm for ^1H NMR and *δ* 53.80 ppm for ^{13}C NMR). NMR data are reported as follows: chemical shift (δ ppm), multiplicity (s = singlet; d = doublet; t = triplet; q = quartet; m = multiplet; br = broad; app. = apparent), coupling constants (Hz) and integrals. High resolution mass spectra were obtained using an Agilent QTOF 6520 with ESI source. Purified by chromatography refers to normal phase chromatography using Davisil LC 60A 70–200 MICRON silica gel. Thin layer chromatography was performed on Macherey-Nagel pre-coated TLC plates (silica gel, 60 F_{254}, 0.20 mm, ALUGRAM® Xtra SIL). TLC plates were visualized under a 254 nm UV lamp or via staining with $KMnO_4$. Enantiomeric ratios (*e.r.*) for the cyclophane derivatives were determined by HPLC analysis using a Dionex Summit HPLC system with a CHIRAL ART Cellulose-SB (250 × 4.6 mm, 5 µm) chiral stationary phase. Dry solvents were taken from an mBRAUN SPS solvent purifier. All reactions were run under an Argon atmosphere unless otherwise stated. All chemicals were purchased from commercial suppliers and used without further purification unless otherwise stated.

1.2 St Andrews

^{1}H nuclear magnetic resonance (NMR) spectra were acquired on either a Bruker Avance II 400 spectrometer at 298 K in the deuterated solvent stated with spectra referenced to the solvent residue signal reported in literature (see above). For analysis of NMR-spectra MestReNova and tools therein were used.

1.3 Munich

Kinetic measurements were performed by employing stopped-flow UV/Vis photometry on Applied Photophysics SX.20 systems. The temperature (20.0 ± 0.2 °C) was maintained constant by using circulating bath cryostats. All solutions were prepared by using dry solvents and were kept under an atmosphere of dry nitrogen. Equilibrium measurements were carried out using a J&M TIDAS diode array spectrophotometer, which was controlled by TIDASDAQ3 (v3) software and connected to a Hellma 661.502-QX quartz Suprasil immersion probe (light path d = 5 mm) via fiber optic cables and standard SMA connectors.

High-resolution mass spectrometry (HRMS) was performed on either a Thermo Finnigan LTQ FT Ultra Fourier transform ion cyclotron resonance spectrometer or a Thermo Finnigan LTQ Orbitrap XL (res. = 100 000 at m/z = 400, 5 kV, 250 °C). Samples were ionized by electron spray ionisation (ESI).

2. Synthesis of Isoureas

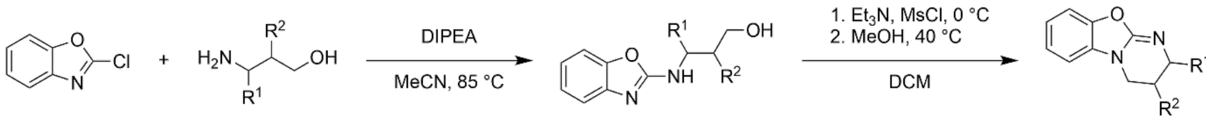

2.1 General Procedure A for the Synthesis of 2-Aminobenzoxazoles

According to literature[1], 2-chlorobenzoxazole (1.0 eq), the respective amino alcohol (2 eq) and DIPEA (4.0 eq) were dissolved in MeCN (1.0 mol L^{-1}) and heated to reflux for 16 h. MeCN was removed on the rotary evaporator and the residue was taken up in an Et_2O. It was washed with water and then the aqueous phase was extracted with Et_2O three times. The combined organic layers were dried over Na_2SO_4, filtered and concentrated. The crude product was triturated with little amounts of Et_2O to give the 2-aminobezoxazoles as white solids.

2.2 General Procedure B for the Synthesis of Isourea Catalysts

According to literature[1], the 2-aminobenzoxazole (1 eq) obtained from the first step and Et_3N (3 eq) were dissolved in anhydrous DCM (0.15 mol L^{-1}). Then, the mixture was cooled to 0 °C and MsCl (1.5 eq) was added dropwise. The mixture was stirred at r.t. for 1 h after which MeOH (10 eq) was added and the mixture was heated to reflux for 16 h. After cooling to r.t., the mixture was washed with water three times. The organic layer was dried over Na2SO4, filtered and concentrated. The crude was triturated with Et_2O to give isoureas as white solids.

2.3 Product Characterization

3-(Benzo[d]oxazol-2-ylamino)propan-1-ol

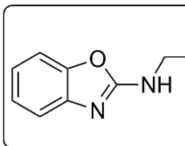

3a

Following general procedure A, 2-aminobenzoxazole was obtained as a white powder in a yield of 80% (20 mmol scale).

^{1}H-NMR (600 MHz, $CDCl_3$, 298 K) δ / ppm = 7.34 (d, J = 7.7 Hz, 1 H, Ar-**H**), 7.23 (d, J = 7.9 Hz, 1 H, Ar-**H**), 7.16 (td, J_1 = 0.9 Hz, J_2 = 7.7 Hz, 1 H, Ar-**H**), 7.03 (td, J_1 = 1.1 Hz, J_2 = 7.9 Hz, 1 H, Ar-**H**), 5.49 (s, 1 H, -O**H**), 4.02 (br. s, 1 H, -N**H**), 3.75 (t, J = 5.6 Hz, 2 H, -C**H₂**), 3.66 (m, 2 H, -C**H₂**), 1.85 (m, 2 H, -C**H₂**).

^{13}C-NMR (150 MHz, $CDCl_3$, 298 K) δ / ppm = 162.9 (1 C, -**C**=N), 148.6 (1 C, -$\mathbf{C}_{Ar}$), 142.5 (1 C, -$\mathbf{C}_{Ar}$), 124.1 (1 C, -$\mathbf{C}_{Ar}$), 121.1 (1 C, -$\mathbf{C}_{Ar}$), 116.4 (1 C, -$\mathbf{C}_{Ar}$), 108.9 (1 C, -$\mathbf{C}_{Ar}$), 59.1 (1 C, -$\mathbf{C}H_2$), 40.1 (1 C, -$\mathbf{C}H_2$), 32.8 (1 C, -$\mathbf{C}H_2$).

(*R*)-2-((*R*)-(Benzo[d]oxazol-2-ylamino)(phenyl)methyl)-3-methylbutan-1-ol

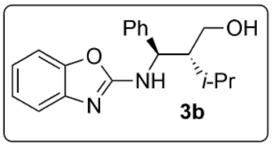

Following general procedure A, 2-aminobenzoxazole was obtained as a white powder in a yield of 73% (20 mmol scale). Spectral data was in accordance with literature.[1]

^{1}H-NMR (400 MHz, $CDCl_3$, 298 K) δ / ppm = 7.46-7.44 (m, 2 H, Ar-**H**), 7.37-7.33 (m, 2 H, Ar-**H**), 7.31-7.27 (m, 2 H, Ar-**H**), 7.21 (d, J = 7.9 Hz, 1 H, Ar-**H**), 7.12 (td, J_1 = 1.1 Hz, J_2 = 7.7 Hz, 1 H, Ar-**H**), 6.99 (td, J_1 = 1.1 Hz, J_2 = 7.7 Hz, 1 H, Ar-**H**), 6.56 (br. d, J = 9.0 Hz, 1 H, -N**H**), 5.35 (dd, J_1 = 4.3 Hz, 1 H, -NC**H**), 3.83-3.81 (m, 1 H, -C**H₂**), 3.60 (t, J = 10.5 Hz, 1 H, -C**H₂**), 3.22 (br. s, 1 H, -O**H**), 2.11-2.05 (m, 1 H, -C**H**), 1.74 (sept., J = 6.8 Hz, 1 H, -C**H**), 1.08 (d, J = 6.8 Hz, 3 H, -C**H₃**), 0.85 (d, J = 6.8 Hz, 3 H, -C**H₃**).

3,4-Dihydro-2H-benzo[4,5]oxazolo[3,2-a]pyrimidine (ODHPB, IU3)

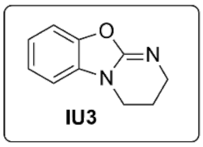

Following general procedure B, ODHPB **IU3** was obtained as a white crystalline solid in a yield of 70% (15 mmol scale).

Melting point: 81.4-83.2 °C

^{1}H-NMR (600 MHz, $CDCl_3$, 298 K) δ / ppm = 7.08-7.04 (m, 2 H, Ar-**H**), 6.94 (td, J_1 = 1.1 Hz, J_2 = 7.8 Hz, 1 H, Ar-**H**), 6.74 (d, J = 7.6 Hz, 1 H, Ar-**H**), 3.78 (t, J_1 = 6.0 Hz, 2 H, -C**H₂**), 3.59 (t, J = 5.6 Hz, 2 H, -C**H₂**), 1.97 (quint., J = 5.8 Hz, 2 H, -C**H₂**).

^{13}C-NMR (150 MHz, $CDCl_3$, 298 K) δ / ppm = 154.1 (1 C, -N=**C**), 144.5 (1 C, **C**$_{Ar}$), 133.1 (1 C, **C**$_{Ar}$), 123.2 (1 C, **C**$_{Ar}$), 121.1 (1 C, **C**$_{Ar}$), 109.2 (1 C, **C**$_{Ar}$), 105.6 (1 C, **C**$_{Ar}$), 43.9 (1 C, -**C**H_2), 40.8 (1 C, -**C**H_2), 20.1 (1 C, -**C**H_2).

HRMS (ESI-TOF): *m/z*: $[M+H]^+$ calcd for $C_{10}H_{11}N_2O^+$: 175.0866, found 175.0868.

3-isopropyl-2-phenyl-3,4-dihydro-2H-benzo[4,5]oxazolo[3,2-a]pyrimidine (O*Hyper*BTM, IU5)

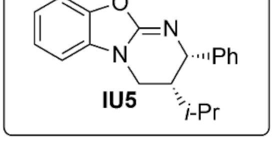

Following general procedure B, O*Hyper*BTM **IU5** was obtained as a white crystalline solid in a yield of 55% (15 mmol scale). Spectral data was in accordance with literature.[1]

^{1}H-NMR (400 MHz, $CDCl_3$, 298 K) δ / ppm = 7.32-7.25 (m, 2 H, Ar-**H**), 7.22-7.19 (m, 3 H, Ar-**H**), 7.16-7.09 (m, 2 H, Ar-**H**), 7.01 (td, J_1 = 1.2 Hz, 1 H, J_2 = 7.8 Hz, 1 H, Ar-**H**), 6.83 (d, J = 7.7 Hz, 1 H, Ar-**H**), 4.95 (d, J = 3.2 Hz, 1 H, -C**H**), 3.88 (ddd, J_1 = 1.5 Hz, J_2 = 5.4 Hz, J_3 = 11.5 Hz, 1 H, -C**H₂**), 3.43 (t, J = 11.3 Hz, 1 H, -C**H₂**), 1.98-1.90 (m, 1 H, -C**H**), 1.35-1.24 (m, 1 H, -C**H**), 1.13 (d, J = 6.5 Hz, 3 H, -C**H₃**), 0.83 (d, J = 6.7 Hz, 3 H, Ar-**H**).

3. Synthesis of Isothioureas

Isothiourea catalysts TM (CAS 16595-80-5), BTM (CAS 885051-07-0), HBTM (CAS 1316861-19-4), and HyperBTM (CAS 1203507-02-1) are commercially available and were used as obtained from the provider without further purification.

DHPB

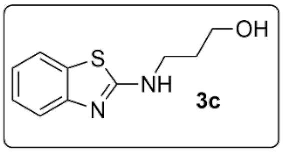

According to literature[2], 2-chlorobenzothiazole (1.0 eq) and aminopropamol (1.1 eq) were suspended in chlorobenzene (0.3 mol L^{-1}). DIPEA (4.0 eq) was added, and the mixture was heated to 140°C overnight. After cooling to r.t. the reaction was quenched with water and extracted with EtOAc three times. The combined organic layers were dried with Na_2SO_4, filtered and concentrated on the rotavapor. The crude products were purified by column chromatography on silica (heptanes/EtOAc 5/1 followed by 2/1 and pure EtOAc).

Performing the reaction on a 30 mmol scale gave the product as a white solid in a yield of 89%. Different scales from 10-100 mmol gave similar results. Spectral data was in accordance with literature, albeit chemical shifts of aliphatic protons may vary from experiment to experiment.[12]

^{1}H-NMR (300 MHz, $CDCl_3$, 298 K) δ / ppm = 7.55 (dd, J_1 = 1.2 Hz, J_2 = 7.9 Hz, 1 H, Ar-**H**), 7.52 (dd, J_1 = 1.2 Hz, J_2 = 8.2 Hz, 1 H, Ar-**H**), 7.31-7.26 (m, 1 H, Ar-**H**), 7.09 (td, J_1 = 1.2 Hz, J_2 = 7.6 Hz, 1 H, Ar-**H**), 3.76-3.68 (m, 4 H, -C**H₂**), 1.89-1.81 (m, 2 H, -C**H₂**).

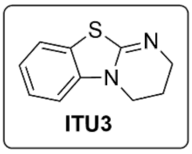

According to literature[2], the 2-aminobenzothiazole (1.0 eq) obtained in the previous step was dissolved in anhydrous DCM (0.1 mol L^{-1}) and cooled to 0 °C. Et_3N (3.0 eq) and MsCl (1.5 eq) were added dropwise simultaneously. After complete addition, the mixture was stirred another 90 min at r.t. After this, MeOH (1.5 eq) and another portion Et_3N (3.0 eq) was added, and the mixture was heated to reflux overnight. After cooling to r.t., water was added, and it was extracted with DCM three times. The combined organic layers were dried over Na_2SO_4, filtered and concentrated. The residue was then recrystalized from EtOAc. In case of impure samples, a further column chromatographic step is necessary (using a heptanes/EtOAc gradient, with the catalyst eluting using an 1/1 mixture or pure EtOAc).

DHPB (**ITU V**) was obtained as an off-white solid in a yield of 77% when performing the reaction on a 20 mmol scale. Spectral data was in accordance with literature.[2]

^{1}H-NMR (300 MHz, $CDCl_3$, 298 K) δ / ppm = 7.26 (dd, J = 1.1 Hz, J_2 = 7.6 Hz, 1 H, Ar-**H**), 7.17 (td, J_1 = 1.0 Hz, J_2 = 7.9 Hz, 1 H, Ar-**H**), 6.97 (td, J_1 = 1.0 Hz, J_2 = 7.6 Hz, 1 H, Ar-**H**), 6.72 (d, J = 7.9 Hz, 1 H, Ar-**H**), 3.76 (t, J = 6.1 Hz, 2 H, -C**H₂**), 3.56 (t, J = 5.6 Hz, 2 H, -C**H₂**), 2.01 (m, 2 H, -C**H₂**).

F-ITU2 (7-F-BTM)

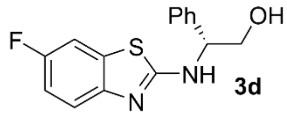

3d

With slight adaptions to literature[2], fluoro-2-chlorobenzothiazole (5.3 mmol, 1.00 g, 1 eq) and (*R*)-phenyl glycinol (5.3 mmol, 0.73 g, 1 eq) were suspended in chlorobenzene (17 mL, 0.3 mol L^{-1}). DIPEA (19.5 mmol, 3.4 mL, 3.7 eq) was added and the mixture was stirred at 140 °C for 72 h. After cooling to r.t., the mixture was washed with water twice. The organic layer was dried over Na_2SO_4, filtered and concentrated. The crude product was purified by column chromatography (silica, gradient heptanes/EtOAc 20/1, 10/1, 5/1, 2/1 pure EtOAc). 2-aminobenzothiazole **3d** was obtained in a yield of 0.66 g (43%) as a brownish solid.

^{1}H-NMR (300 MHz, $CDCl_3$, 298 K) δ / ppm = 7.43-7.31 (m, 7 H, Ar-**H**), 7.22 (dd, J_1 = 2.6 Hz, J_2 = 8.1 Hz, 1 H, Ar-**H**), 6.99 (td, J_1 = 2.6 Hz, J_2 = 9.0 Hz, 1 H, Ar-**H**), 4.85 (dd, J_1 = 4.1 Hz, J_2 = 6.4 Hz, 1 H, -C**H**), 4.02-3.91 (m, 2 H, Ar-**H**).

^{13}C-NMR (75 MHz, $CDCl_3$, 298 K) δ / ppm = 167.2 (1 C, -N=**C**), 158.5 (d, *J* = 240.8 Hz, 1 C, **C**$_{Ar}$-F), 148.1 (d, *J* = 1.9 Hz, 1 C, **C**$_{Ar}$), 138.5 (1 C, **C**$_{Ar}$), 131.4 (d, *J* = 10.8 Hz, 1 C, **C**$_{Ar}$), 129.1 (2 C, **C**$_{Ar}$), 128.5 (1 C, **C**$_{Ar}$), 127.0 (2 C, **C**$_{Ar}$), 119.5 (d, *J* = 8.8 Hz, 1 C, **C**$_{Ar}$), 113.9 (d, *J* = 23.9 Hz, 1 C, **C**$_{Ar}$), 107.7 (d, *J* = 27.2 Hz, 1 C, **C**$_{Ar}$), 67.9 (1 C, -**C**H), 62.0 (1 C, -**C**H_2).

^{19}F-NMR (282 MHz, $CDCl_3$, 298 K) δ / ppm = -120.7 (m, 1 F, Ar-**F**).

F-ITU2

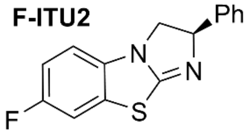

Adapting a literature procedure[2], 2-aminobenzothaiazole **3d** from the first step (2.3 mmol, 0.66 g, 1 eq) was dissolved in anhydrous DCM (15 mL, 0.15 mol L^{-1}) and cooled to 0 °C.) MsCl (3.4 mmol, 0.267 mL, 1.5 eq) was added dropwise. The mixture was warmed to r.t. over 90 min after which MeOH (23 mmol, 0.930 mL, 10 eq) and Et_3N (23 mmol, 3.8 mL, 10 eq) were added. The mixture was heated to reflux for 16 h. After cooling to r.t., the mixture was washed with 1.0 M NaOH once. The aqueous layer was then extracted with DCM three times. The combined organic layers were washed with brine, dried over Na_2SO_4, filtered and concentrated. The crude product was purified by column chromatography (silica, heptanes/EtOAc 10/1, 5/1, 2/1 followed by pure EtOAc) and then triturated with an EtOAc/pentane mixture to yield 7-F-BTM (**F-ITU2**) as a slightly yellow powder in a yield of 0.40 g (64%).

Melting point: 116-119 °C

^{1}H-NMR (300 MHz, $CDCl_3$, 298 K) δ / ppm = 7.38-7.27 (m, 2 H, Ar-**H**), 7.07 (dd, J_1 = 2.5 Hz, 1 H, Ar-**H**), 6.90 (td, J_1 = 2.5 Hz, J_2 = 8.8Hz, 1 H, Ar-**H**), 6.58 (dd, J_1 = 4.3 Hz, J_2 = 8.6 Hz, 1 H, Ar-**H**), 5.67 (dd, J_1 = 8.3 Hz, J_2 = 10.1 Hz, 1 H, -C**H**), 4.26 (dd, J_1 = 8.8 Hz, J_2 = 10.1 Hz, 1 H, -C**H$_2$**), 3.69 (t, *J* = 8.5 Hz, 1 H, -C**H$_2$**).

^{13}C-NMR (75 MHz, $CDCl_3$, 298 K) δ / ppm = 166.8 (1 C, -N=**C**), 158.0 (d, *J* = 240.7 Hz, 1 C, **C**$_{Ar}$-F), 142.8 (1 C, **C**$_{Ar}$), 133.8 (d, *J* = 1.9 Hz, 1 C, **C**$_{Ar}$), 128.9 (2 C, **C**$_{Ar}$), 128.6 (d, *J* = 9.9 Hz, 1 C, **C**$_{Ar}$), 127.8

(1 C, **C**$_{Ar}$), 126.6 (2 C, **C**$_{Ar}$), 113.3 (d, J = 23.7 Hz, 1 C, **C**$_{Ar}$), 111.0 (d, J = 27.5 Hz, 1 C, **C**$_{Ar}$), 108.6 (d, J = 8.4 Hz, 1 C, **C**$_{Ar}$), 75.7 (1 C, -**C**H), 53.0 (1 C, -**C**H_2).

^{19}F-NMR (282 MHz, $CDCl_3$, 298 K) δ / ppm = -121.1 (m, 1 F, Ar-**F**).

MeO-ITU2 (7-MeO-BTM)

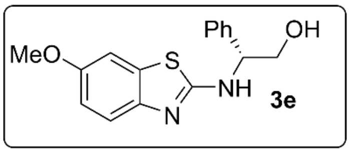

According to literature,[3] a yellow suspension of *(R)*-phenyl glycinol (3.61 g, 26.3 mmol, 1.05 eq), iPr$_2$NEt (6.50 mL, 37.6 mmol, 1.5 equiv.), 2-chloro-6-methoxybenzo[d]thiazole (5.0 g, 25.0 mmol, 1 eq) and o-dichlorobenzene (15 mL) was heated at reflux (195 °C) until completion as judged by TLC (ca. 48 h). The orange mixture was allowed to cool to r.t., H_2O (40 mL) was added and the aqueous phase was extracted with CH_2Cl_2 (3 × 50 mL). The organic layers were combined, washed with brine, dried ($MgSO_4$), filtered and concentrated in vacuo. The resulting residue was triturated with hexane to afford the crude product as an off-white solid that was recrystallized from toluene to give product **3e** as fluffy colorless crystals (5.80 g, 82%). Spectral data was in accordance with literature.[3]

^{1}H NMR (400 MHz, $CDCl_3$, 298 K) δ / ppm = 3.80 (s, 3 H, -OC**H$_3$**), 3.85 -4.06 (m, 2 H, -C**H$_2$**), 4.80 (1H, dd, J_1 = 4.0 Hz, J_2 = 6.3 Hz, 1 H, -C**H**), 5.98 (br. s, 1 H, -N**H**), 6.88 (dd, J_1 = 2.6 Hz, J_2 = 8.8 Hz, 1 H, Ar-**H**)), 7.04 (d, J = 2.6 Hz, 1 H, Ar-**H**), 7.22-7.40 (m, 5 H, Ar-**H**), 7.44 (d, J = 8.8 Hz, 1 H, Ar-**H**).

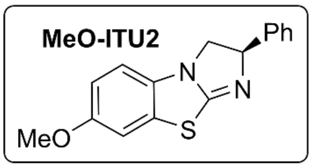

According to literature,[3] Compound **3e** (5.8g, 19.31 mmol, 1 eq) and Et_3N (10.80 mL, 77.24 mmol, 4 eq) in anhydrous CH_2Cl_2 was stirred at 0 °C. After 10 min MsCl (1.74 mL, 25.1 mmol, 1.3 eq was added. The ice/water bath was removed, and the reaction stirred for 15 mins. Once complete was observed by TLC, *i*PrOH (0.3 mL) was added and the reaction was heated at reflux for 16 h. The reaction was quenched with 1 M NaOH (50 mL) and the biphasic mixture stirred vigorously for 30 mins. The aqueous layer was extracted with CH_2Cl_2 and the combined organic phases washed with brine, dried (MgSO4), filtered and concentrated in vacuo to afford the crude product which was purified by column chromatography on silica (using a heptanes/EtOAc gradient, with the catalyst eluting using an 1/1 mixture or pure EtOAc) to give **MeO-ITU2** as a colourless crystalline solid (2.90 g, 53%). Spectral data was in accordance with literature.[3]

^{1}H NMR (500 MHz, $CDCl_3$, 298 K) δ / ppm = 3.68 (app. t, J = 8.6 Hz, -C**H$_2$**), 3.78 (s, 3 H, -OC**H$_3$**), 4.26 (dd, J_1 = 8.6 Hz, J_2 = 10.1 Hz, 1 H, -C**H$_2$**), 5.65 (dd, J_1 = 8.6 Hz, J_2 = 10.1 Hz, 1 H, -C**H**), 6.60 (d, J = 8.5 Hz, Ar-**H**), 6.74 (dd, J_1 = 2.5 Hz, J_2 = 8.6 Hz, 1 H, Ar-**H**), 6.93 (d, J = 2.6 Hz, 1 H, Ar-**H**), 7.27-7.32 (m, 1 H, Ar-**H**), 7.33-7.41 (m, 4 H, Ar-**H**).

4. Synthesis of Benzannulated Isoselenoureas

4.1 General Procedure C for the Synthesis of Formamides

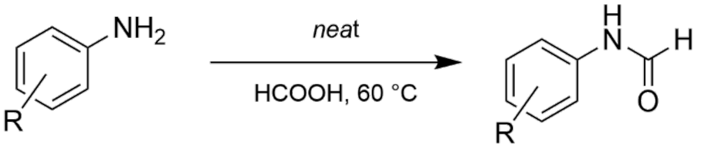

With slight adaptions to a known procedure[4], the respective aniline derivative (1 eq) was suspended in formic acid (10 eq) and heated to 60 °C for 24 h. After cooling to r.t., the mixture was diluted with EtOAc and then washed with water and brine. The organic layer was dried over Na_2SO_4 and concentrated on the rotary evaporator. The products were purified by column chromatography (silica, heptanes/EtOAc gradient 5/2, 2/1, 1/1).

Note: For successful isoselenocyanate formation in the next step, it was important that no residual formic acid was contained in the product.

4.2 General Procedure D for the Synthesis of Isoselenocyanates

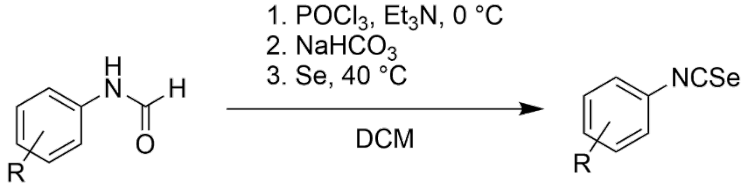

With slight adaptions to a known procedure[5], the respective formamide (1 eq) was dissolved in dry DCM (0.4 mol L^{-1}) in a flame dried three necked round bottom flask. It was cooled to 0°C and Et_3N (6 eq) and $POCl_3$ (0.8 eq) were added dropwise simultaneously. The mixture was left to stir at r.t. for 24 h. Then, a saturated $NaHCO_3$ solution (same amount as DCM) was added over a period of 30 min via dropping funnel. The mixture was stirred for further 2 h. The layers were separated, and the aqueous phase was washed with DCM three times. The combined organic phases were dried with $NaSO_4$ and filtered. The DCM was distilled off in a well-vented fume hood. The obtained isonitriles were used in the next step without further purification. The respective isonitrile was taken up in anhydrous DCM (0.4 mol L^{-1}) in a flame dried three necked round bottom flask and the mixture was heated to 40 °C. Black metallic Selenium powder (3 eq) was added to the refluxing mixture. It is crucial to note, that at this stage, lights in the fume hood were turned off and the reaction flask was covered with aluminum foil to protect it further from light. The mixture was stirred at 40 °C for 20 h. The mixture was left to cool to r.t. and then filtered over a bed of Celite. The solids were washed with additional DCM until the filtrated was colorless. The filtrate was concentrated on the rotary evaporator to give the corresponding

isoselenocyanates. The products were purified by column chromatography (Silica, heptanes) and stored in a freezer.

Notes:

(i) Isonitriles are quite odorous compounds. Working in a well-vented fume hood with the cover as much down as possible is strongly recommended and the solvent should not be removed on the rotary evaporator due to the high volatility of the isonitriles. The isonitriles should not be stored for too long and rater be transformed into the isoselenocyanates right away. Too long exposure led to major headaches.

(ii) The formation of the isoselenocyanates can be easily monitored with TLC: After short exposure to air, PhNCSe spots will turn red on TLC plates. When monitoring the reaction with MS; it is crucial to note, that any MeOH present in the MS solvent, will react with the isoselenocyanates and only the respective carbamates will be detectable.

(iii) Carrying out column chromatography in the end was crucial for isoselenocyanate quality, even though there are no apparent changes in the NMR spectra. It is strongly recommended to do the column rather fast in order to prevent degradation. The same is true for the formamide starting materials. Those also need to be perfectly pure (and should not contain any residual amounts of formic acid).

4.3 General Procedure E for the Synthesis of Isoselenourea Catalysts

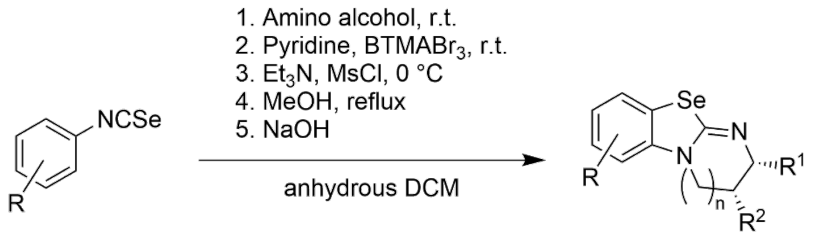

According to literature[1], the respective amino alcohol (1 eq) and isoselenocyanate (1 eq) were dissolved in anhydrous DCM (0.4 mol L^{-1}) in a flame dried flask. The mixture was protected from light and stirred at r.t. for 1 h. Then, pyridine (1 eq) was added. A solution of $BTMABr_3$ (1 eq) in anhydrous DCM (0.4 mol L^{-1}) was added in on portion and the mixture was warmed to r.t. overnight. Then, it was cooled to 0 °C and Et_3N (6 eq) was added. MsCl (3 eq) in anhydrous DCM (1.8 mol L^{-1}) was added dropwise. After complete addition, the mixture was further stirred at r.t. for 2.5 h. Then, MeOH (10 eq) was added, and it was heated to reflux for 16 h. After cooling to r.t., 1 M NaOH (same amount as DCM) was added, and it was further stirred for 0.5 h. Then, the phases were separated, and the aqueous phase was extracted with DCM three times. The combined organic layers were washed with brine, dried over Na_2SO_4, filtered and concentrated. The crude products were purified by column chromatography (silica, heptanes/EtOAc 10/1 to 3/1) and then triturated with either Et_2O or *n*-pentane.

Notes (all Se catalysts):

(i) The use of an older batch of the phenyl isoselenocyanate led to inferior results. The quality of the isoselenocyanates can roughly be assessed by color. Freshy prepared batches are almost colorless to yellow, while after time the color changes from yellow over orange to red. The darker the color, the poorer the reaction outcome was concerning yield and purity.

(ii) Light protection seems to be an important factor as it is known from selenium chemistry in general.

(iii) After the addition of $BTMABr_3$ and stirring overnight, the formation of a black, powdery precipitate was observed. The precipitate was insoluble in both DCM and water and had a metallic appearance. It appears to be elemental selenium. In the synthesis of Se-*Hyper*BTM, which was done analogously, no such precipitation was observed. This precipitate can be easily removed by a simple filtration over a cotton plug before the extraction step and is a likely reason for the relatively low yield for the selenium catalyst.

4.4 Product Characterization

***N*-Phenylformamide**

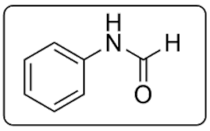

Following general procedure C, *N*-phenyl formamide was obtained as a mixture of both tautomers (roughly 1:1) in a yield of 90% as an off-white solid. Spectral data was in accordance with literature.[6]

^{1}H-NMR (300 MHz, $CDCl_3$, 298 K) *δ* / ppm = 9.24 (d = 9.4 Hz, 1 H), 8.70 (d, *J* = 11.4 Hz, 1 H), 8.53 (br. s, 1 H), 8.31 (d, *J* = 1.9 Hz, 1 H), 7.56 (d, *J* = 7.8 Hz, 2 H), 7.35-7.08 (m, 8 H)

Phenyl isoselenocyanate

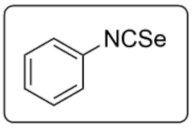

Following general procedure D, phenyl isoselenocyanate was obtained as a yellow oil in a yield of 70% (40 mmol scale). Spectral data was in accordance with literature.[1]

^{1}H-NMR (300 MHz, $CDCl_3$, 298 K) *δ* / ppm = 7.43-7.35 (m, 3 H, Ar-**H**), 7.32-7.29 (m, 2 H, Ar-**H**).

Se*Hyper*BTM (ISeU5)

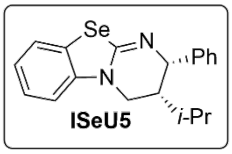

Catalyst **ISeU5** was prepared according to general procedure E and obtained as an off-white powder in a yield of 60% (5.7 mmol scale). Spectral data was in accordance with literature.[1]

^{1}H-NMR (300 MHz, $CDCl_3$, 298 K) δ / ppm = 7.42 (dd, J_1 = 1.0 Hz, J_2 = 7.6 Hz, 1 H, Ar-**H**), 7.34-7.21 (m, 6 H, Ar-**H**), 7.02 (td, J_1 = 0.9 Hz, J_2 = 7.6 Hz, 1 H, Ar-**H**), 6.81 (d, J = 8.0 Hz, 1 H, Ar-**H**), 4.94 (dd, J_1 = 1.5 Hz, J_2 = 4.3 Hz, 1 H, -C**H**), 3.86 (ddd, J_1 = 1.5 Hz, J_2 = 5.2 Hz, J_3 = 11.6 Hz, 1 H, -C**H$_2$**), 3.34 (App. t, J = 11.6 Hz, 1 H, -C**H$_2$**), 2.05-1.95 (m, 1 H, -C**H**), 1.37-1.26 (m, 1 H, -C**H**), 1.14 (d, J = 6.6 Hz, 3 H, -C**H$_3$**), 0.85 (d, J = 6.6 Hz, 3 H, -C**H$_3$**).

^{77}Se-NMR (95 MHz, CD_2Cl_2, 298 K) δ / ppm = 400.8 (s, 1 Se).

SeHBTM (ISeU4)

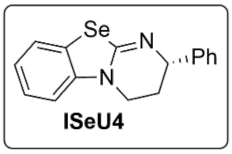

Catalyst **ISeU4** was prepared according to general procedure E and obtained as a brownish solid in a yield of 42% (1.5 mmol scale).

Melting point: 141.0-143.5 °C.

^{1}H-NMR (300 MHz, $CDCl_3$, 298 K): δ / ppm = 7.42-7.34 (m, 6 H, Ar-**H**), 7.23 (t, J = 7.7 Hz, 1 H, Ar-**H**), 6.99 (t, J = 7.7 Hz, 1 H, Ar-**H**), 6.73 (d, J = 7.7 Hz, 1 H, Ar-**H**), 4.72 (dd, J_1 = 4.1 Hz, J_2 = 8.0 Hz, 1 H, -C**H**), 3.85-3.76 (m, 1 H, -C**H$_2$**), 3.71-3.63 (m, 1 H, -C**H$_2$**), 2.39-2.29 (m, 1 H, -C**H$_2$**), 2.09-1.97 (m, 1 H, -C**H$_2$**).

^{13}C-NMR (75 MHz, $CDCl_3$, 298 K): δ / ppm = 156.3 (1 C, -N=**C**), 144.2 (1 C, **C$_{Ar}$**), 142.4 (1 C, **C$_{Ar}$**), 128.6 (2 C, **C$_{Ar}$**), 127.0 (1 C, **C$_{Ar}$**), 126.7 (2 C, **C$_{Ar}$**), 126.5 (1 C, **C$_{Ar}$**), 125.5 (1 C, **C$_{Ar}$**), 122.2 (1 C, **C$_{Ar}$**), 121.1 (1 C, **C$_{Ar}$**), 108.8 (1 C, **C$_{Ar}$**), 59.3 (1 C, -**C**H), 41.3 (1 C, -**C**H$_2$), 28.2 (1 C, -**C**H$_2$).

HRMS (ESI-TOF): *m/z:* $[M+H]^+$ calcd for $C_{16}H_{15}N_2Se^+$ 315.0359 found 315.0397.

SeBTM (ISeU2)

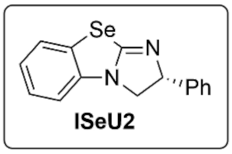

Catalyst **ISeU2** was prepared according to general procedure E and obtained as a slightly yellow solid in a yield of 43% (5.7 mmol scale).

Melting point: 103.5-105.9 °C

^{1}H-NMR (300 MHz, $CDCl_3$, 298 K): δ / ppm = 7.39-7.37 (m, 5 H, Ar-**H**), 7.31-7.28 (m, 1 H, Ar-**H**), 7.20 (t, J = 7.7 Hz, 1 H, Ar-**H**), (t, J = 7.7 Hz, 1 H, Ar-**H**), 6.65 (d, J = 7.9 Hz, 1 H, Ar-**H**), 5.63 (dd, J_1 = 7.9 Hz, J_2 = 10.4 Hz, 1 H, -C**H**), 4.25 (dd, J_1 = 8.9 Hz, J_2 = 10.4 Hz, 1 H, -C**H$_2$**), 3.70 (dd, J_1 = 7.9 Hz, J_2 = 8.9 Hz, 1 H, -C**H$_2$**).

^{13}**C-NMR** (75 MHz, $CDCl_3$, 298 K): *δ* / ppm = 163.7 (1 C, -N=**C**), 143.2 (1 C, $\mathbf{C}_{Ar}$), 138.2 (1 C, $\mathbf{C}_{Ar}$), 128.9 (2 C, $\mathbf{C}_{Ar}$), 127.7 (1 C, $\mathbf{C}_{Ar}$), 127.2 (1 C, $\mathbf{C}_{Ar}$), 126.7 (2 C, $\mathbf{C}_{Ar}$), 126.5 (1 C, $\mathbf{C}_{Ar}$), 125.1 (1 C, $\mathbf{C}_{Ar}$), 121.8 (1 C, $\mathbf{C}_{Ar}$), 109.6 (1 C, $\mathbf{C}_{Ar}$),75.1 (1 C, -**C**H), 52.5 (1 C, -**C**H_2).

^{77}**Se-NMR** (95 MHz, CD_2Cl_2, 298 K) *δ* / ppm = 335.9 (s, 1 Se).

HRMS (ESI-TOF): *m/z:* $[M+H]^+$ calcd for $C_{15}H_{13}N_2Se^+$ 301.0238 found 301.0248.

SeDHPB (ISeU3)

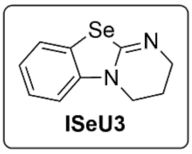

Catalyst **ISeU3** was prepared according to general procedure E and obtained as a brownish solid in a yield of 42% (1.9 mmol scale).

Melting point: 79.0-81.2 °C

^{1}**H-NMR** (300 MHz, $CDCl_3$, 298 K): *δ* / ppm = 7.34 (dd, J_1 = 1.2 Hz, J_2 = 7.7 Hz, 1 H, Ar-**H**), 7.21 (td, J_1 = 1.2 Hz, J_2 = 7.7 Hz, 1 H, Ar-**H**), 6.96 (td, J_1 = 1.1 Hz, J_2 = 7.6 Hz, 1 H, Ar-**H**), 6.72 (dd, J_1 = 0.8 Hz, J_2 = 8.1 Hz, 1 H, Ar-**H**), 3.75 (t, *J* = 6.1 Hz, 2 H, -C$\mathbf{H_2}$), 3.56 (t, *J* = 5.6 Hz, 2 H, -C$\mathbf{H_2}$), 2.05 (quint., *J* = 5.9 Hz, 1 H, -C$\mathbf{H_2}$).

^{13}**C-NMR** (75 MHz, $CDCl_3$, 298 K): *δ* / ppm = 156.2 (1 C, -N=**C**), 142.5 (1 C, $\mathbf{C}_{Ar}$), 126.5 (1 C, $\mathbf{C}_{Ar}$), 125.5 (1 C, $\mathbf{C}_{Ar}$), 122.1 (1 C, $\mathbf{C}_{Ar}$), 121.0 (1 C, $\mathbf{C}_{Ar}$), 108.6 (1 C, $\mathbf{C}_{Ar}$), 46.2 (1 C, -**C**H_2), 42.8 (1 C, -**C**H_2), 20.1 (1 C, -**C**H_2).

^{77}**Se-NMR** (95 MHz, $CDCl_3$, 298 K) *δ* / ppm = 413.6 (s, 1 Se).

HRMS (ESI-TOF): *m/z:* $[M+H]^+$ calcd for $C_{16}H_{15}N_2Se^+$ 239.0082 found 239.0082.

5. Synthesis of SeTM

(±)-SeTM was synthesized according to a previously reported 5-step procedure.[7,8]

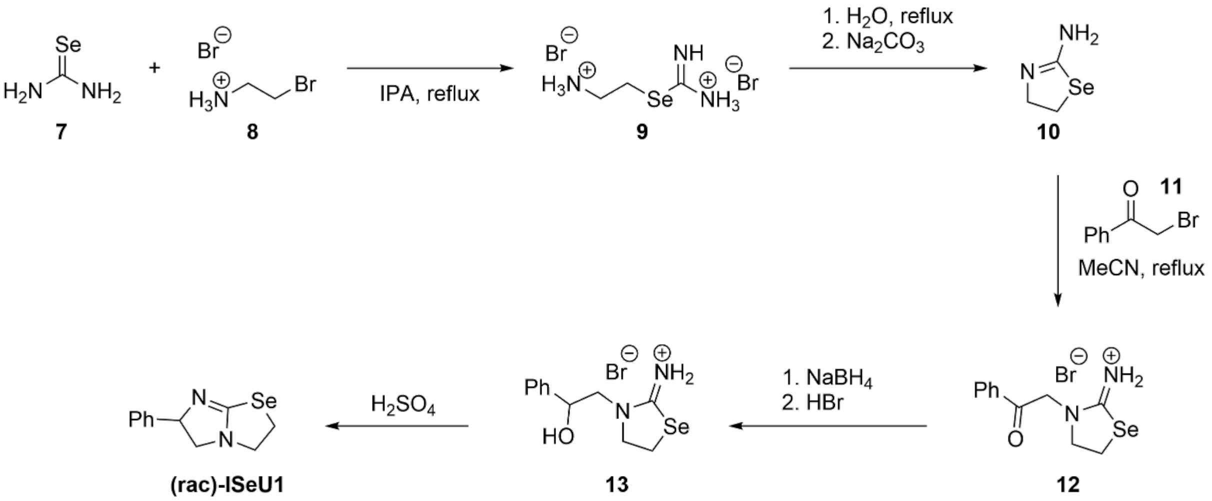

2-(2-Ammonioethyl)isoselenouronium bromide (9)

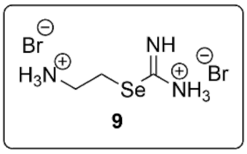

According to literature[7], selenourea (16.3 mmol, 3..33 g, 1 eq) and 2-bromoethylamin hydrobromide (16.3 mmol, 2.00 g, 1 eq) were dissolved in IPA (100 mL, 0.16 mol L^{-1}) and heated to reflux for 60 min. After cooling to r.t., the emerging gray solid was filtered off and washed with cold IPA. Compound **9** was obtained in a yield of 4.31 g (81%).

No useful spectrum could be obtained due to insolubility in most common NMR solvents and overlap between product and solvent/water signals in DMSO-d6 and D_2O. However, the melting point matched the literature value. The recrystallization done in literature (from EtOAc/EtOH 1/10) was not performed due to high insolubility of the product and the fact that it was of sufficient purity anyways.

Melting Point: 197-201 °C, (Lit[7]: 200-204 °C).

4,5-Dihydro-1,3-selenazol-2-amine (10)

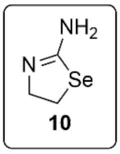

With slight adaptions to literature[7], compound **9** from the first step (13.1 mmol, 4.31 g) was dissolved in water (200 mL, 0.07 mol L^{-1}) and heated to reflux for 45 min. The water was removed under reduced pressure and the residue was taken up in a saturated, aqueous Na_2CO_3 solution. This was extracted with DCM 6 times. The combined organic layers were dried over Na_2SO_4, filtered and concentrated. Selenazol **10** was obtained as a white crystalline solid in a yield of 1.22 g (62%). The product was used without further purification.

^{1}H-NMR (300 MHz, DMSO-d6, 298 K): *δ* / ppm = 6.37 (br. s, 2 H, -N**H₂**), 3.77 (t, *J* = 7.1 Hz, 2 H, -C**H₂**), 3.36 (t, *J* = 7.1 Hz, 2 H, -C**H₂**).

^{13}C-NMR (75 MHz, $CDCl_3$, 298 K): *δ* / ppm = 154.9 (1 C, -N=**C**), 62.3 (1 C, -**C**H_2), 33.2 (1 C, -**C**H_2).

Melting Point: 162-165 °C, (Lit[7] 170-171 °C).

3-(2-Oxo-2-phenylethyl)-1,3-selenazolidin-2-iminium bromide (12)

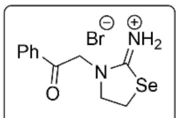

12

According to literature[8], selenazol **10** (8.2 mmol, 1.22 g, 1 eq) was dissolved in MeCN (20 mL, 0.4 mol L^{-1}). The solution was stirred until everything had dissolved. Then, ω-bromo acetophenone (8.5 mmol, 1.04 eq) was added portionwise over a period of 10 min. After complete addition, the mixture was heated to reflux for 60 min. The mixture was cooled to r.t. and then to 0 °C to ensure complete crystallization. The crystals were filtered off, washed with little cold MeCN and then dried under vacuo to obtain compound **12** as a white solid in a yield of 2.85 g (quantitative).

^{1}H-NMR (300 MHz, DMSO-d6, 298 K): *δ* / ppm = 9.73 (br. s, 1 H, -N**H**), 9.34 (br. s, 1 H, -N**H**), 8.00-7.98 (m, 2 H, Ar-**H**), 7.76-7.15 (m, 1 H, Ar-**H**), 7.64-7.59 (m, 2 H, Ar-**H**), 5.34 (s, 2 H, -C**H$_2$**), 4.08 (t, *J* = 7.3 Hz, 2 H, -C**H$_2$**), 3.54 (t, *J* = 7.3 Hz, 2 H, -C**H$_2$**).

^{13}C-NMR (75 MHz, $CDCl_3$, 298 K): *δ* / ppm = 191.0 (1 C, -**C**=O), 170.7 (1 C, -N=**C**), 134.1 (1 C, **C$_{Ar}$**), 128.8 (2 C, **C$_{Ar}$**), 128.2 (2 C, **C$_{Ar}$**), 118.1 (1 C, **C$_{Ar}$**), 57.7 (1 C, -**C**H_2), 54.4 (1 C, -**C**H_2), 23.5 (1 C, -**C**H_2).

Melting Point: 176-177 °C (evaporation), (Lit[8] 195-196 °C).

3-(2-Hydroxy-2-phenylethyl)-1,3-selenazolidin-2-iminium bromide (13)

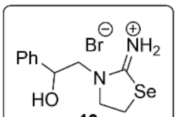

13

According to literature[8], compound **12** from the previous step (2.85 g, 8.2 mmol, 1 eq) was suspended in MeOH (50 mL, 0.16 mol L^{-1}). $NaBH_4$ was added portionwise over a period of 15 min. After compete addition, the mixture was further stirred at r.t. for 60 min. The solvent was removed under reduced pressure and the residual solid was taken up in 1 M HBr aq. (50 mL, tempered to 80 °C) and stirred for 15 min. It was filtered over a pre-heated glass sinter funnel and the filtrate was concentrated until a precipitate started to form. This precipitate was collected via vacuum filtration, washed with little amounts of water, and dried under vacuo. Compound **13** was obtained as a white solid in a yield of 2.03 g (71%).

^{1}H-NMR (300 MHz, DMSO-d6, 298 K): *δ* / ppm = 9.69 (br.vs, 1 H, -N**H**),9.14 (br. s, 1 H, -N**H**), 7.51-7.48 (m, 2 H, Ar-**H**), 7.40-7.27 (m, 3 H, Ar-**H**), 4.93 (dd, J_1 = 3.7 Hz, J_2 = 9.0 Hz, 1 H, -C**H**), 4.16 (br., 1 H, -O**H**), 4.13 (t, *J* = 7.3 Hz, 2 H, -C**H$_2$**), 3.74 (dd, J_1 = 9.0 Hz, J_2 = 14.3 Hz, 1 H, -C**H$_2$**), 3.61 (dd, J1 = 3.7 Hz, J2 = 14.3 Hz, 1 H, -CH2), 3.43 (t, J = 7.3 Hz, 2 H, -CH2).

^{13}C-NMR (75 MHz, $CDCl_3$, 298 K): *δ* / ppm = 169.1 (1 C, -N=**C**), 141.9 (1 C, **C$_{Ar}$**), 128.1 (2 C, **C$_{Ar}$**), 127.6 (1 C, **C$_{Ar}$**), 126.3 (2 C, **C$_{Ar}$**), 69.1 (1 C, -**C**OH), 57.2 (1 C, -**C**H_2), 54.2 (1 C, -**C**H_2), 23.4 (1 C, -**C**H_2).

SeTM (ISeU1)

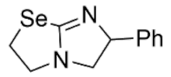

ISeU1

Adapting a literature procedure[8], 5.5 mL of concentrated sulfuric acid were cooled to 0 °C. Product **13** from the last step (5.4 mmol, 1.90 g) was cautiously added in small portions over a period of 30 min. The mixture was further stirred at 0 °C for 5 min. Then, little amounts of ice were added cautiously in order to dilute and cool the thick mixture. Then, the mixture was poured into an ice/water mixture and adjusted to pH = 12 by the addition of a 25% aq. ammonia solution. It was extracted with DCM 6 times and the combined organic layers were washed with brine, dried over Na_2SO_5, filtered and concentrated. SeTM (**ISeU1**) was obtained as an off-white solid after trituration with Et_2O in a yield of 1.11 g (82%).

Melting point: 79.5-82.3 °C

^{1}H-NMR (500 MHz, CD_2Cl_2, 298 K): *δ* / ppm = 7.39-7.35 (m, 4 H, Ar-**H**), 7.32-7.27 (m, 1 H, Ar-**H**), 5.41 (t, *J* = 9.0 Hz, 1 H, -C**H**), 3.72-3.67 (m, 2 H, -C**H₂**), 3.58 (ddd, J_1 = 4.8 Hz, J_2 = 6.1 Hz, J_3 = 10.0 Hz, 1 H, -C$\mathbf{H_2}$), 3.40 (ddd, J_1 = 4.8 Hz, J_2 = 6.1 Hz, J_3 = 9.3 Hz, 1 H, -C$\mathbf{H_2}$), 3.15 (td, J_1 = 6.1 Hz, J_2 = 8.8 Hz, 1 H, -C$\mathbf{H_2}$), 2.98 (t, *J* = 8.8 Hz, 1 H, -C$\mathbf{H_2}$).

^{13}C-NMR (125 MHz, CD_2Cl_2 , 298 K): *δ* / ppm = 169.9 (1 C, -N=**C**), 143.3 (1 C, $\mathbf{C_{Ar}}$), 128.3 (2 C, $\mathbf{C_{Ar}}$), 127.1 (1 C, $\mathbf{C_{Ar}}$), 126.5 (2 C, $\mathbf{C_{Ar}}$), 76.9 (1 C, -**C**H), 58.3 (1 C, -**C**H_2), 49.7 (1 C, -**C**H_2), 27.9 (1 C, -**C**H_2).

^{77}Se-NMR (95 MHz, CD_2Cl_2, 298 K) *δ* / ppm = 205.9 (s, 1 Se).

HRMS (ESI-TOF): *m/z:* $[M+H]^+$ calcd for $C_{11}H_{13}N_2Se^+$ 253.0238 found 253.0239.

6. Synthesis of Isotelluroureas

6.1 Synthesis of the Ditelluride Precursor (14)

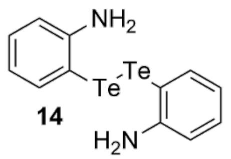

14

According to a procedure developed by D. Bonifazi[9], NaH (60% in mineral oil, 94.3 mmol, 3.77 g, 3 eq) and tellurium powder (~30 mesh, 31.3 mmol, 4.00 g, 1 eq) were suspended in freshly distilled dry NMP[a)] (50 mL, 0.6 mol L^{-1}) in a flame dried two necked round bottom flask. The flask was emerged into an oil bath pre-heated to 180 °C and kept at this temperature for 0.5 h. After this time, a dark purple solution was obtained. 2-bromoaniline (31.3 mmol, 3.55 mL, 1 eq) was added in one portion and the mixture was further stirred at 180 °C for 4.5 h. After cooling to r.t., the mixture was diluted with 200 mL water and buffered by the addition of NaH_4Cl (94.3 mmol, 5.04 g, 3 eq). The mixture was then extracted with 200 mL portions of Et_2O until no color change in the organic extracts was visible anymore (10 extraction steps). As there was a lot of insoluble solid was present during the extraction, multiple filtration steps over cotton plugs were performed during the extraction. The combined organic layers were washed with brine, dried over Na_2SO_4 and concentrated. The crude product was

recrystallized from a 1/1 mixture of *n*-pentane/toluene. Ditelluride **14** was obtained as a dark red glittery solid in a yield of 2.92 g (42%).

a) 200 mL NMP were stirred with 1 g CaH_2 over the weekend, filtered and then vacuum distilled (Oil bath: 170 °C, Head: 130 °C, 100mbar).

Spectral data was in accordance with literature.[9]

^{1}H-NMR (300 MHz, $CDCl_3$, 298 K): δ / ppm = 7.69 (dd, J_1 = 1.5 Hz, J_2 = 7.6 Hz, 2 H, Ar-**H**), 7.12 (ddd, J_1 = 1.5 Hz, J_2 = 7.4 Hz, J_3 = 8.0 Hz, 2 H, Ar-**H**), 6.72 (dd, J_1 = 1.2 Hz, J_2 = 8.0 Hz, 2 H, Ar-**H**), 6.51 (dd, J_1 = 1.2 Hz, J_2 = 7.4 Hz, 1 H, Ar-**H**), 4.17 (br. s, 4 H, -N**H_2**).

Isotelluroureas are accessible via the following 5-step procedure:

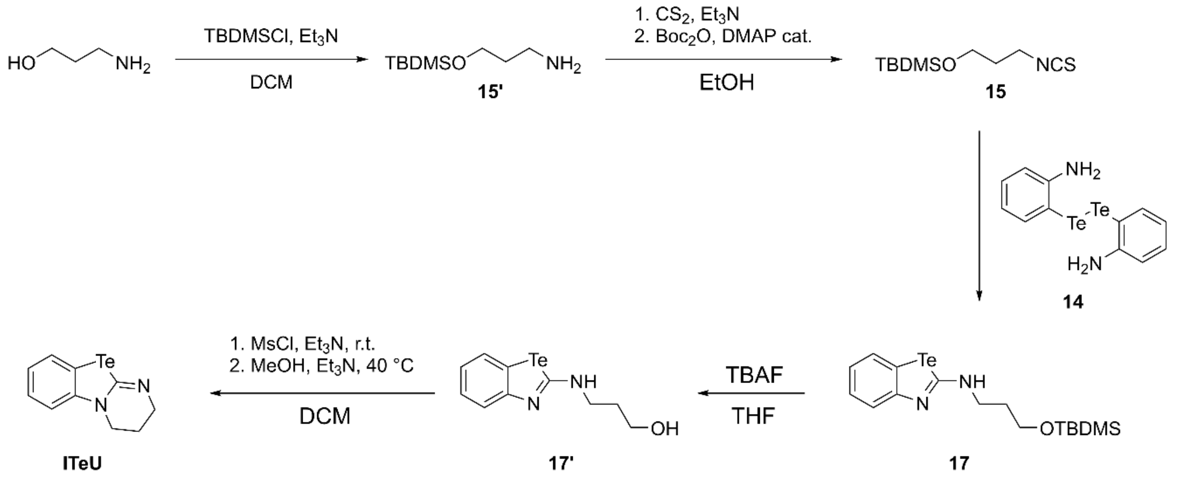

6.2 General Procedure F for the Synthesis of Protected Amino Alcohols

According to literature[10], TBDMSCl (1.1 eq) was dissolved in DCM (0.6 mol L^{-1}). Et_3N (1.5 eq) and the respective amino alcohol (1.0 eq) were added, and the mixture was stirred at r.t. overnight. The reaction was quenched by the addition of water and the phases were separated. The aqueous layer was extracted with DCM three times. The combined organic layers were dried over Na2SO4, filtered and concentrated. The obtained TBDMS protected amino alcohols were used in the next step without any further purification unless otherwise specified.

6.3 General Procedure G for the Synthesis of Isothiocyanates

In analogy to a known procedure[11], the respective TBDMS protected amino alcohol (1 eq) was dissolved in EtOH (1.0 mol L^{-1}). Then, CS_2 (10 eq) and Et_3N (1 eq) were added, and the mixture was

stirred at r.t. for 30 min. Subsequently, Boc_2O (1eq) and DMAP (2.5mol%) in EtOH each were added. (1/10 of the amount of solvent used initially). The mixture was further stirred until gas evolution has ceased. Excess CS_2 was removed by pulling off the headspace of the reaction through a NaOH washing flask using water jet vacuum for at least 1 h. Remaining EtOH was evaporated, and the crude product was purified by column chromatography (silica, pure DCM as the eluent).

6.4 General Procedure H for the Synthesis of 2-Aminobenzotellurazoles

In a so far unprecedented procedure, ditelluride **14** (1.0 mmol, 0.42 g, 1 eq) and the respective isothiocyanate (2 eq) were suspended in MeOH (20 mL, 0.05 mol L^{-1}) in a pressure resistant Schlenk flask under air. The flask was closed, and the mixtures were heated to 80 °C for 14 h. After cooling to r.t., the flask was opened, and the contents were transferred to a round bottom flask with DCM (washing the flask a few times might be necessary) and then concentrated. The crude product was purified by column chromatography (silica, heptanes/EtOAc 20/1, 10/1, 5/1, 2/1). *Note: The concentration is crucial! For bigger scales, it is recommended to split between more flasks and not increase the reaction scale. Given yields refer to a mechanism, where only 1 benzotellurazole molecule can form from the ditelluride. Considering the fact, that 1 mmol ditelluride contains 2 mmol tellurium, also the formation of 2 mmol product would be possible. Considering this scenario, the given yields must be divided by 2.*

6.5 General Procedures I for the Deprotection Reaction

Procedure I: Using TBAF

The respective benzotellurazole from the previous step (1 eq) was dissolved in anhydrous THF (0.075 mol L^{-1}) and cooled to 0 °C. TBAF (1.0 M in THF, 1.1 eq) was added dropwise via syringe. After complete addition, the mixture was further stirred at 0 °C for 30 min. The reaction was quenched by the addition of a saturated aq. NH_4Cl solution. The organic phase was washed another two times with the sat. NH_4Cl solution, dried over Na_2SO_4, filtered and concentrated. The crude product was purified by column chromatography (silica, DCM/MeOH 100/1, 50/1, 20/1).

Procedure I2: Using HCOOH

The respective benzotellurazole from the previous step (1 eq) was dissolved in a 6/1 THF/H2O mixture (0.15 mol L^{-1}). Then, HCOOH (1.0 mL per 1.0 mmol of benzotellurazole) was added and the mixture was stirred at r.t for at least 24 h. After complete deprotection (monitored via MS spectrometry), the reaction was quenched by the careful addition of a sat. $NaHCO_3$ solution (until gas evolution ceases, or pH = 8). The phases were separated, and the organic phase was washed another time with a sat. $NaHCO_3$ solution. The combined aqueous phases were extracted with Et_2O twice and all combined organic layers were washed with brine, dried over Na_2SO_4, filtered and concentrated. The crude products were purified by column chromatography (silica, DCM/MeOH 100/1, 50/1, 20/1).

6.6 General Procedure J for the Synthesis of Isotelluroureas

The respective deprotected benzotellurazole (1 eq) was dissolved in anhydrous DCM (0.08 mol L^{-1}). It was cooled to 0 °C and Et_3N (3 eq) and MsCl (1.5 eq) were added dropwise simultaneously. The mixture was warmed to r.t. over 60 min. Then, another portion Et_3N (12 eq) and MeOH (5.3 eq) were added and the mixture was heated to reflux for 16 h. After cooling to r.t., the reaction was quenched by the addition of 1 M NaOH. After 15 min of stirring, the phases were separated, and the aqueous layer was extracted with DCM three times. The combined organic layers were washed with brine, dried over Na2SO4, filtered and concentrated. The crude product was triturated with small amounts of the indicated solvent system. Supernatant solvent was carefully decanted and the solid was then washed with *n*-pentane three times.

6.7 Characterisation Data

<u>Note:</u> *We have observed that chemical shifts of both protected and deprotected benzotellurazoles in ^{1}H-NMR spectra can change when repeating the experiments. It is therefore recommended to always check mass spectra as well.*

TBDMS protected Amino Alcohol 15a'

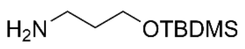

15a'

Following general procedure F, TBDMS protected amino alcohol **15a** was obtained as a clear liquid in a yield of 98% (40 mmol scale). Spectral data were in accordance with literature.[10]

^{1}H-NMR (300 MHz, $CDCl_3$, 298 K): δ / ppm = 3.68 (t, *J* = 6.1 Hz, 2 H, -C**H₂**), 2.78 (t, *J* = 6.8 Hz, 2 H, -C**H₂**), 1.64 (quint., *J* = 6.4 Hz, 2 H, -C**H₂**), 0.88 (s, 9 H, -SiC(C**H₃**)₃, 0.04 (s, 6 H, -SiC**H₃**).

TBDMS protected Amino Alcohol 15b'

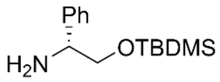

15b'

Following general procedure F, TBDMS protected amino alcohol **483d** was obtained as a clear liquid in a yield of 80% (15 mmol scale) after column chromatography on silica (heptanes/EtOAc 5/1 followed by 2/1 and pure EtOAc, TLC plates needed to be stained with ninhydrin in order to see the spots). Spectral data was in accordance with literature.[11]

1H-NMR (300 MHz, $CDCl_3$, 298 K): δ / ppm = 7.41-7.30 (m, 5 H, Ar-**H**), 4.86 (dd, J_1 = 4.8 Hz, J_2 = 7.5 Hz, 1 H, -C**H**), 3.85 (dd, J_1 = 4.8 Hz, J_2 = 10.3 Hz, 1 H, -C**H₂**), 3.79 (dd, J_1 = 7.5 Hz, J_2 = 10.3 Hz, 1 H, -C**H₂**), 0.90 (s, 9 H, -SiC(C**H₃**)₃), 0.05 (s, 3 H, -SiC**H₃**), 0.04 (s, 3 H, -SiC**H₃**).

Isothiocyanate 15a

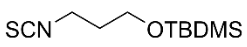

15a

Following general procedure G, the product was obtained as a colourless liquid in a yield of 82% (20 mmol scale).

^{1}H-NMR (300 MHz, $CDCl_3$, 298 K): δ / ppm = 5.66 (t, J = 5.7 Hz, 2 H, -C**H2**), 3.64 (t, J = 6.4 Hz, 2 H, -C**H2**), 1.86 (quint., J = 6.0 Hz, 2 H, -C**H2**), 0.89 (s, 9 H, -SiC(C**H3**)3), 0.07 (s, 6 H, -SiC**H3**).

^{13}C-NMR (75 MHz, $CDCl_3$, 298 K): δ / ppm = 129.9 (1 C, -N**C**S, 59.1 (1 C, -C**H2**), 41.9 (1 C, -**C**H2), 32.9 (1 C, -**C**H2), 26.0 (3 C, -SiC(**C**H3)3), 18.4 (1 C, -Si**C**(CH3)3), -5.3 (2 C, -Si**C**H3).

HRMS (ESI-TOF): *m/z:* $[M+H]^+$ calcd for $C_{10}H_{22}OSSi^+$ 232.1186 found 232.1175.

Isothiocyanate 15b

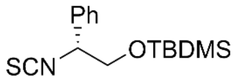

15b

Following general procedure G, the product was obtained as a slightly yellow liquid in a yield of 80% (15 mmol scale). Spectral data was in accordance with literature.[13]

^{1}H-NMR (300 MHz, $CDCl_3$, 298 K): δ / ppm = 7.40-7.30 (m, 5 H, Ar-**H**), 4.86 (dd, J_1 = 4.9 Hz, J_2 = 7.4 Hz, 1 H, -C**H**), 3.85 (dd, J_1 = 4.9 Hz, J_2 = 10.4 Hz, 1 H, -C**H2**), 3.79 (dd, J_1 = 7.4 Hz, J_2 = 10.4 Hz, 1 H, -C**H2**), 0.90 (s, 9 H, -SiC(C**H3**)3), 0.05 (s, 3 H, -SiC**H3**), 0.06 (s, -SiC**H3**).

Benzotellurazole 17a

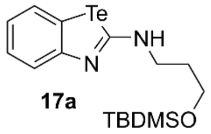

17a

Following general procedure H, benzotellurazole **17a** was obtained in quantitative yield (1.0 mmol scale) as a yellow oil. The compound has not been known to literature before.

^{1}H-NMR (300 MHz, $CDCl_3$, 298 K): δ / ppm = 7.59 (dd, J_1 = 1.1 Hz, J_2 = 7.8 Hz, 1 H, Ar-**H**), 7.56 (dd, J_1 = 1.1 Hz, J_2 = 8.0 Hz, 1 H, Ar-**H**), 7.27 (ddd, J_1 = 1.3 Hz, J_2 = 7.4 Hz, J_3 = 8.0 Hz, 1 H, Ar-**H**), 6.89 (td, J_1 = 1.3 Hz, J_2 = 7.4 Hz, 1 H, Ar-**H**), 3.77 (t, J = 5.6 Hz, 2 H, -C**H2**), 3.48 (t, J = 6.4 Hz, 2 H, -C**H2**), 1.88 (quint., J = 6.0 Hz, 2 H, -C**H2**), 0.93 (s, 9 H, -SiC(C**H3**)3), 0.08 (s, 6 H, -SiC**H3**).

^{13}C-NMR (75 MHz, $CDCl_3$, 298 K): δ / ppm = 162.9 (1 C, -N=**C**), 159.6 (1 C, **C**Ar), 131.1 (1 C, **C**Ar), 127.5 (1 C, **C**Ar), 127.2 (1 C, **C**Ar), 121.5 (1 C, **C**Ar), 121.2 (1 C, **C**Ar), 61.7 (1 C, -**C**H2), 46.3 (1 C, -**C**H2), 31.9 (1 C, -**C**H2), 26.1 (3 C, -SiC(**C**H3)3), 18.4 (1 C, -Si**C**(CH3)3), -5.2 (2 C, -Si**C**H3).

HRMS (ESI-TOF): *m/z:* $[M+H]^+$ calcd for $C_{16}H_{27}N_2OSiTe^+$ 421.0949 found 421.0948.

Benzotellurazole 17b

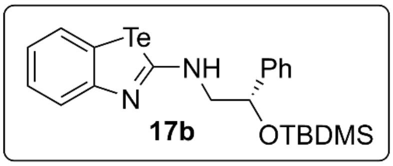

Following general procedure H, benzotellurazole **502d** was obtained in a yield of 95% (1.0 mmol scale) as a slightly yellow liquid. The compound has not been known to literature before.

^{1}H-NMR (500 MHz, $CDCl_3$, 298 K): δ / ppm = 7.62 (dd, J_1 = 1.0 Hz, J_2 = 8.1 Hz, 1 H, Ar-**H**), 7.54 (dd, J_1 = 1.2 Hz, J_2 = 7.7 Hz, 1 H, Ar-**H**), 7.43-7.41 (m, 2 H, Ar-**H**), 7.38-7.33 (m, 3 H, Ar-**H**), 7.29-7.25 (m, 1 H, Ar-**H**), 6.87 (td, J_1 = 1.2 Hz, J_2 = 7.6 Hz, 1 H, Ar-**H**), 6.58 (br., 1 H, -N**H**), 4.44 (dd, J_1 = 4.2 Hz, J_2 = 6.4 Hz, 1 H, -C**H**), 3.96 (dd, J_1 = 4. Hz, J_2 = 10.3 Hz, 1 H, -C**H₂**), 3.80 (dd, J_1 = 6.7 Hz, J_2 = 10.3 Hz, 1 H, -C**H₂**), 0.88 (s, 9 H, -SiC(C**H₃**)₃), -0.00 (s, 3 H, -SiC**H₃**), -0.01 (s, 3 H, -SiC**H₃**).

^{13}C-NMR (125 MHz, $CDCl_3$, 298 K): δ / ppm = 164.1 (1 C, -**C**=N), 158.4 (1 C, **C**$_{Ar}$), 137.8 (1 C, **C**$_{Ar}$), 130.8 (1 C, **C**$_{Ar}$), 128.9 (2 C, **C**$_{Ar}$), 128.7 (1 C, **C**$_{Ar}$), 128.5 (1 C, **C**$_{Ar}$), 128.2 (2 C, **C**$_{Ar}$), 127.1 (1 C, **C**$_{Ar}$), 121.7 (1 C, **C**$_{Ar}$), 121.5 (1 C, **C**$_{Ar}$), 67.5 (1 C, -**C**H_2), 63.8 (1 C, -**C**H_2), 26.0 (1 C, -SiC(**C**H_3)$_3$), 18.5 (1 C, -Si**C**(CH_3)$_3$), -5.4 (2 C, -Si**C**H_3).

HRMS (ESI-TOF): *m/z:* [M+H]+ calcd for $C_{21}H_{29}N_2OSiTe^+$ 483.1106 found 483.1109.

Deprotected Benzotellurazole (17a')

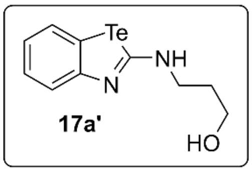

Following general procedure I1, deprotected compound **17a'** was obtained as an orange oil in a yield of 54% (2.0 mmol scale).

^{1}H-NMR (300 MHz, $CDCl_3$, 298 K): δ / ppm = 7.58-7.53 (m, 2 H, Ar-**H**), 7.27 (ddd, J_1 = 1.3 Hz, J_2 = 7.4 Hz, J_3 = 8.0 Hz, 1 H, Ar-**H**), 6.91 (td, J_1 = 1.3 Hz, J_2 = 7.4 Hz, 1 H, Ar-**H**), 3.74 (t, J = 5.6 Hz, 2 H, -C**H₂**), 3.63 (t, J = 6.0 Hz, 2 H, -C**H₂**), 1.82 (quint., J = 5.8 Hz, 2 H, -C**H₂**).

^{13}C-NMR (75 MHz, $CDCl_3$, 298 K): δ / ppm = 162.3 (1 C, -N=**C**), 158.6 (1 C, **C**$_{Ar}$), 131.2 (1 C, **C**$_{Ar}$), 127.4 (1 C, **C**$_{Ar}$), 126.8 (1 C, **C**$_{Ar}$),122.0 (1 C, **C**$_{Ar}$), 121.2 (1 C, **C**$_{Ar}$), 59.2 (1 C, -**C**H_2), 43.7 (1 C, -**C**H_2), 33.0 (1 C, -**C**H_2).

HRMS (ESI-TOF): *m/z:* $[M+H]^+$ calcd for $C_{10}H_{13}N_2OTe^+$ 307.0085 found 307.0087.

Deprotected Benzotellurazole (17b')

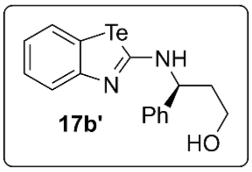

Following general procedure I2, deprotected compound **17b'** was obtained as a brownish solid in a yield of 60% (1.0 mmol scale).

^{1}H-NMR (300 MHz, $CDCl_3$, 298 K): δ / ppm = 7.59 (dd, J_1 = 1.0 Hz, *J2* = 8.1 Hz, 1 H, Ar-**H**), 7.54 (dd, J_1 = 1.2 Hz, J_2 = 7.8 Hz, 1 H, Ar-**H**), 7.37-7.28 (m, 6 H, Ar-**H**), 6.91 (td, J_1 = 1.1 Hz, J_2 = 7.5 Hz, 1 H, Ar-**H**), 4.59 (dd, J_1 = 3.8 Hz, J_2 = 7.8 Hz, 1 H, -C**H**), 3.98 (dd, J_1 = 3.8 Hz, J_2 = 11.4 Hz, 1 H, -C**H₂**), 3.89 (dd, J_1 = 7.8 Hz, J_2 = 11.4 Hz, 1 H, -C**H₂**).

^{13}C-NMR (75 MHz, $CDCl_3$, 298 K): *δ* / ppm = 163.9 (1 C, -**C**=N), 157.8 (1 C, **C$_{Ar}$**), 137.8 (1 C, **C$_{Ar}$**), 131.0 (1 C, **C$_{Ar}$**), 129.3 (2 C, **C$_{Ar}$**), 128.7 (1 C, **C$_{Ar}$**), 127.9 (1 C, **C$_{Ar}$**), 127.7 (2 C, **C$_{Ar}$**), 127.3 (1 C, **C$_{Ar}$**), 122.1 (1 C, **C$_{Ar}$**), 121.4 (1 C, **C$_{Ar}$**), 67.4 (1 C, -**C**H), 64.2 (1 C, -**C**H_2).

HRMS (ESI-TOF): *m/z:* [M+H]+ calcd for $C_{15}H_{15}N_2OTe^+$ 369.0241 found 369.0254.

TeDHPB (ITeU3)

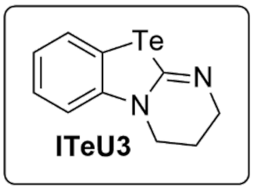

Following general procedure J, the isotellurourea catalyst was obtained as a yellow solid in a yield of 93% (2 mmol scale) after trituration from EtOAc.

Some solvent impurities could not be removed, even after prolonged drying under high vacuum.

Melting point: 162-165°C (decomposition).

^{1}H-NMR (300 MHz, $CDCl_3$, 298 K): *δ* / ppm = 7.43 (dd, J_1 = 1.3 Hz, J_2 = 7.5 Hz, 1 H, Ar-**H**), 7.24 (ddd, J_1 = 1.3 Hz, J_2 = 7.5 Hz, J_3 = 8.2 Hz, 1 H, Ar-**H**), 6.97 (td, J_1 = 1.1 Hz, J_2 = 7.5 Hz, 1 H, Ar-**H**), 6.78 (d, *J* = 8.3 Hz, 1 H, Ar-**H**), 3.77 (t, *J* = 6.1 Hz, 2 H, -C**H_2**), 3.64 (t, *J* = 5.7 Hz, 2 H, -C**H_2**), 2.13 (quint., *J* = 5.9 Hz, 1 H, -C**H_2**).

^{13}C-NMR (176 MHz, CD2Cl2, 298 K): *δ* / ppm = 149.21(1 C, -N**C**Te), 145.68 (1 C, **C$_{Ar}$**), 132.2 (1 C, **C$_{Ar}$**), 127.5 (1 C, **C$_{Ar}$**),122.8 (1 C, **C$_{Ar}$**),110.6 (1 C, **C$_{Ar}$**), 110.2 (1 C, Te**C$_{Ar}$**), 45.7 (1 C, -**C**H_2), 44.0 (1 C, -**C**H_2), 20.4 (1 C, -**C**H_2).

Recording on our 700 MHz spectrometer with D1 = 30s was crucial to make the carbon signals adjacent to the tellurium atom visible. A better visualization could be obtained in the HMBC (vide infra).

HRMS (ESI-TOF): *m/z:* [M+H]$^+$ calcd for $C_{10}H_{10}N_2Te^+$ 288.9979 found 288.9977.

TeBTM (ITeU2)

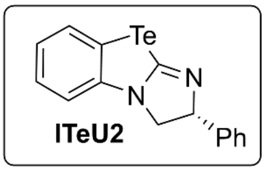

Following general procedure J, the isotellurourea catalyst was obtained as a yellowish solid in quantitative yield (2.5 mmol scale) after trituration from *n*-pentane.

^{1}H-NMR (700 MHz, $CDCl_3$, 298 K): δ / ppm = 7.44-7.42 (m, 1 H, Ar-**H**), 7.37-7.36 (m, 4 H, Ar-**H**), 7.30-7.28 (m, 1 H, Ar-**H**), 7.20 (t, *J* = 7.8 Hz, 1 H, Ar-**H**), 6.87 (t, *J* = 7.8 Hz, 1 H, Ar-**H**), 6.64 (d, *J* = 7.8 Hz, 1 H, Ar-**H**), 5.65 (dd, J_1 = 10.5 Hz, J_2 = 7.9 Hz, 1 H, -C**H**), 4.20 (dd, J_1 = 10.4 Hz, J_2 = 9.3 Hz, 1 H, -C**H_2**), 3.67 (t, *J* = 8.4 Hz, 1 H, -C**H_2**).

^{13}C-NMR (176 MHz, $CDCl_3$, 298 K): δ / ppm = 152.0 (1 C, -N=**C**), 143.4 (1 C, **C$_{Ar}$**), 140.8 (1 C, **C$_{Ar}$**), 132.8 (1 C, **C$_{Ar}$**), 128.9 (2 C, **C$_{Ar}$**), 128.1 (1 C, **C$_{Ar}$**), 127.7 (1 C, **C$_{Ar}$**), 126.7 (2 C, **C$_{Ar}$**), 121.9 (1 C, **C$_{Ar}$**), 111.1 (1 C, **C$_{Ar}$**), 110.5 (1 C, **C$_{Ar}$**), 74.2 (1 C, -**C**H), 52.7 (1 C, -**C**H_2).

HRMS (ESI-TOF): m/z: [M+H]$^+$ calcd for $C_{15}H_{13}N_2Te^+$ 351.0135 found 351.0131.

6.8 Optimization of the Cyclization Reaction between Ditelluride 14 and Isothiocyanate 15

The crucial transformation in the synthesis sequence of the tellurium catalyst is the third step, where the ditelluride reacts with the isothiocyanate. As stated in the main manuscript, it was actually not the idea to achieve the cyclization already in this step, but rather synthesize a thiourea derivative. It came to us as a big surprise, that when we performed the reaction in alcohols the cyclic products were obtained.

In the beginning of our investigations, we found that the reaction does not work in acetone, but reasonably well in EtOH (Table S1, entries 2 and 3). Better results were obtained in MeOH and worse ones in *i*-PrOH (entries 8 and 9). A major problem, however, was always the scale up of the reaction: As soon we scaled up the reaction, the yield drastically dropped, and side product formation became worse (Entries 3-6). As MeOH proofed to be the most suitable solvent, further optimization was carried out in that solvent. Switching to degassed MeOH rather worsened the reaction outcome (entry 12). Shortening the reaction time led to less formation of the unidentified side product and better yield (entry 11). While the presence of water was not too problematic, the use of $NH_4(HCOO)$, TMSCl or $BF_3.OEt_2$ as additives led to worse results compared to the reaction without any additives (entries 8, 14-17). Together with the insight that MeOH is the most suitable solvent, these observations suggest that the reaction most likely proceeds via a nucleophilic attack of the MeOH onto one tellurium atom rather than a reductive Te-Te bond cleavage.

What we then realized was, that we always carried out the small-scale reactions in far more diluted conditions compared to the big scale reactions. As literature procedures for similar reactions often use highly concentrated solutions (e.g. 0.8 mol L^{-1})[14], we opted for fairly high concentrations as well. On 0.1 mmol scale however, concentration is somewhat limited to practical amounts of solvent, usually not smaller than 1 mL (e.g. solvent needed to transfer all reactants into the reaction flask etc.). Screening the concentration then revealed, that it is in fact crucial to the reaction outcome (entries 19-21). With the optimize conditions, we could carry out the reaction smoothly on a 1.0 mmol scale as well (entry 22). However, there were still some reproducibility issues which could not be completely overcome (entries 8 and 21).

Additionally, we tested the use of only 1 eq isothiocyanate instead of the usually employed 2. This led to a far worse reaction outcome concerning both side product formation and conversion. Bigger scales than 1.0 mmol proofed to be problematic with regard to the available pressure resistant vessels and also product yield. Hence, we recommend to split up bigger scales into more smaller batches of 1.0 mmol each.

Table S1: Optimization of the reaction conditions of the first cyclisation reaction during the synthesis of TeDHPB. All reactions were performed on the given scales using 1 eq of ditelluride **14** and 2 equivalents of isothiocyanate **15.**

Entry	Scale (mmol)	solvent	Conc. (mol L^{-1})	Additive	Time (h)	T (°C)	Conv. (%)	yield
1	0.5	Acetone	0.10	-	20	r.t.	-	-
2	0.5	Acetone	0.25	-	20	80	-	-
3	0.5	EtOH	0.25	-	20	80	100	84[a)]
4	3.0	EtOH	0.30	-	20	80	77[c)]	28[a)]
5	0.1	EtOH	0.10	-	20	80	100	80[b)]
6	2.3	EtOH	0.25	-	20	80	100	38[a)]
7	4 x 0.5[d)]	EtOH	0.25	-	20	80	100	31[a)]
8	0.1	MeOH	0.10	-	20	80	100	99[b)]
9	0.1	*i*-PrOH	0.10	-	20	80	100	60[b)]
10	1.0	MeOH	0.25	-	20	80	100	74[a)]
11	1.0	MeOH	0.25	-	6	80	100	89[a)]
12	1.0	MeOH[e)]	0.25	-	6	80	60	67[a)]
13	1.0	MeOH	0.25	-	2	80	60	48[a)]
14	0.1	MeOH	0.10	H_2O	11	80	100	80[b)]
15	0.1	MeOH	0.10	$NH_4(HCOO)$	11	80	100	50[b)]
16	0.1	MeOH	0.10	TMSCl	11	80	100	-
17	0.1	MeOH	0.10	BF_3 OEt_2	11	80	100	61[b)]
18	0.1	MeOH	0.10	-	11	r.t.	-	-
19	0.1	MeOH	0.20	-	20	80	100	14[b)]
20	0.1	MeOH	0.10	-	20	80	100	80[b)]
21	0.1	MeOH	0.05	-	20	80	100	85[b)]
22	1.0	MeOH	0.05	-	20	80	100	99[a)]

[a)] Isolated yield after column chromatography
[b)] NMR yield using trimethoxy benzene as internal standard.
[c)] As judged form educt and product only, as the structure of the side product is unknown.
[d)] Reaction was performed in parallel in 4 different reaction flasks under exactly the same conditions. The crude products were then combined before chromatographic purification.
[e)] MeOH was degassed via three freeze pump thaw cycles before setting up the reaction.

7. Single Crystal X-Ray Diffraction of TeDHPB (ITeU3)

Single crystals suitable for single crystal X-ray diffraction were obtained by the vapor diffusion method of cyclohexane into EtOH. Single-crystal structure analysis was carried out at room temperature on a Bruker D8 Quest ECO diffractometer with graphite-monochromated MoKα radiation (λ = 0.71073 Å). The structures were solved by direct methods (SHELXS-2013/1[15]) and refined by full-matrix least-squares on F2 (SHELXL-2018/3[16]). The H atoms were calculated geometrically, and a riding model was applied in the refinement process. Crystallographic details for **ITeU3** can be found in Table S2. CCDC 2452429 contain the supplementary crystallographic data. This information can be obtained free of charge via https://www.ccdc.cam.ac.uk/structures.

Table S2: Crystal data for the structures of TeDHPB (**ITeU3**).

**Compound**	**ITeU3**
Empirical formula	$C_{10}H_{10}N_2Te$
Formula weight	285.80
Crystal system	tetragonal
Space group	$P\bar{4}2_1c$
Temp/K	296
a (Å)	16.928(8)
c (Å)	7.360(5)
V (Å^3)	2109(2)
Z	8
D_{calc} (g/cm^3)	1.800
μ / mm^{-1}	2.78
Reflns collected	232551
Indep. reflns	5119
Obs. reflns [I > 2σ(I)]	3154
Param. refin./restr.	129 / 2
Absorption correction	multi-scan
Final R_1 [$I > 2\ \sigma(I)$]	0.0392
Final $wR_2(F^2)$ [$I > 2\ \sigma(I)$]	0.0989
Final R_1 (all data)	0.0762
Final $wR_2(F^2)$ (all data)	0.1132
$\Delta\rho_{(max/min)}$ / e Å^{-3}	0.84 / -1.48
CCDC	2452429

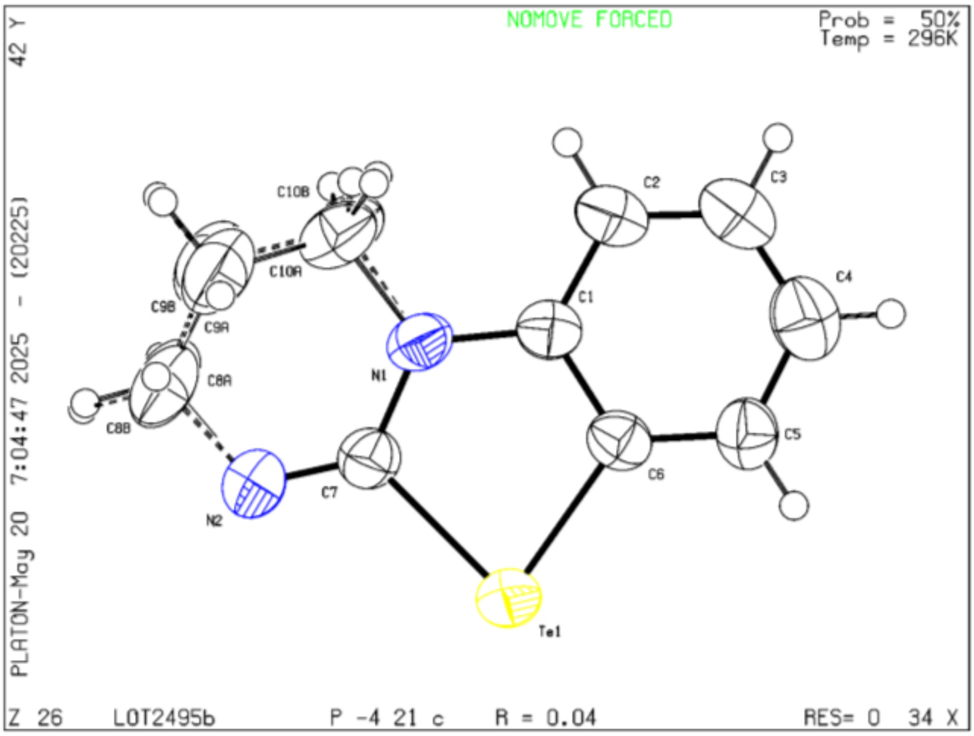

Figure S1: Structure of TeDHPB.

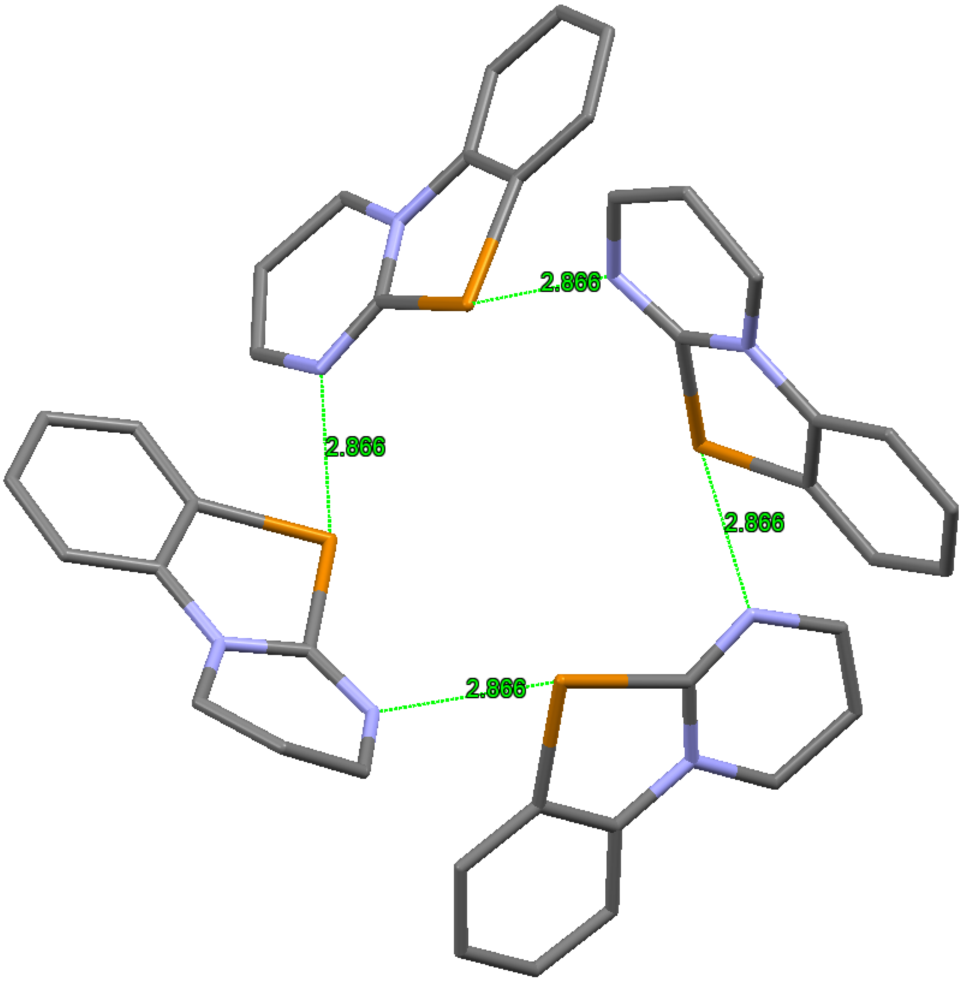

Figure S2: Formation of tetramers via chalcogen bonds in the solid state for TeDHPB.

8. Reactions between IChUs and Benzhydrylium Ions: Product Studies with Representative Examples

For the product studies, the respective catalyst (0.05 mmol) and benzhydrylium ion (0.05 mmol) were mixed well in 0.7 mL CD_2Cl_2 and ^{1}H-, ^{13}C- and ^{77}Se-NMR were taken. As the compounds were used in stochiometric fashion, there was an equilibrium between reactants and product (also see determination of equilibrium constants and Lewis basicity). Hence, the products were not isolated and characterized by NMR only. More signals were visible in the spectra (both unreacted starting materials and the desired adducts). Data for the products of three representative examples are given here (Se*Hyper*BTM, SeBTM and SeTM with benzhydrylium ion (dma)$_2$CH$^+$BF$_4^-$ each).

SeTM-dma (19a)

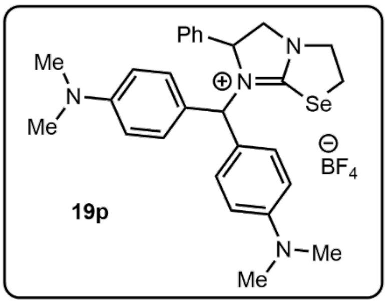

^{1}H-NMR (500 MHz, CD_2Cl_2, 298 K): *δ* / ppm = 7.59-7.55 (m, 3 H, Ar-**H**), 7.48-7.38 (m, 2 H, Ar-**H**), 7.04 (d, *J* = 8.8 Hz, 2 H, Ar-**H**), 6.95 (d, *J* = 8.8 Hz, 2 H, Ar-**H**), 6.82 (d, *J* = 8.8 Hz, 2 H, Ar-**H**), 6.64 (d, *J* = 8.8 Hz, 2 H, Ar-**H**), 5.29 (t, *J* = 10.3 Hz, 1 H, -C**H**), 5.24 (s, 1 H, -C**H**), 4.37 (t, *J* = 10.8 Hz, 1 H, -C**H2**), 4.02-3.99 (m, 1 H, -C**H2**), 3.88 (t, *J* = 10.5 Hz, 1 H, -C**H2**), 3.81-3.72 (m, 2 H, -C**H2**), 3.66-3.63 (m, 1 H, -C**H2**), 3.04 (s, 6 H, -NC**H3**), 2.98 (s, 6 H, -NC**H3**).

^{13}C-NMR (125 MHz, $CDCl_3$, 298 K): *δ* / ppm = 173.8 (1 C, -N=**C**), 151.4 (1 C, **C**Ar), 150.9 (1 C, **C**Ar), 134.9 (1 C, **C**Ar), 131.8 (2 C, **C**Ar), 130.3 (1 C, **C**Ar), 129.8 (2 C, **C**Ar), 128.4 (2 C, **C**Ar), 128.1 (2 C, **C**Ar), 121.0 (1 C, **C**Ar), 120.0 (1 C, **C**Ar), 112.7 (2 C, **C**Ar), 111.7 (2 C, **C**Ar), 70.7 (1 C, -**C**H), 63.7 (1 C, -**C**H2), 55.4 (1 C, -**C**H2), 48.4 (1 C, -**C**H2), 40.1 (2 C, -N**C**H3), 39.9 (2 C, -N**C**H3), 30.4 (1 C, -**C**H).

^{77}Se-NMR (95 MHz, $CDCl_3$, 298 K): *δ* / ppm = 336.0 (1 Se, -C**Se**C-).

SeBTM-dma (19q)

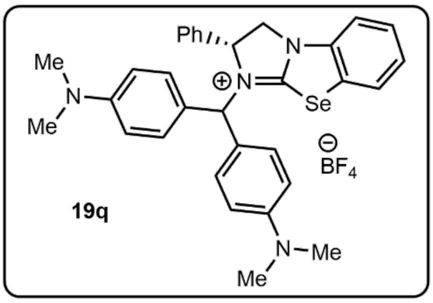

^{1}H-NMR (500 MHz, CD_2Cl_2, 298 K): *δ* / ppm = 7.92 (d, *J* = 8.7Hz, 1 H, Ar-**H**), 7.60-7.58 (m, 3 H, Ar-**H**), 7.45-7.35 (m, 5 H, Ar-**H**), 7.11 (d, *J* = 8.7 Hz, 2 H, Ar-**H**), 7.07 (d, *J* = 8.7 Hz, 2 H, Ar-**H**), 6.82 (d, *J* = 8.8 Hz, 2 H, Ar-**H**), 6.72 (d, *J* = 8.7 Hz, 2 H, Ar-**H**), 5.57 (dd, J_1 = 8.9 Hz, J_2 = 11.0 Hz, 1 H, -C**H**), 5.40 (s, 1 H, -C**H**), 5.14 (t, *J* = 11.3 Hz, 1 H, -C**H2**), 4.57 (dd, J_1 = 8.8 Hz, J_2 = 11.2 Hz, 1 H, -C**H2**), 3.04 (s, 12 H, -NC**H3**).

^{13}C-NMR (125 MHz, $CDCl_3$, 298 K): *δ* / ppm = 169.0 (1 C, -N=**C**), 161.5 (1 C, **C**Ar), 157.1 (1 C, **C**Ar), 151.7 (1 C, **C**Ar), 151.1 (1 C, **C**Ar), 134.9 (1 C, **C**Ar), 134.4 (1 C, **C**Ar), 131.8 (2 C, **C**Ar), 130.5 (1 C, **C**Ar), 129.9 (2 C, **C**Ar), 128.7 (1 C, **C**Ar), 128.3 (2 C, **C**Ar), 127.9 (2 C, **C**Ar), 126.1 (1 C, **C**Ar), 125.6 (1 C, CAr), 120.6 (1 C, **C**Ar), 113.3 (1 C, **C**Ar), 112.7 (2 C, **C**Ar), 112.1 (2 C, **C**Ar), 69.2 (1 C, -**C**H), 64.1 (1 C, -**C**H2), 40.9 (1 C, -**C**H), 40.1 (2 C, -N**C**H3), 39.9 (2 C, -N**C**H3).

^{77}Se-NMR (95 MHz, $CDCl_3$, 298 K): *δ* / ppm = 464.7 (1 Se, -C**Se**C-).

Se*Hyper*BTM-dma (19r)

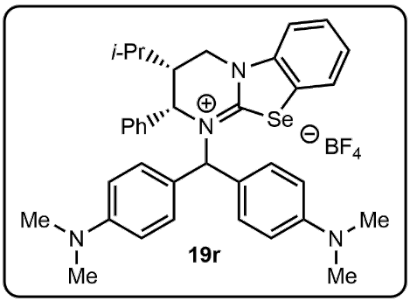

^{1}H-NMR (500 MHz, CD_2Cl_2, 298 K): δ / ppm = 7.92 (d, J = 9.5Hz, 1 H, Ar-**H**), 7.76 (d, J = 7.9 Hz, 1 H, Ar-**H**), 7.67-7.59 (m, 2 H, Ar-**H**), 7.52-7.45 (m, 5 H, Ar-**H**), 7.09-7.06 (m 4 H, Ar-**H**), 7.71-6.67 (m, 4 H, Ar-**H**), 5.91 (s, 1 H, -C**H**), 4.99 (d, J = 3.8 Hz, 1 H, -C**H**), 4.47 (dd, J_1 = 4.8 Hz, J_2 = 12.7 Hz, 1 H, -C**H**$_2$), 3.84 (t, J = 12.7 Hz, 1 H, -C**H**$_2$), 3.03 (s, 6 H, -NC**H**$_3$), 3.01 (s, 6 H, -NC**H**$_3$), 2.24-2.20 (m, 1 H, -C**H**), 1.42-1.38 (m, 1 H, -C**H**), 1.08 (d, J = 6.5 Hz, 3 H, -C**H**$_3$), 0.96 (d, J = 6.7 Hz, 3 H, -C**H**$_3$).

^{13}C-NMR (125 MHz, $CDCl_3$, 298 K): δ / ppm = 166.1 (1 C, -N=**C**), 151.7 (1 C, **C**$_{Ar}$), 151.1 (1 C, **C**$_{Ar}$), 138.8 (1 C, **C**$_{Ar}$), 134.5 (1 C, **C**$_{Ar}$), 131.8 (2 C, **C**$_{Ar}$), 129.6 (1 C, **C**$_{Ar}$), 129.5 (2 C, **C**$_{Ar}$), 129.0 (2 C, **C**$_{Ar}$), 128.4 (1 C, **C**$_{Ar}$), 127.8 (2 C, **C**$_{Ar}$), 126.3 (1 C, **C**$_{Ar}$), 125.1 (1 C, **C**$_{Ar}$), 120.5 (1 C, **C**$_{Ar}$), 118.7 (1 C, **C**$_{Ar}$), 114.4 (1 C, **C**$_{Ar}$), 114.0 (1 C, **C**$_{Ar}$), 112.3 (2 C, **C**$_{Ar}$), 112.1 (2 C, **C**$_{Ar}$), 74.0 (1 C, -**C**H), 64.7 (1 C, -**C**H$_2$), 44.7 (1 C, -**C**H), 40.9 (1 C, -**C**H), 40.0 (2 C, -N**C**H$_3$), 39.9 (2 C, -N**C**H$_3$), 26.8 (1 C, -**C**H), 21.0 (1 C, -**C**H$_3$), 19.3 (1 C, -**C**H$_3$).

^{77}Se-NMR (95 MHz, $CDCl_3$, 298 K): δ / ppm = 519.9 (1 Se, -C**Se**C-).

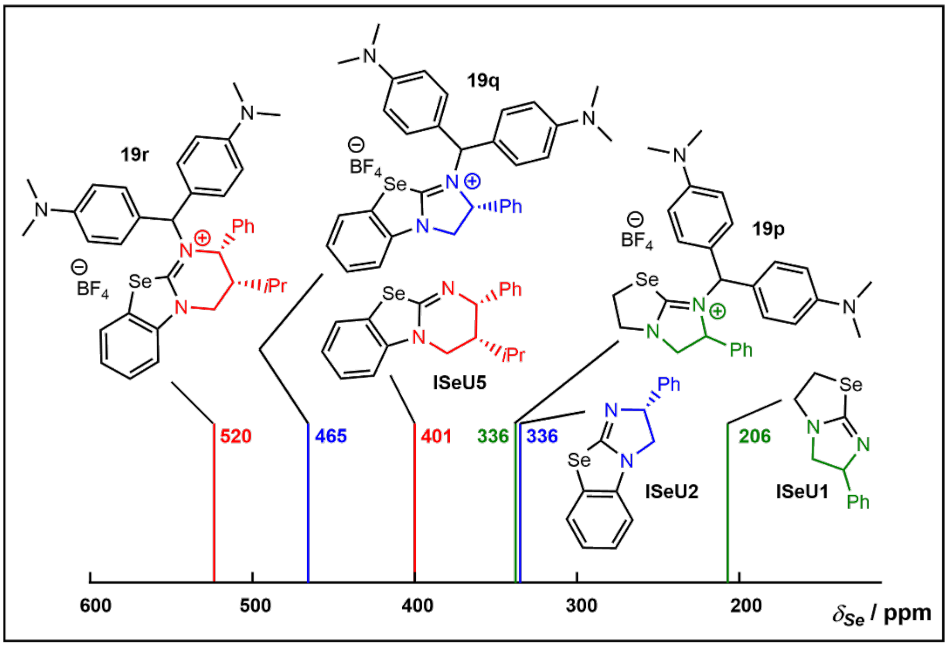

Figure S3: Comparison of ^{77}Se chemical shifts of the catalysts and catalyst-benzhydrylium adducts.

9. Reactivity towards Allenoates

9.1 General Procedure F for the Synthesis of Catalyst Hydrochloride Salts

According to a known procedure[17], the respective catalyst (1 eq) was suspended in Et_2O (0.05 mol L^{-1}). Then, HCl (4 M in dioxane, 2 eq) was added. The mixture was stirred at r.t. for 10 min after which excess solvent was removed under reduced pressure. The hydrochloride salts were collected as white solids.

9.2 General Procedure G for the Synthesis of the Free Base Catalysts

The respective catalyst hydrochloride salt was taken up in a sat. aq. Na_2CO_3 solution. This was extracted with Et_2O six times under vigorous shaking. The combined organic phases were dried over Na_2SO_4, filtered and concentrated. The free base catalysts were obtained as white to yellow solids (*Note: this procedure was used to make sure that no "accidentally" partially protonated catalyst species was used*).

9.3 Comparison of NMR data for Free Base Catalysts and their Hydrochloride Salts

In any case, NMR signals for the hydrochloride salts tend to shift more downfield in NMR spectra. During the reactions between catalysts and allenoates, mixtures between the catalyst and the hydrochloride salts are present. In those cases, an average of the respective signals is visible, with chemical shifts between the signals for the free base catalyst and the respective hydrochloride salt.

***Hyper*BTM / *Hyper*BTM·HCl**

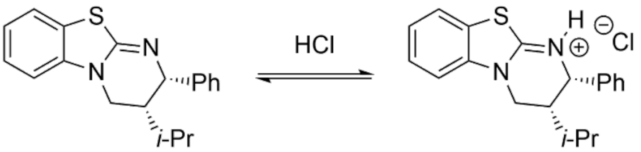

***Hyper*BTM**

^{1}H-NMR (300 MHz, $CDCl_3$, 298 K): δ / ppm = 7.35-7.20 (m, 7 H, Ar-**H**), 7.61 (t, J = 7.6 Hz, 1 H, Ar-**H**), 6.81 (d, J = 8.00 Hz, 1 H, Ar-**H**), 4.93 (d, J = 3.3 Hz 1 H, -C**H**), 3.88 (dd, J_1 = 5.2 Hz, J_2 = 10.8 Hz, 1 H, -C**H$_2$**), 3.36 (t, J = 11.5 Hz, 1 H, -C**H$_2$**), 2.00-1.93 (m, 1 H, -C**H**), 1.38-1.26 (m, 1 H, -C**H**), 1.14 (d, J = 6.4 Hz, 3 H, -C**H$_3$**), 0.85 (d, J = 6.6 Hz, 3 H, -C**H$_3$**).

*Hyper*BTM·HCl

¹H-NMR (300 MHz, $CDCl_3$, 298 K): *δ* / ppm = 7.62 (d, *J* = 7.7 Hz, 1 H, Ar-**H**), 7.48 (t, *J* = 7.4 Hz, 1 H, Ar-**H**), 7.37-7.32 (m, 4 H, Ar-**H**), 7.26-7.22 (m, 1 H, Ar-**H**), 7.12-7.09 (m, 2 H, Ar-**H**), 5.15 (d, *J* = 3.8 Hz, 1 H, -C**H**), 4.19 (dd, J_1 = 4.3 Hz, J_2 = 12.2. Hz, 1 H, -C**H**$_2$), 3.61 (t, *J* = 12.2 Hz, 1 H, -C**H**$_2$), 2.16-2.06 (m, 1 H, -C**H**), 1.40-1.33 (m, 1 H, -C**H**), 1.19 (d, *J* = 6.4 Hz, 3 H, -C**H**$_3$), 0.91 (d, *J* = 6.7 Hz, 3 H, -C**H**$_3$).

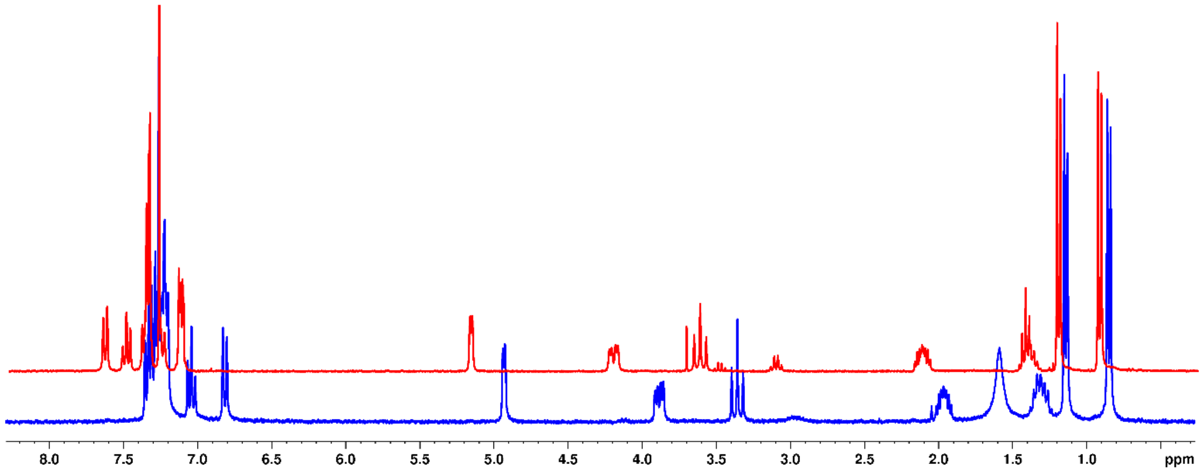

BTM / BTM · HCl

Figure S4: Comparison between NMR data for *Hyper*BTM (blue) and *Hyper*BTM·HCl (red).

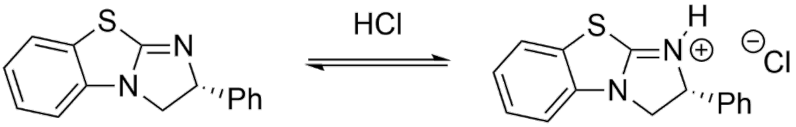

BTM

¹H-NMR (300 MHz, $CDCl_3$, 298 K): *δ* / ppm = 7.38-7.29 (m, 6 H, Ar-**H**), 7.19 (td, J_1 = 1.1 Hz, J_2 = 7.7 Hz, 1 H, Ar-**H**), 6.97 (td, J_1 = 1.1 Hz, J_2 = 7.7 Hz, 1 H, Ar-**H**), 6.67 (dd, J_1 = 0.7 Hz, J_2 = 7.8 Hz, 1 H, Ar-**H**), 5.67 (dd, J_1 = 8.1 Hz, J_2 = 10.2 Hz, 1 H, -C**H**), 4.28 (dd, J_1 = 8.9 Hz, J_2 = 10.2 Hz, 1 H, -C**H**$_2$), 3.72 (dd, J_1 = 8.1 Hz, J_2 = 8.9 Hz, 1 H, -C**H**$_2$).

BTM · HCl

¹H-NMR (300 MHz, $CDCl_3$, 298 K): *δ* / ppm = 7.67 (App. d, *J* = 8.0 Hz, 1 H, Ar-**H**), 7.49 (td, J_1 = 1.0 Hz, J_2 =7.8 Hz, 1 H, Ar-**H**), 7.44-7.31 (m, 6 H, Ar-**H**), 7.22 (d, *J* = 7.7 Hz, 1 H, Ar-**H**), 6.14 (dd, J_1 = 8.0 Hz, J_2 = 10.6 Hz, 1 H, -C**H**), 5.09 (App. t, *J* = 10.6 Hz, 1 H, -C**H**$_2$), 4.28 (dd, J_1 = 8.0 Hz, J_2 = 10.2 Hz, 1 H, -C**H**$_2$).

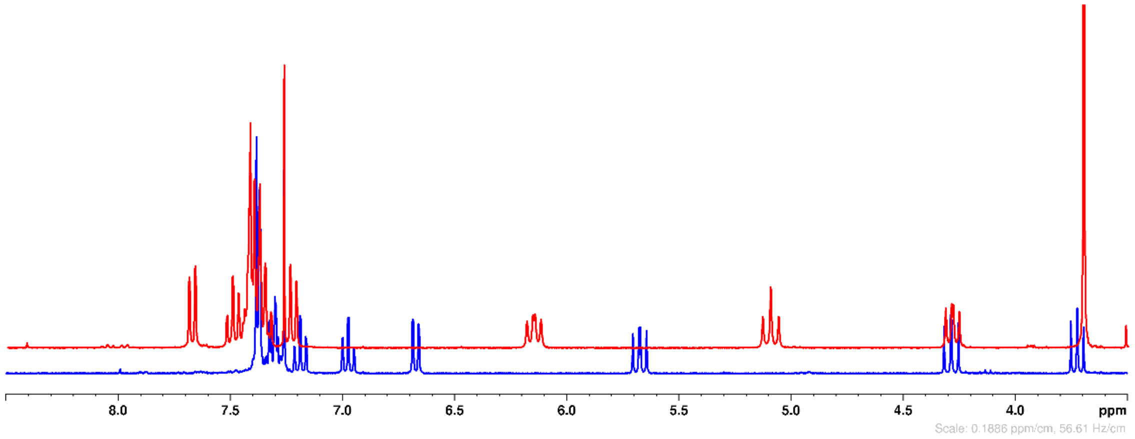

Figure S5: Comparison between NMR data for BTM (blue) and BTM·HCl (red).

HBTM / HBTM · HCl

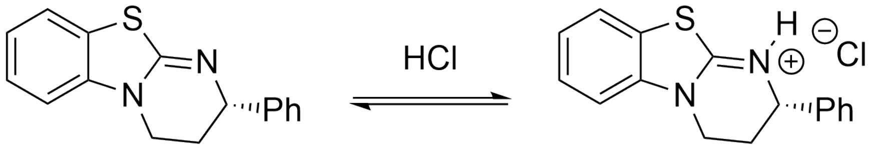

HBTM

^{1}H-NMR (300 MHz, $CDCl_3$, 298 K): *δ* / ppm = 7.35-7.18 (m, 7 H, Ar-**H**), 7.02 (td, J_1 = 1.0 Hz, J_2 = 7.6 Hz, 1 H, Ar-**H**), 6.76 (d, *J* = 7.6 Hz, 1 H, Ar-**H**), 4.74 (dd, J_1 = 4.1 Hz, J_2 = 7.9 Hz, 1 H, -C**H**), 3.84 (ddd, J_1 = 4.9 Hz, J_2= 8.5 Hz, J_3 = 11.7 Hz, 1 H, -C**H$_2$**), 3.73-3.66 (m, 1 H, -C**H$_2$**), 2.36-2.27 (m, 1 H, -C**H$_2$**), 2.06-1.94 (m, 1 H, -C**H$_2$**).

HBTM · HCl

^{1}H-NMR (300 MHz, $CDCl_3$, 298 K): *δ* / ppm = 7.52 (d, *J* = 7.4 Hz, 1 H, Ar-**H**), 7.43-7.24 (m, 7 H, Ar-**H**), 7.13 (d, *J* = 8.1 Hz, 1 H, Ar.**H**), 5.07 (t, *J* = 5.0 Hz, 1 H, -C**H**), 4.22-4.15 (m 1 H, -C**H$_2$**), 3.87-3.78 (m 1 H, -C**H$_2$**), 2.52-2.42 (m 1 H, -C**H$_2$**), 2.32-2.22 (m, 1 H, -C**H$_2$**).

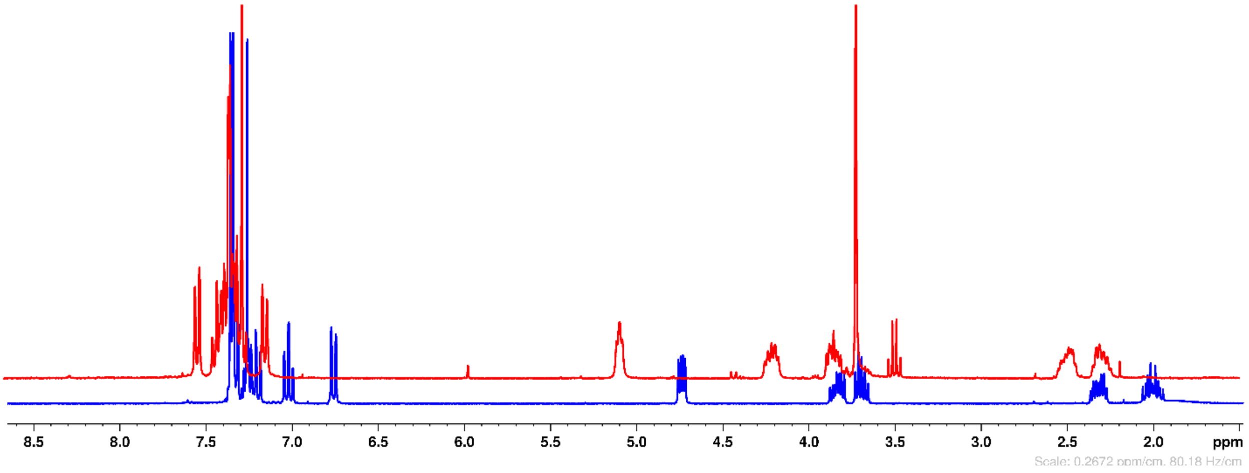

FigureS6: Comparison between NMR data for HBTM (blue) and HBTM·HCl (red).

TM / TM · HCl

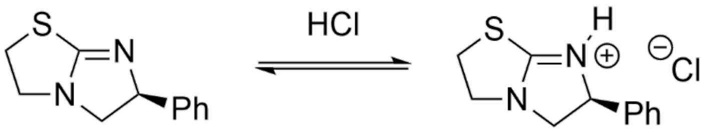

TM

[1]**H-NMR** (300 MHz, $CDCl_3$, 298 K): *δ* / ppm = 7.35-7.22 (m, 5 H, Ar-H), 5.46 (App. t, *J* = 5.5 Hz, 1 H, -CH), 3.71-3.61 (m, 2 H, $-CH_2$), 3.53 (ddd, J_1 = 4.4 Hz, J_2 = 6.4 Hz, J_3 = 10.8 Hz, 1H, $-CH_2$), 3.37 (ddd, J_1 = 4.4 Hz, J_2 = 6.4 Hz, J_3 = 8.6 Hz, 1 H, $-CH_2$), 3.13 (td, J_1 = 6.4 Hz, J_2 = 8.6 Hz, 1 H, $-CH_2$), 2.99 (dd, J_1 = 8.4 Hz, J_2 =9.1 Hz, 1 H, $-CH_2$).

TM · HCl

[1]**H-NMR** (300 MHz, $CDCl_3$, 298 K): *δ* / ppm = 7.46-7.36 (m, 5 H, Ar-**H**), 5.80 (App. t, *J* = 9.3 Hz, 1 H, -C**H**), 4.27 (t, *J* = 9.9 Hz, 1 H, -C**H₂**), 3.98-3.80 (m, 3 H, -C**H₂**), 3.74-3.61 (m, 2 H, -C**H₂**).

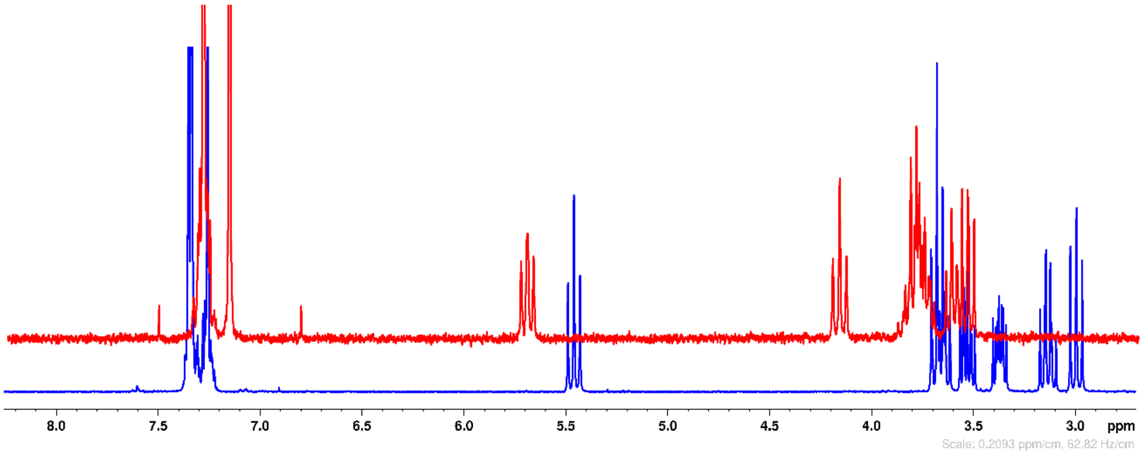

Figure S7: Comparison between NMR data for TM (blue) and TM·HCl (red).

O*Hyper*BTM / O*Hyper*BTM·HCl

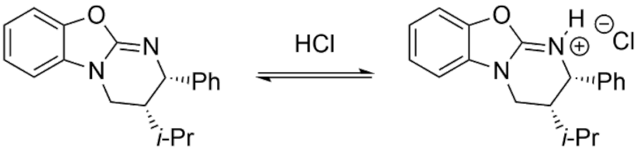

O*Hyper*BTM

^{1}H-NMR (400 MHz, $CDCl_3$, 298 K) δ / ppm = 7.32-7.25 (m, 2 H, Ar-**H**), 7.22-7.19 (m, 3 H, Ar-**H**), 7.16-7.09 (m, 2 H, Ar-**H**), 7.01 (td, J_1 = 1.2 Hz, 1 H, J_2 = 7.8 Hz, 1 H, Ar-**H**), 6.83 (d, J = 7.7 Hz, 1 H, Ar-**H**), 4.95 (d, J = 3.2 Hz, 1 H, -C**H**), 3.88 (ddd, J_1 = 1.5 Hz, J_2 = 5.4 Hz, J_3 = 11.5 Hz, 1 H, -C**H$_2$**), 3.43 (t, J = 11.3 Hz, 1 H, -C**H$_2$**), 1.98-1.90 (m, 1 H, -C**H**), 1.35-1.24 (m, 1 H, -C**H**), 1.13 (d, J = 6.5 Hz, 3 H, -C**H$_3$**), 0.83 (d, J = 6.7 Hz, 3 H, Ar-**H**).

O*Hyper*BTM·HCl

^{1}H-NMR (300 MHz, $CDCl_3$, 298 K): δ / ppm = 7.51 (d, J = 7.8 Hz, 1 H, Ar-**H**), 7.47-7.31 (m, 6 H, Ar-**H**), 7.11-7.09 (m, 2 H, Ar-**H**), 5.25 (App. s, 1 H, -C**H**), 4.25 (dd, J_1 = 3.4 Hz, J_2 = 12.2 Hz, 1 H, -C**H$_2$**), 3.73-3.65 (m, 1 H, -C**H$_2$**), 2.15-2.09 (m, 1 H, -C**H**), 1.41-1.29 (m, 1 H, -C**H**), 1.16 (d, J = 6.4 Hz, 3 H, -C**H$_3$**), 0.90 (d, J = 6.6 Hz, 3 H, -C**H$_3$**).

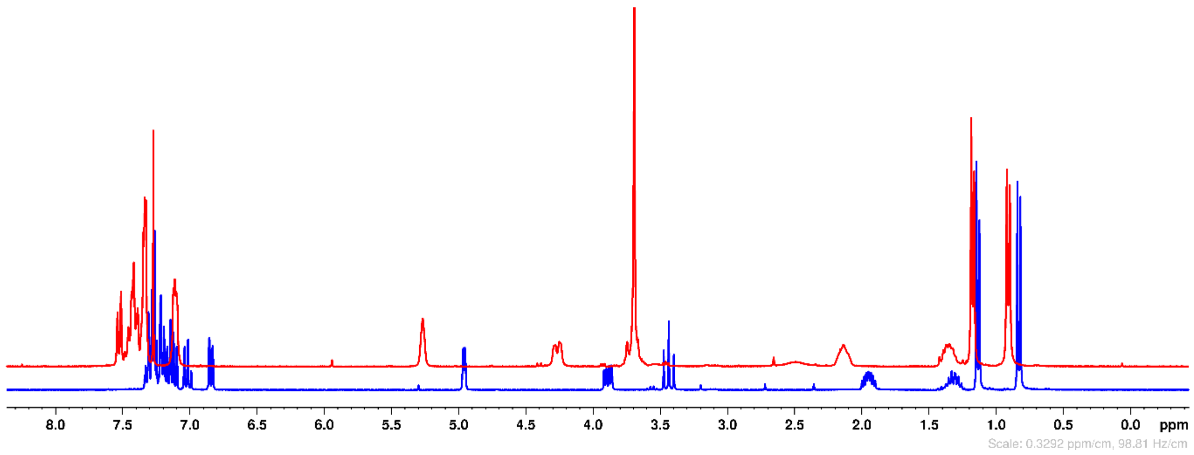

Figure S8: Comparison between NMR data for O*Hyper*BTM (blue) and O*Hyper*BTM ·HCl (red).

<u>Se*Hyper*BTM / Se*Hyper*BTM·HCl</u>

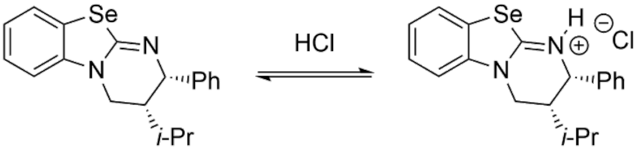

Se*Hyper*BTM

^{1}H-NMR (300 MHz, $CDCl_3$, 298 K) δ / ppm = 7.42 (dd, J_1 = 1.0 Hz, J_2 = 7.6 Hz, 1 H, Ar-**H**), 7.34-7.21 (m, 6 H, Ar-**H**), 7.02 (td, J_1 = 0.9 Hz, J_2 = 7.6 Hz, 1 H, Ar-**H**), 6.81 (d, J = 8.0 Hz, 1 H, Ar-**H**), 4.94 (dd, J_1 = 1.5 Hz, J_2 = 4.3 Hz, 1 H, -C**H**), 3.86 (ddd, J_1 = 1.5 Hz, J_2 = 5.2 Hz, J_3 = 11.6 Hz, 1 H, -C**H$_2$**), 3.34 (App. t, J = 11.6 Hz, 1 H, -C**H$_2$**), 2.05-1.95 (m, 1 H, -C**H**), 1.37-1.26 (m, 1 H, -C**H**), 1.14 (d, J = 6.6 Hz, 3 H, -C**H$_3$**), 0.85 (d, J = 6.6 Hz, 3 H, -C**H$_3$**).

Se*Hyper*BTM·HCl

^{1}H-NMR (300 MHz, $CDCl_3$, 298 K): δ / ppm = 7.56 (d, J = 7.6 Hz, 1 H, Ar-**H**), 7.39-7.28 (m, 4 H, Ar-**H**), 7.18-7.14 (m, 3 H, Ar-**H**), 7.01 (d, J = 8.0 Hz, 1 H, Ar-**H**), 5.02 (dd, J_1 = 1.3 Hz, J_2 = 4.6 Hz, 1 H, -C**H**), 4.02 (dd, J_1 = 4.6 Hz, J_2 = 11.6 Hz, 1 H, -C**H$_2$**), 3.46 (t, J = 11.9 Hz, 1 H, -C**H$_2$**), 2.11-2.01 (m, 1 H, -C**H**), 1.43-1.27 (m, 1 H, -C**H**), 1.16 (d, J = 6.4 Hz, 3 H, -C**H$_3$**), 0.88 (d, J = 6.6 Hz, 3 H, -C**H$_3$**).

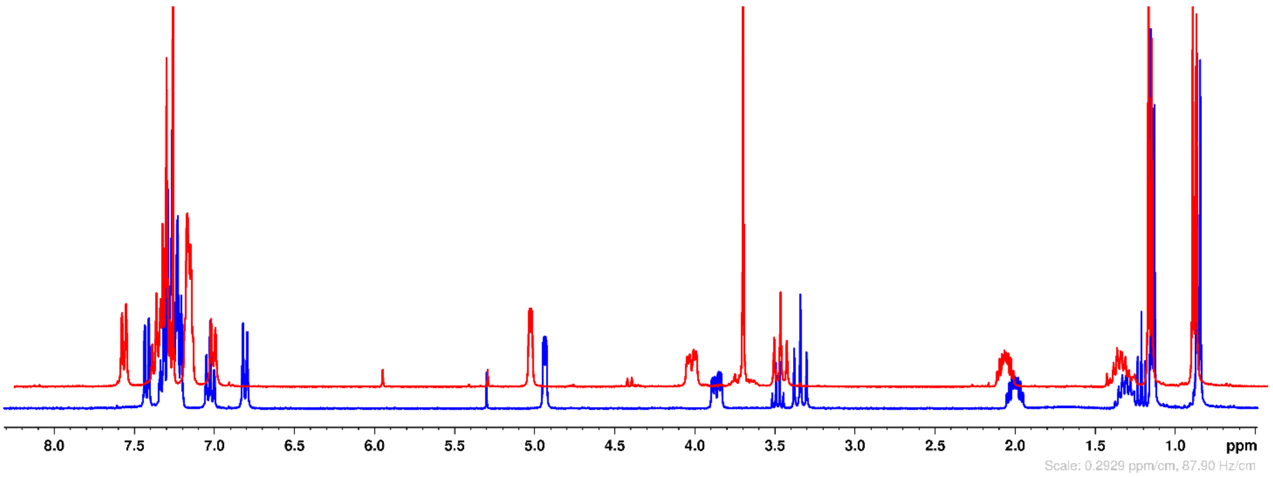

Figure S9: Comparison between NMR data for Se*Hyper*BTM (blue) and Se*Hyper*BTM ·HCl (red).

TeDHPB / TeDHPB·HCl

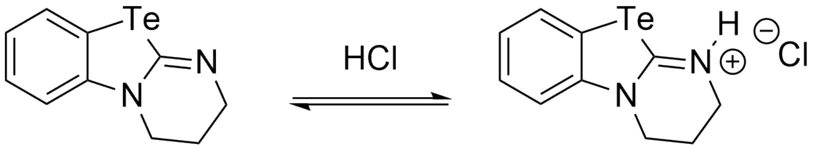

TeDHPB

¹H-NMR (300 MHz, $CDCl_3$, 298 K): *δ* / ppm = 7.43 (dd, J_1 = 1.3 Hz, J_2 = 7.5 Hz, 1 H, Ar-**H**), 7.24 (ddd, J_1 = 1.3 Hz, J_2 = 7.5 Hz, J_3 = 8.2 Hz, 1 H, Ar-**H**), 6.97 (td, J_1 = 1.1 Hz, J_2 = 7.5 Hz, 1 H, Ar-**H**), 6.78 (d, *J* = 8.3 Hz, 1 H, Ar-**H**), 3.77 (t, *J* = 6.1 Hz, 2 H, -C**H₂**), 3.64 (t, *J* = 5.7 Hz, 2 H, -C**H₂**), 2.13 (quint., *J* = 5.9 Hz, 1 H, -C**H₂**).

TeDHPB·HCl

The compound was hardly soluble in $CDCl_3$.

¹H-NMR (300 MHz, $CDCl_3$, 298 K): *δ* / ppm = 7.74 (App. d, *J* = 7.7 Hz, 1 H, Ar-**H**), 7.41 (td, J_1 = 1.2 Hz, J_2 = 7.6 Hz, 1 H, Ar-**H**), 7.26-7.21 (m, 2 H, Ar-**H**), 4.12 (t, *J* = 6.2 Hz, 2 H, -C**H₂**), 3.74-3.73 (br., 2 H, -C**H₂**), 2.38-2.34 (m, 1 H, -C**H₂**).

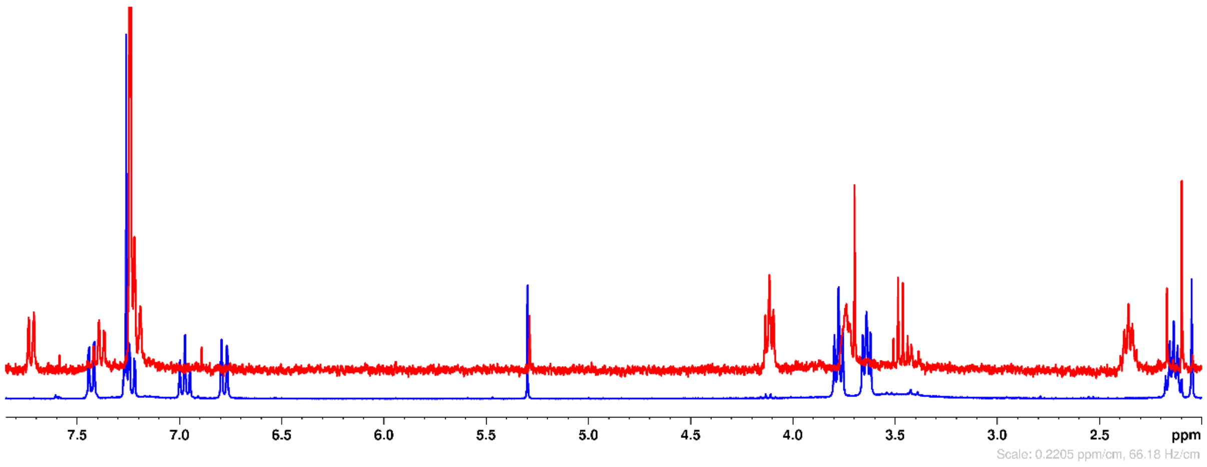

Figure S10: Comparison between NMR data for TeDHPB (blue) and TeDHPB ·HCl (red).

9.4 Trapping Experiments

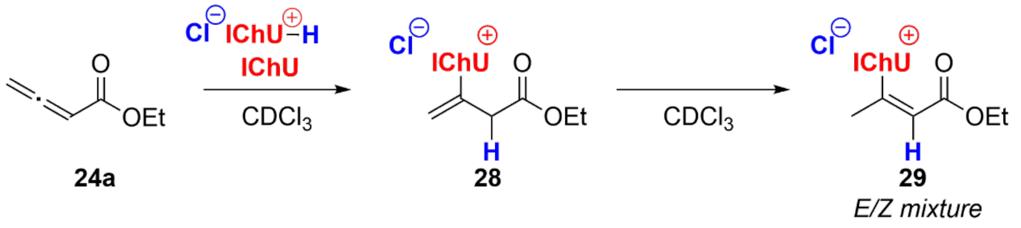

For the trapping experiments, the respective catalysts (0.05 mmol, 1 eq), their hydrochloride salts (0.05 mmol, 1 eq) and allenoate **24a** (0.05 mmol, 5.8 µL) were suspended in 0.6 mL $CDCl_3$. Then, mesitylene (0.05 mmol, 1 eq) was added as internal standard and the mixture was transferred into an NMR tube. Repeated proton NMR spectra were measured over the curse of 150000s (4.2 h). The recording of new spectra was automated, so that the sample always remained in the spectrometer. The list from Table S3 was used for repeated measurements.

In the beginning, consumption of the allenoate and formation of the adduct with terminal alkene **28** was visible. Depending on the catalyst, after a certain time, isomerization to the adduct with an internal alkene **29** (*E*/*Z* mixture) was predominant.

The decease of allenoate concentration was observed via referencing the allenoate signals to the ones for the internal standard, mesitylene. In the beginning of the reaction, the concentration of the allenoate decreased, while the concentration of the product increased. The integral of the allenoate + the integral of product equaled the integral of the internal standard for roughly the first hour of the reaction. Then, the allenoate and/or adduct started to decompose. The time of beginning of the decomposition is also the time where the isomerization starts to occur.

In the following, detailed data is given for the trapping reaction performed with *Hyper*BTM, DHPB and BTM. For the other catalysts, the decrease in allenoate concentration is given. For HyperBTM, exemplary NMR spectra are given as well. Spectra looked similar for all other catalysts as well. Due to overlap, no clear assignment of all signals could be made. Unfortunately, we did not succeed in isolating the catalyst bound species yet.

For the reaction of TeDHPB with allenoate **24a**, no decrease of allenoate signals could be observed as the reaction was already complete upon recording the first spectrum.

Table S3: Time, after which the next NMR measurement was performed. 0 refers to the start of the first measurement. Until the start of the first measurement, roughly 5 min passed for mixing and setting up the NMR experiment (locking, shimming, tuning and matching).

Measurement	Time delay (s)	Time total (s)
1	0	0
2	300	300
3	300	600
4	300	900
5	300	1200
6	300	1500
7	300	1800
8	600	2400
9	600	3000
10	600	3600
11	600	4200
12	600	4800
13	600	5400
14	1200	6600
15	1200	7800
16	1200	9000
17	1200	10200
18	1200	11400
19	1800	13200
20	1800	15000

*Hyper*BTM – Tabulated Integrals

Table S4: Detailed evaluation of the data obtained for the trapping experiment using *Hyper*BTM. Mesitylene was used as internal standard. 0 refers to the start of the first measurement. Until the start of the first measurement, roughly 5 min passed for mixing and setting up the NMR experiment (locking, shimming, tuning, and matching).

time added	time total	Integral ISTD	Number protons ISTD	Calib.	Integral Allenoate	Protons Allenoate	Calib.	Integral terminal DB	Protons terminal DB	Calib.	Integral E/Z mix	Protons E/Z mix	Calib.	Sum Allenoate + Adduct
0	0	3	3	1	0.72	1.00	0.72	0.28	1.00	0.28	0.00	1.00	0.00	1.00
300	300	3	3	1	0.62	1.00	0.62	0.38	1.00	0.38	0.00	1.00	0.00	1.00
300	600	3	3	1	0.53	1.00	0.53	0.48	1.00	0.48	0.00	1.00	0.00	1.02
300	900	3	3	1	0.46	1.00	0.46	0.57	1.00	0.57	0.00	1.00	0.00	1.03
300	1200	3	3	1	0.39	1.00	0.39	0.63	1.00	0.63	0.00	1.00	0.00	1.02
300	1500	9	9	1	0.32	1.00	0.32	0.66	1.00	0.66	0.00	1.00	0.00	0.98
300	1800	9	9	1	0.28	1.00	0.28	0.73	1.00	0.73	0.00	1.00	0.00	1.01
600	2400	9	9	1	0.22	1.00	0.22	0.53	1.00	0.53	0.22	1.00	0.22	0.96
600	3000	9	9	1	0.13	1.00	0.13	0.21	1.00	0.21	0.49	1.00	0.49	0.83
600	3600	9	9	1	0.10	1.00	0.10	0.17	1.00	0.17	0.52	1.00	0.52	0.78
600	4200	9	9	1	0.10	1.00	0.10	0.17	1.00	0.17	0.52	1.00	0.52	0.78
600	4800	9	9	1	0.08	1.00	0.08	0.15	1.00	0.15	0.55	1.00	0.55	0.78
600	5400	9	9	1	0.06	1.00	0.06	0.13	1.00	0.13	0.54	1.00	0.54	0.73
1200	6600	9	9	1	0.04	1.00	0.04	0.13	1.00	0.13	0.54	1.00	0.54	0.71
1200	7800	9	9	1	0.03	1.00	0.03	0.12	1.00	0.12	0.55	1.00	0.55	0.70
1200	9000	9	9	1	0.02	1.00	0.02	0.11	1.00	0.11	0.54	1.00	0.54	0.68
1200	10200	9	9	1	0.02	1.00	0.02	0.11	1.00	0.11	0.54	1.00	0.54	0.67
1200	11400	9	9	1	0.02	1.00	0.02	0.11	1.00	0.11	0.55	1.00	0.55	0.68
1800	13200	9	9	1	0.01	1.00	0.01	0.11	1.00	0.11	0.54	1.00	0.54	0.66
1800	15000	9	9	1	0.01	1.00	0.01	0.10	1.00	0.10	0.53	1.00	0.53	0.64

***Hyper*BTM – Graphical Depict of Present Species**

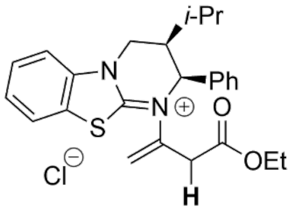

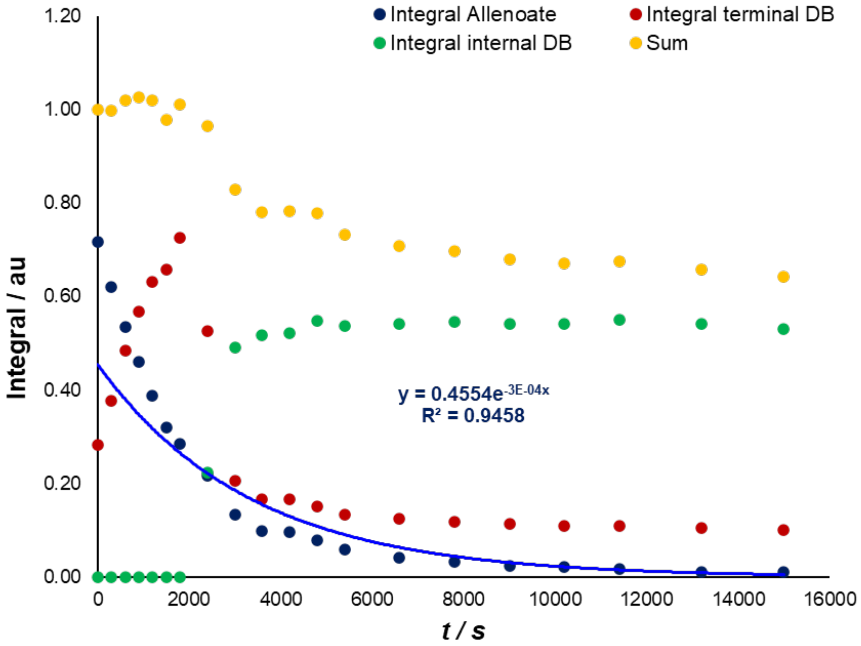

Figure S11: Decrease in allenoate concentration (blue), increase in adduct concentration (green and red) and decomposition (yellow) over the whole timespan investigated. 0 refers to the start of the first measurement. Until the start of the first measurement, roughly 5 min passed for mixing and setting up the NMR experiment (locking, shimming, tuning, and matching).

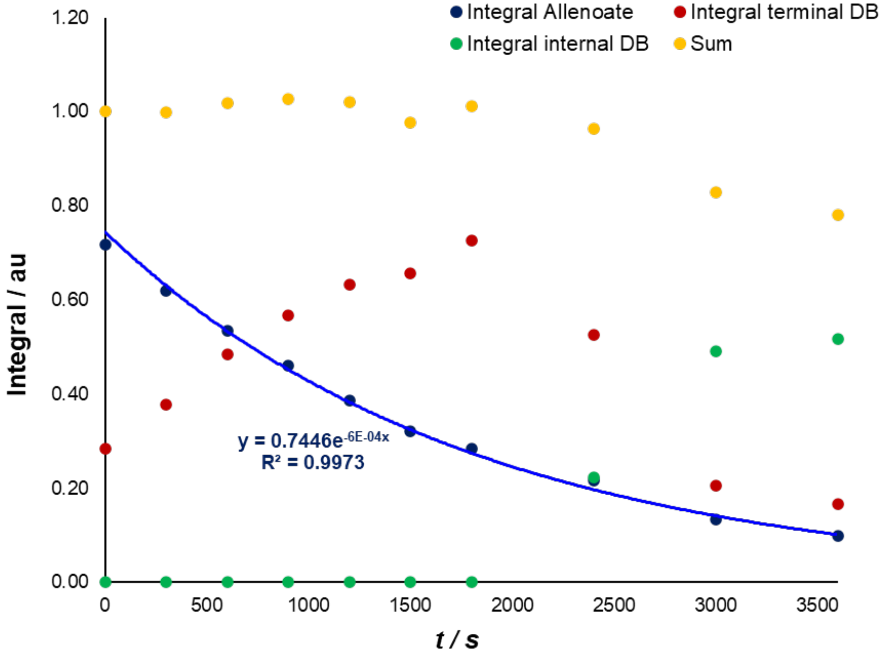

Figure S12: Decrease in allenoate concentration (blue), increase in adduct concentration (green and red) and decomposition (yellow) over the first 1 h investigated. 0 refers to the start of the first measurement. Until the start of the first measurement, roughly 5 min passed for mixing and setting up the NMR experiment (locking, shimming, tuning, and matching).

***Hyper*BTM – Selected NMR Spectra over Time (Representative Example)**

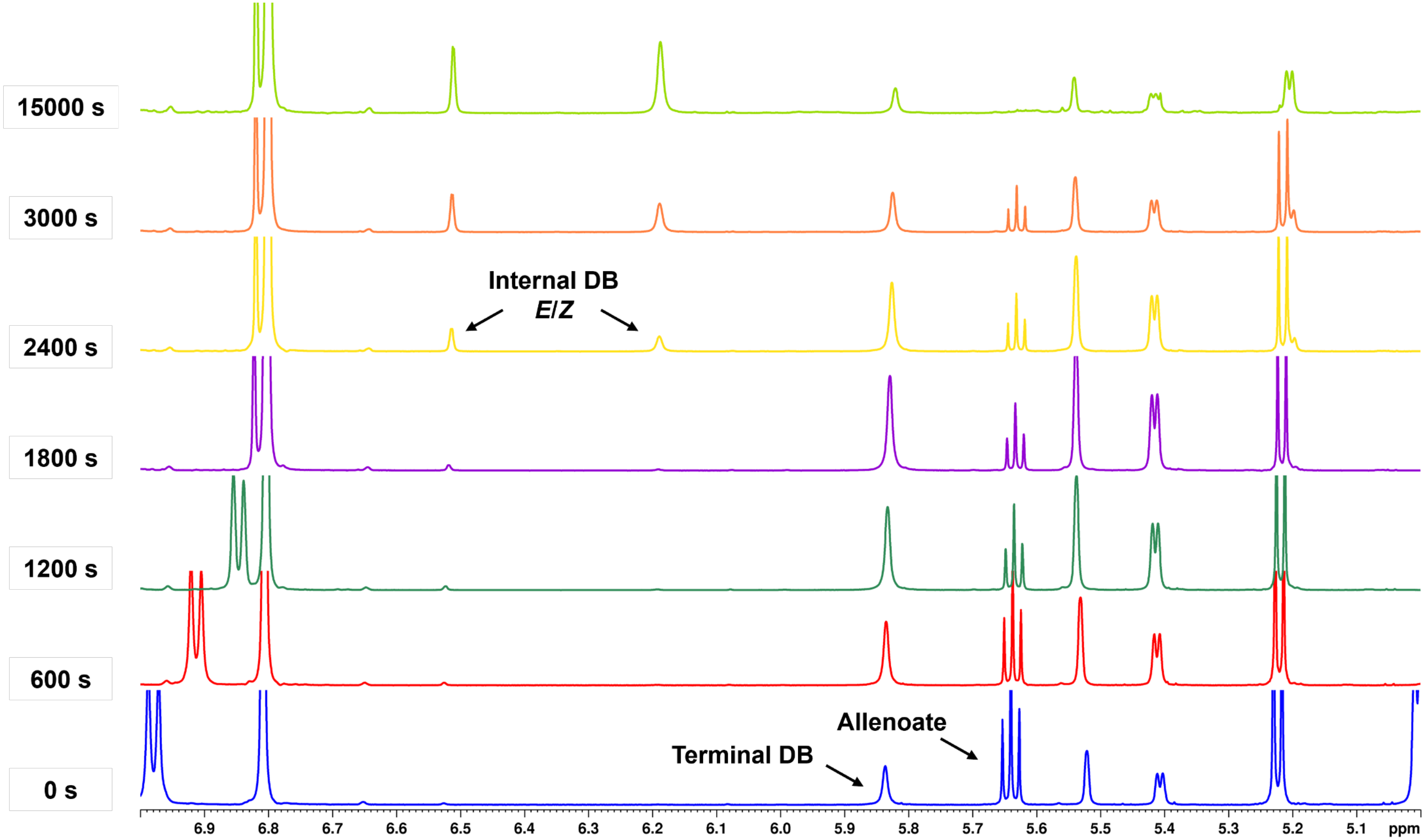

Figure S13: Selected spectra of the trapping experiment. Observed peaks for the allenoate, terminal double bond and internal double bond are marked. 0 refers to the start of the first measurement. Until the start of the first measurement, roughly 5 min passed for mixing and setting up the NMR experiment (locking, shimming, tuning, and matching).

DHPB – Tabulated Integrals

Table S5: Detailed evaluation of the data obtained for the trapping experiment using DHPB. Mesitylene was used as internal standard. 0 refers to the start of the first measurement. Until the start of the first measurement, roughly 5 min passed for mixing and setting up the NMR experiment (locking, shimming, tuning, and matching).

time added	time total	Integral ISTD	Number protons ISTD	Calib.	Integral Allenoate	Protons Allenoate	Calib.	Integral terminal DB	Protons terminal DB	Calib.	Integral E/Z mix	Protons E/Z mix	Calib.	Sum Allenoate + Adduct
0	0	3	3	1	1.51	2.00	0.75	0.28	1.00	0.28	0.00	1.00	0.00	1.03
300	300	3	3	1	1.22	2.00	0.61	0.43	1.00	0.43	0.02	1.00	0.02	1.06
300	600	3	3	1	0.98	2.00	0.49	0.52	1.00	0.52	0.04	1.00	0.04	1.05
300	900	3	3	1	0.77	2.00	0.39	0.62	1.00	0.62	0.06	1.00	0.06	1.07
300	1200	9	9	1	0.63	2.00	0.32	0.66	1.00	0.66	0.07	1.00	0.07	1.04
300	1500	3	3	1	0.53	2.00	0.26	0.73	1.00	0.73	0.10	1.00	0.10	1.09
300	1800	9	9	1	0.42	2.00	0.21	0.74	1.00	0.74	0.12	1.00	0.12	1.08
600	2400	9	9	1	0.25	2.00	0.12	0.09	1.00	0.09	0.69	1.00	0.69	0.90
600	3000	9	9	1	0.13	2.00	0.06	0.09	1.00	0.09	0.68	1.00	0.68	0.83
600	3600	9	9	1	0.07	2.00	0.04	0.09	1.00	0.09	0.63	1.00	0.63	0.75
600	4200	9	9	1	0.04	2.00	0.02	0.09	1.00	0.09	0.60	1.00	0.60	0.71
600	4800	9	9	1	0.03	2.00	0.01	0.09	1.00	0.09	0.59	1.00	0.59	0.69
600	5400	9	9	1	0.02	2.00	0.01	0.08	1.00	0.08	0.57	1.00	0.57	0.66
1200	6600	9	9	1	0.01	2.00	0.01	0.08	1.00	0.08	0.56	1.00	0.56	0.64
1200	7800	9	9	1	0.01	2.00	0.00	0.08	1.00	0.08	0.55	1.00	0.55	0.64
1200	9000	9	9	1	0.01	2.00	0.00	0.08	1.00	0.08	0.55	1.00	0.55	0.63
1200	10200	9	9	1	0.01	2.00	0.00	0.08	1.00	0.08	0.54	1.00	0.54	0.62
1200	11400	9	9	1	0.01	2.00	0.00	0.07	1.00	0.07	0.54	1.00	0.54	0.62
1800	13200	9	9	1	0.01	2.00	0.00	0.07	1.00	0.07	0.54	1.00	0.54	0.62
1800	15000	9	9	1	0.01	2.00	0.00	0.07	1.00	0.07	0.54	1.00	0.54	0.61

DHPB – Graphical Depict of Present Species

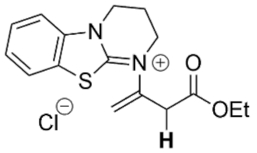

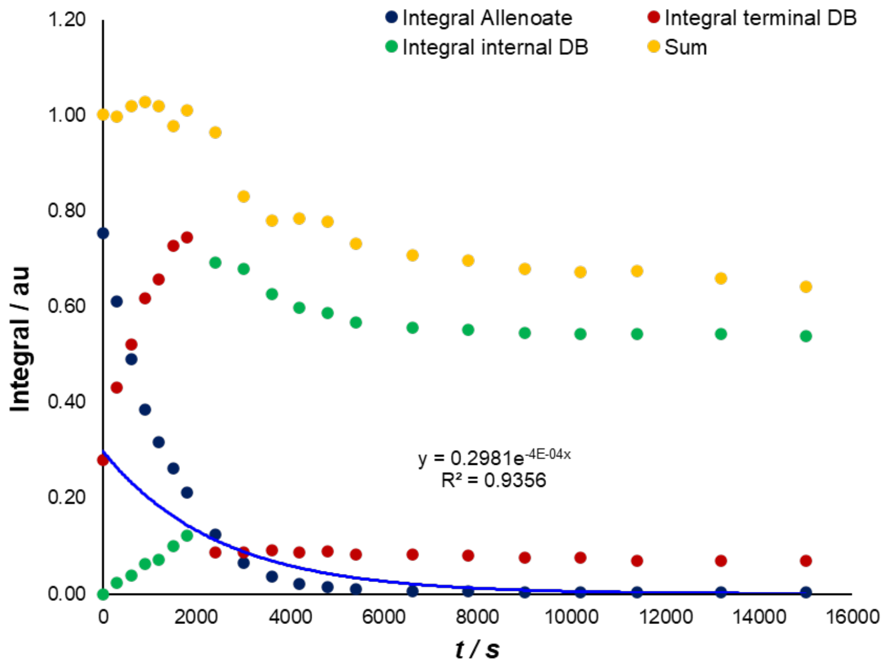

Figure S14: Decrease in allenoate concentration (blue), increase in adduct concentration (green and red) and decomposition (yellow) over the whole timespan investigated. 0 refers to the start of the first measurement. Until the start of the first measurement, roughly 5 min passed for mixing and setting up the NMR experiment (locking, shimming, tuning, and matching).

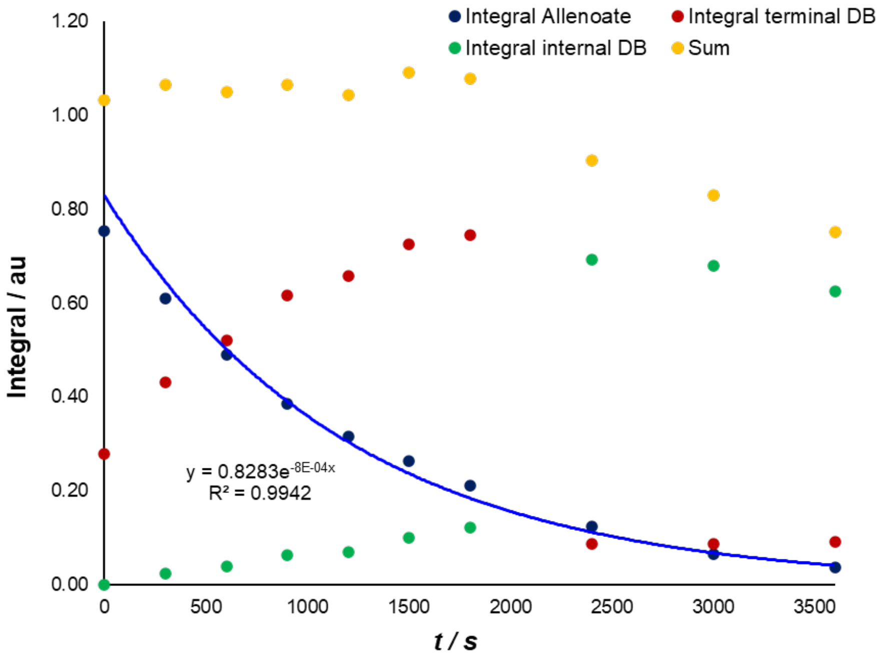

Figure S15: Decrease in allenoate concentration (blue), increase in adduct concentration (green and red) and decomposition (yellow) over the first 1 h investigated. 0 refers to the start of the first measurement. Until the start of the first measurement, roughly 5 min passed for mixing and setting up the NMR experiment (locking, shimming, tuning, and matching).

BTM – Tabulated Integrals

Table S6: Detailed evaluation of the data obtained for the trapping experiment using BTM. Mesitylene was used as internal standard. 0 refers to the start of the first measurement. Until the start of the first measurement, roughly 5 min passed for mixing and setting up the NMR experiment (locking, shimming, tuning, and matching).

time added	time total	Integral ISTD	Number protons ISTD	Calib.	Integral Allenoate	Protons Allenoate	Calib.	Integral terminal DB	Protons terminal DB	Calib.	Integral E/Z mix	Protons E/Z mix	Calib.	Sum Allenoate + Adduct
0.00	0.00	3.00	3.00	1.00	0.99	1.00	0.99	0.03	1.00	0.03	-	1.00	0.00	1.03
300.00	300.00	3.00	3.00	1.00	0.98	1.00	0.98	0.05	1.00	0.05	-	1.00	0.00	1.03
300.00	600.00	3.00	3.00	1.00	0.95	1.00	0.95	0.07	1.00	0.07	-	1.00	0.00	1.02
300.00	900.00	3.00	3.00	1.00	0.94	1.00	0.94	0.09	1.00	0.09	-	1.00	0.00	1.03
300.00	1200.00	3.00	3.00	1.00	0.92	1.00	0.92	0.11	1.00	0.11	-	1.00	0.00	1.02
300.00	1500.00	3.00	3.00	1.00	0.90	1.00	0.90	0.12	1.00	0.12	-	1.00	0.00	1.02
300.00	1800.00	3.00	3.00	1.00	0.88	1.00	0.88	0.14	1.00	0.14	-	1.00	0.00	1.02
600.00	2400.00	3.00	3.00	1.00	0.85	1.00	0.85	0.17	1.00	0.17	-	1.00	0.00	1.02
600.00	3000.00	3.00	3.00	1.00	0.82	1.00	0.82	0.20	1.00	0.20	-	1.00	0.00	1.02
600.00	3600.00	3.00	3.00	1.00	0.79	1.00	0.79	0.23	1.00	0.23	-	1.00	0.00	1.02
600.00	4200.00	3.00	3.00	1.00	0.75	1.00	0.75	0.24	1.00	0.24	-	1.00	0.00	0.99
600.00	4800.00	3.00	3.00	1.00	0.71	1.00	0.71	0.26	1.00	0.26	-	1.00	0.00	0.97
600.00	5400.00	3.00	3.00	1.00	0.71	1.00	0.71	0.30	1.00	0.30	-	1.00	0.00	1.01
1200.00	6600.00	3.00	3.00	1.00	0.67	1.00	0.67	0.35	1.00	0.35	-	1.00	0.00	1.02
1200.00	7800.00	3.00	3.00	1.00	0.65	1.00	0.65	0.41	1.00	0.41	-	1.00	0.00	1.05
1200.00	9000.00	3.00	3.00	1.00	0.62	1.00	0.62	0.45	1.00	0.45	-	1.00	0.00	1.07
1200.00	10200.00	9.00	9.00	1.00	0.56	1.00	0.56	0.46	1.00	0.46	-	1.00	0.00	1.02
1200.00	11400.00	9.00	9.00	1.00	0.50	1.00	0.50	0.53	1.00	0.53	-	1.00	0.00	1.03
1800.00	13200.00	9.00	9.00	1.00	0.45	1.00	0.45	0.54	1.00	0.54	-	1.00	0.00	0.99
1800.00	15000.00	9.00	9.00	1.00	0.40	1.00	0.40	0.57	1.00	0.57	-	1.00	0.00	0.97

BTM – Graphical Depict of Present Species

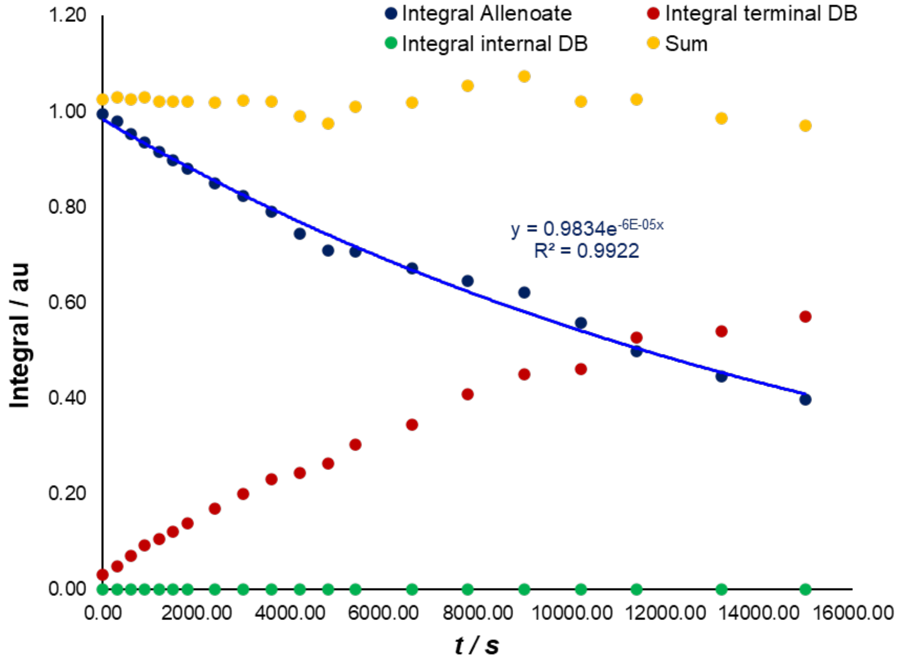

Figure S16: Decrease in allenoate concentration (blue), increase in adduct concentration (green and red) and decomposition (yellow) over the whole timespan investigated. 0 refers to the start of the first measurement. Until the start of the first measurement, roughly 5 min passed for mixing and setting up the NMR experiment (locking, shimming, tuning, and matching).

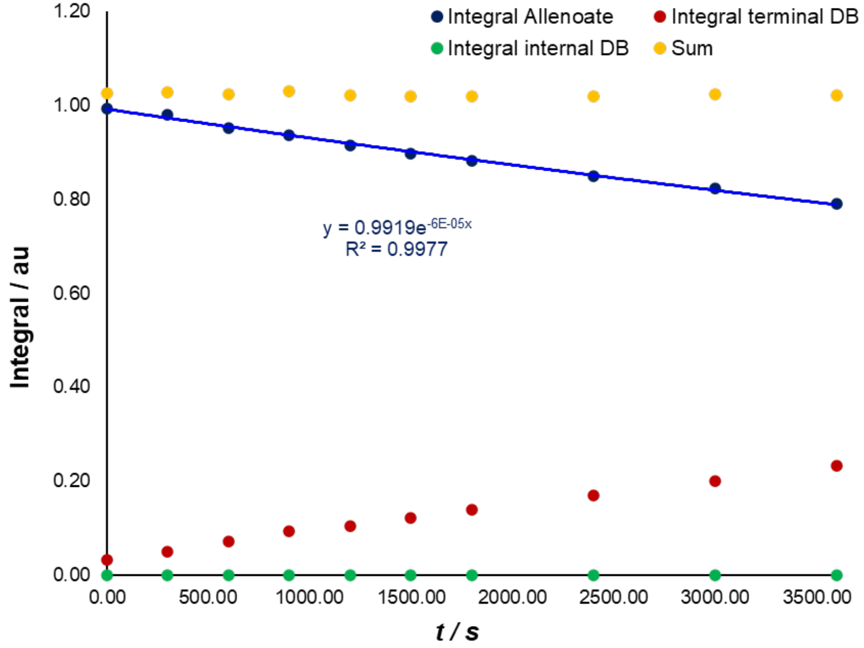

Figure S17: Decrease in allenoate concentration (blue), increase in adduct concentration (green and red) and decomposition (yellow) over the first 1 h investigated. 0 refers to the start of the first measurement. Until the start of the first measurement, roughly 5 min passed for mixing and setting up the NMR experiment (locking, shimming, tuning, and matching).

Table S7: Summarized integrals for the allenoate during the trapping experiments with TM, HBTM, Se*Hyper*BTM and O*Hyper*BTM. 0 refers to the start of the first measurement. Until the start of the first measurement, roughly 5 min passed for mixing and setting up the NMR experiment (locking, shimming, tuning, and matching).

**Mesitylen**			**TM**			**HBTM**			**Se*Hyper*BTM**			**O*Hyper*BTM**		
**Integral ISTD**	**Number protons ISTD**	**Calib.**	**Integral Allenoate**	**Protons Allenoate**	**Calib.**	**Integral Allenoate**	**Protons Allenoate**	**Calib.**	**Integral Allenoate**	**Protons Allenoate**	**Calib.**	**Integral Allenoate**	**Protons Allenoate**	**Calib.**
3.00	3.00	1.00	1.96	2.00	0.98	2.10	2.00	1.05	0.66	1.00	0.66	0.91	1.00	0.91
3.00	3.00	1.00	1.92	2.00	0.96	2.07	2.00	1.04	0.53	1.00	0.53	0.90	1.00	0.90
3.00	3.00	1.00	1.87	2.00	0.94	2.06	2.00	1.03	0.43	1.00	0.43	0.90	1.00	0.90
3.00	3.00	1.00	1.84	2.00	0.92	2.02	2.00	1.01	0.36	1.00	0.36	0.89	1.00	0.89
3.00	3.00	1.00	1.79	2.00	0.90	1.98	2.00	0.99	0.30	1.00	0.30	0.89	1.00	0.89
3.00	3.00	1.00	1.78	2.00	0.89	1.95	2.00	0.98	0.24	1.00	0.24	0.88	1.00	0.88
3.00	3.00	1.00	1.73	2.00	0.87	1.91	2.00	0.95	0.19	1.00	0.19	0.88	1.00	0.88
3.00	3.00	1.00	1.70	2.00	0.85	1.86	2.00	0.93	0.13	1.00	0.13	0.87	1.00	0.87
3.00	3.00	1.00	1.70	2.00	0.85	1.81	2.00	0.91	0.09	1.00	0.09	0.86	1.00	0.86
3.00	3.00	1.00	1.68	2.00	0.84	1.75	2.00	0.88	0.06	1.00	0.06	0.85	1.00	0.85
3.00	3.00	1.00	1.65	2.00	0.83	1.71	2.00	0.85	0.04	1.00	0.04	0.85	1.00	0.85
3.00	3.00	1.00	1.60	2.00	0.80	1.65	2.00	0.83	0.03	1.00	0.03	0.84	1.00	0.84
3.00	3.00	1.00	1.55	2.00	0.78	1.60	2.00	0.80	0.02	1.00	0.02	0.83	1.00	0.83
3.00	3.00	1.00	1.43	2.00	0.71	1.52	2.00	0.76	0.01	1.00	0.01	0.82	1.00	0.82
3.00	3.00	1.00	1.33	2.00	0.67	1.43	2.00	0.71	0.00	1.00	0.00	0.80	1.00	0.80
3.00	3.00	1.00	1.24	2.00	0.62	1.35	2.00	0.67	0.00	1.00	0.00	0.79	1.00	0.79
3.00	3.00	1.00	1.16	2.00	0.58	1.28	2.00	0.64	0.00	1.00	0.00	0.78	1.00	0.78
3.00	3.00	1.00	1.09	2.00	0.55	1.21	2.00	0.61	0.00	1.00	0.00	0.77	1.00	0.77
3.00	3.00	1.00	1.00	2.00	0.50	1.12	2.00	0.56	0.00	1.00	0.00	0.75	1.00	0.75
3.00	3.00	1.00	0.93	2.00	0.47	1.04	2.00	0.52	0.00	1.00	0.00	0.73	1.00	0.73

10. Reactivity in Acylation Reactions

10.1 NMR Monitoring of the Acylation of 1-ethynylcyclohexan-1-ol

Preparation of Stock Solutions

For each experiments the following stock solutions were prepared in by weighing solids in volumetric flasks and using $CDCl_3$ as the solvent. Liquids were added via 1 ml syringe with approximately 0.5 ml of $CDCl_3$ initially present in the volumetric flask:

1-Ehynylcyclohexan-1-ol stock: 124.2 mg in 2 ml $CDCl_3$ (0.5 mol L^{-1}); Acetic anhydride / triethylamine stock: 380 µl / 840 µl in 2 ml $CDCl_3$ (2 mol L^{-1} / 3 mol L^{-1}), respectively; Catalyst Stock: X mg in 2 ml $CDCl_3$ (0.05 mol L^{-1}) for masses see Table S8.

Table S8: Used amounts of catalysts for the NMR monitoring the acylation of 1-ethynylcyclohexan-1-ol.

Entry	Catalyst	m (mg)
1	HyperBTM	30.8
2	SeHyperBTM	35.5
3	SeHBTM	31.3
4	SeDHPB	23.7

General Procedure for NMR Monitoring of the Acylation of 1-ethynylcyclohexan-1-ol

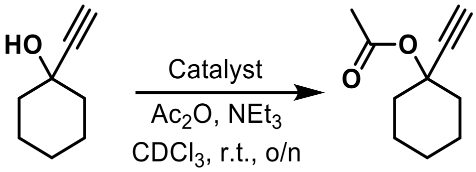

200 µl catalyst stock solution and 400 µl 1-ethynylcyclohexan-1-ol stock solution were transferred to an NMR tube. A reference spectrum was recorded. At the NMR machine, 200 µl of anhydride/base stock solution was added and a loop was initiated measuring 96 spectra with a delay between experiments of 543 s at 298 K.

HyperBTM (ITU5)

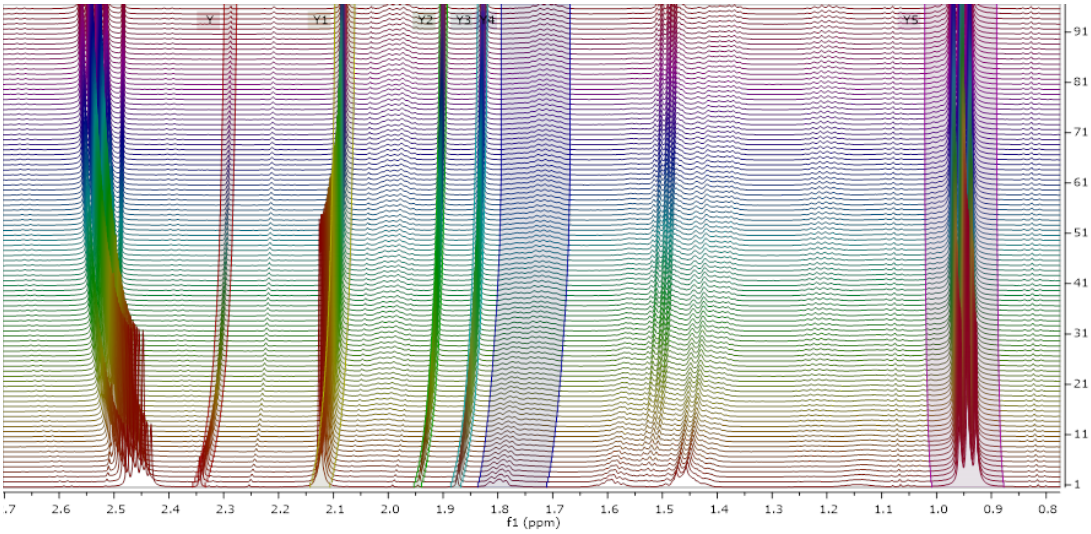

Figure S18: NMR spectra during the NMR Monitoring the Acylation of 1-ethynylcyclohexan-1-ol using HyperBTM (**ITU5**).

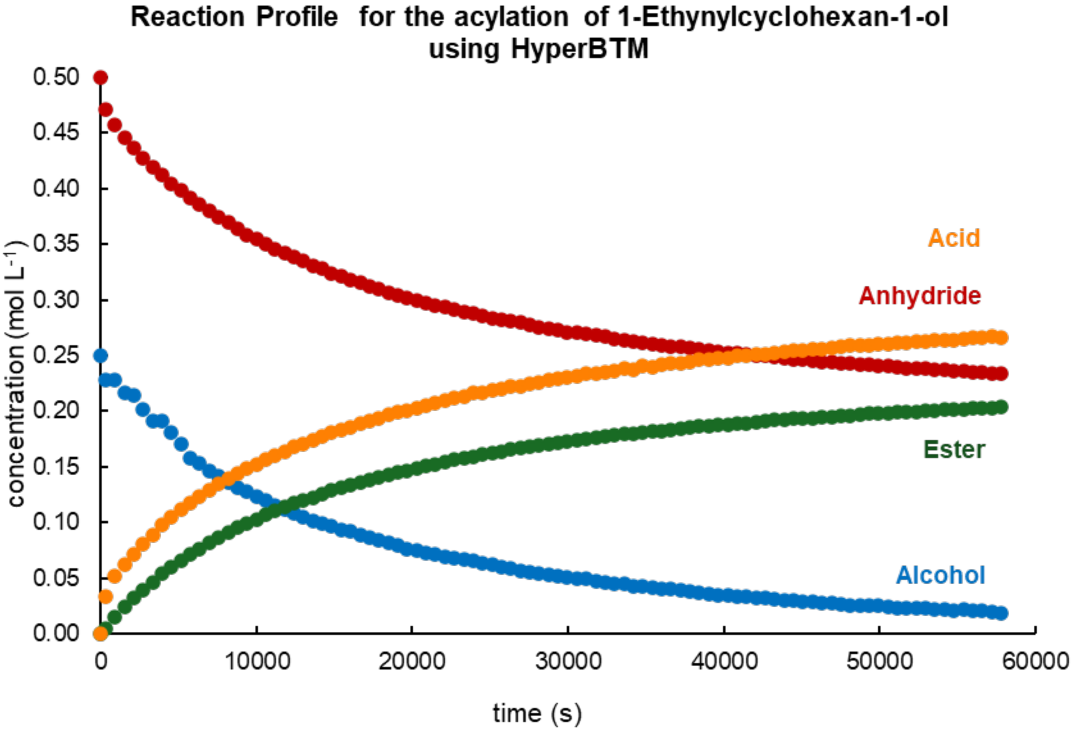

Figure S19: Reaction Profile for the acylation of 1-ethynylcyclohexan-1-ol using HyperBTM (**ITU5**).

Table S9: Raw data for the NMR Monitoring the Acylation of 1-ethynylcyclohexan-1-ol using HyperBTM (**ITU5**).

Time (s)	Integral(2.357,2.334)	c (alc, alkyne) (mol L^{-1})	Integral(2.142,2.106)	c (anhydride) (mol L^{-1})	Integral(1.953,1.939)	c (ester Me) (mol L^{-1})	Integral(1.886,1.867)	c (carboxylic acid Me) (mol L^{-1})	Integral(1.837,1.722)	c (alc+ester) (mol L^{-1})	Integral(1.009,0.877)	c (Me, NEt3) (mol L^{-1})
0		0.25		0.5		0		0		0.25		0.75333531
326	14503.72	0.228655192	179356.817	0.471268376	978.297	0.005141042	6435.986	0.033821705	32231.812	0.25	430060.121	0.75333531
931	14568.911	0.228399454	174762.874	0.456631533	2920.883	0.015263737	9874.866	0.051603354	33356.058	0.25	432476.847	0.75333531
1536	14196.771	0.216491095	175357.739	0.445681028	4732.642	0.024056523	12307.492	0.062560292	34300.362	0.25	444611.174	0.75333531
2140	13991.98	0.213884787	171196.995	0.436159771	6257.173	0.031882886	14118.151	0.071937822	33301.148	0.25	443537.265	0.75333531
2745	13420.293	0.201676614	170580.788	0.427240496	7922.572	0.039686106	16221.877	0.081259359	34430.511	0.25	451166.961	0.75333531
3350	12605.787	0.19175121	165330.221	0.419149685	9178.069	0.046536982	17559.607	0.089035189	33317.789	0.25	445720.578	0.75333531
3955	12450.388	0.191831253	160544.87	0.41226993	10527.289	0.054066937	19002.245	0.09759333	34607.644	0.25	440042.229	0.75333531
4559	11701.101	0.181016366	157008.871	0.404821953	11700.46	0.060335483	20348.767	0.104932002	34374.96	0.25	438267.956	0.75333531
5164	10921.422	0.170267445	153253.618	0.398209775	12745.985	0.066237599	21459.05	0.111517151	34068.35	0.25	434888.98	0.75333531
5768	10144.168	0.158174405	150503.881	0.391125584	13726.744	0.071345413	22601.492	0.117472343	33209.58	0.25	434821.548	0.75333531
6373	9793.834	0.153481065	147533.841	0.385338554	14679.942	0.076684069	23544.434	0.122989791	33184.577	0.25	432642.091	0.75333531
6978	9229.494	0.145831409	144127.219	0.379549073	15502.05	0.081647155	24476.19	0.128912711	32796.976	0.25	429099.15	0.75333531
7583	8822.886	0.141346566	140189.17	0.374315498	16218.785	0.086610722	25178.791	0.134458485	32532.935	0.25	423210.309	0.75333531
8187	8671.945	0.135705407	141551.116	0.369183842	17435.019	0.090945624	26681.126	0.139175739	33174.542	0.25	433261.596	0.75333531
8792	9047.919	0.131142956	150477.671	0.363510597	19733.941	0.095343005	29713.405	0.143558011	35787.714	0.25	467772.372	0.75333531
9396	9146.422	0.127619201	154154.582	0.358484164	21374.58	0.099412529	31918.753	0.148453161	37202.098	0.25	485921.422	0.75333531
10000	8318.462	0.123846659	142819.72	0.354387327	20780.429	0.103127505	30599.621	0.151857431	35043.808	0.25	455396.383	0.75333531
10605	8461.962	0.119996683	147997.386	0.349784824	22643.291	0.107032695	33104.061	0.156479766	36657.927	0.25	478115.325	0.75333531
11209	8685.422	0.115826807	155416.146	0.345432379	24820.431	0.110333202	35981.104	0.159945265	39031.571	0.25	508408.35	0.75333531
11814	8034.396	0.111650826	147531.958	0.341698886	24554.343	0.113740667	35342.964	0.163715734	37248.174	0.25	487890.236	0.75333531
12418	7504.887	0.108587645	140256.862	0.338227664	24276.904	0.117086899	34818.966	0.167931	35878.341	0.25	468591.682	0.75333531
13023	7197.534	0.104835101	137869.538	0.334687938	24659.195	0.119723838	35024.447	0.170048585	35496.552	0.25	465487.306	0.75333531
13628	6888.413	0.101912861	134248.017	0.331029306	24868.027	0.122639364	35325.411	0.174211084	34949.516	0.25	458269.569	0.75333531
14232	6686.704	0.098607424	133378.371	0.327817153	25592.698	0.125803387	36058.361	0.177248367	34968.216	0.25	459762.258	0.75333531
14837	7080.886	0.096574863	142631.701	0.324221126	28296.458	0.128643344	39827.437	0.18106629	38035.111	0.25	497112.101	0.75333531
15441	7129.166	0.093540284	146746.637	0.320905282	30023.167	0.131309215	42012.574	0.183746043	39440.572	0.25	516738.565	0.75333531
16046	7167.691	0.09170419	149076.777	0.317883984	31324.958	0.133591598	43488.062	0.185463607	40609.184	0.25	529932.958	0.75333531

16651	6930.864	0.088866919	147466.519	0.315133755	31869.603	0.136209734	44277.922	0.18924252	40401.525	0.25	528783.734	0.75333531
17255	6387.126	0.086422881	138603.546	0.312569318	30720.476	0.138557469	42480.888	0.191600036	38363.833	0.25	501080.588	0.75333531
17860	6526.437	0.083944283	144323.799	0.309386724	32772.53	0.140508853	45143.27	0.193547128	40226.039	0.25	527127.727	0.75333531
18465	6483.447	0.081593858	146282.316	0.306825946	34094.805	0.143027142	47045.198	0.197353826	41091.671	0.25	538740.132	0.75333531
19069	6047.408	0.079129973	139611.446	0.304467908	33227.083	0.1449248	45769.519	0.199630476	39356.888	0.25	518154.275	0.75333531
19674	5956.216	0.076552105	140990.684	0.30201316	34297.76	0.146937012	46840.142	0.200670554	40035.909	0.25	527526.32	0.75333531
20279	6045.725	0.07489824	145009.982	0.299412462	36022.177	0.148755121	49074.981	0.202657233	41519.239	0.25	547277.52	0.75333531
20883	6013.766	0.07322568	146320.951	0.296942359	37105.069	0.150601355	50559.881	0.205211492	42292.253	0.25	556818.868	0.75333531
21488	5550.508	0.071258674	138008.514	0.295297408	35644.194	0.152536069	48376.615	0.2070233	40157.762	0.25	528111.747	0.75333531
22092	5168.408	0.069590904	130844.339	0.293629291	34403.117	0.154408864	46658.499	0.209413752	38351.097	0.25	503541.355	0.75333531
22697	4904.504	0.067904117	126427.29	0.291736381	33858.93	0.156261859	45893.263	0.211801336	37235.834	0.25	489699.681	0.75333531
23302	5063.85	0.06667898	131914.179	0.289499849	35983.695	0.157940175	48637.225	0.213479239	39264.621	0.25	514899.797	0.75333531
23906	5153.887	0.065366644	136129.609	0.287754844	37734.256	0.159527601	51137.118	0.216190343	40794.719	0.25	534576.098	0.75333531
24511	5118.51	0.063658357	137761.653	0.285554469	38817.592	0.160923401	52302.224	0.216825705	41599.002	0.25	545153.704	0.75333531
25116	5046.477	0.061998747	138578.18	0.283751534	39663.171	0.162427961	53406.227	0.218708297	42081.805	0.25	551869.278	0.75333531
25720	4584.831	0.05995225	129570.833	0.282382742	37610.064	0.163932465	50535.28	0.220270112	39491.807	0.25	518499.906	0.75333531
26325	4220.26	0.058664013	121298.27	0.281019151	35719.406	0.165506683	47937.782	0.222120807	37134.797	0.25	487751.116	0.75333531
26930	3980.972	0.056275283	118608.775	0.279443579	35369.274	0.166660797	47276.154	0.222766278	35829.175	0.25	479625.504	0.75333531
27534	4104.715	0.055511673	123137.026	0.277548394	37290.664	0.168104822	49914.652	0.225013255	38036.367	0.25	501336.73	0.75333531
28139	4137.443	0.053953008	126855.698	0.275703556	38899.862	0.169087088	52021.069	0.226121395	39353.488	0.25	519932.774	0.75333531
28743	4094.02	0.052904809	127301.744	0.274175277	39542.29	0.170327883	52902.953	0.22787876	39824.063	0.25	524669.291	0.75333531
29348	4036.194	0.051787957	127566.198	0.272797955	40093.823	0.171479798	53589.698	0.229201156	39982.983	0.25	528413.719	0.75333531
29953	4051.303	0.050647742	130126.731	0.271131912	41417.968	0.172596864	55224.984	0.23013343	41173.508	0.25	542332.295	0.75333531
30557	3858.265	0.049798238	125590.546	0.270164091	40418.695	0.173893344	53907.882	0.231927871	39991.543	0.25	525301.822	0.75333531
31162	3559.723	0.049247965	116817.658	0.269357661	37982.142	0.175158125	50591.261	0.233306231	37399.709	0.25	490070.708	0.75333531
31766	3325.604	0.047733232	112055.18	0.268059373	36805.815	0.17609438	48894.469	0.233931546	35918.613	0.25	472368.058	0.75333531
32371	3196.048	0.04610092	111077.544	0.267036922	36867.812	0.177264759	48817.932	0.234722336	35626.911	0.25	470039.697	0.75333531
32976	3271.885	0.045382553	114779.038	0.265339693	38559.253	0.178278204	51101.538	0.236267295	37155.629	0.25	488809.841	0.75333531
33580	3329.796	0.044701246	117965.414	0.263940144	40046.39	0.179202524	53278.469	0.238414402	38441.817	0.25	505043.55	0.75333531
34185	3281.535	0.043332185	119517.182	0.263034457	40898.705	0.180020453	54006.755	0.237717075	38610.86	0.25	513448.967	0.75333531
34790	3275.607	0.043185035	119102.196	0.261703785	41178.867	0.180965015	54674.478	0.240272947	39249.133	0.25	514267.819	0.75333531

35395	3229.453	0.041885336	120485.667	0.260445685	42037.258	0.181738172	55466.988	0.239798443	39862.018	0.25	522754.527	0.75333531
35999	3189.149	0.040968688	121146.451	0.259380122	42630.534	0.182547867	56439.563	0.241679399	40167.019	0.25	527780.801	0.75333531
36604	3091.456	0.040052545	119647.649	0.258356844	42502.789	0.183553735	56191.295	0.2426693	39860.516	0.25	523315.722	0.75333531
37209	2788.22	0.039367164	109562.156	0.257819886	39195.665	0.184469205	51691.894	0.243281051	36617.286	0.25	480201.752	0.75333531
37813	2658.804	0.038178122	107357.622	0.256927072	38767.404	0.185555444	50990.978	0.244062088	35925.021	0.25	472174.575	0.75333531
38418	2668.909	0.037075212	110459.757	0.255742385	40207.457	0.186180945	53170.524	0.246206528	36945.177	0.25	488068.698	0.75333531
39023	2634.055	0.035580595	113127.118	0.254685277	41504.611	0.186880273	54784.165	0.246673309	37658.05	0.25	501929.205	0.75333531
39627	2647.199	0.034971522	115155.833	0.253549428	42558.728	0.187411108	56296.971	0.247908672	38869.012	0.25	513219.192	0.75333531
40232	2651.37	0.034654645	115825.817	0.252316257	43184.098	0.188145446	56883.341	0.247830615	39409.185	0.25	518728.036	0.75333531
40837	2616.213	0.033852123	116776.131	0.251834739	43792.881	0.188883955	57977.173	0.25006251	39392.781	0.25	523984.002	0.75333531
41441	2642.213	0.033951437	117008.556	0.250585942	44240.862	0.189492776	58394.629	0.250116292	40259.338	0.25	527643.388	0.75333531
42046	2393.496	0.032975215	109065.529	0.25043279	41464.961	0.190421042	54589.703	0.25069427	37437.668	0.25	492125.537	0.75333531
42651	2211.305	0.03209171	103285.186	0.249822182	39533.478	0.191244071	51922.395	0.25117573	35570.491	0.25	467182.56	0.75333531
43255	2117.71	0.031193886	101480.582	0.249134973	39113.002	0.192044951	51473.869	0.252736843	34641.351	0.25	460286.073	0.75333531
43860	2168.227	0.030665965	105219.57	0.24802597	40788.623	0.192295745	53862.669	0.253932624	36391.36	0.25	479378.937	0.75333531
44464	2160.499	0.029864668	107213.884	0.247003667	41923.305	0.1931692	55169.245	0.254202261	37128.05	0.25	490486.672	0.75333531
45069	2122.696	0.028986181	108209.247	0.24627273	42513.262	0.19351132	55997.925	0.254890635	37568.605	0.25	496509.581	0.75333531
45674	2126.594	0.028550561	109693.774	0.245448728	43332.656	0.193920674	57071.762	0.255405405	38264.6	0.25	505010.928	0.75333531
46278	2085.491	0.027748419	110279.266	0.24455274	43824.668	0.194369133	57774.407	0.256238367	38584.203	0.25	509566.556	0.75333531
46883	2095.542	0.027663951	110868.914	0.243936275	44306.093	0.194966522	58432.854	0.257130556	39016.146	0.25	513585.778	0.75333531
47487	2060.48	0.02688736	111763.816	0.24306909	44929.921	0.19543132	59240.169	0.257676491	39403.371	0.25	519578.377	0.75333531
48092	1792.512	0.025317302	103256.85	0.243065307	41688.898	0.196270268	55065.136	0.259245255	35851.919	0.25	480037.847	0.75333531
48696	1721.896	0.025369541	98657.488	0.242261551	40094.338	0.196909869	52722.33	0.258928008	34766.433	0.25	460177.249	0.75333531
49301	1695.948	0.025341478	97178.997	0.242014049	39685.284	0.197664034	52127.318	0.259635182	34345.051	0.25	453744.546	0.75333531
49905	1724.761	0.025049688	99682.479	0.241290923	40931.675	0.198158026	53716.384	0.260051234	35048.396	0.25	466828.572	0.75333531
50510	1713.631	0.024361931	101390.065	0.240236257	41875.54	0.198441987	55019.335	0.260728487	36157.559	0.25	476910.003	0.75333531
51114	1687.714	0.023635248	102674.776	0.239648007	42593.207	0.198829305	56016.865	0.261492268	36592.888	0.25	484138.395	0.75333531
51719	1692.049	0.023453954	103472.083	0.239042472	43112.15	0.199196433	56491.139	0.261013041	36937.28	0.25	489133.832	0.75333531
52323	1660.282	0.022852247	103959.341	0.238484037	43473.08	0.199455586	57256.364	0.26269364	37310.944	0.25	492587.952	0.75333531
52928	1672.618	0.022881377	104438.145	0.238118581	43869.423	0.200044241	57572.846	0.262531748	37272.857	0.25	495616.147	0.75333531
53533	1532.929	0.022234231	98241.299	0.237488679	41462.337	0.200462245	54503.911	0.26351569	35569.604	0.25	467445.267	0.75333531

54137	1445.638	0.021830849	94219.823	0.237138523	39945.408	0.201074354	52491.098	0.264225956	34103.248	0.25	448972.517	0.75333531
54742	1356.116	0.020665964	93177.622	0.236656919	39678.603	0.201555175	52038.455	0.264339445	33664.885	0.25	444909.827	0.75333531
55346	1478.134	0.021636824	96689.387	0.235888765	41410.087	0.202052667	54211.556	0.264515008	35303.471	0.25	463181.424	0.75333531
55951	1480.295	0.020999149	99557.476	0.235383519	42771.047	0.202246982	56156.443	0.265541106	36332.595	0.25	477944.436	0.75333531
56555	1469.867	0.020664863	100094.711	0.234538628	43233.783	0.202607951	56838.345	0.266363474	36711.281	0.25	482254.548	0.75333531
57160	1397.922	0.01977476	99318.073	0.234156016	43006.425	0.202787123	56573.188	0.266758142	36429.336	0.25	479294.613	0.75333531
57765	1273.961	0.019150024	93425.694	0.234060576	40595.195	0.203407314	53198.832	0.266559417	34121.751	0.25	451042.687	0.75333531

SeHyperBTM (ISe5)

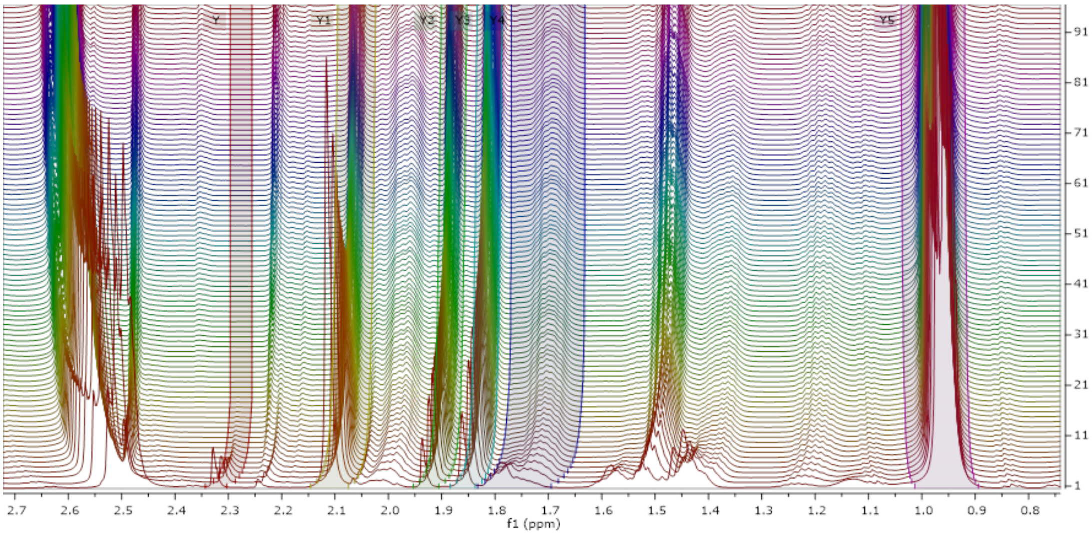

Figure S20: NMR spectra during the NMR Monitoring the Acylation of 1-ethynylcyclohexan-1-ol using SeHyperBTM (**ISeU5**).

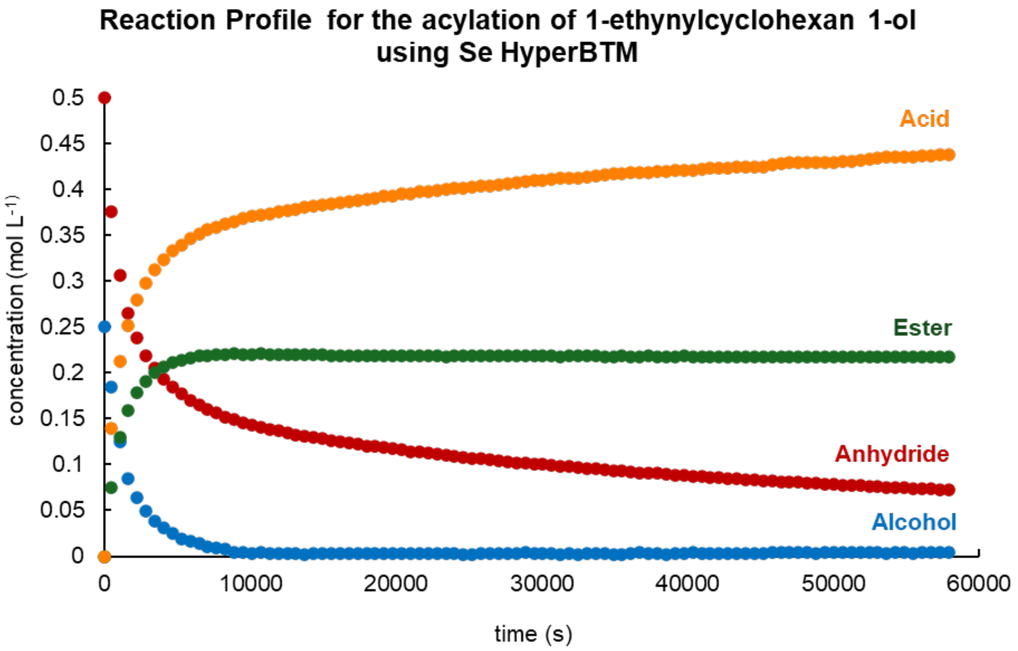

Figure S21: Reaction Profile for the acylation of 1-ethynylcyclohexan-1-ol using SeHyperBTM (**ISeU5**).

Table S10: Raw data for the NMR Monitoring the Acylation of 1-ethynylcyclohexan-1-ol using SeHyperBTM (**ISeU5**).

Time (s)	Integral(2.343,2.302)	c (alc, alkyne) (mol L^{-1})	Integral(2.146,2.075)	c (anhydride) (mol L^{-1})	Integral(1.953,1.905)	c (ester Me) (mol L^{-1})	Integral(1.884,1.837)	c (carboxylic acid Me) (mol L^{-1})	Integral(1.824,1.657)	c (alc+ester) (mol L^{-1})	Integral(1.013,0.894)	c (Me, NEt3) (mol L^{-1})
0		0.25		0.5		0		0		0.25		0.75333531
431	11024.951	0.184832062	134630.786	0.376178328	13413.134	0.07495656	24989.745	0.139650087	33912.093	0.25	404417.735	0.75333531
1037	7832.618	0.125395128	114645.275	0.305899401	24412.023	0.130273545	39779.943	0.212283685	35765.342	0.25	423503.61	0.75333531
1642	5373.718	0.084505274	101003.731	0.264725093	30382.035	0.159259207	48035.252	0.251795383	35251.372	0.25	431143.546	0.75333531
2246	4105.216	0.064106701	91454.772	0.23802498	34248.529	0.178274031	53587.542	0.278939488	35446.444	0.25	434173.604	0.75333531
2850	3164.08	0.049205223	84522.831	0.219071827	36830.906	0.190921524	57536.063	0.298251497	35448.775	0.25	435980.523	0.75333531
3455	2555.13	0.03875183	80991.046	0.204722215	39552.539	0.199955027	61824.28	0.312548217	36252.896	0.25	447045.389	0.75333531
4060	2033.503	0.030980616	76169.752	0.193408932	40625.35	0.206310388	63722.704	0.323607201	36024.027	0.25	445026.218	0.75333531
4664	1666.557	0.024807638	74304.753	0.184344674	42472.881	0.210744242	67058.099	0.332732508	36829.863	0.25	455476.089	0.75333531
5269	1257.333	0.018597897	71701.857	0.176763531	43392.336	0.213946551	68840.442	0.339418813	36761.524	0.25	458371.196	0.75333531
5873	1114.95	0.016479843	69167.282	0.170391186	43885.37	0.216220156	70215.088	0.345944839	37028.904	0.25	458704.676	0.75333531
6478	914.642	0.013421802	67413.214	0.164874486	44591.598	0.218117973	71718.65	0.350808835	37293.367	0.25	462031.049	0.75333531
7082	745.672	0.010961619	65353.957	0.160120704	44671.531	0.2188953	72574.954	0.35562507	37254.656	0.25	461215.59	0.75333531
7687	662.893	0.009548216	64945.816	0.155911715	45783.832	0.219821266	74817.415	0.359219799	38010.602	0.25	470708.469	0.75333531
8292	537.319	0.00787635	62311.171	0.15223253	45085.685	0.220297831	74265.634	0.362876999	37341.611	0.25	462528.002	0.75333531
8897	335.731	0.004886318	61390.083	0.148914583	45457.624	0.220534092	75256.657	0.36510176	37210.787	0.25	465844.073	0.75333531
9501	292.611	0.004293393	59707.167	0.146010873	45060.815	0.220387912	75248.837	0.36803449	36891.996	0.25	462083.914	0.75333531
10106	241.514	0.003567456	58192.499	0.143262333	44768.136	0.220426608	75223.13	0.370379044	36639.619	0.25	459001.994	0.75333531
10711	269.65	0.004013649	56850.511	0.141033419	44476.229	0.220671179	75054.926	0.372389012	36387.468	0.25	455503.713	0.75333531
11315	192.069	0.002689905	59289.534	0.138390565	47129.147	0.220012836	80087.867	0.373873916	38735.522	0.25	484117.898	0.75333531
11920	230.69	0.003099714	60962.121	0.136521715	49129.664	0.220047003	83849.962	0.375555852	40337.108	0.25	504589.158	0.75333531
12525	231.083	0.003030802	61489.199	0.134411711	50248.921	0.219682271	86206.663	0.376885217	41465.256	0.25	516941.394	0.75333531
13129	220.471	0.002869161	61125.259	0.132578433	50574.4	0.219388017	87239.429	0.378438209	41772.357	0.25	520987.633	0.75333531
13733	137.544	0.001826767	59164.496	0.130963883	49540.206	0.219319969	85926.465	0.380405961	40621.356	0.25	510492.317	0.75333531
14338	209.609	0.002811786	57930.423	0.129517308	49085.763	0.219485913	85400.806	0.381867831	40307.659	0.25	505427.041	0.75333531
14943	212.229	0.002749445	59265.083	0.127964061	50768.213	0.219235556	88702.245	0.383048463	41723.243	0.25	523347.876	0.75333531
15548	246.895	0.003142361	59626.669	0.12648328	51660.308	0.219169217	90480.512	0.383864203	42476.518	0.25	532705.295	0.75333531
16152	208.282	0.002766621	56272.469	0.124578389	49365.844	0.218576417	87111.827	0.385703748	40873.797	0.25	510426.063	0.75333531

16757	214.442	0.002751984	57629.502	0.123262126	51160.031	0.218849512	90376.661	0.386608213	42297.261	0.25	528317.255	0.75333531
17361	206.506	0.002615571	57734.78	0.12187656	51750.894	0.218489476	91857.829	0.387818787	42896.55	0.25	535299.591	0.75333531
17965	238.116	0.003002915	57361.28	0.120565226	51979.435	0.218506711	92562.68	0.389107091	43064.925	0.25	537621.16	0.75333531
18570	230.591	0.003038681	54489.594	0.119675445	49845.633	0.218951835	88883.699	0.390430372	40834.625	0.25	514503.228	0.75333531
19174	225.474	0.003031245	52843.647	0.118403921	48822.121	0.218786208	87517.463	0.392191356	40007.872	0.25	504320.104	0.75333531
19779	223.867	0.00306529	51357.042	0.117200714	47960.143	0.218897459	86167.142	0.393280071	39308.396	0.25	495164.302	0.75333531
20384	223.65	0.003114401	50014.689	0.116078557	47145.58	0.218839346	85008.262	0.394589534	38570.472	0.25	486883.609	0.75333531
20988	205.795	0.002885375	48941.11	0.114364171	46697.078	0.218240765	84671.624	0.395716408	38584.979	0.25	483574.521	0.75333531
21593	221.317	0.003174787	47381.767	0.113281719	45673.58	0.218395471	83048.274	0.397108501	37743.592	0.25	472640.578	0.75333531
22198	206.175	0.002932832	47286.401	0.112107877	46037.857	0.218295591	83937.484	0.398002511	38035.668	0.25	476628.178	0.75333531
22802	214.42	0.002939066	48641.932	0.111122895	47748.768	0.218164908	87383.124	0.399254934	39450.879	0.25	494637.289	0.75333531
23407	270.733	0.00361735	49448.102	0.110115309	48937.73	0.217957538	89928.936	0.400523062	40650.174	0.25	507436.27	0.75333531
24012	232.206	0.003059365	49739.98	0.109222533	49739.834	0.218444425	91275.586	0.400858654	40897.381	0.25	514603.747	0.75333531
24616	138.087	0.001789534	50035.25	0.108071696	50560.405	0.218411969	92993.64	0.401716007	41365.977	0.25	523171.032	0.75333531
25221	162.654	0.00207128	50427.371	0.107025957	51437.646	0.218340286	94711.84	0.402028705	42127.235	0.25	532422.977	0.75333531
25825	229.621	0.003090399	47259.887	0.106009399	48656.264	0.218283268	90017.154	0.403837799	40090.272	0.25	503764.884	0.75333531
26430	260.819	0.003391352	48644.226	0.105417745	50483.721	0.218808294	93262.732	0.404222566	41249.077	0.25	521431.372	0.75333531
27034	265.882	0.003378479	49302.05	0.104410955	51601.733	0.21856236	95637.102	0.405076914	42292.134	0.25	533578.711	0.75333531
27639	269.583	0.003370184	49650.695	0.103451129	52368.552	0.218227996	97424.025	0.405981241	43097.448	0.25	542337.556	0.75333531
28244	247.147	0.003203833	47335.645	0.102270768	50476.187	0.218112098	94254.334	0.407281369	41643.333	0.25	523017.672	0.75333531
28848	273.108	0.003684557	45098.652	0.101405892	48492.018	0.218071989	90849.342	0.40855583	40128.575	0.25	502550.781	0.75333531
29453	254.553	0.003496349	43924.215	0.100551681	47664.63	0.218228541	89378.373	0.409211442	39326.071	0.25	493621.713	0.75333531
30057	226.885	0.003170901	42898.187	0.099922795	46892.989	0.218455784	88077.621	0.410318603	38333.934	0.25	485125.325	0.75333531
30662	236.524	0.0033184	42375.54	0.099087179	46691.308	0.218357571	87951.964	0.411318039	38208.332	0.25	483256.121	0.75333531
31267	155.831	0.002091674	43831.258	0.098055714	48714.384	0.217959691	92017.986	0.411710262	39833.101	0.25	505115.401	0.75333531
31872	265.483	0.003482271	44609.113	0.097521002	49979.325	0.218521892	94286.491	0.41224371	40827.809	0.25	516898.193	0.75333531
32477	174.499	0.002249152	44873.637	0.096397523	50811.92	0.218308279	96038.778	0.412620903	41541.914	0.25	526023.297	0.75333531
33081	273.842	0.003504675	44997.753	0.095981447	51206.391	0.218449286	97005.672	0.413831543	41879.051	0.25	529764.823	0.75333531
33686	268.021	0.003385308	45160.626	0.095068819	51873.979	0.218402549	98377.113	0.414192485	42524.017	0.25	536786.318	0.75333531
34291	240.655	0.003215412	42280.72	0.094152721	48940.306	0.217965208	93413.605	0.416035728	40292.223	0.25	507445.123	0.75333531
34895	142.053	0.001967425	40461.166	0.093397416	47186.531	0.217843452	90299.047	0.416878625	38621.68	0.25	489534.29	0.75333531

35500	119.396	0.001691512	39244.429	0.092664221	46182.016	0.2180906	88362.905	0.417286221	37624.881	0.25	478570.054	0.75333531
36104	232.665	0.003302785	38881.733	0.09199064	46037.379	0.217840493	88467.879	0.418613892	37963.481	0.25	477618.959	0.75333531
36709	274.488	0.003710082	40497.28	0.091229392	48331.023	0.217753383	92903.59	0.418573201	40014.293	0.25	501615.162	0.75333531
37313	272.356	0.003590058	41335.398	0.090810354	49646.486	0.218138214	95342.208	0.418917444	40634.334	0.25	514358.995	0.75333531
37918	276.06	0.003599954	41476.926	0.090146485	50131.95	0.21791485	96573.026	0.419786114	41145.873	0.25	519920.988	0.75333531
38522	182.057	0.002346448	41513.086	0.089173822	50707.213	0.217847257	97672.672	0.41961927	41549.122	0.25	526050.24	0.75333531
39127	263.229	0.003378762	41379.747	0.088523869	50847.711	0.21755745	98359.814	0.420843139	42054.969	0.25	528210.495	0.75333531
39732	289.016	0.003673114	41487.407	0.087877482	51463.419	0.218016792	99228.249	0.420365085	42357.836	0.25	533480.156	0.75333531
40336	293.759	0.003711606	41412.798	0.08720754	51664.45	0.217591171	99967.899	0.421027073	42702.353	0.25	536611.679	0.75333531
40941	277.107	0.003456914	41668.604	0.086636074	52348.291	0.217681898	101384.039	0.421589121	43181.465	0.25	543487.765	0.75333531
41545	269.709	0.00353353	39429.554	0.086096205	49862.03	0.21775197	96869.9	0.423039567	41115.354	0.25	517508.445	0.75333531
42150	269.815	0.003449215	40052.725	0.085336538	51060.917	0.217581295	99305.165	0.423160172	42156.679	0.25	530367.167	0.75333531
42755	274.595	0.003443858	40526.925	0.084712005	52020.101	0.217471572	101214.058	0.423128366	43016.622	0.25	540602.781	0.75333531
43359	267.51	0.003333919	40500.762	0.084125371	52328.813	0.217387555	102063.223	0.423997282	43299.14	0.25	544021.15	0.75333531
43964	283.146	0.003487466	40700.701	0.08355072	52935.929	0.217334584	103240.373	0.423865303	43861.069	0.25	550466.985	0.75333531
44568	271.009	0.003323956	40584.826	0.082962914	53181.725	0.217426625	103798.765	0.424367866	44031.039	0.25	552788.849	0.75333531
45173	261.554	0.003203907	40358.628	0.082395538	53162.485	0.217071381	104091.82	0.425024434	44176.353	0.25	553493.192	0.75333531
45777	362.93	0.004631196	38585.425	0.082062038	51129.972	0.217482621	100335.235	0.426778445	42456.436	0.25	531325.396	0.75333531
46382	333.74	0.004448183	36675.66	0.081470633	48954.477	0.217493141	96348.781	0.428054803	40570.277	0.25	508693.781	0.75333531
46987	313.957	0.004328197	35209.936	0.080900424	47266.845	0.217206177	93433.169	0.429355111	39246.304	0.25	491806.224	0.75333531
47591	285.621	0.003796613	36355.041	0.08054147	49064.852	0.217397929	96803.42	0.428919322	40350.081	0.25	510063.996	0.75333531
48196	290.725	0.003761975	37086.996	0.079984152	50456.265	0.21763432	99478.122	0.429081571	41343.975	0.25	523958.988	0.75333531
48800	225.939	0.002884108	37285.582	0.079324995	51126.755	0.217544122	100864.004	0.429175902	41762.049	0.25	531141.767	0.75333531
49405	291.46	0.003693063	37400.671	0.078983422	51522.273	0.217611358	101716.102	0.429611852	42405.569	0.25	535085.316	0.75333531
50009	306.953	0.003862184	37360.782	0.078347613	51933.797	0.217816051	102386.351	0.429419607	42731.621	0.25	538852.341	0.75333531
50614	325.91	0.004085081	37259.489	0.077837454	52092.852	0.217650596	103002.934	0.430359427	42892.193	0.25	540913.541	0.75333531
51219	335.684	0.004161144	37444.283	0.077359993	52666.363	0.217617705	104230.675	0.43068173	43571.644	0.25	546951.327	0.75333531
51823	302.336	0.003739958	37256.036	0.076810792	52708.964	0.217340206	104581.47	0.431231361	43493.738	0.25	548092.657	0.75333531
52428	285.768	0.003763863	34731.117	0.07624084	49427.28	0.217002944	98734.213	0.433477523	40986.185	0.25	514766.96	0.75333531
53032	279.394	0.003850148	33129.269	0.07608883	47365.328	0.217570293	94535.15	0.434242539	38762.262	0.25	492006.15	0.75333531
53636	192.764	0.002726953	32017.379	0.075489441	46101.458	0.217392768	92232.669	0.434925836	37599.149	0.25	479268.789	0.75333531

54241	283.792	0.003879666	33061.446	0.075329442	47697.026	0.217352281	95578.188	0.435543658	39062.028	0.25	495948.61	0.75333531
54845	305.669	0.004038069	34019.467	0.074902899	49453.013	0.217767907	98860.82	0.435336747	40420.422	0.25	513225.775	0.75333531
55450	198.159	0.002572002	34335.905	0.074277074	50306.083	0.217649056	100655.722	0.435486557	40777.322	0.25	522364.066	0.75333531
56054	323.686	0.00413919	34746.123	0.074053672	51062.408	0.217656445	102308.894	0.436097534	41725.063	0.25	530199.529	0.75333531
56659	296.619	0.003867611	33788.274	0.073427466	50021.052	0.217407914	100484.014	0.436736514	41007.031	0.25	519980.492	0.75333531
57263	301.084	0.004037353	32652.876	0.07297587	48601.546	0.217239065	97989.835	0.437994712	39820.393	0.25	505617.082	0.75333531
57868	319.344	0.004138168	33655.125	0.072685761	50334.257	0.217416144	101417.44	0.438067235	41167.784	0.25	523216.524	0.75333531

SeHBTM (ISeU4)

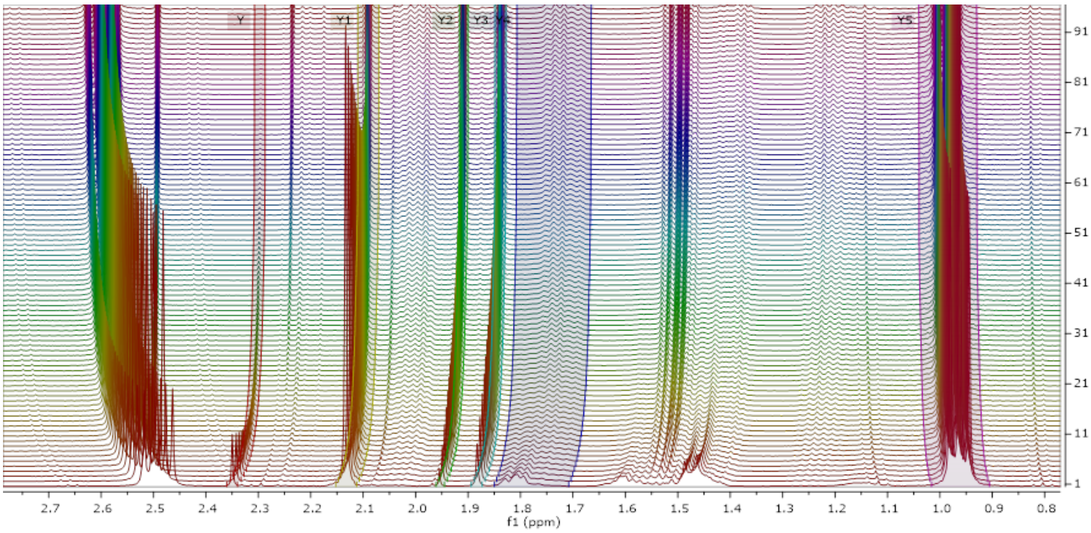

Figure S22: NMR spectra during the NMR Monitoring the Acylation of 1-ethynylcyclohexan-1-ol using SeHBTM (**ITU4**).

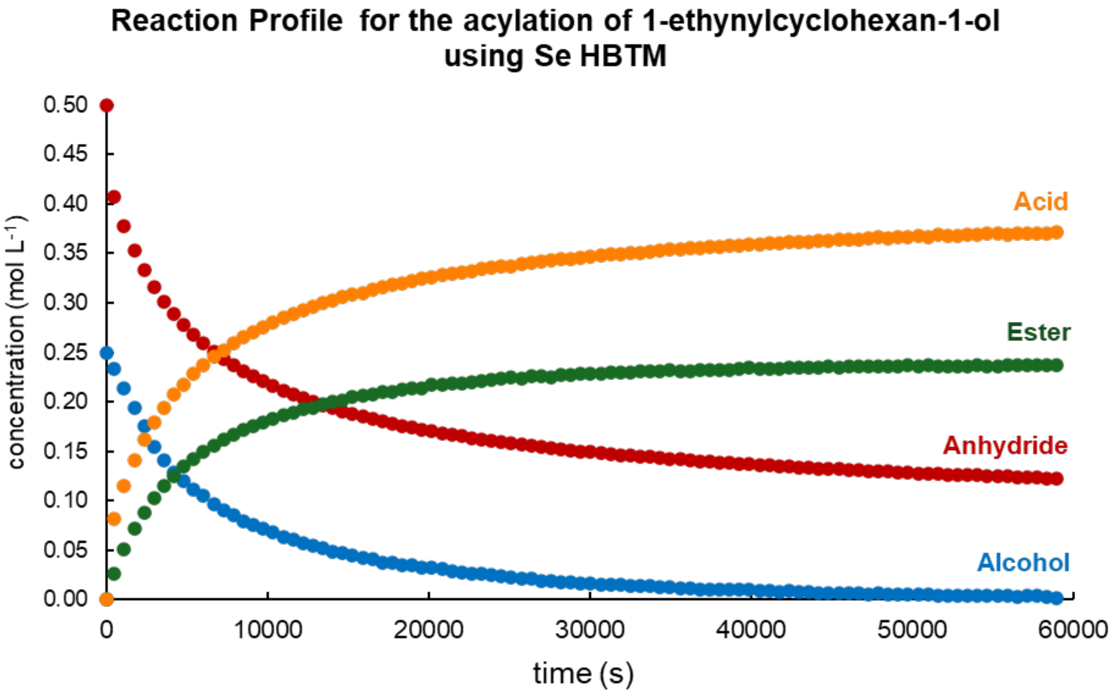

Figure S23: Reaction Profile for the acylation of 1-ethynylcyclohexan-1-ol using SeHBTM (**ISeU4**).

Table S11: Raw data for the NMR Monitoring the Acylation of 1-ethynylcyclohexan-1-ol using SeHBTM (**ISeU4**).

Time (s)	Integral(2.360,2.340)	c (alc, alkyne) (mol L^{-1})	Integral(2.152,2.113)	c (anhydride) (mol L^{-1})	Integral(1.961,1.945)	c (ester Me) (mol L^{-1})	Integral(1.892,1.871)	c (carboxylic acid Me) (mol L^{-1})	Integral(1.850,1.662)	c (alc+ester) (mol L^{-1})	Integral(1.040,0.906)	c (Me, NEt3) (mol L^{-1})
0		0.25		0.5		0		0		0.25		0.75333531
468	24585369.4	0.233864704	257213380.1	0.407784064	8258355.186	0.026185462	25933648.07	0.082230002	61120891.17	0.25	712759295.8	0.75333531
1084	21096501.47	0.2139978	223361734.8	0.377621221	15077873.97	0.0509821	34145078.78	0.115453136	57343498.44	0.25	668393111.5	0.75333531
1700	18311177.27	0.194074923	199609707.2	0.352601019	20345858.43	0.071879975	39882037.4	0.140899431	54826287.62	0.25	639701953.9	0.75333531
2316	16111946.8	0.175710956	183288850.7	0.333146779	24320913.47	0.088411641	44537700.01	0.161903916	53413609.13	0.25	621698775.3	0.75333531
2931	13934977.94	0.15501318	170705931.2	0.316489787	27625528.85	0.102435782	48287965.61	0.179052337	51601292.08	0.25	609492678.9	0.75333531
3547	12465792.5	0.141038279	159931109.4	0.301577409	30514060.11	0.115078939	51500399.93	0.194225592	51581817.93	0.25	599257843.5	0.75333531
4163	11503547.85	0.128533745	155340264.9	0.289279827	33417253.53	0.12446145	55579527.94	0.207004104	52156574.09	0.25	606799865.4	0.75333531
4779	10597186.66	0.119491209	147869120.3	0.277889162	35985287.26	0.13525368	57975833.65	0.217906968	51608387.11	0.25	601292051.3	0.75333531
5396	9778293.273	0.110986193	141521450.5	0.267717596	37557630.16	0.142096317	60243842.15	0.227927802	51517582.15	0.25	597344593.7	0.75333531
6012	9234236.632	0.104919976	136889509.6	0.259224534	39523998.18	0.149691383	62703583.66	0.237480685	50719319.25	0.25	596724196.4	0.75333531
6628	8502978.7	0.096912857	132200577.8	0.251126421	40963916.43	0.155628998	64607060.9	0.24545339	51306230.26	0.25	594867893.3	0.75333531
7243	7891883.134	0.090184132	127999911.5	0.243785519	42477385.07	0.161802789	66165879.81	0.252035852	51092406.33	0.25	593309562.1	0.75333531
7860	7491086.744	0.085559504	124558613.5	0.237107859	43756599.81	0.166588779	68013019.82	0.258937075	51064180.74	0.25	593618462.6	0.75333531
8476	6908972.251	0.079393302	120776646.1	0.231314113	44718891.2	0.171293226	69185182.35	0.265009994	50779516.25	0.25	590011418.7	0.75333531
9092	6583230.51	0.075506156	118045372.8	0.22565295	46016081.04	0.175926666	70721262.05	0.270378432	51012303.69	0.25	591136173.2	0.75333531
9707	6241767.045	0.071583896	115498472.1	0.220766292	47013966.17	0.179727035	72138885.96	0.275775672	50799398.17	0.25	591184526.4	0.75333531
10323	5893177.728	0.067733246	112714578.3	0.215914192	47812017.26	0.183175827	73109734.26	0.280095608	50778367.49	0.25	589900120.6	0.75333531
10943	5520267.271	0.063341457	110690705.4	0.211683911	48899328.31	0.187029273	74447989.34	0.284747333	50930955.72	0.25	590884893.5	0.75333531
11558	5272148.823	0.060687658	108271808.1	0.207719302	49420088.19	0.189624731	75346566.57	0.28910455	50811041.1	0.25	589003829	0.75333531
12174	5008254.351	0.057252341	106893654.4	0.203660849	50531474.9	0.192551806	76798092.39	0.292641594	51158603.77	0.25	593094583	0.75333531
12790	4753608.924	0.054406656	104925142	0.2001506	50966726.04	0.194443784	77683314.35	0.296370569	51097264.97	0.25	592382542.3	0.75333531
13406	4548140.872	0.052100521	103043627.4	0.196733377	51729473.07	0.197526314	78475348.99	0.299654056	51253225.69	0.25	591865020.4	0.75333531
14022	4229194.47	0.048496624	101304860.3	0.193612271	52328003.36	0.20001693	79009852.34	0.302004798	51232980.85	0.25	591257939.7	0.75333531
14638	4123011.512	0.047485451	99323048.72	0.190653513	52577293.23	0.201847321	79798000.73	0.306349218	50935059.13	0.25	588687499.1	0.75333531
15253	3895713.116	0.044824609	97996979.08	0.187927779	53398113.45	0.204802004	80357736.17	0.308202375	50868235.43	0.25	589252305.5	0.75333531
15869	3737447.443	0.042497854	97734841.89	0.185220932	54502586.81	0.206579757	81852607.84	0.31024384	51602003.49	0.25	596264470.2	0.75333531
16486	3584921.325	0.040918399	96051264.73	0.182722001	54446833.55	0.207152595	82444429.81	0.313674396	51153733.63	0.25	594007361.6	0.75333531

17101	3324154.027	0.038129518	94431799.76	0.180529089	54778613.92	0.209444981	82659781.54	0.316048092	51125258.73	0.25	591085981.2	0.75333531
17716	3250719.812	0.037154606	93562488.83	0.178231162	55151921.8	0.21012248	83552646.47	0.318325976	51655984.55	0.25	593195311.8	0.75333531
18332	3080697.95	0.035252483	92263428.43	0.175962017	55561824.22	0.211931658	83927285.82	0.320127157	51388009.15	0.25	592502571.9	0.75333531
18947	3014604.021	0.03458218	91052034.59	0.174084657	55838873.88	0.213519473	84320364.7	0.322428419	51094419.73	0.25	591028932.3	0.75333531
19563	2847831.664	0.03256873	90392524.36	0.172293055	56153912.9	0.214064809	85096702.1	0.324397861	51370470.62	0.25	592849319.7	0.75333531
20179	2881648.655	0.033092382	89163643.09	0.170656783	56751684.74	0.217242356	85181320.64	0.326069453	51352210.17	0.25	590396579.1	0.75333531
20794	2704335.917	0.031084115	87982544.84	0.16854782	56641744.33	0.21701674	85677447.77	0.328263909	51046287.61	0.25	589865454.4	0.75333531
21411	2505635.351	0.028763744	87204309.62	0.166845403	57069553.2	0.218378946	86197457.34	0.329838046	50900064.93	0.25	590613385.2	0.75333531
22026	2449353.69	0.027984064	86866344.01	0.165409166	57533250.52	0.219107344	86991586.84	0.331295301	51316729.93	0.25	593432811.2	0.75333531
22641	2383532.915	0.026856599	87185251.7	0.163727503	58693114.48	0.220442722	88384891.89	0.331960679	52197553.58	0.25	601729037.7	0.75333531
23257	2314631.75	0.026116474	86300549.48	0.16229119	58815128.13	0.221207794	88860022.95	0.334208737	51461529.66	0.25	600894460.5	0.75333531
23872	2206213.34	0.025177393	84646376.7	0.160997958	58455478.15	0.222365398	88148342.15	0.335317438	51443978.66	0.25	594110992.1	0.75333531
24489	2123877.643	0.024124157	84161778.03	0.159325872	59048272.7	0.223567462	89015725.54	0.33702967	51490528.95	0.25	596909072	0.75333531
25105	2006905.261	0.022587268	84254289.99	0.158043853	59796589.87	0.224332398	89919987.77	0.337343091	51973389.59	0.25	602412531.8	0.75333531
25720	1932934.4	0.021709067	83845725.58	0.156947256	59630361.29	0.223239086	90712415.65	0.339601443	52479969.14	0.25	603679993.8	0.75333531
26336	1919449.995	0.02144278	83707363.33	0.155853547	60614108.72	0.225713091	91397809.58	0.340344559	52347675.36	0.25	606913158.8	0.75333531
26951	1725004.786	0.019424899	82255269.84	0.154376413	60115727	0.225649987	90988665.57	0.341534441	52180766.61	0.25	602091323.3	0.75333531
27566	1726696.995	0.019304343	82269637.9	0.153294734	60400329.03	0.225090388	92062110.31	0.343082505	52703174.3	0.25	606445716	0.75333531
28183	1593762.618	0.017829119	81593586.61	0.152128654	60831752.25	0.226837745	92255449.67	0.34401472	52564897.51	0.25	606072506.1	0.75333531
28798	1655397.701	0.018341247	81801869.41	0.151056179	61559835.71	0.227354061	93369584.36	0.344834483	53155678.1	0.25	611933622	0.75333531
29414	1518835.708	0.016886817	81006440.64	0.150108504	61743621.83	0.228827304	93228641.01	0.34551356	52762056.24	0.25	609809008.6	0.75333531
30030	1483752.769	0.0165517	80430468.68	0.149537599	61412142.05	0.228356852	93276600.06	0.34684266	52235139.3	0.25	607784720.3	0.75333531
30645	1423780.928	0.015774514	80445290.31	0.148546422	61787541.8	0.228187833	94110312.37	0.347559194	53019223.64	0.25	611952920.8	0.75333531
31261	1410096.741	0.015616349	79833430.68	0.147354751	62112069.34	0.229290121	94487636.66	0.348806309	53042984.69	0.25	612209738.2	0.75333531
31876	1355735.486	0.014838364	80294144.46	0.146468311	62832346.26	0.229230853	95830226.16	0.34961681	53813185.82	0.25	619469296	0.75333531
32493	1348153.094	0.014700027	80213619.51	0.145772563	63381452.03	0.230366782	96474419.14	0.35064677	53823165.95	0.25	621801704.2	0.75333531
33109	1335863.882	0.014497768	80131204.6	0.144940365	63727657.42	0.23053965	97028240.65	0.351007044	54117415.97	0.25	624729341.3	0.75333531
33724	1260507.017	0.013596704	80208677.87	0.144197747	64394666.46	0.231535191	97902585.62	0.352015084	54525057.85	0.25	628553815.7	0.75333531
34340	1219607.588	0.013231871	79360769.66	0.143501271	63783184	0.230667319	97627946.7	0.353064481	53888786.73	0.25	624927599.4	0.75333531
34955	1141377.411	0.012359623	79012270.19	0.142599904	64186506.55	0.231685273	98130398.3	0.354207906	54472323.85	0.25	626116125.5	0.75333531
35571	1146170.409	0.012211816	79862716.97	0.141815565	64906102.21	0.230512958	99575300.45	0.353640047	54992880.17	0.25	636355445.5	0.75333531

36186	1037092.966	0.011104778	78858190.43	0.140730346	64677824.95	0.230848124	99598538.21	0.355487151	55060014.77	0.25	633196689.3	0.75333531
36802	1014394.136	0.010688678	79812844.39	0.140164745	66118159.99	0.232229164	101261470.8	0.355664263	56122120.83	0.25	643448184.1	0.75333531
37417	1034380.727	0.010945159	79038087.38	0.139388467	65908632.41	0.232467752	101008967.1	0.356270896	55435556.81	0.25	640750807.4	0.75333531
38032	987103.972	0.010457149	78628931.65	0.138829428	65810733.13	0.232394521	101122789	0.357090417	55823230.68	0.25	640000660.5	0.75333531
38648	969995.44	0.010184205	78882092.69	0.138033537	66244077.13	0.231837265	102258145.9	0.35787726	56060789.82	0.25	645763343.6	0.75333531
39265	973576.938	0.010014862	80129555.02	0.137377675	68141453.9	0.233649481	104362305.2	0.357846759	57380889.03	0.25	659107347.7	0.75333531
39881	948752.258	0.009864715	79002770.44	0.136906102	67557376.53	0.23414412	103533270.5	0.358831378	56472782.08	0.25	652077325.7	0.75333531
40497	867980.286	0.008988772	79171445.41	0.136649437	67671957.74	0.233602782	104117367.8	0.359411898	56386420.63	0.25	654696936.1	0.75333531
41113	877781.244	0.009159103	78133200.55	0.135878581	67227734.96	0.233826572	103488613.1	0.359946497	56358270.61	0.25	649776790.3	0.75333531
41728	776148.71	0.008033492	78255990.26	0.134997505	67728216.83	0.233672599	104517670.8	0.360601783	57044864.3	0.25	655045446.1	0.75333531
42345	897418.17	0.00930733	77876593.44	0.134612669	67864269.27	0.234611968	104491925.7	0.361236577	56660405	0.25	653733277.3	0.75333531
42960	731429.856	0.007507995	78292796.58	0.133943568	68713257.83	0.235109724	105663105.3	0.36153756	57638432.36	0.25	660510196.6	0.75333531
43575	728493.428	0.007413806	78534961.51	0.133207106	69014143.45	0.234116733	106743593.2	0.362106374	58038361.05	0.25	666216257.4	0.75333531
44192	661110.278	0.006738176	78208256.18	0.13285252	69208234.58	0.235128331	106737860.5	0.36263163	57277218.63	0.25	665215544.6	0.75333531
44808	676250.385	0.006923291	77603573.7	0.132414499	69068903.12	0.235703687	106347432.1	0.362919936	57197859.88	0.25	662255785.4	0.75333531
45424	714842.378	0.007293913	77532281.96	0.131850445	68883444.78	0.234284678	107124001.1	0.364347518	57765582.29	0.25	664477911	0.75333531
46039	663578.111	0.006732098	77739530.06	0.131446504	69699950.04	0.235705431	107630788.2	0.363976751	57970333.2	0.25	668301529.7	0.75333531
46655	633956.215	0.006365131	78222305.51	0.130896313	70442113.87	0.235754059	108764160.2	0.364009409	58524812.35	0.25	675278278.3	0.75333531
47271	603781.75	0.006077496	77775128.02	0.130477065	70462151.1	0.236417346	108722574	0.364790203	58363327.6	0.25	673575277.1	0.75333531
47886	619163.006	0.006212319	77785326.09	0.130075397	70372506.44	0.235358831	109571744.4	0.366459559	58031524.84	0.25	675743845.9	0.75333531
48502	571014.032	0.005633035	78722467.88	0.129432427	71923361.38	0.2365072	111014489.8	0.365051434	59582672	0.25	687282344.4	0.75333531
49118	571166.998	0.005618233	78577321.7	0.128819799	71877245.31	0.235671364	111839284.4	0.366699037	59594739.77	0.25	689277636.5	0.75333531
49734	580259.298	0.005681857	78903167.13	0.128769021	72552128.07	0.236808403	112325574.4	0.366627976	59862379.73	0.25	692408874.3	0.75333531
50351	571925.713	0.005575002	78802420.85	0.128024684	72584239.81	0.235844896	113068149.4	0.367387549	60066311.18	0.25	695545314.1	0.75333531
50966	594715.76	0.005658399	80468032.63	0.127601642	74627290.52	0.236679449	115610645.1	0.366657607	61366332.87	0.25	712601452.1	0.75333531
51583	499793.354	0.004790655	79626494.81	0.127206941	73720742.66	0.235544468	115434763.2	0.368824551	61701719.77	0.25	707336993.2	0.75333531
52199	439931.017	0.00419116	79798992.53	0.12670559	74308196.84	0.235974507	115988258.1	0.368334493	61807131.81	0.25	711674187.7	0.75333531
52815	445683.33	0.004200307	80652404.02	0.126683706	75234791.27	0.236348124	117251445.9	0.368342343	62014158.66	0.25	719409451.8	0.75333531
53430	488120.59	0.004598527	80208581.51	0.125939277	75021823.04	0.235590606	117460063.6	0.368859173	62786998.88	0.25	719679649.3	0.75333531
54046	423338.184	0.00396884	80447203.41	0.12570017	75789251.75	0.236844077	117900470.9	0.368443118	62371021.18	0.25	723193758.5	0.75333531
54662	445973.076	0.004126516	81244842.77	0.125290877	76652675.55	0.23641823	119911355.4	0.369840064	63401952.58	0.25	732750181.5	0.75333531

55277	475072.52	0.004426794	80392430.85	0.124851371	76128271.04	0.236458058	119060354.8	0.369807167	62577012.94	0.25	727614636.6	0.75333531
55893	439756.277	0.004042801	81232947.5	0.124466159	77317239.66	0.236932922	120465138.1	0.369156443	63600230.37	0.25	737497425	0.75333531
56509	374323.181	0.003440518	80970340.85	0.124037177	77435455.45	0.237244285	120788820.9	0.370068948	63840129.46	0.25	737655653.6	0.75333531
57125	416227.058	0.003799817	81260779.23	0.123640872	77861857.7	0.236938609	121537962.2	0.369847786	64364020.77	0.25	742674489	0.75333531
57742	448262.847	0.004040319	82074733.12	0.123293769	78986810.59	0.237310101	123219705.3	0.370204601	65309271.46	0.25	752225294	0.75333531
58357	402649.484	0.003552172	83634143.3	0.122970008	80591849.1	0.236993647	126008952.2	0.370550142	66776292.38	0.25	768535609.4	0.75333531
58973	172101.548	0.001518362	83161949.06	0.122282439	80451238.9	0.236593148	126212090.4	0.37116788	66272527.41	0.25	768493416.8	0.75333531

SeDHPB (ISeU3)

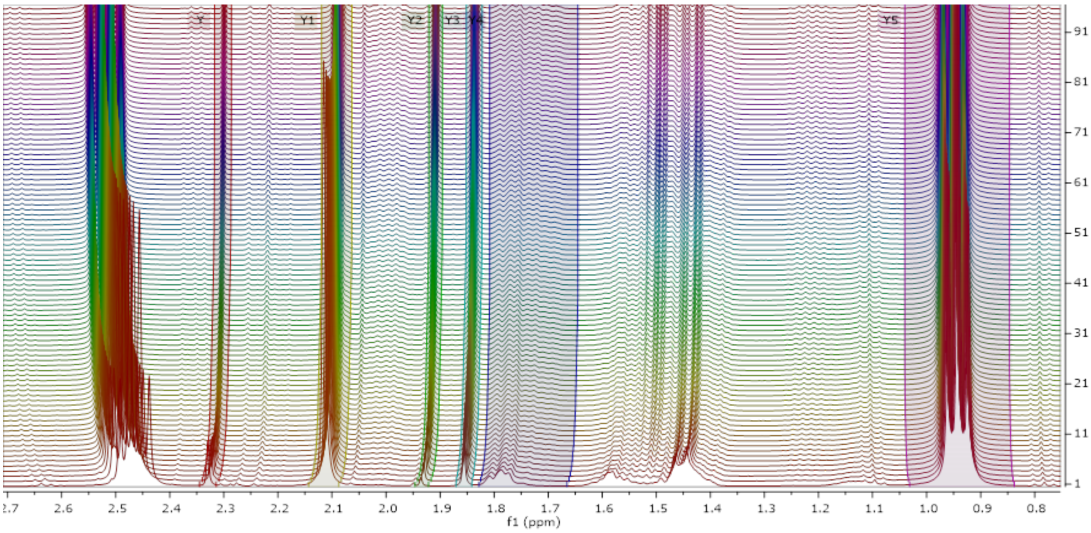

Figure S24: NMR spectra during the NMR Monitoring the Acylation of 1-ethynylcyclohexan-1-ol using SeDHPB (**ISeU3**).

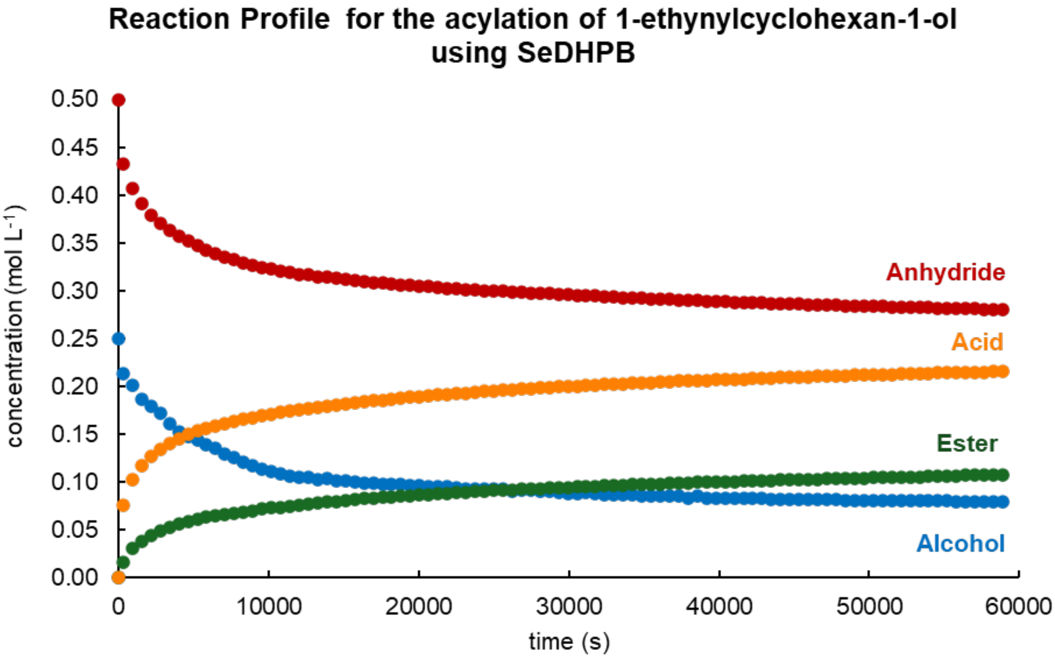

Figure S25: Reaction Profile for the acylation of 1-ethynylcyclohexan-1-ol using SeDHPB (**ISeU3**).

Table S12: Raw data for the NMR Monitoring the Acylation of 1-ethynylcyclohexan-1-ol using Se*DHPB* (**ISeU*3***).

Time (s)	Integral(2.345,2.314)	c (alc, alkyne) (mol L^{-1})	Integral(2.149,2.078)	c (anhydride) (mol L^{-1})	Integral(1.952,1.912)	c (ester Me) (mol L^{-1})	Integral(1.874,1.846)	c (carboxylic acid Me) (mol L^{-1})	Integral(1.828,1.665)	c (alc+ester) (mol L^{-1})	Integral(1.031,0.838)	c (Me, NEt3) (mol L^{-1})
0		0.25		0.5		0		0		0.25		0.75333531
308	20727979.89	0.213755058	252063203.6	0.433229103	4671230.221	0.016057186	22010506.4	0.075660325	50361253.63	0.25	657463142.7	0.75333531
925	18549244.18	0.201717221	224477461.5	0.406853682	8340766.513	0.030234408	28190670.27	0.102188237	48210013.62	0.25	623467867	0.75333531
1542	16882363.82	0.186122245	212754691.2	0.390924529	10487967.26	0.038542075	31789503.63	0.116822775	47394651.93	0.25	614986817.7	0.75333531
2158	16163780.15	0.179939783	204445332.6	0.379323901	11967199.29	0.044407418	34182583.54	0.126843404	47071217.78	0.25	609041063.9	0.75333531
2775	15485964.36	0.171948732	200167103.4	0.37042661	13316989.23	0.04928849	36346789.6	0.134525782	47376287.64	0.25	610618714.4	0.75333531
3392	14585597.59	0.161517385	196690275.2	0.363016767	14340442.84	0.0529342	38075618.3	0.140546734	47504998.07	0.25	612259857.5	0.75333531
4008	13764481.58	0.152806889	192907967	0.356929126	15125198.9	0.05597098	39283946.96	0.145370718	47471698.89	0.25	610727897.5	0.75333531
4624	13295151.89	0.148098592	189597083.5	0.351996244	15853213.02	0.058864528	40327504.77	0.149739964	47292485.41	0.25	608657819.7	0.75333531
5241	12806182.49	0.143798838	185604569.7	0.34735464	16359786.87	0.061233922	40981137.39	0.153390493	47002776.34	0.25	603802827.9	0.75333531
5858	12552572.25	0.139234334	185572000	0.343063734	17072947.99	0.063124925	42306812.18	0.156423738	47684389.45	0.25	611247675.6	0.75333531
6474	12065893.39	0.135163302	181722356.4	0.339278561	17403155.64	0.064983943	42631620.47	0.159187842	47278552.53	0.25	605245437.9	0.75333531
7091	11676900.74	0.129814425	181003630	0.335375552	17894021.12	0.066310462	43554548.56	0.161401523	47612182.77	0.25	609867467.5	0.75333531
7707	11339798.66	0.126272723	179106696.2	0.332402879	18284892.24	0.06786961	44115164.4	0.163746057	47663302.04	0.25	608872878.4	0.75333531
8324	10864662.12	0.121481398	176729510.1	0.32934523	18537061.72	0.069089682	44431498.91	0.165601118	47349178.79	0.25	606369402.3	0.75333531
8941	10463612.28	0.117520241	174696435.7	0.327012106	18779362.49	0.070305715	44777451.17	0.167636718	47188147.31	0.25	603670283	0.75333531
9557	10166070.16	0.113656188	174257743.6	0.3246989	19233260.63	0.071675651	45469242.23	0.169447999	47385658.84	0.25	606444206.6	0.75333531
10174	9906917.072	0.11071112	173147987	0.322491977	19547699.84	0.072816052	45933702.3	0.171105085	47459005.78	0.25	606705755.4	0.75333531
10791	9611887.078	0.108168006	170981987.5	0.320692848	19684777.16	0.073841313	46039725.19	0.172703696	47238926.43	0.25	602477274.5	0.75333531
11408	9477437.546	0.105922002	171204407.7	0.318903246	20100085.15	0.074881044	46706155.34	0.173999545	47666457.37	0.25	606646344.6	0.75333531
12025	9360708.015	0.104678802	170370756.5	0.31753664	20349764.44	0.075855692	47088802.72	0.175528013	47583166.04	0.25	606290537.8	0.75333531
12641	9409904.027	0.105562548	169376825.7	0.316684939	20693070.61	0.077379934	47397359.88	0.177238297	47900950.62	0.25	604374541.9	0.75333531
13257	9180569.105	0.10287978	168698797.1	0.315080233	20917634.97	0.078136103	47702566.42	0.178189009	47639952.24	0.25	605020947.2	0.75333531
13874	9268272.747	0.104047015	168028332.8	0.314385195	21198094.12	0.079324324	48086047.55	0.179940385	47957320.74	0.25	603948650.6	0.75333531
14491	9022853.896	0.101294471	167231534	0.312902289	21379061.85	0.08000354	48277518.42	0.180661454	47668755.43	0.25	603933355.6	0.75333531
15108	9039809.672	0.100942932	167607298	0.311930832	21755762.53	0.080978492	48867270.88	0.181891942	47930076.4	0.25	607175451.1	0.75333531
15725	8968241.575	0.100267561	166747783.9	0.310714823	21925831.49	0.0817124	49071708.6	0.182878679	47891646.68	0.25	606425817.1	0.75333531

16342	8860853.479	0.099459128	165579791.3	0.309759884	22065157.74	0.082557185	49139639.28	0.183856844	47728917.01	0.25	604034495.1	0.75333531
16958	8748021.1	0.098730632	164181068.7	0.308826047	22117007.07	0.083204573	49135443.69	0.184848411	47478315.08	0.25	600743026.7	0.75333531
17575	8701479.897	0.098184091	163777754	0.308000671	22324109.9	0.083965504	49413247.06	0.185853242	47505436	0.25	600873195.6	0.75333531
18192	8676087.643	0.097620859	163783431.9	0.307140729	22564109.26	0.084628303	49790043.16	0.186741113	47648593.17	0.25	602576428.9	0.75333531
18809	8809145.915	0.09835441	164657794.2	0.306401633	22944081.71	0.085390481	50478539.25	0.187864861	48413214.02	0.25	607254582.6	0.75333531
19426	8622920.971	0.096617866	163631533.3	0.305575677	23011058.9	0.085944558	50481902.1	0.188546072	47933633.49	0.25	605100901.6	0.75333531
20044	8505732.022	0.095949298	162132861.7	0.304824523	22984861.41	0.086427259	50369962.18	0.189400218	47635553.55	0.25	601036335.5	0.75333531
20660	8534022.272	0.095624047	162887153.2	0.304192801	23345383.77	0.087195307	50937196.4	0.19025108	47870321.66	0.25	605086529.5	0.75333531
21277	8535553.849	0.095161189	163263251.6	0.303364908	23604880.88	0.087722037	51411083.24	0.191057307	48160022.37	0.25	608138756.9	0.75333531
21894	8394852.58	0.094970177	160660268.5	0.302922414	23422045.57	0.088323799	50890071.66	0.191904863	47533983.48	0.25	599317089.3	0.75333531
22511	8284261.304	0.093845066	160129970.8	0.302328459	23516722.18	0.088800046	51001724.18	0.192584469	47415021.06	0.25	598512432.6	0.75333531
23128	8235735.902	0.093420391	159521932.9	0.301584085	23674139.54	0.089514258	51095377.33	0.193196664	47319922.63	0.25	597711437.1	0.75333531
23744	8225848.627	0.093412704	159008000.8	0.300949035	23754747.78	0.089919606	51239400.79	0.193958142	47388867.75	0.25	597042991.8	0.75333531
24360	8175071.32	0.093125238	158275524.6	0.300495764	23836262.57	0.090509205	51280408.17	0.194717983	47268001.7	0.25	595189128.4	0.75333531
24977	8116802.825	0.093065935	157087735	0.300190384	23835309.72	0.091097256	51149892.17	0.195492102	47067549.84	0.25	591323425.7	0.75333531
25593	8125213.807	0.092425244	157935060.7	0.299421588	24103372.82	0.091392882	51726935.09	0.196133284	47443375.56	0.25	596039478.2	0.75333531
26210	7912605.25	0.090338892	156932813.1	0.298619227	24124530.6	0.091810611	51610745.31	0.196414766	46862045.77	0.25	593848378.7	0.75333531
26828	8060460.946	0.091660157	157450840.4	0.29841074	24435356.15	0.092622849	52093633.76	0.197462265	47437771.59	0.25	596224909	0.75333531
27444	7968802.88	0.091122212	156373225.1	0.298017874	24397246.21	0.0929931	51933276.06	0.197950059	47269141.2	0.25	592924865.5	0.75333531
28061	7928014.337	0.090501167	156329673.2	0.29742668	24564854.06	0.093472248	52212513.34	0.198674943	47267735.02	0.25	593937956.4	0.75333531
28679	7862946.029	0.09004686	155568578.8	0.296929887	24561965.1	0.093761627	52157366.59	0.199102943	47146985.64	0.25	592035234.8	0.75333531
29296	7797055.506	0.089666621	154810411.3	0.296721553	24632229.59	0.094424055	52102167.01	0.199726049	46904384.04	0.25	589563589.5	0.75333531
29912	7696644.996	0.088031739	155170167.5	0.295797707	24779892.43	0.094474801	52460491.68	0.200008718	47016064.03	0.25	592779271.9	0.75333531
30530	7711581.868	0.088027007	155192698.8	0.295251762	25005484.05	0.09514511	52725674.23	0.200619595	47052278.85	0.25	593961603.7	0.75333531
31146	7727790.476	0.088769935	154191144.9	0.295201627	24954311.73	0.095550927	52567098.37	0.201281247	47062225.58	0.25	590228626.4	0.75333531
31764	7711164.736	0.088120065	154635946.1	0.294519484	25175160.05	0.095897174	52969916.12	0.201772909	47332838.6	0.25	593302266.9	0.75333531
32379	7609052.229	0.086909798	154431741.2	0.293983865	25246282.47	0.096120132	53071135.41	0.202057652	47157919.3	0.25	593598310.3	0.75333531
32996	7573706.933	0.086723459	153820485.5	0.293556042	25327118.42	0.096670201	53040604.37	0.202448846	47069753.37	0.25	592110464.7	0.75333531
33612	7549563.337	0.086289716	153830296.9	0.293040628	25427737.95	0.096877669	53247515.36	0.202868819	47169610.44	0.25	593189732.7	0.75333531
34228	7519211.292	0.086222354	153155187	0.292703587	25427926.53	0.097193513	53212605.34	0.203395272	47089740.4	0.25	591266465.2	0.75333531
34845	7495335.389	0.085764649	153237185.1	0.292233606	25614429.9	0.097696877	53451997.1	0.203873099	47103398.41	0.25	592534430.1	0.75333531

35461	7494657.257	0.085612452	153217027.9	0.291703031	25706531.8	0.097883027	53627386.39	0.204197554	47304745.66	0.25	593534099	0.75333531
36077	7479608.455	0.085359023	153239110.2	0.291466698	25844134.54	0.098313081	53800132.81	0.204659855	47293939.91	0.25	594100972.4	0.75333531
36693	7502082.36	0.08560468	153074156.9	0.291116154	25958865.81	0.098737048	53954434.22	0.20522089	47351738.88	0.25	594176065.8	0.75333531
37309	7476056.944	0.0852441	152971685.8	0.29070435	26071626.05	0.099091999	54110163.37	0.205659757	47414597.2	0.25	594619442.6	0.75333531
37925	7319230.6	0.083469962	152540933.3	0.289934526	26072419.85	0.099111688	54081648.98	0.205585962	47035200.46	0.25	594519422.6	0.75333531
38541	7473383.503	0.085173028	152676133.9	0.290004489	26228289.48	0.099639957	54337549.52	0.206425626	47520394.25	0.25	594902805.7	0.75333531
39159	7341421.551	0.083729599	152210121.3	0.289328443	26281852.91	0.099915663	54318286.91	0.206501713	47287126.47	0.25	594472795.6	0.75333531
39776	7344601.849	0.083635296	152226014.2	0.288907599	26371332.39	0.100099558	54501265.32	0.206874361	47283836.57	0.25	595400909.3	0.75333531
40392	7325016.476	0.083330424	152153617.7	0.288486848	26457277.33	0.100327244	54641117.5	0.207201695	47397663	0.25	595985709.7	0.75333531
41008	7284761.374	0.083063026	151710512.6	0.288308099	26466782.04	0.100594052	54624205.13	0.207613836	47387634.38	0.25	594618497.9	0.75333531
41624	7274428.177	0.082987105	151427381.5	0.287915413	26535655.25	0.100906772	54673625.85	0.207906647	47286384.54	0.25	594318269.9	0.75333531
42241	7293797.08	0.083065579	151481677.2	0.287525439	26676762.83	0.101269647	54855988.49	0.208242904	47357555.91	0.25	595337737.1	0.75333531
42859	7281390.565	0.082682769	151753745.1	0.287202922	26808777.8	0.10147439	55121229.38	0.208640362	47427792.22	0.25	597076730.5	0.75333531
43475	7261291.549	0.082399661	151670123.8	0.286853625	26911564.9	0.101795657	55210310.95	0.208838464	47564291.98	0.25	597474371.8	0.75333531
44091	7269070.022	0.082298346	151879217.9	0.286588893	27039708.38	0.102045299	55460595.4	0.209303035	47614120.9	0.25	598850725.2	0.75333531
44708	7251881.305	0.082252205	151463601.9	0.286321453	27038883.19	0.102226703	55449854.82	0.209640901	47620718.99	0.25	597769804.5	0.75333531
45325	7209561.838	0.081831213	151163319.7	0.285959999	27117793.27	0.102599019	55482796.09	0.209916802	47508831	0.25	597338789.6	0.75333531
45942	7238532.412	0.082051042	151221170.5	0.285689922	27204487.94	0.102790476	55648067.93	0.210262784	47645963.1	0.25	598132303.1	0.75333531
46558	7210231.147	0.081692411	151140639.3	0.285405627	27274709.88	0.103008108	55754569.2	0.210567692	47641531.04	0.25	598409261.6	0.75333531
47174	7200982.863	0.081624837	150878755.1	0.285041037	27329942.8	0.103263779	55799586.87	0.210833819	47676000.22	0.25	598136473.3	0.75333531
47790	7189438.619	0.081562925	150613324.7	0.284780308	27376420.48	0.103526902	55837841.17	0.21115685	47651357.86	0.25	597630871.3	0.75333531
48406	7148345.199	0.081020734	150605458.1	0.28449859	27440622.09	0.103672449	55948793.93	0.211378169	47591013.28	0.25	598191414.1	0.75333531
49022	7136043.057	0.080933585	150434974.6	0.284360249	27512151.32	0.104009885	55996261.08	0.211694266	47518150.85	0.25	597804960.4	0.75333531
49638	7150630.54	0.08099417	150456610.5	0.28403342	27644414.47	0.104374777	56115526.62	0.211870849	47703220.8	0.25	598578914.1	0.75333531
50255	7171654.227	0.081239433	150223968.6	0.28361913	27680557.52	0.104520413	56210522.73	0.212248148	47691275.78	0.25	598526375.9	0.75333531
50872	7147811.169	0.080864502	150356674.5	0.283502116	27762686.83	0.104694793	56344693.08	0.21247929	47769921.43	0.25	599302361.7	0.75333531
51488	7144773.815	0.080900082	150076941.2	0.28321953	27809947.86	0.104963765	56374196.81	0.212774506	47764028.85	0.25	598784231.3	0.75333531
52105	7102673.976	0.080569489	149740641.2	0.283098238	27824106.31	0.105207984	56324503.2	0.212973145	47640850.27	0.25	597698416.4	0.75333531
52722	7112110.813	0.080443307	149981202.2	0.282733311	27960091.19	0.105416533	56574540.38	0.213300159	47744920.22	0.25	599431324.3	0.75333531
53339	7105488.019	0.080262842	150035370.4	0.28246395	28061903.73	0.105661434	56671195.81	0.213383948	47889432.58	0.25	600219651.2	0.75333531
53956	7107054.172	0.080361102	149783331.5	0.282272451	28112908.76	0.105959716	56726793.32	0.213807649	47875315.7	0.25	599617879.8	0.75333531

54572	7112983.306	0.080433892	149672900.6	0.282084496	28179007.7	0.106216438	56790668.14	0.214063694	47809100.44	0.25	599575033.7	0.75333531
55189	7081516.512	0.080099947	149508784.2	0.281852192	28220102.01	0.106400405	56800433.95	0.214159013	47786034.41	0.25	599411230.5	0.75333531
55804	7078541.585	0.080031419	149532835.6	0.281774733	28304601.22	0.10667251	56937046.22	0.214580576	47777229.19	0.25	599672461.5	0.75333531
56420	7071082.612	0.079970724	149396740.7	0.281601515	28362312.52	0.106921612	56952812.77	0.214703457	47779268.34	0.25	599495211.2	0.75333531
57037	7055811.572	0.079820266	149175993.5	0.281263831	28387522.1	0.10704649	57024636.15	0.215034165	47788686.91	0.25	599328091.3	0.75333531
57653	7024663.877	0.079409554	149147515.9	0.281003664	28460175.24	0.107241659	57074806.97	0.21506533	47736595.33	0.25	599768461.2	0.75333531
58269	6982837.758	0.079090898	148868460.8	0.281025678	28428394.12	0.107331112	57037641.95	0.215345035	47571428.65	0.25	598599400.4	0.75333531
58885	6978219.499	0.078943356	148829278.1	0.280613191	28529951.26	0.107584754	57205466.81	0.215718423	47658357.53	0.25	599321524.4	0.75333531

10.2 Acylative Kinetic Resolution of 4-Hydroxy[2.2]paracyclophane

Following our published work[18], 4-Hydroxy[2.2]paracyclophane (25 mg, 0.11 mmol) and HyperSeBTM (10 mol%) were dissolved in dry toluene (0.025 mol L^{-1}) in a Schlenk flask. The solution was then cooled to -84°C and 1.25 eq. of diisopropylethylamine as well as 1.25 eq. of isobutyric anhydride were added and stirred for 24 h. The reaction was quenched with MeOH at -84°C and then warmed to room temperature. The crude product was filtered over Na_2SO_4 and the solvents removed in vacuum, before the desired products was purified by column chromatography. The product was isolated as off-white solid in a yield of 38% and the starting material was recovered in a yield of 43%. Spectroscopic data was in accordance with literature.[15]

Table S13: Re-Optimization of the acylative kinetic resolution of 4-hydroxy[2.2]paracyclophane.

Entry	ITU	Solvent	T / °C	t / h	conv. (*C*) / %	ee (SM) / %	ee (product) / %	*s*
1	**BTM**	$CHCl_3$	25	1	41	42	60	6
2	**BTM**	PhMe	25	1	38	39	64	6.5
3	**BTM**	PhMe	-15	1	34	38	74	10
4	**BTM**	PhMe	-78	1	15	13	74	7.5
5	**HyperBTM**	PhMe	-78	1	16	16	85	14.5
6	**HyperBTM**	PhMe	-40	4	30	35	82	14
7	**HyperBTM**	$CHCl_3$	-40	4	45	55	67	9
8	**HyperBTM**	PhMe (0.11 mol L^{-1})	-40	4	33	37	75	10
9	**HyperBTM**	PhMe (0.012 mol L^{-1})	-40	4	16	16	85	14.5
10	**HyperBTM**	PhMe	-40	4	30	34	79	12
11	**HyperBTM**	PhMe	-40	24	57	94	71	20
12	**SeHyperBTM**	PhMe	-84	24	48	80	86	33

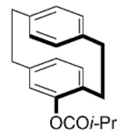

(*S*$_p$)-**23**

Acylated compound (*Sp)*-**23**:

^{1}H-NMR (300 MHz, $CDCl_3$, 298 K): δ / ppm = 6.91 (dd, *J* = 7.8, 1.8 Hz, 1 H), 6.56-6.43 (m, 5 H), 6.00 (d, *J* = 1.7 Hz, 1 H), 3.17 - 2.94 (m, 7 H), 2.85 (s, *J* = 7.2 Hz, 1 H), 2.73- 2.64 (m, 1 H), 1.34 (t, *J* = 7.5 Hz, 3 H), 1.40 (dd, *J* = 11.9, 7 Hz, 6 H).

Alcohol *(Rp)*-**22**:

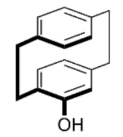

(*R*$_p$)-**22**

^{1}H-NMR (300 MHz, $CDCl_3$, 298 K): δ / ppm =7.00 (dd, *J* = 8, 1.8 Hz, 1 H), 6.55 (dd, *J* = 8, 1.8 Hz, 1 H), 6.45 (dd, *J* = 8, 1.8 Hz, 1 H), 6.41-6.37 (m, 2 H), 6.26 (dd, *J* = 8, 1.8 Hz, 1 H), 5.54 (d, *J* = 1.6 Hz, 1 H), 4.42 (s, 1 H), 3.37-3.29 (m, 1 H), 3.14-3.02 (m, 4 H), 2.98-2.85 (m, 2 H), 2.71-2.60 (m, 1 H).

Chromatogram

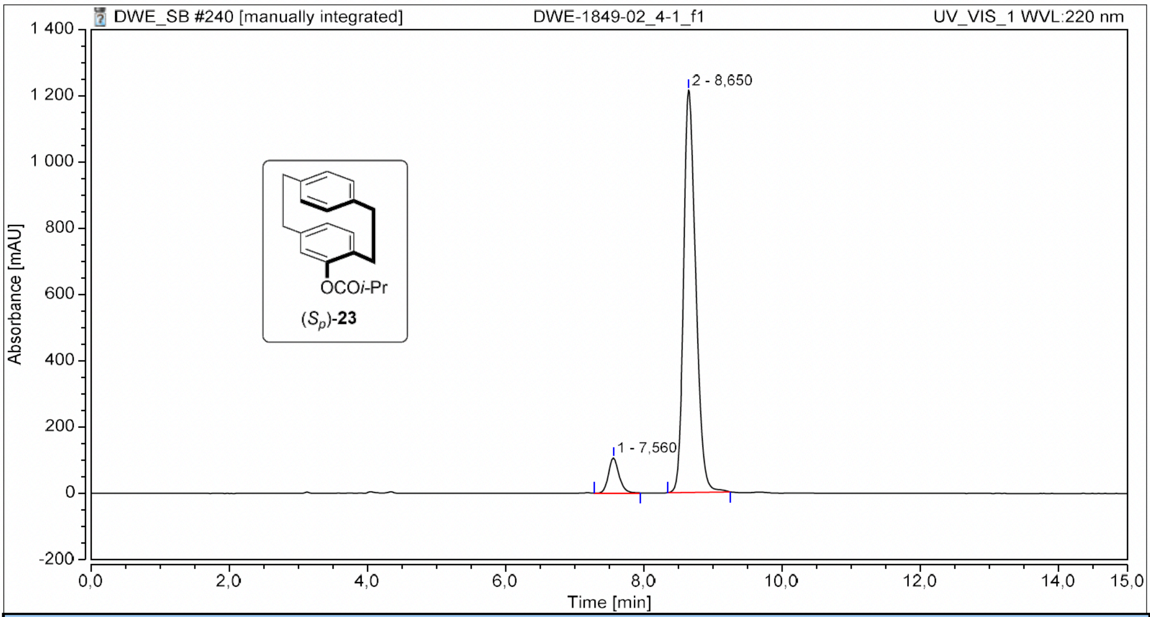

Integration Results

No.	Peak Name	Retention Time min	Area mAU*min	Height mAU	Relative Area %	Relative Height %	Amount n.a.
1		7,560	18,744	106,201	7,03	8,04	n.a.
2		8,650	247,732	1214,281	92,97	91,96	n.a.
**Total:**			**266,476**	**1320,481**	**100,00**	**100,00**	

Figure S26: HPLC chromatogram of acylated product (YMC-SB, hexane/IPA = 4/1, 1ml/min).

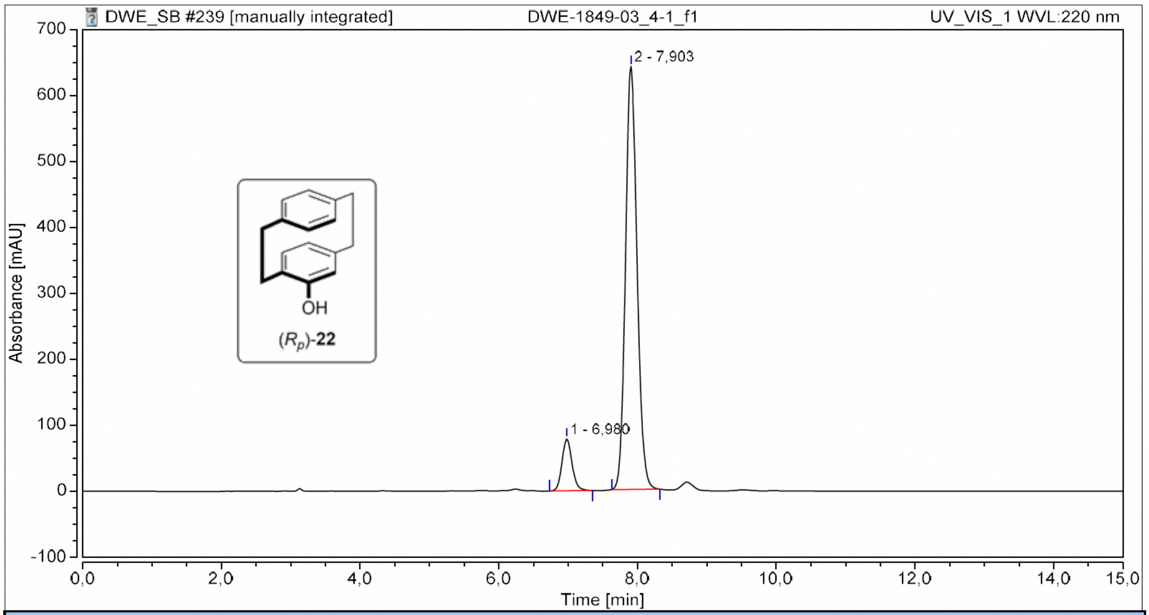

Integration Results

No.	Peak Name	Retention Time min	Area mAU*min	Height mAU	Relative Area %	Relative Height %	Amount n.a.
1		6,980	13,302	78,893	9,74	10,95	n.a.
2		7,903	123,250	641,402	90,26	89,05	n.a.
**Total:**			**136,552**	**720,295**	**100,00**	**100,00**	

Figure S27: HPLC chromatogram of recovered starting material (YMC-SB, hexane/IPA = 4/1, 1ml/min.

11. Kinetic Studies

11.1 General Information

All kinetic measurements were carried out at 20 °C in dry solvents (dichloromethane, acetonitrile, or THF). The kinetics of the adduct formation of the isochalcogenoureas with the benzhydrylium ions (structures are shown in Figure S1, counterion: tetrafluoroborate BF_4^-) were determined under pseudo-first-order conditions by using the nucleophilic isochalcogenoureas in excess concentrations. The decay of the absorbance of solutions of the coloured cations at (or close to) their respective absorption maxima was determined photometrically by standard stopped-flow techniques (AppliedPhotophysics SX.20 systems).

First-order rate constants k_{obs} (s^{-1}) were determined by least-squares fitting of the mono-exponential function $A = A_0 \cdot e^{-k_{obs}t} + C$ to the time-dependent absorbance. As k_{obs} and the concentration of the nucleophilic catalyst correlated linearly, second-order order rate constants (k_2, in L mol^{-1} s^{-1}) could be derived from the slopes of the plots of k_{obs} vs. [nucleophile].

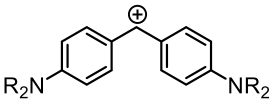

abbreviation	NR_2	E[a]
$(pfa)_2CH^+$	$N(Ph)CH_2CF_3$	–3.14
$(mfa)_2CH^+$	$N(Me)CH_2CF_3$	–3.85
$(dpa)_2CH$	$N(Ph)_2$	–4.72
$(mor)_2CH^+$	$N(CH_2CH_2)_2O$	–5.53
$(mpa)_2CH^+$	$N(Ph)CH_3$	–5.89
$(dma)_2CH^+$	NMe_2	–7.02
$(pyr)_2CH^+$	$N(CH_2)_4$	–7.69

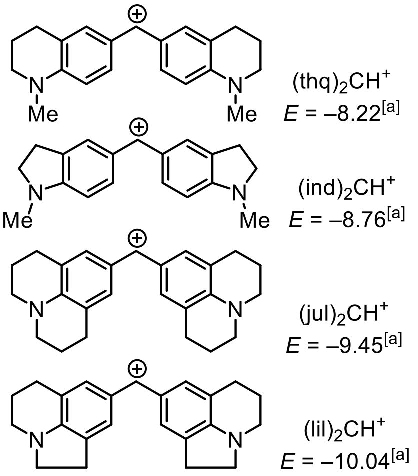

Figure S28: Benzhydrylium ions (Ar_2CH^+) used as reference electrophiles to determine the nucleophilic reactivity of the IChU catalysts. [a] Electrophilicity parameters E are taken from literature.[19]

11.2 Kinetic Studies in Dichloromethane

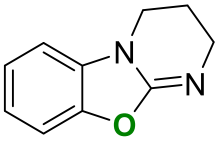

IU3
ODHPB

Table S14: Kinetics of the reactions between ODHPB (**IU3**) and benzhydrylium ions $(Ar)_2CH^+$ in CH_2Cl_2 at 20 °C.

$[(ind)_2CH^+]$ (mol L^{-1})	[ODHPB] (mol L^{-1})	k_{obs} (s^{-1})	λ = 625 nm	k_2 (L mol^{-1} s^{-1})
1.00×10^{-5}	4.00×10^{-4}	4.18		1.20×10^{3}
	8.00×10^{-4}	4.69		LSM-053
	1.20×10^{-3}	5.10		
	1.60×10^{-3}	5.62		
	2.00×10^{-3}	6.11		

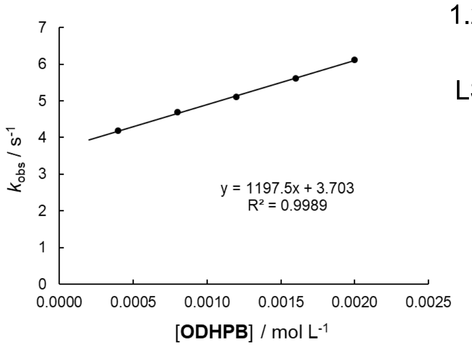

$[(pyr)_2CH^+]$ (mol L^{-1})	[ODHPB] (mol L^{-1})	k_{obs} (s^{-1})	λ = 620 nm	k_2 (L mol^{-1} s^{-1})
1.00×10^{-5}	4.00×10^{-4}	11.4		7.93×10^{3}
	8.00×10^{-4}	15.4		LSM-052
	1.20×10^{-3}	18.3		
	1.60×10^{-3}	21.5		
	2.00×10^{-3}	24.2		

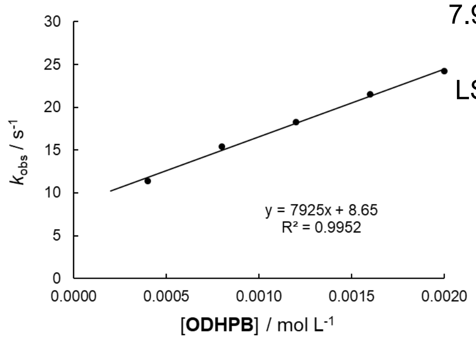

$[(dma)_2CH^+]$ (mol L^{-1})	[ODHPB] (mol L^{-1})	k_{obs} (s^{-1})	λ = 613 nm	k_2 (L mol^{-1} s^{-1})
1.00×10^{-5}	4.00×10^{-4}	13.8		2.79×10^{4}
	8.00×10^{-4}	25.1		LSM-051
	1.20×10^{-3}	35.2		
	1.60×10^{-3}	47.7		
	2.00×10^{-3}	58.3		

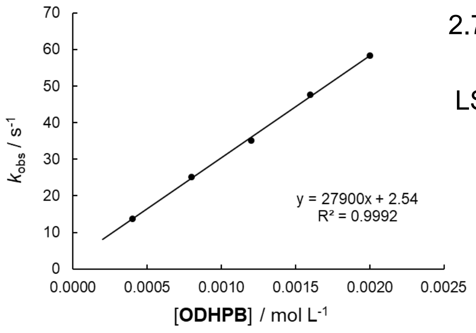

Table S14 continued: Kinetics of the reactions between ODHPB (**IU3**) and the benzhydrylium ions $(Ar)_2CH^+$ in CH_2Cl_2 at 20 °C.

$[(mpa)_2CH^+]$ (mol L^{-1})	[ODHPB] (mol L^{-1})	k_{obs} (s^{-1})	λ = 622 nm	k_2 (L mol^{-1} s^{-1})
8.00×10^{-6}	4.00×10^{-5}	5.31	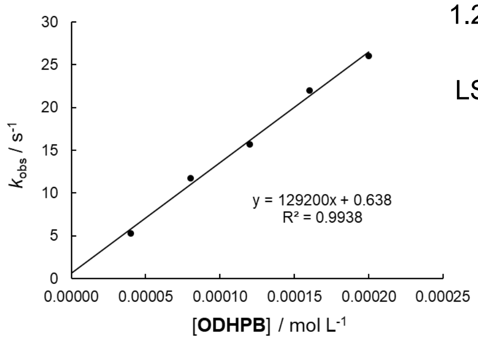	1.29×10^5
	8.00×10^{-5}	11.7		LSM-057
	1.20×10^{-4}	15.7		
	1.60×10^{-4}	22.0		
	2.00×10^{-4}	26.0		

$[(mor)_2CH^+]$ (mol L^{-1})	[ODHPB] (mol L^{-1})	k_{obs} (s^{-1})	λ = 620 nm	k_2 (L mol^{-1} s^{-1})
8.00×10^{-6}	4.00×10^{-5}	6.55	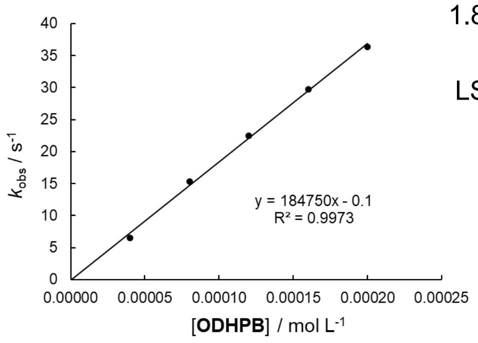	1.85×10^5
	8.00×10^{-5}	15.3		LSM-054
	1.20×10^{-4}	22.5		
	1.60×10^{-4}	29.7		
	2.00×10^{-4}	36.3		

$[(dpa)_2CH^+]$ (mol L^{-1})	[ODHPB] (mol L^{-1})	k_{obs} (s^{-1})	λ = 672 nm	k_2 (L mol^{-1} s^{-1})
8.00×10^{-6}	4.00×10^{-5}	27.6	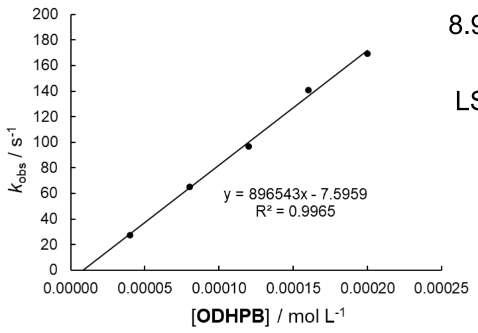	8.96×10^5
	8.00×10^{-5}	65.4		LSM-056
	1.20×10^{-4}	97.0		
	1.60×10^{-4}	141		
	2.00×10^{-4}	169		

Table S14 continued: Kinetics of the reactions between ODHPB (**IU3**) and the benzhydrylium ions $(Ar)_2CH^+$ in CH_2Cl_2 at 20 °C.

Reactivity Parameters for ODHPB in CH_2Cl_2 at 20 °C			
$(Ar)_2CH^+$	*E*	k_2 (L mol^{-1} s^{-1})	
$(ind)_2CH^+$	-8.76	1.20×10^3	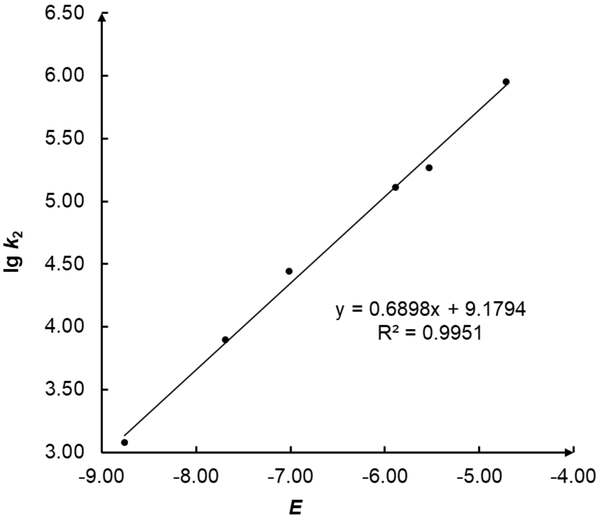
$(pyr)_2CH^+$	-7.69	7.93×10^3	
$(dma)_2CH^+$	-7.02	2.79×10^4	
$(mpa)_2CH^+$	-5.89	1.29×10^5	
$(mor)_2CH^+$	-5.53	1.85×10^5	
$(dpa)_2CH^+$	-4.72	8.96×10^5	
***N* = 13.31**			
**s_N = 0.69**			

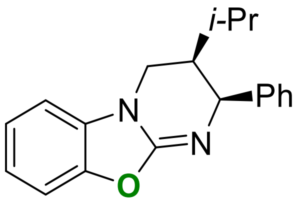

IU5
O*Hyper*BTM

Table S15: Kinetics of the reactions between O*Hyper*BTM (**IU5**) and the benzhydrylium ions $(Ar)_2CH^+$ in CH_2Cl_2 at 20 °C.

$[(dma)_2CH^+]$ (mol L^{-1})	[O*Hyper*BTM] (mol L^{-1})	k_{obs} (s^{-1})	λ = 613 nm	k_2 (L mol^{-1} s^{-1})
7.00×10^{-6}	2.10×10^{-4}	28.2	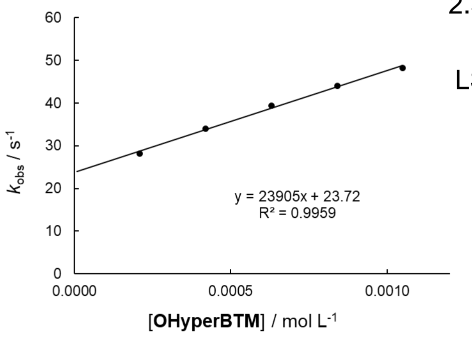	2.39×10^4
	4.20×10^{-4}	34.0		LSM-065
	6.30×10^{-4}	39.4		
	8.40×10^{-4}	44.0		
	1.05×10^{-3}	48.3		

$[(mpa)_2CH^+]$ (mol L^{-1})	[O*Hyper*BTM] (mol L^{-1})	k_{obs} (s^{-1})	λ = 622 nm	k_2 (L mol^{-1} s^{-1})
8.00×10^{-6}	4.00×10^{-5}	8.08	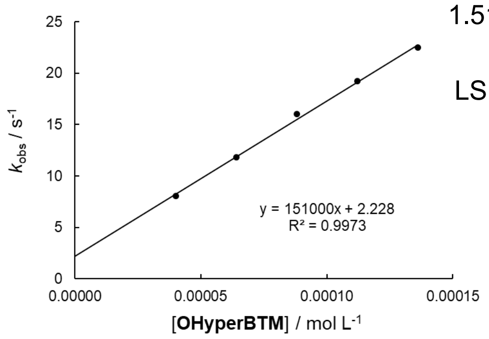	1.51×10^5
	6.40×10^{-5}	11.8		LSM-068
	8.80×10^{-5}	16.0		
	1.12×10^{-4}	19.2		
	1.36×10^{-4}	22.5		

$[(mor)_2CH^+]$ (mol L^{-1})	[O*Hyper*BTM] (mol L^{-1})	k_{obs} (s^{-1})	λ = 620 nm	k_2 (L mol^{-1} s^{-1})
8.00×10^{-6}	4.00×10^{-5}	8.67	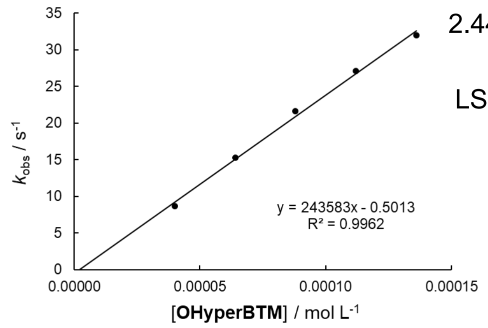	2.44×10^5
	6.40×10^{-5}	15.3		LSM-067
	8.80×10^{-5}	21.6		
	1.12×10^{-4}	27.1		
	1.36×10^{-4}	32.0		

Table S15 continued: Kinetics of the reactions between O*Hyper*BTM (**IU5**) and the benzhydrylium ions $(Ar)_2CH^+$ in CH_2Cl_2 at 20 °C.

$[(dpa)_2CH^+]$ (mol L^{-1})	[O*Hyper*BTM] (mol L^{-1})	k_{obs} (s^{-1})	λ = 674 nm	k_2 (L mol^{-1} s^{-1})
8.00×10^{-6}	4.00×10^{-5}	36.8	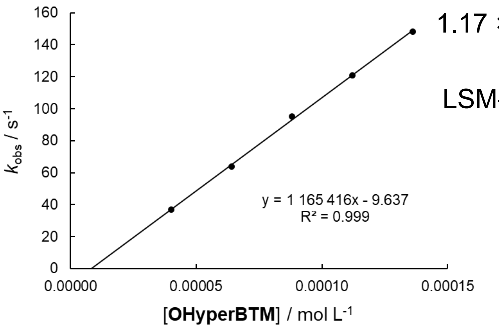	1.17×10^6
	6.40×10^{-5}	63.7		LSM-069
	8.80×10^{-5}	95.1		
	1.12×10^{-4}	121		
	1.36×10^{-4}	148		

Reactivity Parameters for O*Hyper*BTM (**IU5**) in CH_2Cl_2 at 20 °C

$(Ar)_2CH^+$	*E*	k_2 (L mol^{-1} s^{-1})
$(dma)_2CH^+$	-7.02	2.39×10^4
$(mpa)_2CH^+$	-5.89	1.51×10^5
$(mor)_2CH^+$	-5.53	2.44×10^5
$(dpa)_2CH^+$	-4.72	1.17×10^6

***N* = 13.01**

s_N = 0.73

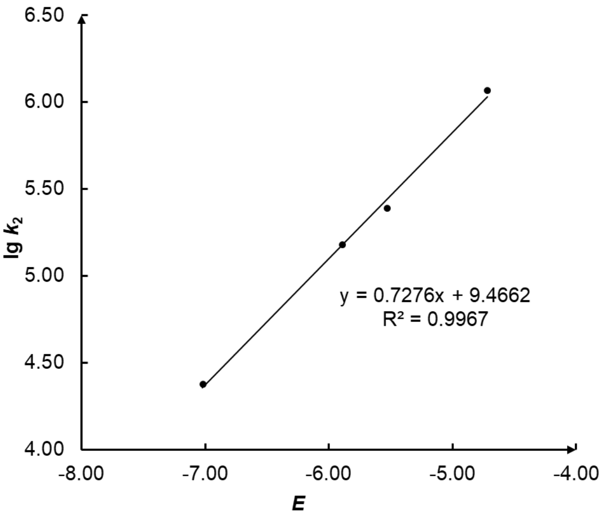

.

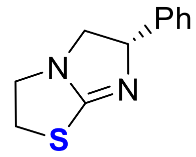

ITU1
TM

Table S16: Kinetics of the reactions between TM (**ITU1**) and the benzhydrylium ions $(Ar)_2CH^+$ in CH_2Cl_2 at 20 °C.

$[(ind)_2CH^+]$ (mol L^{-1})	[TM] (mol L^{-1})	k_{obs} (s^{-1})	λ = 625 nm	k_2 (L mol^{-1} s^{-1})
6.00×10^{-6}	6.00×10^{-5}	0.247	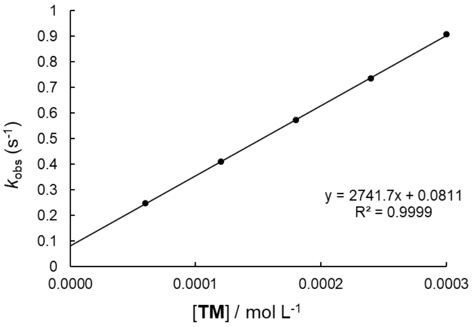	2.74×10^3
	1.20×10^{-4}	0.411		kk47m-63
	1.80×10^{-4}	0.572		
	2.40×10^{-4}	0.736		
	3.00×10^{-4}	0.907		

$[(pyr)_2CH^+]$ (mol L^{-1})	[TM] (mol L^{-1})	k_{obs} (s^{-1})	λ = 620 nm	k_2 (L mol^{-1} s^{-1})
6.00×10^{-6}	6.00×10^{-5}	1.00	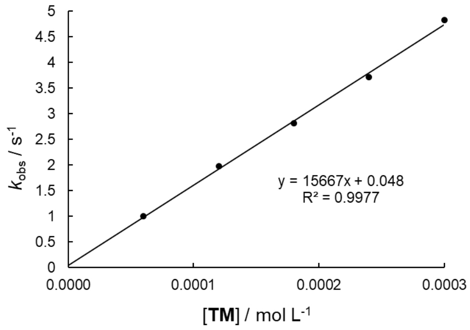	1.57×10^4
	1.20×10^{-4}	1.98		kk47m-62
	1.80×10^{-4}	2.81		
	2.40×10^{-4}	3.72		
	3.00×10^{-4}	4.83		

Table S16 continued: Kinetics of the reactions between TM (**ITU1**) and the benzhydrylium ions $(Ar)_2CH^+$ in CH_2Cl_2 at 20 °C.

$[(mpa)_2CH^+]$ (mol L^{-1})	[TM] (mol L^{-1})	k_{obs} (s^{-1})	λ = 622 nm	k_2 (L mol^{-1} s^{-1})
6.00×10^{-6}	3.00×10^{-5}	8.77	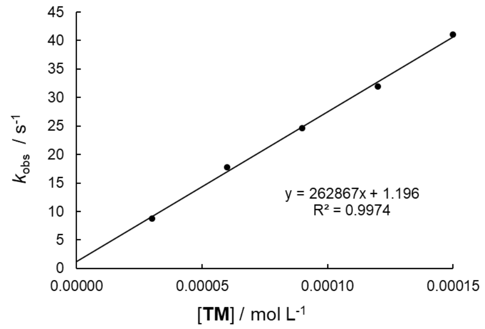	2.63×10^5
	6.00×10^{-5}	17.8		kk47m-64
	9.00×10^{-5}	24.6		
	1.20×10^{-4}	32.0		
	1.50×10^{-4}	41.1		

$[(mor)_2CH^+]$ (mol L^{-1})	[TM] (mol L^{-1})	k_{obs} (s^{-1})	λ = 620 nm	k_2 (L mol^{-1} s^{-1})
6.00×10^{-6}	3.00×10^{-5}	14.9	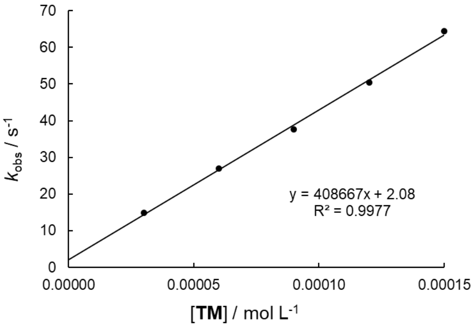	4.09×10^5
	6.00×10^{-5}	26.9		kk47m-65
	9.00×10^{-5}	37.6		
	1.20×10^{-4}	50.5		
	1.50×10^{-4}	64.4		

Reactivity Parameters for TM (**ITU1**) in CH_2Cl_2 at 20 °C

$(Ar)_2CH^+$	E	k_2 (L mol^{-1} s^{-1})
$(ind)_2CH^+$	-8.76	2.74×10^3
$(pyr)_2CH^+$	-7.69	1.57×10^4
$(mpa)_2CH^+$	-5.89	2.63×10^5
$(mor)_2CH^+$	-5.53	4.09×10^5

***N* = 13.86**

s_N = 0.68

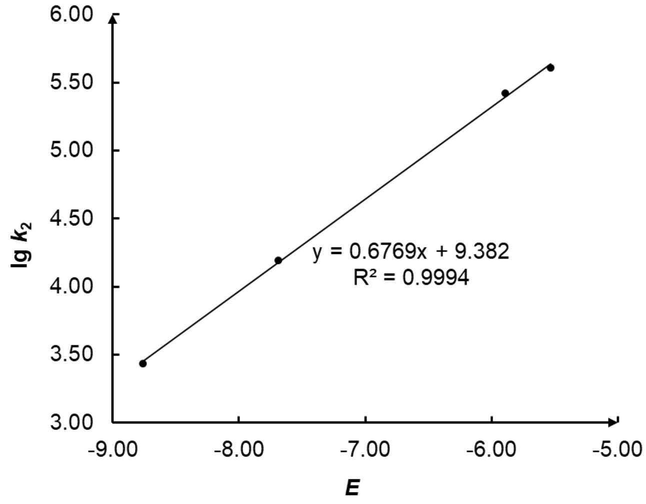

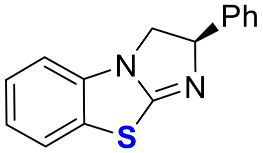

ITU2
BTM

Table S17: Kinetics of the reactions between BTM (**ITU2**) and the benzhydrylium ions $(Ar)_2CH^+$ in CH_2Cl_2 at 20 °C.

$[(jul)_2CH^+]$ (mol L^{-1})	[BTM] (mol L^{-1})	k_{obs} (s^{-1})	λ = 642 nm	k_2 (L mol^{-1} s^{-1})
5.00×10^{-6}	2.50×10^{-4}	2.66		3.88×10^{2}
	5.00×10^{-4}	2.72		LSM-139
	7.50×10^{-4}	2.81		
	1.00×10^{-3}	2.93		
	1.25×10^{-3}	3.04		

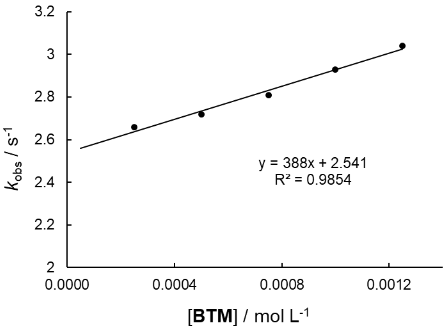

$[(pyr)_2CH^+]$ (mol L^{-1})	[BTM] (mol L^{-1})	k_{obs} (s^{-1})	λ = 620 nm	k_2 (L mol^{-1} s^{-1})
6.00×10^{-6}	6.00×10^{-5}	1.19		1.02×10^{4}
	1.20×10^{-4}	1.78		kk47m-57
	1.80×10^{-4}	2.37		
	2.40×10^{-4}	3.00		
	3.00×10^{-4}	3.65		

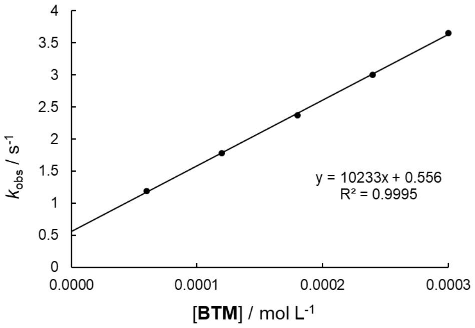

$[(dma)_2CH^+]$ (mol L^{-1})	[BTM] (mol L^{-1})	k_{obs} (s^{-1})	λ = 613 nm	k_2 (L mol^{-1} s^{-1})
6.00×10^{-6}	6.00×10^{-5}	2.13		3.41×10^{4}
	1.20×10^{-4}	4.04		kk47m-56
	1.80×10^{-4}	6.08		
	2.40×10^{-4}	8.15		
	3.00×10^{-4}	10.3		

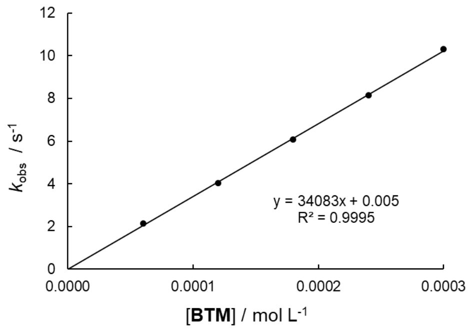

Table S17 continued: Kinetics of the reactions between BTM (**ITU2**) and the benzhydrylium ions $(Ar)_2CH^+$ in CH_2Cl_2 at 20 °C.

$[(mpa)_2CH^+]$ (mol L^{-1})	[BTM] (mol L^{-1})	k_{obs} (s^{-1})	λ = 622 nm	k_2 (L mol^{-1} s^{-1})
6.00 × 10^{-6}	3.00 × 10^{-5}	4.17	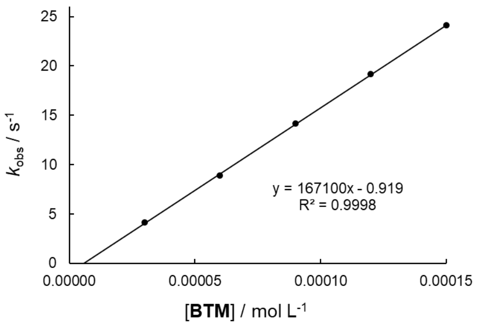	1.67 × 10^5
	6.00 × 10^{-5}	8.93		kk47m-60
	9.00 × 10^{-5}	14.2		
	1.20 × 10^{-4}	19.2		
	1.50 × 10^{-4}	24.1		

$[(mor)_2CH^+]$ (mol L^{-1})	[BTM] (mol L^{-1})	k_{obs} (s^{-1})	λ = 620 nm	k_2 (L mol^{-1} s^{-1})
6.00 × 10^{-6}	3.00 × 10^{-5}	6.49	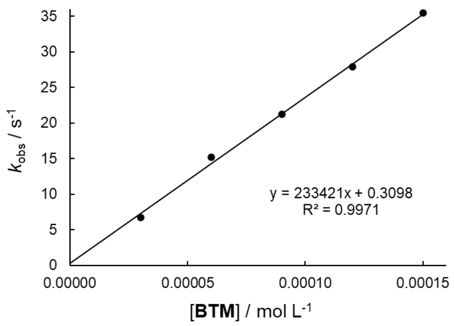	3.11 × 10^5
	6.00 × 10^{-5}	16.0		kk47m-61
	9.00 × 10^{-5}	25.5		
	1.20 × 10^{-4}	34.4		

Reactivity Parameters for BTM (**ITU2**) in CH_2Cl_2 at 20 °C

$(Ar)_2CH^+$	E	k_2 (L mol^{-1} s^{-1})
$(jul)_2CH^+$	-9.45	3.88 × 10^2
$(pyr)_2CH^+$	-7.69	1.02 × 10^4
$(dma)_2CH^+$	-7.02	3.41 × 10^4
$(mpa)_2CH^+$	-5.89	1.67 × 10^5
$(mor)_2CH^+$	-5.53	3.11 × 10^5

***N* = 13.06**

s_N = 0.73

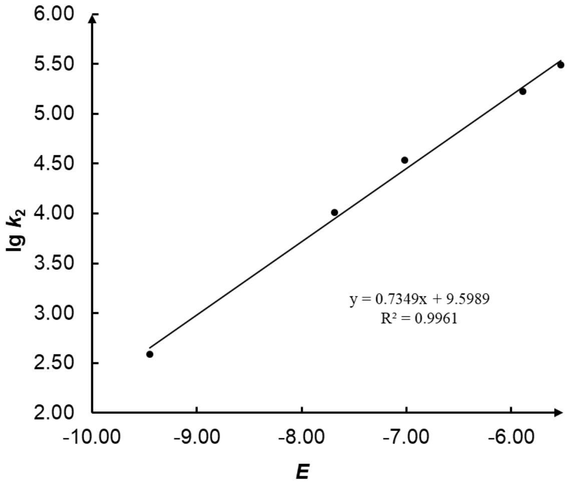

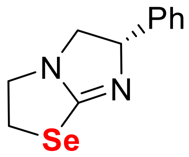

ISeU1
SeTM

Table S18: Kinetics of the reactions between SeTM (**ISeU1**) and the benzhydrylium ions $(Ar)_2CH^+$ in CH_2Cl_2 at 20 °C.

$[(pyr)_2CH^+]$ (mol L^{-1})	[SeTM] (mol L^{-1})	k_{obs} (s^{-1})	λ = 620 nm	k_2 (L mol^{-1} s^{-1})
8.00×10^{-6}	8.00×10^{-5}	2.36	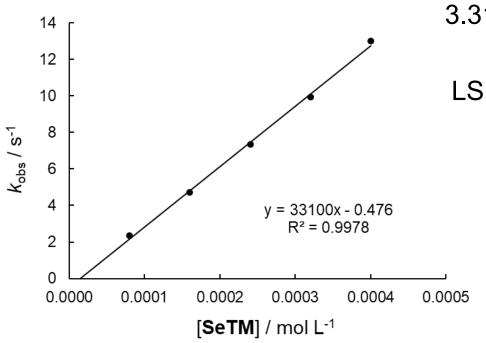	3.31×10^{4}
	1.60×10^{-4}	4.72		LSM-025
	2.40×10^{-4}	7.34		
	3.20×10^{-4}	9.92		
	4.00×10^{-4}	13.0		

$[(dma)_2CH^+]$ (mol L^{-1})	[SeTM] (mol L^{-1})	k_{obs} (s^{-1})	λ = 613 nm	k_2 (L mol^{-1} s^{-1})
8.00×10^{-6}	8.00×10^{-5}	6.29	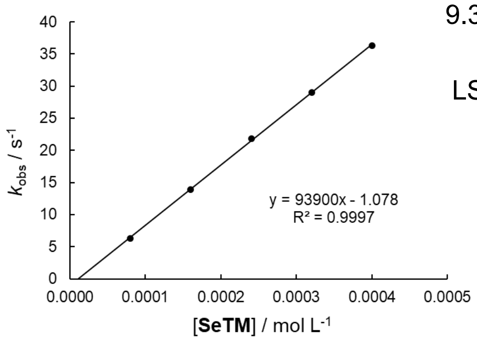	9.39×10^{4}
	1.60×10^{-4}	13.9		LSM-024
	2.40×10^{-4}	21.8		
	3.20×10^{-4}	29.0		
	4.00×10^{-4}	36.3		

$[(mpa)_2CH^+]$ (mol L^{-1})	[SeTM] (mol L^{-1})	k_{obs} (s^{-1})	λ = 620 nm	k_2 (L mol^{-1} s^{-1})
8.00×10^{-6}	4.00×10^{-5}	16.0	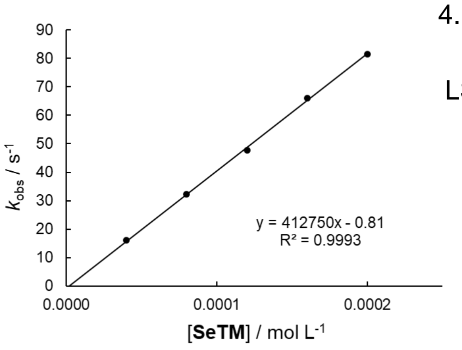	4.13×10^{5}
	8.00×10^{-5}	32.2		LSM-026
	1.20×10^{-4}	47.7		
	1.60×10^{-4}	66.1		
	2.00×10^{-4}	81.6		

Table S18 continued: Kinetics of the reactions between SeTM (**ISeU1**) and the benzhydrylium ions $(Ar)_2CH^+$ in CH_2Cl_2 at 20 °C.

$[(mor)_2CH^+]$ (mol L^{-1})	[SeTM] (mol L^{-1})	k_{obs} (s^{-1})	λ = 620 nm	k_2 (L mol^{-1} s^{-1})
8.00×10^{-6}	4.00×10^{-5}	25.2	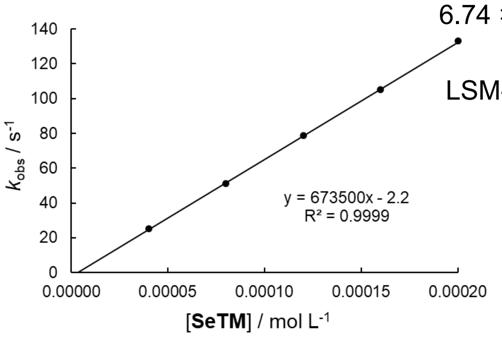	6.74×10^{5}<br>LSM-027
	8.00×10^{-5}	51.2		
	1.20×10^{-4}	78.7		
	1.60×10^{-4}	105		
	2.00×10^{-4}	133		

Reactivity Parameters for SeTM (**ISeU1**) in CH_2Cl_2 at 20 °C

$(Ar)_2CH^+$	E	k_2 (L mol^{-1} s^{-1})
$(pyr)_2CH^+$	-7.69	3.31×10^{4}
$(dma)_2CH^+$	-7.02	9.39×10^{4}
$(mpa)_2CH^+$	-5.89	4.13×10^{5}
$(mor)_2CH^+$	-5.53	6.74×10^{5}

***N* = 15.26**

s_N = 0.60

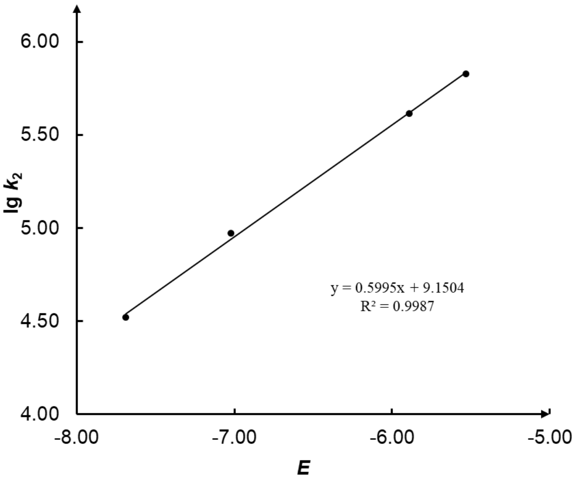

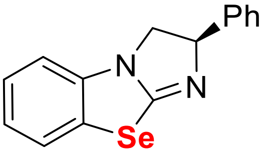

ISeU2
SeBTM

Table S19: Kinetics of the reactions between SeBTM (**ISeU2**) and the benzhydrylium ions $(Ar)_2CH^+$ in CH_2Cl_2 at 20 °C.

$[(thq)_2CH^+]$ (mol L^{-1})	[SeBTM] (mol L^{-1})	k_{obs} (s^{-1})	λ = 628 nm	k_2 (L mol^{-1} s^{-1})
8.00×10^{-6}	1.20×10^{-4}	0.932		5.30×10^{3}
	2.40×10^{-4}	1.52		LSM-023
	3.60×10^{-4}	2.15		
	4.80×10^{-4}	2.78		
	6.00×10^{-4}	3.48		

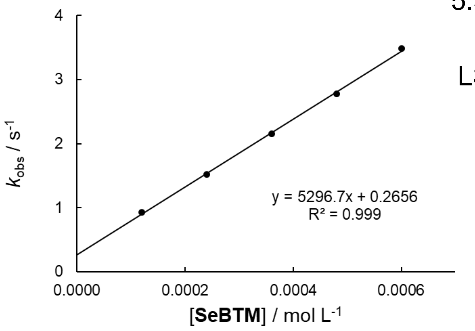

$[(pyr)_2CH^+]$ (mol L^{-1})	[SeBTM] (mol L^{-1})	k_{obs} (s^{-1})	λ = 620 nm	k_2 (L mol^{-1} s^{-1})
8.00×10^{-6}	8.00×10^{-5}	1.62		1.64×10^{4}
	1.60×10^{-4}	2.71		LSM-020
	2.40×10^{-4}	3.90		
	3.20×10^{-4}	5.37		
	4.00×10^{-4}	6.86		

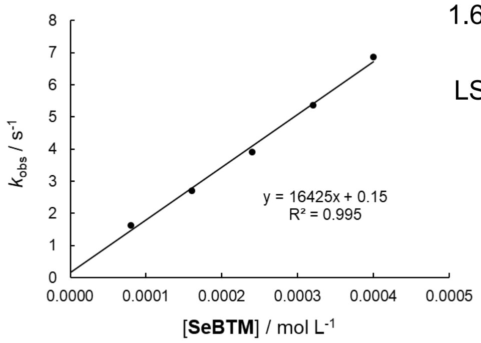

$[(dma)_2CH^+]$ (mol L^{-1})	[SeBTM] (mol L^{-1})	k_{obs} (s^{-1})	λ = 613 nm	k_2 (L mol^{-1} s^{-1})
8.00×10^{-6}	8.00×10^{-5}	4.07		5.14×10^{4}
	1.60×10^{-4}	7.32		LSM-019
	2.40×10^{-4}	11.4		
	3.20×10^{-4}	16.0		
	4.00×10^{-4}	20.3		

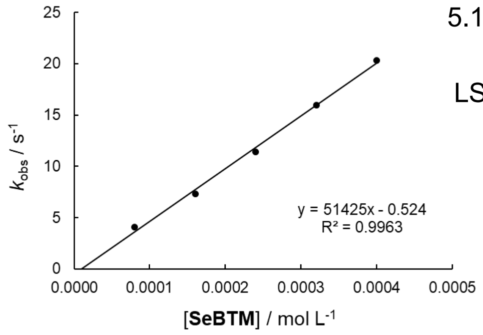

Table S19 continued: Kinetics of the reactions between SeBTM (**ISeU2**) and the benzhydrylium ions $(Ar)_2CH^+$ in CH_2Cl_2 at 20 °C.

$[(mpa)_2CH^+]$ (mol L^{-1})	[SeBTM] (mol L^{-1})	k_{obs} (s^{-1})	λ = 622 nm	k_2 (L mol^{-1} s^{-1})
8.00×10^{-6}	4.00×10^{-5}	5.84	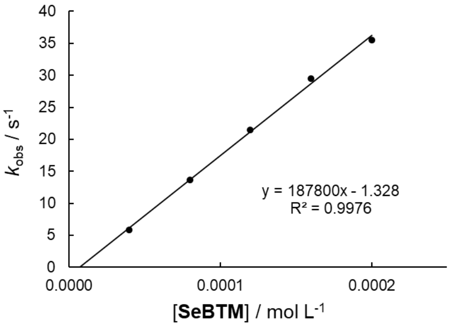	1.88×10^5
	8.00×10^{-5}	13.7		LSM-021
	1.20×10^{-4}	21.5		
	1.60×10^{-4}	29.5		
	2.00×10^{-4}	35.5		

$[(dpa)_2CH^+]$ (mol L^{-1})	[SeBTM] (mol L^{-1})	k_{obs} (s^{-1})	λ = 672 nm	k_2 (L mol^{-1} s^{-1})
8.00×10^{-6}	4.00×10^{-5}	29.1	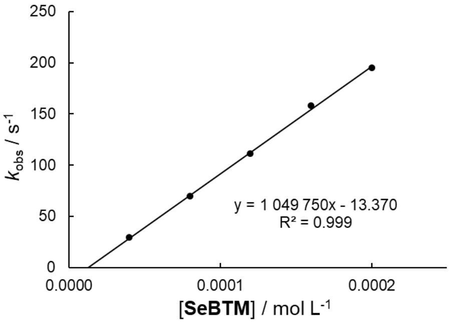	1.05×10^6
	8.00×10^{-5}	69.9		LSM-022
	1.20×10^{-4}	111		
	1.60×10^{-4}	158		
	2.00×10^{-4}	195		

Reactivity Parameters for SeBTM (**ISeU2**) in CH_2Cl_2 at 20 °C

$(Ar)_2CH^+$	E	k_2 (L mol^{-1} s^{-1})
$(thq)_2CH^+$	-8.22	5.30×10^3
$(pyr)_2CH^+$	-7.69	1.64×10^4
$(dma)_2CH^+$	-7.02	5.14×10^4
$(mpa)_2CH^+$	-5.89	1.88×10^5
$(dpa)_2CH^+$	-4.72	1.05×10^6

***N* = 14.27**

***s_N* = 0.63**

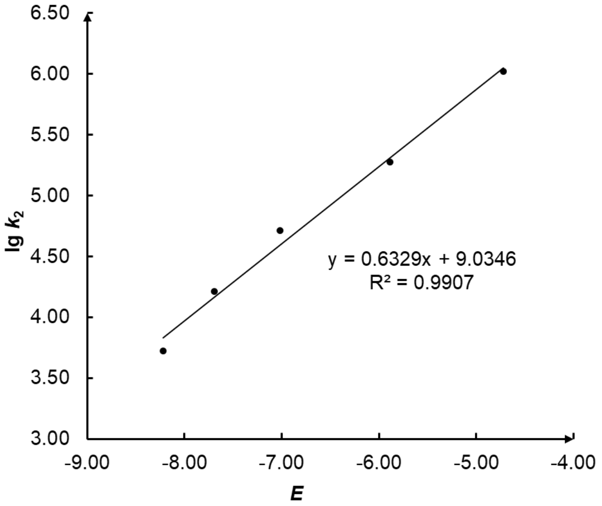

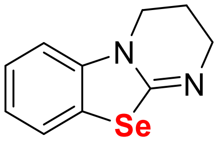

ISeU3
SeDHPB

Table S20: Kinetics of the reactions between SeDHPB (**ISeU3**) and the benzhydrylium ions $(Ar)_2CH^+$ in CH_2Cl_2 at 20 °C.

$[(lil)_2CH^+]$ (mol L^{-1})	[SeDHPB] (mol L^{-1})	k_{obs} (s^{-1})	λ = 639 nm	k_2 (L mol^{-1} s^{-1})
1.00×10^{-5}	2.00×10^{-4}	2.70	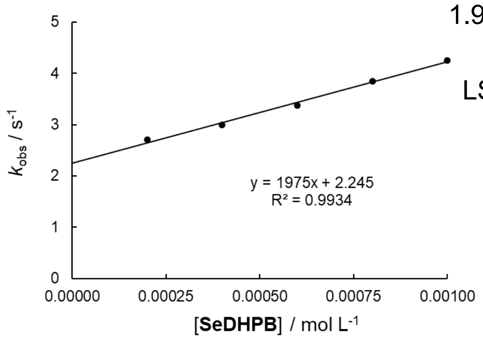	1.98×10^{3}
	4.00×10^{-4}	2.99		LSM-05
	6.00×10^{-4}	3.37		
	8.00×10^{-4}	3.84		
	1.00×10^{-3}	4.25		

$[(ind)_2CH^+]$ (mol L^{-1})	[SeDHPB] (mol L^{-1})	k_{obs} (s^{-1})	λ = 625 nm	k_2 (L mol^{-1} s^{-1})
2.00×10^{-5}	2.00×10^{-4}	3.97	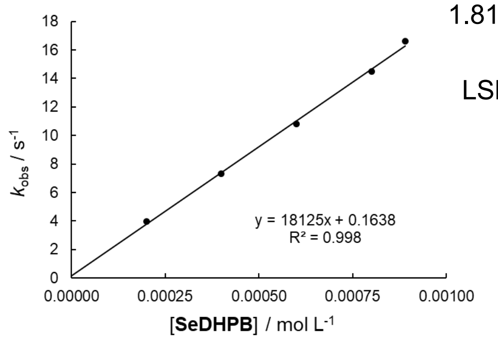	1.81×10^{4}
	4.00×10^{-4}	7.33		LSM-03
	6.00×10^{-4}	10.8		
	8.00×10^{-4}	14.5		
	8.90×10^{-4}	16.6		

$[(thq)_2CH^+]$ (mol L^{-1})	[SeDHPB] (mol L^{-1})	k_{obs} (s^{-1})	λ = 628 nm	k_2 (L mol^{-1} s^{-1})
1.00×10^{-5}	2.00×10^{-4}	7.76	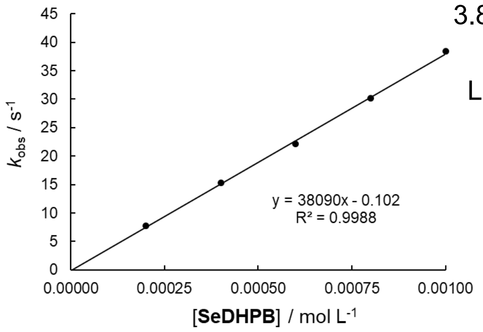	3.81×10^{4}
	4.00×10^{-4}	15.3		LSM-07
	6.00×10^{-4}	22.1		
	8.00×10^{-4}	30.2		
	1.00×10^{-3}	38.4		

Table S20 continued: Kinetics of the reactions between SeDHPB (**ISeU3**) and the benzhydrylium ions $(Ar)_2CH^+$ in CH_2Cl_2 at 20 °C.

$[(pyr)_2CH^+]$ (mol L^{-1})	[SeDHPB] (mol L^{-1})	k_{obs} (s^{-1})	λ = 620 nm	k_2 (L mol^{-1} s^{-1})
2.00×10^{-5}	2.00×10^{-4}	20.1		1.07×10^{5}
	4.00×10^{-4}	39.1		LSM-02
	6.00×10^{-4}	60.6		
	8.00×10^{-4}	84.3		

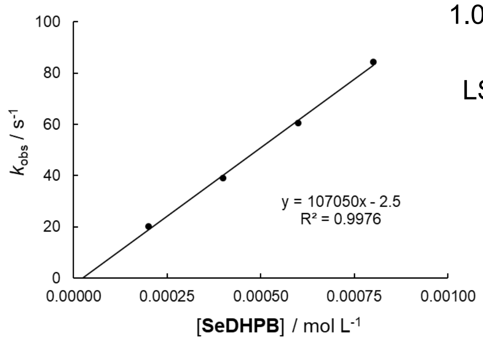

$[(dma)_2CH^+]$ (mol L^{-1})	[SeDHPB] (mol L^{-1})	k_{obs} (s^{-1})	λ = 613 nm	k_2 (L mol^{-1} s^{-1})
2.00×10^{-5}	1.00×10^{-4}	14.3		1.94×10^{5}
	2.00×10^{-4}	37.0		LSM-01
	3.00×10^{-4}	58.6		
	4.00×10^{-4}	79.5		
	6.00×10^{-4}	111		

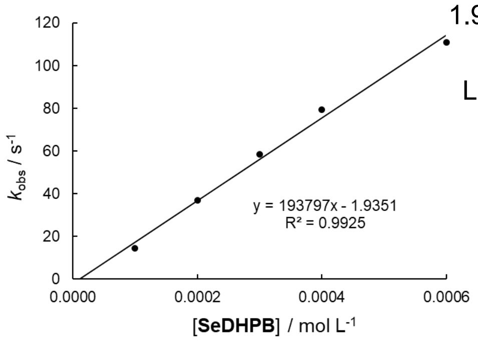

$[(mpa)_2CH^+]$ (mol L^{-1})	[SeDHPB] (mol L^{-1})	k_{obs} (s^{-1})	λ = 622 nm	k_2 (L mol^{-1} s^{-1})
7.00×10^{-6}	3.50×10^{-5}	32.9		1.16×10^{6}
	5.60×10^{-5}	59.8		LSM-04
	7.70×10^{-5}	83.0		
	9.80×10^{-5}	109		
	1.19×10^{-4}	130		

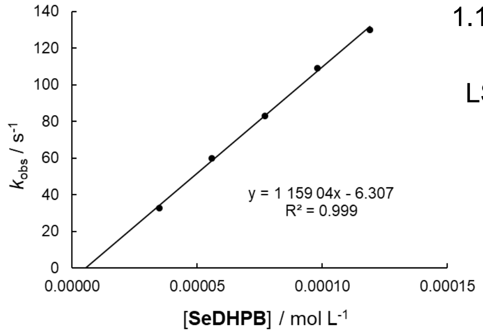

Table S20 continued: Kinetics of the reactions between SeDHPB (**ISeU3**) and the benzhydrylium ions $(Ar)_2CH^+$ in CH_2Cl_2 at 20 °C.

Reactivity Parameters for SeHDHPB (**ISeU3**) in CH_2Cl_2 at 20 °C		
$(Ar)_2CH^+$	E	k_2 (L mol^{-1} s^{-1})
$(lil)_2CH^+$	-10.04	1.98×10^3
$(ind)_2CH^+$	-8.76	1.81×10^4
$(thq)_2CH^+$	-8.22	3.81×10^4
$(pyr)_2CH^+$	-7.69	1.07×10^5
$(dma)_2CH^+$	-7.02	1.94×10^5
$(mpa)_2CH^+$	-5.89	1.16×10^6
***N* = 15.16**		
**s_N = 0.66**		

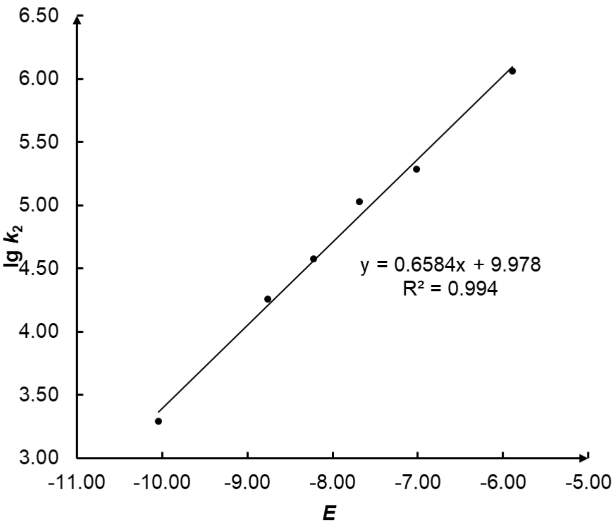

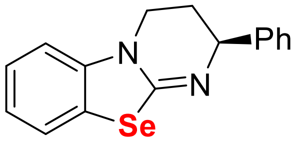

ISeU4
SeHBTM

Table S21: Kinetics of the reactions between SeHBTM (**ISeU4**) and the benzhydrylium ions $(Ar)_2CH^+$ in CH_2Cl_2 at 20 °C.

$[(ind)_2CH^+]$ (mol L^{-1})	[SeHBTM] (mol L^{-1})	k_{obs} (s^{-1})	λ = 625 nm	k_2 (L mol^{-1} s^{-1})
7.00×10^{-6}	1.40×10^{-4}	13.5	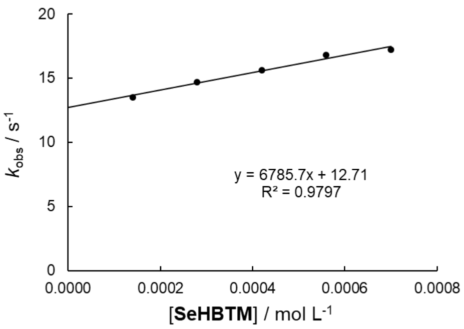	6.79×10^{3}
	2.80×10^{-4}	14.7		LSM-018
	4.20×10^{-4}	15.6		
	5.60×10^{-4}	16.8		
	7.00×10^{-4}	17.2		

$[(thq)_2CH^+]$ (mol L^{-1})	[SeHBTM] (mol L^{-1})	k_{obs} (s^{-1})	λ = 628 nm	k_2 (L mol^{-1} s^{-1})
7.00×10^{-6}	2.80×10^{-4}	15.3	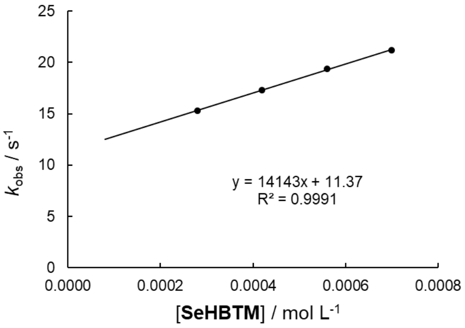	1.41×10^{4}
	4.20×10^{-4}	17.3		LSM-017
	5.60×10^{-4}	19.4		
	7.00×10^{-4}	21.2		

$[(pyr)_2CH^+]$ (mol L^{-1})	[SeHBTM] (mol L^{-1})	k_{obs} (s^{-1})	λ = 620 nm	k_2 (L mol^{-1} s^{-1})
8.00×10^{-6}	8.00×10^{-5}	18.0	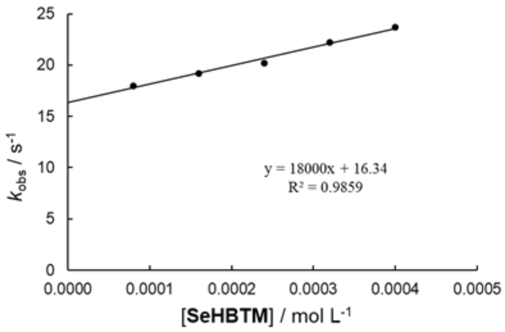	1.80×10^{4}
	1.60×10^{-4}	19.2		LSM-016
	2.40×10^{-4}	20.2		
	3.20×10^{-4}	22.2		
	4.00×10^{-4}	23.7		

Table S21 continued: Kinetics of the reactions between SeHBTM (**ISeU4**) and the benzhydrylium ions $(Ar)_2CH^+$ in CH_2Cl_2 at 20 °C.

$[(dma)_2CH^+]$ (mol L^{-1})	[SeHBTM] (mol L^{-1})	k_{obs} (s^{-1})	λ = 613 nm	k_2 (L mol^{-1} s^{-1})
8.00×10^{-6}	8.00×10^{-5}	13.0	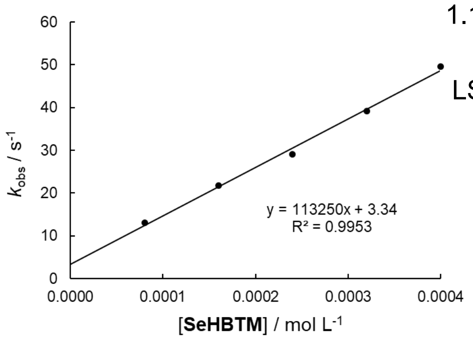	1.13×10^{5}
	1.60×10^{-4}	21.8		LSM-015
	2.40×10^{-4}	29.0		
	3.20×10^{-4}	39.2		
	4.00×10^{-4}	49.6		

$[(mpa)_2CH^+]$ (mol L^{-1})	[SeHBTM] (mol L^{-1})	k_{obs} (s^{-1})	λ = 622 nm	k_2 (L mol^{-1} s^{-1})
7.00×10^{-6}	3.50×10^{-5}	17.2	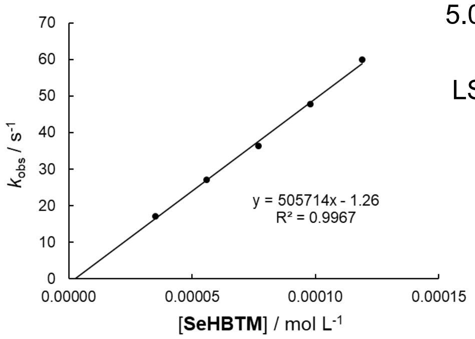	5.06×10^{5}
	5.60×10^{-5}	27.1		LSM-013
	7.70×10^{-5}	36.4		
	9.80×10^{-5}	47.7		
	1.19×10^{-4}	60.0		

$[(mor)_2CH^+]$ (mol L^{-1})	[SeHBTM] (mol L^{-1})	k_{obs} (s^{-1})	λ = 620 nm	k_2 (L mol^{-1} s^{-1})
7.00×10^{-6}	3.50×10^{-5}	23.2	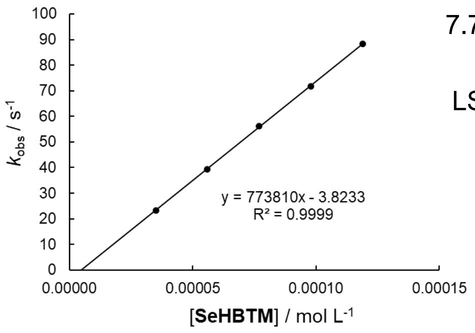	7.74×10^{5}
	5.60×10^{-5}	39.4		LSM-014
	7.70×10^{-5}	56.2		
	9.80×10^{-5}	71.7		
	1.19×10^{-4}	88.3		

Table S21 continued: Kinetics of the reactions between SeHBTM (**ISeU4**) and the benzhydrylium ions $(Ar)_2CH^+$ in CH_2Cl_2 at 20 °C.

Reactivity Parameters for SeHBTM (**ISeU4**) in CH_2Cl_2 at 20 °C		
$(Ar)_2CH^+$	*E*	k_2 (L mol^{-1} s^{-1})
$(ind)_2CH^+$	-8.76	6.79×10^3
$(thq)_2CH^+$	-8.22	1.41×10^4
$(pyr)_2CH^+$	-7.69	1.80×10^4
$(dma)_2CH^+$	-7.02	1.13×10^5
$(mpa)_2CH^+$	-5.89	5.06×10^5
$(mor)_2CH^+$	-5.53	7.74×10^5
***N* = 14.42**		
**s_N = 0.67**		

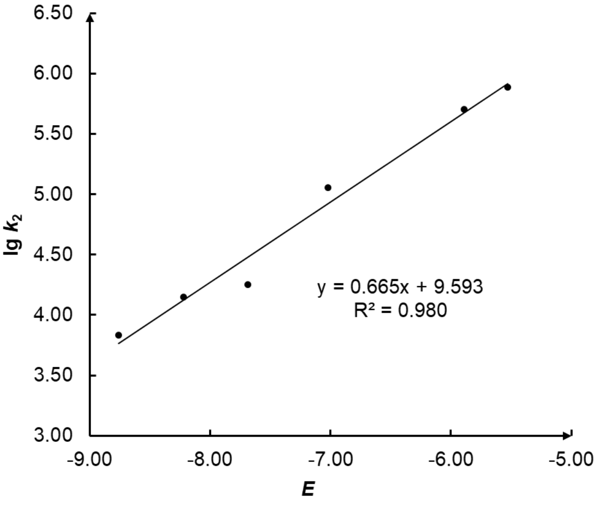

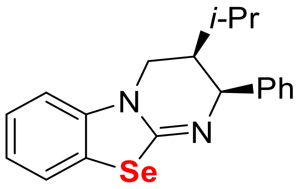

ISeU5
Se*Hyper*BTM

Table S22: Kinetics of the reactions between Se*Hyper*BTM (**ISeU5**) and the benzhydrylium ions $(Ar)_2CH^+$ in CH_2Cl_2 at 20 °C.

$[(thq)_2CH^+]$ (mol L^{-1})	[SeHyperBTM] (mol L^{-1})	k_{obs} (s^{-1})	λ = 628 nm	k_2 (L mol^{-1} s^{-1})
8.00×10^{-6}	1.20×10^{-4}	12.5	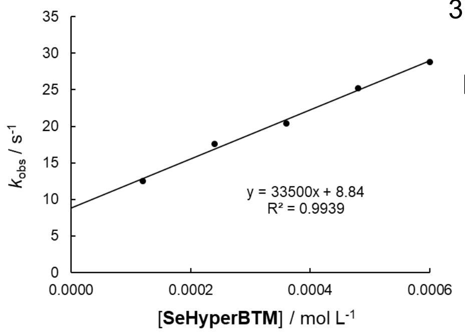	3.35×10^{4}
	2.40×10^{-4}	17.6		LSM-08
	3.60×10^{-4}	20.4		
	4.80×10^{-4}	25.2		
	6.00×10^{-4}	28.8		

$[(pyr)_2CH^+]$ (mol L^{-1})	[SeHyperBTM] (mol L^{-1})	k_{obs} (s^{-1})	λ = 620 nm	k_2 (L mol^{-1} s^{-1})
8.00×10^{-6}	8.00×10^{-5}	14.5	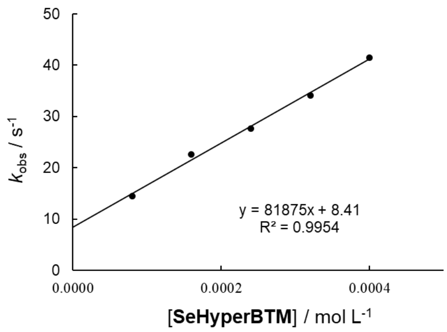	8.19×10^{4}
	1.60×10^{-4}	22.6		LSM-010
	2.40×10^{-4}	27.6		
	3.20×10^{-4}	34.1		
	4.00×10^{-4}	41.5		

$[(dma)_2CH^+]$ (mol L^{-1})	[SeHyperBTM] (mol L^{-1})	k_{obs} (s^{-1})	λ = 613 nm	k_2 (L mol^{-1} s^{-1})
8.00×10^{-6}	8.00×10^{-5}	23.5	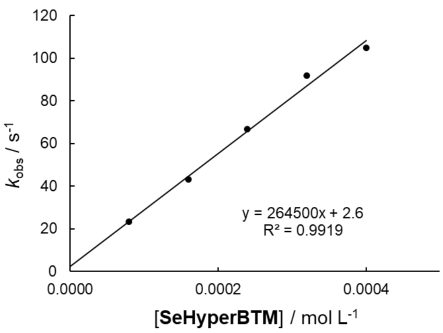	2.65×10^{5}
	1.60×10^{-4}	43.3		LSM-09
	2.40×10^{-4}	66.7		
	3.20×10^{-4}	91.9		
	4.00×10^{-4}	105		

Table S22 continued: Kinetics of the reactions between SeHyperBTM (**ISeU5**) and the benzhydrylium ions $(Ar)_2CH^+$ in CH_2Cl_2 at 20 °C.

$[(mpa)_2CH^+]$ (mol L^{-1})	[SeHyperBTM] (mol L^{-1})	k_{obs} (s^{-1})	λ = 622 nm	k_2 (L mol^{-1} s^{-1})
7.00×10^{-6}	3.50×10^{-5}	24.0		8.47×10^{5}
	5.60×10^{-5}	37.5		LSM-011
	7.70×10^{-5}	53.3		
	9.80×10^{-5}	75.0		
	1.19×10^{-4}	94.2		

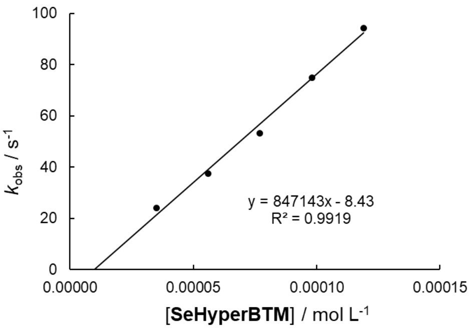

$[(mor)_2CH^+]$ (mol L^{-1})	[SeHyperBTM] (mol L^{-1})	k_{obs} (s^{-1})	λ = 620 nm	k_2 (L mol^{-1} s^{-1})
7.00×10^{-6}	3.50×10^{-5}	33.7		1.35×10^{6}
	5.60×10^{-5}	57.4		LSM-012
	7.70×10^{-5}	86.9		
	9.80×10^{-5}	115		
	1.19×10^{-4}	147		

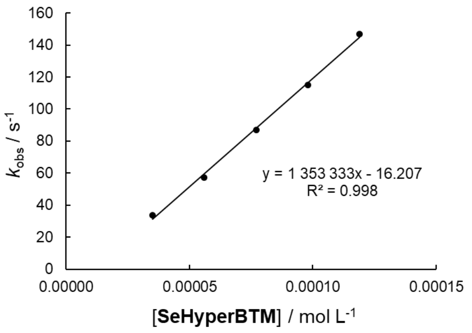

Reactivity Parameters for Se*Hyper*BTM (**ISeU5**) in CH_2Cl_2 at 20 °C

$(Ar)_2CH^+$	E	k (L mol^{-1} s^{-1})
$(thq)_2CH^+$	-8.22	3.35×10^{4}
$(pyr)_2CH^+$	-7.69	8.19×10^{4}
$(dma)_2CH^+$	-7.02	2.65×10^{5}
$(mpa)_2CH^+$	-5.89	8.47×10^{5}
$(mor)_2CH^+$	-5.53	1.35×10^{6}

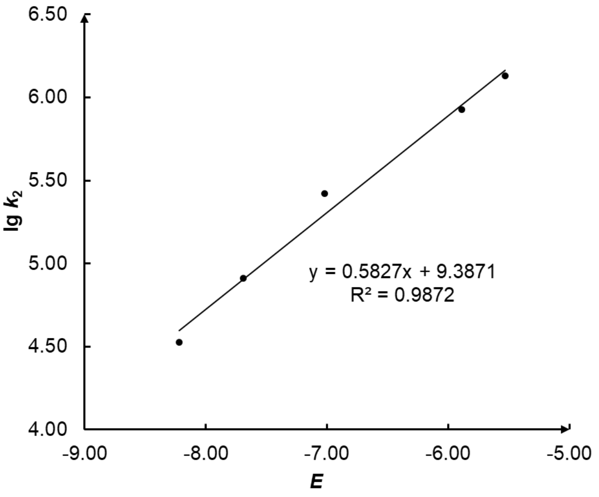

***N* = 16.11**

s_N = 0.58

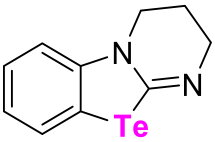

ITeU3
TeDHPB

Table S23: Kinetics of the reactions between TeDHPB (**ITeU3**) and the benzhydrylium ions $(Ar)_2CH^+$ in CH_2Cl_2 at 20 °C.

$[(lil)_2CH^+]$ (mol L^{-1})	[TeDHPB] (mol L^{-1})	k_{obs} (s^{-1})	λ = 639 nm	k_2 (L mol^{-1} s^{-1})
8.00×10^{-6}	8.00×10^{-5}	1.99	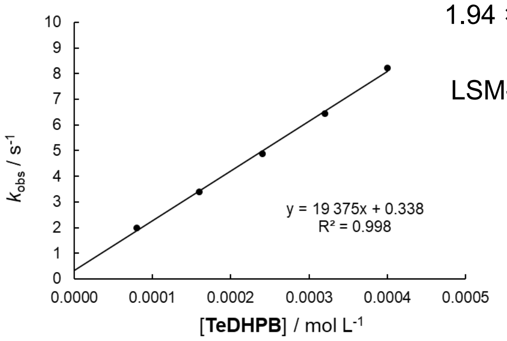	1.94×10^{4}
	1.60×10^{-4}	3.40		LSM-147
	2.40×10^{-4}	4.89		
	3.20×10^{-4}	6.44		
	4.00×10^{-4}	8.22		

$[(ind)_2CH^+]$ (mol L^{-1})	[TeDHPB] (mol L^{-1})	k_{obs} (s^{-1})	λ = 625 nm	k_2 (L mol^{-1} s^{-1})
8.00×10^{-6}	8.00×10^{-5}	9.95	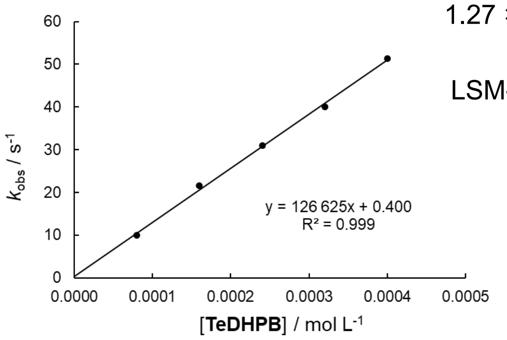	1.27×10^{5}
	1.60×10^{-4}	21.6		LSM-146
	2.40×10^{-4}	31.0		
	3.20×10^{-4}	40.0		
	4.00×10^{-4}	51.4		

$[(pyr)_2CH^+]$ (mol L^{-1})	[TeDHPB] (mol L^{-1})	k_{obs} (s^{-1})	λ = 620 nm	k_2 (L mol^{-1} s^{-1})
7.00×10^{-6}	3.50×10^{-5}	23.6	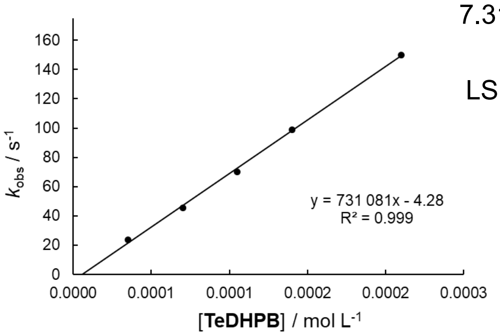	7.31×10^{5}
	7.00×10^{-5}	45.3		LSM-145
	1.05×10^{-4}	70.3		
	1.40×10^{-4}	98.8		
	2.10×10^{-4}	150		

Table S23 continued: Kinetics of the reactions between TeDHPB (**ITeU3**) and the benzhydrylium ions $(Ar)_2CH^+$ in CH_2Cl_2 at 20 °C.

$[(dma)_2CH^+]$ (mol L^{-1})	[TeDHPB] (mol L^{-1})	k_{obs} (s^{-1})	λ = 613 nm	k_2 (L mol^{-1} s^{-1})
7.00×10^{-6}	3.50×10^{-5}	48.1		1.67×10^{6}
	7.00×10^{-5}	109		LSM-144
	1.05×10^{-4}	165		
	1.40×10^{-4}	224		
	2.10×10^{-4}	341		

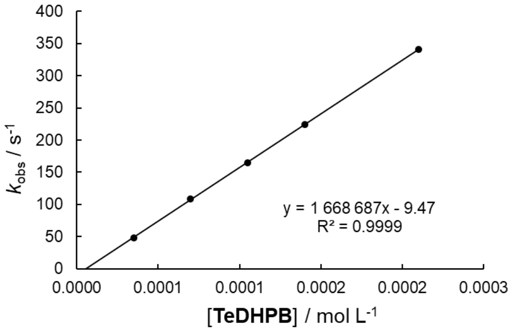

Reactivity Parameters for TeDHPB (**ITeU3**) in CH_2Cl_2 at 20 °C

$(Ar)_2CH^+$	E	k_2 (L mol^{-1} s^{-1})
$(lil)_2CH^+$	-10.04	1.94×10^{4}
$(ind)_2CH^+$	-8.76	1.27×10^{5}
$(pyr)_2CH^+$	-7.69	7.31×10^{5}
$(dma)_2CH^+$	-7.02	1.67×10^{6}

***N* = 16.63**

s_N = 0.65

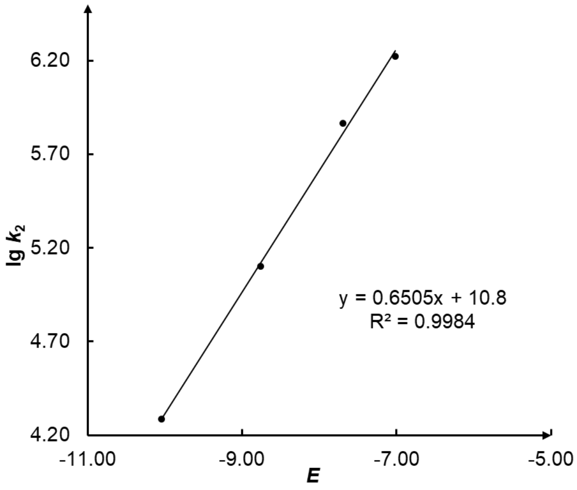

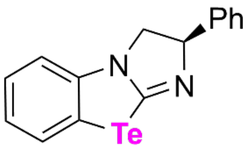

ITeU2
TeBTM

Table S24: Kinetics of the reactions between TeBTM (**ITeU2**) and the benzhydrylium ions $(Ar)_2CH^+$ in CH_2Cl_2 at 20 °C.

$[(lil)_2CH^+]$ (mol L^{-1})	[TeDHPB] (mol L^{-1})	k_{obs} (s^{-1})	λ = 639 nm	k_2 (L mol^{-1} s^{-1})
8.00×10^{-6}	1.60×10^{-4}	0.171	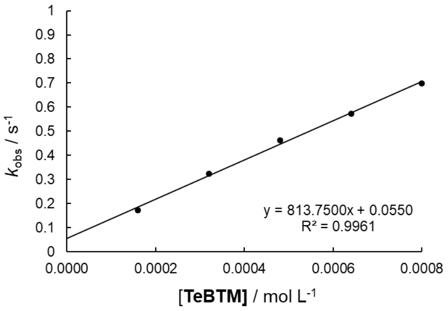	8.14×10^2
	3.20×10^{-4}	0.324		LSM-174
	4.80×10^{-4}	0.463		
	6.40×10^{-4}	0.572		
	8.00×10^{-4}	0.698		

$[(ind)_2CH^+]$ (mol L^{-1})	[TeDHPB] (mol L^{-1})	k_{obs} (s^{-1})	λ = 625 nm	k_2 (L mol^{-1} s^{-1})
8.00×10^{-6}	1.60×10^{-4}	1.15	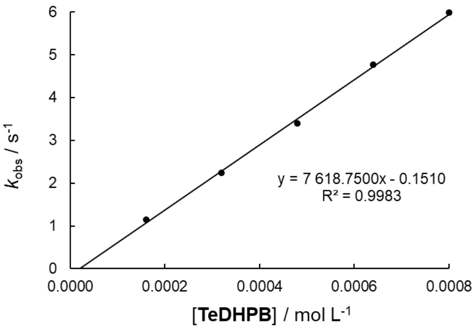	7.62×10^3
	3.20×10^{-4}	2.24		LSM-173
	4.80×10^{-4}	3.39		
	6.40×10^{-4}	4.77		
	8.00×10^{-4}	5.98		

$[(pyr)_2CH^+]$ (mol L^{-1})	[TeDHPB] (mol L^{-1})	k_{obs} (s^{-1})	λ = 620 nm	k_2 (L mol^{-1} s^{-1})
7.00×10^{-6}	7.00×10^{-5}	3.05	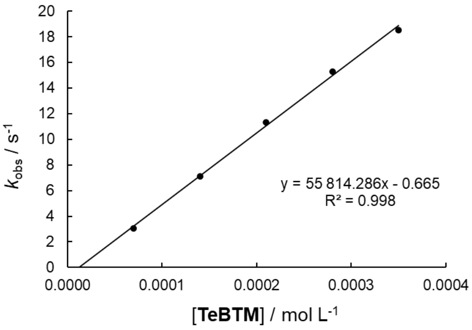	5.58×10^4
	1.40×10^{-4}	7.13		LSM-172
	2.10×10^{-4}	11.3		
	2.80×10^{-4}	15.3		
	3.50×10^{-4}	18.5		

Table S24 continued: Kinetics of the reactions between TeBTM (**ITeU2**) and the benzhydrylium ions $(Ar)_2CH^+$ in CH_2Cl_2 at 20 °C.

$[(dma)_2CH^+]$ (mol L^{-1})	[TeDHPB] (mol L^{-1})	k_{obs} (s^{-1})	λ = 613 nm	k_2 (L mol^{-1} s^{-1})
7.00×10^{-6}	7.00×10^{-5}	7.70	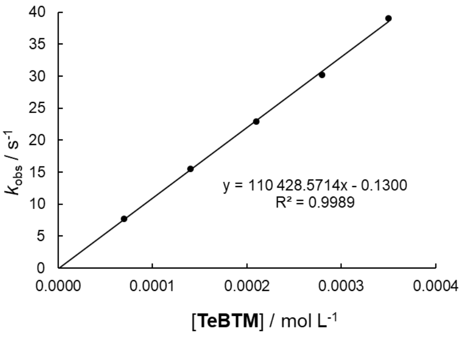	1.10×10^5
	1.40×10^{-4}	15.5		LSM-171
	2.10×10^{-4}	22.9		
	2.80×10^{-4}	30.2		
	3.50×10^{-4}	39.0		
$[(mor)_2CH^+]$ (mol L^{-1})	[TeDHPB] (mol L^{-1})	k_{obs} (s^{-1})	λ = 620 nm	k_2 (L mol^{-1} s^{-1})
7.00×10^{-6}	7.00×10^{-5}	39.1	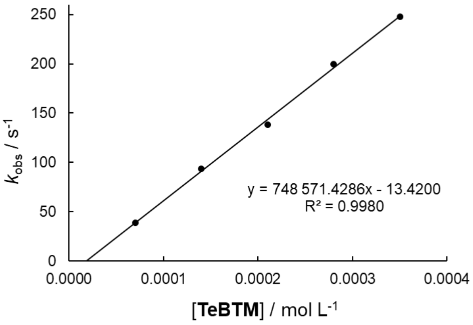	7.49×10^5
	1.40×10^{-4}	93.8		LSM-180
	2.10×10^{-4}	138		
	2.80×10^{-4}	200		
	3.50×10^{-4}	248		

Reactivity Parameters for TeBTM (**ITeU2**) in CH_2Cl_2 at 20 °C

$(Ar)_2CH^+$	E	k_2 (L mol^{-1} s^{-1})
$(lil)_2CH^+$	-10.04	8.14×10^2
$(ind)_2CH^+$	-8.76	7.62×10^3
$(pyr)_2CH^+$	-7.69	5.58×10^4
$(dma)_2CH^+$	-7.02	1.10×10^5
$(mor)_2CH^+$	-5.53	7.49×10^5

***N* = 14.62**

***s*$_N$ = 0.66**

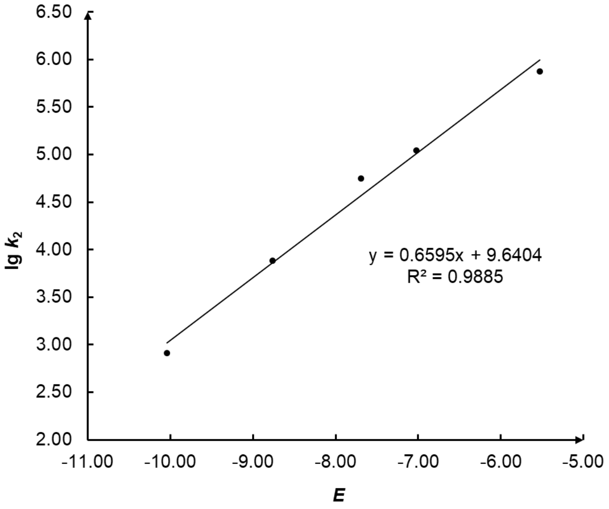

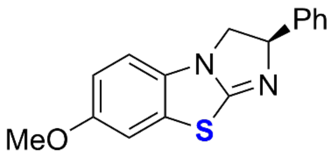

MeO-ITU2
7-AnTM

Table S25: Kinetics of the reaction between 7-MeO-BTM (7-AnTM; **MeO-ITU2**) and the benzhydrylium ions $(Ar)_2CH^+$ in CH_2Cl_2.at 20 °C.

$[(jul)_2CH^+]$ (mol L^{-1})	[7-AnTM] (mol L^{-1})	k_{obs} (s^{-1})	λ = 613 nm	k (M^{-1} s^{-1})
$5.00 \cdot 10^{-6}$	2.50×10^{-4}	1.52	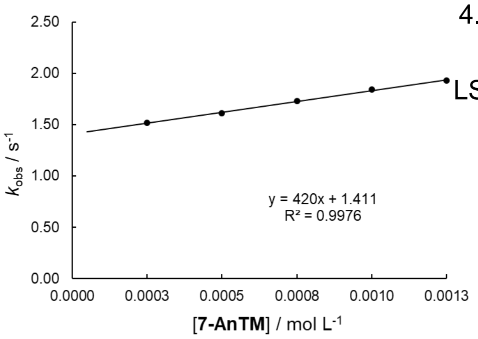	4.20×10^{2}
	5.00×10^{-4}	1.61		LS390-136
	7.50×10^{-4}	1.73		
	1.00×10^{-3}	1.84		
	1.25×10^{-3}	1.93		

$[(pyr)_2CH^+]$ (mol L^{-1})	[7-AnTM] (mol L^{-1})	k_{obs} (s^{-1})	λ = 620 nm	k (M^{-1} s^{-1})
$6.00 \cdot 10^{-6}$	6.00×10^{-5}	1.37	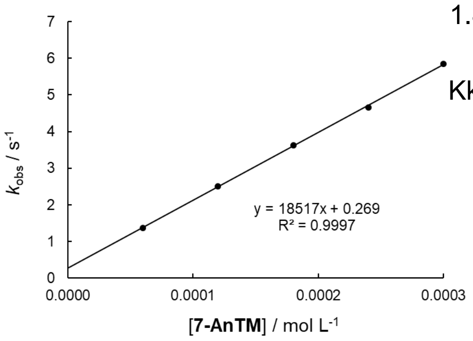	1.85×10^{4}
	1.20×10^{-4}	2.51		Kk47m-55
	1.80×10^{-4}	3.62		
	2.40×10^{-4}	4.66		
	3.00×10^{-4}	5.85		

$[(dma)_2CH^+]$ (mol L^{-1})	[7-AnTM] (mol L^{-1})	k_{obs} (s^{-1})	λ = 613 nm	k (M^{-1} s^{-1})
$6.00 \cdot 10^{-6}$	6.00×10^{-5}	3.52	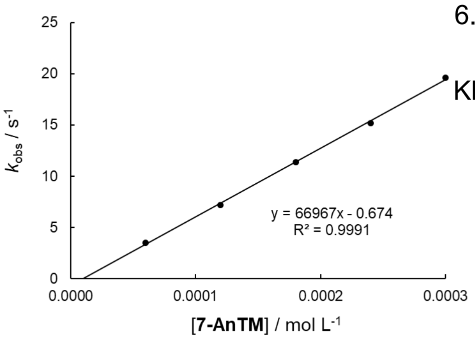	6.70×10^{4}
	1.20×10^{-4}	7.18		Kk47m-54
	1.80×10^{-4}	11.4		
	2.40×10^{-4}	15.2		
	3.00×10^{-4}	19.6		

Table S25 continued: Kinetics of the reaction between 7-MeO-BTM (7-AnTM; **MeO-ITU2**) and the benzhydrylium ions $(Ar)_2CH^+$ in CH_2Cl_2 at 20 °C.

$[(mpa)_2CH^+]$ (mol L^{-1})	[7-AnTM] (mol L^{-1})	k_{obs} (s^{-1})	λ = 622 nm	k (M^{-1} s^{-1})
$6.00 \cdot 10^{-6}$	3.00×10^{-5}	6.53	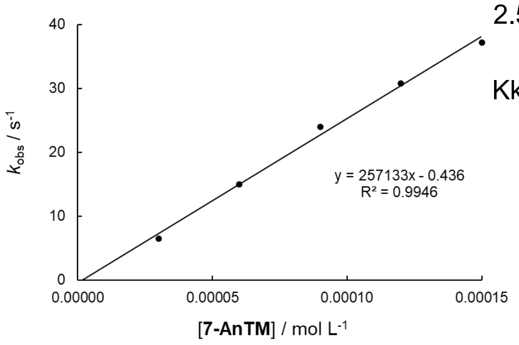	2.57×10^{5}
	6.00×10^{-5}	15.0		Kk47m-58
	9.00×10^{-5}	24.0		
	1.20×10^{-4}	30.8		
	1.50×10^{-4}	37.2		

$[(mor)_2CH^+]$ (mol L^{-1})	[7-AnTM] (mol L^{-1})	k_{obs} (s^{-1})	λ = 620 nm	k (M^{-1} s^{-1})
$6.00 \cdot 10^{-6}$	3.00×10^{-5}	10.3	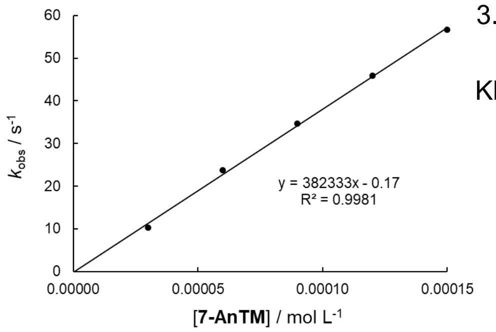	3.82×10^{5}
	6.00×10^{-5}	23.8		Kk47m-59
	9.00×10^{-5}	34.6		
	1.20×10^{-4}	45.9		
	1.50×10^{-4}	56.6		

Reactivity Parameters for 7-AnTM (**MeO-ITU2**) in CH_2Cl_2 at 20 °C

$(Ar)_2CH^+$	E	k (M^{-1} s^{-1})
$(jul)_2CH^+$	-9.45	4.20×10^{2}
$(pyr)_2CH^+$	-7.69	1.85×10^{4}
$(dma)_2CH^+$	-7.02	6.70×10^{4}
$(mpa)_2CH^+$	-5.89	2.57×10^{5}
$(mor)_2CH^+$	-5.53	3.82×10^{5}

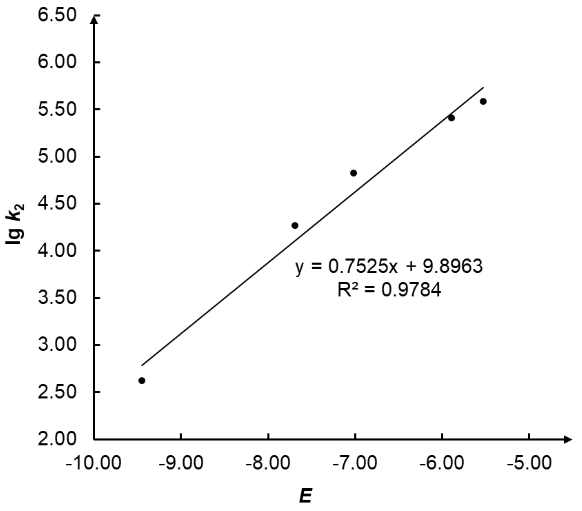

***N* = 13.15**

s_N = 0.75

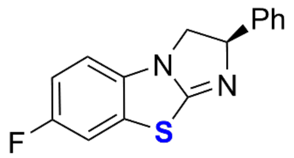

F-ITU2
7-F-BTM

Table S26: Kinetics of the reaction between 7-F-BTM (**F-ITU2**) and the benzhydrylium ions $(Ar)_2CH^+$ in CH_2Cl_2.at 20 °C.

$[(pyr)_2CH^+]$ (mol L^{-1})	[6-F-BTM] (mol L^{-1})	k_{obs} (s^{-1})	λ = 620 nm	k (M^{-1} s^{-1})
7.00 · 10^{-6}	7.00 x 10^{-5}	1.44		5.97 x 10^3
	1.40 x 10^{-4}	1.88		LSM-150
	2.10 x 10^{-4}	2.30		
	2.80 x 10^{-4}	2.70		
	3.50 x 10^{-4}	3.12		

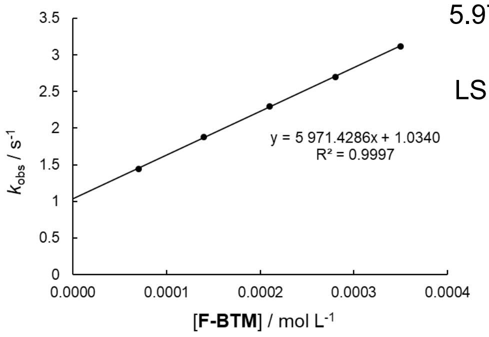

$[(dma)_2CH^+]$ (mol L^{-1})	[6-F-BTM] (mol L^{-1})	k_{obs} (s^{-1})	λ = 613 nm	k (M^{-1} s^{-1})
7.00 · 10^{-6}	7.00 x 10^{-5}	1.53		1.81 x 10^4
	1.40 x 10^{-4}	2.71		LSM-149
	2.10 x 10^{-4}	4.00		
	2.80 x 10^{-4}	5.18		
	3.50 x 10^{-4}	6.63		

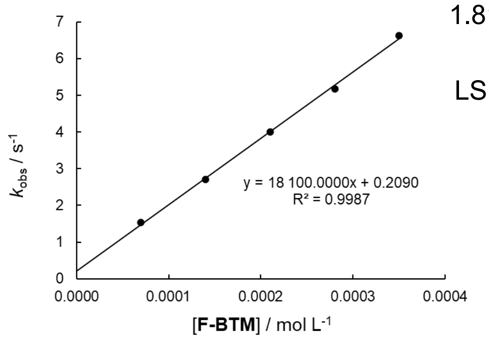

$[(mpa)_2CH^+]$ (mol L^{-1})	[6-F-BTM] (mol L^{-1})	k_{obs} (s^{-1})	λ = 622 nm	k (M^{-1} s^{-1})
7.00 · 10^{-6}	3.50 x 10^{-5}	2.80		9.79 x 10^4
	7.00 x 10^{-5}	5.94		LS390-151
	1.05 x 10^{-4}	9.19		
	1.40 x 10^{-4}	12.6		
	1.75 x 10^{-4}	16.6		

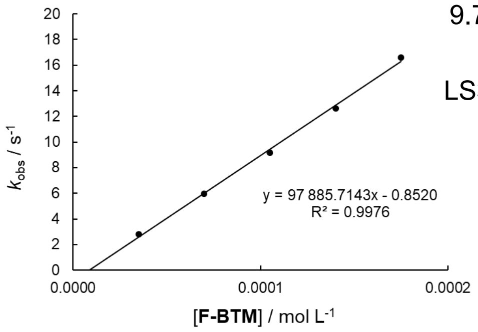

Table S26 continued: Kinetics of the reaction between 7-F-BTM (**F-ITU2**) and the benzhydrylium ions $(Ar)_2CH^+$ in CH_2Cl_2.at 20 °C.

$[(mor)_2CH^+]$ (mol L^{-1})	[6-F-BTM] (mol L^{-1})	k_{obs} (s^{-1})	λ = 620 nm	k (M^{-1} s^{-1})
$7.00 \cdot 10^{-6}$	3.50×10^{-5}	4.61	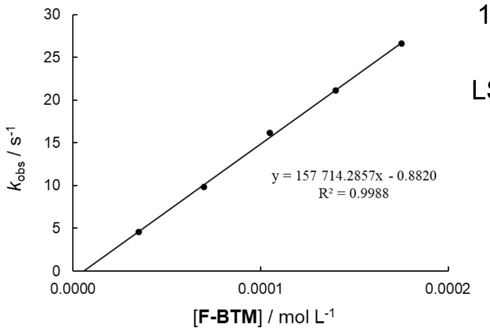	1.58×10^5
	7.00×10^{-5}	9.88		LS390-152
	1.05×10^{-4}	16.2		
	1.40×10^{-4}	21.1		
	1.75×10^{-4}	26.6		

Reactivity Parameters for 7-F-BTM (**F-ITU2**) in CH_2Cl_2 at 20 °C

$(Ar)_2CH^+$	E	k (M^{-1} s^{-1})
$(pyr)_2CH^+$	-7.69	5.97×10^3
$(dma)_2CH^+$	-7.02	1.81×10^4
$(mpa)_2CH^+$	-5.89	9.79×10^4
$(mor)_2CH^+$	-5.53	1.58×10^5

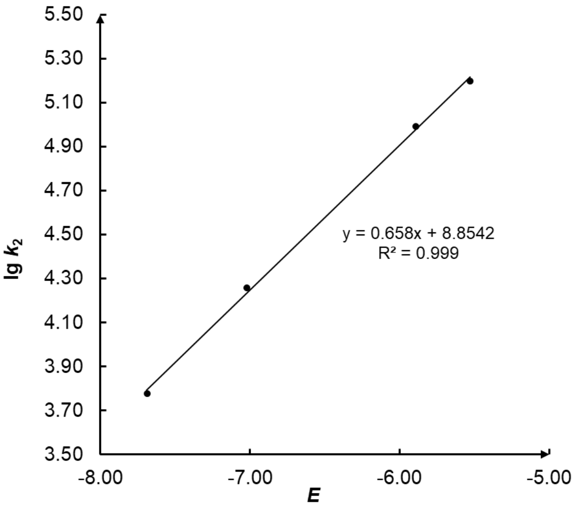

***N* = 13.45**

***s_N* = 0.66**

11.3 Solvent Effects

Apart from CH_2Cl_2 also MeCN and THF are solvents that are commonly employed in reactions involving isochalcogenoureas. We, therefore, performed the kinetic studies for SeHyperBTM (**ISeU5**) also in these two solvents analogously as described before.

Owing to the low solubility of benzhydrylium tetrafluoroborates in pure THF, stock solutions were prepared by dissolving $(Ar)_2CH^+$ BF_4^- in 0.5 mL acetonitrile. Then, THF was added to achieve a total volume of 10 mL of the solution [95/5 v/v) THF/MeCN]. In the optical cell of the stopped-flow instrument the $(Ar)_2CH^+$ BF_4^- solution is mixed in a 1:1 ratio with THF solution of the nucleophile. Consequently, "in THF" refers to a solvent system that actually consists of THF with a content of 2.5 vol-% MeCN.

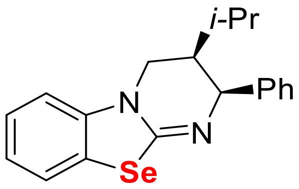

ISeU5
Se*Hyper*BTM

Table S27: Kinetics of the reaction between Se*Hyper*BTM (**ISeU5**) and the benzhydrylium ions $(Ar)_2CH^+$ in **MeCN** at 20 °C.

$[(ind)_2CH^+]$ (mol L^{-1})	[SeHyperBTM] (mol L^{-1})	k_{obs} (s^{-1})	λ = 616 nm	k_2 (L mol^{-1} s^{-1})
8.00×10^{-6}	8.00×10^{-5}	1.82	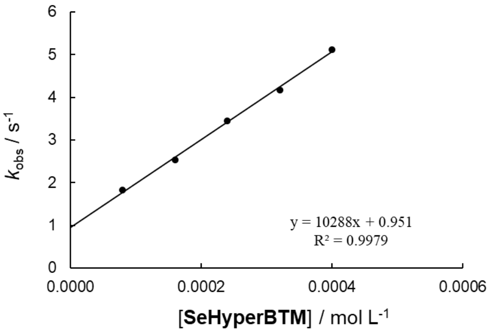	1.03×10^4
	1.60×10^{-4}	2.54		LSM-030
	2.40×10^{-4}	3.45		
	3.20×10^{-4}	4.17		
	4.00×10^{-4}	5.12		
$[(pyr)_2CH^+]$ (mol L^{-1})	[SeHyperBTM] (mol L^{-1})	k_{obs} (s^{-1})	λ = 611 nm	k_2 (L mol^{-1} s^{-1})
8.00×10^{-6}	8.00×10^{-5}	4.63	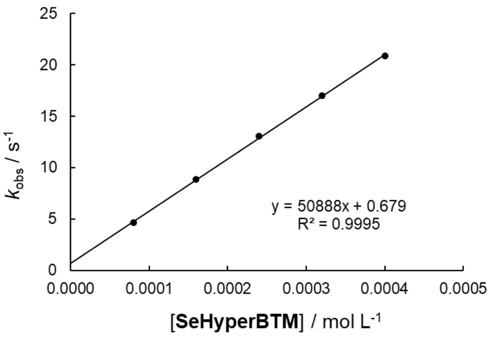	5.09×10^4
	1.60×10^{-4}	8.83		LSM-028
	2.40×10^{-4}	13.1		
	3.20×10^{-4}	17.0		
	4.00×10^{-4}	20.9		

Table S27 continued: Kinetics of the reaction between Se*Hyper*BTM (**ISeU5**) and the benzhydrylium ions $(Ar)_2CH^+$ in **MeCN** at 20 °C.

$[(dma)_2CH^+]$ (mol L^{-1})	[SeHyperBTM] (mol L^{-1})	k_{obs} (s^{-1})	λ = 605 nm	k_2 (L mol^{-1} s^{-1})
8.00×10^{-6}	8.00×10^{-5}	10.8	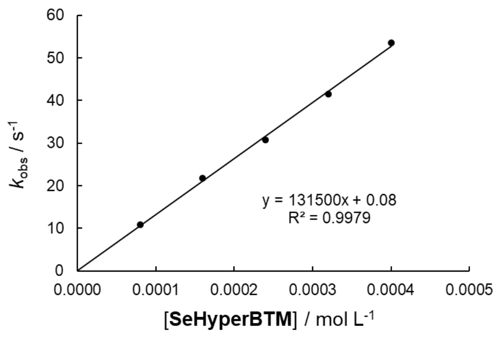	1.32×10^5
	1.60×10^{-4}	21.7		LSM-029
	2.40×10^{-4}	30.7		
	3.20×10^{-4}	41.5		
	4.00×10^{-4}	53.5		

$[(mor)_2CH^+]$ (mol L^{-1})	[SeHyperBTM] (mol L^{-1})	k_{obs} (s^{-1})	λ = 620 nm	k_2 (L mol^{-1} s^{-1})
8.00×10^{-6}	4.00×10^{-5}	26.2	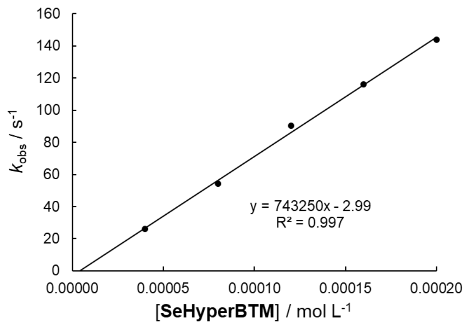	7.43×10^5
	8.00×10^{-5}	54.3		LSM-031
	1.20×10^{-4}	90.5		
	1.60×10^{-4}	116		
	2.00×10^{-4}	144		

Table S28: Kinetics of the reaction between Se*Hyper*BTM (**ISeU5**) and the benzhydrylium ions $(Ar)_2CH^+$ in **THF** at 20 °C.

$[(ind)_2CH^+]$ (mol L^{-1})	[SeHyperBTM] (mol L^{-1})	k_{obs} (s^{-1})	λ = 625 nm	k_2 (L mol^{-1} s^{-1})
$8.00\ 10^{-6}$	8.00×10^{-5}	2.28	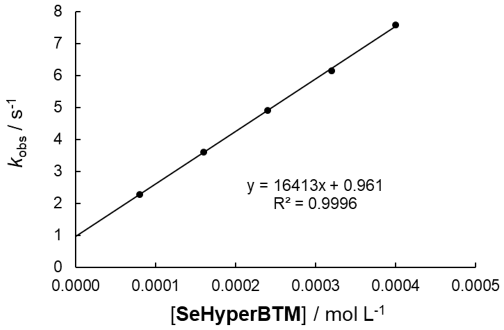	1.64×10^4
	1.60×10^{-4}	3.60		LSM-035
	2.40×10^{-4}	4.90		
	3.20×10^{-4}	6.15		
	4.00×10^{-4}	7.57		

Table S28 continued: Kinetics of the reaction between Se*Hyper*BTM (**ISeU5**) and the benzhydrylium ions $(Ar)_2CH^+$ in **THF** at 20 °C.

$[(pyr)_2CH^+]$ (mol L^{-1})	[SeHyperBTM] (mol L^{-1})	k_{obs} (s^{-1})	λ = 620 nm	k_2 (L mol^{-1} s^{-1})
8.00 10^{-6}	8.00×10^{-5}	9.22	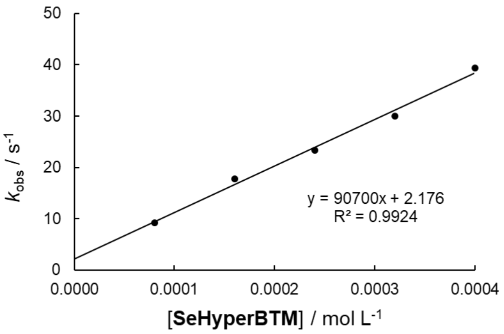	9.07×10^4
	1.60×10^{-4}	17.8		LSM-033
	2.40×10^{-4}	23.3		
	3.20×10^{-4}	30.0		
	4.00×10^{-4}	39.4		

$[(dma)_2CH^+]$ (mol L^{-1})	[SeHyperBTM] (mol L^{-1})	k_{obs} (s^{-1})	λ = 613 nm	k_2 (L mol^{-1} s^{-1})
8.00 10^{-6}	8.00×10^{-5}	21.1	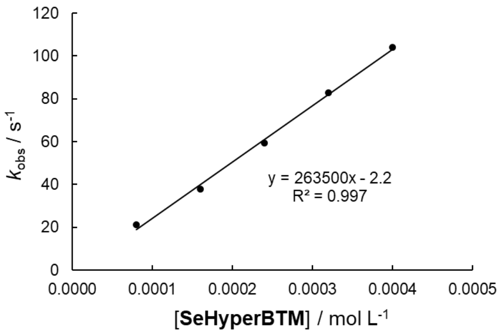	2.64×10^5
	1.60×10^{-4}	37.9		LSM-034
	2.40×10^{-4}	59.3		
	3.20×10^{-4}	82.9		
	4.00×10^{-4}	104		

$[(mpa)_2CH^+]$ (mol L^{-1})	[SeHyperBTM] (mol L^{-1})	k_{obs} (s^{-1})	λ = 622 nm	k_2 (L mol^{-1} s^{-1})
8.00×10^{-6}	4.00×10^{-5}	88.3	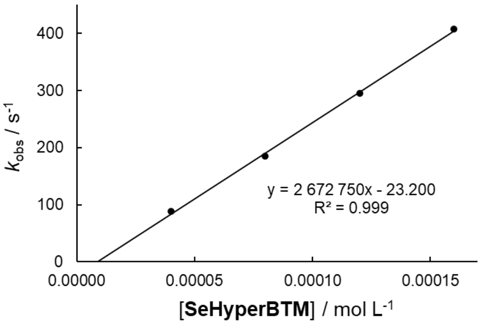	2.67×10^6
	8.00×10^{-5}	185		LSM-036
	1.20×10^{-4}	295		
	1.60×10^{-4}	408		

Table S29: Reactivity parameters for the reactions of Se*Hyper*BTM (**ISeU5**) and the benzhydrylium ions in different solvents.

Reactivity Parameters for Se*Hyper*BTM (**ISeU5**) in CH_2Cl_2, MeCN and THF at 20 °C						
$(Ar)_2CH^+$	E	k_{DCM} (L mol^{-1} s^{-1})	k_{MeCN} (L mol^{-1} s^{-1})	k_{THF} (L mol^{-1} s^{-1})		
$(ind)_2CH^+$	-8.76		1.03×10^4	1.64×10^4	**DCM**	N = 16.11
$(thq)_2CH^+$	-8,22	3.35×10^4	-	-		s_N = 0.58
$(pyr)_2CH^+$	-7.69	8.19×10^4	5.09×10^4	9.07×10^4	**MeCN**	N = 15.84
$(dma)_2CH^+$	-7.02	2.65×10^5	1.32×10^5	2.64×10^5		s_N = 0.57
$(mpa)_2CH^+$	-5.89	8.47×10^5	-	2.67×10^6	**THF**	N = 14.19
$(mor)_2CH^+$	-5.53	1.35×10^6	7.43×10^5	-		s_N = 0.77

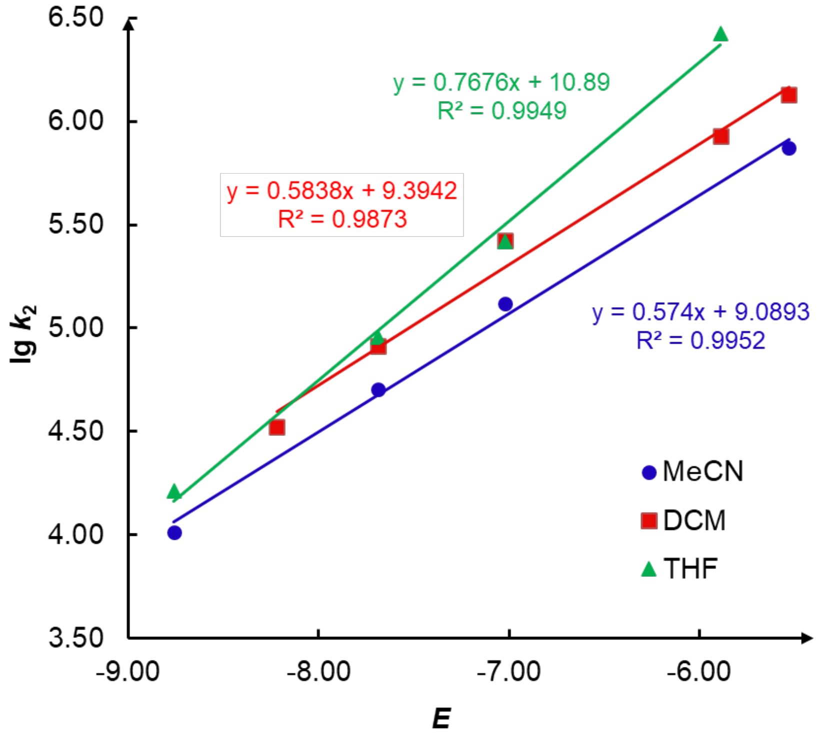

Figure S29: Correlation of second-order rate contants (lg k_2) for reactions of SeHyperBTM (**ISeU5**) with benzhydrylium ions in DCM (red), MeCN (blue) and THF (green) with the solvent-independent electrophilicity parameters E of $(Ar)_2CH^+$.

12. Equilibrium Constants

Assuming the validity of the Lambert-Beer law for dilute solutions, the concentration and the absorbance of the benzhydrylium ions in solution are proportional. Thus, equilibrium constants K (M^{-1}) as defined in equation (S2) can be calculated from the absorbance of the benzhydrylium ion solutions before (A_0) and after (A_{eq}) IchU addition.

$$IChU + Ar_2CH^+(\mathbf{18}) \rightleftharpoons Ar_2CH - IChU^+\ (\mathbf{19}) \qquad \text{(S1)}$$

$$K = \frac{[Ar_2CH{-}IChU^+]}{[Ar_2CH^+]\cdot[IChU]} = \frac{A_0 - A}{A\cdot[IChU]} \qquad \text{(S2)}$$

In order to determine equilibrium constants for the association reactions of IchUs with benzhydrylium ions Ar_2CH^+ (**18**) to give the adducts **19** (equation S1) IchU solutions in dichloromethane were added stepwise to a solution of benzhydrylium tetrafluoroborates in the same solvent at (20.0 ± 0.1) °C. The decay of the absorbance of the benzhydrylium ions at their respective absorption maxima was monitored via UV/vis spectroscopy. As soon as the absorbance was constant, the equilibrium absorbance (A_{eq}) was recorded. Then the next portion of the IchU solution was added. For each IchU/Ar_2CH^+ combination, the photometric titrations were performed three times.

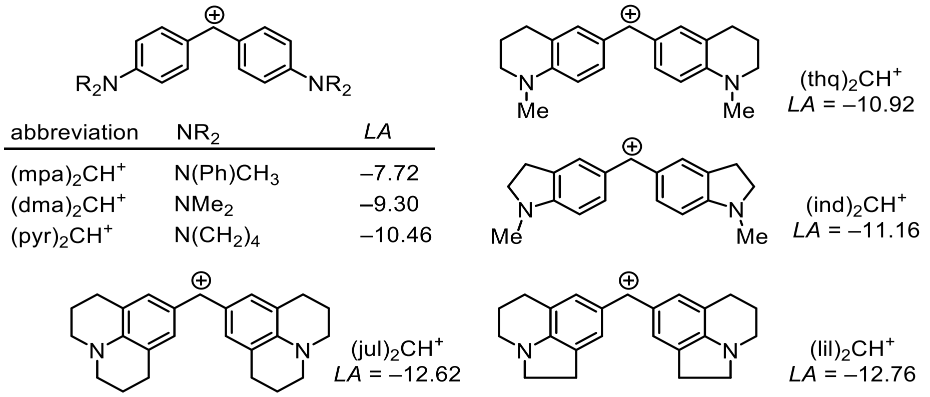

Figure S30: Lewis acidities *LA* of benzhydrylium ions Ar_2CH^+ used as reference Lewis acids (*LA* in dichloromethane as reported in literature[20]; counterion: BF_4^-).

Averaged equilibrium constants were used for subsequent calculations of Lewis basicities *LB*. Accoridng to equation (S3), we calculated Lewis basicities *LB* of IChUs from reported Lewis acidities *LA* of benzhydrylium ions and experimentally determined equilibrium constants *K*(20°C).

$$\lg K(20\ °C) = LA + LB \qquad \text{(S3)}$$

If more than one Lewis acid was used for equilibrium measurements with a certain IChUs, the individual *LB* values were averaged for use in the main manuscript.

ODHPB (IU3) + $(pyr)_2CH^+$ BF_4^-

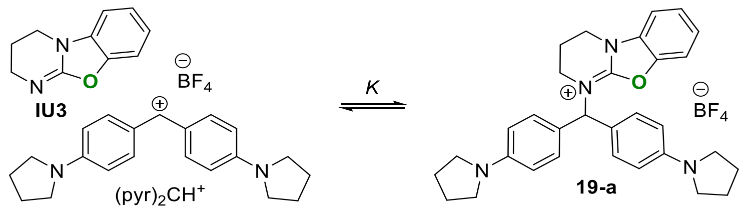

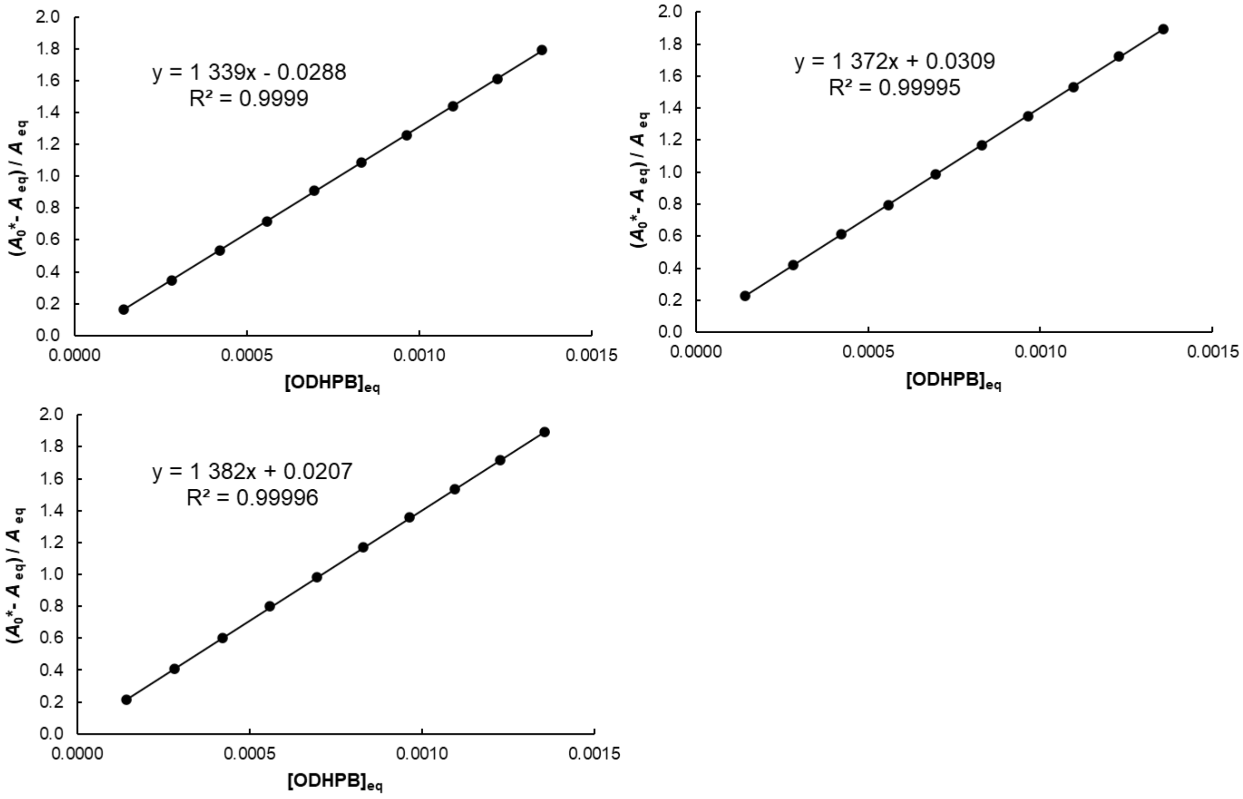

Figure S31: Linear regression plots for the determination of the equilibrium constants for the association of ODHPB (**IU3**) with $(pyr)_2CH^+$ in dichloromethane at 20 °C (monitored at 620 nm, ε(620 nm) = 1.74×10^5 L mol^{-1} cm^{-1}). LSM-63

Table S30: Calculation of the equilibrium constant *K* for the association of ODHPB (**IU3**) with $(pyr)_2CH^+$ in dichloromethane at 20.0 °C.

	K (L mol^{-1})
Titration #1	1.34×10^3
Titration #2	1.37×10^3
Titration #3	1.38×10^3
**K_{av} =**	**(1.36 ± 0.02) × 10^3 L mol^{-1}**

Table S31: Determination of the association constant of ODHPB (**IU3**) with $(pyr)_2CH^+$ in dichloromethane at 20.0 °C (monitored at 620 nm, ε(620 nm) = 1.74 × 10^5 L mol^{-1} cm^{-1}). LSM-63

Titration	Step	$V_{tot,Nu}$ (mL)	V_{tot} (mL)	A_{eq}	$[pyr]_0$ (mol L^{-1})	A_0*	A_0*– A_{eq}	$[ODHPB]_0$ (mol L^{-1})	$[ODHPB]_{eq}$ (mol L^{-1})	(A_0* – A_{eq})/A_{eq}
#1	0	0	17.1	0.808	9.93 × 10^{-6}	0.808				
	1	0.1	17.2	0.689	9.87 × 10^{-6}	0.804	0.114	1.43 × 10^{-4}	1.42 × 10^{-4}	0.166
	2	0.2	17.3	0.593	9.81 × 10^{-6}	0.799	0.206	2.85 × 10^{-4}	2.83 × 10^{-4}	0.348
	3	0.3	17.4	0.517	9.75 × 10^{-6}	0.794	0.277	4.25 × 10^{-4}	4.22 × 10^{-4}	0.537
	4	0.4	17.5	0.461	9.70 × 10^{-6}	0.790	0.329	5.64 × 10^{-4}	5.60 × 10^{-4}	0.715
	5	0.5	17.6	0.411	9.64 × 10^{-6}	0.785	0.374	7.01 × 10^{-4}	6.97 × 10^{-4}	0.910
	6	0.6	17.7	0.374	9.59 × 10^{-6}	0.781	0.406	8.36 × 10^{-4}	8.32 × 10^{-4}	1.09
	7	0.7	17.8	0.344	9.54 × 10^{-6}	0.776	0.432	9.70 × 10^{-4}	9.65 × 10^{-4}	1.26
	8	0.8	17.9	0.316	9.48 × 10^{-6}	0.772	0.456	1.10 × 10^{-3}	1.10 × 10^{-3}	1.44
	9	0.9	18.0	0.294	9.43 × 10^{-6}	0.768	0.474	1.23 × 10^{-3}	1.23 × 10^{-3}	1.61
	10	1.0	18.1	0.273	9.38 × 10^{-6}	0.764	0.490	1.36 × 10^{-3}	1.36 × 10^{-3}	1.80
#2	0	0	17.1	0.806	9.92 × 10^{-6}	0.806				
	1	0.1	17.2	0.653	9.86 × 10^{-6}	0.801	0.148	1.43 × 10^{-4}	1.42 × 10^{-4}	0.227
	2	0.2	17.3	0.561	9.81 × 10^{-6}	0.797	0.236	2.85 × 10^{-4}	2.82 × 10^{-4}	0.421
	3	0.3	17.4	0.492	9.75 × 10^{-6}	0.792	0.301	4.25 × 10^{-4}	4.22 × 10^{-4}	0.611
	4	0.4	17.5	0.439	9.69 × 10^{-6}	0.788	0.349	5.64 × 10^{-4}	5.60 × 10^{-4}	0.794
	5	0.5	17.6	0.394	9.64 × 10^{-6}	0.783	0.389	7.01 × 10^{-4}	6.96 × 10^{-4}	0.986
	6	0.6	17.7	0.359	9.58 × 10^{-6}	0.779	0.420	8.36 × 10^{-4}	8.31 × 10^{-4}	1.17
	7	0.7	17.8	0.329	9.53 × 10^{-6}	0.774	0.445	9.70 × 10^{-4}	9.65 × 10^{-4}	1.35
	8	0.8	17.9	0.304	9.48 × 10^{-6}	0.770	0.466	1.10 × 10^{-3}	1.10 × 10^{-3}	1.53
	9	0.9	18.0	0.281	9.43 × 10^{-6}	0.766	0.485	1.23 × 10^{-3}	1.23 × 10^{-3}	1.72
	10	1.0	18.1	0.263	9.37 × 10^{-6}	0.762	0.498	1.36 × 10^{-3}	1.36 × 10^{-3}	1.89
#3	0	0	17.1	0.812	9.92 × 10^{-6}	0.812				
	1	0.1	17.2	0.666	9.86 × 10^{-6}	0.807	0.142	1.43 × 10^{-4}	1.42 × 10^{-4}	0.213
	2	0.2	17.3	0.570	9.80 × 10^{-6}	0.803	0.233	2.85 × 10^{-4}	2.82 × 10^{-4}	0.409
	3	0.3	17.4	0.498	9.75 × 10^{-6}	0.798	0.300	4.25 × 10^{-4}	4.22 × 10^{-4}	0.603
	4	0.4	17.5	0.441	9.69 × 10^{-6}	0.794	0.353	5.64 × 10^{-4}	5.59 × 10^{-4}	0.800
	5	0.5	17.6	0.398	9.64 × 10^{-6}	0.789	0.391	7.00 × 10^{-4}	6.96 × 10^{-4}	0.982
	6	0.6	17.7	0.361	9.58 × 10^{-6}	0.785	0.424	8.36 × 10^{-4}	8.31 × 10^{-4}	1.17
	7	0.7	17.8	0.331	9.53 × 10^{-6}	0.780	0.449	9.70 × 10^{-4}	9.64 × 10^{-4}	1.36
	8	0.8	17.9	0.306	9.47 × 10^{-6}	0.776	0.470	1.10 × 10^{-3}	1.10 × 10^{-3}	1.53
	9	0.9	18.0	0.284	9.42 × 10^{-6}	0.771	0.487	1.23 × 10^{-3}	1.23 × 10^{-3}	1.71
	10	1.0	18.1	0.265	9.37 × 10^{-6}	0.767	0.502	1.36 × 10^{-3}	1.36 × 10^{-3}	1.89

ODHPB (IU3) + (ind)$_2$CH$^+$

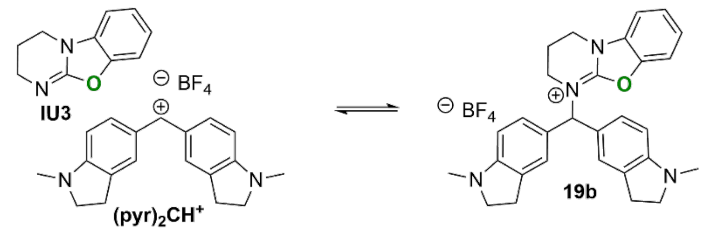

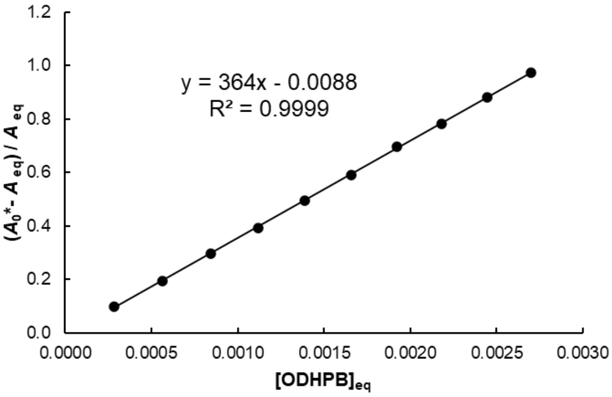

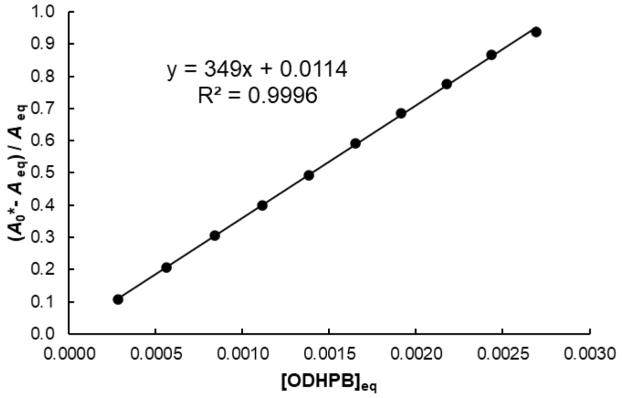

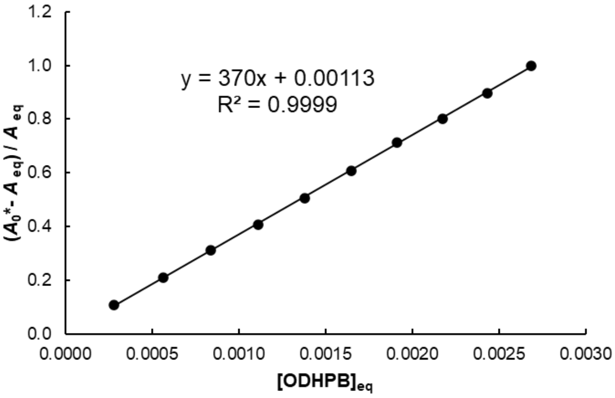

Figure S32: Linear regression plots for the determination of the equilibrium constants for the association of ODHPB (**IU3**) and (ind)$_2$CH$^+$ (observed at 625 nm, ε(625 nm) = 1.32 × 10^5 L mol^{-1} cm^{-1}). LSM-64

Table S32: Calculation of the equilibrium constant *K* for the association of ODHPB (**IU3**) and (ind)$_2$CH$^+$

	K (L mol^{-1})
Titration #1	3.64 × 10^2
Titration #2	3.49 × 10^2
Titration #3	3.70 × 10^2
***K*$_{av}$ =**	**(3.61 ± 0.09) × 10^2 L mol^{-1}**

Table S33: Determination of the association constant of ODHPB (**IU3**) and $(ind)_2CH^+$ (observed at 625 nm, ε(625 nm) = 1.32 × 10^5 M^{-1} cm^{-1}). LSM-64

Titration	Step	$V_{tot,Nu}$ (mL)	V_{tot} (mL)	A_{eq}	$[ind]_0$ (mol L^{-1})	A_0*	A_0*– A_{eq}	$[ODHPB]_0$ (mol L^{-1})	$[ODHPB]_{eq}$ (mol L^{-1})	(A_0* – A_{eq})/A_{eq}
#1	0	0	17.0	0.421	1.01 × 10^{-5}	0.421				
	1	0.1	17.1	0.380	1.00 × 10^{-5}	0.418	0.038	2.84 × 10^{-4}	2.84 × 10^{-4}	0.100
	2	0.2	17.2	0.348	9.98 × 10^{-6}	0.416	0.068	5.65 × 10^{-4}	5.64 × 10^{-4}	0.195
	3	0.3	17.3	0.319	9.92 × 10^{-6}	0.414	0.095	8.43 × 10^{-4}	8.42 × 10^{-4}	0.297
	4	0.4	17.4	0.295	9.86 × 10^{-6}	0.411	0.116	1.12 × 10^{-3}	1.12 × 10^{-3}	0.393
	5	0.5	17.5	0.273	9.80 × 10^{-6}	0.409	0.135	1.39 × 10^{-3}	1.39 × 10^{-3}	0.495
	6	0.6	17.6	0.255	9.75 × 10^{-6}	0.407	0.151	1.66 × 10^{-3}	1.66 × 10^{-3}	0.592
	7	0.7	17.7	0.238	9.69 × 10^{-6}	0.404	0.166	1.92 × 10^{-3}	1.92 × 10^{-3}	0.697
	8	0.8	17.8	0.225	9.64 × 10^{-6}	0.402	0.176	2.19 × 10^{-3}	2.18 × 10^{-3}	0.783
	9	0.9	17.9	0.212	9.58 × 10^{-6}	0.400	0.187	2.44 × 10^{-3}	2.44 × 10^{-3}	0.883
	10	1	18.0	0.201	9.53 × 10^{-6}	0.397	0.196	2.70 × 10^{-3}	2.70 × 10^{-3}	0.975
#2	0	0	17.0	0.436	1.01 × 10^{-5}	0.436				
	1	0.1	17.1	0.391	1.00 × 10^{-5}	0.433	0.042	2.84 × 10^{-4}	2.83 × 10^{-4}	0.107
	2	0.2	17.2	0.357	9.95 × 10^{-6}	0.431	0.074	5.64 × 10^{-4}	5.63 × 10^{-4}	0.207
	3	0.3	17.3	0.328	9.89 × 10^{-6}	0.428	0.100	8.41 × 10^{-4}	8.40 × 10^{-4}	0.306
	4	0.4	17.4	0.304	9.83 × 10^{-6}	0.426	0.121	1.12 × 10^{-3}	1.11 × 10^{-3}	0.398
	5	0.5	17.5	0.284	9.78 × 10^{-6}	0.423	0.140	1.39 × 10^{-3}	1.38 × 10^{-3}	0.493
	6	0.6	17.6	0.264	9.72 × 10^{-6}	0.421	0.157	1.65 × 10^{-3}	1.65 × 10^{-3}	0.592
	7	0.7	17.7	0.248	9.67 × 10^{-6}	0.419	0.170	1.92 × 10^{-3}	1.92 × 10^{-3}	0.685
	8	0.8	17.8	0.234	9.61 × 10^{-6}	0.416	0.182	2.18 × 10^{-3}	2.18 × 10^{-3}	0.777
	9	0.9	17.9	0.222	9.55 × 10^{-6}	0.414	0.192	2.44 × 10^{-3}	2.44 × 10^{-3}	0.866
	10	1	18.0	0.212	9.51 × 10^{-6}	0.412	0.199	2.69 × 10^{-3}	2.69 × 10^{-3}	0.938
#3	0	0	17.1	0.398	1.00 × 10^{-5}	0.398				
	1	0.1	17.2	0.357	9.99 × 10^{-6}	0.396	0.039	2.83 × 10^{-4}	2.83 × 10^{-4}	0.108
	2	0.2	17.3	0.325	9.93 × 10^{-6}	0.393	0.068	5.63 × 10^{-4}	5.62 × 10^{-4}	0.209
	3	0.3	17.4	0.298	9.87 × 10^{-6}	0.391	0.093	8.40 × 10^{-4}	8.38 × 10^{-4}	0.314
	4	0.4	17.5	0.276	9.82 × 10^{-6}	0.389	0.113	1.11 × 10^{-3}	1.11 × 10^{-3}	0.408
	5	0.5	17.6	0.257	9.76 × 10^{-6}	0.387	0.130	1.38 × 10^{-3}	1.38 × 10^{-3}	0.507
	6	0.6	17.7	0.239	9.71 × 10^{-6}	0.385	0.146	1.65 × 10^{-3}	1.65 × 10^{-3}	0.609
	7	0.7	17.8	0.223	9.65 × 10^{-6}	0.382	0.159	1.92 × 10^{-3}	1.91 × 10^{-3}	0.713
	8	0.8	17.9	0.211	9.60 × 10^{-6}	0.380	0.169	2.18 × 10^{-3}	2.17 × 10^{-3}	0.803
	9	0.9	18.0	0.199	9.54 × 10^{-6}	0.378	0.179	2.43 × 10^{-3}	2.43 × 10^{-3}	0.898
	10	1	18.1	0.188	9.49 × 10^{-6}	0.376	0.188	2.69 × 10^{-3}	2.69 × 10^{-3}	1.00

OHyperBTM + $(dma)_2CH^+$

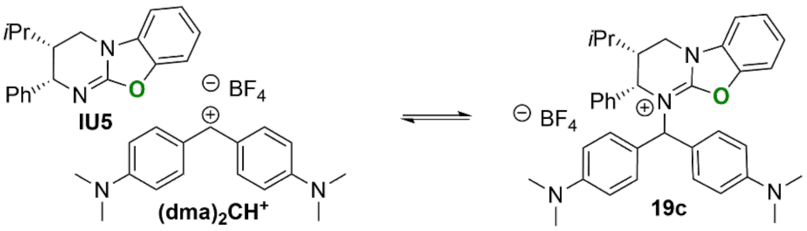

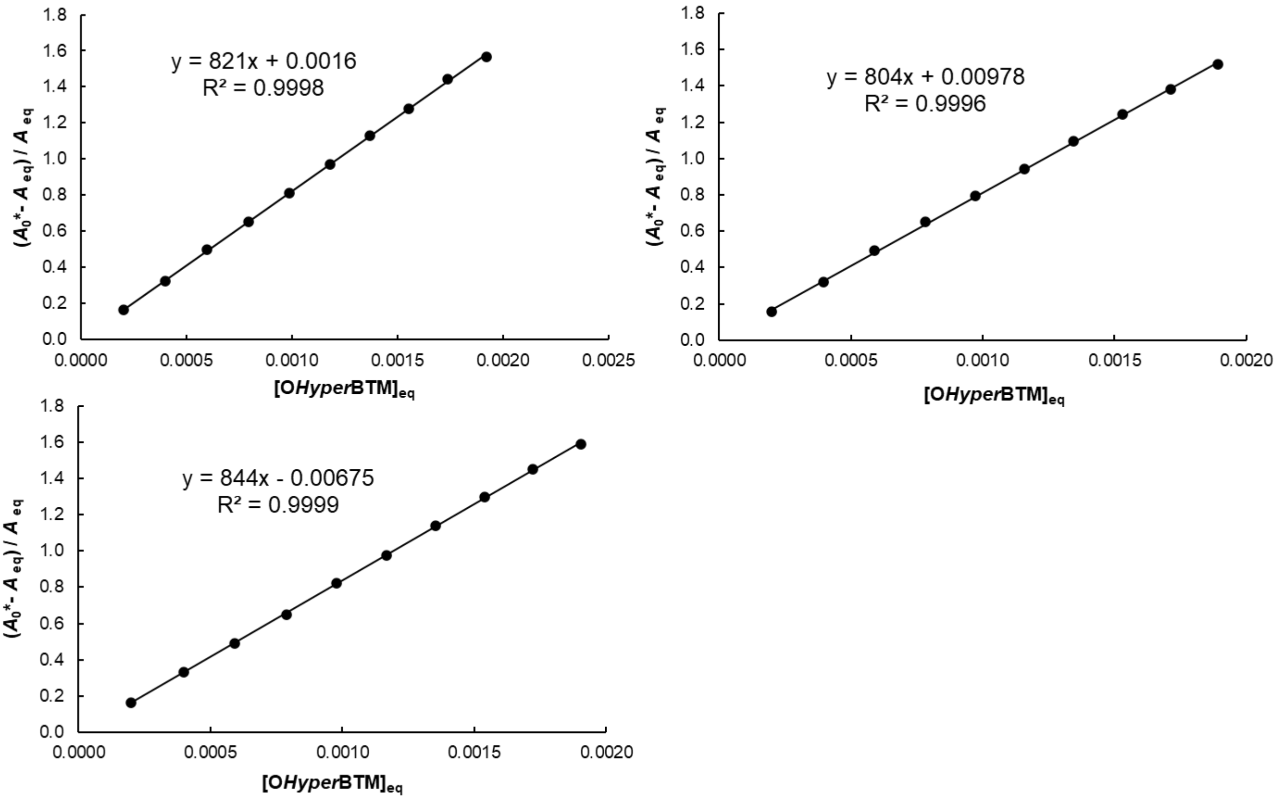

Figure S33: Linear regression plots for the determination of the equilibrium constants between O*Hyper*BTM (**IU5**) and $(dma)_2CH^+$ (observed at 613 nm). ε(dma, 613 nm = 1.70×10^5 L mol^{-1} cm^{-1}). LSM-79

Table S34: Calculation of the equilibrium constant K for the reaction between O*Hype*rBTM (**IU5**) and $(dma)_2CH^+$

	K (L mol^{-1})
Titration #1	8.21×10^2
Titration #2	8.04×10^2
Titration #3	8.44×10^2
**K_{av} =**	**$(8.23 \pm 0.16) \times 10^2$ L mol^{-1}**

Table S35: Determination of the equilibrium constant between between O*Hyper*BTM (**IU5**) and $(dma)_2CH^+$ (observed at 613 nm). ε(dma, 613 nm = 1.70×10^5 L mol^{-1} cm^{-1}). LSM-79

Titration	Step	$V_{tot,Nu}$ (mL)	V_{tot} (mL)	A_{eq}	[dma]$_0$ (mol L^{-1})	A_0*	A_0*– A_{eq}	[OHyper]$_0$ (mol L^{-1})	[OHyper]$_{eq}$ (mol L^{-1})	(A_0* – A_{eq})/A_{eq}
#1	0	0	16.9	0.784	9.91×10^{-6}	0.784				
	1	0.1	17.0	0.668	9.85×10^{-6}	0.779	0.111	2.03×10^{-4}	2.02×10^{-4}	0.167
	2	0.2	17.1	0.585	9.79×10^{-6}	0.774	0.190	4.04×10^{-4}	4.01×10^{-4}	0.324
	3	0.3	17.2	0.514	9.73×10^{-6}	0.770	0.256	6.02×10^{-4}	5.99×10^{-4}	0.498
	4	0.4	17.3	0.463	9.68×10^{-6}	0.766	0.302	7.98×10^{-4}	7.94×10^{-4}	0.652
	5	0.5	17.4	0.420	9.62×10^{-6}	0.761	0.341	9.92×10^{-4}	9.88×10^{-4}	0.814
	6	0.6	17.5	0.384	9.57×10^{-6}	0.757	0.373	1.18×10^{-3}	1.18×10^{-3}	0.971
	7	0.7	17.6	0.353	9.51×10^{-6}	0.753	0.399	1.37×10^{-3}	1.37×10^{-3}	1.13
	8	0.8	17.7	0.328	9.46×10^{-6}	0.748	0.420	1.56×10^{-3}	1.55×10^{-3}	1.28
	9	0.9	17.8	0.305	9.40×10^{-6}	0.744	0.439	1.74×10^{-3}	1.74×10^{-3}	1.44
	10	1	17.9	0.288	9.35×10^{-6}	0.740	0.452	2.93×10^{-3}	1.92×10^{-3}	1.57
#2	0	0	17.2	0.729	9.73×10^{-6}	0.729				
	1	0.1	17.3	0.627	9.68×10^{-6}	0.725	0.097	1.99×10^{-4}	1.98×10^{-4}	0.155
	2	0.2	17.4	0.546	9.62×10^{-6}	0.721	0.174	3.97×10^{-4}	3.95×10^{-4}	0.319
	3	0.3	17.5	0.480	9.57×10^{-6}	0.717	0.236	5.92×10^{-4}	5.89×10^{-4}	0.492
	4	0.4	17.6	0.431	9.51×10^{-6}	0.713	0.282	7.84×10^{-4}	7.81×10^{-4}	0.654
	5	0.5	17.7	0.395	9.46×10^{-6}	0.708	0.314	9.75×10^{-4}	9.71×10^{-4}	0.795
	6	0.6	17.8	0.362	9.40×10^{-6}	0.705	0.342	1.16×10^{-3}	1.16×10^{-3}	0.944
	7	0.7	17.9	0.335	9.35×10^{-6}	0.701	0.366	1.35×10^{-3}	1.35×10^{-3}	1.09
	8	0.8	18.0	0.310	9.30×10^{-6}	0.697	0.386	1.53×10^{-3}	1.53×10^{-3}	1.25
	9	0.9	18.1	0.291	9.25×10^{-6}	0.693	0.402	1.72×10^{-3}	1.71×10^{-3}	1.38
	10	1	18.2	0.273	9.20×10^{-6}	0.689	0.416	2.90×10^{-3}	1.89×10^{-3}	1.52
#3	0	0	17.1	0.780	9.81×10^{-6}	0.780				
	1	0.1	17.2	0.666	9.75×10^{-6}	0.775	0.109	2.01×10^{-4}	2.00×10^{-4}	0.164
	2	0.2	17.3	0.579	9.69×10^{-6}	0.771	0.192	4.00×10^{-4}	3.97×10^{-4}	0.332
	3	0.3	17.4	0.514	9.64×10^{-6}	0.766	0.252	5.96×10^{-4}	5.93×10^{-4}	0.491
	4	0.4	17.5	0.463	9.58×10^{-6}	0.762	0.299	7.90×10^{-4}	7.87×10^{-4}	0.646
	5	0.5	17.6	0.416	9.53×10^{-6}	0.758	0.341	9.82×10^{-4}	9.78×10^{-4}	0.820
	6	0.6	17.7	0.381	9.47×10^{-6}	0.753	0.372	1.17×10^{-3}	1.17×10^{-3}	0.977
	7	0.7	17.8	0.350	9.42×10^{-6}	0.749	0.399	1.36×10^{-3}	1.35×10^{-3}	1.14
	8	0.8	17.9	0.324	9.37×10^{-6}	0.745	0.421	1.54×10^{-3}	1.54×10^{-3}	1.30
	9	0.9	18.0	0.302	9.31×10^{-6}	0.741	0.439	1.73×10^{-3}	1.72×10^{-3}	1.45
	10	1	18.1	0.284	9.26×10^{-6}	0.737	0.452	2.91×10^{-3}	1.90×10^{-3}	1.59

O*Hyper*BTM + (mpa)$_2$CH$^+$

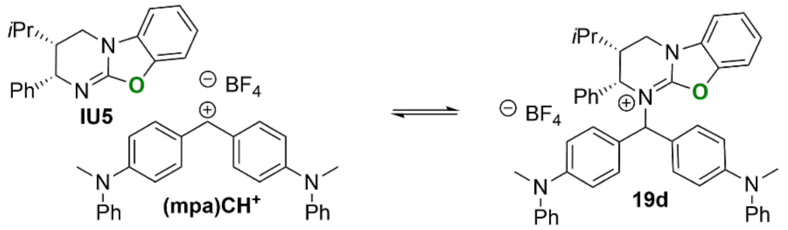

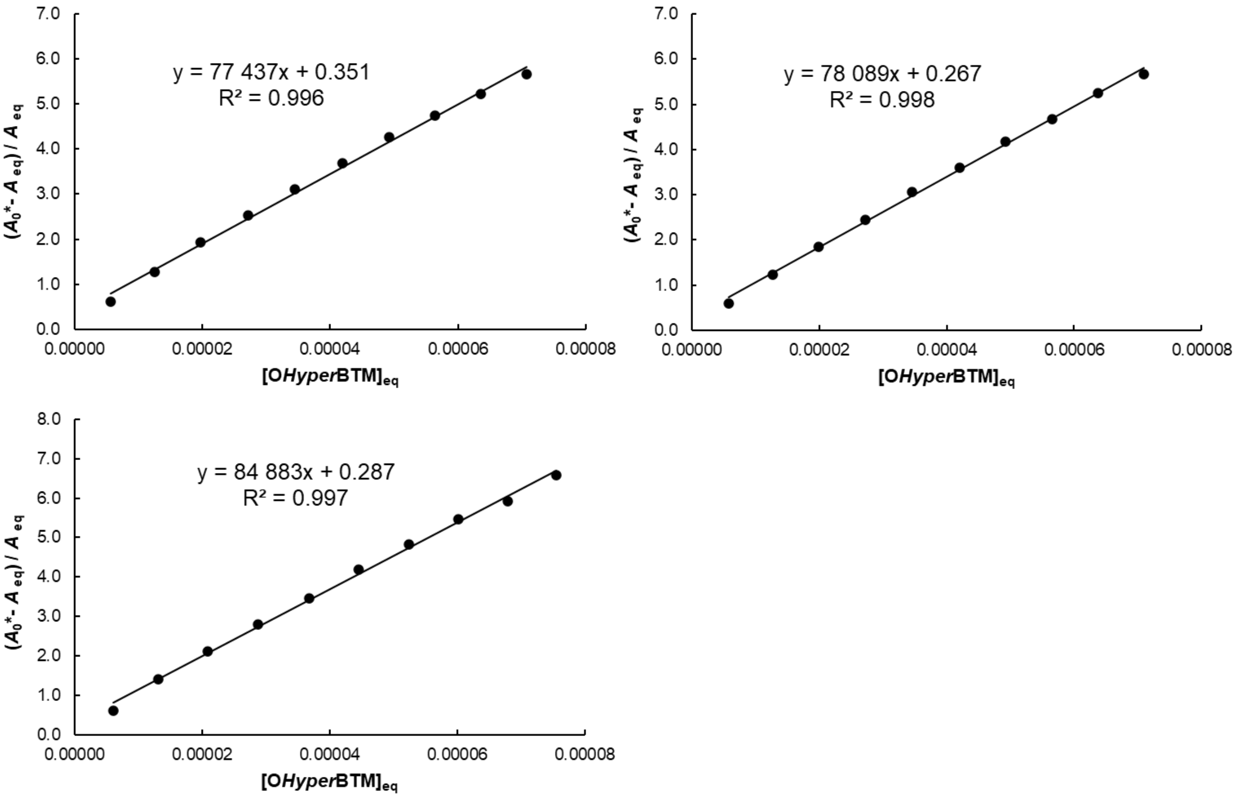

Figure S34: Linear regression plots for the determination of the equilibrium constants between O*Hyper*BTM (**IU5**) and (mpa)$_2$CH$^+$ (observed at 622 nm). ε(mpa, 622 nm = 1.41 × 10^5 L mol^{-1} cm^{-1}). LSM-76

Table S36: Calculation of the equilibrium constant K for the reaction between O*Hype*rBTM (**IU5**) and (mpa)$_2$CH$^+$

	K (L mol^{-1})
Titration #1	7.74 × 10^4
Titration #2	7.81 × 10^4
Titration #3	8.49 × 10^4
**K_{av} =**	**(8.01 ± 0.34) × 10^4 L mol^{-1}**

Table S37: Determination of the equilibrium constant between O*Hyper*BTM (**IU5**) and $(mpa)_2CH^+$ (observed at 622 nm). ε(mpa, 622 nm = 1.41 × 10^5 L mol^{-1} cm^{-1}). LSM-76

Titration	Step	$V_{tot,Nu}$ (mL)	V_{tot} (mL)	A_{eq}	$[mpa]_0$ (mol L^{-1})	A_0*	A_0*– A_{eq}	$[OHyper]_0$ (mol L^{-1})	$[OHyper]_{eq}$ (mol L^{-1})	(A_0* – A_{eq})/A_{eq}
#1	0	0	17.1	0.408	8.74 × 10^{-6}	0.408				
	1	0.1	17.2	0.250	8.69 × 10^{-6}	0.405	0.155	7.93 × 10^{-6}	5.73 × 10^{-6}	0.621
	2	0.2	17.3	0.177	8.64 × 10^{-6}	0.403	0.226	1.58 × 10^{-5}	1.26 × 10^{-5}	1.28
	3	0.3	17.4	0.137	8.59 × 10^{-6}	0.401	0.264	2.35 × 10^{-5}	1.98 × 10^{-5}	1.93
	4	0.4	17.5	0.113	8.55 × 10^{-6}	0.399	0.286	3.12 × 10^{-5}	2.72 × 10^{-5}	2.53
	5	0.5	17.6	0.0964	8.50 × 10^{-6}	0.396	0.300	3.88 × 10^{-5}	3.45 × 10^{-5}	3.11
	6	0.6	17.7	0.0840	8.45 × 10^{-6}	0.394	0.310	4.63 × 10^{-5}	4.19 × 10^{-5}	3.69
	7	0.7	17.8	0.0744	8.40 × 10^{-6}	0.392	0.317	5.37 × 10^{-5}	4.92 × 10^{-5}	4.27
	8	0.8	17.9	0.0679	8.35 × 10^{-6}	0.390	0.322	6.10 × 10^{-5}	5.64 × 10^{-5}	4.74
	9	0.9	18.0	0.0622	8.31 × 10^{-6}	0.387	0.325	6.82 × 10^{-5}	6.36 × 10^{-5}	5.23
	10	1	18.1	0.0578	8.26 × 10^{-6}	0.385	0.328	7.54 × 10^{-5}	7.08 × 10^{-5}	5.67
#2	0	0	17.1	0.397	8.74 × 10^{-6}	0.397				
	1	0.1	17.2	0.247	8.69 × 10^{-6}	0.395	0.148	7.93 × 10^{-6}	5.83 × 10^{-6}	0.600
	2	0.2	17.3	0.177	8.64 × 10^{-6}	0.393	0.216	1.58 × 10^{-5}	1.27 × 10^{-5}	1.22
	3	0.3	17.4	0.137	8.59 × 10^{-6}	0.391	0.254	2.35 × 10^{-5}	1.99 × 10^{-5}	1.85
	4	0.4	17.5	0.113	8.54 × 10^{-6}	0.388	0.276	3.12 × 10^{-5}	2.73 × 10^{-5}	2.45
	5	0.5	17.6	0.0951	8.49 × 10^{-6}	0.386	0.291	3.88 × 10^{-5}	3.46 × 10^{-5}	3.06
	6	0.6	17.7	0.0834	8.45 × 10^{-6}	0.384	0.301	4.62 × 10^{-5}	4.20 × 10^{-5}	3.60
	7	0.7	17.8	0.0737	8.40 × 10^{-6}	0.382	0.308	5.37 × 10^{-5}	4.93 × 10^{-5}	4.18
	8	0.8	17.9	0.0668	8.35 × 10^{-6}	0.380	0.313	6.10 × 10^{-5}	5.65 × 10^{-5}	4.68
	9	0.9	18.0	0.0604	8.30 × 10^{-6}	0.378	0.317	6.82 × 10^{-5}	6.37 × 10^{-5}	5.25
	10	1	18.1	0.0564	8.26 × 10^{-6}	0.375	0.319	7.54 × 10^{-5}	7.09 × 10^{-5}	5.66
#3	0	0	15.9	0.472	9.44 × 10^{-6}	0.472				
	1	0.1	16.0	0.292	9.38 × 10^{-6}	0.469	0.177	8.56 × 10^{-6}	6.05 × 10^{-6}	0.608
	2	0.2	16.1	0.194	9.32 × 10^{-6}	0.466	0.272	1.70 × 10^{-5}	1.32 × 10^{-5}	1.40
	3	0.3	16.2	0.149	9.27 × 10^{-6}	0.463	0.314	2.54 × 10^{-5}	2.09 × 10^{-5}	2.11
	4	0.4	16.3	0.121	9.21 × 10^{-6}	0.460	0.339	3.36 × 10^{-5}	2.88 × 10^{-5}	2.80
	5	0.5	16.4	0.102	9.15 × 10^{-6}	0.457	0.355	4.18 × 10^{-5}	3.67 × 10^{-5}	3.47
	6	0.6	16.5	0.087	9.10 × 10^{-6}	0.455	0.367	4.98 × 10^{-5}	4.46 × 10^{-5}	4.20
	7	0.7	16.6	0.0775	9.04 × 10^{-6}	0.452	0.374	5.78 × 10^{-5}	5.25 × 10^{-5}	4.83
	8	0.8	16.7	0.0695	8.99 × 10^{-6}	0.449	0.380	6.56 × 10^{-5}	6.02 × 10^{-5}	5.46
	9	0.9	16.8	0.0644	8.93 × 10^{-6}	0.446	0.382	7.34 × 10^{-5}	6.80 × 10^{-5}	5.93
	10	1	16.9	0.0585	8.88 × 10^{-6}	0.444	0.385	8.11 × 10^{-5}	7.56 × 10^{-5}	6.59

TM + (jul)$_2$CH$^+$

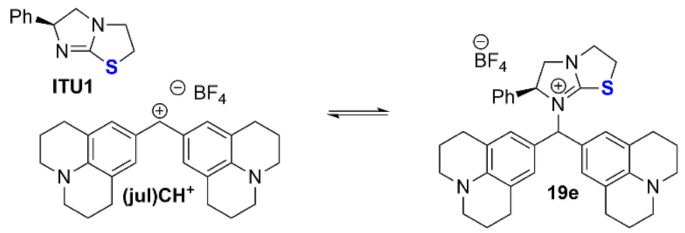

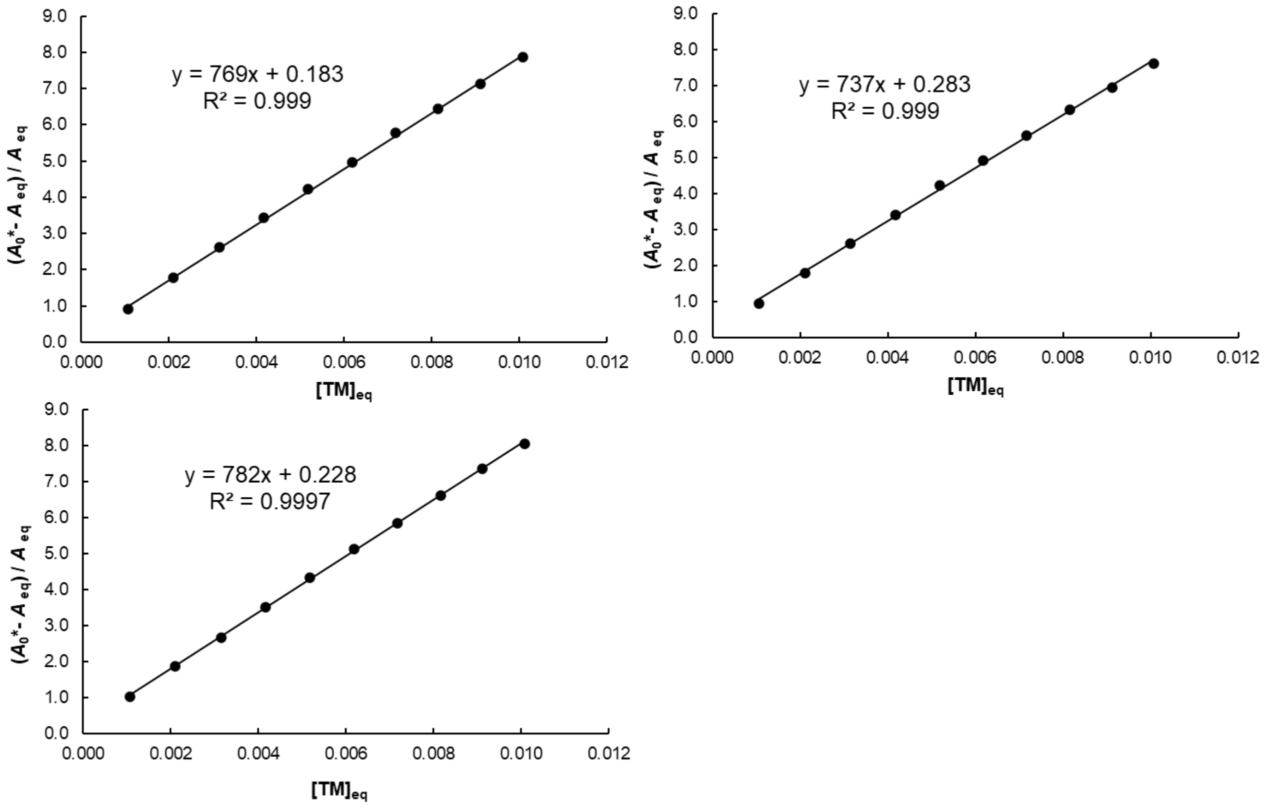

Figure S35: Linear regression plots for the determination of the equilibrium constants between TM (**ITU1**) and (jul)$_2$CH$^+$ (observed at 642 nm). ε(jul, 642 nm = 2.24 × 10^5 L mol^{-1} cm^{-1}). LSM-143

Table S38: Calculation of the equilibrium constant K for the reaction between TM (**ITU1**) and (jul)$_2$CH$^+$

	K (L mol^{-1})
Titration #1	7.69 × 10^2
Titration #2	7.37 × 10^2
Titration #3	7.82 × 10^2
**K_{av} =**	**(7.63 ± 0.19) × 10^2 L mol^{-1}**

Table S39: Determination of the equilibrium constant between TM (**ITU1**) and $(jul)_2CH^+$ (observed at 642 nm). ε(jul, 642 nm = 2.24×10^5 L mol^{-1} cm^{-1}). LSM-143

Titration	Step	$V_{tot,Nu}$ (mL)	V_{tot} (mL)	A_{eq}	$[jul]_0$ (mol L^{-1})	A_0*	A_0*– A_{eq}	$[TM]_0$ (mol L^{-1})	$[TM]_{eq}$ (mol L^{-1})	(A_0* – A_{eq})/A_{eq}
#1	0	0	16.8	1.09	1.01×10^{-5}	1.09				
	1	0.1	16.9	0.571	1.00×10^{-5}	1.09	0.516	1.06×10^{-3}	1.06×10^{-3}	0.904
	2	0.2	17.0	0.389	9.95×10^{-6}	1.08	0.691	2.11×10^{-3}	2.11×10^{-3}	1.78
	3	0.3	17.1	0.297	9.89×10^{-6}	1.07	0.777	3.15×10^{-3}	3.14×10^{-3}	2.62
	4	0.4	17.2	0.241	9.83×10^{-6}	1.07	0.826	4.17×10^{-3}	4.17×10^{-3}	3.43
	5	0.5	17.3	0.203	9.78×10^{-6}	1.06	0.859	5.19×10^{-3}	5.18×10^{-3}	4.23
	6	0.6	17.4	0.177	9.72×10^{-6}	1.06	0.879	6.19×10^{-3}	6.18×10^{-3}	4.98
	7	0.7	17.5	0.155	9.66×10^{-6}	1.05	0.894	7.18×10^{-3}	7.17×10^{-3}	5.77
	8	0.8	17.6	0.140	9.61×10^{-6}	1.04	0.903	8.16×10^{-3}	8.15×10^{-3}	6.45
	9	0.9	17.7	0.128	9.55×10^{-6}	1.04	0.910	9.13×10^{-3}	9.12×10^{-3}	7.14
	10	1	17.8	0.116	9.50×10^{-6}	1.03	0.915	1.01×10^{-2}	1.01×10^{-2}	7.86
#2	0	0	16.8	1.12	1.01×10^{-5}	1.12				
	1	0.1	16.9	0.572	1.00×10^{-5}	1.12	0.543	1.06×10^{-3}	1.06×10^{-3}	0.950
	2	0.2	17.0	0.397	9.95×10^{-6}	1.11	0.711	2.11×10^{-3}	2.11×10^{-3}	1.79
	3	0.3	17.1	0.305	9.89×10^{-6}	1.10	0.797	3.15×10^{-3}	3.14×10^{-3}	2.61
	4	0.4	17.2	0.249	9.83×10^{-6}	1.10	0.847	4.17×10^{-3}	4.17×10^{-3}	3.40
	5	0.5	17.3	0.209	9.78×10^{-6}	1.09	0.881	5.19×10^{-3}	5.18×10^{-3}	4.22
	6	0.6	17.4	0.183	9.72×10^{-6}	1.08	0.901	6.19×10^{-3}	6.18×10^{-3}	4.93
	7	0.7	17.5	0.163	9.66×10^{-6}	1.08	0.914	7.18×10^{-3}	7.17×10^{-3}	5.60
	8	0.8	17.6	0.146	9.61×10^{-6}	1.07	0.925	8.16×10^{-3}	8.15×10^{-3}	6.33
	9	0.9	17.7	0.134	9.55×10^{-6}	1.07	0.931	9.13×10^{-3}	9.12×10^{-3}	6.94
	10	1	17.8	0.123	9.50×10^{-6}	1.06	0.936	1.01×10^{-2}	1.01×10^{-2}	7.60
#3	0	0	16.8	1.13	1.01×10^{-5}	1.13				
	1	0.1	16.9	0.560	1.00×10^{-5}	1.13	0.565	1.06×10^{-3}	1.06×10^{-3}	1.01
	2	0.2	17.0	0.392	9.95×10^{-6}	1.12	0.727	2.11×10^{-3}	2.11×10^{-3}	1.86
	3	0.3	17.1	0.303	9.89×10^{-6}	1.11	0.809	3.15×10^{-3}	3.14×10^{-3}	2.67
	4	0.4	17.2	0.245	9.83×10^{-6}	1.11	0.861	4.17×10^{-3}	4.17×10^{-3}	3.51
	5	0.5	17.3	0.206	9.78×10^{-6}	1.10	0.893	5.19×10^{-3}	5.18×10^{-3}	4.34
	6	0.6	17.4	0.178	9.72×10^{-6}	1.09	0.915	6.19×10^{-3}	6.18×10^{-3}	5.13
	7	0.7	17.5	0.159	9.66×10^{-6}	1.09	0.928	7.18×10^{-3}	7.17×10^{-3}	5.85
	8	0.8	17.6	0.142	9.61×10^{-6}	1.08	0.939	8.16×10^{-3}	8.15×10^{-3}	6.61
	9	0.9	17.7	0.129	9.55×10^{-6}	1.07	0.946	9.13×10^{-3}	9.12×10^{-3}	7.35
	10	1	17.8	0.118	9.50×10^{-6}	1.07	0.950	1.01×10^{-2}	1.01×10^{-2}	8.05

BTM + $(jul)_2CH^+$

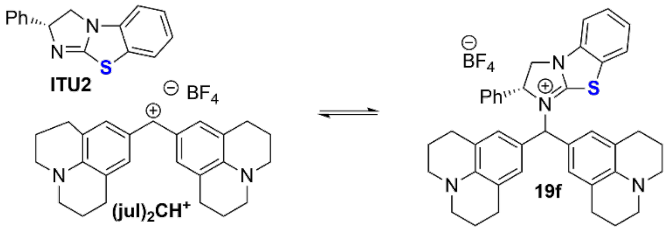

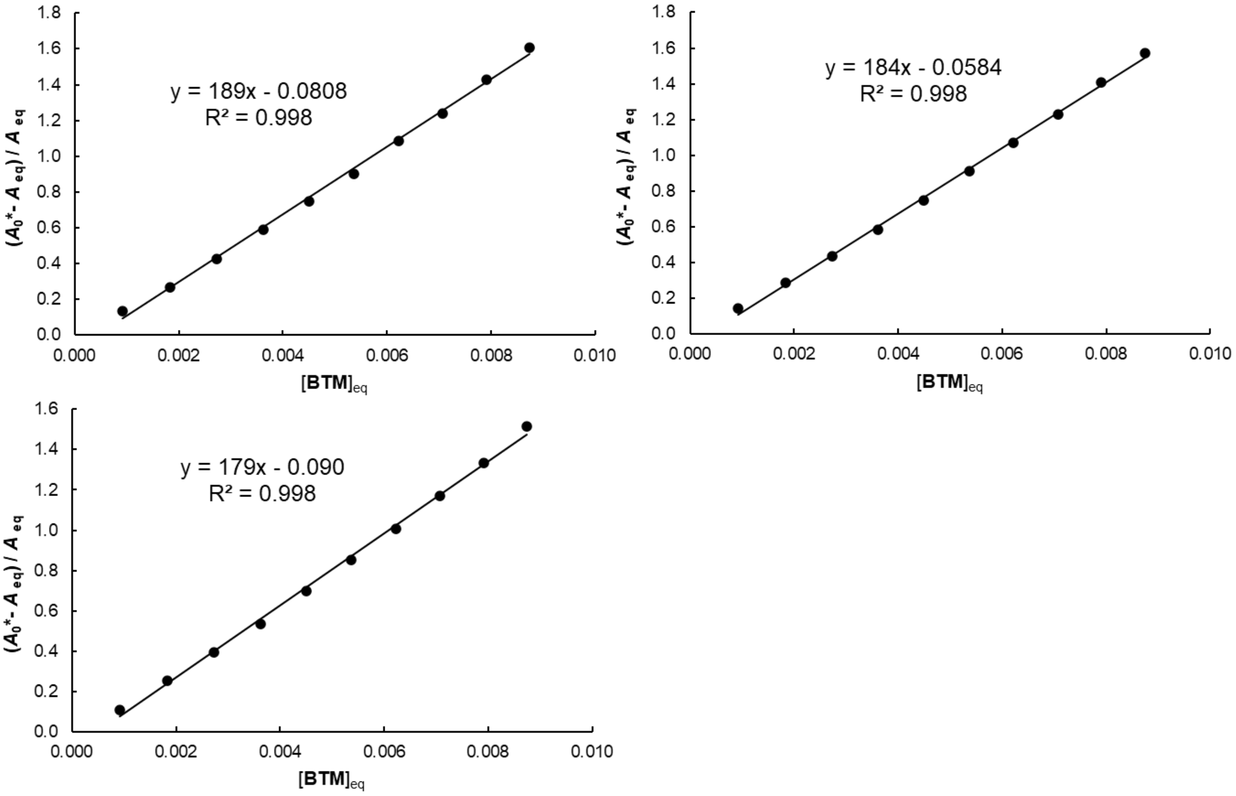

Figure S36: Linear regression plots for the determination of the equilibrium constants between BTM (**ITU2**) and $(jul)_2CH^+$ (monitored at 642 nm, ε(642 nm) = 2.24 × 10^5 L mol^{-1} cm^{-1}). LSM-142

Table S40: Calculation of the equilibrium constant *K*(20 °C) for the reaction between BTM (**ITU2**) and $(jul)_2CH^+$ BF_4^- in dichloromethane.

	K (L mol^{-1})
Titration #1	1.89 × 10^2
Titration #2	1.83 × 10^2
Titration #3	1.79 × 10^2
**K_{av} =**	**(1.84 ± 0.04) × 10^2 L mol^{-1}**

Table S41: Determination of the equilibrium constant between BTM (**ITU2**) and $(jul)_2CH^+$ BF_4^- (monitored at 642 nm, ε(642 nm) = 2.24 × 10^5 L mol^{-1} cm^{-1}). LSM-142

Titration	Step	$V_{tot,Nu}$ (mL)	V_{tot} (mL)	A_{eq}	$[jul]_0$ (mol L^{-1})	A_0*	A_0*– A_{eq}	$[BTM]_0$ (mol L^{-1})	$[BTM]_{eq}$ (mol L^{-1})	(A_0* – A_{eq})/A_{eq}
#1	0	0	16.7	1.16	1.02 × 10^{-5}	1.16				
	1	0.1	16.8	1.02	1.01 × 10^{-5}	1.15	0.136	9.22 × 10^{-4}	9.20 × 10^{-4}	0.133
	2	0.2	16.9	0.905	1.00 × 10^{-5}	1.15	0.242	1.83 × 10^{-3}	1.83 × 10^{-3}	0.268
	3	0.3	17.0	0.798	9.97 × 10^{-5}	1.14	0.342	2.73 × 10^{-3}	2.73 × 10^{-3}	0.429
	4	0.4	17.1	0.714	9.91 × 10^{-5}	1.13	0.420	3.62 × 10^{-3}	3.62 × 10^{-3}	0.588
	5	0.5	17.2	0.645	9.86 × 10^{-5}	1.13	0.482	4.50 × 10^{-3}	4.50 × 10^{-3}	0.747
	6	0.6	17.3	0.589	9.80 × 10^{-6}	1.12	0.532	5.37 × 10^{-3}	5.36 × 10^{-3}	0.90
	7	0.7	17.4	0.534	9.74 × 10^{-6}	1.11	0.580	6.23 × 10^{-3}	6.22 × 10^{-3}	1.09
	8	0.8	17.5	0.495	9.69 × 10^{-6}	1.11	0.612	7.08 × 10^{-3}	7.07 × 10^{-3}	1.24
	9	0.9	17.6	0.454	9.63 × 10^{-6}	1.10	0.647	7.92 × 10^{-3}	7.91 × 10^{-3}	1.43
	10	1	17.7	0.420	9.58 × 10^{-6}	1.09	0.675	8.75 × 10^{-3}	8.74 × 10^{-3}	1.61
#2	0	0	16.7	1.14	1.02 × 10^{-5}	1.14				
	1	0.1	16.8	0.992	1.01 × 10^{-5}	1.13	0.141	9.22 × 10^{-4}	9.20 × 10^{-4}	0.142
	2	0.2	16.9	0.874	1.00 × 10^{-5}	1.13	0.252	1.83 × 10^{-3}	1.83 × 10^{-3}	0.289
	3	0.3	17.0	0.781	9.97 × 10^{-5}	1.12	0.338	2.73 × 10^{-3}	2.73 × 10^{-3}	0.433
	4	0.4	17.1	0.703	9.91 × 10^{-5}	1.11	0.410	3.62 × 10^{-3}	3.62 × 10^{-3}	0.584
	5	0.5	17.2	0.633	9.86 × 10^{-5}	1.11	0.473	4.50 × 10^{-3}	4.50 × 10^{-3}	0.748
	6	0.6	17.3	0.575	9.80 × 10^{-6}	1.10	0.525	5.37 × 10^{-3}	5.36 × 10^{-3}	0.913
	7	0.7	17.4	0.528	9.74 × 10^{-6}	1.09	0.565	6.23 × 10^{-3}	6.22 × 10^{-3}	1.07
	8	0.8	17.5	0.487	9.69 × 10^{-6}	1.09	0.600	7.08 × 10^{-3}	7.07 × 10^{-3}	1.23
	9	0.9	17.6	0.449	9.63 × 10^{-6}	1.08	0.632	7.92 × 10^{-3}	7.91 × 10^{-3}	1.41
	10	1	17.7	0.418	9.58 × 10^{-6}	1.07	0.657	8.75 × 10^{-3}	8.74 × 10^{-3}	1.57
#3	0	0	16.7	1.12	1.02 × 10^{-5}	1.12				
	1	0.1	16.8	1.01	1.01 × 10^{-5}	1.12	0.107	9.22 × 10^{-4}	9.20 × 10^{-4}	0.106
	2	0.2	16.9	0.888	1.00 × 10^{-5}	1.11	0.223	1.83 × 10^{-3}	1.83 × 10^{-3}	0.251
	3	0.3	17.0	0.792	9.97 × 10^{-5}	1.10	0.312	2.73 × 10^{-3}	2.73 × 10^{-3}	0.394
	4	0.4	17.1	0.715	9.91 × 10^{-5}	1.10	0.383	3.62 × 10^{-3}	3.62 × 10^{-3}	0.536
	5	0.5	17.2	0.643	9.86 × 10^{-5}	1.09	0.449	4.50 × 10^{-3}	4.50 × 10^{-3}	0.699
	6	0.6	17.3	0.586	9.80 × 10^{-6}	1.09	0.499	5.37 × 10^{-3}	5.36 × 10^{-3}	0.851
	7	0.7	17.4	0.538	9.74 × 10^{-6}	1.08	0.541	6.23 × 10^{-3}	6.22 × 10^{-3}	1.01
	8	0.8	17.5	0.494	9.69 × 10^{-6}	1.07	0.579	7.08 × 10^{-3}	7.07 × 10^{-3}	1.17
	9	0.9	17.6	0.457	9.63 × 10^{-6}	1.07	0.609	7.92 × 10^{-3}	7.91 × 10^{-3}	1.33
	10	1	17.7	0.422	9.58 × 10^{-6}	1.06	0.639	8.75 × 10^{-3}	8.74 × 10^{-3}	1.52

SeBTM + $(pyr)_2CH^+$

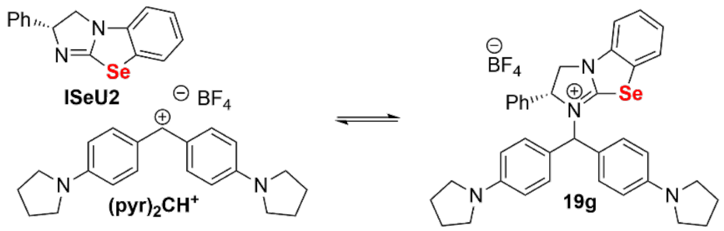

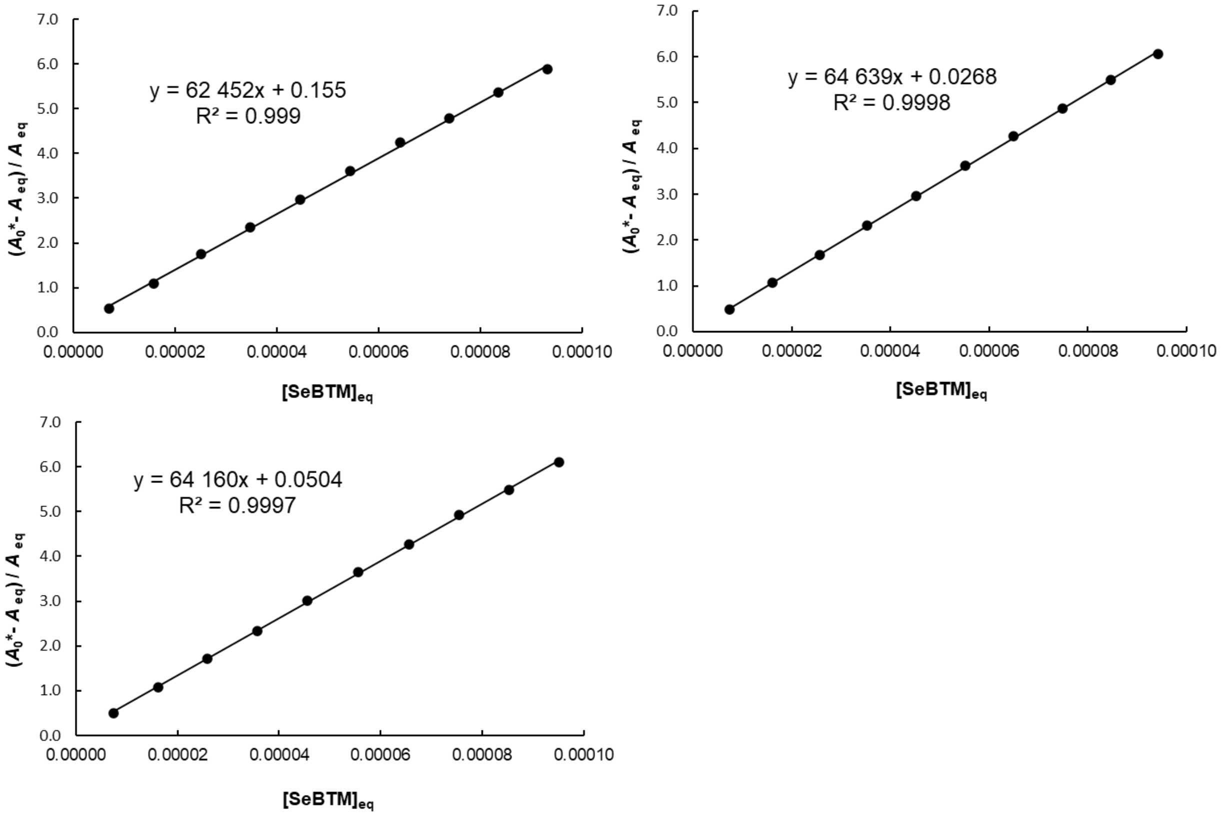

Figure S37: Linear regression plots for the determination of the equilibrium constants between SeBTM (**ISeU2**) and $(pyr)_2CH^+$ (observed at 620 nm). ε(pyr, 620 nm = 1.74 × 10^5 L mol^{-1} cm^{-1}). LSM-46

Table S42: Calculation of the equilibrium constant *K* for the reaction between SeBTM (**ISeU2**) and $(pyr)_2CH^+$

	K (L mol^{-1})
Titration #1	6.25 × 10^4
Titration #2	6.46 × 10^4
Titration #3	6.42 × 10^4
**K_{av} =**	**(6.38 ± 0.09) × 10^4 L mol^{-1}**

Table S43: Determination of the equilibrium constant between SeBTM (**ISeU2**) and $(pyr)_2CH^+$ (observed at 620 nm). ε(pyr, 620 nm = 1.74 × 10^5 L mol^{-1} cm^{-1}). LSM-46

Titration	Step	$V_{tot,Nu}$ (mL)	V_{tot} (mL)	A_{eq}	$[pyr]_0$ (mol L^{-1})	A_0*	A_0*– A_{eq}	$[SeBTM]_0$ (mol L^{-1})	$[SeBTM]_{eq}$ (mol L^{-1})	(A_0* – A_{eq})/A_{eq}
#1	0	0	17.4	0.904	9.88 × 10^{-6}	0.904				
	1	0.1	17.5	0.582	9.82 × 10^{-6}	0.899	0.317	1.07 × 10^{-5}	7.03 × 10^{-6}	0.544
	2	0.2	17.6	0.429	9.76 × 10^{-6}	0.894	0.465	2.12 × 10^{-5}	1.59 × 10^{-5}	1.08
	3	0.3	17.7	0.322	9.71 × 10^{-6}	0.889	0.567	3.17 × 10^{-5}	2.52 × 10^{-5}	1.76
	4	0.4	17.8	0.263	9.66 × 10^{-6}	0.884	0.620	4.20 × 10^{-5}	3.49 × 10^{-5}	2.36
	5	0.5	17.9	0.222	9.60 × 10^{-6}	0.879	0.657	5.22 × 10^{-5}	4.46 × 10^{-5}	2.96
	6	0.6	18.0	0.190	9.55 × 10^{-6}	0.874	0.684	6.23 × 10^{-5}	5.44 × 10^{-5}	3.61
	7	0.7	18.1	0.166	9.50 × 10^{-6}	0.869	0.703	7.23 × 10^{-5}	6.42 × 10^{-5}	4.24
	8	0.8	18.2	0.149	9.44 × 10^{-6}	0.864	0.715	8.22 × 10^{-5}	7.39 × 10^{-5}	4.79
	9	0.9	18.3	0.135	9.39 × 10^{-6}	0.860	0.724	9.19 × 10^{-5}	8.36 × 10^{-5}	5.36
	10	1	18.4	0.124	9.34 × 10^{-6}	0.855	0.731	1.02 × 10^{-4}	9.32 × 10^{-5}	5.88
#2	0	0	17.2	0.907	9.99 × 10^{-6}	0.907				
	1	0.1	17.3	0.609	9.94 × 10^{-6}	0.902	0.293	1.08 × 10^{-5}	7.43 × 10^{-6}	0.481
	2	0.2	17.4	0.434	9.88 × 10^{-6}	0.896	0.462	2.15 × 10^{-5}	1.62 × 10^{-5}	1.07
	3	0.3	17.5	0.334	9.82 × 10^{-6}	0.891	0.557	3.20 × 10^{-5}	2.56 × 10^{-5}	1.67
	4	0.4	17.6	0.266	9.77 × 10^{-6}	0.886	0.620	4.25 × 10^{-5}	3.53 × 10^{-5}	2.33
	5	0.5	17.7	0.222	9.71 × 10^{-6}	0.881	0.659	5.28 × 10^{-5}	4.52 × 10^{-5}	2.96
	6	0.6	17.8	0.190	9.66 × 10^{-6}	0.876	0.686	6.30 × 10^{-5}	5.51 × 10^{-5}	3.62
	7	0.7	17.9	0.166	9.60 × 10^{-6}	0.871	0.706	7.31 × 10^{-5}	6.50 × 10^{-5}	4.27
	8	0.8	18.0	0.148	9.55 × 10^{-6}	0.867	0.719	8.31 × 10^{-5}	7.48 × 10^{-5}	4.87
	9	0.9	18.1	0.133	9.50 × 10^{-6}	0.862	0.729	9.29 × 10^{-5}	8.46 × 10^{-5}	5.50
	10	1	18.2	0.121	9.44 × 10^{-6}	0.857	0.736	1.03 × 10^{-4}	9.42 × 10^{-5}	6.06
#3	0	0	17.1	0.904	1.01 × 10^{-5}	0.904				
	1	0.1	17.2	0.602	1.00 × 10^{-5}	0.898	0.296	1.09 × 10^{-5}	7.47 × 10^{-6}	0.493
	2	0.2	17.3	0.428	9.95 × 10^{-6}	0.893	0.465	2.16 × 10^{-5}	1.63 × 10^{-5}	1.09
	3	0.3	17.4	0.328	9.89 × 10^{-6}	0.888	0.560	3.23 × 10^{-5}	2.58 × 10^{-5}	1.71
	4	0.4	17.5	0.265	9.84 × 10^{-6}	0.883	0.618	4.28 × 10^{-5}	3.57 × 10^{-5}	2.34
	5	0.5	17.6	0.219	9.78 × 10^{-6}	0.878	0.659	5.32 × 10^{-5}	4.56 × 10^{-5}	3.01
	6	0.6	17.7	0.188	9.73 × 10^{-6}	0.873	0.685	6.35 × 10^{-5}	5.56 × 10^{-5}	3.65
	7	0.7	17.8	0.164	9.67 × 10^{-6}	0.868	0.704	7.36 × 10^{-5}	6.55 × 10^{-5}	4.28
	8	0.8	17.9	0.146	9.62 × 10^{-6}	0.863	0.717	8.37 × 10^{-5}	7.54 × 10^{-5}	4.92
	9	0.9	18.0	0.132	9.56 × 10^{-6}	0.858	0.726	9.36 × 10^{-5}	8.53 × 10^{-5}	5.50
	10	1	18.1	0.120	9.51 × 10^{-6}	0.854	0.733	1.03 × 10^{-4}	9.50 × 10^{-5}	6.09

SeBTM + $(thq)_2CH^+$

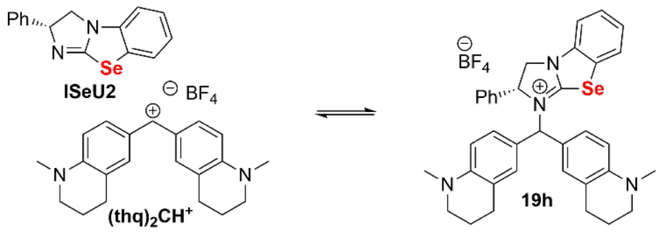

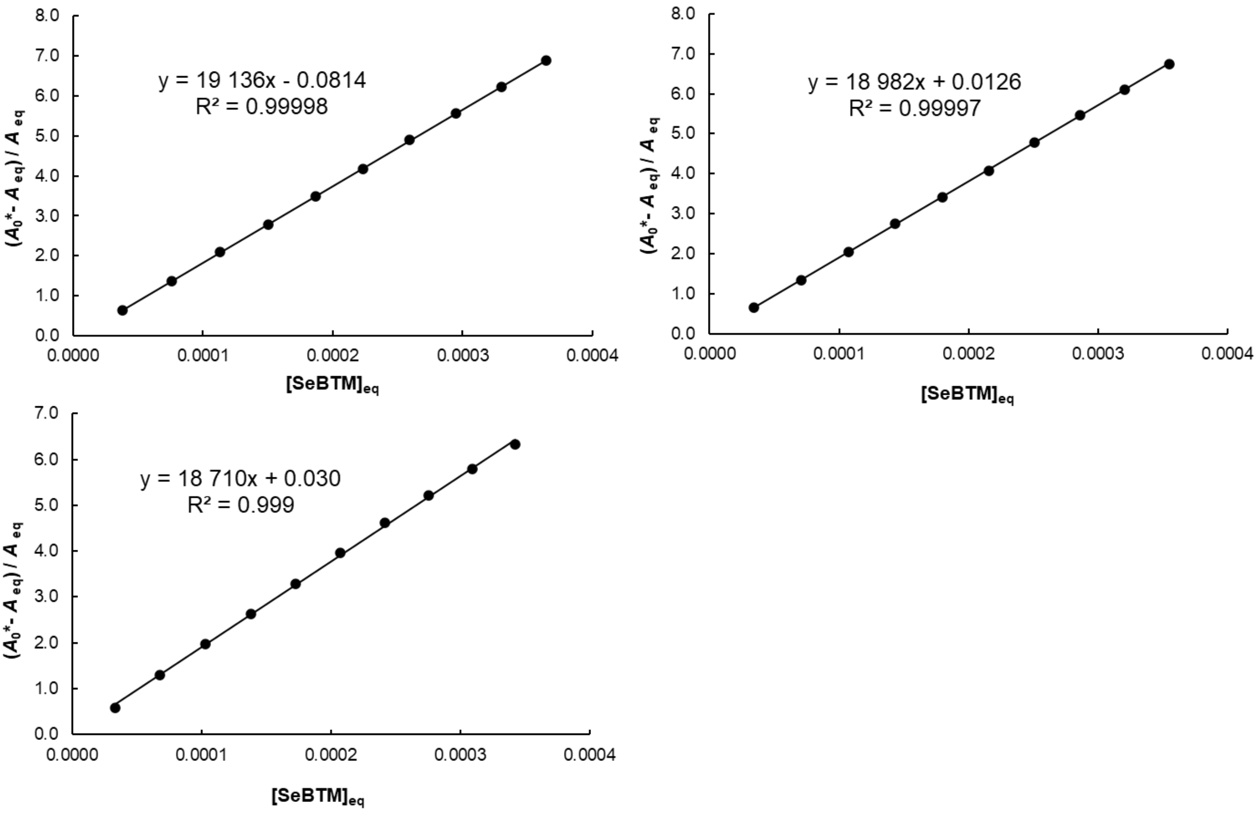

Figure S38: Linear regression plots for the determination of the equilibrium constants between SeBTM (**ISeU2**) and $(thq)_2CH^+$ (observed at 628 nm). ε(thq, 628 nm = 1.78×10^5 L mol^{-1} cm^{-1}). LSM-45

Table S44: Calculation of the equilibrium constant K for the reaction between SeBTM (**ISeU2**) and $(thq)_2CH^+$

	K (L mol^{-1})
Titration #1	1.91×10^4
Titration #2	1.90×10^4
Titration #3	1.87×10^4
**K_{av} =**	**$(1.89 \pm 0.02) \times 10^4$ L mol^{-1}**

Table S45: Determination of the equilibrium constant between SeBTM (**ISeU2**) and $(thq)_2CH^+$ (observed at 628 nm). ε(thq, 628 nm = 1.78×10^5 L mol^{-1} cm^{-1}). LSM-45

Titration	Step	$V_{tot,Nu}$ (mL)	V_{tot} (mL)	A_{eq}	$[thq]_0$ (mol L^{-1})	A_0*	A_0*– A_{eq}	$[SeBTM]_0$ (mol L^{-1})	$[SeBTM]_{eq}$ (mol L^{-1})	(A_0* – A_{eq})/A_{eq}
#1	0	0	17.1	0.877	9.91×10^{-6}	0.877				
	1	0.1	17.2	0.528	9.85×10^{-6}	0.872	0.344	3.84×10^{-5}	3.81×10^{-5}	0.651
	2	0.2	17.3	0.366	9.79×10^{-6}	0.867	0.501	7.65×10^{-5}	7.59×10^{-5}	1.37
	3	0.3	17.4	0.279	9.74×10^{-6}	0.862	0.583	1.14×10^{-4}	1.13×10^{-4}	2.09
	4	0.4	17.5	0.226	9.68×10^{-6}	0.857	0.631	1.51×10^{-4}	1.50×10^{-4}	2.79
	5	0.5	17.6	0.190	9.63×10^{-6}	0.852	0.663	1.88×10^{-4}	1.87×10^{-4}	3.50
	6	0.6	17.7	0.164	9.57×10^{-6}	0.847	0.684	2.24×10^{-4}	2.23×10^{-4}	4.18
	7	0.7	17.8	0.143	9.52×10^{-6}	0.843	0.700	2.60×10^{-4}	2.59×10^{-4}	4.90
	8	0.8	17.9	0.128	9.47×10^{-6}	0.838	0.710	2.96×10^{-4}	2.95×10^{-4}	5.56
	9	0.9	18.0	0.115	9.41×10^{-6}	0.833	0.718	3.31×10^{-4}	3.30×10^{-4}	6.23
	10	1	18.1	0.105	9.36×10^{-6}	0.829	0.724	3.65×10^{-4}	3.65×10^{-4}	6.89
#2	0	0	17.2	0.855	9.85×10^{-6}	0.855				
	1	0.1	17.3	0.510	9.80×10^{-6}	0.850	0.339	3.82×10^{-5}	3.44×10^{-5}	0.665
	2	0.2	17.4	0.360	9.74×10^{-6}	0.845	0.484	7.60×10^{-5}	7.06×10^{-5}	1.34
	3	0.3	17.5	0.276	9.68×10^{-6}	0.840	0.564	1.13×10^{-4}	1.07×10^{-4}	2.05
	4	0.4	17.6	0.223	9.63×10^{-6}	0.835	0.612	1.50×10^{-4}	1.43×10^{-4}	2.75
	5	0.5	17.7	0.188	9.57×10^{-6}	0.830	0.643	1.87×10^{-4}	1.80×10^{-4}	3.42
	6	0.6	17.8	0.163	9.52×10^{-6}	0.826	0.663	2.23×10^{-4}	2.15×10^{-4}	4.08
	7	0.7	17.9	0.142	9.47×10^{-6}	0.821	0.679	2.59×10^{-4}	2.51×10^{-4}	4.79
	8	0.8	18.0	0.127	9.41×10^{-6}	0.817	0.690	2.94×10^{-4}	2.86×10^{-4}	5.45
	9	0.9	18.1	0.114	9.36×10^{-6}	0.812	0.698	3.29×10^{-4}	3.21×10^{-4}	6.10
	10	1	18.2	0.104	9.31×10^{-6}	0.808	0.703	3.63×10^{-4}	3.55×10^{-4}	6.75
#3	0	0	17.0	0.870	9.94×10^{-6}	0.870	0			
	1	0.1	17.1	0.550	9.89×10^{-6}	0.865	0.315	3.69×10^{-5}	3.33×10^{-5}	0.573
	2	0.2	17.2	0.375	9.83×10^{-6}	0.860	0.485	7.33×10^{-5}	6.78×10^{-5}	1.29
	3	0.3	17.3	0.288	9.77×10^{-6}	0.855	0.567	1.09×10^{-4}	1.03×10^{-4}	1.97
	4	0.4	17.4	0.234	9.72×10^{-6}	0.850	0.616	1.45×10^{-4}	1.38×10^{-4}	2.63
	5	0.5	17.5	0.198	9.66×10^{-6}	0.846	0.648	1.80×10^{-4}	1.73×10^{-4}	3.28
	6	0.6	17.6	0.169	9.61×10^{-6}	0.841	0.671	2.15×10^{-4}	2.07×10^{-4}	3.96
	7	0.7	17.7	0.149	9.55×10^{-6}	0.836	0.687	2.49×10^{-4}	2.42×10^{-4}	4.61
	8	0.8	17.8	0.134	9.50×10^{-6}	0.831	0.698	2.83×10^{-4}	2.75×10^{-4}	5.22
	9	0.9	17.9	0.122	9.45×10^{-6}	0.827	0.705	3.17×10^{-4}	3.09×10^{-4}	5.79
	10	1	18.0	0.112	9.39×10^{-6}	0.822	0.710	3.50×10^{-4}	3.42×10^{-4}	6.33

SeDHPB + $(thq)_2CH^+$

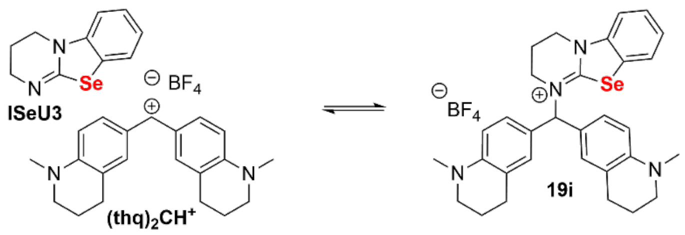

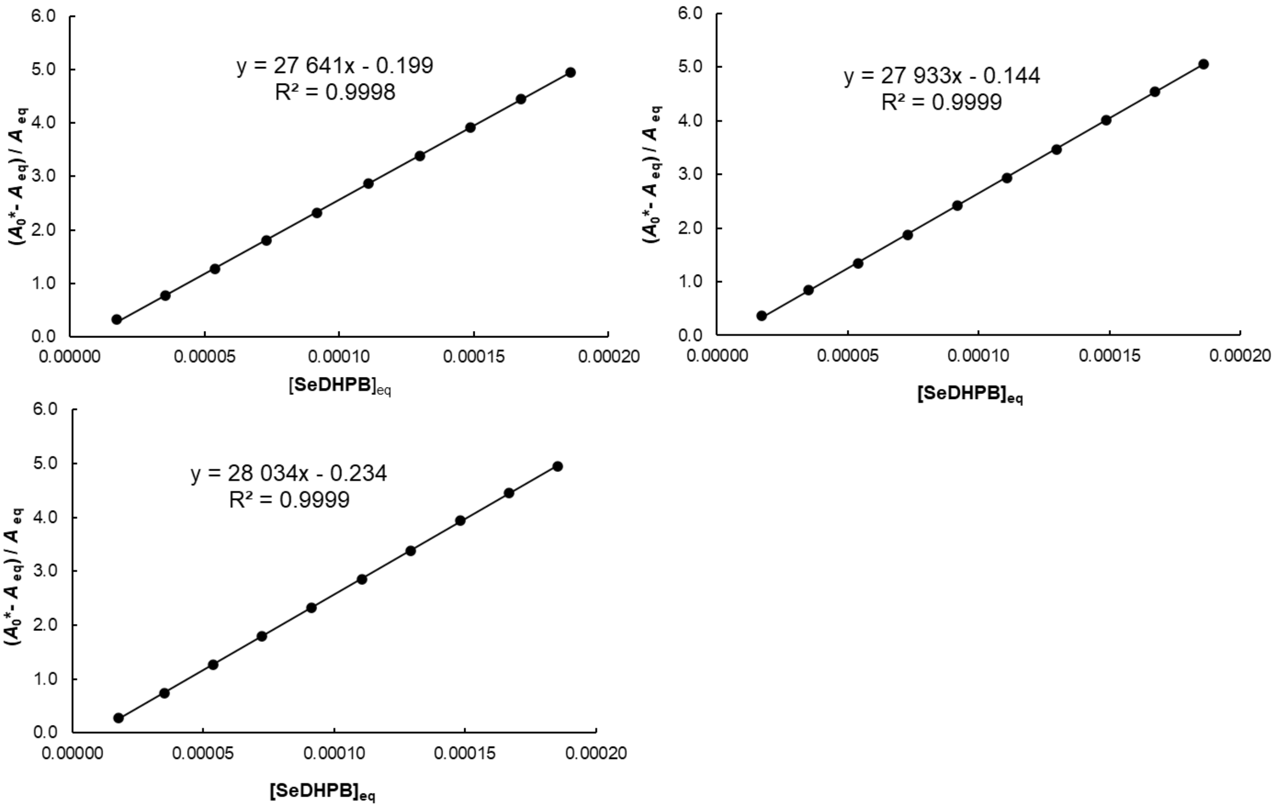

Figure S39: Linear regression plots for the determination of the equilibrium constants between SeDHPB (**ISeU3**) and $(thq)_2CH^+$ (observed at 628 nm). ε(thq, 628 nm = 1.78 × 10^5 L mol^{-1} cm^{-1}). LSM-47

Table S46: Calculation of the equilibrium constant K for the reaction between SeDHPB (**ISeU3**) and $(thq)_2CH^+$

	K (L mol^{-1})
Titration #1	2.76 × 10^4
Titration #2	2.79 × 10^4
Titration #3	2.80 × 10^4
**K_{av} =**	**(2.79 ± 0.02) × 10^4 L mol^{-1}**

Table S47: Determination of the equilibrium constant between SeDHPB (**lSeU3**) and $(thq)_2CH^+$ (observed at 628 nm). ε(thq, 628 nm = 1.78 × 10^5 L mol^{-1} cm^{-1}). LSM-47

Titration	Step	$V_{tot,Nu}$ (mL)	V_{tot} (mL)	A_{eq}	$[thq]_0$ (mol L^{-1})	A_0*	A_0*– A_{eq}	$[SeDHPB]_0$ (mol L^{-1})	$[SeDHPB]_{eq}$ (mol L^{-1})	(A_0* – A_{eq})/A_{eq}
#1	0	0	17.0	1.21	1.01 × 10^{-5}	1.206				
	1	0.1	17.1	0.904	1.01 × 10^{-5}	1.199	0.295	2.07 × 10^{-5}	1.74 × 10^{-5}	0.326
	2	0.2	17.2	0.672	1.00 × 10^{-5}	1.192	0.519	4.11 × 10^{-5}	3.53 × 10^{-5}	0.773
	3	0.3	17.3	0.522	9.95 × 10^{-6}	1.185	0.663	6.13 × 10^{-5}	5.39 × 10^{-5}	1.27
	4	0.4	17.4	0.421	9.89 × 10^{-6}	1.178	0.757	8.13 × 10^{-5}	7.28 × 10^{-5}	1.80
	5	0.5	17.5	0.353	9.84 × 10^{-6}	1.171	0.818	1.01 × 10^{-4}	9.19 × 10^{-5}	2.32
	6	0.6	17.6	0.301	9.78 × 10^{-6}	1.165	0.863	1.21 × 10^{-4}	1.11 × 10^{-4}	2.87
	7	0.7	17.7	0.264	9.73 × 10^{-6}	1.158	0.894	1.40 × 10^{-4}	1.30 × 10^{-4}	3.38
	8	0.8	17.8	0.234	9.67 × 10^{-6}	1.152	0.917	1.59 × 10^{-4}	1.49 × 10^{-4}	3.92
	9	0.9	17.9	0.210	9.62 × 10^{-6}	1.145	0.935	1.78 × 10^{-4}	1.67 × 10^{-4}	4.45
	10	1	18.0	0.192	9.56 × 10^{-6}	1.139	0.947	1.97 × 10^{-4}	1.86 × 10^{-4}	4.94
#2	0	0	17.0	1.19	1.01 × 10^{-5}	1.188				
	1	0.1	17.1	0.866	1.01 × 10^{-5}	1.181	0.315	2.07 × 10^{-5}	1.71 × 10^{-5}	0.364
	2	0.2	17.2	0.636	1.00 × 10^{-5}	1.174	0.538	4.11 × 10^{-5}	3.50 × 10^{-5}	0.845
	3	0.3	17.3	0.499	9.94 × 10^{-6}	1.167	0.668	6.13 × 10^{-5}	5.38 × 10^{-5}	1.34
	4	0.4	17.4	0.404	9.88 × 10^{-6}	1.160	0.756	8.12 × 10^{-5}	7.27 × 10^{-5}	1.87
	5	0.5	17.5	0.338	9.82 × 10^{-6}	1.154	0.816	1.01 × 10^{-4}	9.18 × 10^{-5}	2.42
	6	0.6	17.6	0.292	9.77 × 10^{-6}	1.147	0.855	1.20 × 10^{-4}	1.11 × 10^{-4}	2.93
	7	0.7	17.7	0.256	9.71 × 10^{-6}	1.141	0.885	1.40 × 10^{-4}	1.30 × 10^{-4}	3.46
	8	0.8	17.8	0.226	9.66 × 10^{-6}	1.134	0.908	1.59 × 10^{-4}	1.49 × 10^{-4}	4.02
	9	0.9	17.9	0.203	9.61 × 10^{-6}	1.128	0.925	1.78 × 10^{-4}	1.67 × 10^{-4}	4.55
	10	1	18.0	0.185	9.55 × 10^{-6}	1.122	0.937	1.96 × 10^{-4}	1.86 × 10^{-4}	5.05
#3	0	0	17.1	1.21	1.01 × 10^{-5}	1.213				
	1	0.1	17.2	0.943	1.00 × 10^{-5}	1.206	0.264	2.06 × 10^{-5}	1.77 × 10^{-5}	0.280
	2	0.2	17.3	0.688	9.98 × 10^{-6}	1.199	0.511	4.10 × 10^{-5}	3.53 × 10^{-5}	0.742
	3	0.3	17.4	0.525	9.92 × 10^{-6}	1.192	0.667	6.12 × 10^{-5}	5.37 × 10^{-5}	1.27
	4	0.4	17.5	0.424	9.86 × 10^{-6}	1.186	0.762	8.11 × 10^{-5}	7.25 × 10^{-5}	1.80
	5	0.5	17.6	0.355	9.81 × 10^{-6}	1.179	0.824	1.01 × 10^{-4}	9.15 × 10^{-5}	2.32
	6	0.6	17.7	0.304	9.75 × 10^{-6}	1.172	0.868	1.20 × 10^{-4}	1.10 × 10^{-4}	2.85
	7	0.7	17.8	0.266	9.70 × 10^{-6}	1.166	0.900	1.39 × 10^{-4}	1.29 × 10^{-4}	3.39
	8	0.8	17.9	0.235	9.64 × 10^{-6}	1.159	0.924	1.59 × 10^{-4}	1.48 × 10^{-4}	3.94
	9	0.9	18.0	0.212	9.59 × 10^{-6}	1.153	0.941	1.77 × 10^{-4}	1.67 × 10^{-4}	4.45
	10	1	18.1	0.193	9.54 × 10^{-6}	1.146	0.954	1.96 × 10^{-4}	1.85 × 10^{-4}	4.95

SeDHPB + (lil)2CH+

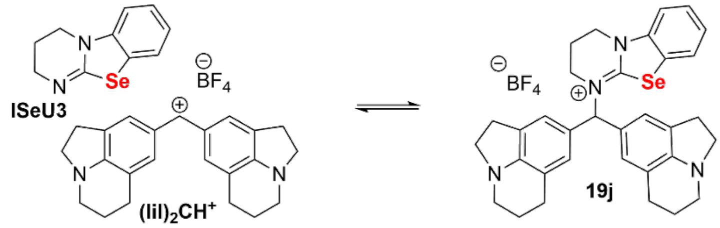

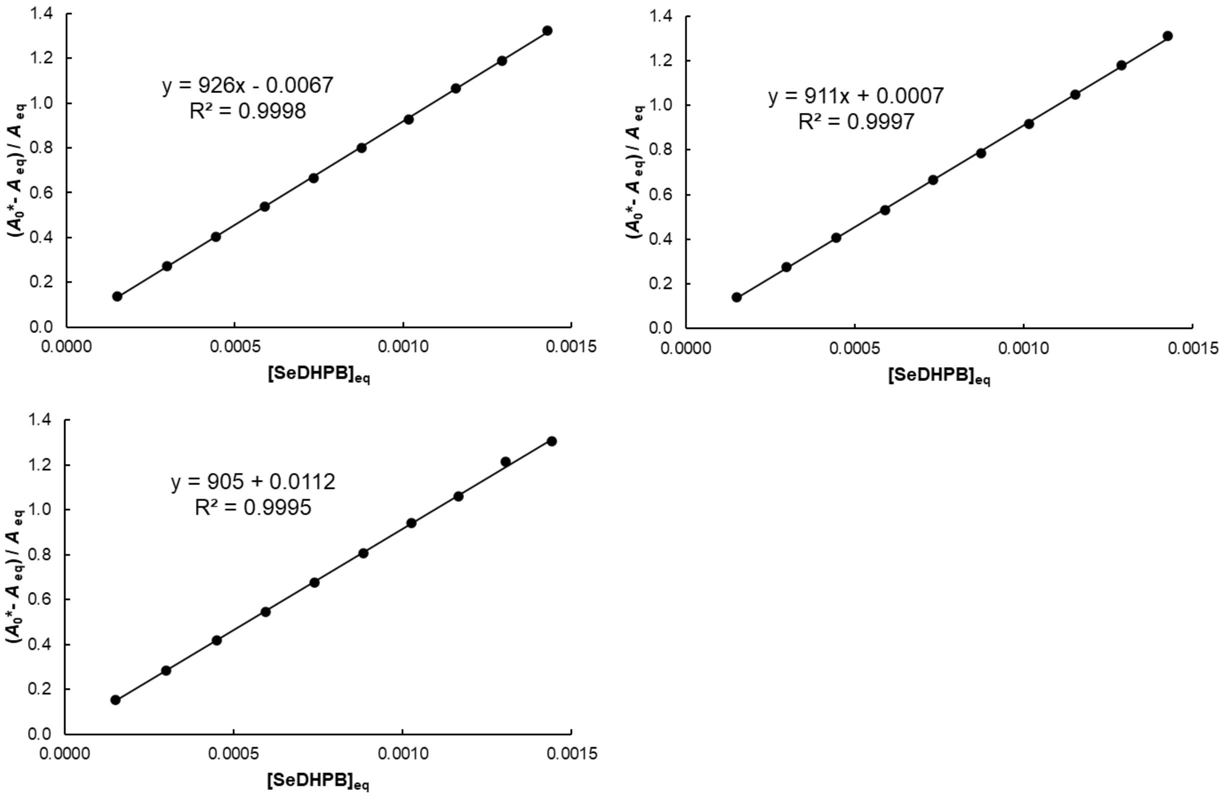

Figure S40: Linear regression plots for the determination of the equilibrium constants between SeDHPB (**ISeU3**) and $(lil)_2CH^+$ (observed at 639 nm). ε(lil, 639 nm = 1.59 × 10^5 L mol^{-1} cm^{-1}). LSM-40

Table S48: Calculation of the equilibrium constant *K* for the reaction between SeDHPB (**ISeU3**) and $(lil)_2CH^+$

	K (L mol^{-1})
Titration #1	9.26 × 10^2
Titration #2	9.11 × 10^2
Titration #3	9.05 × 10^2
**K_{av} =**	**(9.14 ± 0.09) × 10^2 L mol^{-1}**

Table S49: Determination of the equilibrium constant between SeDHPB (**ISeU3**) and $(lil)_2CH^+$ (observed at 639 nm). ε(lil, 639 nm = 1.59 × 10^5 L mol^{-1} cm^{-1}). LSM-40

Titration	Step	$V_{tot,Nu}$ (mL)	V_{tot} (mL)	A_{eq}	$[lil]_0$ (mol L^{-1})	A_0*	A_0*– A_{eq}	$[SeDHPB]_0$ (mol L^{-1})	$[SeDHPB]_{eq}$ (mol L^{-1})	(A_0* – A_{eq})/A_{eq}
#1	0	0	17.1	0.673	1.01 × 10^{-5}	0.673				
	1	0.1	17.2	0.588	1.01 × 10^{-5}	0.669	0.081	1.51 × 10^{-4}	1.50 × 10^{-4}	0.138
	2	0.2	17.3	0.523	1.00 × 10^{-5}	0.665	0.143	3.00 × 10^{-4}	2.98 × 10^{-4}	0.273
	3	0.3	17.4	0.472	9.96 × 10^{-6}	0.661	0.190	4.47 × 10^{-4}	4.45 × 10^{-4}	0.403
	4	0.4	17.5	0.427	9.91 × 10^{-6}	0.658	0.230	5.93 × 10^{-4}	5.90 × 10^{-4}	0.539
	5	0.5	17.6	0.392	9.85 × 10^{-6}	0.654	0.262	7.37 × 10^{-4}	7.34 × 10^{-4}	0.667
	6	0.6	17.7	0.361	9.79 × 10^{-6}	0.650	0.289	8.80 × 10^{-4}	8.76 × 10^{-4}	0.802
	7	0.7	17.8	0.335	9.74 × 10^{-6}	0.646	0.311	1.02 × 10^{-3}	1.02 × 10^{-3}	0.929
	8	0.8	17.9	0.311	9.68 × 10^{-6}	0.643	0.332	1.16 × 10^{-3}	1.16 × 10^{-3}	1.07
	9	0.9	18.0	0.292	9.63 × 10^{-6}	0.639	0.347	1.30 × 10^{-3}	1.29 × 10^{-3}	1.19
	10	1	18.1	0.274	9.58 × 10^{-6}	0.636	0.362	1.43 × 10^{-3}	1.43 × 10^{-3}	1.32
#2	0	0	17.1	0.657	1.01 × 10^{-5}	0.657				
	1	0.1	17.2	0.572	1.01 × 10^{-5}	0.653	0.082	1.51 × 10^{-4}	1.50 × 10^{-4}	0.143
	2	0.2	17.3	0.509	1.00 × 10^{-5}	0.650	0.140	2.99 × 10^{-4}	2.98 × 10^{-4}	0.276
	3	0.3	17.4	0.459	9.94 × 10^{-6}	0.646	0.187	4.46 × 10^{-4}	4.44 × 10^{-4}	0.408
	4	0.4	17.5	0.419	9.89 × 10^{-6}	0.642	0.223	5.92 × 10^{-4}	5.89 × 10^{-4}	0.531
	5	0.5	17.6	0.383	9.83 × 10^{-6}	0.639	0.256	7.36 × 10^{-4}	7.32 × 10^{-4}	0.668
	6	0.6	17.7	0.356	9.77 × 10^{-6}	0.635	0.279	8.78 × 10^{-4}	8.74 × 10^{-4}	0.786
	7	0.7	17.8	0.329	9.72 × 10^{-6}	0.631	0.302	1.02 × 10^{-3}	1.01 × 10^{-3}	0.919
	8	0.8	17.9	0.307	9.66 × 10^{-6}	0.628	0.321	1.16 × 10^{-3}	1.15 × 10^{-3}	1.05
	9	0.9	18.0	0.287	9.61 × 10^{-6}	0.624	0.338	1.29 × 10^{-3}	1.29 × 10^{-3}	1.18
	10	1	18.1	0.269	9.56 × 10^{-6}	0.621	0.352	1.43 × 10^{-3}	1.43 × 10^{-3}	1.31
#3	0	0	16.9	0.653	1.02 × 10^{-5}	0.653				
	1	0.1	17.0	0.562	1.02 × 10^{-5}	0.649	0.087	1.52 × 10^{-4}	1.51 × 10^{-4}	0.155
	2	0.2	17.1	0.503	1.01 × 10^{-5}	0.645	0.142	3.02 × 10^{-4}	3.01 × 10^{-4}	0.283
	3	0.3	17.2	0.453	1.00 × 10^{-5}	0.642	0.189	4.51 × 10^{-4}	4.49 × 10^{-4}	0.417
	4	0.4	17.3	0.412	9.99 × 10^{-6}	0.638	0.226	5.98 × 10^{-4}	5.95 × 10^{-4}	0.547
	5	0.5	17.4	0.378	9.93 × 10^{-6}	0.634	0.256	7.43 × 10^{-4}	7.40 × 10^{-4}	0.677
	6	0.6	17.5	0.349	9.87 × 10^{-6}	0.631	0.281	8.87 × 10^{-4}	8.83 × 10^{-4}	0.805
	7	0.7	17.6	0.323	9.82 × 10^{-6}	0.627	0.304	1.03 × 10^{-3}	1.02 × 10^{-3}	0.940
	8	0.8	17.7	0.303	9.76 × 10^{-6}	0.624	0.321	1.17 × 10^{-3}	1.16 × 10^{-3}	1.06
	9	0.9	17.8	0.280	9.71 × 10^{-6}	0.620	0.340	1.31 × 10^{-3}	1.30 × 10^{-3}	1.21
	10	1	17.9	0.267	9.67 × 10^{-6}	0.617	0.349	1.44 × 10^{-3}	1.44 × 10^{-3}	1.31

<u>SeHBTM + $(pyr)_2CH^+$</u>

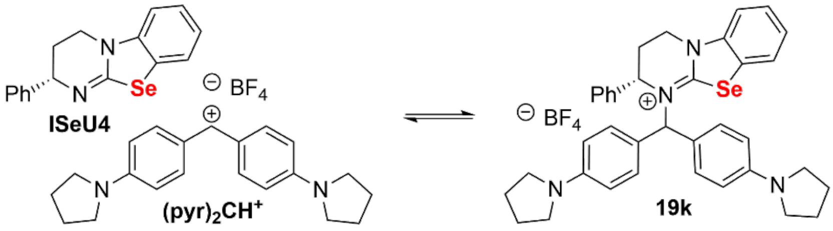

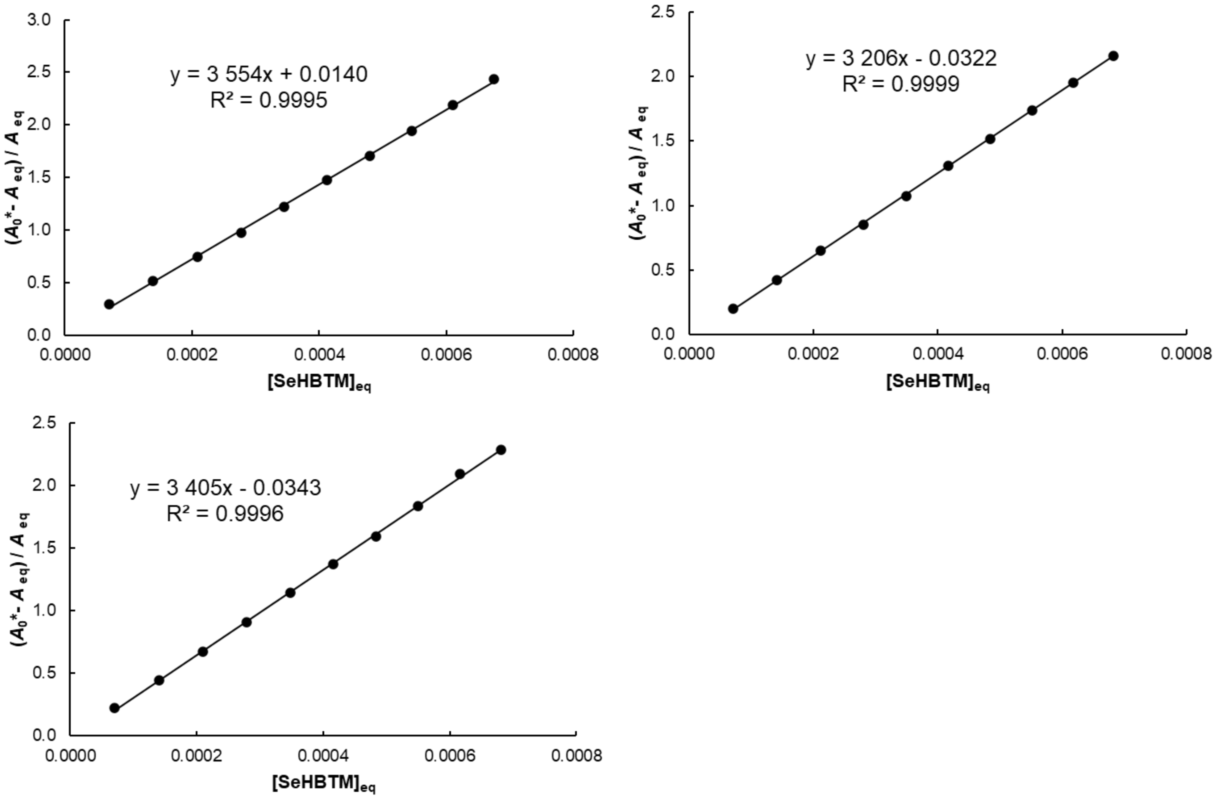

Figure S41: Linear regression plots for the determination of the equilibrium constants between SeHBTM (**ISeU4**) and $(pyr)_2CH^+$ (observed at 620 nm). ε(pyr, 620 nm = 1.74×10^5 L mol^{-1} cm^{-1}). LSM-42

Table S50: Calculation of the equilibrium constant *K* for the reaction between SeHBTM (**ISeU4**) and $(pyr)_2CH^+$

	K (L mol^{-1})
Titration #1	3.55×10^3
Titration #2	3.21×10^3
Titration #3	3.41×10^3
**K_{av} =**	**$(3.39 \pm 0.14) \times 10^3$ L mol^{-1}**

Table S51: Determination of the equilibrium constant between SeHBTM (**ISeU4**) and $(pyr)_2CH^+$ (observed at 620 nm). ε(pyr, 620 nm = 1.74×10^5 L mol^{-1} cm^{-1}). LSM-42

Titration	Step	$V_{tot,Nu}$ (mL)	V_{tot} (mL)	A_{eq}	$[pyr]_0$ (mol L^{-1})	A_0*	A_0*– A_{eq}	$[SeHBTM]_0$ (mol L^{-1})	$[SeHBTM]_{eq}$ (mol L^{-1})	(A_0* – A_{eq})/A_{eq}
#1	0	0	17.3	1.02	9.79×10^{-6}	1.02				
	1	0.1	17.4	0.785	9.74×10^{-6}	1.01	0.228	7.19×10^{-5}	6.92×10^{-5}	0.290
	2	0.2	17.5	0.666	9.68×10^{-6}	1.01	0.341	1.43×10^{-4}	1.39×10^{-4}	0.513
	3	0.3	17.6	0.574	9.63×10^{-6}	1.00	0.427	2.13×10^{-4}	2.08×10^{-4}	0.745
	4	0.4	17.7	0.504	9.57×10^{-6}	0.996	0.492	2.83×10^{-4}	2.77×10^{-4}	0.976
	5	0.5	17.8	0.445	9.52×10^{-6}	0.990	0.545	3.51×10^{-4}	3.45×10^{-4}	1.22
	6	0.6	17.9	0.397	9.46×10^{-6}	0.985	0.587	4.19×10^{-4}	4.12×10^{-4}	1.48
	7	0.7	18.0	0.362	9.41×10^{-6}	0.979	0.618	4.86×10^{-4}	4.79×10^{-4}	1.71
	8	0.8	18.1	0.331	9.36×10^{-6}	0.974	0.643	5.53×10^{-4}	5.45×10^{-4}	1.94
	9	0.9	18.2	0.303	9.31×10^{-6}	0.969	0.665	6.18×10^{-4}	6.11×10^{-4}	2.19
	10	1	18.3	0.280	9.26×10^{-6}	0.963	0.683	6.83×10^{-4}	6.75×10^{-4}	2.44
#2	0	0	17.1	0.998	9.90×10^{-6}	0.998				
	1	0.1	17.2	0.827	9.84×10^{-6}	0.992	0.165	7.26×10^{-5}	7.07×10^{-5}	0.200
	2	0.2	17.3	0.692	9.79×10^{-6}	0.986	0.294	1.44×10^{-4}	1.41×10^{-4}	0.424
	3	0.3	17.4	0.594	9.73×10^{-6}	0.981	0.386	2.15×10^{-4}	2.11×10^{-4}	0.650
	4	0.4	17.5	0.526	9.67×10^{-6}	0.975	0.449	2.86×10^{-4}	2.80×10^{-4}	0.853
	5	0.5	17.6	0.467	9.62×10^{-6}	0.969	0.502	3.55×10^{-4}	3.49×10^{-4}	1.08
	6	0.6	17.7	0.418	9.57×10^{-6}	0.964	0.546	4.24×10^{-4}	4.17×10^{-4}	1.31
	7	0.7	17.8	0.381	9.51×10^{-6}	0.959	0.578	4.91×10^{-4}	4.85×10^{-4}	1.52
	8	0.8	17.9	0.348	9.46×10^{-6}	0.953	0.605	5.58×10^{-4}	5.52×10^{-4}	1.74
	9	0.9	18.0	0.321	9.41×10^{-6}	0.948	0.627	6.25×10^{-4}	6.18×10^{-4}	1.95
	10	1	18.1	0.299	9.35×10^{-6}	0.943	0.644	6.90×10^{-4}	6.83×10^{-4}	2.16
#3	0	0	17.2	0.974	9.87×10^{-6}	0.974				
	1	0.1	17.3	0.789	9.81×10^{-6}	0.968	0.180	7.24×10^{-5}	7.03×10^{-5}	0.228
	2	0.2	17.4	0.665	9.77×10^{-6}	0.963	0.297	1.44×10^{-4}	1.41×10^{-4}	0.447
	3	0.3	17.5	0.572	9.70×10^{-6}	0.957	0.385	2.15×10^{-4}	2.10×10^{-4}	0.673
	4	0.4	17.6	0.499	9.64×10^{-6}	0.952	0.453	2.85×10^{-4}	2.79×10^{-4}	0.908
	5	0.5	17.7	0.442	9.59×10^{-6}	0.946	0.504	3.54×10^{-4}	3.48×10^{-4}	1.14
	6	0.6	17.8	0.396	9.53×10^{-6}	0.941	0.545	4.22×10^{-4}	4.16×10^{-4}	1.37
	7	0.7	17.9	0.360	9.48×10^{-6}	0.936	0.575	4.90×10^{-4}	4.83×10^{-4}	1.60
	8	0.8	18.0	0.329	9.43×10^{-6}	0.931	0.602	5.57×10^{-4}	5.50×10^{-4}	1.83
	9	0.9	18.1	0.300	9.37×10^{-6}	0.925	0.626	6.23×10^{-4}	6.16×10^{-4}	2.09
	10	1	18.2	0.280	9.32×10^{-6}	0.920	0.640	6.88×10^{-4}	6.81×10^{-4}	2.29

SeHBTM + $(thq)_2CH^+$

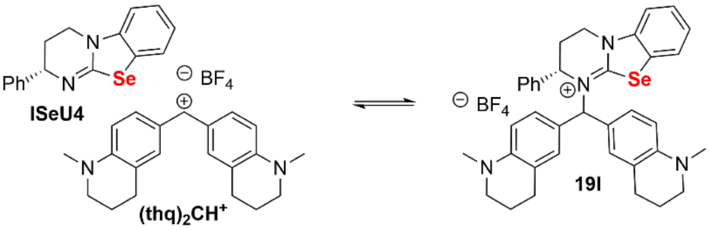

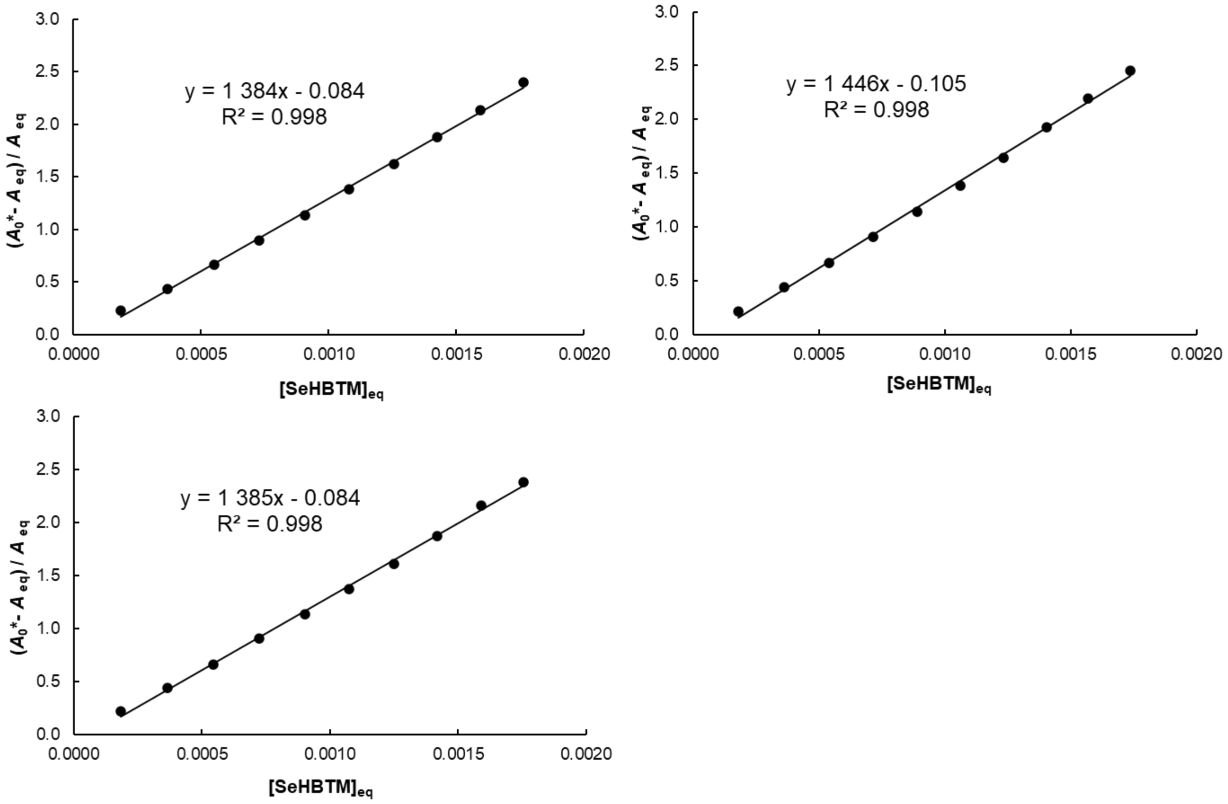

Figure S42: Linear regression plots for the determination of the equilibrium constants between SeHBTM (**ISeU4**) and $(thq)_2CH^+$ (observed at 628 nm). ε(thq, 628 nm = 1.78 × 10^5 L mol^{-1} cm^{-1}). LSM-44

Table S52: Calculation of the equilibrium constant *K* for the reaction between SeHBTM (**ISeU4**) and $(thq)_2CH^+$

	K (L mol^{-1})
Titration #1	1.38 × 10^3
Titration #2	1.45 × 10^3
Titration #3	1.39 × 10^3
**K_{av} =**	**(1.40 ± 0.03) × 10^3 L mol^{-1}**

Table S53: Determination of the equilibrium constant between SeHBTM (**lSeU4**) and $(thq)_2CH^+$ (observed at 628 nm). ε(thq, 628 nm = 1.78×10^5 L mol^{-1} cm^{-1}). LSM-44

Titration	Step	$V_{tot,Nu}$ (mL)	V_{tot} (mL)	A_{eq}	$[thq]_0$ (mol L^{-1})	A_0*	A_0*– A_{eq}	$[SeHBTM]_0$ (mol L^{-1})	$[SeHBTM]_{eq}$ (mol L^{-1})	(A_0* – A_{eq})/A_{eq}
#1	0	0	17.0	0.964	9.88×10^{-6}	0.964				
	1	0.1	17.1	0.780	9.82×10^{-6}	0.958	0.178	1.89×10^{-4}	1.85×10^{-4}	0.228
	2	0.2	17.2	0.665	9.76×10^{-6}	0.953	0.288	3.69×10^{-4}	3.69×10^{-4}	0.433
	3	0.3	17.3	0.570	9.71×10^{-6}	0.947	0.377	5.50×10^{-4}	5.50×10^{-4}	0.662
	4	0.4	17.4	0.496	9.65×10^{-6}	0.942	0.446	7.29×10^{-4}	7.29×10^{-4}	0.900
	5	0.5	17.5	0.438	9.59×10^{-6}	0.936	0.498	9.06×10^{-4}	9.06×10^{-4}	1.14
	6	0.6	17.6	0.391	9.54×10^{-6}	0.931	0.540	1.08×10^{-3}	1.08×10^{-3}	1.38
	7	0.7	17.7	0.353	9.49×10^{-6}	0.926	0.573	1.25×10^{-3}	1.25×10^{-3}	1.62
	8	0.8	17.8	0.319	9.43×10^{-6}	0.921	0.601	1.43×10^{-3}	1.43×10^{-3}	1.88
	9	0.9	17.9	0.292	9.38×10^{-6}	0.916	0.624	1.60×10^{-3}	1.59×10^{-3}	2.14
	10	1	18.0	0.268	9.33×10^{-6}	0.911	0.643	1.76×10^{-3}	1.76×10^{-3}	2.40
#2	0	0	17.2	0.970	9.76×10^{-6}	0.970				
	1	0.1	17.3	0.796	9.70×10^{-6}	0.964	0.168	1.83×10^{-4}	1.81×10^{-4}	0.211
	2	0.2	17.4	0.667	9.64×10^{-6}	0.959	0.292	3.64×10^{-4}	3.61×10^{-4}	0.438
	3	0.3	17.5	0.572	9.59×10^{-6}	0.953	0.382	5.44×10^{-4}	5.39×10^{-4}	0.668
	4	0.4	17.6	0.498	9.53×10^{-6}	0.948	0.450	7.21×10^{-4}	7.16×10^{-4}	0.905
	5	0.5	17.7	0.440	9.48×10^{-6}	0.943	0.502	8.96×10^{-4}	8.90×10^{-4}	1.14
	6	0.6	17.8	0.393	9.43×10^{-6}	0.937	0.545	1.07×10^{-3}	1.06×10^{-3}	1.39
	7	0.7	17.9	0.353	9.38×10^{-6}	0.932	0.579	1.24×10^{-3}	1.23×10^{-3}	1.64
	8	0.8	18.0	0.317	9.32×10^{-6}	0.927	0.610	1.41×10^{-3}	1.40×10^{-3}	1.92
	9	0.9	18.1	0.289	9.29×10^{-6}	0.922	0.633	1.58×10^{-3}	1.57×10^{-3}	2.19
	10	1	18.2	0.265	9.27×10^{-6}	0.917	0.652	1.74×10^{-3}	1.74×10^{-3}	2.46
#3	0	0	17.0	0.996	9.88×10^{-6}	0.996				
	1	0.1	17.1	0.813	9.82×10^{-6}	0.990	0.176	1.86×10^{-4}	1.84×10^{-4}	0.217
	2	0.2	17.2	0.683	9.77×10^{-6}	0.984	0.301	3.69×10^{-4}	3.66×10^{-4}	0.440
	3	0.3	17.3	0.590	9.71×10^{-6}	0.978	0.388	5.50×10^{-4}	5.46×10^{-4}	0.658
	4	0.4	17.4	0.511	9.65×10^{-6}	0.973	0.462	7.30×10^{-4}	7.24×10^{-4}	0.905
	5	0.5	17.5	0.454	9.60×10^{-6}	0.967	0.513	9.07×10^{-4}	9.01×10^{-4}	1.13
	6	0.6	17.6	0.405	9.54×10^{-6}	0.962	0.556	1.08×10^{-3}	1.08×10^{-3}	1.37
	7	0.7	17.7	0.366	9.49×10^{-6}	0.956	0.590	1.26×10^{-3}	1.25×10^{-3}	1.61
	8	0.8	17.8	0.331	9.44×10^{-6}	0.951	0.620	1.43×10^{-3}	1.42×10^{-3}	1.87
	9	0.9	17.9	0.299	9.38×10^{-6}	0.946	0.647	1.60×10^{-3}	1.59×10^{-3}	2.16
	10	1	18.0	0.278	9.33×10^{-6}	0.940	0.662	1.76×10^{-3}	1.76×10^{-3}	2.38

SeHyperBTM + $(pyr)_2CH^+$

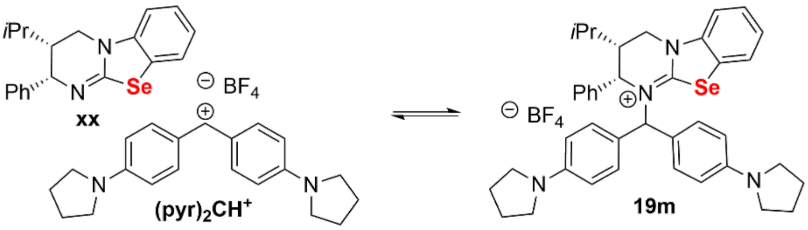

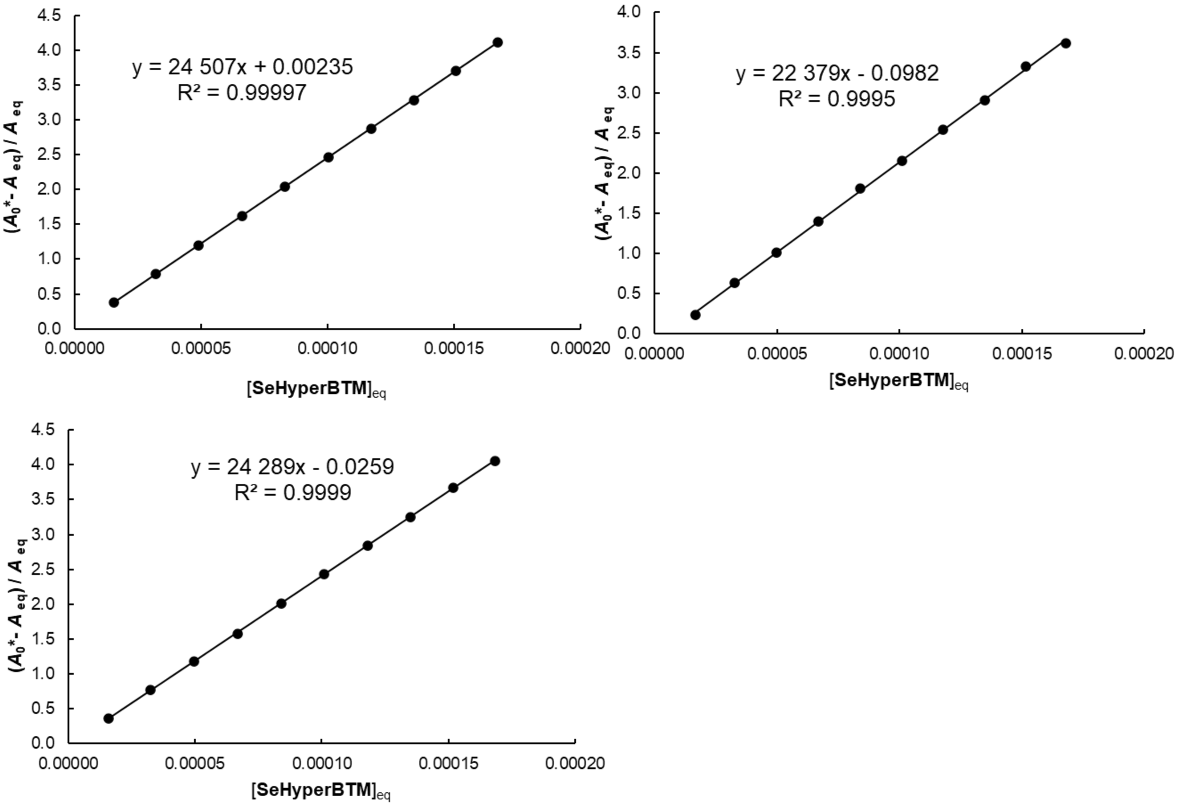

Figure S43: Linear regression plots for the determination of the equilibrium constants between Se*Hyper*BTM (**ISeU5**) and $(pyr)_2CH^+$ (observed at 620 nm). ε(pyr, 620 nm = 1.74 × 10^5 L mol^{-1} cm^{-1}). LSM-37

Table S54: Calculation of the equilibrium constant *K* for the reaction between Se*Hyper*BTM (**ISeU5**) and $(pyr)_2CH^+$

	K (L mol^{-1})
Titration #1	2.45 × 10^4
Titration #2	2.24 × 10^4
Titration #3	2.43 × 10^4
***K*$_{av}$ =**	**(2.37 ± 0.10) × 10^4 L mol^{-1}**

Table S55: Determination of the equilibrium constant between Se*Hyper*BTM (**ISeU5**) and $(pyr)_2CH^+$ (observed at 620 nm). ε(pyr, 620 nm = 1.74×10^5 L mol^{-1} cm^{-1}). LSM-37

Titration	Step	$V_{tot,Nu}$ (mL)	V_{tot} (mL)	A_{eq}	$[pyr]_0$ (mol L^{-1})	A_0*	A_0*– A_{eq}	[SeHyper]$_0$ (mol L^{-1})	[SeHyper]$_{eq}$ (mol L^{-1})	(A_0* – A_{eq})/A_{eq}
#1	0	0	17.1	0.980	3.26×10^{-4}	0.980				
	1	0.1	17.2	0.705	3.25×10^{-5}	0.974	0.269	1.85×10^{-5}	1.54×10^{-5}	0.382
	2	0.2	17.3	0.542	3.23×10^{-5}	0.969	0.427	3.68×10^{-5}	3.19×10^{-5}	0.787
	3	0.3	17.4	0.438	3.21×10^{-5}	0.963	0.525	5.49×10^{-5}	4.89×10^{-5}	1.20
	4	0.4	17.5	0.366	3.19×10^{-5}	0.958	0.592	7.28×10^{-5}	6.60×10^{-5}	1.62
	5	0.5	17.6	0.313	3.17×10^{-5}	0.952	0.639	9.05×10^{-5}	8.32×10^{-5}	2.04
	6	0.6	17.7	0.273	3.15×10^{-5}	0.947	0.674	1.08×10^{-4}	1.00×10^{-5}	2.47
	7	0.7	17.8	0.243	3.14×10^{-5}	0.941	0.698	1.25×10^{-4}	1.17×10^{-5}	2.87
	8	0.8	17.9	0.219	3.12×10^{-5}	0.936	0.717	1.42×10^{-4}	1.34×10^{-4}	3.27
	9	0.9	18.0	0.198	3.10×10^{-5}	0.931	0.733	1.59×10^{-4}	1.51×10^{-4}	3.70
	10	1.0	18.1	0.181	3.08×10^{-5}	0.926	0.745	1.76×10^{-4}	1.67×10^{-4}	4.11
#2	0	0	17.1	0.907	3.26×10^{-5}	0.907				
	1	0.1	17.2	0.726	3.24×10^{-5}	0.902	0.176	1.85×10^{-5}	1.65×10^{-5}	0.24
	2	0.2	17.3	0.548	3.22×10^{-5}	0.897	0.349	3.68×10^{-5}	3.28×10^{-5}	0.64
	3	0.3	17.4	0.442	3.20×10^{-5}	0.892	0.449	5.49×10^{-5}	4.97×10^{-5}	1.02
	4	0.4	17.5	0.369	3.19×10^{-5}	0.887	0.517	7.27×10^{-5}	6.68×10^{-5}	1.40
	5	0.5	17.6	0.313	3.17×10^{-5}	0.881	0.568	9.04×10^{-5}	8.39×10^{-5}	1.81
	6	0.6	17.7	0.277	3.15×10^{-5}	0.877	0.599	1.08×10^{-4}	1.01×10^{-4}	2.16
	7	0.7	17.8	0.246	3.13×10^{-5}	0.872	0.626	1.25×10^{-4}	1.18×10^{-4}	2.54
	8	0.8	17.9	0.222	3.11×10^{-5}	0.867	0.645	1.42×10^{-4}	1.35×10^{-4}	2.91
	9	0.9	18.0	0.199	3.10×10^{-5}	0.862	0.663	1.59×10^{-4}	1.51×10^{-4}	3.33
	10	1.0	18.1	0.186	3.08×10^{-5}	0.857	0.672	1.76×10^{-4}	1.68×10^{-4}	3.62
#3	0	0	17.0	0.911	3.27×10^{-5}	0.911				
	1	0.1	17.1	0.668	3.25×10^{-5}	0.906	0.238	1.86×10^{-5}	1.58×10^{-5}	0.357
	2	0.2	17.2	0.509	3.23×10^{-5}	0.901	0.391	3.69×10^{-5}	3.24×10^{-5}	0.768
	3	0.3	17.3	0.411	3.21×10^{-5}	0.896	0.484	5.50×10^{-5}	4.95×10^{-5}	1.18
	4	0.4	17.4	0.345	3.20×10^{-5}	0.890	0.545	7.30×10^{-5}	6.67×10^{-5}	1.58
	5	0.5	17.5	0.294	3.18×10^{-5}	0.885	0.591	9.07×10^{-5}	8.39×10^{-5}	2.01
	6	0.6	17.6	0.256	3.16×10^{-5}	0.880	0.624	1.08×10^{-5}	1.01×10^{-4}	2.43
	7	0.7	17.7	0.228	3.14×10^{-5}	0.875	0.648	1.26×10^{-4}	1.18×10^{-4}	2.84
	8	0.8	17.8	0.205	3.12×10^{-5}	0.871	0.666	1.43×10^{-4}	1.35×10^{-4}	3.25
	9	0.9	17.9	0.185	3.11×10^{-5}	0.866	0.681	1.60×10^{-4}	1.52×10^{-4}	3.68
	10	1.0	18.0	0.170	3.09×10^{-5}	0.861	0.690	1.76×10^{-4}	1.68×10^{-4}	4.05

SeHyperBTM + (thq)$_2$CH$^+$

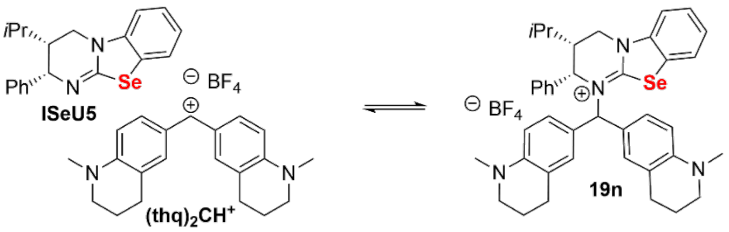

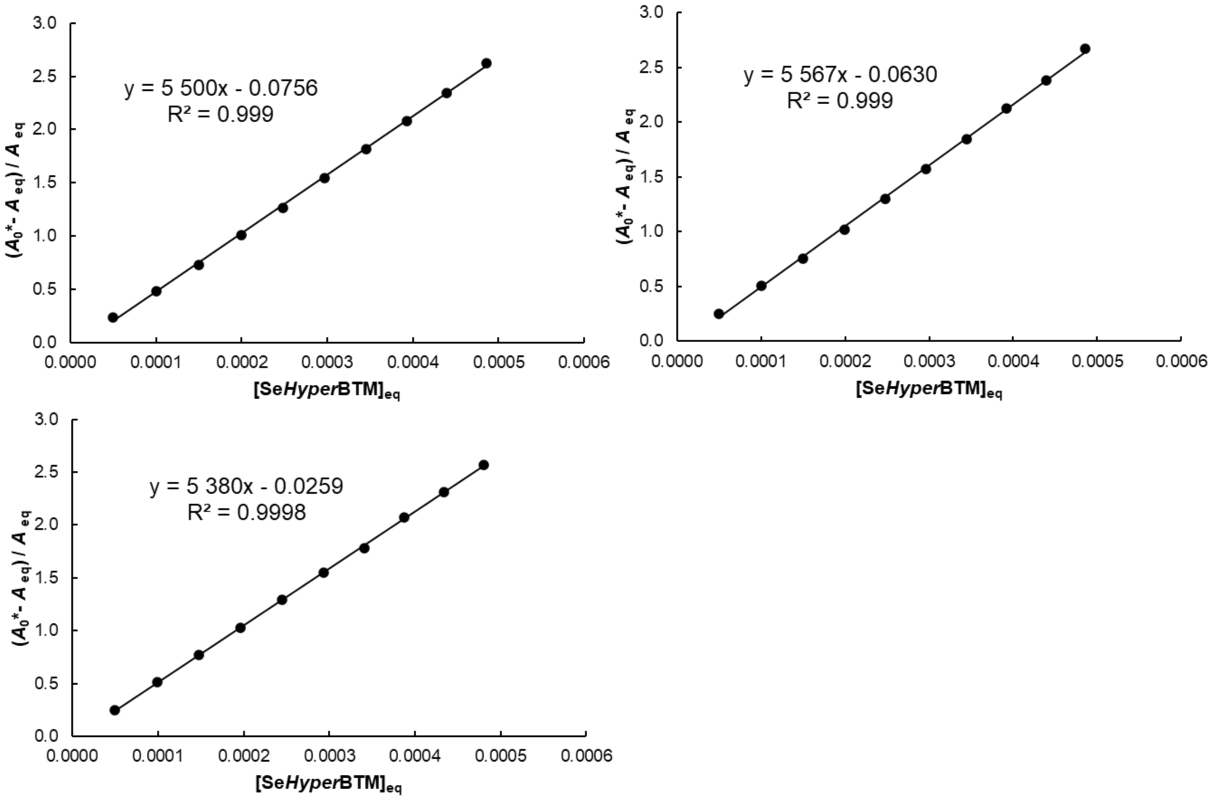

Figure S44: Linear regression plots for the determination of the equilibrium constants between Se*Hyper*BTM (**ISeU5**) and (thq)$_2$CH$^+$ (observed at 628 nm). ε(thq, 628 nm = 1.78 × 10^5 L mol^{-1} cm^{-1}). LSM-38

Table S56: Calculation of the equilibrium constant *K* for the reaction between Se*Hyper*BTM (**ISeU5**) and (thq)$_2$CH$^+$

	K (L mol^{-1})
Titration #1	5.50 × 10^3
Titration #2	5.57 × 10^3
Titration #3	5.38 × 10^3
**K_{av} =**	**(5.48 ± 0.08) × 10^3 L mol^{-1}**

Table S57: Determination of the equilibrium constant between Se*Hyper*BTM (**ISeU5**) and $(thq)_2CH^+$ (observed at 628 nm). ε(thq, 628 nm = 1.78 × 10^5 L mol^{-1} cm^{-1}). LSM-38

Titration	Step	$V_{tot,Nu}$ (mL)	V_{tot} (mL)	A_{eq}	$[thq]_0$ (mol L^{-1})	A_0*	A_0*– A_{eq}	$[SeHyper]_0$ (mol L^{-1})	$[SeHyper]_{eq}$ (mol L^{-1})	(A_0* – A_{eq})/A_{eq}
#1	0	0	17.1	0.784	9.89 × 10^{-6}	0.784				
	1	0.1	17.2	0.631	9.81 × 10^{-6}	0.779	0.149	5.18 × 10^{-5}	5.02 × 10^{-5}	0.236
	2	0.2	17.3	0.522	9.75 × 10^{-6}	0.775	0.253	1.03 × 10^{-4}	1.00 × 10^{-4}	0.483
	3	0.3	17.4	0.446	9.69 × 10^{-6}	0.770	0.324	1.54 × 10^{-4}	1.50 × 10^{-4}	0.728
	4	0.4	17.5	0.381	9.64 × 10^{-6}	0.766	0.385	2.04 × 10^{-4}	1.99 × 10^{-4}	1.01
	5	0.5	17.6	0.336	9.58 × 10^{-6}	0.762	0.426	2.53 × 10^{-4}	2.48 × 10^{-4}	1.27
	6	0.6	17.7	0.298	9.53 × 10^{-6}	0.757	0.460	3.02 × 10^{-4}	2.97 × 10^{-4}	1.54
	7	0.7	17.8	0.267	9.47 × 10^{-6}	0.753	0.486	3.51 × 10^{-4}	3.45 × 10^{-4}	1.82
	8	0.8	17.9	0.243	9.42 × 10^{-6}	0.749	0.506	3.98 × 10^{-4}	3.93 × 10^{-4}	2.08
	9	0.9	18.0	0.222	9.37 × 10^{-6}	0.745	0.522	4.46 × 10^{-4}	4.40 × 10^{-4}	2.35
	10	1	18.1	0.204	9.32 × 10^{-6}	0.741	0.536	4.92 × 10^{-4}	4.86 × 10^{-4}	2.63
#2	0	0	17.1	0.764	9.85 × 10^{-6}	0.764				
	1	0.1	17.2	0.608	9.79 × 10^{-6}	0.760	0.152	5.18 × 10^{-5}	5.01 × 10^{-5}	0.249
	2	0.2	17.3	0.501	9.74 × 10^{-6}	0.755	0.254	1.03 × 10^{-4}	1.00 × 10^{-4}	0.508
	3	0.3	17.4	0.427	9.68 × 10^{-6}	0.751	0.324	1.53 × 10^{-4}	1.50 × 10^{-4}	0.758
	4	0.4	17.5	0.370	9.62 × 10^{-6}	0.747	0.377	2.03 × 10^{-4}	1.99 × 10^{-4}	1.02
	5	0.5	17.6	0.322	9.57 × 10^{-6}	0.742	0.420	2.53 × 10^{-4}	2.48 × 10^{-4}	1.30
	6	0.6	17.7	0.287	9.52 × 10^{-6}	0.738	0.451	3.02 × 10^{-4}	2.97 × 10^{-4}	1.57
	7	0.7	17.8	0.258	9.46 × 10^{-6}	0.734	0.476	3.50 × 10^{-4}	3.45 × 10^{-4}	1.85
	8	0.8	17.9	0.234	9.41 × 10^{-6}	0.730	0.496	3.98 × 10^{-4}	3.92 × 10^{-4}	2.12
	9	0.9	18.0	0.215	9.36 × 10^{-6}	0.726	0.511	4.45 × 10^{-4}	4.39 × 10^{-4}	2.38
	10	1	18.1	0.197	9.31 × 10^{-6}	0.722	0.525	4.92 × 10^{-4}	4.86 × 10^{-4}	2.67
#3	0	0	17.3	0.768	9.73 × 10^{-6}	0.768				
	1	0.1	17.4	0.612	9.67 × 10^{-6}	0.763	0.151	5.11 × 10^{-5}	4.94 × 10^{-5}	0.247
	2	0.2	17.5	0.502	9.62 × 10^{-6}	0.759	0.257	1.02 × 10^{-4}	9.88 × 10^{-5}	0.512
	3	0.3	17.6	0.426	9.56 × 10^{-6}	0.755	0.328	1.52 × 10^{-4}	1.48 × 10^{-4}	0.771
	4	0.4	17.7	0.370	9.51 × 10^{-6}	0.750	0.380	2.01 × 10^{-4}	1.97 × 10^{-4}	1.03
	5	0.5	17.8	0.326	9.46 × 10^{-6}	0.746	0.420	2.50 × 10^{-4}	2.45 × 10^{-4}	1.29
	6	0.6	17.9	0.291	9.40 × 10^{-6}	0.742	0.451	2.98 × 10^{-4}	2.93 × 10^{-4}	1.55
	7	0.7	18.0	0.266	9.35 × 10^{-6}	0.738	0.472	3.46 × 10^{-4}	3.41 × 10^{-4}	1.78
	8	0.8	18.1	0.239	9.30 × 10^{-6}	0.734	0.495	3.93 × 10^{-4}	3.88 × 10^{-4}	2.07
	9	0.9	18.2	0.221	9.25 × 10^{-6}	0.730	0.509	4.40 × 10^{-4}	4.34 × 10^{-4}	2.31
	10	1	18.3	0.203	9.20 × 10^{-6}	0.726	0.523	4.86 × 10^{-4}	4.80 × 10^{-4}	2.57

TeDHPB + (lil)$_2$CH$^+$

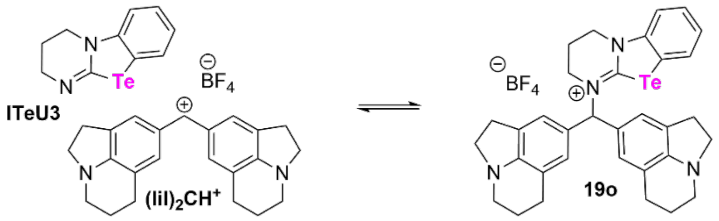

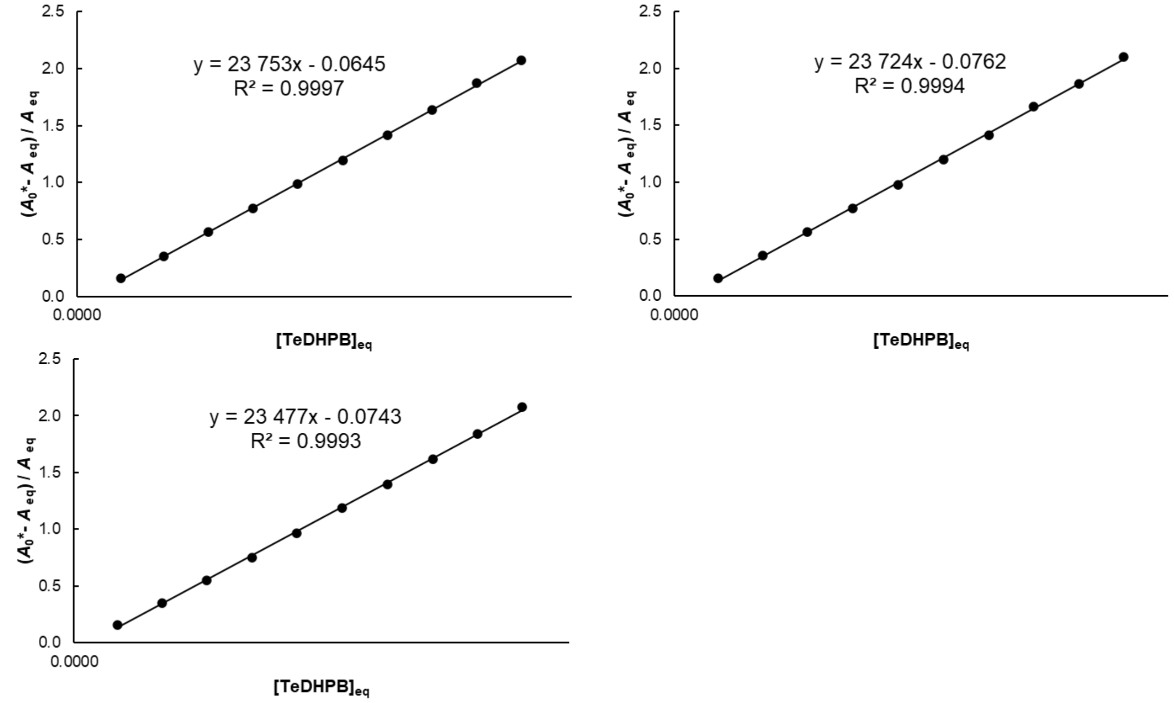

Figure S45: Linear regression plots for the determination of the equilibrium constants between TeDHPB (**ITeU3**) and (lil)$_2$CH$^+$ (observed at 639 nm). ε(lil, 639 nm = 1.59 × 10^5 L mol^{-1} cm^{-1}). LSM-148

Table S58: Calculation of the equilibrium constant K for the reaction between TeDHPB (**ITeU3**) and (ind)$_2$CH$^+$

	K (L mol^{-1})
Titration #1	2.38 × 10^4
Titration #2	2.37 × 10^4
Titration #3	2.35 × 10^4
***K*$_{av}$ =**	**(2.37 ± 0.01) × 10^4 L mol^{-1}**

Table S59: Determination of the equilibrium constant between TeDHPB (**ITeU3**) and $(lil)_2CH^+$ (observed at 639 nm). ε(lil, 639 nm = 1.59 × 10^5 L mol^{-1} cm^{-1}). LSM-148

Titration	Step	$V_{tot,Nu}$ (mL)	V_{tot} (mL)	A_{eq}	$[lil]_0$ (mol L^{-1})	A_0*	A_0*– A_{eq}	$[TeDHPB]_0$ (mol L^{-1})	$[TeDHPB]_{eq}$ (mol L^{-1})	$(A_0* – A_{eq})/A_{eq}$
#1	0	0	17.2	0.791	7.04 × 10^{-6}	0.791				
	1	0.1	17.3	0.678	7.00 × 10^{-6}	0.786	0.108	1.01 × 10^{-5}	8.75 × 10^{-6}	0.159
	2	0.2	17.4	0.577	6.96 × 10^{-6}	0.782	0.205	2.01 × 10^{-5}	1.75 × 10^{-5}	0.355
	3	0.3	17.5	0.497	6.92 × 10^{-6}	0.777	0.281	3.00 × 10^{-5}	2.64 × 10^{-5}	0.565
	4	0.4	17.6	0.436	6.88 × 10^{-6}	0.773	0.337	3.98 × 10^{-5}	3.55 × 10^{-5}	0.772
	5	0.5	17.7	0.387	6.84 × 10^{-6}	0.769	0.382	4.94 × 10^{-5}	4.46 × 10^{-5}	0.987
	6	0.6	17.8	0.349	6.80 × 10^{-6}	0.764	0.416	5.90 × 10^{-5}	5.37 × 10^{-5}	1.19
	7	0.7	17.9	0.314	6.76 × 10^{-6}	0.760	0.446	6.84 × 10^{-5}	6.28 × 10^{-5}	1.42
	8	0.8	18.0	0.287	6.73 × 10^{-6}	0.756	0.469	7.77 × 10^{-5}	7.18 × 10^{-5}	1.64
	9	0.9	18.1	0.262	6.69 × 10^{-6}	0.752	0.490	8.70 × 10^{-5}	8.08 × 10^{-5}	1.87
	10	1	18.2	0.243	6.65 × 10^{-6}	0.747	0.504	9.61 × 10^{-5}	8.97 × 10^{-5}	2.08
#2	0	0	17.0	0.783	7.12 × 10^{-6}	0.783				
	1	0.1	17.1	0.674	7.08 × 10^{-6}	0.778	0.104	1.02 × 10^{-5}	8.92 × 10^{-6}	0.155
	2	0.2	17.2	0.571	7.04 × 10^{-6}	0.774	0.203	2.03 × 10^{-5}	1.78 × 10^{-5}	0.355
	3	0.3	17.3	0.492	7.00 × 10^{-6}	0.769	0.277	3.03 × 10^{-5}	2.68 × 10^{-5}	0.563
	4	0.4	17.4	0.433	6.96 × 10^{-6}	0.765	0.332	4.02 × 10^{-5}	3.60 × 10^{-5}	0.768
	5	0.5	17.5	0.385	6.92 × 10^{-6}	0.760	0.375	5.00 × 10^{-5}	4.52 × 10^{-5}	0.975
	6	0.6	17.6	0.344	6.88 × 10^{-6}	0.756	0.412	5.96 × 10^{-5}	5.44 × 10^{-5}	1.20
	7	0.7	17.7	0.311	6.84 × 10^{-6}	0.752	0.441	6.92 × 10^{-5}	6.36 × 10^{-5}	1.42
	8	0.8	17.8	0.281	6.80 × 10^{-6}	0.748	0.467	7.86 × 10^{-5}	7.27 × 10^{-5}	1.66
	9	0.9	17.9	0.260	6.76 × 10^{-6}	0.743	0.484	8.80 × 10^{-5}	8.19 × 10^{-5}	1.86
	10	1	18.0	0.238	6.73 × 10^{-6}	0.739	0.501	9.72 × 10^{-5}	9.09 × 10^{-5}	2.10
#3	0	0	17.1	0.791	7.09 × 10^{-6}	0.791				
	1	0.1	17.2	0.679	7.05 × 10^{-6}	0.786	0.107	1.02 × 10^{-5}	8.84 × 10^{-6}	0.158
	2	0.2	17.3	0.580	7.01 × 10^{-6}	0.782	0.202	2.03 × 10^{-5}	1.77 × 10^{-5}	0.349
	3	0.3	17.4	0.500	6.97 × 10^{-6}	0.777	0.277	3.02 × 10^{-5}	2.67 × 10^{-5}	0.553
	4	0.4	17.5	0.441	6.93 × 10^{-6}	0.773	0.332	4.01 × 10^{-5}	3.59 × 10^{-5}	0.753
	5	0.5	17.6	0.391	6.89 × 10^{-6}	0.768	0.377	4.98 × 10^{-5}	4.50 × 10^{-5}	0.964
	6	0.6	17.7	0.349	6.85 × 10^{-6}	0.764	0.415	5.94 × 10^{-5}	5.42 × 10^{-5}	1.19
	7	0.7	17.8	0.317	6.81 × 10^{-6}	0.760	0.443	6.89 × 10^{-5}	6.33 × 10^{-5}	1.40
	8	0.8	17.9	0.288	6.78 × 10^{-6}	0.755	0.467	7.83 × 10^{-5}	7.24 × 10^{-5}	1.62
	9	0.9	18.0	0.264	6.74 × 10^{-6}	0.751	0.487	8.76 × 10^{-5}	8.15 × 10^{-5}	1.84
	10	1	18.1	0.243	6.70 × 10^{-6}	0.747	0.505	9.68 × 10^{-5}	9.05 × 10^{-5}	2.08

TeBTM + $(lil)_2CH^+$

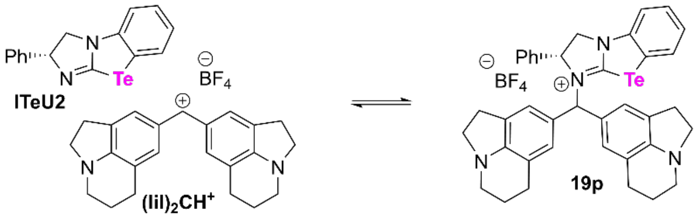

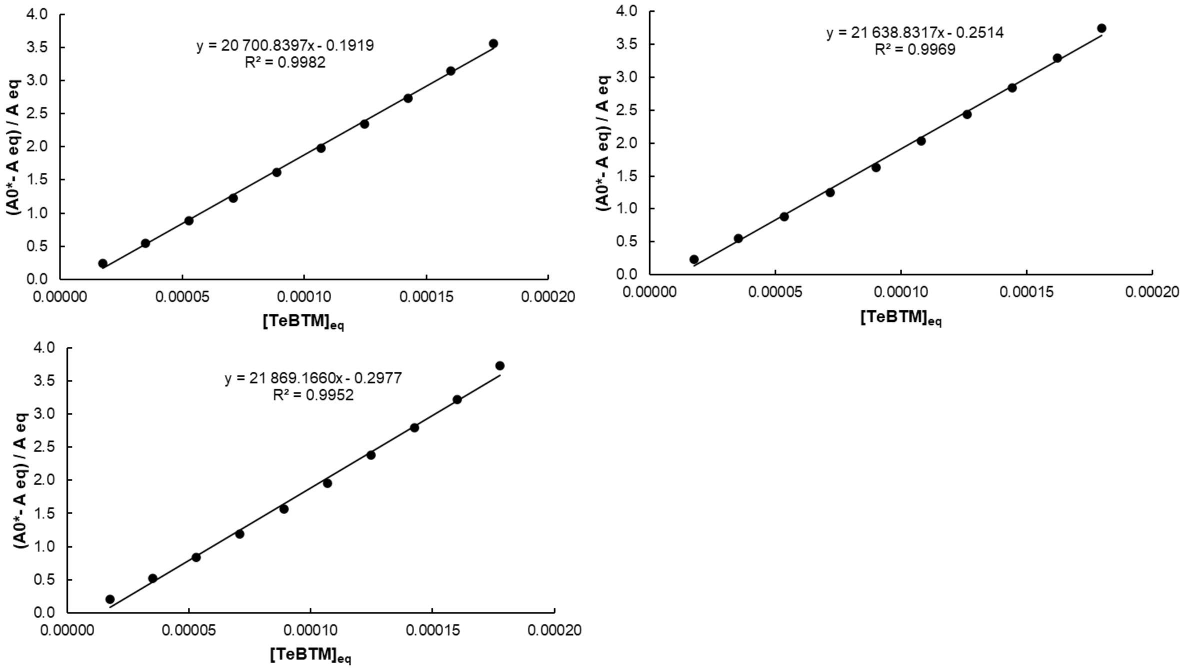

Figure S46: Linear regression plots for the determination of the equilibrium constants between TeBTM (**ITeU2**) and $(lil)_2CH^+$ (observed at 639 nm). ε(lil, 639 nm = 1.59×10^5 L mol^{-1} cm^{-1}). LSM-183

Table S60: Calculation of the equilibrium constant K for the reaction between TeDHPB (**ITeU3**) and $(ind)_2CH^+$

	K (L mol^{-1})
Titration #1	2.07×10^4
Titration #2	2.16×10^4
Titration #3	2.19×10^4
K_{av} =	**$(2.14 \pm 0.05) \times 10^4$ L mol^{-1}**

Table S61: Determination of the equilibrium constant between TeBTM (**ITeU2**) and $(lil)_2CH^+$ (observed at 639 nm). ε(lil, 639 nm = 1.59×10^5 L mol^{-1} cm^{-1}). LSM-183

Titration	Step	$V_{tot,Nu}$ (mL)	V_{tot} (mL)	A_{eq}	$[lil]_0$ (mol L^{-1})	A_0*	A_0*– A_{eq}	$[TeBTM]_0$ (mol L^{-1})	$[TeBTM]_{eq}$ (mol L^{-1})	$(A_0* – A_{eq})/A_{eq}$
#1	0	0	17.2	0.918	9.79×10^{-6}	0.918				
	1	0.1	17.3	0.733	9.74×10^{-6}	0.913	0.180	1.96×10^{-5}	1.73×10^{-5}	0.246
	2	0.2	17.4	0.587	9.68×10^{-6}	0.908	0.321	3.89×10^{-5}	3.49×10^{-5}	0.547
	3	0.3	17.5	0.479	9.63×10^{-6}	0.902	0.423	5.81×10^{-5}	5.28×10^{-5}	0.882
	4	0.4	17.6	0.402	9.57×10^{-6}	0.897	0.495	7.70×10^{-5}	7.08×10^{-5}	1.23
	5	0.5	17.7	0.341	9.52×10^{-6}	0.892	0.551	9.57×10^{-5}	8.88×10^{-5}	1.62
	6	0.6	17.8	0.298	9.46×10^{-6}	0.887	0.589	1.14×10^{-4}	1.07×10^{-4}	1.98
	7	0.7	17.9	0.263	9.41×10^{-6}	0.882	0.619	1.33×10^{-4}	1.25×10^{-4}	2.35
	8	0.8	18.0	0.235	9.36×10^{-6}	0.877	0.643	1.51×10^{-4}	1.42×10^{-4}	2.74
	9	0.9	18.1	0.210	9.31×10^{-6}	0.872	0.662	1.69×10^{-4}	1.60×10^{-4}	3.15
	10	1	18.2	0.190	9.26×10^{-6}	0.868	0.677	1.86×10^{-4}	1.78×10^{-4}	3.56
#2	0	0	17.0	0.923	9.92×10^{-6}	0.923				
	1	0.1	17.1	0.745	9.86×10^{-6}	0.918	0.173	1.98×10^{-5}	1.77×10^{-5}	0.232
	2	0.2	17.2	0.587	9.80×10^{-6}	0.912	0.325	3.94×10^{-5}	3.53×10^{-5}	0.553
	3	0.3	17.3	0.482	9.74×10^{-6}	0.907	0.425	5.88×10^{-5}	5.34×10^{-5}	0.880
	4	0.4	17.4	0.402	9.69×10^{-6}	0.902	0.500	7.79×10^{-5}	7.16×10^{-5}	1.24
	5	0.5	17.5	0.342	9.63×10^{-6}	0.897	0.555	9.69×10^{-5}	8.99×10^{-5}	1.62
	6	0.6	17.6	0.294	9.58×10^{-6}	0.892	0.597	1.16×10^{-4}	1.08×10^{-4}	2.03
	7	0.7	17.7	0.258	9.52×10^{-6}	0.887	0.629	1.34×10^{-4}	1.26×10^{-4}	2.44
	8	0.8	17.8	0.230	9.47×10^{-6}	0.882	0.652	1.52×10^{-4}	1.44×10^{-4}	2.84
	9	0.9	17.9	0.204	9.42×10^{-6}	0.877	0.672	1.70×10^{-4}	1.62×10^{-4}	3.29
	10	1	18.0	0.184	9.37×10^{-6}	0.872	0.688	1.88×10^{-4}	1.80×10^{-4}	3.74
#3	0	0	17.2	0.926	9.80×10^{-6}	0.926	0			
	1	0.1	17.3	0.762	9.75×10^{-6}	0.921	0.159	1.96×10^{-5}	1.76×10^{-5}	0.209
	2	0.2	17.4	0.603	9.69×10^{-6}	0.916	0.312	3.90×10^{-5}	3.50×10^{-5}	0.518
	3	0.3	17.5	0.496	9.64×10^{-6}	0.911	0.414	5.81×10^{-5}	5.29×10^{-5}	0.836
	4	0.4	17.6	0.414	9.58×10^{-6}	0.905	0.491	7.71×10^{-5}	7.09×10^{-5}	1.19
	5	0.5	17.7	0.351	9.53×10^{-6}	0.900	0.549	9.58×10^{-5}	8.89×10^{-5}	1.56
	6	0.6	17.8	0.303	9.47×10^{-6}	0.895	0.592	1.14×10^{-4}	1.07×10^{-4}	1.95
	7	0.7	17.9	0.263	9.42×10^{-6}	0.890	0.627	1.33×10^{-4}	1.25×10^{-4}	2.38
	8	0.8	18.0	0.234	9.37×10^{-6}	0.885	0.652	1.51×10^{-4}	1.44×10^{-4}	2.79
	9	0.9	18.1	0.208	9.32×10^{-6}	0.880	0.672	1.69×10^{-4}	1.60×10^{-4}	3.23
	10	1	18.2	0.185	9.26×10^{-6}	0.876	0.690	1.86×10^{-4}	1.78×10^{-4}	3.73

7-AnTM + $(jul)_2CH^+$

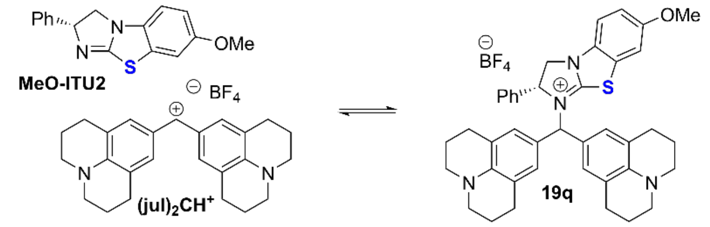

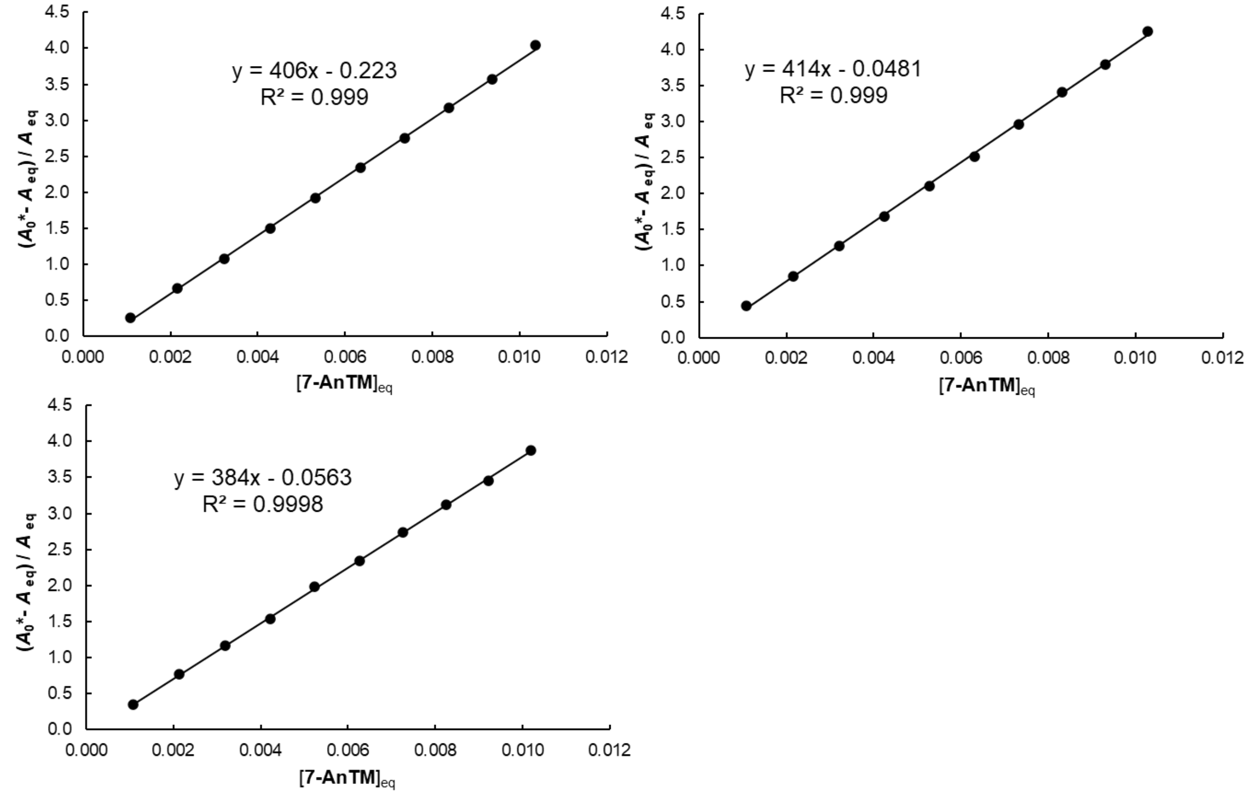

Figure S47: Linear regression plots for the determination of the equilibrium constants between 7-AnTM (**MeO-ITU2**) and $(jul)_2CH^+$ (observed at 642 nm). ε(jul, 642 nm = 2.24 · 10^5 M^{-1} cm^{-1}). LSM-141

Table S62: Calculation of the equilibrium constant K for the reaction between 7-AnTM (**MeO-ITU2**) and $(jul)_2CH^+$

	K (L mol^{-1})
Titration #1	4.06×10^2
Titration #2	4.14×10^2
Titration #3	3.84×10^2
$\boldsymbol{K_{av}}$ **=**	**$(4.01 \pm 0.13) \times 10^4$ L mol^{-1}**

Table S63: Determination of the equilibrium constant between 7-AnTM (**MeO-ITU2**) and $(jul)_2CH^+$ (observed at 642 nm). ε(jul, 642 nm = 2.24 · 10^5 M^{-1} cm^{-1}). LSM-141

Titration	Step	$V_{tot,Nu}$ (mL)	V_{tot} (mL)	A_{eq}	$[jul]_0$ (mol L^{-1})	A_0*	A_0*– A_{eq}	[7-AnTM $]_0$ (mol L^{-1})	[7-AnTM $]_{eq}$ (mol L^{-1})	(A_0* – A_{eq})/A_{eq}
#1	0	0	16.7	1.03	9.97 × 10^{-6}	1.03				
	1	0.1	16.8	0.807	9.91 × 10^{-6}	1.02	0.214	1.09 × 10^{-3}	1.09 × 10^{-3}	0.265
	2	0.2	16.9	0.610	9.85 × 10^{-6}	1.01	0.405	2.17 × 10^{-3}	2.17 × 10^{-3}	0.664
	3	0.3	17.0	0.486	9.79 × 10^{-6}	1.01	0.522	3.24 × 10^{-3}	3.23 × 10^{-3}	1.07
	4	0.4	17.1	0.402	9.73 × 10^{-6}	1.00	0.601	4.29 × 10^{-3}	4.28 × 10^{-3}	1.50
	5	0.5	17.2	0.341	9.68 × 10^{-6}	1.00	0.655	5.33 × 10^{-3}	5.32 × 10^{-3}	1.92
	6	0.6	17.3	0.297	9.62 × 10^{-6}	0.991	0.694	6.36 × 10^{-3}	6.35 × 10^{-3}	2.34
	7	0.7	17.4	0.263	9.57 × 10^{-6}	0.985	0.723	7.38 × 10^{-3}	7.37 × 10^{-3}	2.75
	8	0.8	17.5	0.235	9.51 × 10^{-6}	0.980	0.745	8.38 × 10^{-3}	8.37 × 10^{-3}	3.18
	9	0.9	17.6	0.213	9.46 × 10^{-6}	0.974	0.761	9.38 × 10^{-3}	9.37 × 10^{-3}	3.57
	10	1	17.7	0.192	9.40 × 10^{-6}	0.969	0.777	1.04 × 10^{-2}	1.04 × 10^{-2}	4.04
#2	0	0	16.8	0.997	9.90 × 10^{-6}	0.997				
	1	0.1	16.9	0.688	9.84 × 10^{-6}	0.991	0.304	1.08 × 10^{-3}	1.08 × 10^{-3}	0.441
	2	0.2	17.0	0.530	9.78 × 10^{-6}	0.986	0.455	2.16 × 10^{-3}	2.15 × 10^{-3}	0.858
	3	0.3	17.1	0.431	9.73 × 10^{-6}	0.980	0.549	3.21 × 10^{-3}	3.21 × 10^{-3}	1.27
	4	0.4	17.2	0.362	9.67 × 10^{-6}	0.974	0.612	4.26 × 10^{-3}	4.25 × 10^{-3}	1.69
	5	0.5	17.3	0.311	9.61 × 10^{-6}	0.968	0.657	5.29 × 10^{-3}	5.29 × 10^{-3}	2.11
	6	0.6	17.4	0.274	9.56 × 10^{-6}	0.963	0.689	6.32 × 10^{-3}	6.31 × 10^{-3}	2.52
	7	0.7	17.5	0.241	9.50 × 10^{-6}	0.957	0.716	7.33 × 10^{-3}	7.32 × 10^{-3}	2.97
	8	0.8	17.6	0.216	9.45 × 10^{-6}	0.952	0.736	8.33 × 10^{-3}	8.32 × 10^{-3}	3.42
	9	0.9	17.7	0.197	9.40 × 10^{-6}	0.947	0.749	9.32 × 10^{-3}	9.31 × 10^{-3}	3.79
	10	1	17.8	0.179	9.34 × 10^{-6}	0.941	0.762	1.03 × 10^{-2}	1.03 × 10^{-2}	4.26
#3	0	0	17.0	1.00	9.81 × 10^{-6}	1.00				
	1	0.1	17.1	0.739	9.75 × 10^{-6}	0.994	0.255	1.07 × 10^{-3}	1.07 × 10^{-3}	0.346
	2	0.2	17.2	0.558	9.69 × 10^{-6}	0.989	0.430	2.14 × 10^{-3}	2.13 × 10^{-3}	0.771
	3	0.3	17.3	0.453	9.64 × 10^{-6}	0.983	0.530	3.18 × 10^{-3}	3.18 × 10^{-3}	1.17
	4	0.4	17.4	0.385	9.58 × 10^{-6}	0.977	0.592	4.22 × 10^{-3}	4.22 × 10^{-3}	1.54
	5	0.5	17.5	0.325	9.53 × 10^{-6}	0.972	0.646	5.25 × 10^{-3}	5.24 × 10^{-3}	1.99
	6	0.6	17.6	0.289	9.47 × 10^{-6}	0.966	0.677	6.26 × 10^{-3}	6.25 × 10^{-3}	2.34
	7	0.7	17.7	0.257	9.42 × 10^{-6}	0.961	0.704	7.26 × 10^{-3}	7.26 × 10^{-3}	2.74
	8	0.8	17.8	0.232	9.37 × 10^{-6}	0.955	0.723	8.25 × 10^{-3}	8.25 × 10^{-3}	3.12
	9	0.9	17.9	0.213	9.31 × 10^{-6}	0.950	0.737	9.23 × 10^{-3}	9.23 × 10^{-3}	3.46
	10	1	18.0	0.194	9.26 × 10^{-6}	0.945	0.751	1.02 × 10^{-2}	1.02 × 10^{-2}	3.87

7-F-BTM + (pyr)$_2$CH$^+$

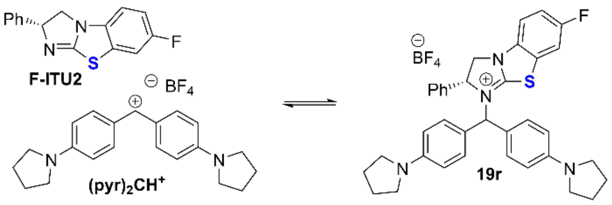

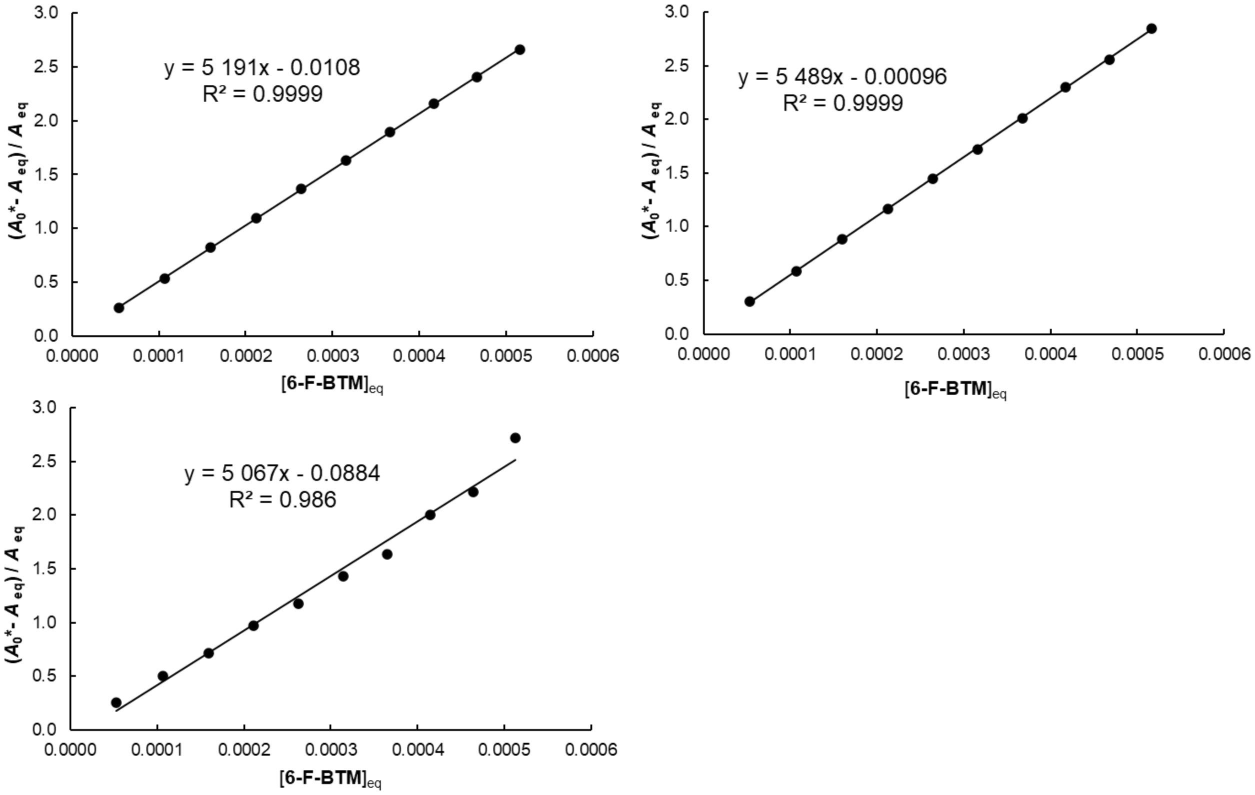

Figure S48: Linear regression plots for the determination of the equilibrium constants between 7-F-BTM (**F-ITU2**) and (pyr)$_2$CH$^+$ (observed at 620 nm). ε(pyr, 620 nm = 1.74 · 10^5 M^{-1} cm^{-1}). LSM-153

Table S64: Calculation of the equilibrium constant K for the reaction between 7-F-BTM (**F-ITU2**) and (pyr)$_2$CH$^+$

	K (L mol^{-1})
Titration #1	5.19 × 10^3
Titration #2	5.49 × 10^3
Titration #3	5.07 × 10^3
**K_{av} =**	**(5.25 ± 0.18) × 10^3 L mol^{-1}**

Table S65: Determination of the equilibrium constant between 7-F-BTM (**F-ITU2**) and $(pyr)_2CH^+$ (observed at 620 nm). ε(pyr, 620 nm = $1.74 \cdot 10^5$ M^{-1} cm^{-1}). LSM-153

Titration	Step	$V_{tot,Nu}$ (mL)	V_{tot} (mL)	A_{eq}	$[pyr]_0$ (mol L^{-1})	A_0*	A_0*– A_{eq}	[7-F-BTM $]_0$ (mol L^{-1})	[7-F-BTM $]_{eq}$ (mol L^{-1})	(A_0* -A_{eq})/A_{eq}
#1	0	0	16.9	0.456	1.02×10^{-5}	0.456				
	1	0.1	17.0	0.359	1.01×10^{-5}	0.454	0.0942	5.47×10^{-5}	5.36×10^{-5}	0.262
	2	0.2	17.1	0.294	1.01×10^{-5}	0.451	0.157	1.09×10^{-4}	1.07×10^{-4}	0.536
	3	0.3	17.2	0.246	9.99×10^{-6}	0.448	0.202	1.62×10^{-4}	1.60×10^{-4}	0.821
	4	0.4	17.3	0.213	9.94×10^{-6}	0.446	0.233	2.15×10^{-4}	2.12×10^{-4}	1.10
	5	0.5	17.4	0.187	9.88×10^{-6}	0.443	0.256	2.67×10^{-4}	2.64×10^{-4}	1.37
	6	0.6	17.5	0.167	9.82×10^{-6}	0.441	0.273	3.19×10^{-4}	3.16×10^{-4}	1.63
	7	0.7	17.6	0.151	9.77×10^{-6}	0.438	0.287	3.70×10^{-4}	3.67×10^{-4}	1.89
	8	0.8	17.7	0.138	9.71×10^{-6}	0.436	0.298	4.20×10^{-4}	4.17×10^{-4}	2.16
	9	0.9	17.8	0.127	9.66×10^{-6}	0.433	0.306	4.70×10^{-4}	4.67×10^{-4}	2.40
	10	1	17.9	0.118	9.60×10^{-6}	0.431	0.313	5.19×10^{-4}	5.16×10^{-4}	2.66
#2	0	0	16.9	0.443	1.02×10^{-5}	0.443				
	1	0.1	17.0	0.338	1.01×10^{-5}	0.440	0.102	5.48×10^{-5}	5.36×10^{-5}	0.302
	2	0.2	17.1	0.276	1.01×10^{-5}	0.438	0.162	1.09×10^{-4}	1.07×10^{-4}	0.585
	3	0.3	17.2	0.231	1.00×10^{-5}	0.435	0.204	1.62×10^{-4}	1.60×10^{-4}	0.883
	4	0.4	17.3	0.200	9.95×10^{-6}	0.433	0.233	2.15×10^{-4}	2.13×10^{-4}	1.17
	5	0.5	17.4	0.176	9.90×10^{-6}	0.430	0.254	2.68×10^{-4}	2.65×10^{-4}	1.44
	6	0.6	17.5	0.157	9.84×10^{-6}	0.428	0.271	3.19×10^{-4}	3.16×10^{-4}	1.72
	7	0.7	17.6	0.141	9.78×10^{-6}	0.425	0.284	3.70×10^{-4}	3.67×10^{-4}	2.01
	8	0.8	17.7	0.128	9.73×10^{-6}	0.423	0.295	4.21×10^{-4}	4.18×10^{-4}	2.30
	9	0.9	17.8	0.118	9.67×10^{-6}	0.421	0.302	4.71×10^{-4}	4.67×10^{-4}	2.56
	10	1	17.9	0.109	9.62×10^{-6}	0.418	0.310	5.20×10^{-4}	5.17×10^{-4}	2.85
#3	0	0	17.1	0.429	1.01×10^{-5}	0.429				
	1	0.1	17.2	0.339	1.00×10^{-5}	0.427	0.0875	5.43×10^{-5}	5.33×10^{-5}	0.258
	2	0.2	17.3	0.282	0.99×10^{-6}	0.424	0.142	1.08×10^{-4}	1.06×10^{-4}	0.503
	3	0.3	17.4	0.245	0.93×10^{-6}	0.422	0.176	1.61×10^{-4}	1.59×10^{-4}	0.719
	4	0.4	17.5	0.212	9.87×10^{-6}	0.419	0.207	2.14×10^{-4}	2.11×10^{-4}	0.974
	5	0.5	17.6	0.192	9.82×10^{-6}	0.417	0.225	2.65×10^{-4}	2.63×10^{-4}	1.17
	6	0.6	17.7	0.171	9.76×10^{-6}	0.414	0.244	3.17×10^{-4}	3.14×10^{-4}	1.43
	7	0.7	17.8	0.156	9.71×10^{-6}	0.412	0.256	3.67×10^{-4}	3.65×10^{-4}	1.63
	8	0.8	17.9	0.136	9.65×10^{-6}	0.410	0.273	4.18×10^{-4}	4.14×10^{-4}	2.00
	9	0.9	18.0	0.127	9.60×10^{-6}	0.408	0.281	4.67×10^{-4}	4.64×10^{-4}	2.22
	10	1	18.1	0.109	9.54×10^{-6}	0.405	0.296	5.16×10^{-4}	5.13×10^{-4}	2.72

Table S66: Equilibrium constants *K* for the association reactions of IChUs with benzhydrylium ions **18** to give adducts **19** and calculation of Lewis basicities *LB* of the IChUs (in CH_2Cl_2 at 20 °C) according to equation (S3). Also see ref. [21]

Catalyst	Lewis Acid (**18**)	*LA*	*K* (L mol^{-1})	Individual *LB*	Averaged *LB*
ODHPB (**IU3**)	$(pyr)_2CH^+$	–10.46	1.36×10^3	13.59	13.66
	$(ind)_2CH^+$	–11.16	3.61×10^2	13.72	
OHyperBTM (**IU5**)	$(dma)_2CH^+$	–9.30	8.23×10^2	12.22	12.42
	$(mpa)_2CH^+$	–7.72	8.01×10^4	12.62	
TM (**ITU1**)	$(jul)_2CH^+$	–12.62	7.63×10^2	15.50	15.50
BTM (**ITU2**)	$(jul)_2CH^+$	–12.62	1.84×10^2	14.88	14.88
SeBTM (**ISeU2**)	$(pyr)_2CH^+$	–10.46	6.38×10^4	15.26	15.23
	$(thq)_2CH^+$	–10.92	1.89×10^4	15.20	
SeDHPB (**ISeU3**)	$(thq)_2CH^+$	–10.92	2.79×10^4	15.36	15.54
	$(lil)_2CH^+$	–12.76	9.14×10^2	15.72	
SeHBTM (**ISeU4**)	$(pyr)_2CH^+$	–10.46	3.39×10^3	13.99	14.03
	$(thq)_2CH^+$	–10.92	1.40×10^3	14.07	
SeHyperBTM (**ISeU5**)	$(pyr)_2CH^+$	–10.46	2.37×10^4	14.83	14.75
	$(thq)_2CH^+$	–10.92	5.48×10^3	14.66	
TeDHPB (**ITeU3**)	$(lil)_2CH^+$	–12.76	2.37×10^4	17.13	17.13
TeBTM (**ITeU2**)	$(lil)_2CH^+$	-12.76	2.14×10^4	17.09	17.09
7-AnTM (**MeO-ITU2**)	$(jul)_2CH^+$	-12.62	4.01×10^4	15.22	15.22
7-F-BTM (**F-ITU2**)	$(pyr)_2CH^+$	-10.46	5.25×10^3	14.18	14.18

13. Determination of pK_a Values for Isochalcogenoureas

13.1 General Information

All NMR experiments were recorded manually on a Bruker Avance DRX 500 MHz spectrometer with a broad band observe probe, which is the property of the Austro-Czech NMR-Research Center "RERI-uasb". The CSI experiments were carried out using the gradient phase encoding of *Wallace et al.*[22,23] The measurement parameters were based on the work of Wallace et al.[22,23] Water suppression was carried out using excitation sculping (Bruker Library zgesgp).[24] The phase encoding gradient pulse was 284 µs and varied from -61.5 to 61.5 G cm^{-1} in 128 increments. For all CSI measurements, 16 dummy scans were run before acquisition, and 8 scans acquired for each of the 128 slices, with an acquisition time of 1 s and a relaxation delay of 0.5 s. After acquisition a spoil gradient of 30.8 G cm^{-1} was included to destroy any remaining transverse magnetisation. The transformation of the time domain data files was carried out without zero filling in the indirect dimension but with zero filling in the direct dimension and sine bell apodisation. A total experiment took 28 minutes, and a theoretical spatial resolution of 0.17 mm was achieved. The measurements were referenced relative to the internal standard tetramethyl silane (TMS: δ = 0 ppm for ^{1}H-NMR in both DMSO-h_6 and acetonitrile-h_3). The shimming was done on the respective ^{1}H solvent peak. All experiments were carried out in non-deuterated solvents and therefore no lock was possible. All CSI experiments were measured at 20 °C.

The solid acids, the pK_a standards and purification agents were purchased from different commercial sources. Liquid amines, except 2-bromopyridine, were distilled twice, first over KOH (10 g L^{-1}) and second over NaH (10 g L^{-1}) and stored under argon in headspace vials prior to use. DMAP was ground in a mortar prior to use. The analytes and 2-bromopyridine were purchased from commercial sources and used as received.

Literature pK_a and experimentally determined limiting shifts (shifts of the fully protonated as well as the free base species) are given in tables S67 and S68. Due to varying accuracies of different pK_a determination methods, also accuracy (significant digits) of the given values may vary as well.

DMSO was pre-dried over Na_2SO_4 and then fractionally distilled once under reduced pressure over NaH (25 g L^{-1}). Acetonitrile was distilled twice, first over NaH (2 g L^{-1}) and then over P_2O_5 (2 g L^{-1}). Both solvents were stored under argon in headspace vials.

13.2 Datapoint Selection

For each chemical shift imaging experiment a total of 128 datapoints per analyte and internal standard were collected. However, due to the top and bottom 15-20 slices being at the edge of the NMR detection coil and thus being of inferior quality, those datapoints were neglected. Also, datapoints where both indicators are at their limiting shifts were disregarded as no definitive pH value could be determined from them. Therefore, the number of datapoints varies in each titration.

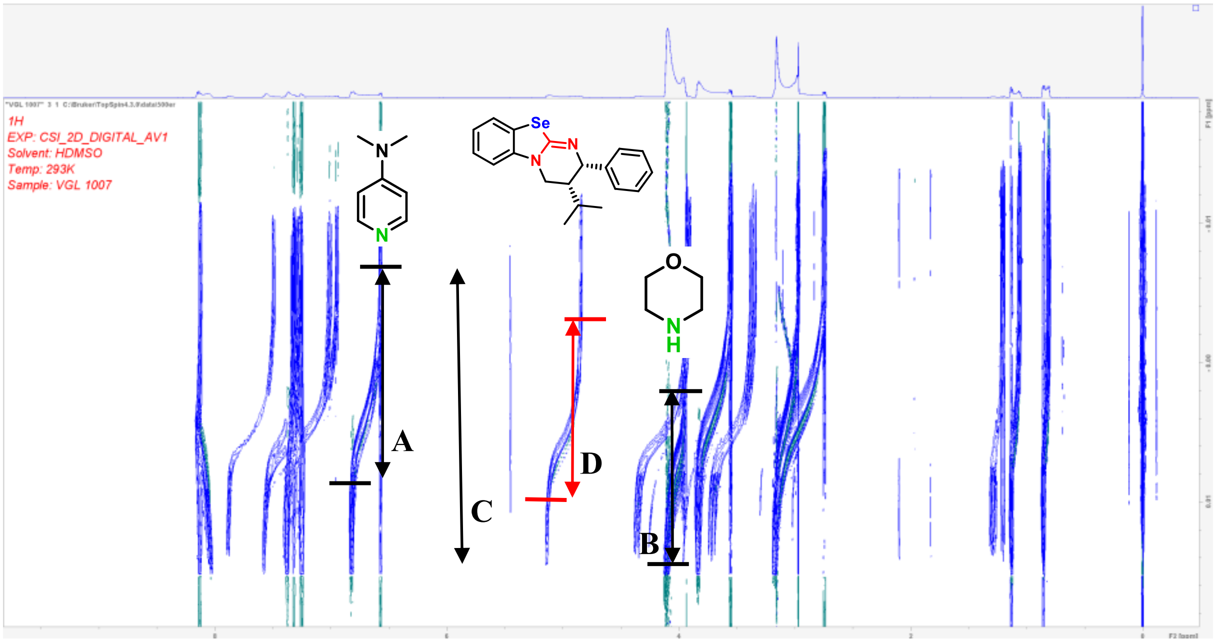

Figure S49: CSI experiment of SeHyperBTM. Arrow A shows the area for which the pH can be calculated from indicator 1 (DMAP). Arrow B shows the area for indicator 2 (morpholine) from which the pH can be calculated. Arrow C indicates the total area for which the pH can be calculated. Arrow D shows the area which is useful for the pKa determination of HyperBTM. Area D must be contained in area C otherwise only the parts of D which are, can be used for the calculation.

13.3 Sample Preparation

First, a stock solution containing the analyte, two basic indicators and tetramethyl silane was prepared with the respective solvent (DMSO or acetonitrile).

Then 5-10 mg of acid were weighed into a NMR tube and subsequently covered with glass beads.

1)

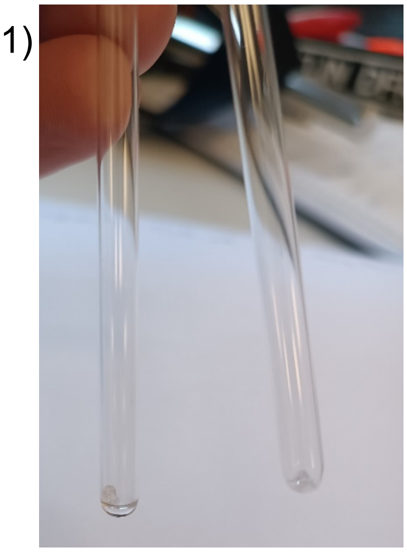

Figure S50: 5-10 mg of acid in NMR tube.

2)

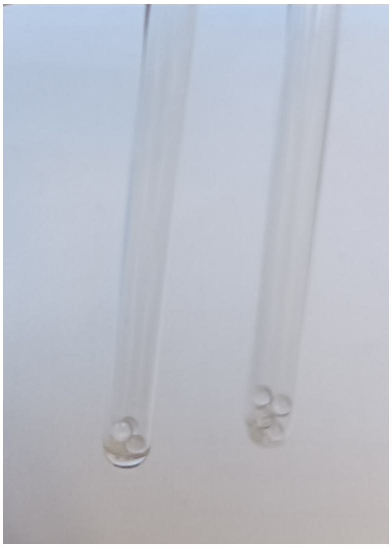

Figure S51: Cover with 1 mm glass beads.

Afterwards, 550 µL of the stock solution was layered carefully over the glass beads and the acid. The sample is then put in a 28 °C water bath (for DMSO) for 20 - 24h or left on the bench at 22 °C (for acetonitrile) for 1.5 - 2 h and then the NMR measurement was carried out at 20 °C.

3)

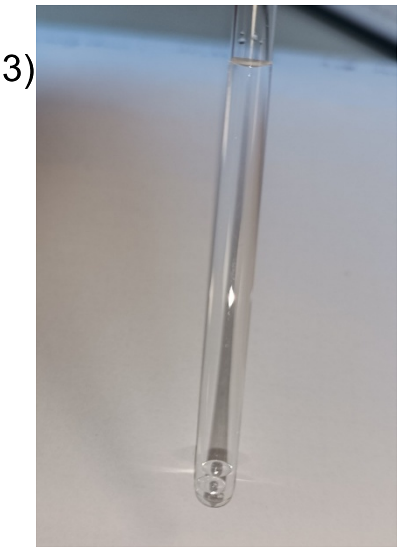

Figure S52: Overlay the acid with the stock solution of two indicators and the

4)

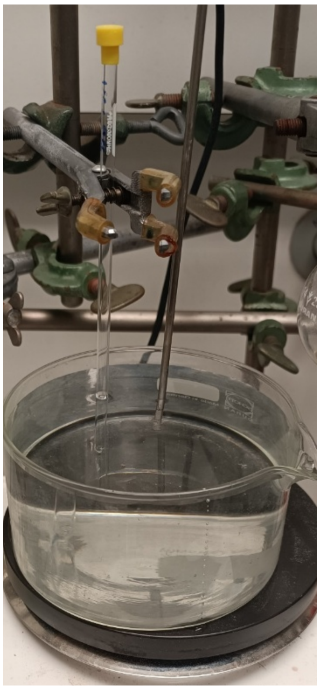

Figure S53: Development of gradients in temperated water

5)

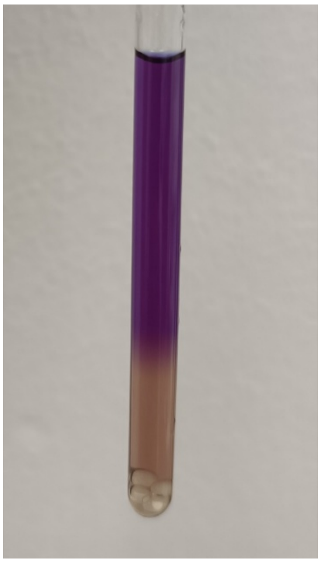

Figure S54: Visualized gradient after 18h in DMSO (with Bromthymolblue)

13.4 Indicator and Acid Data

Table S67: Limiting shifts (δ_H for the fully protonated and δ_L for the free base), pK_as and abbreviations of indicators and acids in acetonitrile.

Compound	Abbreviation	Literature pK_a	δ_H (ppm)	δ_L (ppm)
Tetramethylsilane	TMS	-	0	0
Morpholine	Morph	16.61[25]	3.8946	3.5370
4-(dimethylamino)pyridine	DMAP	17.95[26]	6.8195	6.5503
N-Ethyldiethanolamine	$Et(EtOH)_2N$	18.3[27]	1.2830	0.9993
Pyrrolidine	Pyr	19.56[28]	3.2364	2.7199
Diethyl amine	DEA	19.00[29]	1.2410	1.0211
Chloroacetic acid	ClAc	15.34[30]	-	-

Table S68: Limiting shifts (δ_H for the fully protonated and δ_L for the free base), pKas and abbreviations of indicators and acids in DMSO.

Compound	Abbreviation	Literature pK_a	δ_H (ppm)	δ_L (ppm)
Tetramethylsilane	TMS	-	0	0
Triethylamine	Et_3N	9.0[31]	1.1886	0.9257
Morpholine	Morph	9.2[32]	3.7728	3.4879
4-(dimethylamino)pyridine	DMAP	7.91[33]	6.9810	6.5780
Diethyl amine	DEA	10.5[31]	1.1659	0.9884
Proton Sponge	PrS	7.47[34]	7.7446	7.2854
Pyrrolidine	Pyr	11.06[32]	1.8415	1.5751
Meldrum's acid	Mel	7.3[35]	-	-
2,4-Dinitrobenzoic acid	DNB	6.5[36]	-	-

13.5 Titration Data

Table S69: Determined pK_a of analytes and titration data in acetonitrile. Conc. of titration components based on accuracy of weighing, small deviations possible.

Catalyst	Determined pK_a	Mass of acid (g)	δ_H (ppm)	δ_L (ppm)	Titration components (mmol L^{-1})
**IU3** - ODHBP	16.9	0.0073 ClAc	3.6735	3.4453	10 IU3, 20 DMAP, 20 Morph, 20 TMS
**ISeU3** - SeDHBP	17.9	0.0086 ClAc	7.8579	7.4508	10 ISeU3, 20 DMAP, 20 $Et(EtOH)_2N$, 20 TMS
**ITeU3** - TeDHBP	19.7	0.0069 ClAc	7.3127	7.0243	3.6 ITeU3, 10 DEA, 20 Pyr, 20 TMS
**ISeU2** - SeBTM	17.3	0.0084 ClAc	4.8801	4.2829	10 ISeU2, 20 DMAP, 20 $Et(EtOH)_2N$, 20 TMS
**ITeU2** - TeBTM	18.6	0.0083 ClAc	4.8460	4.2362	3 ITeU2, 10 DMAP, 10 DEA, 20 TMS
**ISeU1** - SeTM	17.9	0.0074 ClAc	5.6519	5.3203	10 ISeU1, 20 DMAP, 20 $Et(EtOH)_2N$, 20 TMS
**IU5** - OHyperBTM	15.8	0.0078 ClAc	5.1950	4.8290	10 IU5, 20 DMAP, 20 Morph, 20 TMS
**ISeU5** - SeHyperBTM	17.4	0.0056 ClAc	5.1455	4.8272	10 ISeU5, 20 DMAP, 20 Morph, 20 TMS

Table S70: Determined pK_a of analytes and titration data in DMSO. Conc. of titration components based on accuracy of weighing, small deviations possible.

Catalyst	Determined pK_a	Mass of acid (g)	δ_H (ppm)	δ_L(ppm)	Titration components (mmol L^{-1})
**IU3** - ODHBP	6.5	0.0092 DNB	4.7149	3.7902	10 IU3, 20 DMAP, 20 Et_3N, 20 TMS
**ISeU3** - SeDHBP	8.3	0.0070 DNB	4.1630	3.7477	10 ISeU3, 14 Et_3N, 20 DMAP, 20 TMS
**ITeU3** - TeDHBP	11.0	0.0080 MeI	7.9890	7.6050	10 ITeU3, 20 Pyr, 20 DEA, 20 TMS
**ISeU2** - SeBTM	7.7	0.0075 DNB	5.8820	5.5427	10 ISeU2, 20 DMAP, 20 Et_3N, 20 TMS
**ITeU2** – TeBTM	9.3	0.0087 MeI	4.8754	4.1986	3 ITeU2, 10 DEA, 10 Morph, 20 TMS
**ISeU1** - SeTM	7.4	0.0072 DNB	5.6923	5.2969	10 ISeU1, 10 PrS, 10 Et_3N, 20 TMS
**IU5** - OHyperBTM	6.5	0.0074 DNB	5.1707	4.8069	10 IU5, 20 DMAP, 20 Morph, 20 TMS
**ISeU5** - SeHyperBTM	8.2	0.0067 MeI	5.2000	4.7800	10 ISeU5, 20 DMAP, 20 Morph, 20 TMS

14. DFT Calculations

14.1 General Information

For all initial structures, a conformational search was carried out using the OPLS4 force field as implemented in Macromodel.[37] The pre-optimized structures were then used as input for DFT calculations using Gaussian 16.[38] Geometry optimizations and frequency calculations were carried out using the PBE0 hybrid functional combined with the def2-TZVP basis set.[39,40] The Grimme D3 dispersion correction with Becke-Johnson damping was applied as well.[41,42,43] Thermochemical corrections to free energies (corr. ΔG) were calculated using the rigid rotor/harmonic oscillator model without scaling. All structures obtained were confirmed to be minima on the PES by absence of imaginary frequencies. Gibbs free energies of found conformers were Boltzmann weighted and conformers with less than 1 % contribution to ΔG_{298} were discarded. To obtain the final electronic energies, single point calculations using the wB97XD functional with the def2-TZVP basis set and with consideration of solvation effects by application of the SMD model for dichloromethane were carried out.[44, 45] Table S63 summarizes functional/basis set combinations that were also tested herein. Electrostatic potential maps of **IU3**, **ITU3**, **ISeU3** and **ITeU3** (SMD(DCM)/wb97XD/def2-TZVP//PBE0-D3BJ/def2-TZVP) are shown as well.

Table S71: Different combinations of functionals and basis sets evaluated by the obtained quality of the correlation of experimental *N* values with calculated MCAs (in kJ mol^{-1}; see below). In all cases, the SMD solvation model for DCM was specified; a) Te is not defined in 6-311+G(d,p).

functional / basis set	**IU3**	**ITU3**	**ISeU3**	**ITeU3**	R^2 (*N* vs. MCA)
PBE0-D3BJ/def2-TZVP	413.4	420.3	428.0	434.8	0.9836
M06-2X/6-311+G(d,p)[46, 47]	381.3	384.9	393.8	– [a]	0.9998
M06-2X/def2-TZVP	386.8	392.7	400.5	407.2	0.9818
M06-2X/def2-QZVPP	385.4	391.9	400.0	406.6	0.9757
wB97XD/def2-TZVP	394.4	398.2	406.4	413.7	0.9982

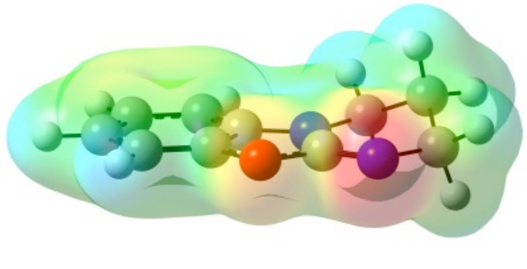

IU3

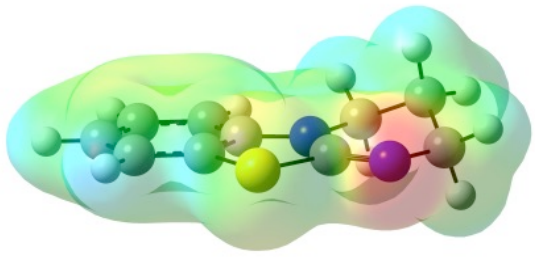

ITU3

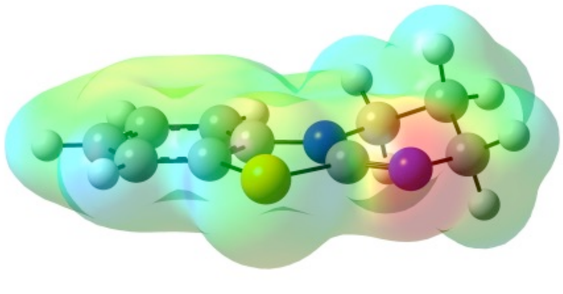

ISeU3

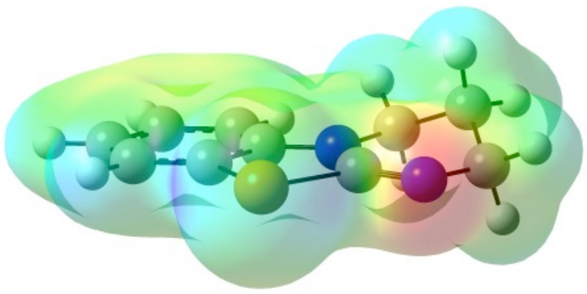

ITeU3

Figure S55: Electrostatic potential surfaces on the 0.01 au isodensity surface (SMD(DCM)/wb97XD/def2-TZVP//PBE0-D3BJ/def2-TZVP).

14.2 Methyl Cation Affinities and Acyl Ion Affinities

Methyl cation affinities (MCAs) are defined as the negative Gibbs free energies (-ΔG_R) of the addition of a nucleophile to the methyl cation at 298.15 K. Analogously, acyl ion affinities (AIA) are calculated as the negative Gibbs free energies (-ΔG_R) of the reaction of a nucleophile with the acyl ion (Scheme S1). Figure S52 gives an overview of the structures of all catalysts investigated. Boltzmann-weighted MCAs and AIAs are compiled in Table S64. Different correlations of MCA, AIA, *N* and *LB* are shown as well.

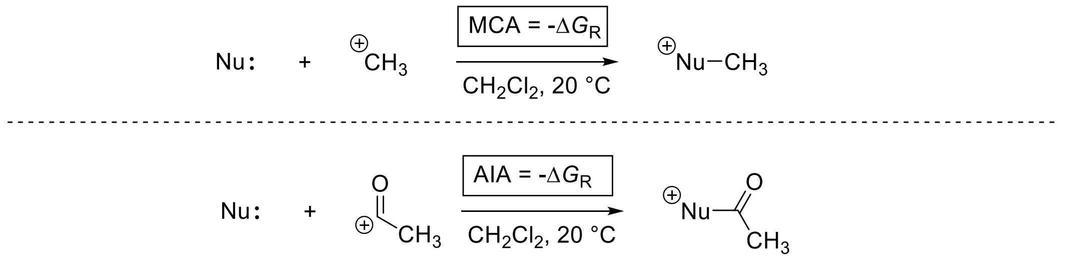

Scheme S1: Reference reactions for the determination of MCA and AIA.

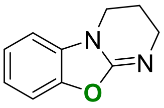

IU3
ODHPB

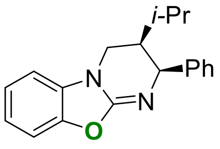

IU5
O*Hyper*BTM

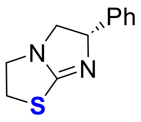

ITU1
TM

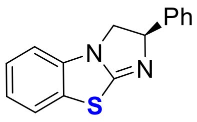

ITU2
BTM

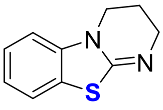

ITU3
DHPB

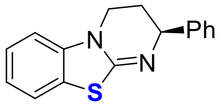

ITU4
HBTM

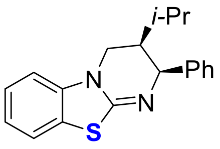

ITU5
*Hyper*BTM

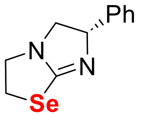

ISeU1
SeTM

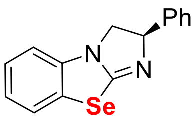

ISeU2
SeBTM

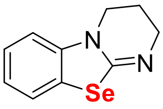

ISeU3
SeDHPB

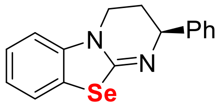

ISeU4
SeHBTM

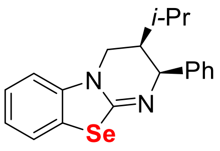

ISeU5
Se*Hyper*BTM

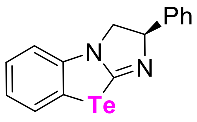

ITeU2
TeBTM

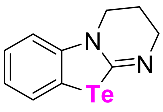

ITeU3
TeDHPB

Figure S56: Overview of the catalysts investigated within this computational study.

Table S72: Summary of *N* (in DCM), LB, Boltzmann-weighted MCAs, AIAs and frontier orbital energies of isochalcogenurea catalysts (and further compounds used as nucleophilic catalysts) calculated at the SMD(CH_2Cl_2)/wB97XD/def2-TZVP//PBE0-D3BJ/def2-TZVP level. MCAs for the methylation at the chalcogen atom are given in square brackets. Selected correlation diagrams are shown below.

label	filename	*N*	*LB*	MCA (kJ mol^{-1})	AIA (kJ mol^{-1})
**ITU5**	*Hyper*BTM	14.96	14.12	399.60	166.21
**ITU4**	HBTM	13.45	13.43	396.25	162.11
**ITU3**	SDHPB	13.86	15.39	398.19 [244.92]	166.74
**ITU2**	BTM	13.06	14.88	391.69	168.97
**ITU1**	TM	13.86	15.50	402.30	171.13
**ISeU5**	Se*Hyper*BTM	16.11	14.75	407.74	176.20
**ISeU4**	SeHBTM	14.42	14.03	403.45	172.29
**ISeU3**	SeDHPB	15.16	15.54	406.42 [270.46]	177.28
**ISeU2**	SeBTM	14.27	15.23	401.67	177.85
**ISeU1**	SeTM	15.26	-	411.78	182.00
**ITeU2**	TeBTM	14.62	17.09	409.20	191.65
**ITeU3**	TeDHPB	16.63	17.13	413.73 [299.64]	197.28
**IU5**	OHyperBTM	13.01	12.42	395.63	144.34
**IU3**	ODHPB	13.31	13.66	394.40	146.12

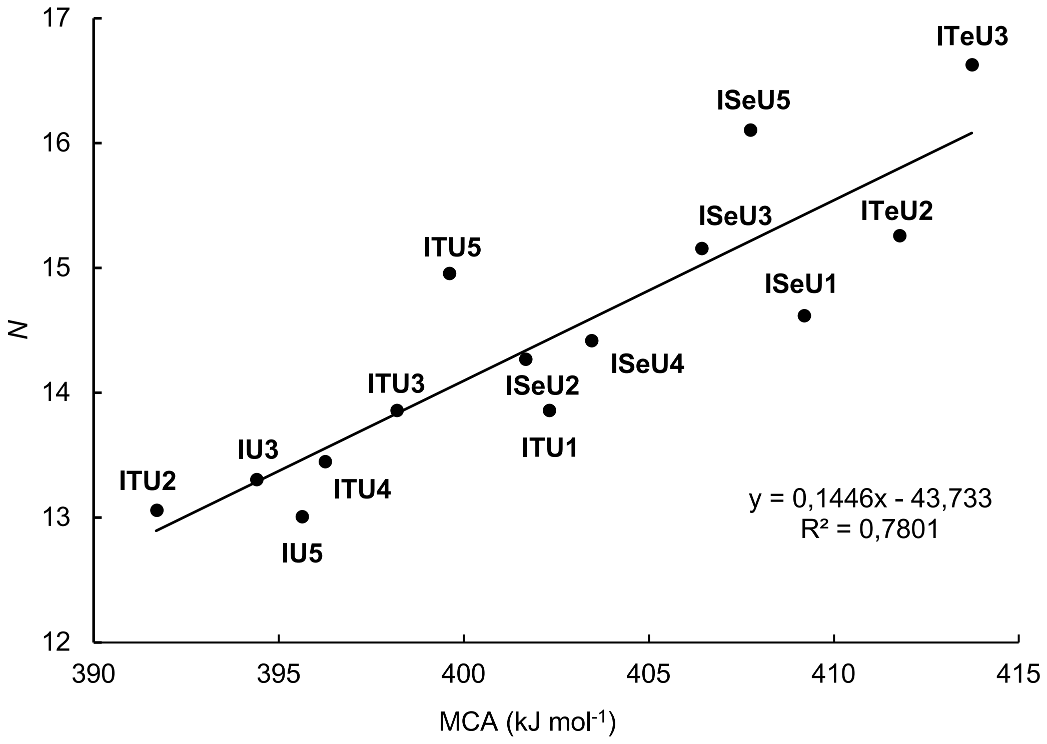

Figure S57: Plot of N vs MCA for all investigated IChUs.

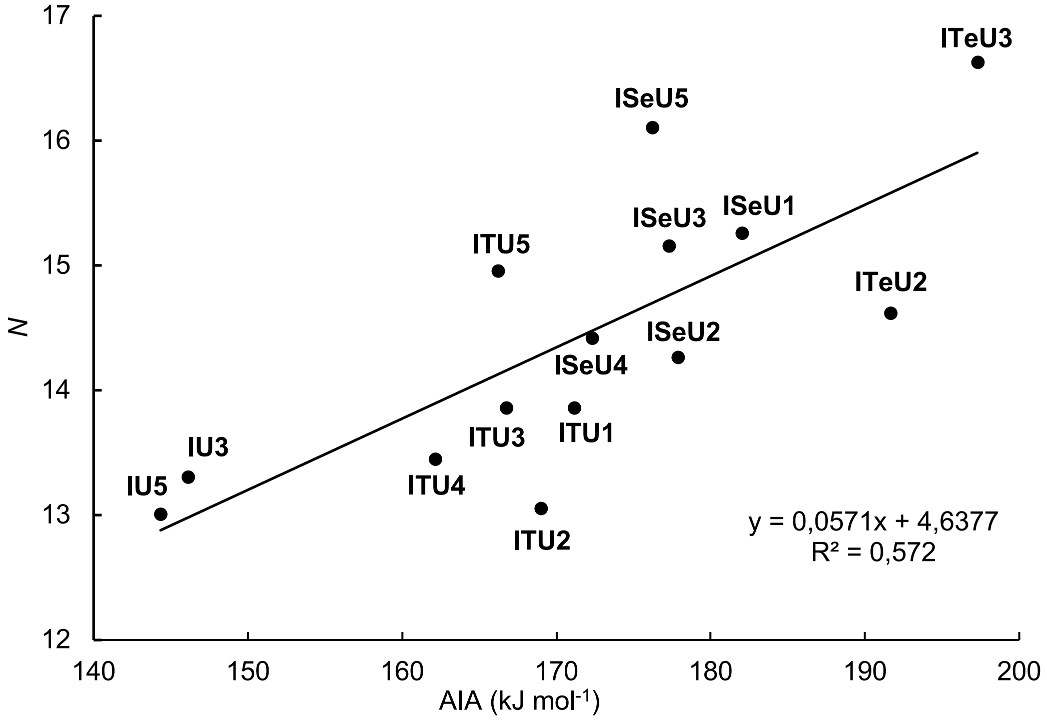

Figure S58: Plot of N vs AIA for all investigated IChUs.

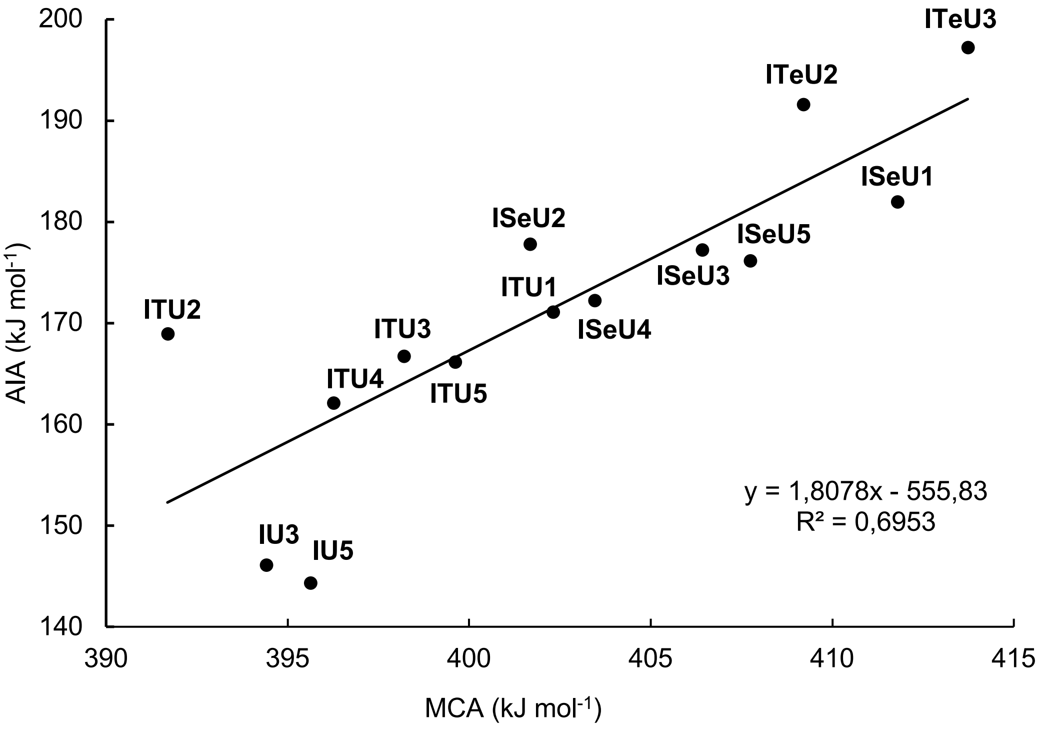

Figure S59: Plot of AIA vs MCA for all investigated IChUs.

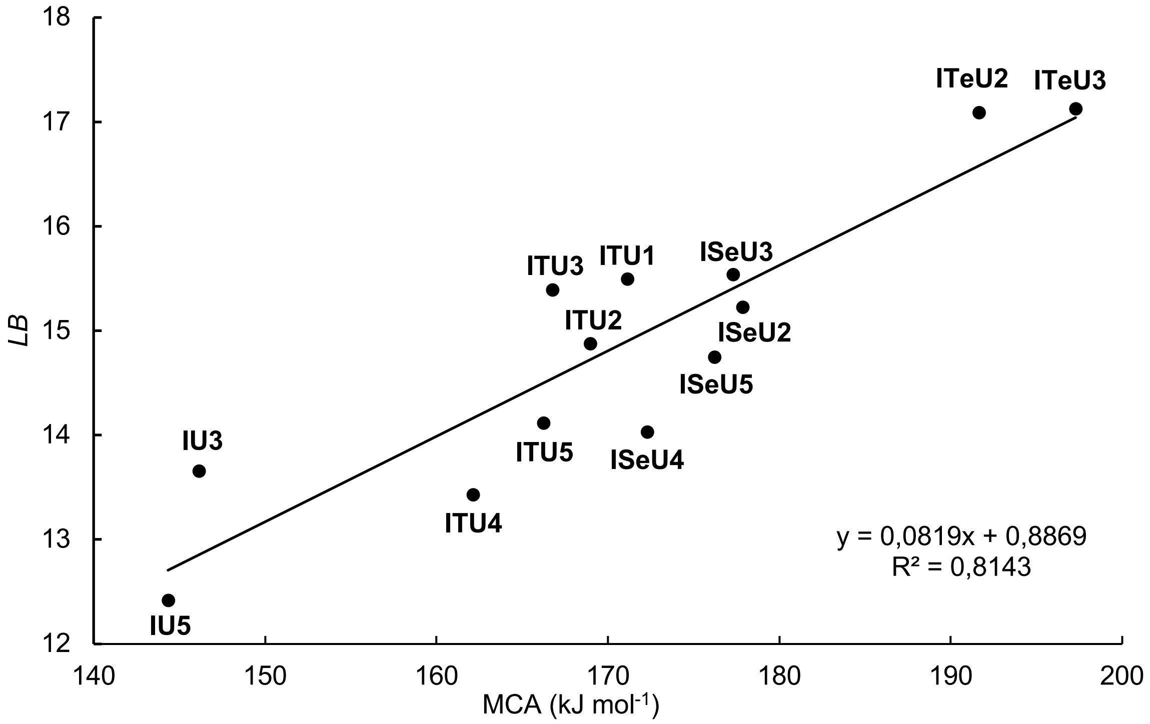

Figure S60: Plot of LB vs MCA for all investigate IChUs.

14.3 Summary of DFT-Calculated Data

Table S73: Summary of electronic energies, thermochemical corrections, frontier orbital energies and buried volumes of conformers of different nucleophiles and methylated products calculated at the SMD(CH_2Cl_2)/wB97XD/def2-TZVP//PBE0-D3BJ/def2-TZVP level. Values are given in Hartee; a) Geometries of hypothetical products resulting from methylation at the chalcogen atom.

label	filename	$E_{gas}^{PBE0-D3BJ}$	E_{SMD}^{wB97XD}	Corr. ZPE	Corr. D*E*	Corr. D*H*	Corr. D*G*	weighting	e_{HOMO}	e_{LUMO}
**M**	methylcation	-39.43404665	-39.60039063	0.031260	0.034114	0.035058	0.013864	1.0000		
**A**	AcCat	-152.81078055	-153.040359352	0.044661	0.048205	0.049149	0.020629	1.0000		
**IU5**	OHyperBTM_1	-920.10028174	-920.86102104	0.354007	0.372080	0.373025	0.307416	0.2258	-0.27510	0.05008
	OHyperBTM_2	-920.10036844	-920.86229680	0.354194	0.372262	0.373206	0.307735	0.6221	-0.27591	0.04998
	OHyperBTM_3	-920.09912996	-920.86115993	0.354286	0.372306	0.373250	0.308213	0.1125	-0.27524	0.05037
	OHyperBTM_4	-920.09717713	-920.85953961	0.354383	0.372417	0.373361	0.308370	0.0171	-0.27519	0.04954
	OHyperBTM_6	-920.09894056	-920.85954939	0.354244	0.372147	0.373091	0.308124	0.0225	-0.27585	0.04993
	OHyperBTM_M_1	-959.78387469	-960.63878642	0.396039	0.416082	0.417026	0.347225	0.8564		
	OHyperBTM_M_2	-959.78238392	-960.63750351	0.396207	0.416180	0.417125	0.347835	0.1153		
	OHyperBTM_M_3	-959.78105295	-960.63630251	0.396266	0.416254	0.417198	0.347962	0.0283		
	OHyperBTM_A_1	-1073.04363989	-1073.98352553	0.405380	0.427055	0.427999	0.354251	0.6142		
	OHyperBTM_A_2	-1073.04177086	-1073.98197721	0.405494	0.427095	0.428039	0.354805	0.0663		
	OHyperBTM_A_3	-1073.04071258	-1073.98089003	0.405628	0.427244	0.428189	0.355026	0.0166		
	OHyperBTM_A_4	-1073.04192683	-1073.98267516	0.405369	0.427048	0.427992	0.354255	0.2485		
	OHyperBTM_A_5	-1073.03989373	-1073.98112985	0.405428	0.427069	0.428013	0.354143	0.0545		
**IU3**	ODHPB	-571.38709775	-571.85148229	0.188174	0.197552	0.198496	0.153329	1.0000	-0.27402	0.05311
	ODHPB_M	-611.06340879	-611.62791184	0.230216	0.241497	0.242441	0.193013	1.0000		
	DHPB_O_A_1	-724.32361152	-724.97357695	0.239551	0.252427	0.253372	0.199778	0.7503		
	DHPB_O_A_2	-724.32016966	-724.97224824	0.239493	0.252427	0.253371	0.199488	0.2497		
**ITU5**	HyperBTM_1	-1243.00190197	-1243.83925814	0.350833	0.369520	0.370465	0.303269	0.2707	-0.27147	0.04686
	HyperBTM_2	-1243.00228400	-1243.84054980	0.351069	0.369721	0.370665	0.303871	0.5620	-0.27261	0.04707
	HyperBTM_3	-1243.00097017	-1243.83936460	0.351115	0.369738	0.370682	0.304162	0.1177	-0.27217	0.04720
	HyperBTM_4	-1242.99910039	-1243.83783480	0.351195	0.369848	0.370792	0.304278	0.0206	-0.27192	0.04679
	HyperBTM_6	-1243.00081648	-1243.83800213	0.351067	0.369568	0.370512	0.304124	0.0289	-0.27215	0.04684
	HyperBTM_M_1	-1282.68810997	-1283.61872443	0.392887	0.413424	0.414368	0.343468	0.7512		

	HyperBTM_M_2	-1282.68656424	-1283.61761388	0.392892	0.413434	0.414378	0.343547	0.2131		
	HyperBTM_M_3	-1282.68525106	-1283.61634463	0.393051	0.413563	0.414507	0.343965	0.0357		
	HyperBTM_A_1	-1395.95890408	-1396.97170319	0.402811	0.424749	0.425693	0.352188	0.8546		
	HyperBTM_A_2	-1395.95690456	-1396.97010479	0.402839	0.424745	0.425689	0.352438	0.1207		
	HyperBTM_A_3	-1395.95534987	-1396.96878537	0.402912	0.424839	0.425783	0.352615	0.0247		
**ITU4**	HBTM_1	-1125.15923950	-1125.88925999	0.265880	0.280493	0.281437	0.222824	0.7222	-0.27239	0.04732
	HBTM_2	-1125.15988899	-1125.88903911	0.266352	0.280804	0.281749	0.223505	0.2778	-0.27367	0.04730
	HBTM_M_1	-1164.84024375	-1165.66441268	0.307853	0.324327	0.325271	0.262285	0.1358		
	HBTM_M_2	-1164.84222024	-1165.66665833	0.308108	0.324483	0.325427	0.262783	0.8642		
	HBTM_A	-1278.11298497	-1279.01951679	0.318157	0.335861	0.336805	0.271856	1.0000		
**ITU3**	SDHPB	-894.28830651	-894.82934258	0.185026	0.194977	0.195921	0.149346	1.0000	-0.27061	0.04836
	SDHPB_M	-933.96722668	-934.60709564	0.226946	0.238753	0.239697	0.188908	1.0000		
	DHPB_SM_1[a]	-933.90762741	-934.54564475	0.223950	0.236043	0.236987	0.185748	0.5529		
	DHPB_SM_2[a]	-933.90753297	-934.54542235	0.223955	0.236061	0.237005	0.185726	0.4471		
	DHPB_S_A_1	-1047.23661776	-1047.96053010	0.236914	0.250094	0.251038	0.197295	1.0000		
	DHPB_S_A_2	-1047.22550931	-1047.94896956	0.236425	0.249753	0.250697	0.195895	0.0000		
**ITU2**	BTM	-1085.86698472	-1086.56098573	0.236577	0.250228	0.251173	0.193966	1.0000	-0.27379	0.04610
	BTM_M	-1125.54694364	-1126.33655826	0.278350	0.293920	0.294865	0.233824	1.0000		
	BTM_A	-1238.81827897	-1239.69218173	0.287922	0.305156	0.306100	0.241076	1.0000		
**ITU1**	TM	-933.54705362	-934.12938092	0.213226	0.224500	0.225445	0.174277	1.0000	-0.30250	0.05725
	TM_M_1	-973.22864951	-973.90873702	0.254948	0.268284	0.269228	0.213731	0.5961		
	TM_M_2	-973.22765701	-973.90780067	0.254930	0.268352	0.269296	0.213162	0.4039		
	TM_A	-1086.49541084	-1087.26074498	0.264232	0.279308	0.280252	0.220731	1.0000		
**ISeU5**	SeHyperBTM_1	-3246.20069940	-3247.24733319	0.349853	0.368945	0.369889	0.301536	0.5713	-0.27040	0.04979
	SeHyperBTM_2	-3246.20014057	-3247.24606278	0.349611	0.368742	0.369686	0.300932	0.2820	-0.26923	0.04962
	SeHyperBTM_3	-3246.19938345	-3247.24612397	0.349969	0.369007	0.369951	0.301986	0.0986	-0.27006	0.04983
	SeHyperBTM_4	-3246.19753169	-3247.24460261	0.349980	0.369064	0.370008	0.301991	0.0196	-0.26985	0.04924
	SeHyperBTM_6	-3246.19914779	-3247.24486927	0.349905	0.368829	0.369773	0.301901	0.0286	-0.26930	0.04956
	SeHyperBTM_M_1	-3285.88617597	-3287.02856056	0.391621	0.412591	0.413535	0.341114	0.6871		
	SeHyperBTM_M_2	-3285.88465991	-3287.02743802	0.391590	0.412605	0.413550	0.340815	0.2872		
	SeHyperBTM_M_3	-3285.88333004	-3287.02606807	0.391794	0.412725	0.413669	0.341727	0.0256		
	SeHyperBTM_A_1	-3399.15962239	-3400.38268237	0.401629	0.423945	0.424890	0.350255	0.8721		

	SeHyperBTM_A_2	-3399.15764611	-3400.38103414	0.401698	0.423973	0.424917	0.350566	0.1095		
	SeHyperBTM_A_3	-3399.15611349	-3400.37958812	0.401789	0.424060	0.425005	0.350806	0.0184		
**ISeU4**	SeHBTM_1	-3128.35747086	-3129.29597098	0.264711	0.279740	0.280684	0.220562	0.7351	-0.27020	0.04998
	SeHBTM_2	-3128.35820297	-3129.29578737	0.265199	0.280060	0.281004	0.221342	0.2649	-0.27138	0.04999
	SeHBTM_M_1	-3168.04038922	-3169.07639662	0.306916	0.323667	0.324611	0.260840	0.8985		
	SeHBTM_M_2	-3168.03826049	-3169.07392362	0.306671	0.323512	0.324456	0.260426	0.1015		
	SeHBTM_A	-3281.31367271	-3282.43033985	0.316951	0.335041	0.335985	0.269834	1.0000		
**ISeU3**	SeDHPB	-2897.48650212	-2898.23583685	0.222196	0.234876	0.235820	0.182728	1.0000	-0.26857	0.05077
	SeDHPB_M	-2937.16513137	-2938.01662764	0.222221	0.234906	0.235850	0.182753	1.0000		
	DHPB_SeM_1[a]	-2937.10680172	-2937.96099399	0.222196	0.234876	0.235820	0.182728	0.5269		
	DHPB_SeM_2[a]	-2937.10675111	-2937.96091714	0.222221	0.234906	0.235850	0.182753	0.4731		
	DHPB_Se_A_1	-3050.43717209	-3051.37120858	0.235717	0.249269	0.250213	0.195274	1.0000		
	DHPB_Se_A_1	-3050.42266115	-3051.35872948	0.235216	0.248927	0.249871	0.193738	0.0000		
**ISeU2**	SeBTM	-3089.06660674	-3089.96971580	0.235386	0.249478	0.250422	0.191535	1.0000	-0.27055	0.04927
	SeBTM_M	-3128.74683318	-3129.74919100	0.277128	0.293118	0.294062	0.231496	1.0000		
	SeBTM_A	-3242.01991166	-3243.10469530	0.286705	0.304332	0.305277	0.239043	1.0000		
**ISeU1**	SeTM_1	-2936.74800079	-2937.53826069	0.211983	0.223639	0.224583	0.171816	0.7141	-0.29867	0.05638
	SeTM_2	-2936.74699890	-2937.53729321	0.212110	0.223740	0.224684	0.171713	0.2859	-0.29836	0.05736
	SeTM_M_1	-2976.43005054	-2977.32092600	0.253646	0.267377	0.268321	0.211219	0.5916		
	SeTM_M_2	-2976.42917980	-2977.32010899	0.253651	0.267449	0.268393	0.210752	0.4084		
	SeTM_A	-3089.69886794	-3090.67354196	0.262939	0.278402	0.279346	0.218295	1.0000		
**ITeU2**	TeBTM	-955.824790195	-956.44609097	0.234522	0.248906	0.249851	0.189692			
	TeBTM_M	-995.507665005	-996.22857909	0.276270	0.292523	0.293467	0.229798			
	TeBTM_A	-1108.78514335	-1109.58672095	0.285909	0.303758	0.304702	0.237597			
**ITeU3**	TeDHPB	-764.24190595	-764.70862487	0.183043	0.193651	0.194595	0.145456	1.0000	-0.26261	0.03410
	TeDHPB_M	-803.92285401	-804.49230942	0.224842	0.237286	0.238230	0.185032	1.0000		
	DHPB_TeM_1[a]	-803.87051104	-804.44395303	0.220653	0.233777	0.234721	0.179965	0.6122		
	DHPB_TeM_2[a]	-803.87046825	-804.44366312	0.220717	0.233826	0.234770	0.180106	0.3878		
	DHPB_Te_A_1	-917.20167073	-917.85198776	0.234979	0.248721	0.249665	0.193950	1.0000		
	DHPB_Te_A_2	-917.17968371	-917.83346310	0.234348	0.248297	0.249241	0.192073	0.0000		

Geometries of Optimized Structures

Filenames, number of imaginary frequencies (NImag) and cartesian coordinates of atoms (in Å) for all stationary points found at the PBE0-D3BJ/def2-TZVP level of theory are given below.

methylcation.log

NImag=0

C	0.0000000	0.0000000	0.0000000
H	0.0000000	1.0932390	0.0000000
H	0.9467730	-0.5466200	0.0000000
H	-0.9467730	-0.5466200	0.0000000

AcCat.log

NImag=0

O	-1.3242150	-0.0000020	-0.0000010
C	-0.2134170	0.0000080	0.0000020
C	1.2044830	0.0000050	-0.0000010
H	1.5490960	-0.5191370	-0.9040500
H	1.5490960	-0.5234060	0.9015850
H	1.5491370	1.0424840	0.0024640

HyperBTM_1.log

NImag=0

C	-3.6214056	0.5530222	-2.0647433
C	-2.3671822	0.2709096	-1.5445488
C	-2.2050385	0.0471112	-0.1815477
C	-3.3130828	0.1076729	0.6402233
C	-4.5808282	0.3902872	0.1157191
C	-4.7401430	0.6178623	-1.2421365
S	-3.3934158	-0.1312799	2.3647807
C	-5.1347799	0.1523241	2.3658430
N	-5.5635405	0.4050141	1.0820134
N	-5.8323733	0.0941899	3.4198607
C	-7.2428805	0.3843447	3.2720352
C	-7.8249120	-0.0606356	1.9103902
C	-6.9763552	0.5475840	0.7987418
C	-8.0682229	-1.5731893	1.7607921
C	-6.8161870	-2.4277960	1.6083365
C	-9.0338617	-1.8461546	0.6142862
C	-8.0407487	-0.1744141	4.4239001
C	-7.5191505	-1.1470429	5.2657195
C	-8.2902879	-1.6786320	6.2899801
C	-9.5907693	-1.2415587	6.4875319
C	-10.1154742	-0.2605632	5.6572399
C	-9.3434232	0.2678890	4.6357944
H	-3.7362777	0.7295126	-3.1274640
H	-1.5061584	0.2250810	-2.1998507
H	-1.2272384	-0.1733339	0.2295586
H	-5.7137079	0.8503549	-1.6551999
H	-7.3608163	1.4787578	3.3099730
H	-8.8102854	0.4095259	1.8326000
H	-7.2163494	1.6085205	0.6690783
H	-7.1834496	0.0483301	-0.1509369
H	-8.5678270	-1.8851586	2.6838931
H	-6.1382329	-2.3159525	2.4543405
H	-6.2665161	-2.1831570	0.6944027
H	-7.0969248	-3.4816227	1.5412666
H	-9.2844079	-2.9085230	0.5692641
H	-8.6022752	-1.5789634	-0.3556461
H	-9.9658163	-1.2871931	0.7330490
H	-6.4966473	-1.4689881	5.1129655
H	-7.8691892	-2.4375040	6.9400222
H	-10.1914326	-1.6558269	7.2890748
H	-11.1270535	0.0981272	5.8106294
H	-9.7584313	1.0414683	3.9961680

HyperBTM_2.log

NImag=0

C	-2.7804416	-1.2442084	-0.7173451
C	-1.8952003	-0.2003514	-0.4935796
C	-2.2635192	0.8744748	0.3086896
C	-3.5238595	0.8846094	0.8728345
C	-4.4211000	-0.1659859	0.6413393
C	-4.0502703	-1.2403928	-0.1516200
S	-4.2320381	2.0909131	1.9139187
C	-5.7303260	1.1641239	2.0415034
N	-5.6288802	0.0187605	1.2786720
N	-6.7056587	1.5437354	2.7536534
C	-7.8584074	0.6618591	2.8030836
C	-8.0420957	-0.1041612	1.4713952
C	-6.7651492	-0.8640766	1.1497517
C	-9.2811633	-1.0033355	1.4173778
C	-9.3059024	-1.8836427	0.1727060
C	-10.5606635	-0.1779220	1.4700839
C	-7.7940735	-0.2549415	4.0068491
C	-6.6002308	-0.8397619	4.4190723
C	-6.5703552	-1.7119951	5.4966508
C	-7.7338066	-2.0078174	6.1905790
C	-8.9259694	-1.4147772	5.8038549
C	-8.9509594	-0.5455262	4.7243293
H	-2.4816031	-2.0797694	-1.3388929
H	-0.9094051	-0.2198087	-0.9416146
H	-1.5742063	1.6905987	0.4891355
H	-4.7313854	-2.0649685	-0.3213397
H	-8.7301151	1.3058211	2.9394476
H	-8.1578521	0.6700393	0.7005265
H	-6.7905392	-1.2278918	0.1216457
H	-6.6519049	-1.7318999	1.8122433
H	-9.2561198	-1.6612615	2.2937990
H	-9.2350776	-1.2800840	-0.7381904
H	-10.2437058	-2.4416742	0.1244758
H	-8.4949887	-2.6143561	0.1595927
H	-11.4377520	-0.8290163	1.4699223
H	-10.6311543	0.4745785	0.5941484
H	-10.6206518	0.4541500	2.3564783
H	-5.6757240	-0.5949811	3.9103505
H	-5.6286534	-2.1539688	5.8020091
H	-7.7088277	-2.6846681	7.0366850
H	-9.8396155	-1.6221638	6.3493961
H	-9.8863480	-0.0760558	4.4396821

HyperBTM_3.log

NImag=0

C	-2.9731045	-1.1594092	-0.9930530
C	-1.9916256	-0.2272094	-0.6912814
C	-2.2372214	0.7756754	0.2405915
C	-3.4734419	0.8285789	0.8536326
C	-4.4684479	-0.1072737	0.5425073
C	-4.2193080	-1.1120569	-0.3789526
S	-4.0364644	1.9606792	2.0542065
C	-5.6063833	1.1549518	2.1426433

N	-5.6348721	0.1078136	1.2434834
N	-6.5153605	1.5305856	2.9402976
C	-7.7557680	0.7769714	2.9360554
C	-8.0280107	0.1415143	1.5563240
C	-6.8340036	-0.6873140	1.1160440
C	-9.3629016	-0.6127851	1.4343021
C	-9.3027837	-2.0869593	1.8199813
C	-9.9288343	-0.4678246	0.0265483
C	-7.8004144	-0.2078968	4.0803921
C	-6.7010992	-0.9959575	4.4075237
C	-6.7853391	-1.9469000	5.4128714
C	-7.9675968	-2.1156043	6.1187526
C	-9.0595490	-1.3135361	5.8227108
C	-8.9710055	-0.3655331	4.8153215
H	-2.7695233	-1.9410451	-1.7151297
H	-1.0259158	-0.2792308	-1.1787131
H	-1.4719725	1.5030629	0.4836750
H	-4.9759329	-1.8509511	-0.6116246
H	-8.5617479	1.4978797	3.1102849
H	-8.0794867	0.9980141	0.8729138
H	-6.9439887	-0.9849114	0.0701004
H	-6.7424796	-1.5990067	1.7156872
H	-10.0617432	-0.1198627	2.1205942
H	-8.7267680	-2.6635242	1.0901418
H	-10.3111422	-2.5070176	1.8345923
H	-8.8625518	-2.2453676	2.8046577
H	-10.8899372	-0.9798923	-0.0631048
H	-9.2560155	-0.9068285	-0.7169137
H	-10.0792243	0.5815603	-0.2380793
H	-5.7622409	-0.8537180	3.8852504
H	-5.9191272	-2.5532103	5.6524733
H	-8.0319039	-2.8568196	6.9068276
H	-9.9817368	-1.4216305	6.3823316
H	-9.8260259	0.2646895	4.5932330

HyperBTM_4.log

NImag=0

C	-2.7790107	-1.1089386	-0.9045561
C	-1.8640145	-0.1164378	-0.5866739
C	-2.2053326	0.8960274	0.3038995
C	-3.4689653	0.8968615	0.8607691
C	-4.3961642	-0.1011087	0.5338197
C	-4.0525751	-1.1141632	-0.3472048
S	-4.1471570	2.0283810	2.0007653
C	-5.6748184	1.1412281	2.0415977
N	-5.6030184	0.0668226	1.1767681
N	-6.6399900	1.4902917	2.7825163
C	-7.8310366	0.6532003	2.7641705
C	-8.0111206	-0.0133343	1.3831758
C	-6.7585861	-0.7791968	0.9945694
C	-9.2783641	-0.8442265	1.1268049
C	-10.5398224	-0.0528864	1.4439295
C	-9.3229291	-2.2182135	1.7873490
C	-7.8208960	-0.2894164	3.9466448
C	-8.9234185	-0.3553635	4.7918133
C	-8.9568611	-1.2388004	5.8597545
C	-7.8743277	-2.0688278	6.1070333
C	-6.7534954	-1.9872471	5.2935265
C	-6.7241085	-1.0990224	4.2301076
H	-2.5011396	-1.8965053	-1.5947142
H	-0.8758539	-0.1278303	-1.0297523
H	-1.4924124	1.6711573	0.5582440
H	-4.7580836	-1.8983527	-0.5922296
H	-8.6795858	1.3270679	2.9088253
H	-8.0733111	0.8383420	0.6927616
H	-6.8176198	-1.0699460	-0.0583984
H	-6.6497893	-1.6938314	1.5867408
H	-9.2782683	-1.0178135	0.0416296
H	-10.6763422	0.0664378	2.5205708
H	-11.4217740	-0.5711179	1.0606400
H	-10.5133312	0.9432568	0.9940523
H	-10.2310024	-2.7411645	1.4766044
H	-9.3333202	-2.1505656	2.8753177
H	-8.4765465	-2.8450701	1.4996876
H	-9.7698470	0.2987238	4.6111277
H	-9.8281029	-1.2733154	6.5039897
H	-7.8952374	-2.7614657	6.9403218
H	-5.8903682	-2.6115627	5.4950254
H	-5.8240516	-1.0213734	3.6318757

HyperBTM_6.log

NImag=0

C	-3.5544691	0.6163167	-2.0537377
C	-2.3104741	0.3104544	-1.5220483
C	-2.1707423	0.0525971	-0.1627656
C	-3.2905548	0.1030523	0.6439066
C	-4.5479514	0.4090423	0.1073148
C	-4.6846874	0.6712995	-1.2466468
S	-3.3967118	-0.1765923	2.3615660
C	-5.1350145	0.1254428	2.3460213
N	-5.5436354	0.4068229	1.0603414
N	-5.8501042	0.0703856	3.3898185
C	-7.2604005	0.3612896	3.2175657
C	-7.7755311	-0.1439723	1.8471191
C	-6.9548367	0.5826521	0.7895527
C	-7.7451581	-1.6878425	1.7395217
C	-7.3640729	-2.2178438	0.3620473
C	-9.0885082	-2.2839694	2.1459309
C	-8.0648312	-0.1627534	4.3782710
C	-7.5775439	-1.1628754	5.2078218
C	-8.3658142	-1.6745999	6.2291703
C	-9.6455370	-1.1847978	6.4391461
C	-10.1307804	-0.1679360	5.6276038
C	-9.3425780	0.3386616	4.6079640
H	-3.6518722	0.8195254	-3.1134181
H	-1.4401514	0.2729665	-2.1654664
H	-1.2010379	-0.1857761	0.2574285
H	-5.6502098	0.9213294	-1.6683315
H	-7.3874011	1.4543571	3.2161380
H	-8.8096457	0.1932002	1.7224224
H	-7.2032970	1.6495973	0.7867249
H	-7.1662657	0.1924840	-0.2060743
H	-6.9876315	-2.0457669	2.4455315
H	-6.3676598	-1.9033405	0.0449871
H	-7.3686929	-3.3100812	0.3767963
H	-8.0835116	-1.9046212	-0.4016576
H	-9.3962849	-1.9702489	3.1427135
H	-9.8678437	-1.9794628	1.4392604
H	-9.0416764	-3.3756849	2.1362657
H	-6.5698483	-1.5248267	5.0459541
H	-7.9749879	-2.4593500	6.8672313
H	-10.2597094	-1.5844167	7.2378677
H	-11.1243826	0.2329558	5.7931703
H	-9.7272528	1.1337262	3.9756713

HyperBTM_M_1.log

NImag=0

C	0.0302725	5.2647629	4.1558760
C	1.2115790	5.1586757	3.4280273
C	1.3231427	4.2571208	2.3818898
C	0.2242844	3.4684860	2.0900406
C	-0.9563673	3.5678444	2.8227499
C	-1.0694892	4.4734502	3.8643403
S	0.0708430	2.2741139	0.8283564
C	-1.5444035	1.9337796	1.3303559

N	-1.9321928	2.6818127	2.3690442
N	-2.3155057	1.0404879	0.7429634
C	-3.7267204	0.9213107	1.1274812
C	-3.8587718	1.2153108	2.6308076
C	-3.2633284	2.5732489	2.9473920
C	-5.2983000	1.0810697	3.1434389
C	-5.4359330	1.5674567	4.5815021
C	-5.7792076	-0.3610302	3.0480775
C	-1.8703209	0.3745651	-0.4664257
C	-4.6065138	1.7518573	0.2224643
C	-4.2683344	3.0540395	-0.1368627
C	-5.1097814	3.8039805	-0.9436756
C	-6.2991160	3.2603169	-1.4050462
C	-6.6360801	1.9589110	-1.0666710
C	-5.7914952	1.2093503	-0.2630054
H	-0.0353142	5.9800148	4.9660809
H	2.0552546	5.7892468	3.6780496
H	2.2397338	4.1748639	1.8114851
H	-1.9857793	4.5699781	4.4321638
H	-3.9763682	-0.1299665	0.9764508
H	-3.2480156	0.4627051	3.1466655
H	-3.1588357	2.6962138	4.0237819
H	-3.8899373	3.3858361	2.5655347
H	-5.9366945	1.7069491	2.5095026
H	-6.4463902	1.3721861	4.9434092
H	-5.2651580	2.6408631	4.6846923
H	-4.7440411	1.0416575	5.2470019
H	-5.1696938	-1.0144354	3.6792984
H	-5.7523382	-0.7576397	2.0322360
H	-6.8108982	-0.4384399	3.3942148
H	-0.8270322	0.0696764	-0.3656384
H	-1.9822468	1.0217659	-1.3399910
H	-2.4734888	-0.5196882	-0.6104451
H	-3.3334104	3.4959791	0.1908114
H	-4.8316178	4.8145538	-1.2178425
H	-6.9553685	3.8459563	-2.0372089
H	-7.5554378	1.5208269	-1.4360713
H	-6.0587818	0.1881757	-0.0146024

HyperBTM_M_2.log

NImag=0

C	0.0113532	5.2060461	4.2555999
C	1.1893474	5.1583517	3.5161929
C	1.3137992	4.3043638	2.4323928
C	0.2315045	3.5026523	2.1150616
C	-0.9456110	3.5432160	2.8592434
C	-1.0717885	4.4019632	3.9384072
S	0.0968959	2.3588379	0.8052966
C	-1.5072869	1.9611925	1.3013516
N	-1.9033102	2.6541140	2.3749664
N	-2.2586594	1.0764558	0.6756846
C	-3.6541781	0.8618609	1.0795717
C	-3.7774919	1.1303912	2.5854911
C	-3.2403121	2.5042024	2.9293443
C	-5.1896561	0.8817946	3.1487262
C	-6.1286775	2.0798549	3.0646710
C	-5.0975536	0.3842649	4.5860168
C	-1.7952229	0.4779026	-0.5624179
C	-4.6007496	1.6200636	0.1846736
C	-4.3660077	2.9438416	-0.1757417
C	-5.2854631	3.6342283	-0.9495573
C	-6.4464843	3.0062401	-1.3764156
C	-6.6759285	1.6805006	-1.0410964
C	-5.7533979	0.9903674	-0.2706607
H	-0.0648491	5.8852229	5.0953841
H	2.0199350	5.7976871	3.7869023
H	2.2275511	4.2679403	1.8527405
H	-1.9857492	4.4536335	4.5157064
H	-3.8380653	-0.2066478	0.9387087
H	-3.1092329	0.4011056	3.0589290
H	-3.1697927	2.6229943	4.0106276
H	-3.8778522	3.2999151	2.5361368
H	-5.6239037	0.0742332	2.5482549
H	-7.1344954	1.7750051	3.3579595
H	-6.1922994	2.5022335	2.0616621
H	-5.8261853	2.8724476	3.7550257
H	-4.6409333	1.1333725	5.2400734
H	-4.5082537	-0.5325003	4.6599774
H	-6.0921040	0.1756237	4.9836830
H	-0.7668009	0.1279682	-0.4505723
H	-1.8548703	1.1862044	-1.3925169
H	-2.4260560	-0.3792149	-0.7879679
H	-3.4566921	3.4490904	0.1332658
H	-5.0927972	4.6644076	-1.2240836
H	-7.1646573	3.5462878	-1.9812723
H	-7.5721622	1.1791738	-1.3860227
H	-5.9353548	-0.0486902	-0.0163039

HyperBTM_M_3.log

NImag=0

C	0.1619139	5.2120695	4.1991413
C	1.3030108	5.1473923	3.4052218
C	1.3686649	4.2834539	2.3240294
C	0.2653644	3.4896531	2.0645398
C	-0.8745493	3.5476341	2.8631525
C	-0.9421721	4.4154819	3.9402166
S	0.0573896	2.3373762	0.7721485
C	-1.5242740	1.9564226	1.3482903
N	-1.8620838	2.6631999	2.4332599
N	-2.3125872	1.0716271	0.7701542
C	-3.7085387	0.9159006	1.2120071
C	-3.7564302	1.1712053	2.7252398
C	-3.1646468	2.5229585	3.0657879
C	-5.1097367	0.9534622	3.4234581
C	-5.6618445	-0.4365550	3.1400495
C	-6.1677124	2.0234698	3.1788120
C	-1.9177068	0.4560184	-0.4827756
C	-4.6285575	1.7204680	0.3271013
C	-4.4191962	3.0758836	0.0854020
C	-5.2979389	3.7922188	-0.7111159
C	-6.3916129	3.1605594	-1.2852671
C	-6.5903494	1.8047911	-1.0759027
C	-5.7084635	1.0900147	-0.2804220
H	0.1318652	5.8983100	5.0360956
H	2.1512014	5.7810378	3.6310269
H	2.2538055	4.2337759	1.7024856
H	-1.8271105	4.4788083	4.5601195
H	-3.9395216	-0.1407623	1.0621311
H	-3.0794314	0.4187551	3.1498253
H	-3.0249551	2.6031135	4.1448932
H	-3.8064109	3.3457271	2.7420001
H	-4.8647327	0.9862734	4.4928521
H	-6.4977165	-0.6524156	3.8070899
H	-4.9080745	-1.2136738	3.2933066
H	-6.0401636	-0.5198146	2.1188771
H	-6.4971420	2.0513044	2.1402312
H	-5.8274081	3.0243004	3.4523344
H	-7.0419412	1.8077081	3.7959756
H	-0.8694445	0.1545226	-0.4394853
H	-2.0714733	1.1353196	-1.3249907
H	-2.5223965	-0.4358357	-0.6348470
H	-3.5582731	3.5883696	0.5006900
H	-5.1252624	4.8469288	-0.8887582
H	-7.0784228	3.7219069	-1.9069187
H	-7.4298983	1.2994208	-1.5377685
H	-5.8668361	0.0278139	-0.1268948

HyperBTM_A_1.log

NImag=0

C	-4.42977500	1.84140800	1.05417000
C	-5.18770700	0.68351800	0.87612400
C	-4.61252600	-0.47629900	0.39102300
C	-3.25904500	-0.45099600	0.08516300
C	-2.50975600	0.70797400	0.25691000
C	-3.08135100	1.87281900	0.74861100
S	-2.28191800	-1.75684100	-0.52426300
C	-0.91327000	-0.72274900	-0.52901600
N	-1.18260800	0.51562300	-0.11753000
N	0.33569200	-1.10042800	-0.88396400
C	1.45436500	-0.15246000	-0.72879100
C	0.93849700	1.26161900	-1.03340400
C	-0.19323900	1.58455000	-0.08516600
C	2.04165400	2.32672900	-1.01866600
C	1.46671800	3.73505600	-1.11802200
C	3.03097200	2.10172200	-2.15508700
C	0.54962100	-2.44098200	-1.26252300
C	1.94723000	-2.85214400	-1.57964800
O	-0.39713800	-3.18540600	-1.31449700
C	2.13044400	-0.31412700	0.61221800
C	1.41859600	-0.61242400	1.77028900
C	2.07065300	-0.72429600	2.98912600
C	3.44289100	-0.54461400	3.06471000
C	4.16286200	-0.26298900	1.91342900
C	3.51057500	-0.15292800	0.69606600
H	-4.90417900	2.73440900	1.44130800
H	-6.24166700	0.69195500	1.12389400
H	-5.19886800	-1.37665100	0.25534800
H	-2.50087600	2.77346600	0.90013000
H	2.16699300	-0.40775700	-1.50976400
H	0.52084400	1.22984700	-2.04870300
H	-0.70231500	2.49669900	-0.38953000
H	0.16497200	1.70963200	0.94163800
H	2.57458100	2.24472300	-0.06490300
H	0.88153100	4.01630500	-0.24034400
H	0.83497700	3.84523400	-2.00497100
H	2.27665300	4.46078700	-1.20357500
H	3.82528900	2.84853900	-2.11743100
H	2.53412200	2.19820100	-3.12491600
H	3.51218500	1.12249700	-2.12361500
H	2.29773500	-2.36116400	-2.49146300
H	1.94361300	-3.92704800	-1.74038900
H	2.63329900	-2.59932100	-0.76910600
H	0.34835600	-0.78401800	1.73804200
H	1.50321400	-0.96156800	3.88093100
H	3.95192400	-0.63681100	4.01632000
H	5.23782300	-0.13719000	1.96026100
H	4.08554300	0.05525800	-0.19961300

HyperBTM_A_2.log

NImag=0

C	-4.40142600	1.90600700	1.03732300
C	-5.16046100	0.73587300	0.99614700
C	-4.60053900	-0.46194100	0.59262700
C	-3.26146400	-0.46239600	0.22761700
C	-2.51167300	0.70818100	0.26223000
C	-3.06751600	1.91162500	0.67251100
S	-2.30430300	-1.81687100	-0.30096400
C	-0.94151500	-0.78587900	-0.46024000
N	-1.20113600	0.48419800	-0.15051800
N	0.29100900	-1.19549200	-0.84075800
C	1.41859000	-0.24009100	-0.85216000
C	0.87358400	1.14717300	-1.20923000
C	-0.20535600	1.54310100	-0.23028200
C	1.96127900	2.22695300	-1.36989800
C	2.33380500	2.95092800	-0.08086800
C	1.54002200	3.23215200	-2.43487800
C	0.48409300	-2.56414200	-1.11909800
C	1.85656400	-3.00461100	-1.49913100
O	-0.46093600	-3.30891900	-1.04418500
C	2.22728400	-0.32350300	0.41635600
C	1.63037300	-0.44563200	1.66716400
C	2.40638300	-0.46244900	2.81611500
C	3.78666100	-0.36431500	2.72606700
C	4.39044000	-0.26522000	1.48129800
C	3.61415900	-0.25120400	0.33385600
H	-4.86333800	2.82948000	1.36324100
H	-6.20289500	0.76519200	1.28729100
H	-5.18733500	-1.37171400	0.56377400
H	-2.48602700	2.82311900	0.71812500
H	2.05536700	-0.53964500	-1.68441300
H	0.39317200	1.02493800	-2.18763700
H	-0.71357900	2.44783400	-0.56324000
H	0.19852900	1.72061700	0.76931100
H	2.85713400	1.71384800	-1.73771100
H	2.59411800	2.27170800	0.73122500
H	1.52245400	3.60106000	0.25865800
H	3.19668700	3.59396900	-0.26092700
H	0.62442800	3.75789000	-2.14736900
H	1.36373000	2.75046300	-3.39926200
H	2.31376400	3.98866800	-2.57516500
H	2.14044700	-2.58988700	-2.47004500
H	1.84337900	-4.08892800	-1.57208600
H	2.59804000	-2.68948200	-0.76295100
H	0.55452800	-0.54700700	1.76198700
H	1.93060400	-0.55969000	3.78448900
H	4.39181700	-0.37973100	3.62424600
H	5.46923500	-0.20628100	1.40245600
H	4.09324100	-0.17974600	-0.63732200

HyperBTM_A_3.log

NImag=0

C	4.49426400	-2.04761100	0.67252200
C	5.24185500	-0.87199300	0.75072800
C	4.66218800	0.35832700	0.50263800
C	3.31450700	0.38630300	0.17198200
C	2.57616100	-0.78894800	0.08752500
C	3.15192000	-2.02583600	0.34051500
S	2.33353700	1.78155800	-0.17651700
C	0.97568000	0.76085300	-0.42054600
N	1.25444700	-0.53397000	-0.26718900
N	-0.27023600	1.19664500	-0.71901100
C	-1.39627000	0.23508100	-0.76902900
C	-0.85002700	-1.08185400	-1.33575400
C	0.27215900	-1.59013100	-0.46412600
C	-1.87079800	-2.18778000	-1.65454600
C	-2.97524700	-1.67783700	-2.56937000
C	-2.44330700	-2.93367500	-0.45476500
C	-0.47724600	2.58474200	-0.84989500
C	-1.86114700	3.05407900	-1.14415200
O	0.46644900	3.32472000	-0.72511100
C	-2.11254700	0.17930000	0.55781800
C	-1.43189300	0.04702200	1.76484400
C	-2.12828800	-0.02832500	2.96114400
C	-3.51321900	0.03801200	2.96789700
C	-4.19881700	0.19856900	1.77340100
C	-3.50053700	0.27471300	0.57918900
H	4.97214900	-2.99743900	0.87705600
H	6.29132400	-0.92318100	1.01205600
H	5.24056800	1.27174200	0.56591900
H	2.57934100	-2.94282200	0.29112100
H	-2.08700600	0.62995200	-1.51166000

H	-0.40431200	-0.80447900	-2.29999500
H	0.77822500	-2 teal.42590200	-0.94893000
H	-0.08697800	-1.92334200	0.51241200
H	-1.29275200	-2.91813200	-2.23498400
H	-3.67648400	-1.03015400	-2.03884100
H	-3.55348400	-2.51498600	-2.96290000
H	-2.57370400	-1.12514400	-3.42326700
H	-3.04131500	-2.29008100	0.19003000
H	-1.66909700	-3.40012500	0.15798100
H	-3.09061200	-3.73764100	-0.80999500
H	-2.57265500	2.68644600	-0.40261500
H	-2.18696400	2.71333200	-2.13036700
H	-1.84618300	4.14077300	-1.13463000
H	-0.34826200	0.02309300	1.79447900
H	-1.58489600	-0.13108100	3.89268300
H	-4.05563500	-0.01940300	3.90364900
H	-5.27959900	0.27223300	1.77066800
H	-4.04567800	0.40685800	-0.34943100

HBTM_1.log

NImag=0

C	-3.0072270	-0.8283530	-0.0458310
C	-2.5617650	0.4969360	0.0383260
C	-4.3465510	-1.1341150	0.0945740
C	-5.2509460	-0.1019450	0.3197120
C	-4.8114790	1.2111240	0.3975750
C	-3.4648240	1.5254310	0.2559460
N	-1.1971260	0.6081280	-0.1209630
C	-0.5268630	-0.5753460	-0.3555480
S	-1.6762990	-1.9155660	-0.3374760
C	-0.4530160	1.8424230	0.0026230
C	0.9893830	1.5237710	0.3447390
C	1.5402790	0.4396890	-0.5844100
N	0.7102850	-0.7470240	-0.5642210
C	2.9699520	0.1169630	-0.2270730
C	3.2968020	-1.0356370	0.4739520
C	4.6122490	-1.2872890	0.8375110
C	5.6156000	-0.3914370	0.5020310
C	5.2971350	0.7592190	-0.2058950
C	3.9830190	1.0083580	-0.5667070
H	-4.6853480	-2.1611560	0.0304140
H	-6.3039390	-0.3288690	0.4318560
H	-5.5248500	2.0084690	0.5680730
H	-3.1320310	2.5546480	0.3075000
H	-0.9083910	2.4445710	0.7929450
H	-0.5196690	2.4130770	-0.9311430
H	1.0558130	1.1591980	1.3741420
H	1.5914130	2.4314470	0.2791510
H	1.5413250	0.8386570	-1.6104660
H	2.5085100	-1.7389580	0.7126210
H	4.8546590	-2.1930570	1.3820900
H	6.6432470	-0.5913100	0.7830000
H	6.0759650	1.4606000	-0.4832890
H	3.7414830	1.9052560	-1.1301390

HBTM_2.log

NImag=0

C	2.7585990	0.4990050	0.4193600
C	2.0631340	-0.5175710	-0.2476960
C	4.0437430	0.2916250	0.8803510
C	4.6386930	-0.9484510	0.6752350
C	3.9476010	-1.9583350	0.0224030
C	2.6537560	-1.7559380	-0.4434020
N	0.8012850	-0.1263920	-0.6401320
C	0.4362780	1.1549030	-0.2806680
S	1.7837920	1.9384080	0.5497020
C	-0.1268040	-0.9520960	-1.3812750
C	-1.1008590	-0.0503720	-2.1125190
C	-1.7014840	0.9783520	-1.1477980
N	-0.6715360	1.7404850	-0.4724400
C	-2.6547460	0.3102390	-0.1775600
C	-2.3284190	0.1033890	1.1561770
C	-3.2067480	-0.5591400	2.0036230
C	-4.4248860	-1.0179750	1.5296360
C	-4.7649560	-0.8049910	0.2002980
C	-3.8861450	-0.1459020	-0.6423930
H	4.5775980	1.0823030	1.3937480
H	5.6460160	-1.1237350	1.0322460
H	4.4173720	-2.9232410	-0.1260050
H	2.1171160	-2.5540520	-0.9409480
H	-0.6573520	-1.6251930	-0.6982240
H	0.4386110	-1.5628920	-2.0886900
H	-1.8843790	-0.6563460	-2.5701990
H	-0.5761440	0.4826320	-2.9105840
H	-2.2885900	1.6905620	-1.7370030
H	-1.3876580	0.4810360	1.5373630
H	-2.9367730	-0.7105350	3.0426890
H	-5.1115250	-1.5313450	2.1927220
H	-5.7210050	-1.1485540	-0.1783920
H	-4.1627460	0.0257340	-1.6785860

HBTM_M_1.log

NImag=0

C	-3.0485590	-0.7627450	0.0964890
C	-2.6470590	0.5691640	0.0190770
C	-4.3701270	-1.1059750	0.3219120
C	-5.2884860	-0.0791150	0.4670740
C	-4.8901370	1.2517060	0.3833870
C	-3.5669250	1.5953270	0.1572420
N	-1.2779040	0.6899040	-0.2127810
C	-0.6383660	-0.4805010	-0.3240450
S	-1.6916370	-1.8335220	-0.1332460
C	-0.5724560	1.9533500	-0.3637720
C	0.8713000	1.7557920	0.0373970
C	1.5191980	0.5861820	-0.6890610
N	0.6522540	-0.6057150	-0.5660650
C	1.1955110	-1.9310750	-0.8202820
C	2.8983610	0.3096590	-0.1542680
C	4.0070020	0.5292490	-0.9604110
C	5.2858540	0.3137120	-0.4663930
C	5.4596880	-0.1325280	0.8336210
C	4.3536500	-0.3599940	1.6423410
C	3.0787900	-0.1359230	1.1521470
H	-4.6775460	-2.1423480	0.3825740
H	-6.3292040	-0.3182330	0.6448960
H	-5.6266480	2.0375840	0.4940360
H	-3.2725120	2.6342310	0.0838600
H	-1.0513900	2.6865730	0.2848910
H	-0.6703170	2.2969130	-1.3977830
H	0.9333180	1.5864890	1.1152200
H	1.4432000	2.6577710	-0.1799300
H	1.6004180	0.8201520	-1.7570680
H	2.2102410	-1.8255120	-1.1938510
H	0.5912940	-2.4471040	-1.5702450
H	1.2285490	-2.5250480	0.0962160
H	3.8729790	0.8716960	-1.9815220
H	6.1455230	0.4906730	-1.1012450
H	6.4570500	-0.3058130	1.2191910
H	4.4871850	-0.7104090	2.6587180
H	2.2201530	-0.3182870	1.7911690

HBTM_M_2.log

NImag=0

C	-2.8198770	0.4180980	-0.4285180

C	-2.1942280	-0.6251630	0.2505570
C	-4.1133020	0.2960020	-0.9052430
C	-4.7698930	-0.9046230	-0.6900330
C	-4.1434570	-1.9506340	-0.0188960
C	-2.8488340	-1.8276010	0.4594740
N	-0.9004780	-0.2906720	0.6478530
C	-0.5250450	0.9428190	0.2892930
S	-1.7557110	1.7926520	-0.5690090
C	0.0007770	-1.1884080	1.3569960
C	1.0160520	-0.3634590	2.1141630
C	1.6927950	0.6495710	1.1966380
N	0.6611210	1.4607170	0.5489800
C	1.0179540	2.7598530	0.0085480
C	2.6287260	0.0113240	0.1969150
C	2.2228190	-0.3249860	-1.0889370
C	3.0984710	-0.9624370	-1.9564050
C	4.3860260	-1.2671660	-1.5449230
C	4.7994370	-0.9271130	-0.2645300
C	3.9260300	-0.2887140	0.5999490
H	-4.5966470	1.1102030	-1.4303120
H	-5.7823650	-1.0287500	-1.0525250
H	-4.6745880	-2.8818650	0.1327660
H	-2.3695210	-2.6515070	0.9720820
H	0.4817070	-1.8533940	0.6340490
H	-0.5943760	-1.7913180	2.0423010
H	1.7738530	-1.0212580	2.5386740
H	0.5264360	0.1625260	2.9379280
H	2.2658330	1.3387970	1.8218940
H	1.9491290	3.0807220	0.4698180
H	0.2441690	3.4937220	0.2451160
H	1.1614240	2.7136430	-1.0739570
H	1.2235970	-0.0827290	-1.4358410
H	2.7733190	-1.2160560	-2.9582350
H	5.0707050	-1.7609100	-2.2236350
H	5.8084850	-1.1522500	0.0590190
H	4.2589890	-0.0164980	1.5968750

HBTM_A.log

NImag=0

C	-2.89916000	0.30295100	-0.39957800
C	-2.31289400	-0.76380300	0.27295200
C	-4.20348200	0.22106000	-0.86693900
C	-4.89598700	-0.95489600	-0.64707500
C	-4.30036400	-2.02554500	0.02089500
C	-3.00200800	-1.94846000	0.49044700
N	-1.00887200	-0.46536900	0.65947200
C	-0.59987300	0.74785300	0.28680000
S	-1.80020500	1.64426100	-0.54828400
C	-0.17320900	-1.39596800	1.40829600
C	0.88793200	-0.61722100	2.14179400
C	1.62891400	0.29803600	1.17690300
N	0.65381700	1.19453100	0.53985300
C	1.02485900	2.46874800	0.06291100
O	0.19610600	3.13850500	-0.50003700
C	2.43202600	2.90491100	0.29239900
C	2.45334600	-0.46456600	0.16555800
C	2.04416900	-0.64780800	-1.14935400
C	2.82107200	-1.39245100	-2.02664300
C	4.01129200	-1.95547700	-1.59691800
C	4.42895400	-1.76838700	-0.28622600
C	3.65467700	-1.02618000	0.58903400
H	-4.66304200	1.05154400	-1.38838400
H	-5.91494800	-1.04704500	-1.00134100
H	-4.86346300	-2.93758300	0.17406800
H	-2.54762900	-2.78960600	0.99746300
H	0.26577900	-2.11405200	0.71029300
H	-0.81739600	-1.93257200	2.10389100
H	1.59843600	-1.30752400	2.59519200
H	0.43680100	-0.02376300	2.94146700
H	2.28862200	0.93465000	1.76605400
H	2.61266000	3.08160900	1.35616900
H	3.14799700	2.15914700	-0.05789000
H	2.57696200	3.83829800	-0.24529200
H	1.12515400	-0.20186300	-1.51358600
H	2.49457400	-1.52553000	-3.05096200
H	4.61902300	-2.53201900	-2.28350900
H	5.36497000	-2.19545600	0.05287100
H	3.99374100	-0.87536300	1.60953600

SDHPB.log

NImag=0

C	3.5098787	-0.2130719	0.0364454
C	2.9431983	-1.4758716	-0.0464211
C	1.5633846	-1.6405049	-0.0903859
C	0.7567922	-0.5141860	-0.0444591
C	1.3307354	0.7617276	0.0317014
C	2.7019368	0.9185379	0.0738231
N	-0.6201357	-0.4752762	-0.0708773
C	-1.1817741	0.7887865	-0.0526440
S	0.1021899	1.9980107	0.0563771
C	-1.4742794	-1.6421587	-0.1020378
C	-2.8396400	-1.2726562	0.4482758
C	-3.3473033	0.0049349	-0.2017221
N	-2.4064181	1.1023792	-0.1070664
H	4.5867474	-0.1026448	0.0690206
H	3.5807973	-2.3511174	-0.0815657
H	1.1307987	-2.6302800	-0.1669556
H	3.1397291	1.9077130	0.1344496
H	-1.0129308	-2.4259013	0.5040185
H	-1.5542156	-2.0192803	-1.1287262
H	-2.7605501	-1.1179037	1.5284938
H	-3.5348434	-2.0986652	0.2836213
H	-4.2846796	0.3170395	0.2651804
H	-3.5782171	-0.1788023	-1.2601888

DHPB_SM_1.log

NImag=0

C	-2.8002619	-4.9299993	-0.2135419
C	-2.4054851	-3.9616291	0.7029572
C	-1.1946061	-3.2924903	0.5907776
C	-0.3685361	-3.5941608	-0.4804544
C	-0.7820126	-4.5642589	-1.4016462
C	-1.9747540	-5.2524181	-1.2785535
N	0.8719229	-3.0495836	-0.7506875
C	1.5307100	-3.5718742	-1.8287606
S	0.4556654	-4.8162232	-2.6141155
C	1.5609929	-2.0280464	0.0286862
C	2.5399460	-1.3006886	-0.8798575
C	3.4175036	-2.2709067	-1.6562949
N	2.6521758	-3.3239628	-2.3152753
C	-0.0641748	-3.8789885	-4.0597927
H	-3.7456221	-5.4418445	-0.0913886
H	-3.0540375	-3.7271108	1.5383771
H	-0.9011475	-2.5597720	1.3311741
H	-2.2576773	-6.0171584	-1.9917333
H	2.0710047	-2.4994557	0.8744605
H	0.8150550	-1.3361710	0.4221658
H	1.9792168	-0.6768869	-1.5816994
H	3.1578386	-0.6319840	-0.2791112
H	3.9943270	-1.7491070	-2.4206265
H	4.1444395	-2.7638038	-1.0018949
H	-0.7857022	-4.4908453	-4.6002583
H	0.8388727	-3.7375188	-4.6559725
H	-0.4955536	-2.9298113	-3.7489341

DHPB_SM_2.log

NImag=0

C	-2.7929595	-4.9197682	-0.2035071
C	-2.4584518	-3.8642958	0.6372098
C	-1.2677748	-3.1637591	0.5001717
C	-0.3968594	-3.5310700	-0.5131578
C	-0.7547325	-4.5825864	-1.3654982
C	-1.9283739	-5.2975859	-1.2186307
N	0.8356772	-2.9708018	-0.7897002
C	1.5026319	-3.4962996	-1.8598933
S	0.5211755	-4.8798385	-2.5282853
C	1.4538001	-1.8569531	-0.0786574
C	2.9515623	-1.8734165	-0.3398560
C	3.2671982	-2.0324840	-1.8197500
N	2.5755125	-3.1747457	-2.4091430
C	-0.0169632	-4.1330402	-4.0736413
H	-3.7247031	-5.4519857	-0.0648377
H	-3.1417561	-3.5795616	1.4281183
H	-1.0298108	-2.3474610	1.1696851
H	-2.1684612	-6.1243678	-1.8760906
H	1.2495999	-1.9739106	0.9870397
H	0.9978751	-0.9199045	-0.4132989
H	3.4039387	-2.7012512	0.2128017
H	3.3901205	-0.9514296	0.0440899
H	4.3365991	-2.1740237	-1.9769868
H	2.9818681	-1.1396801	-2.3870561
H	-0.6683571	-4.8501898	-4.5718930
H	0.8954874	-3.9782397	-4.6522084
H	-0.5300431	-3.1951499	-3.8720243

SDHPB_M.log

NImag=0

C	-3.7618899	-0.2399408	0.0384142
C	-3.4193769	1.1061845	-0.0485334
C	-2.0950311	1.5120684	-0.0876852
C	-1.1177166	0.5322911	-0.0353752
C	-1.4623478	-0.8147601	0.0448425
C	-2.7847226	-1.2206601	0.0850934
N	0.2647612	0.7191810	-0.0591107
C	0.9663319	-0.4193051	-0.0081958
S	-0.0370201	-1.8185986	0.0681284
C	0.9066165	2.0263374	-0.1308311
C	2.3140109	1.9269551	0.4176702
C	3.0522446	0.7592485	-0.2002378
N	2.2867021	-0.4707744	-0.0216442
C	2.9723158	-1.7480897	-0.0716461
H	-4.8050329	-0.5279042	0.0672599
H	-4.2008358	1.8544914	-0.0886843
H	-1.8426125	2.5617529	-0.1641118
H	-3.0482774	-2.2688161	0.1491823
H	0.3121700	2.7226533	0.4607660
H	0.8976960	2.3675947	-1.1698736
H	2.2797520	1.8044815	1.5030605
H	2.8507725	2.8523672	0.2082099
H	4.0179845	0.6163681	0.2849264
H	3.2362685	0.9232248	-1.2681560
H	3.9893573	-1.6121979	0.2911469
H	3.0060910	-2.1434767	-1.0906013
H	2.4750287	-2.4691432	0.5797842

DHPB_S_A_1.log

NImag=0

C	-4.08680100	-0.77378700	-0.02699600
C	-3.99664400	0.61722000	-0.04193300
C	-2.77156200	1.26085200	-0.03951400
C	-1.63130600	0.47151200	-0.02313500
C	-1.72353900	-0.91501000	-0.01647300
C	-2.94945000	-1.56120200	-0.01328900
N	-0.30717100	0.90729600	-0.00984400
C	0.60101200	-0.07494700	-0.01956300
S	-0.14490400	-1.63100400	-0.01485900
C	0.01969700	2.33189000	0.04508700
C	1.45550100	2.50761300	0.46890900
C	2.33647100	1.56990900	-0.31625300
N	1.92222100	0.17624300	-0.08213700
C	2.99659800	-0.78040600	-0.04067000
O	4.09405900	-0.37867300	-0.29205200
C	2.71510000	-2.19698500	0.33538600
H	-5.06040400	-1.24725700	-0.02880700
H	-4.90274800	1.20966100	-0.05673000
H	-2.71682400	2.34128500	-0.05783800
H	-3.01611200	-2.64172200	-0.00457000
H	-0.66594400	2.79445200	0.75559200
H	-0.16723200	2.76196900	-0.94260100
H	1.56503400	2.31342900	1.53890100
H	1.76351400	3.53799500	0.29021800
H	2.28807300	1.77396800	-1.38999300
H	3.37830700	1.63624300	-0.01729400
H	2.22428500	-2.72969600	-0.48323400
H	2.10106800	-2.28037700	1.23335200
H	3.67916300	-2.66923000	0.51168000

DHPB_S_A_2.log

NImag=0

C	4.08950700	-0.75629900	-0.00071000
C	3.99967800	0.63381000	0.08301000
C	2.77531700	1.27592100	0.10883600
C	1.63736900	0.48404700	0.04762600
C	1.72158100	-0.90192100	-0.02870900
C	2.95326700	-1.54130300	-0.05654100
N	0.31162300	0.91239200	0.05540500
C	-0.57573900	-0.08259100	0.01127200
S	0.14439000	-1.63494600	-0.07561400
C	-0.05668500	2.32435300	0.07468700
C	-1.45274600	2.48495000	-0.47569400
C	-2.39500500	1.52104800	0.20646100
N	-1.91206200	0.14510700	0.04898900
C	-2.78496800	-0.96163100	0.02437600
O	-2.31434400	-2.06609900	-0.07455400
C	-4.24804500	-0.68856700	0.12659300
H	5.06392500	-1.22790400	-0.01958800
H	4.90601900	1.22419700	0.13120600
H	2.71990100	2.35404900	0.18457400
H	3.02129900	-2.62033400	-0.11837000
H	0.66879300	2.86048900	-0.53673600
H	0.02166200	2.69358700	1.10104600
H	-1.45384300	2.30540800	-1.55390700
H	-1.79511600	3.50685500	-0.31138200
H	-2.48671100	1.74979600	1.27294000
H	-3.38429600	1.58265600	-0.23856600
H	-4.61152600	-0.16931000	-0.76416500
H	-4.48936500	-0.07756200	0.99855800
H	-4.75434700	-1.64740200	0.20264200

BTM.log

NImag=0

C	2.8754880	0.5487070	0.1275270
C	2.1092460	-0.6023530	-0.1185540
C	4.1866370	0.4487410	0.5442130
C	4.7353140	-0.8168760	0.7289560
C	3.9745820	-1.9539050	0.4986920
C	2.6552370	-1.8610980	0.0715570
N	0.8503520	-0.2821870	-0.5557180

C	0.5328090	1.0597420	-0.5324990
S	1.9446960	2.0143560	-0.1534500
C	-0.3894100	-1.0206990	-0.6422770
C	-1.3747680	0.1277130	-1.0382440
N	-0.6748000	1.3863490	-0.7535080
C	-2.6993850	0.0028300	-0.3388390
C	-2.9800130	0.7397400	0.8043610
C	-4.1860750	0.5705060	1.4682210
C	-5.1221900	-0.3386070	0.9985350
C	-4.8499480	-1.0740130	-0.1461230
C	-3.6467140	-0.8996250	-0.8114780
H	4.7754530	1.3384530	0.7328080
H	5.7623640	-0.9098950	1.0593520
H	4.4125740	-2.9328740	0.6523870
H	2.0631290	-2.7496560	-0.1115040
H	-0.3441340	-1.8133180	-1.3903540
H	-0.6492270	-1.4599450	0.3277750
H	-1.5530540	0.0814900	-2.1200700
H	-2.2495790	1.4593960	1.1539640
H	-4.3975970	1.1564450	2.3555760
H	-6.0648980	-0.4683620	1.5174650
H	-5.5804060	-1.7789130	-0.5266410
H	-3.4434920	-1.4664810	-1.7152680

BTM_M_1.log

NImag=0

C	2.9803700	0.4463760	0.2357080
C	2.2553900	-0.6683560	-0.1963190
C	4.3258490	0.3518880	0.5380990
C	4.9308850	-0.8890280	0.4056850
C	4.2070110	-1.9992150	-0.0184220
C	2.8588110	-1.9059600	-0.3275760
N	0.9387830	-0.3258060	-0.4579140
C	0.6299070	0.9443960	-0.1972410
S	1.9629970	1.8808380	0.3175630
C	-0.2611840	-1.0906260	-0.7667650
C	-1.3259240	0.0219790	-0.9394300
N	-0.6483730	1.2084130	-0.3612320
C	-1.2682780	2.5088520	-0.3063770
C	-2.6445470	-0.2643400	-0.2869610
C	-2.7268280	-0.4498910	1.0907020
C	-3.9481260	-0.7172770	1.6840610
C	-5.0944390	-0.8073600	0.9043870
C	-5.0173770	-0.6233240	-0.4666520
C	-3.7942740	-0.3468120	-1.0606410
H	4.8901760	1.2140780	0.8705360
H	5.9830950	-0.9918680	0.6383800
H	4.7044810	-2.9564340	-0.1109700
H	2.2977780	-2.7707820	-0.6594290
H	-0.5048020	-1.7562480	0.0645880
H	-0.1330150	-1.6799720	-1.6736410
H	-1.4875580	0.2208560	-2.0044490
H	-0.6989460	3.1582860	0.3585060
H	-2.2766240	2.4007350	0.0919870
H	-1.3193280	2.9632820	-1.2999440
H	-1.8364810	-0.3760460	1.7078030
H	-4.0085580	-0.8571360	2.7565680
H	-6.0492530	-1.0198100	1.3698990
H	-5.9099190	-0.6912380	-1.0765400
H	-3.7353410	-0.1971650	-2.1338260

BTM_A.log

NImag=0

C	2.94563000	0.27340600	0.35487800
C	2.30984500	-0.76954100	-0.32763400
C	4.26392600	0.14883200	0.76280700
C	4.92147600	-1.03437800	0.47207100
C	4.28136500	-2.06912600	-0.20930500
C	2.96494100	-1.95386600	-0.62034700
N	1.00492400	-0.41583800	-0.62887000
C	0.65941300	0.79442800	-0.22178200
S	1.87687100	1.65003500	0.58359000
C	-0.07748800	-1.07351900	-1.34504300
C	-1.26789000	-0.08766300	-1.15034600
N	-0.60987700	1.09319300	-0.53712500
C	-1.15479000	2.34448300	-0.19483100
O	-0.43814600	3.11832000	0.38641100
C	-2.56514700	2.59092800	-0.59679300
C	-2.36771600	-0.64479600	-0.29169400
C	-2.18761900	-0.81405800	1.07796700
C	-3.19942700	-1.36251100	1.84810100
C	-4.39377100	-1.74908900	1.25463300
C	-4.57566700	-1.58300300	-0.10937400
C	-3.56505200	-1.02843500	-0.88094500
H	4.76554800	0.94984400	1.29142000
H	5.95223600	-1.15644600	0.77998400
H	4.82369000	-2.98175900	-0.42209900
H	2.46807900	-2.75731300	-1.14982700
H	-0.29467900	-2.04693000	-0.90570200
H	0.19416600	-1.20014800	-2.39453700
H	-1.67069600	0.20092300	-2.12190100
H	-2.81707300	3.62028700	-0.35592500
H	-3.23383900	1.91093600	-0.06382900
H	-2.70320900	2.41761600	-1.66681500
H	-1.26231000	-0.50762800	1.55599900
H	-3.05786200	-1.48632800	2.91488700
H	-5.18484300	-2.17584800	1.85910700
H	-5.50819700	-1.87843800	-0.57454900
H	-3.71329500	-0.88992900	-1.94716000

TM.log

NImag=0

C	-3.8900892	0.2434062	0.4875888
C	-2.7459446	1.1128262	0.9900621
N	-1.7180206	0.9711087	-0.0099953
C	-1.6145502	-0.3346967	-0.4712650
S	-3.0998893	-1.2210467	-0.2535222
C	-0.3403342	1.3853643	0.1896089
C	0.3911378	0.4415568	-0.7986571
N	-0.4939267	-0.7222703	-0.9236104
C	1.7854471	0.1042181	-0.3553944
C	2.0614366	-1.0870518	0.3036073
C	3.3452522	-1.3584154	0.7532362
C	4.3659826	-0.4410324	0.5529023
C	4.0981556	0.7487201	-0.1089184
C	2.8161912	1.0152501	-0.5632875
H	-4.4709899	0.7598214	-0.2775014
H	-4.5532770	-0.0741407	1.2907140
H	-2.3963262	0.7621677	1.9739959
H	-3.0612458	2.1543713	1.0865412
H	-0.1873972	2.4423333	-0.0308922
H	-0.0189422	1.1843015	1.2226681
H	0.4480345	0.9306179	-1.7809400
H	1.2606514	-1.8035820	0.4414701
H	3.5504398	-2.2951126	1.2593551
H	5.3690260	-0.6552385	0.9035688
H	4.8919950	1.4671613	-0.2793741
H	2.6137412	1.9409141	-1.0939018

TM_M_1.log

NImag=0

C	3.9340470	-0.5543630	0.2536290
C	2.8477290	-1.6224650	0.2375480
N	1.7147570	-0.9766340	-0.3959370

C	1.6395750	0.3332170	-0.1587130
S	3.0689610	1.0496360	0.4500420
C	0.3649360	-1.5034330	-0.5628390
C	-0.4434670	-0.2332170	-0.8966960
N	0.4498620	0.8326290	-0.3766080
C	0.0902240	2.2301330	-0.4042900
C	-1.8205400	-0.1805560	-0.3092360
C	-2.0052370	-0.2105000	1.0707200
C	-3.2820480	-0.1642930	1.6028160
C	-4.3838090	-0.0957720	0.7596440
C	-4.2052660	-0.0662550	-0.6140810
C	-2.9249420	-0.1024140	-1.1471020
H	4.4863200	-0.5189640	-0.6849270
H	4.6294970	-0.6746290	1.0817830
H	2.5827540	-1.9432400	1.2523030
H	3.1650940	-2.4940140	-0.3358680
H	0.0190200	-1.9574130	0.3720650
H	0.3160260	-2.2448240	-1.3584880
H	-0.5079670	-0.1028530	-1.9826120
H	0.0182490	2.5872160	-1.4348380
H	0.8424860	2.8125500	0.1260930
H	-0.8729040	2.3600270	0.0881870
H	-1.1496400	-0.2594380	1.7374170
H	-3.4207180	-0.1832790	2.6770120
H	-5.3828250	-0.0634560	1.1772700
H	-5.0625510	-0.0112230	-1.2737970
H	-2.7857650	-0.0730980	-2.2228640

TM_M_2.log

NImag=0

C	3.5207000	-0.9100850	0.6811450
C	2.7872670	-1.5255890	-0.5050860
N	1.5000710	-0.8623880	-0.5153250
C	1.5201690	0.3859610	-0.0549700
S	2.9357450	0.8230700	0.8024410
C	0.4017780	-1.0179950	-1.4593680
C	-0.4709610	0.2301380	-1.1690750
N	0.4645070	1.0804270	-0.3901900
C	0.1083620	2.4013380	0.0688890
C	-1.7382590	-0.0364730	-0.4079960
C	-1.7037690	-0.6668770	0.8337370
C	-2.8783750	-0.9060010	1.5252380
C	-4.0967290	-0.5194890	0.9807850
C	-4.1364810	0.1080000	-0.2535750
C	-2.9581330	0.3520690	-0.9447780
H	4.6006400	-0.8999390	0.5483890
H	3.2730990	-1.4094000	1.6173910
H	2.6663810	-2.6014400	-0.3729060
H	3.3175620	-1.3425870	-1.4472450
H	-0.1459180	-1.9428680	-1.2831370
H	0.7850360	-1.0164080	-2.4845390
H	-0.7133020	0.7533910	-2.0962310
H	-0.0371890	3.0642080	-0.7856390
H	0.9056590	2.7986450	0.6953700
H	-0.8147620	2.3543700	0.6492740
H	-0.7569200	-0.9743650	1.2672160
H	-2.8468810	-1.3962840	2.4907500
H	-5.0153960	-0.7090750	1.5225960
H	-5.0847280	0.4108920	-0.6805960
H	-2.9906650	0.8454840	-1.9108060

TM_A.log

NImag=0

C	3.87663200	-0.97925800	0.25274000
C	2.77247000	-2.01313200	0.06216200
N	1.65176800	-1.25622800	-0.46705300
C	1.64194700	0.01849000	-0.13339600
S	3.05642200	0.63682200	0.56684600
C	0.31432300	-1.71544700	-0.80694200
C	-0.47751900	-0.39250000	-0.96680600
N	0.46401500	0.60047900	-0.38012500
C	0.28937400	1.99368100	-0.18165400
O	1.18626500	2.59276900	0.34695900
C	-0.98989000	2.58722400	-0.65219400
C	-1.81946500	-0.42044200	-0.29785200
C	-1.91949900	-0.47460400	1.08944200
C	-3.16405400	-0.51875200	1.69432400
C	-4.31497900	-0.52105400	0.91679900
C	-4.21848200	-0.47519100	-0.46504300
C	-2.97197900	-0.41976500	-1.07133600
H	4.48643900	-0.86616500	-0.64360300
H	4.52226800	-1.21113200	1.09745100
H	2.48042900	-2.48157900	1.00896400
H	3.07181000	-2.79381100	-0.63757500
H	-0.09347200	-2.32190700	0.00730500
H	0.31687300	-2.30698000	-1.72187500
H	-0.59858000	-0.15567500	-2.02666200
H	-1.23111600	2.26735000	-1.66777300
H	-0.89605100	3.66951600	-0.61195000
H	-1.81023200	2.26667100	-0.00594600
H	-1.02596100	-0.46615700	1.70629400
H	-3.23813400	-0.55119800	2.77454100
H	-5.28802200	-0.55672200	1.39140300
H	-5.11430600	-0.47623600	-1.07390300
H	-2.89857000	-0.37254400	-2.15310800

SeHyperBTM_1.log

NImag=0

C	-3.7253357	1.6134174	1.9869257
C	-4.6917439	0.7709508	1.4596420
C	-4.3514035	-0.1490275	0.4743128
C	-3.0466185	-0.2065500	0.0272166
C	-2.0693065	0.6498740	0.5496924
C	-2.4106965	1.5596156	1.5416816
Se	-2.3077916	-1.3543391	-1.2736479
C	-0.6323529	-0.5041209	-0.9276201
N	-0.8074764	0.4927284	0.0089075
N	0.4380758	-0.8619165	-1.4960486
C	1.6546160	-0.1668353	-1.1119223
C	1.3603382	1.3035757	-0.7380711
C	0.3122062	1.3396226	0.3613839
C	2.5983429	2.1337705	-0.3844704
C	2.2383761	3.5089310	0.1669338
C	3.5076127	2.3042838	-1.5950159
C	2.3925164	-0.9186226	-0.0242536
C	1.7191149	-1.5401042	1.0233457
C	2.4165447	-2.1840734	2.0342049
C	3.8022057	-2.2284027	2.0107029
C	4.4842143	-1.6323098	0.9608764
C	3.7826609	-0.9876949	-0.0459662
H	-3.9918338	2.3234726	2.7606963
H	-5.7132238	0.8228839	1.8158875
H	-5.1018025	-0.8137860	0.0631095
H	-1.6625915	2.2131192	1.9722672
H	2.2986758	-0.1672499	-1.9941468
H	0.9042909	1.7490960	-1.6327696
H	-0.0649545	2.3555679	0.4840104
H	0.7433829	1.0218771	1.3196016
H	3.1521763	1.5943503	0.3926804
H	1.7229989	3.4540731	1.1276648
H	1.5987320	4.0583949	-0.5315928
H	3.1428909	4.1020437	0.3195619
H	3.8273669	1.3532097	-2.0216793
H	4.4065980	2.8626755	-1.3244343
H	2.9939078	2.8634455	-2.3833254

H	0.6360241	-1.5446961	1.0413778
H	1.8713808	-2.6637221	2.8392451
H	4.3465320	-2.7371924	2.7977775
H	5.5667023	-1.6759614	0.9198643
H	4.3257642	-0.5374173	-0.8697521

SeHyperBTM_2.log

NImag=0

C	-4.6002520	1.6948550	-0.3754350
C	-5.2187480	0.5274720	0.0443440
C	-4.4685950	-0.6262930	0.2412590
C	-3.1066040	-0.5906210	0.0193580
C	-2.4760050	0.5879460	-0.3988730
C	-3.2305060	1.7359430	-0.6024460
Se	-1.8583110	-1.9910050	0.1995240
C	-0.5284020	-0.7416260	-0.3590760
N	-1.1093060	0.4864780	-0.5770940
N	0.6904390	-1.0542810	-0.4686160
C	1.5899160	-0.0194840	-0.9344190
C	1.1749520	1.3973170	-0.4812580
C	-0.2744980	1.6306580	-0.8913620
C	1.4760800	1.7336220	0.9904490
C	0.5895000	1.0404640	2.0172730
C	1.4518890	3.2415210	1.2074880
C	3.0192810	-0.3272420	-0.5642780
C	3.3329050	-1.2627910	0.4119770
C	4.6546010	-1.4936510	0.7670700
C	5.6789690	-0.7946540	0.1478900
C	5.3740650	0.1343140	-0.8375420
C	4.0539860	0.3624750	-1.1896940
H	-5.1885110	2.5907570	-0.5340820
H	-6.2877070	0.5089140	0.2174980
H	-4.9475500	-1.5421790	0.5664780
H	-2.7632660	2.6509760	-0.9436050
H	1.5432150	-0.0149600	-2.0345470
H	1.7735640	2.0958180	-1.0747010
H	-0.3401600	1.8436060	-1.9640370
H	-0.6771280	2.4974310	-0.3623000
H	2.5041280	1.3996420	1.1655520
H	0.9029240	1.3248120	3.0246370
H	0.6477890	-0.0452800	1.9432170
H	-0.4592250	1.3338320	1.9117820
H	0.4482890	3.6574860	1.0729990
H	2.1249270	3.7572080	0.5173950
H	1.7641640	3.4867890	2.2251900
H	2.5262280	-1.8148370	0.8778100
H	4.8844030	-2.2279110	1.5311380
H	6.7109960	-0.9771310	0.4244620
H	6.1680220	0.6776580	-1.3374160
H	3.8243430	1.0848070	-1.9676720

SeHyperBTM_3.log

NImag=0

C	-3.7224959	1.8195512	1.8194163
C	-4.6823545	0.8869715	1.4578066
C	-4.3494153	-0.1606090	0.6065863
C	-3.0593228	-0.2537733	0.1242349
C	-2.0894349	0.6931300	0.4771906
C	-2.4226372	1.7306156	1.3379738
Se	-2.3320403	-1.5669184	-1.0165239
C	-0.6689430	-0.6386927	-0.8618046
N	-0.8438398	0.4875060	-0.0841507
N	0.3899141	-1.0588614	-1.4097570
C	1.6031795	-0.2860310	-1.2120001
C	1.2831733	1.2055855	-0.9919662
C	0.2853029	1.3645868	0.1404452
C	2.5077030	2.1254407	-0.8480465
C	3.0253652	2.2840760	0.5775604
C	2.2178748	3.4953228	-1.4500916
C	2.4670077	-0.8937050	-0.1327340
C	1.9244536	-1.3544510	1.0630484
C	2.7430274	-1.8399346	2.0712048
C	4.1181501	-1.8863566	1.8942997
C	4.6667544	-1.4595196	0.6942012
C	3.8445198	-0.9732437	-0.3101439
H	-3.9823584	2.6296162	2.4903833
H	-5.6922952	0.9676940	1.8405820
H	-5.0938131	-0.8966147	0.3268130
H	-1.6795380	2.4577332	1.6399656
H	2.1725493	-0.3508581	-2.1453386
H	0.7623635	1.5058944	-1.9094719
H	-0.0783723	2.3943586	0.1762999
H	0.7453378	1.1382012	1.1080998
H	3.3091473	1.6696367	-1.4416628
H	3.2062395	1.3268576	1.0668581
H	2.3236157	2.8558279	1.1919755
H	3.9672989	2.8373737	0.5697466
H	1.9425673	3.4187705	-2.5047028
H	3.0911372	4.1478605	-1.3754483
H	1.3954457	3.9915752	-0.9252644
H	0.8497845	-1.3523207	1.2028315
H	2.3028408	-2.1928118	2.9969661
H	4.7572136	-2.2690521	2.6815343
H	5.7381014	-1.5112957	0.5366834
H	4.2783033	-0.6477760	-1.2500701

SeHyperBTM_4.log

NImag=0

C	-3.8615310	1.8378350	1.7328800
C	-4.7903630	0.8854070	1.3431250
C	-4.4109160	-0.1543830	0.5017060
C	-3.1053970	-0.2208930	0.0580030
C	-2.1665450	0.7454420	0.4407160
C	-2.5463370	1.7761410	1.2901400
Se	-2.3173250	-1.5162700	-1.0625560
C	-0.6790550	-0.5536330	-0.8581770
N	-0.9004240	0.5661510	-0.0820710
N	0.4035310	-0.9496400	-1.3757600
C	1.6027870	-0.1641650	-1.1179520
C	1.2415090	1.3235330	-0.9317750
C	0.1934890	1.4827450	0.1542810
C	2.3803150	2.3424510	-0.7664520
C	3.3965000	2.2317780	-1.8947440
C	3.0781090	2.3514760	0.5896850
C	2.4003190	-0.7900570	0.0036290
C	1.8159900	-1.1211960	1.2233010
C	2.5758170	-1.6539520	2.2526680
C	3.9323170	-1.8842150	2.0751420
C	4.5173590	-1.5965170	0.8516270
C	3.7532140	-1.0599040	-0.1731230
H	-4.1582880	2.6422410	2.3952910
H	-5.8127260	0.9449260	1.6956280
H	-5.1316600	-0.9048410	0.1993190
H	-1.8275120	2.5189960	1.6121870
H	2.2165070	-0.2393830	-2.0193880
H	0.7456030	1.5879420	-1.8751930
H	-0.1967410	2.5043160	0.1348780
H	0.6184070	1.3079090	1.1483120
H	1.8922630	3.3210830	-0.8748490
H	4.0744550	3.0882520	-1.8831520
H	2.9092910	2.2020280	-2.8730750
H	4.0081410	1.3329160	-1.7946780
H	2.3834570	2.5298450	1.4128950
H	3.8174400	3.1561550	0.6124560
H	3.5989970	1.4149320	0.7897310

H	0.7505860	-0.9872810	1.3680900
H	2.1023570	-1.9017310	3.1959440
H	4.5250370	-2.3037860	2.8796270
H	5.5706330	-1.7966730	0.6908990
H	4.2155570	-0.8476160	-1.1312890

SeHyperBTM_6.log

NImag=0

C	-4.6477630	1.6552720	-0.4849900
C	-5.2574870	0.5100180	0.0035380
C	-4.4980470	-0.6229040	0.2717090
C	-3.1355440	-0.5886570	0.0515980
C	-2.5140750	0.5682180	-0.4355230
C	-3.2780180	1.6946030	-0.7109200
Se	-1.8768850	-1.9663620	0.3199670
C	-0.5552410	-0.7439420	-0.3136280
N	-1.1458490	0.4664790	-0.6014900
N	0.6654290	-1.0539870	-0.4227130
C	1.5570030	-0.0281970	-0.9305060
C	1.1106980	1.3758770	-0.4587940
C	-0.2993920	1.5760080	-0.9963880
C	1.2497640	1.5556220	1.0722170
C	0.1435670	2.3855640	1.7131850
C	2.5995270	2.1723740	1.4227880
C	2.9900570	-0.3413350	-0.5892570
C	3.3191630	-1.2036650	0.4470780
C	4.6476140	-1.4222190	0.7842070
C	5.6618040	-0.7893800	0.0822720
C	5.3405120	0.0568730	-0.9706310
C	4.0138820	0.2745360	-1.3028290
H	-5.2432890	2.5346110	-0.6993420
H	-6.3267750	0.4925480	0.1746790
H	-4.9700680	-1.5218260	0.6500780
H	-2.8175780	2.5914890	-1.1054360
H	1.4873630	-0.0286090	-2.0284280
H	1.7388930	2.1232880	-0.9545240
H	-0.2788500	1.6573940	-2.0887860
H	-0.7337650	2.4959170	-0.6058820
H	1.2060850	0.5568300	1.5202710
H	0.3200960	2.4612500	2.7885080
H	-0.8482710	1.9494150	1.5783530
H	0.1278980	3.4067270	1.3183300
H	2.7230690	2.2327390	2.5068570
H	2.6658580	3.1904350	1.0245420
H	3.4341070	1.5985340	1.0215190
H	2.5194990	-1.7062580	0.9768170
H	4.8911630	-2.0945470	1.5992790
H	6.6989750	-0.9617450	0.3457370
H	6.1265870	0.5446970	-1.5358480
H	3.7678920	0.9379910	-2.1269460

SeHyperBTM_M_1.log

NImag=0

C	-3.8868920	2.0350180	1.5704100
C	-4.8031880	1.0391310	1.2523780
C	-4.4101420	-0.0745430	0.5275280
C	-3.0886390	-0.1624190	0.1297010
C	-2.1691450	0.8380010	0.4362120
C	-2.5637680	1.9472990	1.1693000
Se	-2.2683480	-1.5595530	-0.8321570
C	-0.7006510	-0.5441690	-0.7066350
N	-0.8771090	0.6021340	-0.0442060
N	0.4550260	-0.9325200	-1.2037800
C	1.6762390	-0.1617710	-0.9406500
C	1.3082920	1.3270890	-0.8596750
C	0.2214820	1.5352880	0.1753400
C	2.5215890	2.2329190	-0.6135950
C	2.1082370	3.6745100	-0.3408490
C	3.4787930	2.1957220	-1.7978610
C	0.5870990	-2.2549020	-1.7850410
C	2.4171470	-0.7169990	0.2530230
C	3.7984220	-0.8665880	0.1901660
C	4.5092060	-1.3401520	1.2817470
C	3.8437770	-1.6815800	2.4489470
C	2.4643200	-1.5561520	2.5151010
C	1.7555200	-1.0789270	1.4236790
H	-4.2077170	2.8958650	2.1434100
H	-5.8325590	1.1288220	1.5754980
H	-5.1211140	-0.8540100	0.2835470
H	-1.8608480	2.7264030	1.4334220
H	2.2994380	-0.2991980	-1.8255270
H	0.8843960	1.5932940	-1.8369400
H	-0.1870640	2.5397400	0.0865050
H	0.6051800	1.4064950	1.1927100
H	3.0442750	1.8548270	0.2724310
H	2.9946800	4.3057670	-0.2641180
H	1.5572000	3.7882120	0.5947760
H	1.4938870	4.0727340	-1.1545140
H	4.3478840	2.8250130	-1.6006720
H	2.9925670	2.5782140	-2.7001790
H	3.8523360	1.1947770	-2.0181790
H	1.5185540	-2.2965560	-2.3455330
H	0.6068090	-3.0275890	-1.0120960
H	-0.2358110	-2.4450550	-2.4779900
H	4.3275050	-0.6138700	-0.7220690
H	5.5848990	-1.4504020	1.2159600
H	4.3969940	-2.0566130	3.3013460
H	1.9356760	-1.8370450	3.4181170
H	0.6750520	-1.0129410	1.4939920

SeHyperBTM_M_2.log

NImag=0

C	-3.8636450	2.1403730	1.4785380
C	-4.7822090	1.1149220	1.2852460
C	-4.4041770	-0.0598750	0.6552850
C	-3.0957160	-0.1783540	0.2234650
C	-2.1742960	0.8506820	0.4046370
C	-2.5536360	2.0215440	1.0441430
Se	-2.2973860	-1.6555500	-0.6314510
C	-0.7325560	-0.6280310	-0.6485460
N	-0.8973790	0.5763270	-0.0942240
N	0.4077140	-1.0635440	-1.1427810
C	1.6308900	-0.2548930	-1.0555250
C	1.2351180	1.2260330	-1.0635930
C	0.2156420	1.5108910	0.0184640
C	2.4331750	2.1935740	-1.0257960
C	2.9266130	2.5419130	0.3740600
C	2.1020270	3.4640290	-1.7990810
C	0.5153150	-2.4345030	-1.6063940
C	2.4994320	-0.7118310	0.0880220
C	3.8716530	-0.8224970	-0.1054040
C	4.7046420	-1.1766350	0.9441480
C	4.1698840	-1.4380430	2.1965350
C	2.7987440	-1.3568710	2.3913450
C	1.9674580	-0.9988350	1.3417600
H	-4.1721920	3.0494780	1.9791850
H	-5.8012260	1.2297550	1.6323200
H	-5.1165270	-0.8622270	0.5091110
H	-1.8485850	2.8249580	1.2121480
H	2.1721440	-0.4386720	-1.9875490
H	0.7287240	1.3788860	-2.0242790
H	-0.1813260	2.5187260	-0.0996200
H	0.6490620	1.4259790	1.0180590
H	3.2523080	1.6914300	-1.5532650
H	3.8504600	3.1179870	0.3012270

H	3.1345440	1.6627290	0.9842450
H	2.2057030	3.1691330	0.9061760
H	1.2655810	4.0028280	-1.3435860
H	1.8394410	3.2489840	-2.8373420
H	2.9560170	4.1433530	-1.8037800
H	0.4380210	-3.1396720	-0.7747730
H	-0.2620180	-2.6470750	-2.3448620
H	1.4851870	-2.5648140	-2.0806630
H	4.2950220	-0.6244110	-1.0846890
H	5.7727190	-1.2554160	0.7812130
H	4.8189670	-1.7182710	3.0171750
H	2.3742540	-1.5778960	3.3633080
H	0.8963320	-0.9581210	1.5107520

SeHyperBTM_M_3.log

NImag=0

C	-3.9902590	2.2074240	1.2942500
C	-4.8791930	1.1514480	1.1289900
C	-4.4602170	-0.0397380	0.5582590
C	-3.1407680	-0.1440950	0.1574350
C	-2.2491020	0.9149310	0.3108700
C	-2.6692890	2.1030100	0.8901210
Se	-2.2879620	-1.6359930	-0.6147980
C	-0.7494620	-0.5690240	-0.6523410
N	-0.9560150	0.6527010	-0.1517120
N	0.4108420	-0.9921240	-1.1095180
C	1.6231030	-0.1667240	-0.9756280
C	1.1997550	1.3039110	-1.0798970
C	0.1228470	1.6288040	-0.0673830
C	2.3238170	2.3541010	-1.0660150
C	3.3639500	2.0656750	-2.1394220
C	2.9849250	2.6071010	0.2841160
C	0.5683530	-2.3784130	-1.5069750
C	2.4118300	-0.5954530	0.2370410
C	1.8266200	-0.7001050	1.4963300
C	2.5851890	-1.0686800	2.5956380
C	3.9360300	-1.3485790	2.4483670
C	4.5196500	-1.2785790	1.1929930
C	3.7581910	-0.9114350	0.0944830
H	-4.3311590	3.1295180	1.7479550
H	-5.9074600	1.2554150	1.4513460
H	-5.1498850	-0.8652360	0.4338390
H	-1.9875690	2.9309690	1.0338360
H	2.2218780	-0.3890110	-1.8613000
H	0.7319060	1.3835910	-2.0696410
H	-0.2964570	2.6117280	-0.2855640
H	0.5098480	1.6410570	0.9542600
H	1.8201250	3.2858580	-1.3538100
H	3.9924680	1.2129940	-1.8735160
H	4.0270040	2.9234760	-2.2609840
H	2.9027000	1.8647940	-3.1102310
H	3.5348680	1.7383720	0.6457110
H	2.2701570	2.8997390	1.0559580
H	3.6961550	3.4292300	0.1842320
H	-0.2504390	-2.6758000	-2.1663470
H	1.5010730	-2.4778750	-2.0579100
H	0.6024740	-3.0391200	-0.6368650
H	0.7672430	-0.5127790	1.6340460
H	2.1185340	-1.1432350	3.5705540
H	4.5281320	-1.6357150	3.3087430
H	5.5688390	-1.5164760	1.0658170
H	4.2206540	-0.8652720	-0.8857820

SeHyperBTM_A_1.log

NImag=0

C	-3.93224000	2.54165500	1.01744300
C	-4.84974700	1.49447900	0.95857100
C	-4.45433400	0.23125500	0.55778000
C	-3.12573700	0.03309400	0.21228900
C	-2.21516800	1.08348200	0.26138200
C	-2.60708200	2.35191500	0.67157900
Se	-2.30119100	-1.56992000	-0.34487700
C	-0.74565800	-0.55447700	-0.44217300
N	-0.91963800	0.72394400	-0.12520000
N	0.46518300	-1.05324200	-0.77433700
C	1.65914800	-0.19103300	-0.70353900
C	1.24718900	1.22847100	-1.11599700
C	0.15383100	1.71057200	-0.19178300
C	2.42691900	2.20563900	-1.19084100
C	1.95858400	3.64116300	-1.39996500
C	3.38530000	1.81712400	-2.30945500
C	0.57032400	-2.42877600	-1.05085400
C	1.92624400	-2.97449900	-1.34246300
O	-0.43092900	-3.10459300	-1.04233100
C	2.33606800	-0.29956500	0.64214600
C	3.72551100	-0.24270200	0.70511500
C	4.38136500	-0.30723100	1.92384000
C	3.65494600	-0.43798900	3.09785300
C	2.27196500	-0.51296100	3.04440100
C	1.61631200	-0.44683600	1.82417100
H	-4.25835700	3.52193700	1.34156700
H	-5.88245600	1.66956200	1.23309200
H	-5.16737400	-0.58324600	0.51758900
H	-1.90420200	3.17207600	0.73440700
H	2.33948900	-0.56271900	-1.46628700
H	0.81793000	1.14849000	-2.12379300
H	0.53233200	1.89498000	0.81871400
H	-0.28544000	2.63025600	-0.57137700
H	2.96162600	2.15927400	-0.23565100
H	2.81993900	4.29421100	-1.54703700
H	1.40551000	4.03490300	-0.54504100
H	1.32753900	4.03490300	-2.28997200
H	2.88775000	1.87410900	-3.28205300
H	3.79157100	0.80975200	-2.20183300
H	4.23404500	2.50205900	-2.33431800
H	2.30310700	-2.58645600	-2.29260800
H	1.83598500	-4.05499900	-1.41714400
H	2.63997100	-2.71353500	-0.55884800
H	4.30447300	-0.15225800	-0.20747000
H	5.46337300	-0.26396500	1.95463800
H	4.16626700	-0.49432600	4.05103800
H	1.69807700	-0.63207200	3.95548300
H	0.53549300	-0.53368500	1.81111700

SeHyperBTM_A_2.log

NImag=0

C	-3.89336900	2.58943000	1.01609900
C	-4.81495300	1.54456900	1.04705100
C	-4.43878700	0.26118700	0.69503200
C	-3.12572100	0.04029300	0.30629600
C	-2.21180800	1.08789100	0.26503600
C	-2.58381600	2.37692400	0.62701600
Se	-2.32759100	-1.59255200	-0.19914800
C	-0.77438000	-0.59012900	-0.41495600
N	-0.93435500	0.70349600	-0.15581100
N	0.41825800	-1.11310000	-0.77927100
C	1.62297400	-0.25889300	-0.83532100
C	1.18703500	1.14873600	-1.25182100
C	0.14820500	1.67150300	-0.29054200
C	2.35450800	2.13256100	-1.46152400
C	2.79152700	2.87838400	-0.20548600
C	2.00646100	3.12240400	-2.56660600
C	0.49834400	-2.50069900	-1.00184100
C	1.82751800	-3.07054200	-1.36117300
O	-0.50335800	-3.16820000	-0.89915000

C	2.42675400	-0.35306100	0.43533100
C	1.82615900	-0.37467900	1.69015400
C	2.60254800	-0.40526300	2.83857800
C	3.98594200	-0.42138800	2.74426000
C	4.59140000	-0.42260700	1.49635800
C	3.81470600	-0.39474300	0.34945500
H	-4.20379900	3.58613500	1.30346100
H	-5.83526900	1.73768900	1.35390700
H	-5.15448400	-0.55142300	0.72521600
H	-1.87753800	3.19649800	0.62054700
H	2.22891000	-0.64619200	-1.65424600
H	0.69400500	1.02179700	-2.22333600
H	0.57022100	1.86440000	0.69865100
H	-0.28460000	2.59732500	-0.66759900
H	3.20508700	1.53588900	-1.81049800
H	3.70191500	3.44206000	-0.41519000
H	3.00164200	2.21548500	0.63417800
H	2.03684200	3.60513800	0.10836200
H	1.13691000	3.73018300	-2.29862400
H	1.78650000	2.61616600	-3.50919400
H	2.83630700	3.80910000	-2.74125100
H	2.59528500	-2.78419600	-0.64037000
H	2.14075800	-2.72515900	-2.35007900
H	1.72483000	-4.15224100	-1.38485100
H	0.74595300	-0.38519500	1.78889400
H	2.12393400	-0.42343000	3.81026500
H	4.59108700	-0.44742600	3.64220500
H	5.67119500	-0.45281900	1.41498500
H	4.29445700	-0.40146600	-0.62402300

SeHyperBTM_A_3.log

NImag=0

C	4.00808500	-2.67181100	0.62752500
C	4.90698900	-1.61530500	0.76117200
C	4.49763900	-0.30996000	0.55819000
C	3.17367200	-0.07792200	0.21556100
C	2.28250100	-1.13595600	0.07217900
C	2.68798500	-2.44824400	0.28267100
Se	2.33172900	1.58090800	-0.09907100
C	0.79628100	0.57216900	-0.39655300
N	0.99014000	-0.73831700	-0.28550300
N	-0.41553200	1.10225100	-0.67685100
C	-1.60938000	0.22859000	-0.75852400
C	-1.15360600	-1.10022000	-1.37230200
C	-0.06736700	-1.71359300	-0.52404700
C	-2.24630400	-2.12373700	-1.72562000
C	-3.31579600	-1.50857900	-2.61706600
C	-2.86597900	-2.87086900	-0.55031600
C	-0.52576200	2.50267200	-0.76045400
C	-1.87224700	3.08040900	-1.03177300
O	0.46638700	3.17679600	-0.61536900
C	-2.32596400	0.17626600	0.56812800
C	-1.65453400	-0.04583400	1.76713300
C	-2.35327300	-0.11475000	2.96245700
C	-3.73024500	0.04755300	2.97652400
C	-4.40445100	0.29760900	1.79096000
C	-3.70389700	0.36728000	0.59768900
H	4.34502200	-3.68661600	0.79785100
H	5.93617100	-1.81720800	1.03049100
H	5.19633300	0.51061900	0.66745200
H	1.99932200	-3.27798400	0.19324300
H	-2.27098900	0.69733600	-1.48458000
H	-0.69193000	-0.81954900	-2.32804800
H	-0.44713000	-2.06123500	0.43964800
H	0.37754500	-2.56176100	-1.04511400
H	-1.71970000	-2.87036000	-2.33359600
H	-3.95046800	-2.29017200	-3.03699400
H	-2.87976700	-0.95447300	-3.45296400
H	-3.97009100	-0.83406200	-2.06057700
H	-3.42061500	-2.21156500	0.11700300
H	-2.12370000	-3.40689900	0.04475800
H	-3.56452600	-3.61843000	-0.93075300
H	-2.22246300	2.80008100	-2.02860900
H	-1.78018000	4.16219000	-0.98258300
H	-2.60723300	2.73679600	-0.30172300
H	-0.57523500	-0.14659600	1.79138400
H	-1.81734300	-0.28824700	3.88781000
H	-4.27428800	-0.00516900	3.91161400
H	-5.47749800	0.44621000	1.79472600
H	-4.23946300	0.56971800	-0.32379000

SeHBTM_1.log

NImag=0

C	-2.9111900	-0.4602510	0.0442130
C	-2.3348130	0.8160360	0.0524810
C	-4.2735090	-0.6237760	0.1963440
C	-5.0783050	0.4983660	0.3564440
C	-4.5131750	1.7641930	0.3565180
C	-3.1433360	1.9349210	0.2027250
N	-0.9615440	0.8361020	-0.1025770
C	-0.3212540	-0.3706800	-0.2926480
Se	-1.5971490	-1.7891100	-0.2014900
C	-0.1743570	2.0511850	-0.0373670
C	1.2612330	1.7096670	0.3073370
C	1.7752410	0.5764070	-0.5805810
N	0.9072000	-0.5811870	-0.5037470
C	3.1967410	0.2236890	-0.2207330
C	3.4928400	-0.9041070	0.5324400
C	4.8028360	-1.1812350	0.8969350
C	5.8313550	-0.3358140	0.5105290
C	5.5434850	0.7896130	-0.2490980
C	4.2346890	1.0642610	-0.6108700
H	-4.7108580	-1.6151000	0.1907130
H	-6.1479700	0.3790920	0.4771680
H	-5.1439050	2.6372440	0.4740530
H	-2.7173490	2.9302170	0.1916820
H	-0.6021810	2.6994440	0.7312080
H	-0.2336990	2.5844920	-0.9935780
H	1.3268520	1.3848500	1.3500250
H	1.8846620	2.5990940	0.2017510
H	1.7780070	0.9311470	-1.6224570
H	2.6847140	-1.5689100	0.8118440
H	5.0210470	-2.0673050	1.4825890
H	6.8546580	-0.5556750	0.7923940
H	6.3418890	1.4510830	-0.5660330
H	4.0167240	1.9408110	-1.2144660

SeHBTM_2.log

NImag=0

C	-2.6208240	-0.1128780	0.2912090
C	-1.7872240	0.9007050	-0.1977270
C	-3.8843250	0.1756960	0.7668000
C	-4.3269320	1.4934310	0.7617820
C	-3.5008080	2.5015840	0.2894370
C	-2.2285240	2.2172990	-0.1895320
N	-0.5539810	0.4725090	-0.6512880
C	-0.2547530	-0.8674610	-0.5219860
Se	-1.7652620	-1.7899660	0.1965040
C	0.4339290	1.3607240	-1.2306600
C	1.3776710	0.5535800	-2.0976570
C	1.9100090	-0.6541250	-1.3213270
N	0.8257670	-1.4633550	-0.8025230
C	2.8713930	-0.2160860	-0.2355730
C	2.5223120	-0.2214200	1.1084620
C	3.4122300	0.2373850	2.0707760

C	4.6641370	0.7019080	1.7019830
C	5.0262390	0.6998900	0.3614620
C	4.1360780	0.2435190	-0.5957200
H	-4.5232160	-0.6152850	1.1412750
H	-5.3168030	1.7288700	1.1328930
H	-3.8448860	3.5289400	0.2954260
H	-1.5887790	3.0166640	-0.5413080
H	0.9826330	1.8785320	-0.4356480
H	-0.0842220	2.1121760	-1.8301830
H	2.1962520	1.1919320	-2.4342640
H	0.8442750	0.1937820	-2.9821590
H	2.4731810	-1.2852760	-2.0165470
H	1.5535190	-0.6045940	1.4045070
H	3.1241260	0.2247280	3.1158940
H	5.3594470	1.0561470	2.4541510
H	6.0079820	1.0496390	0.0626140
H	4.4292440	0.2359900	-1.6414850

SeHBTM_M_1.log

NImag=0

C	-2.7319660	0.0514550	-0.3010560
C	-1.9520830	-0.9764900	0.2234950
C	-4.0264280	-0.1727860	-0.7328940
C	-4.5358150	-1.4576540	-0.6350170
C	-3.7578990	-2.4892620	-0.1221090
C	-2.4611480	-2.2636240	0.3099360
N	-0.6686480	-0.5831800	0.6171480
C	-0.3582200	0.6994630	0.4035020
Se	-1.7782460	1.6763390	-0.3277620
C	0.2991630	-1.5040160	1.2030300
C	1.2720800	-0.7208710	2.0525660
C	1.8937720	0.4180370	1.2548680
N	0.8210600	1.2239330	0.6671250
C	1.1076410	2.6100880	0.3508030
C	2.8781000	-0.0536390	0.2107790
C	2.5049910	-0.2839380	-1.1079800
C	3.4278920	-0.7731770	-2.0215220
C	4.7292360	-1.0343290	-1.6235290
C	5.1093580	-0.7987610	-0.3096960
C	4.1889870	-0.3081150	0.6012990
H	-4.6286490	0.6309260	-1.1377750
H	-5.5472220	-1.6567420	-0.9658520
H	-4.1670070	-3.4897950	-0.0601360
H	-1.8649780	-3.0813830	0.6930280
H	0.8105470	-2.0443370	0.4013860
H	-0.2476430	-2.2216620	1.8131190
H	2.0619000	-1.3834530	2.4051800
H	0.7563010	-0.3160960	2.9274620
H	2.4145360	1.0768210	1.9550590
H	2.1846950	2.7205570	0.2374350
H	0.7550340	3.2767240	1.1419600
H	0.6431510	2.8885960	-0.5977510
H	1.4949270	-0.0706400	-1.4414430
H	3.1283830	-0.9452770	-3.0483590
H	5.4502640	-1.4124600	-2.3379470
H	6.1283730	-0.9898290	0.0043840
H	4.4949160	-0.1170310	1.6253770

SeHBTM_M_2.log

NImag=0

C	-2.9563880	-0.4163000	0.1421960
C	-2.4286440	0.8668950	0.0174880
C	-4.3036850	-0.6229270	0.3766510
C	-5.1276000	0.4857750	0.4832950
C	-4.6074410	1.7681230	0.3503430
C	-3.2580530	1.9744200	0.1150140
N	-1.0504320	0.9019050	-0.2176490
C	-0.4398710	-0.2834060	-0.3110720
Se	-1.6141500	-1.7210400	-0.0673750
C	-0.3036410	2.1407200	-0.3967430
C	1.1339410	1.9150000	0.0073530
C	1.7499960	0.7184730	-0.6991110
N	0.8447180	-0.4432970	-0.5586160
C	1.3550300	-1.7854580	-0.7893280
C	3.1190350	0.4087320	-0.1568260
C	3.2835990	-0.0197690	1.1574060
C	4.5501790	-0.2745420	1.6540490
C	5.6640290	-0.0949050	0.8439870
C	5.5061040	0.3343580	-0.4637500
C	4.2352770	0.5804870	-0.9641440
H	-4.7077030	-1.6228710	0.4738700
H	-6.1855100	0.3482810	0.6675300
H	-5.2652620	2.6245680	0.4281580
H	-2.8738740	2.9796000	0.0013860
H	-0.7570220	2.9014710	0.2375500
H	-0.3942750	2.4654610	-1.4375280
H	1.1923300	1.7623380	1.0878630
H	1.7269440	2.7995240	-0.2249110
H	1.8393280	0.9319290	-1.7706650
H	2.3723260	-1.7142720	-1.1640880
H	0.7384470	-2.2981410	-1.5319540
H	1.3738580	-2.3632870	0.1382750
H	2.4187040	-0.1642860	1.7977280
H	4.6712520	-0.6113460	2.6765740
H	6.6549750	-0.2919750	1.2346430
H	6.3718620	0.4743410	-1.0995650
H	4.1135170	0.9097050	-1.9911260

SeHBTM_A_1.log

NImag=0

C	-2.79206000	-0.05763700	-0.29830700
C	-2.06466300	-1.07560800	0.30938200
C	-4.09326800	-0.27522200	-0.72672800
C	-4.64655500	-1.52894700	-0.54161800
C	-3.91068400	-2.54992700	0.05690700
C	-2.61437100	-2.33920500	0.48863900
N	-0.77374200	-0.69379700	0.69058900
C	-0.42643800	0.55356700	0.38860300
Se	-1.77836500	1.52647700	-0.43971500
C	0.12377400	-1.61844500	1.37780000
C	1.15960700	-0.83299100	2.13838100
C	1.84059500	0.16905100	1.21835300
N	0.80758800	1.04540700	0.64804200
C	1.10616300	2.36082200	0.24487800
O	0.23609700	3.02702600	-0.26262000
C	2.49371700	2.85487500	0.47308500
C	2.68312100	-0.49230400	0.15254000
C	2.25606900	-0.62876400	-1.16243100
C	3.05270800	-1.28337000	-2.09220900
C	4.28024300	-1.80235900	-1.71514900
C	4.71529800	-1.66138300	-0.40436800
C	3.92144000	-1.00892700	0.52333800
H	-4.66411700	0.51596900	-1.19742600
H	-5.66081000	-1.71956100	-0.86939400
H	-4.35755500	-3.52772400	0.18552900
H	-2.05288600	-3.14683700	0.93925300
H	0.58321700	-2.27748400	0.63606600
H	-0.47891700	-2.22094800	2.05572500
H	1.90805600	-1.51438400	2.54159700
H	0.69531200	-0.30758500	2.97720000
H	2.47780000	0.80451800	1.83287300
H	2.68651200	2.98214000	1.54181000
H	2.58160700	3.82245000	-0.01410400
H	3.23844700	2.16622300	0.06992200
H	1.30618900	-0.21634300	-1.48471100

H	2.71173700	-1.38096000	-3.11580900
H	4.90295000	-2.30862600	-2.44252700
H	5.67969200	-2.05422600	-0.10583700
H	4.27332100	-0.89366900	1.54413200

SeHBTM_A_2.log

NImag=0

C	3.02712400	0.28176000	0.29849200
C	2.54982800	-0.96770500	-0.08450300
C	4.35945400	0.45736700	0.64237800
C	5.20145900	-0.63837700	0.59765300
C	4.72247400	-1.88668600	0.20499800
C	3.39679700	-2.06809700	-0.14241600
N	1.18780100	-0.97028800	-0.40305700
C	0.56286400	0.19985700	-0.31581600
Se	1.67033600	1.58965400	0.23517000
C	0.48010500	-2.16882900	-0.83446100
C	-0.93532200	-2.08289900	-0.32941500
C	-1.64082100	-0.82426900	-0.80891200
N	-0.74541300	0.34876600	-0.63383500
C	-1.22804800	1.67084800	-0.78817300
O	-0.54109700	2.58496400	-0.40055400
C	-2.53452500	1.88440000	-1.47210200
C	-2.93328200	-0.66164100	-0.05018600
C	-2.93748800	-0.19937900	1.26126300
C	-4.12974300	-0.10196800	1.96021500
C	-5.32215500	-0.47906800	1.35814600
C	-5.31896100	-0.95282800	0.05506200
C	-4.12708200	-1.04015100	-0.64836800
H	4.73369800	1.42932900	0.94015200
H	6.24411800	-0.52347800	0.86616500
H	5.39829200	-2.73169400	0.16568600
H	3.04965900	-3.04207300	-0.46076300
H	0.98604600	-3.03107200	-0.40567800
H	0.53418400	-2.24471800	-1.92455500
H	-0.93220100	-2.10097000	0.76347700
H	-1.51418200	-2.94413600	-0.66291700
H	-1.85333400	-0.91779100	-1.87807500
H	-3.34465100	1.86174000	-0.74091000
H	-2.74782200	1.13588000	-2.23326600
H	-2.49869500	2.87675300	-1.91803400
H	-2.01113700	0.10060400	1.74139100
H	-4.12809100	0.26798300	2.97837300
H	-6.25374200	-0.40192800	1.90534500
H	-6.24704200	-1.24799300	-0.41924700
H	-4.13054700	-1.39775200	-1.67339400

SeDHPB.log

NImag=0

C	-3.5013091	0.6796006	0.0192064
C	-2.8665136	1.9096290	-0.0526038
C	-1.4805499	1.9976818	-0.0857844
C	-0.7261039	0.8324927	-0.0395706
C	-1.3726301	-0.4087386	0.0222211
C	-2.7502519	-0.4899272	0.0535424
N	0.6539425	0.7707771	-0.0572159
C	1.2396943	-0.4814881	-0.0687035
Se	-0.1215679	-1.8184524	0.0416985
C	1.4991345	1.9479583	-0.0737934
C	2.8748961	1.5909383	0.4559892
C	3.3963250	0.3372273	-0.2250175
N	2.4669175	-0.7715312	-0.1422470
H	-4.5826817	0.6245517	0.0440409
H	-3.4538885	2.8194067	-0.0880539
H	-0.9999119	2.9652148	-0.1563342
H	-3.2412165	-1.4546143	0.1031363
H	1.0366694	2.7140795	0.5530240
H	1.5628692	2.3465077	-1.0936085
H	2.8091433	1.4120663	1.5333702
H	3.5530034	2.4333230	0.3028263
H	4.3416118	0.0256310	0.2258137
H	3.6125120	0.5436288	-1.2821541

DHPB_SeM_1.log

NImag=0

C	-0.7553487	3.9316687	2.3806240
C	-0.5139241	2.5683637	2.4944302
C	0.1787428	1.8632986	1.5208609
C	0.6564465	2.5428336	0.4084225
C	0.4090136	3.9173165	0.3121386
C	-0.2988549	4.6203496	1.2678044
N	1.3549749	1.9708207	-0.6412217
C	1.6717301	2.7648179	-1.7082836
Se	1.0906446	4.5978183	-1.3158769
C	1.7133853	0.5579941	-0.7375128
C	2.9132047	0.4115278	-1.6585947
C	2.7020516	1.1406019	-2.9752550
N	2.2454203	2.5135937	-2.7854700
C	2.8389382	5.2647828	-0.7908655
H	-1.3064352	4.4556006	3.1504279
H	-0.8835665	2.0330961	3.3606533
H	0.3316864	0.7973451	1.6268369
H	-0.4927546	5.6801134	1.1547693
H	0.8527013	-0.0096627	-1.1048039
H	1.9570117	0.1994118	0.2632729
H	3.0994003	-0.6483930	-1.8375612
H	3.7989200	0.8138212	-1.1587411
H	1.9558963	0.6355144	-3.5978306
H	3.6226339	1.1720706	-3.5589095
H	3.4427927	5.2541988	-1.6980893
H	3.2449503	4.6100135	-0.0240305
H	2.7012384	6.2802821	-0.4241946

DHPB_SeM_2.log

NImag=0

C	-0.7172399	3.9177759	2.4019161
C	-0.3598869	2.5875955	2.5809993
C	0.3587424	1.8871258	1.6227184
C	0.7333628	2.5359210	0.4541542
C	0.3818869	3.8818414	0.2983929
C	-0.3476583	4.5794976	1.2410592
N	1.4351617	1.9589420	-0.5910374
C	1.7706131	2.7524472	-1.6511192
Se	0.9813643	4.5290584	-1.3760578
C	1.9012430	0.5737637	-0.6120324
C	2.1940938	0.1614178	-2.0447245
C	3.0413125	1.1967941	-2.7665259
N	2.4658720	2.5345181	-2.6620854
C	2.6438724	5.4251147	-0.9219539
H	-1.2842922	4.4388415	3.1620247
H	-0.6500513	2.0764347	3.4909331
H	0.6252038	0.8519042	1.7907927
H	-0.6235950	5.6150170	1.0829223
H	1.1174297	-0.0566241	-0.1888079
H	2.7900513	0.4852915	0.0207144
H	1.2510976	0.0329745	-2.5829988
H	2.6970426	-0.8066220	-2.0399344
H	3.1370905	0.9564765	-3.8254537
H	4.0573702	1.2365092	-2.3588522
H	3.2537637	5.3773145	-1.8242377
H	3.1069074	4.8998777	-0.0905413
H	2.4001419	6.4545917	-0.6666646

SeDHPB_M.log

NImag=0

C	-3.7960834	0.1121937	0.0063765
C	-3.4041708	1.4438308	-0.0708159
C	-2.0651087	1.7975497	-0.0955843
C	-1.1159268	0.7878079	-0.0390573
C	-1.5139000	-0.5450576	0.0280981
C	-2.8505143	-0.8992397	0.0547621
N	0.2711854	0.9756456	-0.0518535
C	1.0111103	-0.1366486	-0.0152473
Se	-0.0220358	-1.6943948	0.0537617
C	0.8808063	2.3018864	-0.1042377
C	2.2882465	2.2403499	0.4469690
C	3.0652087	1.1093041	-0.1879416
N	2.3308935	-0.1438319	-0.0371809
C	3.0521276	-1.4010392	-0.0970757
H	-4.8485429	-0.1408273	0.0248357
H	-4.1553431	2.2223081	-0.1150124
H	-1.7791545	2.8387497	-0.1667799
H	-3.1539117	-1.9372540	0.1094104
H	0.2679056	2.9733803	0.4963833
H	0.8635381	2.6573114	-1.1384629
H	2.2550351	2.0981947	1.5300233
H	2.7933254	3.1872449	0.2552885
H	4.0316941	0.9845726	0.3005657
H	3.2495634	1.2977791	-1.2515971
H	4.0906134	-1.2224289	0.1734679
H	3.0157781	-1.8336774	-1.1007903
H	2.6345565	-2.1107103	0.6211573

DHPB_Se_A_1.log

NImag=0

C	-4.14394000	-0.41748100	-0.02918200
C	-3.98310200	0.96479000	-0.10549300
C	-2.72582100	1.53937800	-0.11711000
C	-1.62323600	0.69617000	-0.04942700
C	-1.77994000	-0.68429300	0.01625700
C	-3.04462600	-1.25403700	0.03026400
N	-0.28616400	1.11080200	-0.04735600
C	0.61993600	0.13763700	-0.01657600
Se	-0.11505800	-1.56748500	0.06650600
C	0.06631200	2.52963000	-0.04846600
C	1.46174100	2.70824200	0.49603600
C	2.41754000	1.77125600	-0.20229100
N	1.95093700	0.38820100	-0.05939700
C	2.84044200	-0.70223400	-0.05013200
O	2.39257500	-1.81947300	0.04180900
C	4.29854600	-0.40895600	-0.15993600
H	-5.13998800	-0.84205600	-0.02032800
H	-4.85580300	1.60335500	-0.15953200
H	-2.62016800	2.61376200	-0.18974800
H	-3.17085600	-2.32852000	0.08421600
H	-0.66046700	3.04751200	0.57623400
H	-0.02528900	2.91238000	-1.06880700
H	1.47224300	2.51492700	1.57184100
H	1.78313300	3.73879600	0.34356800
H	3.40762700	1.84187000	0.23953500
H	2.50053600	2.01371200	-1.26636200
H	4.52485700	0.21636900	-1.02570200
H	4.81745300	-1.35962100	-0.25159400
H	4.66155300	0.10414800	0.73460700

DHPB_Se_A_2.log

NImag=0

C	-4.14288300	-0.42519500	-0.04549900
C	-3.97565900	0.95676100	-0.06431100
C	-2.71547600	1.52775100	-0.05092300
C	-1.61300900	0.68328100	-0.01987200
C	-1.78590900	-0.69507400	-0.01281600
C	-3.04615300	-1.26869000	-0.01932200
N	-0.27587200	1.09749500	0.00348800
C	0.65132400	0.13582600	-0.00702900
Se	-0.13082100	-1.57015200	0.00471100
C	0.03281900	2.52907600	0.07072100
C	1.46717300	2.73226500	0.48106900
C	2.35498100	1.81718900	-0.31955900
N	1.96487900	0.41711900	-0.08126500
C	3.06039700	-0.51552500	-0.05831500
O	4.14360100	-0.09363100	-0.33754800
C	2.82318800	-1.93549400	0.33492300
H	-5.13986400	-0.84726300	-0.05527100
H	-4.84537000	1.60101600	-0.09117600
H	-2.60703400	2.60369300	-0.07423700
H	-3.17385400	-2.34387300	-0.00921700
H	-0.65171400	2.97219400	0.79408800
H	-0.17331700	2.96596600	-0.91010700
H	1.59321500	2.53358400	1.54841000
H	1.75074200	3.77027800	0.30582800
H	3.39972100	1.90092500	-0.03615000
H	2.28643700	2.02202500	-1.39200800
H	2.21936400	-2.02987000	1.23894300
H	3.80218200	-2.37684500	0.51000700
H	2.35079200	-2.49293600	-0.47885900

SeBTM.log

NImag=0

C	-2.6966330	-0.0785330	0.1759500
C	-1.7949990	0.9543130	-0.1237800
C	-3.9822040	0.2033050	0.5877160
C	-4.3756790	1.5321640	0.7128290
C	-3.4829190	2.5547330	0.4281050
C	-2.1884980	2.2783850	0.0077370
N	-0.5682150	0.5092630	-0.5442870
C	-0.3163250	-0.8447030	-0.5011320
Se	-1.8919610	-1.7863320	-0.0526940
C	0.7009630	1.1862810	-0.6986940
C	1.6310420	-0.0191280	-1.0534480
N	0.8673430	-1.2338200	-0.7385880
C	2.9541640	0.0580430	-0.3437830
C	3.1804860	-0.6463140	0.8315820
C	4.3873090	-0.5188550	1.5032590
C	5.3787660	0.3155700	1.0090770
C	5.1608150	1.0178300	-0.1675200
C	3.9563590	0.8851540	-0.8403370
H	-4.6757300	-0.5967140	0.8183430
H	-5.3823610	1.7637860	1.0379660
H	-3.7955420	3.5867860	0.5333990
H	-1.4934260	3.0787290	-0.2165260
H	1.0011030	1.6681420	0.2386320
H	0.6678770	1.9389060	-1.4882010
H	1.8211290	-0.0165620	-2.1336610
H	2.4069140	-1.3085710	1.2013800
H	4.5557270	-1.0789360	2.4161630
H	6.3219750	0.4126570	1.5341890
H	5.9343000	1.6639290	-0.5669190
H	3.7949360	1.4255570	-1.7684020

SeBTM_M.log

NImag=0

C	-2.8123710	-0.0299710	0.1782220
C	-1.9556930	1.0029860	-0.2091170
C	-4.1394400	0.2159470	0.4728700
C	-4.5978750	1.5220910	0.3789710

C	-3.7445450	2.5518690	-0.0012580
C	-2.4140300	2.3064670	-0.3012310
N	-0.6676630	0.5649100	-0.4791710
C	-0.4133420	-0.7281370	-0.2877010
Se	-1.9071750	-1.7022000	0.2251930
C	0.5616550	1.2915120	-0.7704020
C	1.5851990	0.1490960	-0.9822910
N	0.8491960	-1.0362280	-0.4788180
C	1.4188350	-2.3599470	-0.4791760
C	2.8999740	0.3457750	-0.2893090
C	4.0712080	0.4039640	-1.0322210
C	5.2910350	0.5952050	-0.3990590
C	5.3426720	0.7180410	0.9799860
C	4.1742400	0.6519480	1.7286610
C	2.9567730	0.4694360	1.0965630
H	-4.8070560	-0.5825010	0.7718260
H	-5.6339600	1.7378010	0.6070630
H	-4.1229560	3.5642340	-0.0660620
H	-1.7513790	3.1101440	-0.5983390
H	0.8322630	1.9231350	0.0788370
H	0.4501540	1.9128810	-1.6581900
H	1.7618970	-0.0028590	-2.0523300
H	2.4274680	-2.3097870	-0.0698770
H	1.4604330	-2.7699770	-1.4922170
H	0.8201390	-3.0168140	0.1518770
H	4.0318320	0.3027070	-2.1119410
H	6.2007100	0.6449220	-0.9849280
H	6.2946610	0.8641100	1.4758550
H	4.2145170	0.7442230	2.8072510
H	2.0487360	0.4141170	1.6894760

SeBTM_A.log

NImag=0

C	2.81201600	-0.09133800	0.25126000
C	2.04210900	-1.08622700	-0.35978900
C	4.12176400	-0.34873200	0.61980600
C	4.64026800	-1.60826300	0.36716900
C	3.86767600	-2.59417700	-0.24257700
C	2.55820600	-2.34724200	-0.61456400
N	0.75785800	-0.65063300	-0.65753200
C	0.47031100	0.59610700	-0.33400900
Se	1.83300700	1.52802600	0.46325400
C	-0.36403100	-1.30507900	-1.31777900
C	-1.50751200	-0.25770200	-1.18032300
N	-0.78641100	0.92929000	-0.65725800
C	-1.26690500	2.22327300	-0.39198300
O	-0.50745700	3.00200600	0.12882900
C	-2.66980600	2.51150700	-0.79087400
C	-2.61810300	-0.70226600	-0.27140400
C	-2.42197300	-0.78983800	1.10375300
C	-3.44553200	-1.23490000	1.92341400
C	-4.66793600	-1.59914200	1.37456900
C	-4.86584400	-1.51435900	0.00533300
C	-3.84315700	-1.06330900	-0.81621500
H	4.73007100	0.41196600	1.09347600
H	5.66263900	-1.82727400	0.64855700
H	4.29678300	-3.57042400	-0.42977000
H	1.95733200	-3.11336700	-1.08922200
H	-0.61785400	-2.23625100	-0.81146200
H	-0.11045800	-1.51449500	-2.35859400
H	-1.91099800	-0.01826200	-2.16484300
H	-3.36116100	1.90251200	-0.20363600
H	-2.83385700	2.27240800	-1.84440400
H	-2.86842400	3.56584100	-0.61783900
H	-1.47386300	-0.50016900	1.54631900
H	-3.29100700	-1.29555300	2.99386100
H	-5.46803000	-1.94503000	2.01762800
H	-5.81987200	-1.79283000	-0.42545200
H	-4.00330200	-0.98847900	-1.88706600

SeTM_1.log

NImag=0

C	-3.4122460	0.8334000	0.5598530
C	-2.1369610	1.5465950	0.9765770
N	-1.1497580	1.2370240	-0.0274440
C	-1.1459610	-0.0920520	-0.4250170
Se	-2.8258010	-0.9065460	-0.1361530
C	0.2580580	1.5638070	0.1302620
C	0.9082730	0.5179390	-0.8090470
N	-0.0636880	-0.5818600	-0.8669040
C	2.2772120	0.0993220	-0.3567680
C	2.4655110	-1.0627040	0.3807980
C	3.7294910	-1.4067700	0.8365460
C	4.8180470	-0.5916360	0.5638150
C	4.6377500	0.5679910	-0.1763250
C	3.3749880	0.9067950	-0.6364950
H	-3.9272540	1.3659650	-0.2384960
H	-4.0902420	0.6761300	1.3961300
H	-1.8117680	1.2014230	1.9709700
H	-2.3016900	2.6262030	1.0278350
H	0.5802540	1.4010090	1.1697510
H	0.4789510	2.5936520	-0.1525320
H	0.9905390	0.9462040	-1.8173080
H	1.6117640	-1.7006450	0.5754790
H	3.8656280	-2.3203160	1.4044660
H	5.8054900	-0.8626960	0.9193710
H	5.4845340	1.2058240	-0.4032620
H	3.2401800	1.8075400	-1.2279470

SeTM_2.log

NImag=0

C	-2.9460860	0.3421470	1.2925000
C	-2.0283730	1.5286180	1.0468460
N	-0.8237470	0.9957700	0.4664820
C	-1.0273170	0.0152300	-0.4888360
Se	-2.7011300	-0.8313960	-0.2630360
C	0.2344330	1.8246760	-0.0796840
C	0.9142350	0.8240920	-1.0559060
N	-0.1326920	-0.1578620	-1.3706200
C	2.1415170	0.1851340	-0.4489080
C	2.0976550	-1.0737850	0.1347620
C	3.2301870	-1.6130820	0.7281590
C	4.4199320	-0.9017530	0.7429040
C	4.4736310	0.3543370	0.1549530
C	3.3416410	0.8900370	-0.4373940
H	-3.9918870	0.6343670	1.3612970
H	-2.6564640	-0.2091450	2.1859940
H	-1.8042790	2.0391260	1.9873520
H	-2.5099970	2.2525740	0.3699510
H	0.9115940	2.1907610	0.6930000
H	-0.1913170	2.6811570	-0.6230450
H	1.2152480	1.3350310	-1.9739010
H	1.1719020	-1.6352660	0.1062420
H	3.1823770	-2.5984320	1.1780770
H	5.3046290	-1.3261410	1.2034700
H	5.4018860	0.9145370	0.1514680
H	3.3910640	1.8696240	-0.9040940

SeTM_M_1.log

NImag=0

C	-3.4937750	1.0332930	0.1613550
C	-2.2746090	1.9281360	0.3097750
N	-1.1820680	1.2408550	-0.3504340
C	-1.1964060	-0.0878850	-0.2701880

Se	-2.8225010	-0.8190090	0.2614620
C	0.2009040	1.6970340	-0.4483360
C	0.9387060	0.4223130	-0.9022930
N	-0.0352740	-0.6332410	-0.5258710
C	0.2394730	-2.0371090	-0.7131620
C	2.2909940	0.2148620	-0.2915450
C	2.4340930	0.0830960	1.0873780
C	3.6881410	-0.1055480	1.6417190
C	4.8090570	-0.1562080	0.8228680
C	4.6718650	-0.0252850	-0.5496860
C	3.4137000	0.1538350	-1.1061320
H	-3.9761280	1.1478630	-0.8076190
H	-4.2187870	1.1808580	0.9582840
H	-2.0284330	2.0945390	1.3656900
H	-2.4473220	2.8960450	-0.1639120
H	0.5560660	2.0317910	0.5321800
H	0.3060470	2.5126440	-1.1619090
H	1.0284910	0.4066620	-1.9941160
H	1.1772590	-2.2871440	-0.2179660
H	0.3208510	-2.2733630	-1.7773150
H	-0.5626060	-2.6293630	-0.2745230
H	1.5628750	0.1168320	1.7345210
H	3.7941460	-0.2119640	2.7145380
H	5.7904680	-0.3001020	1.2583200
H	5.5440560	-0.0659070	-1.1906550
H	3.3065730	0.2504140	-2.1816270

SeTM_M_2.log

NImag=0

C	-3.0812480	1.1836670	0.7009850
C	-2.2165810	1.9254320	-0.3058370
N	-0.9940240	1.1597770	-0.4327400
C	-1.1007740	-0.1578600	-0.2993630
Se	-2.7149730	-0.7354740	0.4254740
C	0.1754520	1.4805810	-1.2416060
C	0.9625480	0.1463450	-1.2439480
N	-0.0608580	-0.8091180	-0.7494280
C	0.2034240	-2.2219520	-0.6274400
C	2.1941080	0.1285100	-0.3840250
C	2.1143260	0.4085940	0.9781600
C	3.2560790	0.3908720	1.7599390
C	4.4866880	0.0956690	1.1866560
C	4.5712550	-0.1841540	-0.1674450
C	3.4255330	-0.1713060	-0.9503900
H	-4.1435780	1.3567870	0.5459150
H	-2.8121340	1.4180620	1.7292660
H	-1.9915120	2.9327350	0.0493170
H	-2.7144570	2.0021660	-1.2799720
H	0.7537320	2.2901400	-0.7976610
H	-0.1354990	1.7721140	-2.2495250
H	1.2319200	-0.1404500	-2.2624690
H	0.3513070	-2.6597930	-1.6161180
H	-0.6404780	-2.7120540	-0.1438780
H	1.1007020	-2.3793690	-0.0263680
H	1.1573080	0.6408770	1.4355330
H	3.1893280	0.6085780	2.8190630
H	5.3797350	0.0838440	1.7996650
H	5.5290890	-0.4155000	-0.6172330
H	3.4929380	-0.3930260	-2.0105820

SeTM_A_1.log

NImag=0

C	3.47148200	-1.33419200	0.07472900
C	2.24935200	-2.23531900	-0.02379000
N	1.16466100	-1.41057300	-0.53051200
C	1.22409100	-0.12685000	-0.25814100
Se	2.81006300	0.50093900	0.43269200
C	-0.19603600	-1.81588900	-0.85267600
C	-0.93805100	-0.46368300	-1.01768400
N	0.07170000	0.50191100	-0.50507200
C	-0.03094700	1.90426000	-0.34451700
O	0.90787400	2.47838900	0.14154500
C	-1.29313100	2.54600300	-0.79672200
C	-2.24556300	-0.41014600	-0.28457300
C	-2.27815900	-0.41470500	1.10699100
C	-3.49159400	-0.38426600	1.77291900
C	-4.67909600	-0.36045100	1.05289400
C	-4.65005800	-0.36327200	-0.33276900
C	-3.43427200	-0.38306400	-1.00062800
H	4.02851900	-1.29546800	-0.86006000
H	4.13603700	-1.63285200	0.88208900
H	1.96248200	-2.63698300	0.95492500
H	2.42674400	-3.07110100	-0.70232500
H	-0.61864500	-2.39894100	-0.02935600
H	-0.22444300	-2.41643000	-1.76163800
H	-1.10017200	-0.25268700	-2.07726300
H	-1.14691900	3.62317900	-0.79413500
H	-2.10767000	2.28569600	-0.11679400
H	-1.58021200	2.20687500	-1.79398100
H	-1.35522700	-0.42602100	1.67896900
H	-3.51275800	-0.37859700	2.85594300
H	-5.62777300	-0.33752500	1.57521400
H	-5.57449800	-0.34390200	-0.89691700
H	-3.41322000	-0.37419200	-2.08567000

SeTM_A_2.log

NImag=0

C	-3.02939000	-1.65024600	-0.39058200
C	-2.15431900	-2.09912700	0.76718200
N	-0.98029200	-1.24944600	0.75682100
C	-1.10651700	-0.02859600	0.27736300
Se	-2.72421300	0.28547400	-0.59225900
C	0.20506100	-1.36714800	1.59587100
C	0.99405000	-0.08847900	1.24543800
N	-0.04990300	0.72971100	0.55454000
C	0.19329700	2.10993600	0.30678900
O	1.18477700	2.58295000	0.77635600
C	-0.81209100	2.85053800	-0.50940700
C	2.18525600	-0.31434300	0.35901000
C	2.06063600	-1.01669800	-0.83694900
C	3.16794600	-1.22678700	-1.64055500
C	4.40866200	-0.73979700	-1.25138700
C	4.53672500	-0.03955700	-0.06232800
C	3.42610100	0.17729500	0.73893400
H	-4.08740500	-1.81539400	-0.20068600
H	-2.74478700	-2.11726800	-1.33185100
H	-1.85700200	-3.14254600	0.64939600
H	-2.66927600	-1.99069300	1.72859200
H	0.76850300	-2.26657400	1.34981900
H	-0.09196400	-1.40402900	2.64816700
H	1.29902500	0.45280100	2.13922600
H	-1.77622900	2.89852800	0.00505300
H	-0.95391300	2.38128300	-1.48596700
H	-0.44493200	3.86452800	-0.64913300
H	1.09649100	-1.40569200	-1.15175900
H	3.06585500	-1.77264300	-2.57071200
H	5.27575500	-0.90606400	-1.87909500
H	5.50232900	0.34625500	0.24086900
H	3.52141600	0.74398300	1.65826100

OHyperBTM_1.log

NImag=0

C	-5.1038360	0.5742080	-0.1576330
C	-5.3082010	-0.6992650	0.3518340

C	-4.2501200	-1.6007020	0.4988820
C	-3.0117800	-1.1593560	0.1141830
C	-2.7962630	0.1157920	-0.4033360
C	-3.8365490	1.0095640	-0.5487660
O	-1.8244350	-1.8372010	0.1664230
C	-0.8508650	-0.9988980	-0.3029650
N	-1.4457650	0.1838510	-0.6713190
N	0.3657250	-1.3176350	-0.3358630
C	1.2372870	-0.3028820	-0.8968140
C	0.7871110	1.1518070	-0.5977530
C	-0.6583280	1.3286170	-1.0611640
C	1.0456150	1.6362030	0.8402330
C	0.1271820	1.0496870	1.9053050
C	1.0181650	3.1582960	0.9017150
C	2.6699980	-0.5251820	-0.4792360
C	2.9955810	-1.3532600	0.5861530
C	4.3177550	-1.5065540	0.9797200
C	5.3305970	-0.8374260	0.3106570
C	5.0140420	-0.0174230	-0.7638110
C	3.6936880	0.1337540	-1.1541550
H	-5.9457360	1.2484930	-0.2576770
H	-6.3053990	-1.0044520	0.6437030
H	-4.3942410	-2.5955270	0.8999210
H	-3.6821670	2.0027470	-0.9517420
H	1.2113670	-0.4072880	-1.9927470
H	1.3961620	1.7914380	-1.2437930
H	-0.7026090	1.4523670	-2.1481960
H	-1.1025850	2.2209090	-0.6121310
H	2.0668330	1.3223240	1.0798600
H	-0.9179230	1.3296190	1.7402130
H	0.4097070	1.4361790	2.8874700
H	0.1895440	-0.0378790	1.9446830
H	1.7180800	3.6006880	0.1880200
H	1.2932710	3.5067380	1.8998930
H	0.0217550	3.5580190	0.6874690
H	2.1980270	-1.8846710	1.0901550
H	4.5570300	-2.1574350	1.8133070
H	6.3629760	-0.9597010	0.6174840
H	5.7993230	0.5001920	-1.3031560
H	3.4558850	0.7694430	-2.0021510

OHyperBTM_2.log

NImag=0

C	-4.6419780	0.3072040	1.3849280
C	-5.2007800	-0.4443550	0.3623620
C	-4.4261430	-0.9047020	-0.7064150
C	-3.0968680	-0.5747910	-0.6870850
C	-2.5257100	0.1804920	0.3351960
C	-3.2851080	0.6353000	1.3928460
O	-2.1378600	-0.8993580	-1.6074060
C	-0.9555590	-0.3562490	-1.1817230
N	-1.1932250	0.3006250	0.0050480
N	0.1226150	-0.4857690	-1.8183020
C	1.2831080	0.1301770	-1.1945150
C	0.9078160	1.4037780	-0.3893120
C	-0.1546740	1.0623190	0.6480070
C	2.1029100	2.1396010	0.2245750
C	1.6726160	3.2584110	1.1668740
C	3.0070340	2.7188690	-0.8562560
C	2.0645620	-0.8720750	-0.3705540
C	3.4559290	-0.8693600	-0.4054950
C	4.1952710	-1.7474620	0.3718170
C	3.5507200	-2.6536890	1.1997140
C	2.1646140	-2.6800350	1.2305390
C	1.4302460	-1.8013250	0.4488290
H	-5.2699110	0.6470190	2.1996440
H	-6.2570280	-0.6817780	0.3889700
H	-4.8488840	-1.4911550	-1.5118340
H	-2.8512050	1.2170210	2.1965780
H	1.9353110	0.4458440	-2.0115790
H	0.4367080	2.0799700	-1.1158990
H	-0.6010560	1.9701990	1.0571870
H	0.2808940	0.4945070	1.4798570
H	2.6804150	1.4127880	0.8074370
H	1.0140350	3.9703060	0.6584070
H	2.5462360	3.8126920	1.5172070
H	1.1517410	2.8856550	2.0509390
H	3.8735470	3.2105650	-0.4083840
H	2.4677760	3.4675780	-1.4450260
H	3.3795270	1.9625920	-1.5475780
H	3.9706570	-0.1746050	-1.0602890
H	5.2781630	-1.7287720	0.3232010
H	4.1244710	-3.3449890	1.8059660
H	1.6490440	-3.3974150	1.8590030
H	0.3486560	-1.8598000	0.4622210

OHyperBTM_3.log

NImag=0

C	-4.6365220	0.3657230	1.4183650
C	-5.1906530	-0.5406040	0.5271810
C	-4.4261520	-1.1148820	-0.4926640
C	-3.1119740	-0.7350970	-0.5622210
C	-2.5462500	0.1771240	0.3265280
C	-3.2952120	0.7438010	1.3365920
O	-2.1645320	-1.1498330	-1.4578970
C	-0.9932210	-0.5122180	-1.1473000
N	-1.2314740	0.3045560	-0.0639800
N	0.0737630	-0.7021020	-1.7884520
C	1.2305370	0.0397490	-1.3153960
C	0.8340730	1.3738690	-0.6353600
C	-0.1823490	1.1315800	0.4714990
C	2.0160810	2.2508670	-0.1895710
C	2.5198700	1.9768810	1.2234680
C	1.6683620	3.7274030	-0.3384330
C	2.1300080	-0.8258860	-0.4647000
C	1.6184210	-1.6540400	0.5297590
C	2.4651030	-2.3828030	1.3505600
C	3.8399580	-2.3064990	1.1810180
C	4.3597810	-1.5086640	0.1729690
C	3.5086470	-0.7803600	-0.6436480
H	-5.2557840	0.7903790	2.1992210
H	-6.2347950	-0.8122040	0.6205570
H	-4.8448620	-1.8241670	-1.1947640
H	-2.8649680	1.4474230	2.0383350
H	1.8098640	0.3157610	-2.2027460
H	0.3042330	1.9249090	-1.4221340
H	-0.6154780	2.0754260	0.8135150
H	0.2798570	0.6414810	1.3344490
H	2.8396840	2.0370430	-0.8810330
H	1.7904960	2.2987420	1.9727270
H	3.4360100	2.5433370	1.4057740
H	2.7416360	0.9230600	1.3929100
H	1.4010640	3.9736840	-1.3688540
H	2.5117590	4.3586350	-0.0482940
H	0.8229090	4.0006800	0.3008690
H	0.5458920	-1.7462000	0.6547380
H	2.0481710	-3.0212010	2.1212640
H	4.5014980	-2.8789640	1.8207310
H	5.4314350	-1.4580200	0.0171710
H	3.9205550	-0.1639730	-1.4361170

OHyperBTM_4.log

NImag=0

C	-4.7451800	0.2814590	1.3787770
C	-5.2693500	-0.4960190	0.3573080

C	-4.4713590	-0.9339800	-0.7037160
C	-3.1551360	-0.5556840	-0.6782190
C	-2.6190920	0.2268510	0.3427130
C	-3.4013800	0.6595690	1.3929290
O	-2.1789300	-0.8520140	-1.5893170
C	-1.0181260	-0.2662630	-1.1592480
N	-1.2909080	0.3961930	0.0182320
N	0.0674890	-0.3692750	-1.7879290
C	1.2171400	0.2791110	-1.1690560
C	0.7942480	1.5258440	-0.3524230
C	-0.2711790	1.1703680	0.6746900
C	1.8960560	2.4042280	0.2614460
C	2.9181320	2.8324890	-0.7826140
C	2.5916220	1.8423550	1.4972470
C	2.0470020	-0.7352470	-0.4144230
C	3.4060260	-0.8576250	-0.6824200
C	4.1989940	-1.7507470	0.0218280
C	3.6376470	-2.5490250	1.0063390
C	2.2762730	-2.4622230	1.2596180
C	1.4883330	-1.5707350	0.5491940
H	-5.3903760	0.6026350	2.1875460
H	-6.3163080	-0.7719910	0.3787540
H	-4.8669060	-1.5409630	-1.5077400
H	-2.9945150	1.2623750	2.1952880
H	1.8401250	0.6414560	-1.9907400
H	0.2940800	2.1598380	-1.0966710
H	-0.7204200	2.0821230	1.0798660
H	0.1512400	0.6056320	1.5119010
H	1.3728360	3.3153550	0.5836990
H	3.5639480	2.0016550	-1.0734230
H	3.5621810	3.6200130	-0.3847110
H	2.4348930	3.2173260	-1.6846900
H	1.8898020	1.6141870	2.3018730
H	3.2958010	2.5829140	1.8849870
H	3.1520620	0.9339470	1.2758430
H	3.8503460	-0.2440140	-1.4589950
H	5.2564430	-1.8274990	-0.2044490
H	4.2528210	-3.2484930	1.5602760
H	1.8220990	-3.1012570	2.0084480
H	0.4212090	-1.5459100	0.7355370

OHyperBTM_6.log

NImag=0

C	5.1518939	0.5404661	0.2416692
C	5.3500466	-0.6586677	-0.4262805
C	4.2864729	-1.5255651	-0.6921527
C	3.0490827	-1.1278493	-0.2594948
C	2.8396980	0.0728974	0.4156608
C	3.8858527	0.9320307	0.6796353
O	1.8577407	-1.7862952	-0.4029239
C	0.8876296	-1.0095424	0.1672238
N	1.4877366	0.1158530	0.6812715
N	-0.3311748	-1.3268667	0.1737866
C	-1.1948590	-0.3760783	0.8528619
C	-0.7212600	1.0892424	0.6436469
C	0.6849010	1.1827747	1.2287684
C	-0.8392145	1.5341148	-0.8351214
C	0.3014902	2.4170409	-1.3275010
C	-2.1626082	2.2518281	-1.0771048
C	-2.6335816	-0.5783044	0.4544508
C	-2.9765312	-1.2423560	-0.7147789
C	-4.3071925	-1.3571420	-1.0926807
C	-5.3096562	-0.8192348	-0.3006656
C	-4.9750290	-0.1742218	0.8826382
C	-3.6464083	-0.0590379	1.2553966
H	5.9980296	1.1897202	0.4313570
H	6.3466235	-0.9316571	-0.7503307
H	4.4258063	-2.4623999	-1.2158204
H	3.7360881	1.8669033	1.2051720
H	-1.1396360	-0.5695582	1.9346047
H	-1.3552494	1.7395775	1.2544858
H	0.6452712	1.0987040	2.3199779
H	1.1577300	2.1355373	0.9880472
H	-0.8316104	0.6250261	-1.4463592
H	0.3518567	3.3576719	-0.7693887
H	0.1361705	2.6759059	-2.3757744
H	1.2764327	1.9288404	-1.2658534
H	-3.0195390	1.6475650	-0.7813701
H	-2.2757246	2.5013742	-2.1349653
H	-2.1960732	3.1881518	-0.5099547
H	-2.1860521	-1.6756569	-1.3148262
H	-4.5616283	-1.8744871	-2.0109994
H	-6.3484591	-0.9108737	-0.5962482
H	-5.7523480	0.2360417	1.5173290
H	-3.3901943	0.4467330	2.1819485

OHyperBTM_M_1.log

NImag=0

C	-4.7240520	0.6189270	1.3594140
C	-5.2409220	-0.3757090	0.5345420
C	-4.4440600	-1.0474330	-0.3895590
C	-3.1313360	-0.6581670	-0.4228190
C	-2.6024510	0.3301200	0.3888700
C	-3.3861740	0.9971020	1.3066720
O	-2.1140740	-1.1505330	-1.2366450
C	-1.0198370	-0.4755940	-0.9194740
N	-1.2515600	0.4207270	0.0408240
N	0.1359260	-0.6863020	-1.4924710
C	1.3088030	0.0536470	-1.0006750
C	0.8707740	1.4428370	-0.4926100
C	-0.2258890	1.3088120	0.5527220
C	2.0490300	2.2871180	0.0095850
C	1.5781720	3.5731780	0.6787000
C	3.0031110	2.6275740	-1.1279070
C	0.3477000	-1.7487980	-2.4592010
C	2.0931300	-0.7796410	-0.0149690
C	3.4747340	-0.8690410	-0.1449190
C	4.2258770	-1.5968730	0.7647480
C	3.6006330	-2.2552570	1.8120820
C	2.2211310	-2.1900940	1.9391010
C	1.4724580	-1.4584080	1.0308030
H	-5.3804580	1.1131470	2.0644150
H	-6.2886380	-0.6368200	0.6109710
H	-4.8335040	-1.8222040	-1.0363460
H	-2.9898410	1.7683960	1.9542090
H	1.9307820	0.2137930	-1.8829310
H	0.4315400	1.9611330	-1.3554120
H	-0.6915560	2.2740450	0.7460630
H	0.1624480	0.9160730	1.4973680
H	2.5894470	1.6927060	0.7550530
H	2.4387980	4.1890110	0.9435020
H	1.0224000	3.3931210	1.6009620
H	0.9492070	4.1643150	0.0056770
H	2.4975220	3.2268670	-1.8909190
H	3.4223150	1.7471300	-1.6165590
H	3.8427410	3.2141760	-0.7523740
H	1.0665370	-2.4644380	-2.0559620
H	0.7401340	-1.3233340	-3.3840360
H	-0.5891550	-2.2578180	-2.6683160
H	3.9722830	-0.3682940	-0.9681490
H	5.3012950	-1.6561740	0.6485730
H	4.1850960	-2.8284470	2.5213930
H	1.7241840	-2.7158570	2.7455520
H	0.3934450	-1.4430320	1.1413340

OHyperBTM_M_2.log

NImag=0

C	-4.6949810	0.7272380	1.3876640
C	-5.2215870	-0.3773270	0.7246660
C	-4.4445970	-1.1580660	-0.1278520
C	-3.1410250	-0.7594750	-0.2612780
C	-2.6029320	0.3375510	0.3886380
C	-3.3669170	1.1127220	1.2353720
O	-2.1434690	-1.3446180	-1.0371060
C	-1.0507940	-0.6188290	-0.8542090
N	-1.2675020	0.3987860	-0.0190610
N	0.0890190	-0.8973100	-1.4322990
C	1.2645010	-0.0651120	-1.1268200
C	0.8044400	1.3601640	-0.7689620
C	-0.2262800	1.3398010	0.3466240
C	1.9675620	2.3323370	-0.4948030
C	2.4553480	2.3495220	0.9494740
C	1.5861840	3.7391890	-0.9389120
C	0.2788310	-2.0763500	-2.2586070
C	2.1612240	-0.7457670	-0.1251130
C	1.6562570	-1.3336590	1.0311010
C	2.5127070	-1.8958730	1.9644000
C	3.8831800	-1.8801140	1.7493300
C	4.3922030	-1.3158880	0.5896070
C	3.5336920	-0.7585530	-0.3450060
H	-5.3357600	1.3039630	2.0426000
H	-6.2611800	-0.6395180	0.8737360
H	-4.8416790	-2.0190170	-0.6487770
H	-2.9629130	1.9702910	1.7575150
H	1.8123200	0.0168090	-2.0696500
H	0.2875070	1.7233310	-1.6655700
H	-0.6827250	2.3226770	0.4693580
H	0.2112980	1.0400670	1.3014220
H	2.8013860	2.0028240	-1.1251210
H	3.3564290	2.9606090	1.0214950
H	2.6997550	1.3572880	1.3292700
H	1.7143830	2.8017750	1.6148520
H	0.7344290	4.1216600	-0.3683470
H	1.3250210	3.7724120	-1.9990730
H	2.4156660	4.4295100	-0.7772340
H	1.0686400	-2.6919490	-1.8248310
H	0.5682130	-1.7730750	-3.2661810
H	-0.6408250	-2.6527670	-2.3076850
H	0.5871890	-1.3705700	1.2130920
H	2.1087690	-2.3509640	2.8607370
H	4.5520380	-2.3192490	2.4794380
H	5.4600650	-1.3159940	0.4075070
H	3.9370080	-0.3245660	-1.2540620

OHyperBTM_M_3.log

NImag=0

C	-4.7971430	0.6870260	1.3502400
C	-5.2958830	-0.3571140	0.5769990
C	-4.4892150	-1.0539510	-0.3195760
C	-3.1858040	-0.6375250	-0.3801490
C	-2.6751820	0.4000530	0.3800460
C	-3.4686040	1.0923780	1.2703680
O	-2.1623730	-1.1455390	-1.1757900
C	-1.0808160	-0.4326620	-0.8989600
N	-1.3293730	0.5052240	0.0175890
N	0.0754980	-0.6497000	-1.4695950
C	1.2473450	0.1276240	-1.0210570
C	0.7718710	1.5228460	-0.5735450
C	-0.3137040	1.4284630	0.4847770
C	1.8678050	2.5269740	-0.1761200
C	2.9043060	2.6825960	-1.2799980
C	2.5358660	2.2885010	1.1733270
C	0.2962230	-1.7510310	-2.3897900
C	2.0649240	-0.6842670	-0.0473940
C	1.5048450	-1.2431100	1.0985120
C	2.2897260	-1.9592640	1.9876350
C	3.6431460	-2.1344760	1.7376750
C	4.2031580	-1.6079170	0.5842510
C	3.4149370	-0.8937360	-0.3047990
H	-5.4606160	1.1993850	2.0353970
H	-6.3369060	-0.6378330	0.6728910
H	-4.8645750	-1.8673050	-0.9260660
H	-3.0864290	1.9028120	1.8773720
H	1.8485340	0.2759790	-1.9207190
H	0.2937620	1.9460930	-1.4666170
H	-0.7796400	2.4041500	0.6337540
H	0.0741390	1.0832060	1.4453730
H	1.3353160	3.4838960	-0.1029840
H	3.5606480	1.8119360	-1.3430250
H	2.4392130	2.8361550	-2.2576060
H	3.5403340	3.5458670	-1.0789820
H	1.8209690	2.2513000	1.9977750
H	3.1194580	1.3681740	1.1907890
H	3.2176080	3.1147310	1.3836440
H	1.0330450	-2.4340460	-1.9632000
H	0.6705760	-1.3607470	-3.3374310
H	-0.6328560	-2.2863910	-2.5645530
H	0.4452460	-1.1414590	1.3063990
H	1.8422520	-2.3864170	2.8770320
H	4.2560200	-2.6933780	2.4342940
H	5.2545870	-1.7582740	0.3711670
H	3.8586110	-0.4906260	-1.2089900

OHyperBTM_A_1.log

NImag=0

C	-4.69531300	1.43198400	1.06618700
C	-5.23150400	0.18236500	0.75936300
C	-4.45449900	-0.83639100	0.21721700
C	-3.13700300	-0.51617000	0.01302300
C	-2.59040800	0.71789500	0.30955200
C	-3.35629400	1.73210400	0.84828100
O	-2.13113100	-1.32213400	-0.50601700
C	-1.02634200	-0.59691600	-0.52041100
N	-1.24430200	0.62884300	-0.05365800
N	0.15177800	-1.04749000	-0.93388900
C	1.31730600	-0.14669600	-0.74580400
C	0.88309300	1.31274800	-0.95577700
C	-0.23257400	1.66911400	0.00768100
C	2.05913800	2.29404900	-0.86686000
C	1.58475100	3.74244300	-0.88224100
C	3.04733700	2.06820200	-2.00345500
C	0.44254900	-2.35399100	-1.48253500
O	1.57494300	-2.56290400	-1.79681400
C	-0.66108800	-3.34666400	-1.62450900
C	2.01039100	-0.43673200	0.56277500
C	1.31835500	-0.57767900	1.76292000
C	1.99926000	-0.80837600	2.94830700
C	3.38232600	-0.90228000	2.94874100
C	4.07889700	-0.78040600	1.75627900
C	3.39576000	-0.55605400	0.57205500
H	-5.34067700	2.19203200	1.48803500
H	-6.28117000	-0.00334700	0.94800400
H	-4.85950600	-1.80988400	-0.02451600
H	-2.94502800	2.70296000	1.09185600
H	1.99243900	-0.41995200	-1.55233000
H	0.47206900	1.37563400	-1.97244700
H	-0.71272000	2.60590500	-0.27127900
H	0.13219300	1.75328400	1.03563700
H	2.57094600	2.11156400	0.08489900
H	0.99845100	4.00729600	0.00015800
H	0.98485000	3.95480900	-1.77283200

H	2.44456700	4.41342200	-0.90256600
H	3.46777600	1.06201100	-2.01487700
H	3.88320300	2.76395500	-1.91779400
H	2.57029500	2.24367500	-2.97222600
H	-1.08989300	-3.59761000	-0.65321700
H	-0.23024100	-4.23697100	-2.07522500
H	-1.46490700	-2.96542500	-2.25547800
H	0.23486900	-0.53096100	1.79439600
H	1.44616400	-0.92062800	3.87312000
H	3.91441100	-1.08423600	3.87451800
H	5.15809200	-0.87347300	1.74413700
H	3.94617100	-0.48852000	-0.35923000

OHyperBTM_A_2.log

NImag=0

C	-4.64905400	1.50886400	1.11669300
C	-5.20049600	0.24295500	0.92533100
C	-4.44909700	-0.81703200	0.42807500
C	-3.14063200	-0.51968500	0.14597200
C	-2.57934100	0.72992200	0.32758100
C	-3.31970200	1.78499800	0.82171600
O	-2.15946200	-1.36693700	-0.35369400
C	-1.05388100	-0.65132400	-0.46766800
N	-1.25013500	0.60695800	-0.08408100
N	0.10228800	-1.14150000	-0.90010500
C	1.28001000	-0.23377600	-0.86930900
C	0.82266300	1.20679200	-1.13511500
C	-0.22426500	1.63411600	-0.12724700
C	1.99027100	2.20688900	-1.24779500
C	2.44063300	2.80970400	0.07798700
C	1.63607800	3.31083300	-2.23648000
C	0.36285700	-2.49255600	-1.34830300
O	1.47009200	-2.72434700	-1.72893200
C	-0.73515100	-3.50073000	-1.30840000
C	2.09180900	-0.45598600	0.37883600
C	1.51428300	-0.51525500	1.64377900
C	2.30614600	-0.65562400	2.77290900
C	3.68458200	-0.74199500	2.64819900
C	4.26547300	-0.70733600	1.38945000
C	3.47175700	-0.57312800	0.26195900
H	-5.27441000	2.30128700	1.50799200
H	-6.24181700	0.07728000	1.17041900
H	-4.86603600	-1.80350500	0.27579000
H	-2.89647400	2.76888400	0.97621000
H	1.88217600	-0.54538900	-1.72106500
H	0.33012400	1.17764300	-2.11494600
H	-0.70036500	2.57047800	-0.42041500
H	0.19653900	1.75353100	0.87327600
H	2.83458300	1.64726900	-1.66541000
H	2.66137900	2.05695800	0.83507900
H	1.68978500	3.49661200	0.47954700
H	3.34825400	3.39458300	-0.07882700
H	0.77384200	3.89216900	-1.89540000
H	1.40395500	2.90930800	-3.22545200
H	2.46865200	4.00761300	-2.34600400
H	-1.10567800	-3.63998900	-0.29187300
H	-0.32051900	-4.43450000	-1.67935700
H	-1.57756300	-3.19880700	-1.93203100
H	0.43737600	-0.46616000	1.76955400
H	1.84497600	-0.70175600	3.75216200
H	4.30346400	-0.85153600	3.53039800
H	5.33979200	-0.79685600	1.28335200
H	3.92820100	-0.56691700	-0.72171400

OHyperBTM_A_3.log

NImag=0

C	4.75325800	-1.64959300	0.76037400
C	5.27264500	-0.35597800	0.75338100
C	4.48901300	0.74762200	0.43176800
C	3.18247400	0.46372400	0.12763200
C	2.65283300	-0.81257500	0.12761800
C	3.42528100	-1.91127700	0.44647100
O	2.17358000	1.35149900	-0.22370300
C	1.08244500	0.63407700	-0.42920700
N	1.31438700	-0.66269300	-0.24330300
N	-0.09330800	1.15381900	-0.76194100
C	-1.26155700	0.22782100	-0.78289700
C	-0.79449400	-1.14128400	-1.29681400
C	0.31564100	-1.69997000	-0.43132400
C	-1.89821900	-2.18387800	-1.55093600
C	-2.97664000	-1.63142700	-2.47234100
C	-2.51219300	-2.82026100	-0.30919500
C	-0.38311600	2.54294100	-1.03941400
O	-1.50997600	2.80708500	-1.33244900
C	0.71303800	3.55014400	-0.94973100
C	-1.98351000	0.28006900	0.53928600
C	-1.35100600	0.02303000	1.75310900
C	-2.06621700	0.04847400	2.93995800
C	-3.42223500	0.33853500	2.92905600
C	-4.05366600	0.62631800	1.72877100
C	-3.33523200	0.60542200	0.54441600
H	5.40324900	-2.47594900	1.01875700
H	6.31419000	-0.20309400	1.00574200
H	4.88098600	1.75581200	0.42362100
H	3.02711800	-2.91748100	0.45617400
H	-1.91941400	0.65392700	-1.53659800
H	-0.35269100	-0.93336200	-2.28031800
H	0.79888200	-2.54851600	-0.91878400
H	-0.04615400	-2.02118300	0.54731100
H	-1.38681000	-2.98436500	-2.10094500
H	-3.61330000	-0.90761900	-1.95921800
H	-3.62435300	-2.43875800	-2.81690500
H	-2.55098800	-1.14864300	-3.35607400
H	-1.77096300	-3.30793100	0.32760400
H	-3.21829900	-3.59312200	-0.61846000
H	-3.05813600	-2.09912600	0.29879800
H	1.53679900	3.30876700	-1.62255600
H	1.11426100	3.60301100	0.06339700
H	0.28352600	4.51028800	-1.22374500
H	-0.28762700	-0.18564800	1.79879400
H	-1.56164600	-0.15542000	3.87677000
H	-3.98126600	0.35612000	3.85668900
H	-5.10689000	0.87923200	1.71428500
H	-3.82811000	0.85490500	-0.38830100

OHyperBTM_A_4.log

NImag=0

C	-4.72420600	1.33191100	1.08632600
C	-5.24791600	0.09098800	0.72332200
C	-4.46003400	-0.89764500	0.14471700
C	-3.14274300	-0.55940400	-0.03666000
C	-2.61000000	0.66635700	0.31531900
C	-3.38701500	1.65228500	0.89073000
O	-2.13319700	-1.33457200	-0.58286900
C	-1.03857300	-0.60596000	-0.56193500
N	-1.26237200	0.59934100	-0.04434900
N	0.15764500	-1.03407400	-0.97695000
C	1.30704500	-0.13206600	-0.73509200
C	0.85492700	1.33060300	-0.91414200
C	-0.25690000	1.63859200	0.06828800
C	2.01249900	2.33301100	-0.82725800
C	1.50783600	3.77131000	-0.80195900
C	2.97893500	2.15524900	-1.99134100
C	0.33370200	-2.35460600	-1.53348000
O	-0.61447200	-3.04637500	-1.74247900

C	1.74763100	-2.74745300	-1.81748800
C	1.98007700	-0.43280200	0.58316900
C	1.26333900	-0.78472600	1.72285200
C	1.91716700	-1.02192300	2.92260300
C	3.29697400	-0.91483600	2.99745000
C	4.02169000	-0.57961900	1.86373600
C	3.36697200	-0.34461800	0.66558600
H	-5.37917400	2.06806500	1.53496000
H	-6.29748500	-0.10987200	0.89659100
H	-4.85193100	-1.86400100	-0.14276700
H	-2.98614000	2.61563800	1.17773600
H	2.01035100	-0.33910200	-1.53732200
H	0.42930100	1.40241500	-1.92413100
H	-0.73961100	2.58628800	-0.16508200
H	0.11085600	1.67497800	1.09832400
H	2.54907300	2.14588600	0.10937600
H	0.94081300	4.00531100	0.10116100
H	0.87920200	3.98782500	-1.67132900
H	2.35264900	4.46073100	-0.83203500
H	2.47784400	2.35157200	-2.94374500
H	3.41597000	1.15648000	-2.04443900
H	3.80675200	2.86041500	-1.90468000
H	2.14306600	-2.18345100	-2.66670300
H	1.73892300	-3.80250000	-2.07892600
H	2.40035700	-2.58071700	-0.95921300
H	0.18582100	-0.90216300	1.69168200
H	1.34483300	-1.30026500	3.79925900
H	3.80729000	-1.10502500	3.93369100
H	5.10178800	-0.50956000	1.90846800
H	3.94546700	-0.09613000	-0.21747500

OHyperBTM_A_5.log

NImag=0

C	-4.70922600	1.40379100	1.09512200
C	-5.23714600	0.12984300	0.88432700
C	-4.46410400	-0.91163900	0.38381500
C	-3.15704000	-0.58985400	0.11775700
C	-2.62050800	0.66761000	0.31917900
C	-3.38254500	1.70611100	0.81702000
O	-2.16295100	-1.41489300	-0.38055600
C	-1.07209800	-0.68603000	-0.47698600
N	-1.28660800	0.56660500	-0.08061000
N	0.10605800	-1.15299800	-0.90512000
C	1.26195000	-0.22206700	-0.86058400
C	0.77414400	1.21382900	-1.11740000
C	-0.27656400	1.60590400	-0.10082600
C	1.91436200	2.24501600	-1.22904200
C	2.33625400	2.87058700	0.09574300
C	1.53304400	3.33208800	-2.22659700
C	0.26883400	-2.53657200	-1.29138500
O	-0.66344500	-3.27896500	-1.26662000
C	1.64709600	-2.92730200	-1.71660800
C	2.08279400	-0.41691200	0.38757700
C	1.50031000	-0.58146700	1.64023000
C	2.29149900	-0.69790200	2.77288400
C	3.67331700	-0.65718900	2.66448500
C	4.26226100	-0.51492600	1.41687500
C	3.47027300	-0.40146700	0.28587800
H	-5.35231400	2.18085300	1.48856100
H	-6.27810200	-0.05502400	1.11712200
H	-4.85915800	-1.90437600	0.21508500
H	-2.97822500	2.69546400	0.98684700
H	1.87958800	-0.48043800	-1.71957400
H	0.27597100	1.17689500	-2.09394000
H	-0.76251500	2.54264900	-0.37598600
H	0.14390400	1.71252000	0.90153600
H	2.77913200	1.71123200	-1.63899600
H	2.56982900	2.13150100	0.86247400

H	1.56252200	3.53930000	0.48360600
H	3.22887900	3.47909500	-0.05713500
H	0.64929200	3.88578100	-1.89532300
H	1.32163400	2.91857300	-3.21526200
H	2.34322000	4.05525600	-2.33284000
H	1.91961100	-2.43626300	-2.65453700
H	1.63795400	-4.00239400	-1.87603400
H	2.39420100	-2.67017600	-0.96413800
H	0.42262800	-0.63849700	1.75047800
H	1.82613600	-0.82800200	3.74244100
H	4.29032100	-0.75054300	3.54977500
H	5.34129600	-0.49983900	1.32248500
H	3.93776500	-0.29667200	-0.68792600

ODHPB.log

NImag=0

C	-3.4344106	-0.1887262	0.0698504
C	-3.0368906	1.1365407	-0.0180936
C	-1.6884383	1.4914127	-0.0886663
C	-0.7685472	0.4639559	-0.0639169
C	-1.1787402	-0.8650029	0.0237184
C	-2.4980809	-1.2266628	0.0921207
N	0.6075660	0.4232827	-0.1104075
C	1.0293090	-0.8895840	-0.0511723
O	-0.0835272	-1.6836381	0.0300401
C	1.5587417	1.5060913	-0.1319906
C	2.8691847	0.9918444	0.4430299
C	3.2441821	-0.3535196	-0.1706616
N	2.1984128	-1.3543059	-0.0658769
H	-4.4892061	-0.4284544	0.1216789
H	-3.7876647	1.9173176	-0.0350954
H	-1.3832318	2.5277653	-0.1632902
H	-2.7936195	-2.2655292	0.1612540
H	1.1645073	2.3340014	0.4633841
H	1.6941706	1.8679664	-1.1579283
H	2.7599223	0.8713310	1.5249940
H	3.6583147	1.7268414	0.2711650
H	4.1390354	-0.7423053	0.3210163
H	3.5103654	-0.2187096	-1.2279853

ODHPB_M.log

NImag=0

C	-3.6220381	-0.5670329	0.0596838
C	-3.4515368	0.8115751	-0.0292458
C	-2.1901735	1.3950042	-0.0888958
C	-1.1189201	0.5274126	-0.0532976
C	-1.3055205	-0.8406767	0.0336978
C	-2.5363832	-1.4385125	0.0933500
N	0.2672939	0.7164755	-0.0877459
C	0.8407802	-0.4866675	-0.0261127
O	-0.0549216	-1.4555951	0.0475278
C	1.0303099	1.9510135	-0.1490884
C	2.4064663	1.6829629	0.4328618
C	3.0297891	0.4343508	-0.1639996
N	2.1314594	-0.7134544	-0.0359113
C	2.6923546	-2.0532222	-0.0359054
H	-4.6234738	-0.9756407	0.1031652
H	-4.3252826	1.4503341	-0.0546012
H	-2.0664223	2.4676128	-0.1632308
H	-2.6555034	-2.5115115	0.1621090
H	0.5006469	2.7081722	0.4299791
H	1.0837794	2.2895344	-1.1872881
H	2.3279784	1.5685503	1.5169706
H	3.0566906	2.5365103	0.2404037
H	3.9512011	0.1826876	0.3624861
H	3.2787070	0.5761835	-1.2210428
H	3.2547822	-2.2162091	-0.9574791

H	1.8973182	-2.7907892	0.0317425
H	3.3613838	-2.1671781	0.8183911

DHPB_O_A_1.log

NImag=0

C	-3.83548700	-1.10854900	0.01794800
C	-3.96857800	0.27855500	-0.02692900
C	-2.86629500	1.12343900	-0.06049200
C	-1.63234000	0.50475200	-0.04626100
C	-1.51794800	-0.87146700	-0.00293000
C	-2.58935800	-1.72640400	0.03171600
N	-0.32037800	0.98914400	-0.06270500
C	0.50121700	-0.05683200	-0.03979400
O	-0.16556100	-1.19421700	-0.00221200
C	0.12021000	2.37653500	-0.04564700
C	1.54595200	2.40752900	0.46341500
C	2.39619800	1.37339900	-0.24237200
N	1.82894400	0.02214800	-0.07154700
C	2.77287800	-1.07555400	-0.02538100
O	3.92382100	-0.79095000	-0.15946700
C	2.26703000	-2.46066400	0.19310200
H	-4.72551100	-1.72431300	0.04193000
H	-4.96076400	0.71161600	-0.03734900
H	-2.97769200	2.19916800	-0.09943900
H	-2.47402600	-2.80142200	0.06612000
H	-0.55362200	2.93305700	0.60674600
H	0.03584800	2.78343500	-1.05644600
H	1.56093900	2.22674400	1.54141200
H	1.97252300	3.39625200	0.29328500
H	3.40401200	1.33486800	0.16242000
H	2.47916400	1.58094700	-1.31269700
H	1.65653700	-2.53009700	1.09410200
H	3.13783500	-3.10487000	0.28448000
H	1.65785000	-2.79152800	-0.64961400

DHPB_O_A_2.log

NImag=0

C	3.86899200	-1.11715200	0.00253400
C	3.99721500	0.27157200	0.04236300
C	2.89255600	1.11275900	0.06490700
C	1.66131000	0.48767500	0.04569600
C	1.55041600	-0.88922200	0.00837000
C	2.62650000	-1.74007500	-0.01676700
N	0.34655700	0.96304300	0.04926300
C	-0.46586700	-0.09151200	0.02538300
O	0.20469800	-1.21940500	-0.00151000
C	-0.09841500	2.34664700	0.03500100
C	-1.52899500	2.37479400	-0.45791600
C	-2.36574500	1.33128500	0.25568800
N	-1.80272400	-0.01537500	0.06213400
C	-2.62977900	-1.19573800	-0.04089000
O	-2.13756900	-2.24590300	-0.31234900
C	-4.08747900	-0.97976400	0.21266500
H	4.76201400	-1.72888600	-0.01363400
H	4.98798800	0.70779500	0.05682600
H	3.00026200	2.18907500	0.09892100
H	2.51150500	-2.81516300	-0.04876700
H	0.56368400	2.90652200	-0.62656300
H	-0.00371900	2.75766200	1.04337500
H	-1.55614900	2.19269500	-1.53532500
H	-1.95789400	3.36133000	-0.28077500
H	-3.37376200	1.33036200	-0.14776700
H	-2.42945700	1.54265200	1.32741700
H	-4.27252500	-0.41127400	1.12558300
H	-4.54659000	-1.96204700	0.29201100
H	-4.55346600	-0.44955400	-0.62241300

TeDHPB.log

NImag=0

C	-3.4510617	1.2251458	0.0000883
C	-2.7106311	2.3939781	-0.0680240
C	-1.3236103	2.3579532	-0.0886974
C	-0.6602361	1.1348353	-0.0324860
C	-1.4159801	-0.0466647	0.0211932
C	-2.7972613	0.0001938	0.0394730
N	0.7219809	1.0164818	-0.0374029
C	1.3073767	-0.2375429	-0.0852213
Te	-0.1845895	-1.7207924	0.0411645
C	1.5808985	2.1879921	-0.0231338
C	2.9637104	1.8130045	0.4710299
C	3.4693609	0.5851737	-0.2620364
N	2.5350783	-0.5202330	-0.1865804
H	-4.5334884	1.2611767	0.0162010
H	-3.2133452	3.3529450	-0.1113646
H	-0.7671836	3.2833141	-0.1605858
H	-3.3727838	-0.9172931	0.0828000
H	1.1356517	2.9329742	0.6402247
H	1.6299116	2.6250258	-1.0280844
H	2.9168920	1.5954071	1.5422831
H	3.6377055	2.6621047	0.3370103
H	4.4245928	0.2552415	0.1532716
H	3.6565678	0.8232335	-1.3179997

DHPB_TeM_1.log

NImag=0

C	-0.8030630	3.8622831	2.4128582
C	-0.5085675	2.5078822	2.4894102
C	0.1979259	1.8583944	1.4892760
C	0.6408683	2.5745854	0.3815780
C	0.3360699	3.9435554	0.3189659
C	-0.3861510	4.5874855	1.3090285
N	1.3533508	2.0037582	-0.6645022
C	1.6376298	2.7456244	-1.7826469
Te	1.0240734	4.7692796	-1.4521539
C	1.7399872	0.5932140	-0.6937472
C	2.9165155	0.4077035	-1.6350072
C	2.6534686	1.0498615	-2.9846411
N	2.1900611	2.4264444	-2.8569156
C	2.9535144	5.3899763	-0.8137451
H	-1.3619772	4.3487627	3.2015017
H	-0.8451344	1.9349337	3.3450109
H	0.3884192	0.7966966	1.5709462
H	-0.6225465	5.6417492	1.2284606
H	0.8810623	-0.0117299	-1.0016834
H	2.0203005	0.2971022	0.3176961
H	3.1164118	-0.6586869	-1.7501421
H	3.8083245	0.8558649	-1.1876300
H	1.8910807	0.5007815	-3.5479526
H	3.5519510	1.0548498	-3.6027428
H	3.6001396	5.3382976	-1.6893259
H	3.2809748	4.7087733	-0.0320081
H	2.8762103	6.4117576	-0.4468881

DHPB_TeM_2.log

NImag=0

C	-0.7291217	3.8553479	2.4521274
C	-0.3206343	2.5362042	2.5912196
C	0.3889436	1.8847904	1.5942149
C	0.7057402	2.5604636	0.4200675
C	0.3037770	3.9003405	0.3027918
C	-0.4157534	4.5462813	1.2929862
N	1.3985378	1.9777199	-0.6329775
C	1.7463222	2.7336242	-1.7210854

Te	0.8910061	4.6867914	-1.5232204
C	1.8527562	0.5863102	-0.6087814
C	2.1749487	0.1224197	-2.0174519
C	3.0458421	1.1283748	-2.7488740
N	2.4607399	2.4641760	-2.7103519
C	2.7391066	5.5691351	-0.9606542
H	-1.2876158	4.3438652	3.2398247
H	-0.5596039	1.9950186	3.4987385
H	0.6979645	0.8582124	1.7387548
H	-0.7293317	5.5761515	1.1710239
H	1.0534446	-0.0254471	-0.1871761
H	2.7256121	0.5069285	0.0473003
H	1.2448664	-0.0168736	-2.5752760
H	2.6686495	-0.8491742	-1.9661046
H	3.1788754	0.8506137	-3.7945680
H	4.0468906	1.1852928	-2.3069135
H	3.4029169	5.4444748	-1.8163233
H	3.1097727	5.0401559	-0.0859155
H	2.5662478	6.6226021	-0.7497755

TeDHPB_M.log

NImag=0

C	3.8485365	0.4637643	0.0212744
C	3.3973042	1.7745149	0.0993440
C	2.0432829	2.0630423	0.1165773
C	1.1283789	1.0191633	0.0494454
C	1.5866701	-0.2975473	-0.0134957
C	2.9419289	-0.5818093	-0.0300169
N	-0.2652359	1.2102329	0.0567865
C	-1.0522959	0.1291559	0.0704428
Te	0.0157373	-1.6481817	-0.0436027
C	-0.8370026	2.5567628	0.0556124
C	-2.2326042	2.5251465	-0.5238222
C	-3.0693505	1.4733009	0.1630180
N	-2.3681806	0.1911266	0.1470349
C	-3.1641569	-1.0193257	0.1756350
H	4.9106047	0.2539980	0.0070931
H	4.1096040	2.5882423	0.1524441
H	1.7189903	3.0918061	0.1973362
H	3.2948688	-1.6045958	-0.0807373
H	-0.1944910	3.1887782	-0.5555242
H	-0.8304661	2.9478016	1.0772739
H	-2.1811984	2.3138080	-1.5950496
H	-2.6967777	3.5043133	-0.4043589
H	-4.0192086	1.3364806	-0.3552612
H	-3.2897191	1.7473715	1.2003755
H	-4.1218660	-0.8013986	0.6455744
H	-2.6680285	-1.7838103	0.7796672
H	-3.3420726	-1.4035884	-0.8326934

DHPB_Te_A_1.log

NImag=0

C	4.20989700	-0.03527100	0.08110100
C	3.94494400	1.32926200	0.10125500
C	2.64687200	1.80506000	0.06950100
C	1.59550200	0.89385300	0.01876400
C	1.86620800	-0.47152200	0.01498700
C	3.16907600	-0.94520300	0.03903600
N	0.24124000	1.28008200	-0.02076400
C	-0.70786300	0.33864500	-0.00191300
Te	0.12864600	-1.57346500	-0.02220200
C	-0.04741300	2.71838400	-0.12069600
C	-1.48023400	2.95148600	-0.51182500
C	-2.37025200	2.07354600	0.32264900
N	-2.01253100	0.66500000	0.08817200
C	-3.13121900	-0.23396100	0.09195500
O	-4.19926100	0.21229400	0.39443500
C	-2.94691600	-1.66164100	-0.30270500
H	5.23266300	-0.38994200	0.10397400
H	4.76349300	2.03665300	0.14420200
H	2.47277500	2.87153700	0.09727300
H	3.37532100	-2.00852600	0.03057000
H	0.63218700	3.12883300	-0.86710600
H	0.18447800	3.17634900	0.84457600
H	-1.63206700	2.73512000	-1.57234700
H	-1.73195200	4.00026700	-0.35237400
H	-3.41977900	2.17776100	0.06597600
H	-2.26772300	2.28895800	1.39036100
H	-2.49325300	-2.23549800	0.51189500
H	-2.36221900	-1.77984700	-1.21720500
H	-3.94200800	-2.07126900	-0.46529100

DHPB_Te_A_2.log

NImag=0

C	-4.21098000	-0.08370700	-0.05652500
C	-3.97840700	1.28655500	-0.13071700
C	-2.69153400	1.78952300	-0.12836000
C	-1.62566600	0.89714400	-0.04899200
C	-1.85051300	-0.47781800	0.00965200
C	-3.15054700	-0.96796800	0.00993600
N	-0.27817900	1.30857700	-0.03631700
C	0.65269500	0.36292900	-0.02203500
Te	-0.07214100	-1.56060200	0.05983100
C	0.04927200	2.73584700	-0.01257800
C	1.44354200	2.94207900	0.52437700
C	2.42020200	2.04489500	-0.19497200
N	1.97840900	0.65300400	-0.07096200
C	2.88099900	-0.41231200	-0.07834100
O	2.45623700	-1.54780000	0.00268700
C	4.33472400	-0.10619800	-0.19176500
H	-5.22650900	-0.45979300	-0.05709900
H	-4.81361300	1.97265600	-0.19473700
H	-2.53711800	2.85746300	-0.20324900
H	-3.33803600	-2.03430700	0.05858200
H	-0.67912200	3.22816700	0.62980700
H	-0.06065800	3.13741700	-1.02398400
H	1.46745500	2.73025200	1.59653400
H	1.73355900	3.98426500	0.38871500
H	3.41120500	2.13111700	0.24238300
H	2.49001700	2.30583300	-1.25553700
H	4.69673100	0.39950600	0.70748000
H	4.54916300	0.53364600	-1.04992200
H	4.86405600	-1.04942200	-0.29883000

TeBTM.log

E = -955.824790

H = -955.574940

G = -955.635098

NImag=0

C	-3.9310215	2.2466944	0.6887106
C	-2.8995188	3.1187136	0.3750552
C	-1.6632614	2.6413639	-0.0348722
C	-1.4588675	1.2690041	-0.1314032
C	-2.5022470	0.3864515	0.1953149
C	-3.7296610	0.8736854	0.5991060
N	-0.2807546	0.6948833	-0.5401861
C	-0.1049301	-0.6731791	-0.5183809
Te	-1.8985690	-1.6095943	-0.0065091
C	1.0137725	1.3057776	-0.7608488
C	1.8843037	0.0534347	-1.0951705
N	1.0519438	-1.1178553	-0.7891392
C	3.1949018	0.0595041	-0.3573195
C	3.3411902	-0.6131781	0.8491338
C	4.5380836	-0.5488858	1.5467270

C	5.6004024	0.1899702	1.0472929
C	5.4626698	0.8595923	-0.1599151
C	4.2671650	0.7902394	-0.8580559
H	-4.8929033	2.6296297	1.0067147
H	-3.0551550	4.1884894	0.4494915
H	-0.8605062	3.3266983	-0.2798307
H	-4.5347116	0.1931842	0.8516811
H	1.3624647	1.8146140	0.1444209
H	0.9836015	2.0276426	-1.5795827
H	2.0974820	0.0378264	-2.1702647
H	2.5126872	-1.2022236	1.2240868
H	4.6430835	-1.0834723	2.4840931
H	6.5359108	0.2375348	1.5926171
H	6.2913136	1.4305803	-0.5631334
H	4.1673431	1.3048382	-1.8091087

TeBTM_M.log

E = -995.507665

H = -995.214198

G = -995.277867

NImag=0

C	1.5341595	1.3871182	-0.0900550
C	0.4290779	1.9187209	0.5627073
C	-0.7570536	1.2087885	0.6463847
C	-0.8214626	-0.0489329	0.0649715
C	0.2914889	-0.5873437	-0.5882461
C	1.4724190	0.1287580	-0.6701977
N	-1.9571535	-0.8502396	0.0773179
C	-1.9213369	-2.0667015	-0.4659218
Te	-0.1082902	-2.4939531	-1.3482979
C	-3.2258755	-0.6280028	0.7631160
C	-4.0600180	-1.8632722	0.3526853
N	-3.0463655	-2.7245415	-0.2995515
C	-3.3768306	-4.0090087	-0.8606574
C	-4.7580393	-2.5567400	1.4847059
C	-6.1415390	-2.6697155	1.4801100
C	-6.7942631	-3.3022706	2.5286830
C	-6.0631317	-3.8323874	3.5793395
C	-4.6776836	-3.7291539	3.5845359
C	-4.0273662	-3.0914029	2.5426730
H	2.4538860	1.9554674	-0.1477510
H	0.4912395	2.9012853	1.0133142
H	-1.6164638	1.6268881	1.1564297
H	2.3400441	-0.2785197	-1.1747679
H	-3.0643993	-0.5904844	1.8428249
H	-3.6855114	0.3051360	0.4385198
H	-4.7974926	-1.5861035	-0.4077478
H	-3.9950107	-4.5578430	-0.1504888
H	-2.4640030	-4.5789166	-1.0358166
H	-3.9217191	-3.8991245	-1.8026227
H	-6.7143521	-2.2587061	0.6551346
H	-7.8744482	-3.3823511	2.5215691
H	-6.5711706	-4.3291377	4.3970474
H	-4.1052215	-4.1464513	4.4041000
H	-2.9439132	-3.0187580	2.5520522

TeBTM_A.log

E = -1108.785143

H = -1108.480442

G = -1108.547546

NImag=0

C	-0.8914674	2.0362719	4.0691891
C	-0.3246877	1.6166798	2.8694538
C	-0.8092737	0.5020500	2.2101458
C	-1.8739071	-0.1859524	2.7755121
C	-2.4541295	0.2266374	3.9827437
C	-1.9544408	1.3470547	4.6297206
N	-2.4496600	-1.3209661	2.2079045
C	-3.4616985	-1.8905493	2.8290705
Te	-4.0446535	-1.0213383	4.5632891
C	-2.1322314	-2.0101711	0.9609682
C	-3.0624134	-3.2574010	0.9978280
N	-3.9149203	-2.9697013	2.1766269
C	-4.9891834	-3.6905610	2.7070976
C	-5.4528603	-4.8786356	1.9447258
O	-5.4712877	-3.2948366	3.7458344
C	-2.3190945	-4.5562592	1.1290428
C	-1.6610247	-4.8823509	2.3114649
C	-0.9541433	-6.0691086	2.4074383
C	-0.8962660	-6.9340804	1.3225415
C	-1.5488369	-6.6113267	0.1431757
C	-2.2621219	-5.4252679	0.0478126
H	-0.4992268	2.9115540	4.5719132
H	0.5051610	2.1671216	2.4444337
H	-0.3688150	0.1755117	1.2756961
H	-2.3852247	1.6872987	5.5638663
H	-1.0819178	-2.3012811	0.9410075
H	-2.3427788	-1.3566078	0.1124435
H	-3.6893910	-3.2803026	0.1055510
H	-6.3564376	-5.2612086	2.4117752
H	-4.6790030	-5.6501575	1.9476406
H	-5.6542955	-4.6204881	0.9023649
H	-1.7045890	-4.2173284	3.1686515
H	-0.4480880	-6.3219263	3.3312537
H	-0.3440771	-7.8627438	1.4000309
H	-1.5090544	-7.2857088	-0.7035437
H	-2.7798605	-5.1784204	-0.8735700

15. References

[1] C. M. Young, A. Elmi, D. J. Pascoe, R. K. Morris, C. McLaughlin, A. M. Woods, A. B. Frost, A. de La Houpliere, K. B. Ling, T. K. Smith, A. M. Z. Slawin, P. H. Willoughby, S. L. Cockroft, A. D. Smith, *Angew. Chem. Int. Ed.* **2020**, *59*, 3705–3710.

[2] M. Brindisi, S. Maramai, S. Gemma, S. Brogi, A. Grillo, L. Di Cesare Mannelli, E. Gabellieri, S. Lamponi, S. Saponara, B. Gorelli, D. Tedesco, T. Bonfiglio, C. Landry, K-M. Jung, A. Armirotti, L. Luongo, A. Ligresti, F. Piscitelli, C. Bertucci, M-P. Dehouck, G. Campiani, S. Maione, C. Ghelardini, A. Pittaluga, D. Piomelli, V. Di Marzo, S. Butini, *J. Med. Chem.* **2016**, *59*, 2612–2632.

[3] N. R. Guha, R. M. Neyyappadath, M. D. Greenhalgh, R. Chisholm, S. M. Smith, M. L. McEvoy, C. Rodríguez-Escrich, M. A. Pericàs, G. Hähner, A. D. Smith, *Green Chem.* **2018**, *20*, 4537-4546.

[4] R. Nickisch, S. M. Gabrielsen, M. A. R. Meier, *ChemistrySelect* **2020**, *5*, 11915–11920.

[5] G. Picci, R. Mocci, G. Ciancaleoni, V. Lippolis, M. Zielińska-Błajet, C. Caltagirone, *ChemPlusChem* **2020**, *85*, 1389–1395.

[6] V. Thakur, A. Kumar, N. Sharma, A. K. Shil, P. Das, *Adv. Synth. Catal.* **2018**, *360*, 432–437.

[7] S.-H. Chu, H. G. Mautner, *J. Org. Chem.* **1962**, *27*, 2899–2901.

[8] R. N. Hanson, R. W. Giese, M. A. Davis, S. M. Costello, *J. Med. Chem.* **1978**, *21*, 496–498.

[9] A. Kremer, C. Aurisicchio, F. de Leo, B. Ventura, J. Wouters, N. Armaroli, A. Barbieri, D. Bonifazi, *Chem. Eur. J.* **2015**, *21*, 15377–15387.

[10] E. van de Winckel, R. J. Schneider, A. de La Escosura, T. Torres, *Chem. Eur. J.* **2015**, *21*, 18551–18556.

[11] C. Palomo, J. M. Aizpurua, E. Balentova, A. Jimenez, J. Oyarbide, R. M. Fratila, J. I. Miranda, *Org. Lett.* **2007**, *9*, 101–104.

[12] H. Munch, J. S. Hansen, M. Pittelkow, J. B. Christensen, U. Boas, *Tetrahedron Lett.* **2008**, 49, 3117–3119.

[13] T. Isobe, K. Fukuda, K. Yamaguchi, H. Seki, T. Tokunaga, T. Ishikawa, *J. Org. Chem.* **2000**, *65*, 7779–7785.

[14] A. S. Pankova, M. A. Samartsev, I. A. Shulgin, P. R. Golubev, M. S. Avdontceva, M. A. Kuznetsov, *RSC Adv.* **2014**, *4*, 51780–51786.

[15] G. M. Sheldrick, *Acta Cryst. A* **2008**, *64*, 112-122.

[16] G. M Sheldrick, *Acta Cryst. C* **2015**, *71*, 3-8.

[17] C. M. Young, D. G. Stark, T. H. West, J. E. Taylor, A. D. Smith, *Angew. Chem. Int. Ed*. **2016**, *55*, 14394–14399.

[18] D. Weinzierl, M. Waser, *Beilstein J. Org. Chem.* **2021**, *17*, 800–804.

[19] H. Mayr, T. Bug, M. F. Gotta, N. Hering, B. Irrgang, B. Kempf, R. Loos, A. R. Ofial, G. Remennikov, H. Schimmel, *J. Am. Chem. Soc.* **2001**, *123*, 9500-9512.

[20] H. Mayr, J. Ammer, M. Baidya, B. Maji, T. A. Nigst, A. R. Ofial, T. Singer, *J. Am. Chem. Soc.* **2015**, *137*, 2580-2599.

[21] B. Maji, C. Joannesse, T. A. Nigst, A. D. Smith, H. Mayr, *J. Org. Chem.* **2011**, *76*, 5104–5112.

[22] M. Wallace, D. J. Adams, J. A. Iggo, *Anal. Chem.* **2018**, *90*, 4160-4166.

[23] G. Schenck, K. Baj, J. A. Iggo, M. Wallace, *Anal. Chem.* **2022**, *94*, 8115-8119.

[24] T. L. Hwang, A. J. Shaka, *J. Magn. Reson., Series A* **1995**, *112*, 275-279.

[25] J. F. Coetzee, G. R. Padmanabhan, *J. Am. Chem. Soc.* **1965**, *87*, 5005–5010.

[26] I. Kaljurand, A. Kütt, L. Sooväli, T. Rodima, V. Mäemets, I. Leito, I. A. Koppel I, *J. Org. Chem.* **2005**, *70*, 1019–1028.

[27] L. S. Vogl, M. Bechmann, M. Waser, *Eur. J. Org. Chem.* **2025**, e202401412.

[28] S. Searles, M. Tamres, F. Block, L. A. Quarterman, *J. Am. Chem. Soc.* **1956**, *78*, 4917–4920.

[29] A. B. Teitel´baum, L. A. Kudryavtseva, V. E. Bel´skii, B. E. Ivanov, *Russ. Chem. Bul.l* **1980**, *29*, 1571-1580.

[30] I. M. Kolthoff, M. K. Chantooni, *J. Chem. Eng. Data* **1999**, *44*, 124–129.

[31] I. M. Kolthoff, M. K. Chantooni Jr., S. Bhowmik, *J. Am. Chem. Soc.* **1968**, *90*, 23–28.

[32] R. M. Crampton, I. A. Robotham, *J. Chem. Res. (S)* **1997**, 22-23.

[33] R. Carabias-Martínez, E. Rodríguez-Gonzalo, J. Domínguez-Alvarez, E. Miranda-Cruz, *Anal. Chim. Acta* **2007**, *584*, 410-418.

[34] R. L. Benoit, D. Lefebvre, M. Fréchette, *Can. J. Chem.* **1987**, *65*, 996-1001.

[35] E. M. Arnett, S. G. Maroldo, S. L. Schilling, J. A. Harrelson, *J. Am. Chem. Soc.* **1984**, *106*, 6759–6767.

[36] F. Maran, D. Celadon, M. G. Severin, E. Vianello, *J. Am. Chem. Soc.* **1991**, *113*, 9320–9329.

[37] Schrödinger Release 2019-4: MacroModel, Schrödinger, LLC, New York, NY, **2019**.

[38] Gaussian 16, Revision B.01, Frisch, M. J.; Trucks, G. W.; Schlegel, H. B.; Scuseria, G. E.; Robb, M. A.; Cheeseman, J. R.; Scalmani, G.; Barone, V.; Petersson, G. A.; Nakatsuji, H.; Li, X.; Caricato, M.; Marenich, A. V.; Bloino, J.; Janesko, B. G.; Gomperts, R.; Mennucci, B.; Hratchian, H. P.; Ortiz, J. V.; Izmaylov, A. F.; Sonnenberg, J. L.; Williams-Young, D.; Ding, F.; Lipparini, F.;

Egidi, F.; Goings, J.; Peng, B.; Petrone, A.; Henderson, T.; Ranasinghe, D.; Zakrzewski, V. G.; Gao, J.; Rega, N.; Zheng, G.; Liang, W.; Hada, M.; Ehara, M.; Toyota, K.; Fukuda, R.; Hasegawa, J.; Ishida, M.; Nakajima, T.; Honda, Y.; Kitao, O.; Nakai, H.; Vreven, T.; Throssell, K.; Montgomery, J. A., Jr.; Peralta, J. E.; Ogliaro, F.; Bearpark, M. J.; Heyd, J. J.; Brothers, E. N.; Kudin, K. N.; Staroverov, V. N.; Keith, T. A.; Kobayashi, R.; Normand, J.; Raghavachari, K.; Rendell, A. P.; Burant, J. C.; Iyengar, S. S.; Tomasi, J.; Cossi, M.; Millam, J. M.; Klene, M.; Adamo, C.; Cammi, R.; Ochterski, J. W.; Martin, R. L.; Morokuma, K.; Farkas, O.; Foresman, J. B.; Fox, D. J. Gaussian, Inc., Wallingford CT, **2016**.

[39] C. Adamo, V. Barone, *J. Chem. Phys.* **1999**, *110*, 6158–6170.

[40] F. Weigend, R. Ahlrichs, Phys. *Chem. Chem. Phys.* **2005**, *7*, 3297–3305.

[41] S. Grimme, *J. Comp. Chem.* **2006**, *27*, 1787-1799.

[42] S. Grimme, J. Antony, S. Ehrlich, H. Krieg, *J. Chem. Phys.* **2010**, *132*, 154104.

[43] S. Grimme, S. Ehrlich, L. Goerigk, *J. Comp. Chem.* **2011**, 32, 1456–1465.

[44] J.-D. Chai, M. Head-Gordon, Phys. *Chem. Chem. Phys.* **2008**, *10*, 6615–6620.

[45] A. V. Marenich, C. J. Cramer, D. G. Truhlar, *J. Phys. Chem. B* **2009**, *113*, 6378–6396.

[46] Y. Zhao, D. G. Truhlar, *Theor. Chem. Account* **2008**, *120*, 215–241.

[47] R. Krishnan, J. S. Binkley, R. Seeger, J. A. Pople, *J. Chem. Phys.* **1980**, *72*, 650–654.

16. NMR Spectra for Novel Compounds

3-(benzo[d]oxazol-2-ylamino)propan-1-ol

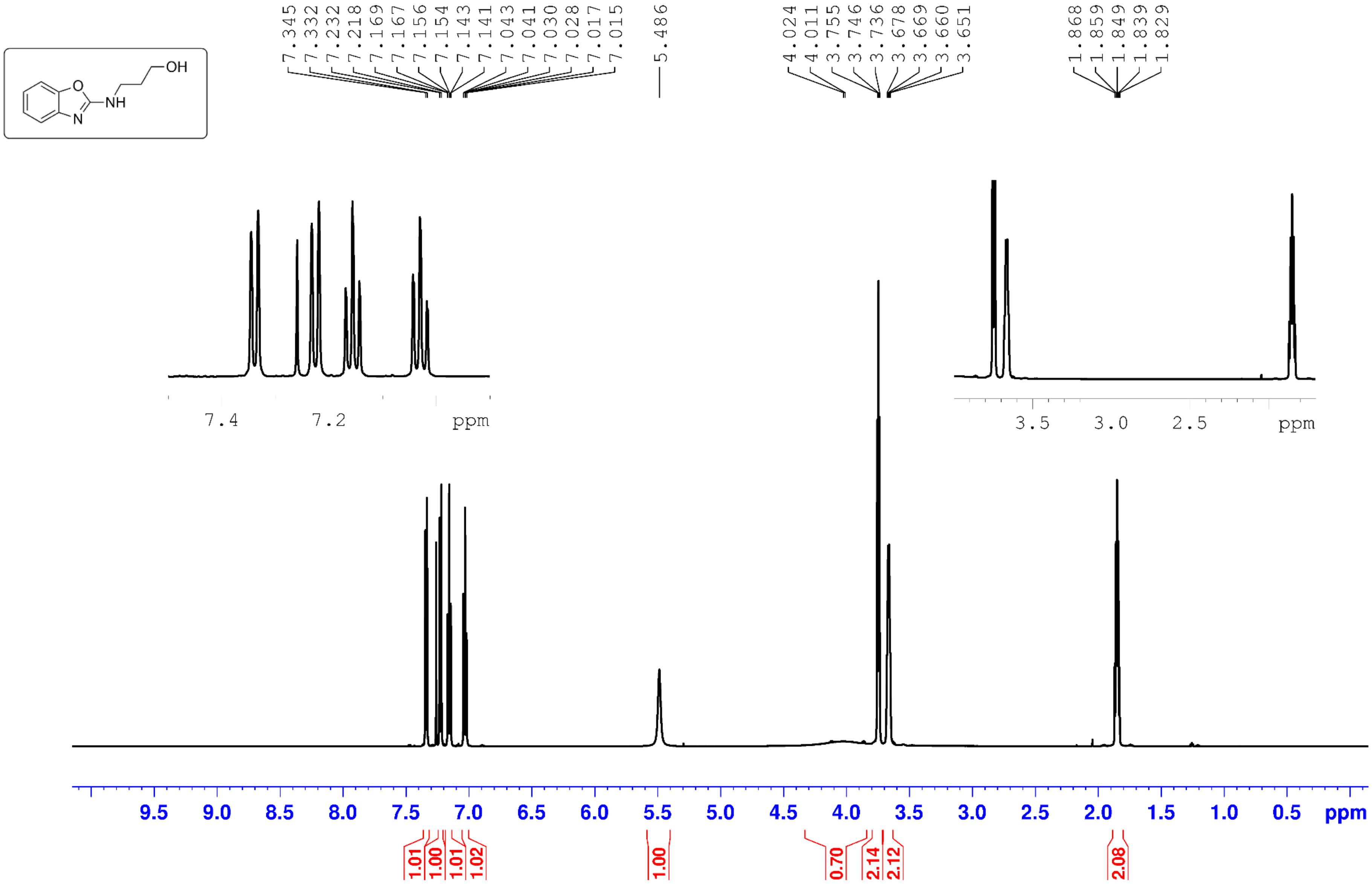

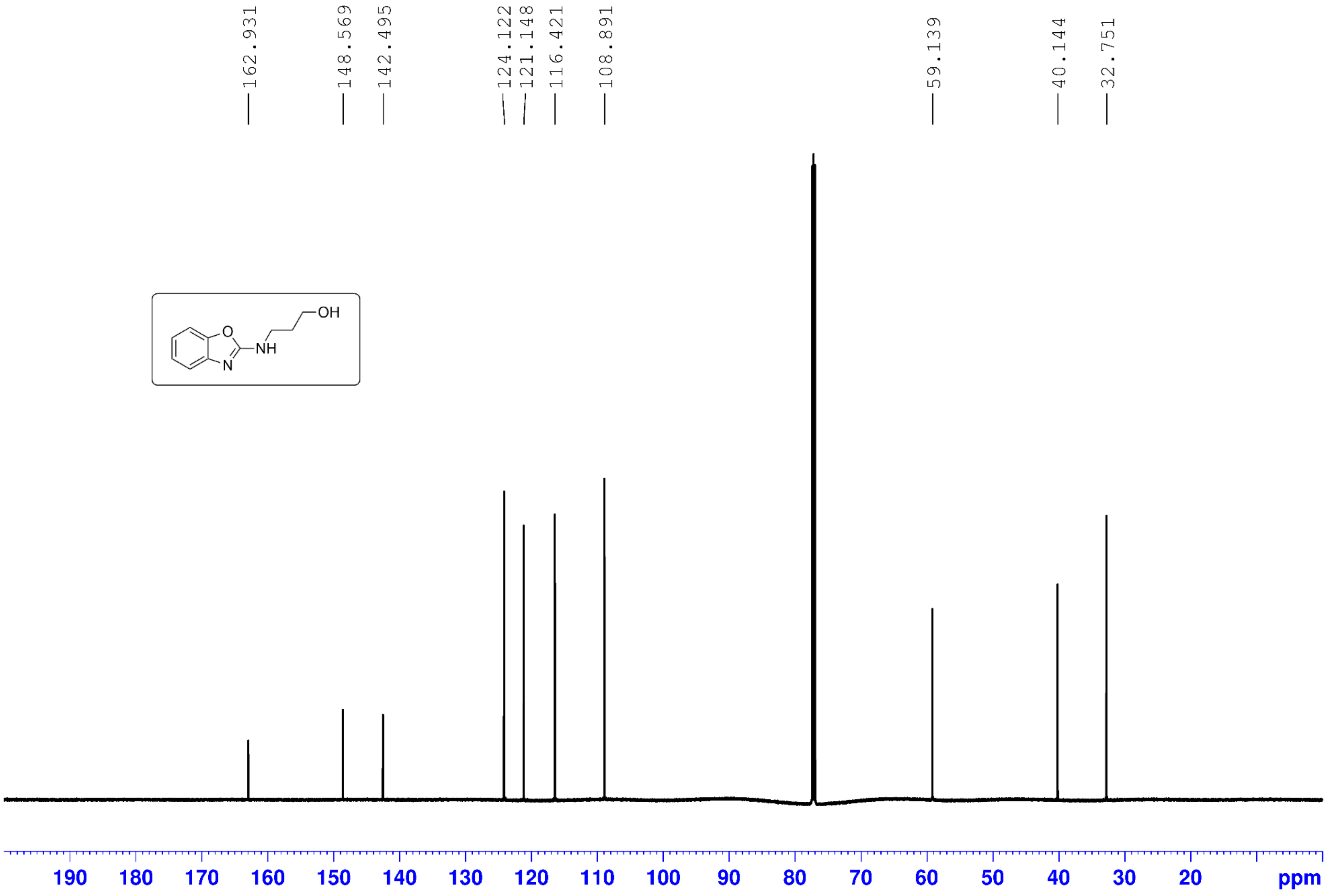
162.931
148.569
142.495
124.122
121.148
116.421
108.891
59.139
40.144
32.751
OH
O
NH
N
190
180
170
160
150
140
130
120
110
100
90
80
70
60
50
40
30
20
ppm

1,2,3,4-Tetrahydro-5l4-benzo[4,5]oxazolo[3,2-a]pyrimidine IU3 (ODHPB)

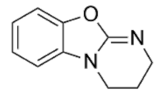

IU3

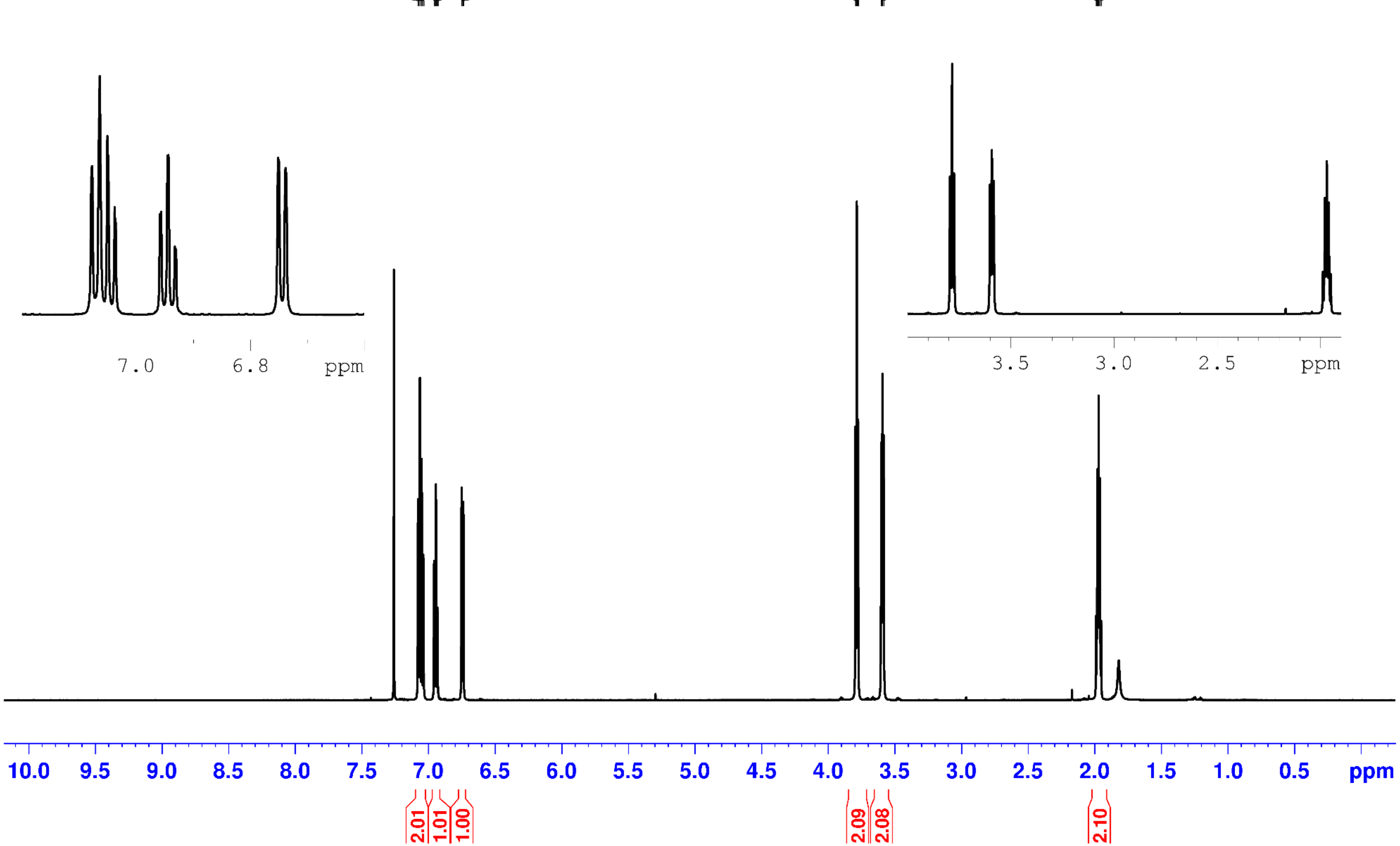

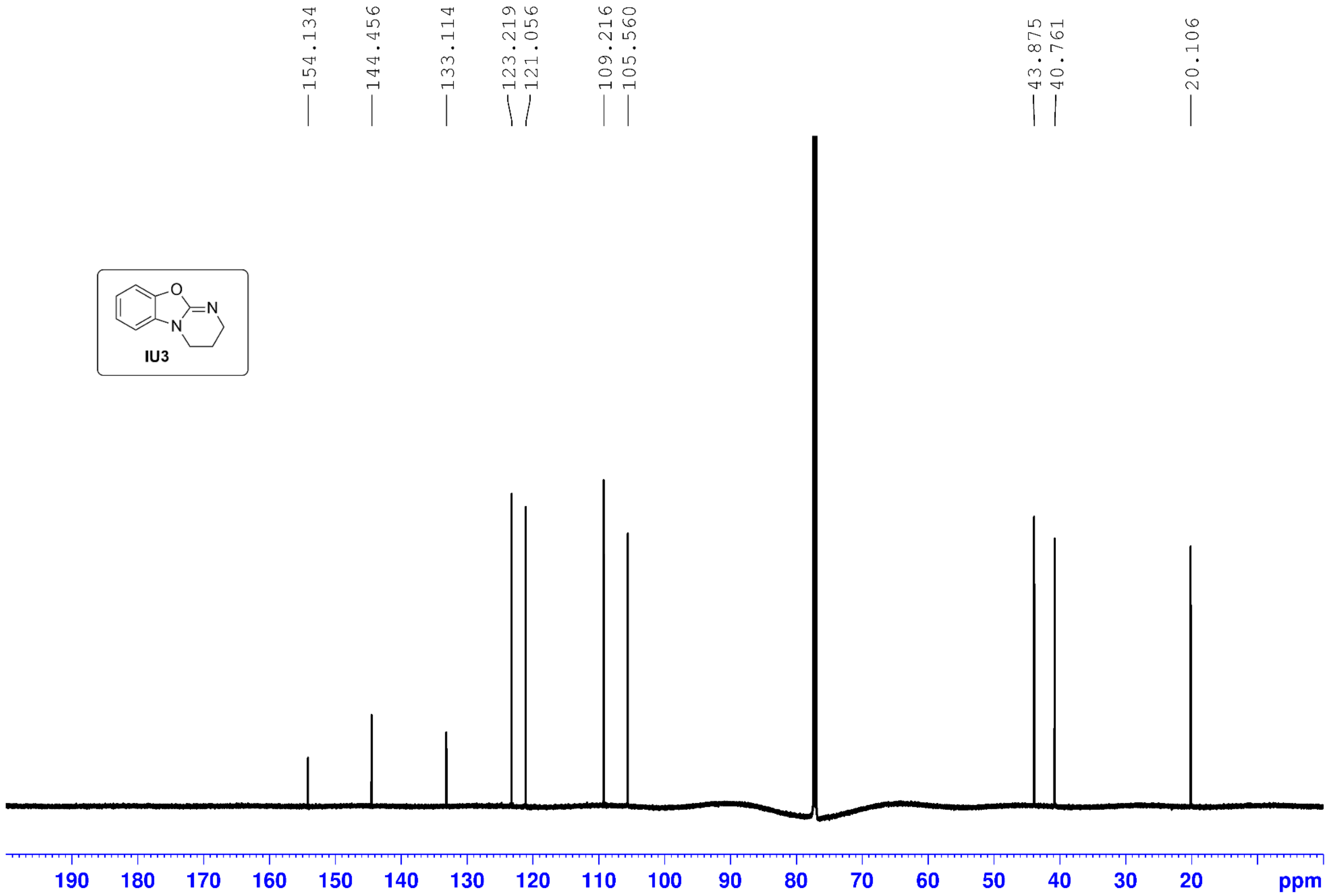

154.134
144.456
133.114
123.219
121.056
109.216
105.560
43.875
40.761
20.106
O
N
N
IU3
190
180
170
160
150
140
130
120
110
100
90
80
70
60
50
40
30
20
ppm

(*R*)-2-((6-Fluorobenzo[d]thiazol-2-yl)amino)-2-phenylethan-1-ol (3d)

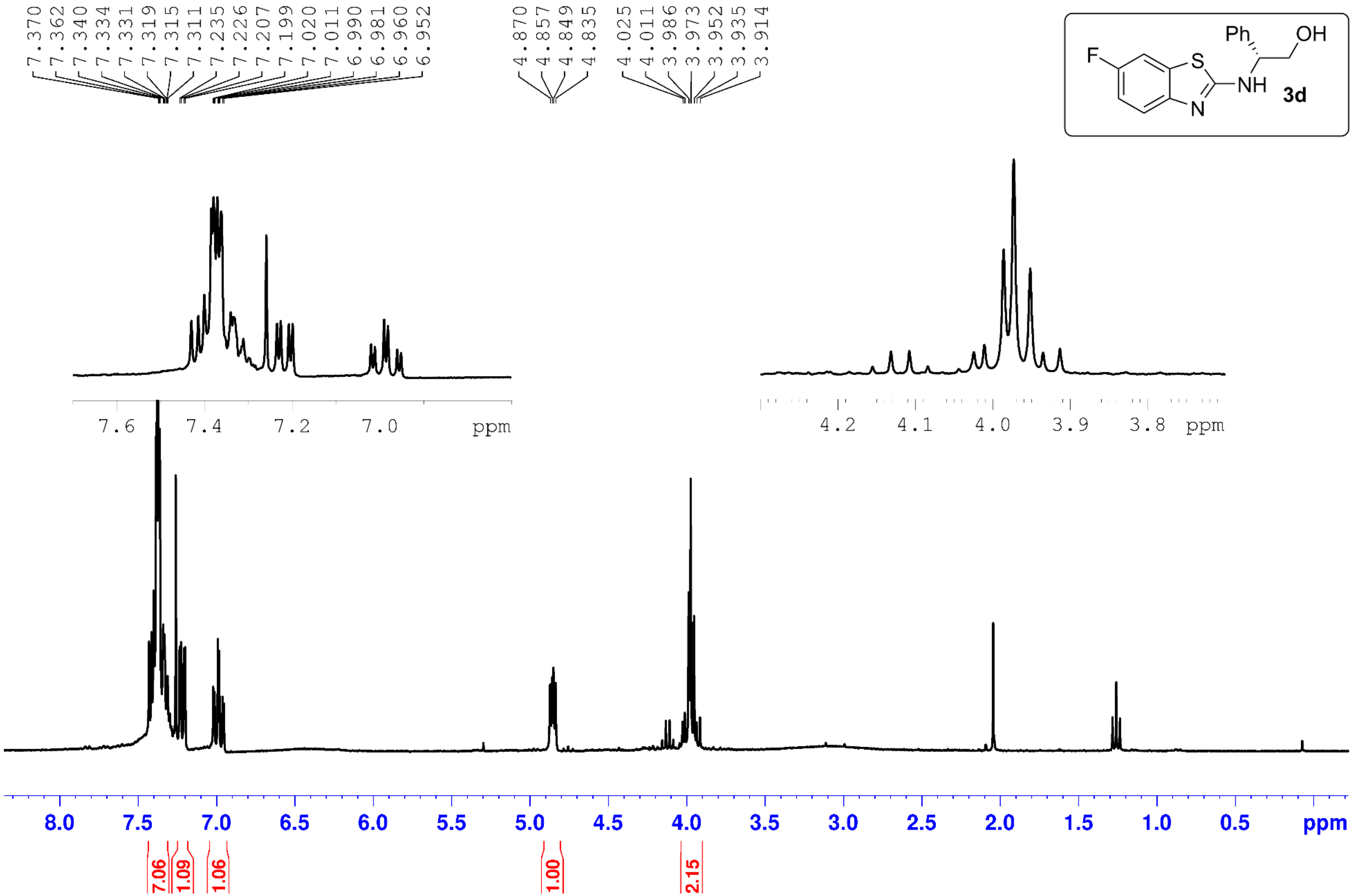

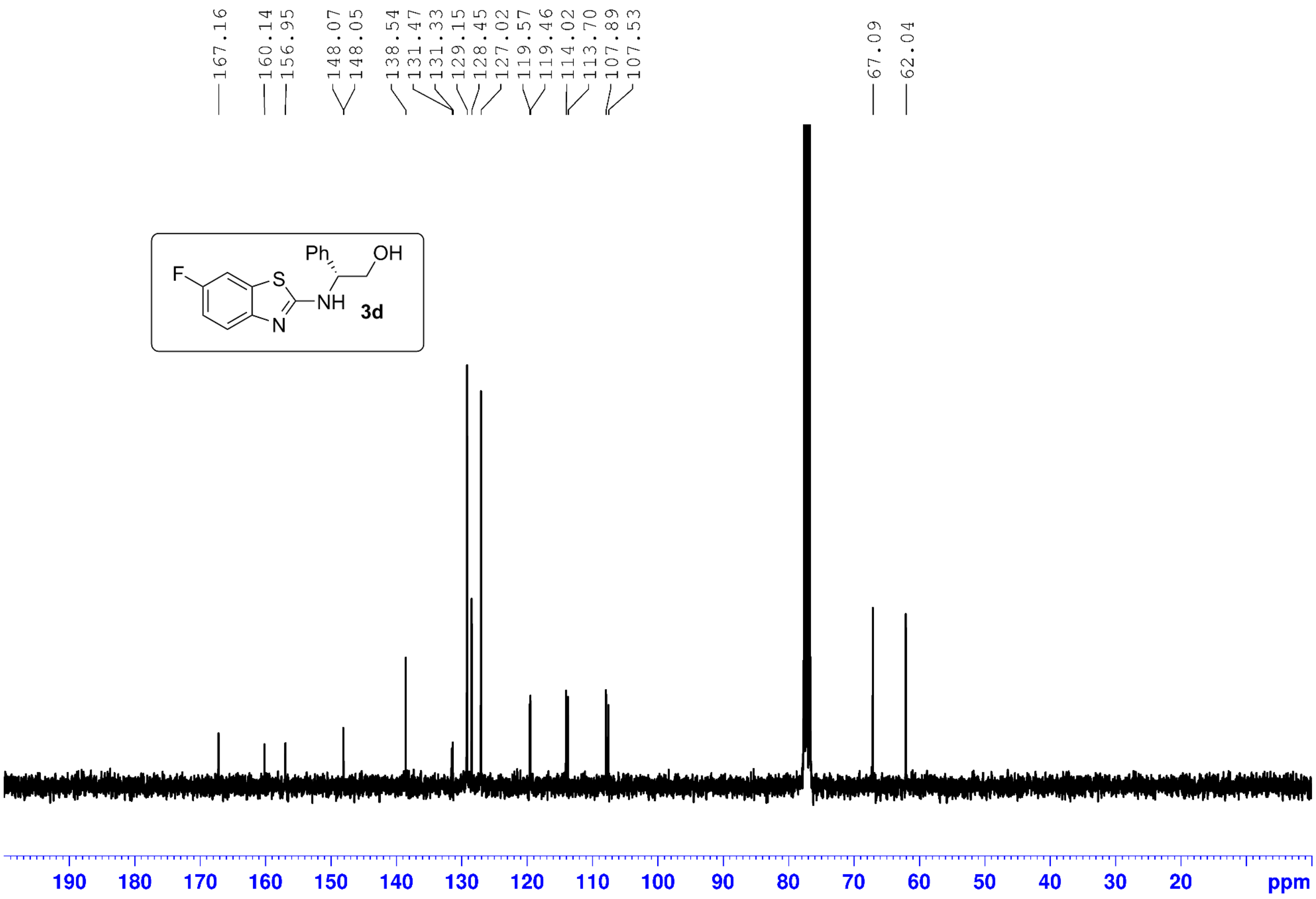
167.16
160.14
156.95
148.07
148.05
138.54
131.47
131.33
129.15
128.45
127.02
119.57
119.46
114.02
113.70
107.89
107.53
67.09
62.04
F
S
N
NH
Ph
OH
3d
190
180
170
160
150
140
130
120
110
100
90
80
70
60
50
40
30
20
ppm

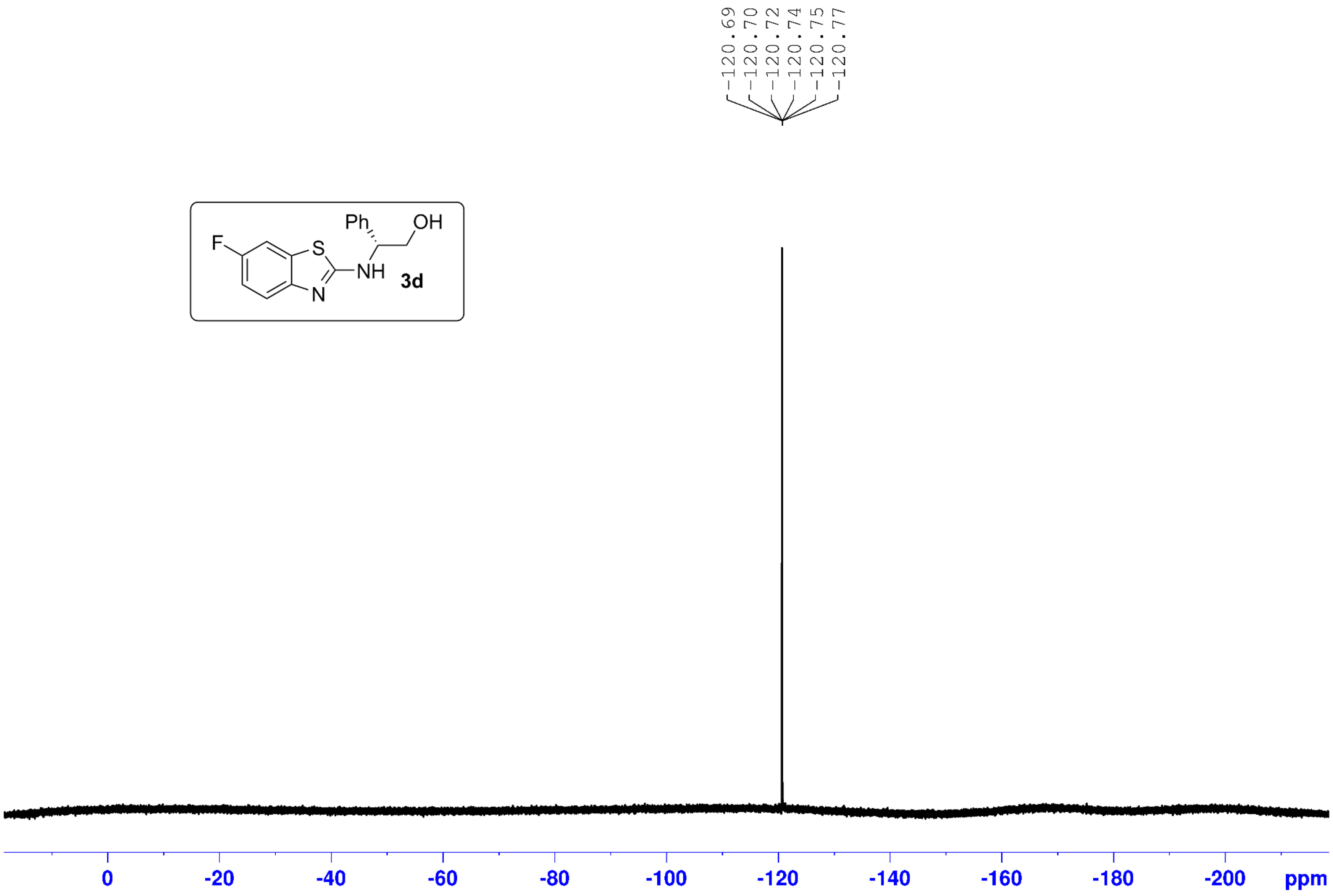
-120.69
-120.70
-120.72
-120.74
-120.75
-120.77
F
S
N
NH
Ph
OH
0
-20
-40
-60
-80
-100
-120
-140
-160
-180
-200
ppm

3d

7-F-BTM (F-ITU2)

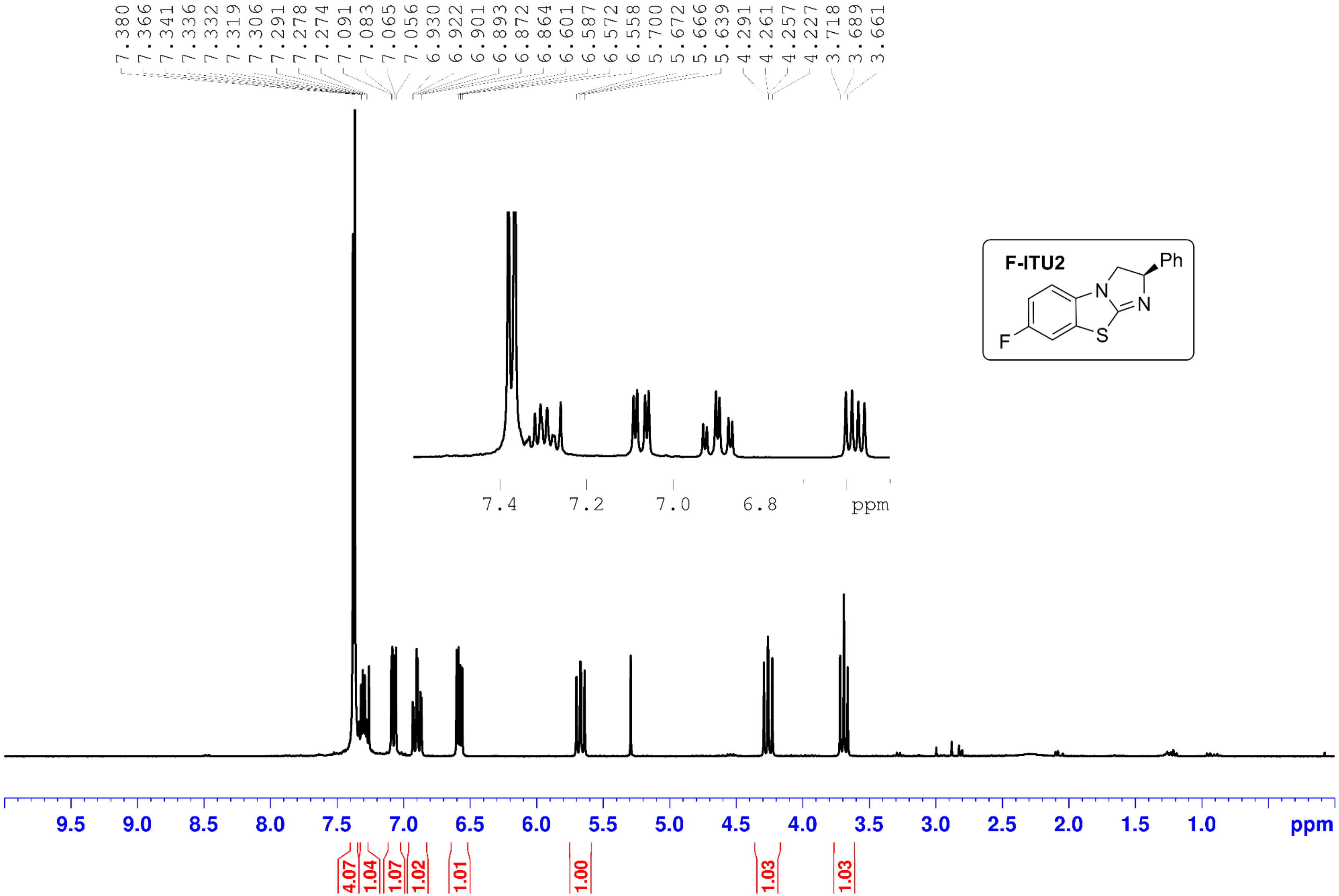
7.380
7.366
7.341
7.336
7.332
7.319
7.306
7.291
7.278
7.274
7.091
7.083
7.065
7.056
6.930
6.922
6.901
6.893
6.872
6.864
6.601
6.587
6.572
6.558
5.700
5.672
5.666
5.639
4.291
4.261
4.257
4.227
3.718
3.689
3.661
7.4
7.2
7.0
6.8
ppm
F-ITU2
Ph
N
N
S
F
9.5
9.0
8.5
8.0
7.5
7.0
6.5
6.0
5.5
5.0
4.5
4.0
3.5
3.0
2.5
2.0
1.5
1.0
ppm
4.07
1.04
1.07
1.02
1.01
1.00
1.03
1.03

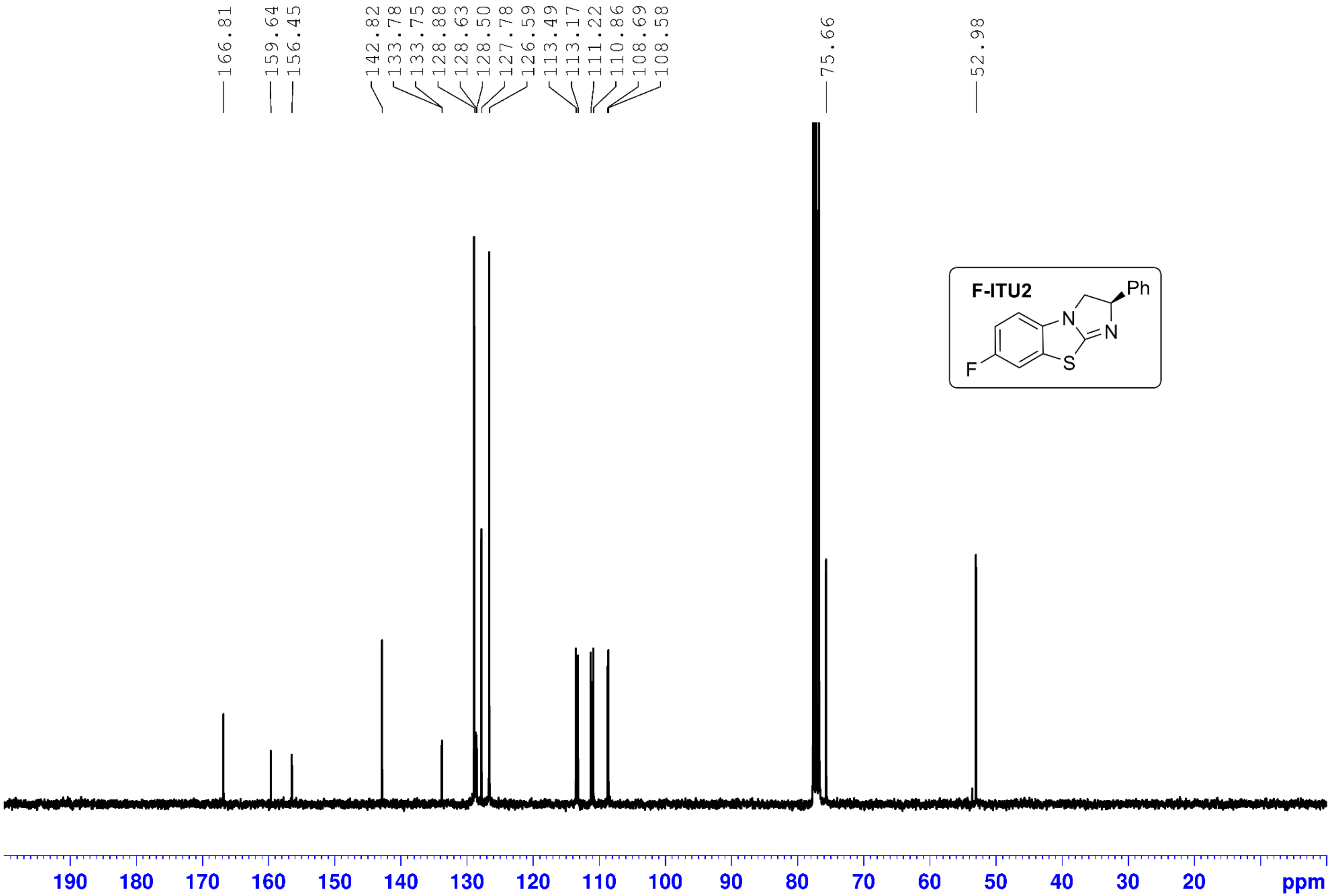
166.81
159.64
156.45
142.82
133.78
133.75
128.88
128.63
128.50
127.78
126.59
113.49
113.17
111.22
110.86
108.69
108.58
75.66
52.98
F-ITU2
Ph
N
N
S
F
190 180 170 160 150 140 130 120 110 100 90 80 70 60 50 40 30 20 ppm

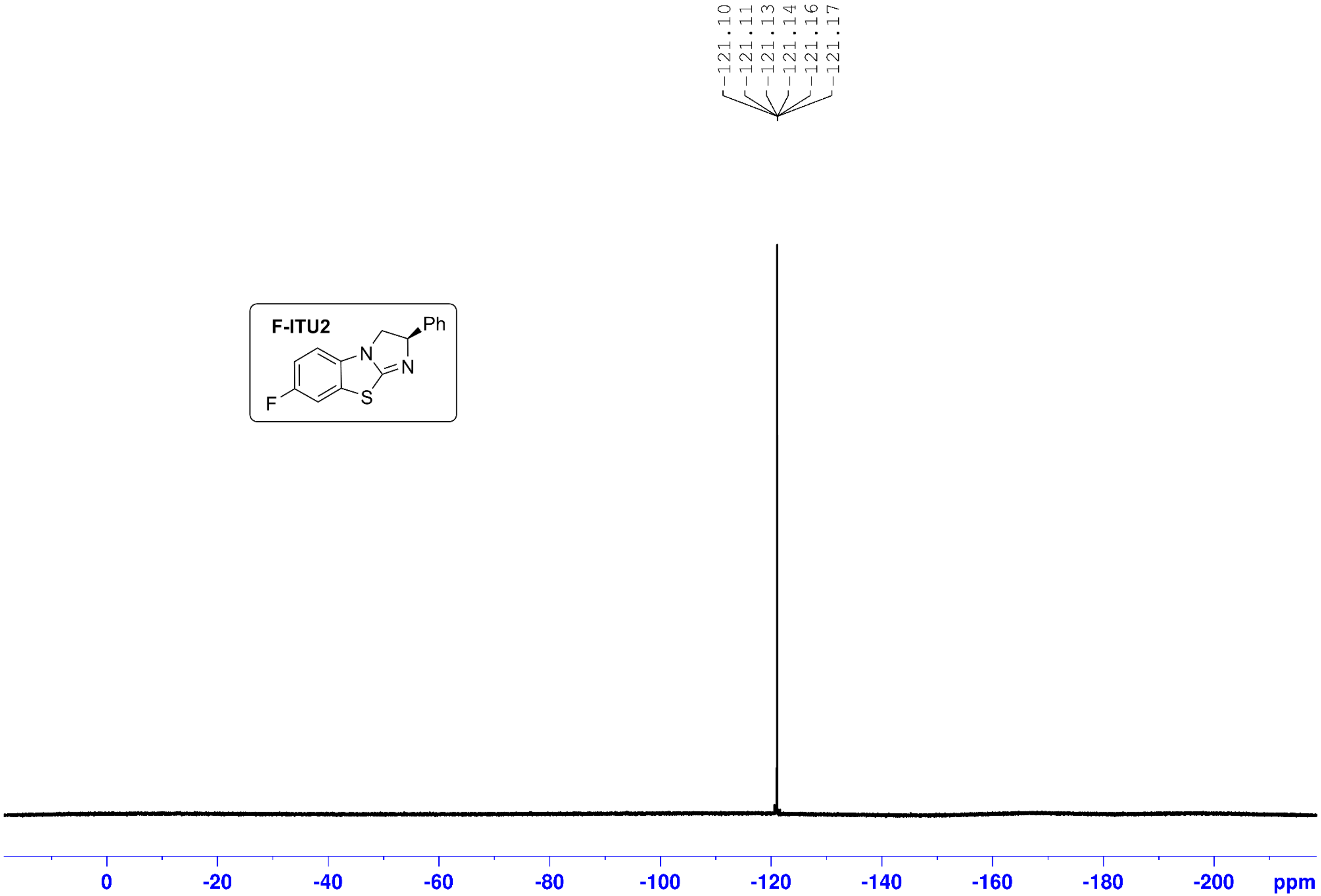
-121.10
-121.11
-121.13
-121.14
-121.16
-121.17
F-ITU2
Ph
N
N
S
F
0
-20
-40
-60
-80
-100
-120
-140
-160
-180
-200
ppm

^{77}Se-NMR for Se*Hyper*BTM (ISeU5)

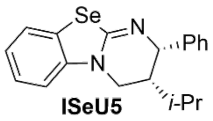

ISeU5

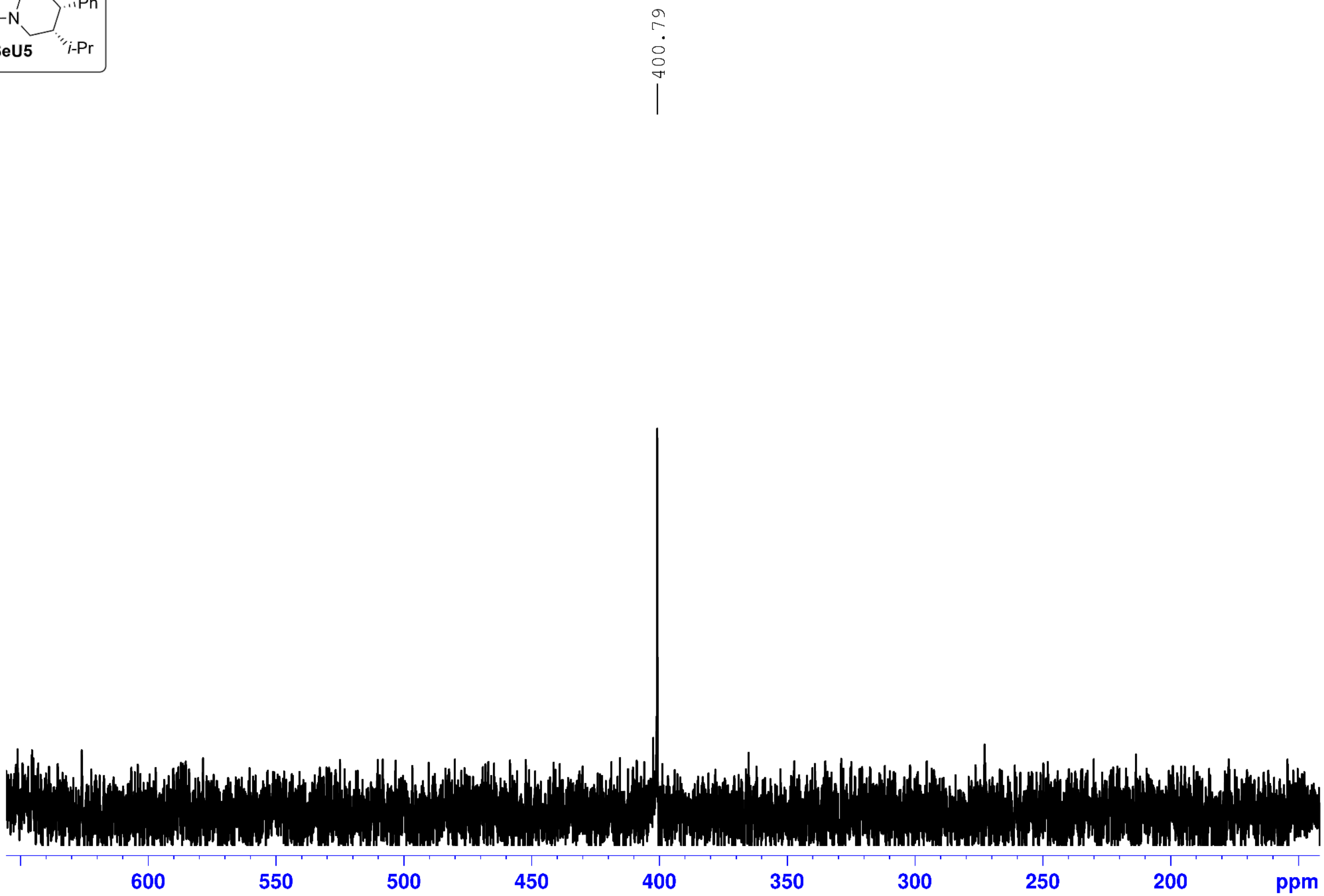

SeHBTM (ISeU4)

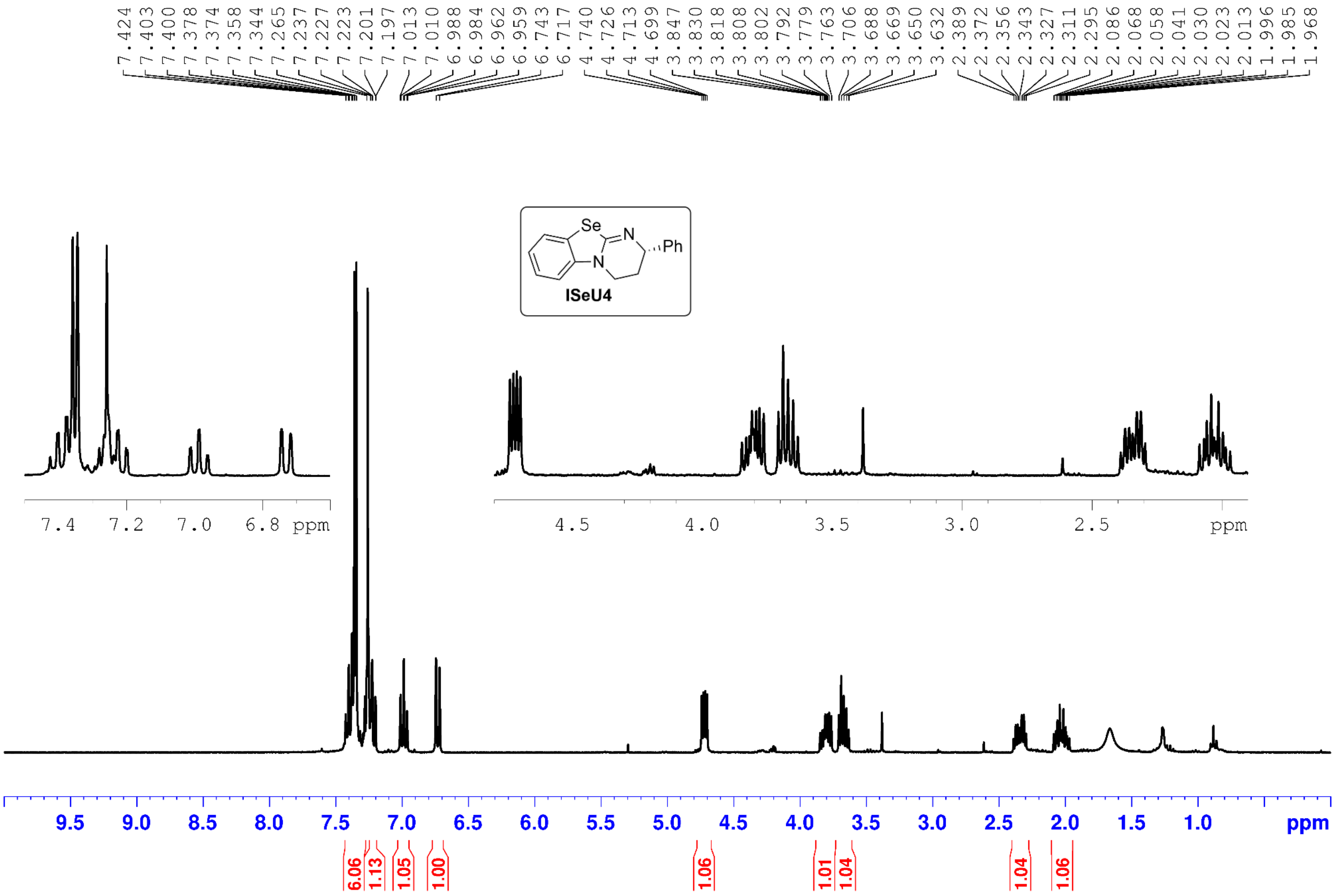

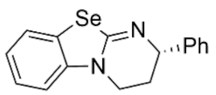
Se
N
Ph
N

ISeU4

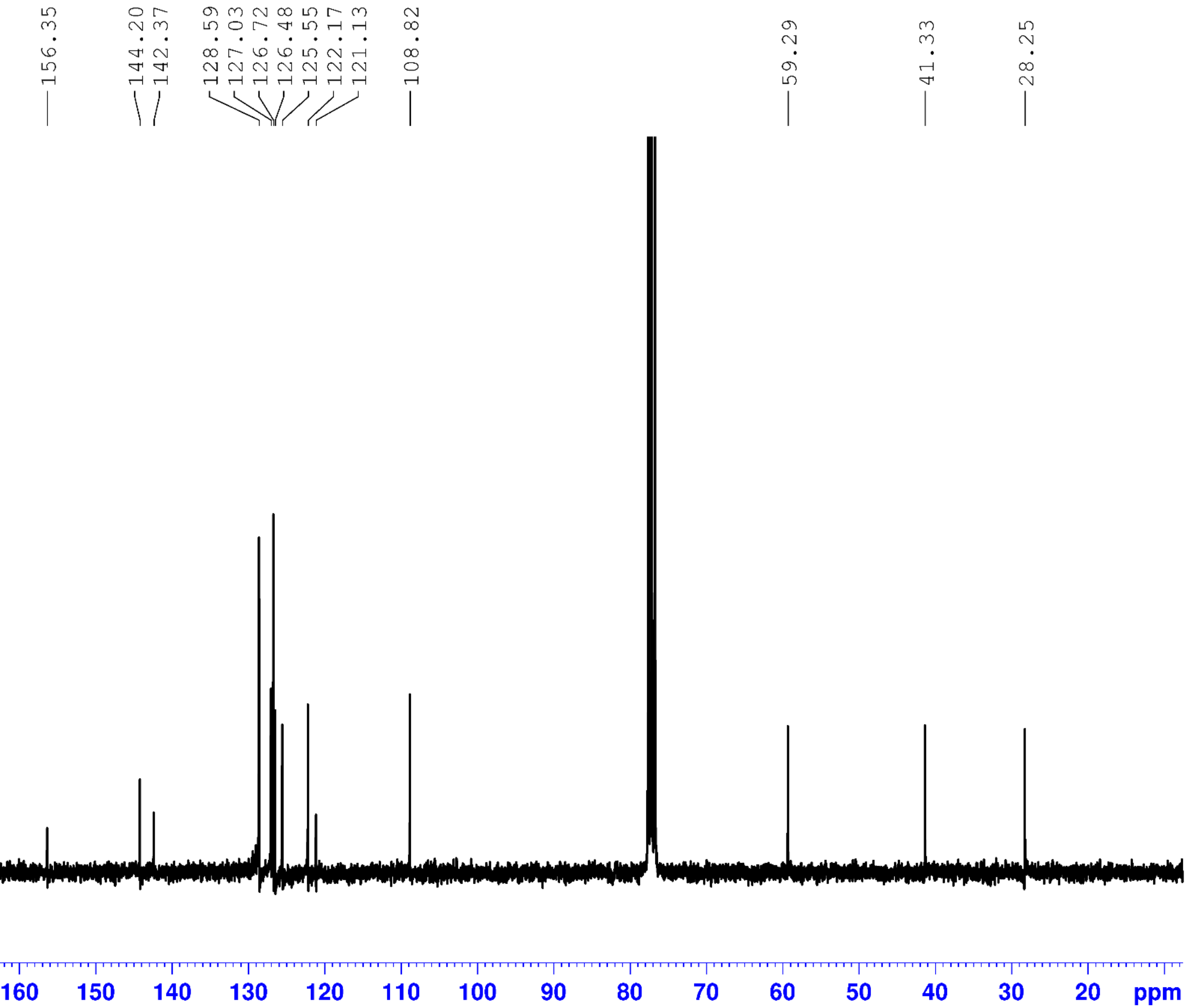
156.35
144.20
142.37
128.59
127.03
126.72
126.48
125.55
122.17
121.13
108.82
59.29
41.33
28.25
190 180 170 160 150 140 130 120 110 100 90 80 70 60 50 40 30 20 ppm

SeBTM (ISeU2)

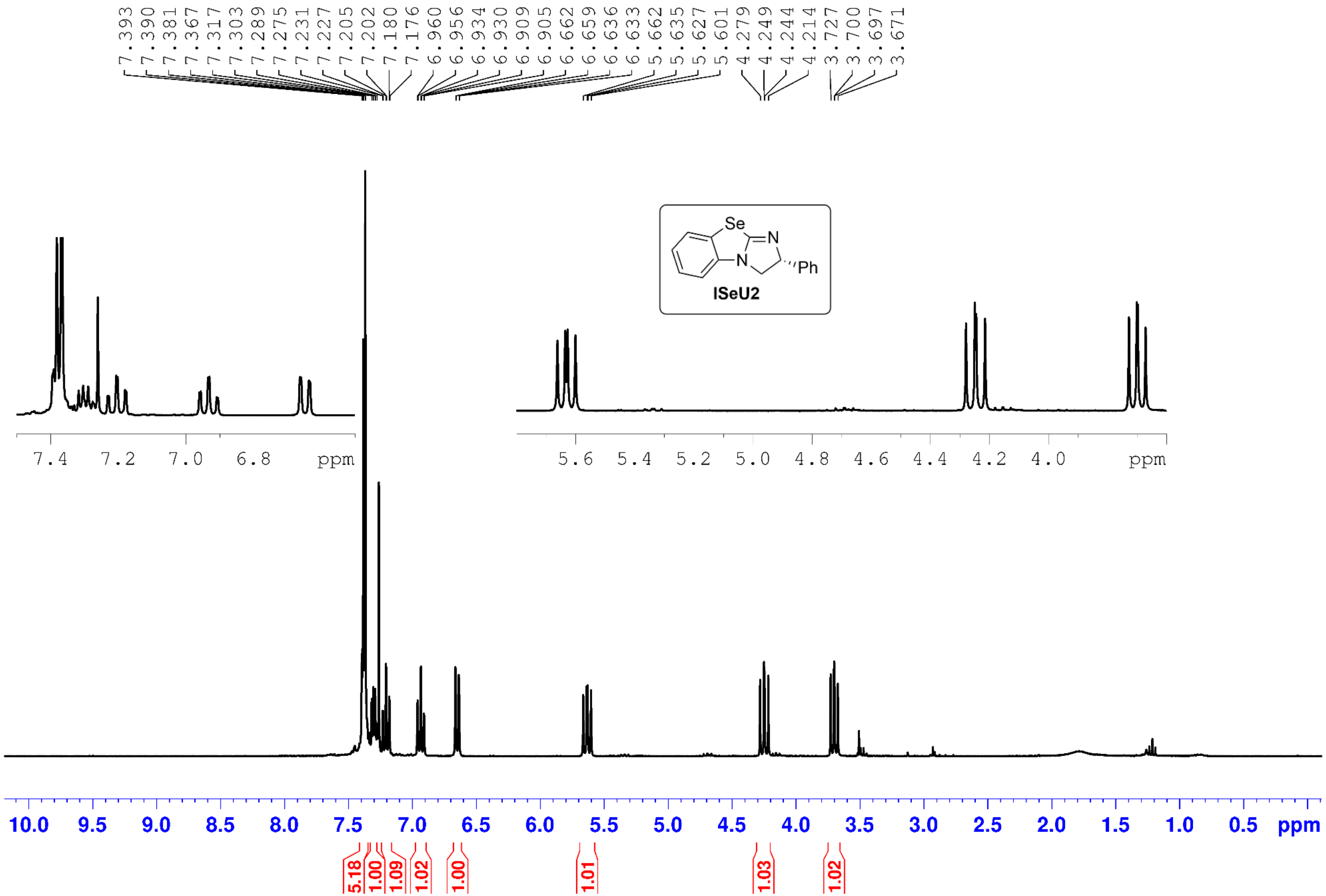
7.393
7.390
7.381
7.367
7.317
7.303
7.289
7.275
7.231
7.227
7.205
7.202
7.180
7.176
6.960
6.956
6.934
6.930
6.909
6.905
6.662
6.659
6.636
6.633
5.662
5.635
5.627
5.601
4.279
4.249
4.244
4.214
3.727
3.700
3.697
3.671
Se
N
N
Ph
ISeU2
7.4 7.2 7.0 6.8 ppm
5.6 5.4 5.2 5.0 4.8 4.6 4.4 4.2 4.0 ppm
10.0 9.5 9.0 8.5 8.0 7.5 7.0 6.5 6.0 5.5 5.0 4.5 4.0 3.5 3.0 2.5 2.0 1.5 1.0 0.5 ppm
5.18
1.00
1.09
1.02
1.00
1.01
1.03
1.02

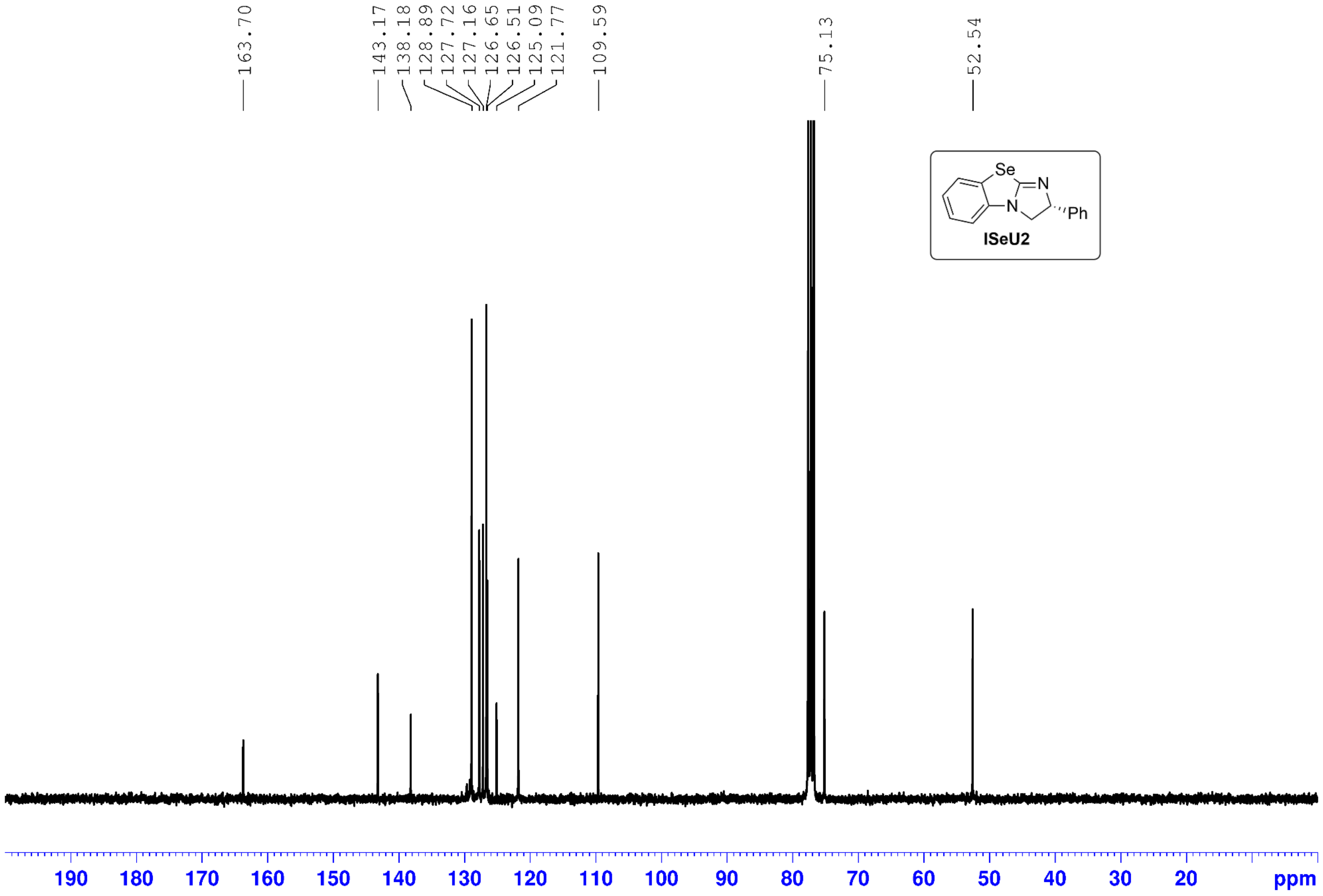
163.70
143.17
138.18
128.89
127.72
127.16
126.65
126.51
125.09
121.77
109.59
75.13
52.54
Se
N
N
Ph
ISeU2
190
180
170
160
150
140
130
120
110
100
90
80
70
60
50
40
30
20
ppm

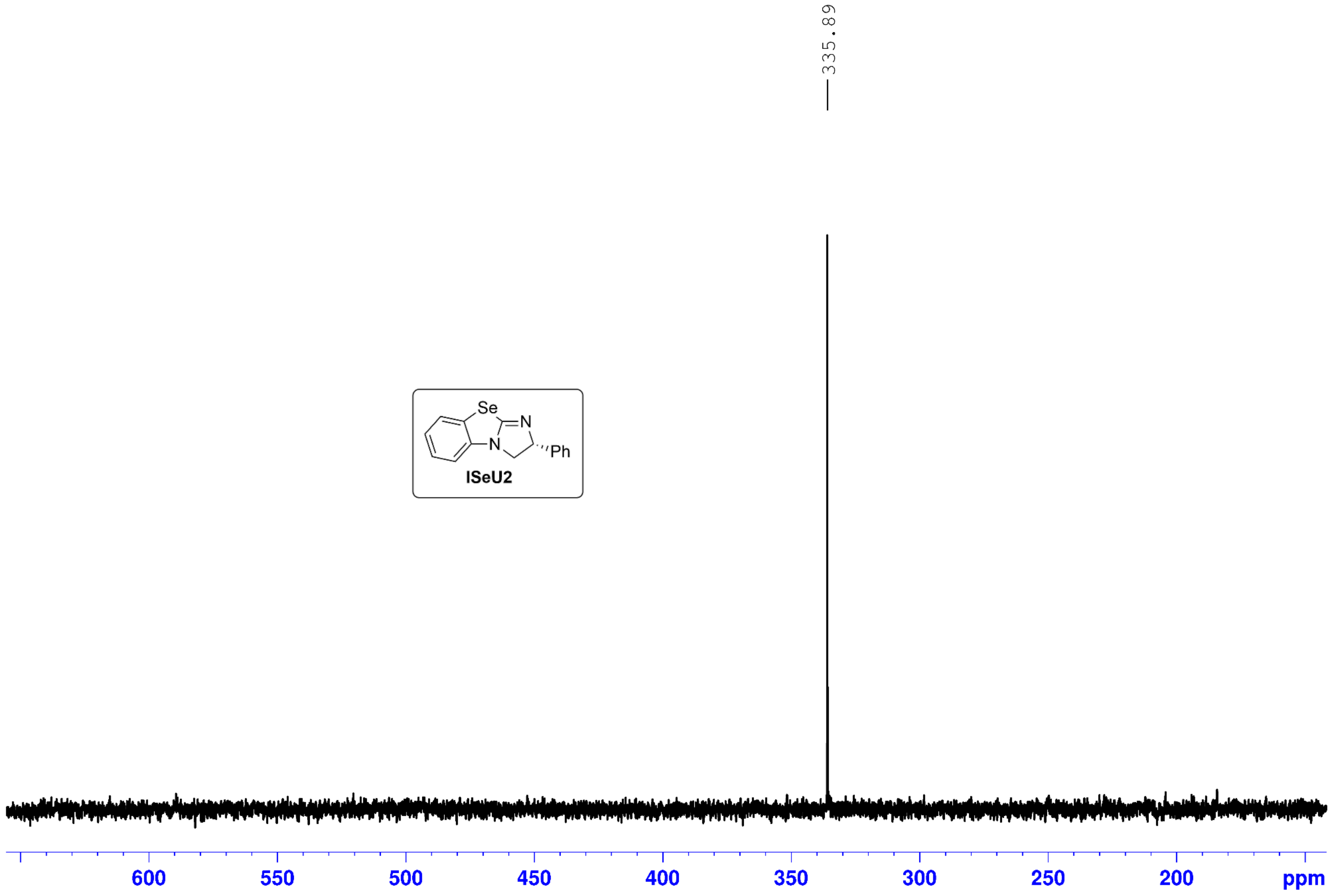
335.89
Se
N
N
Ph
ISeU2
600
550
500
450
400
350
300
250
200
ppm

SeDHPB (ISeU3)

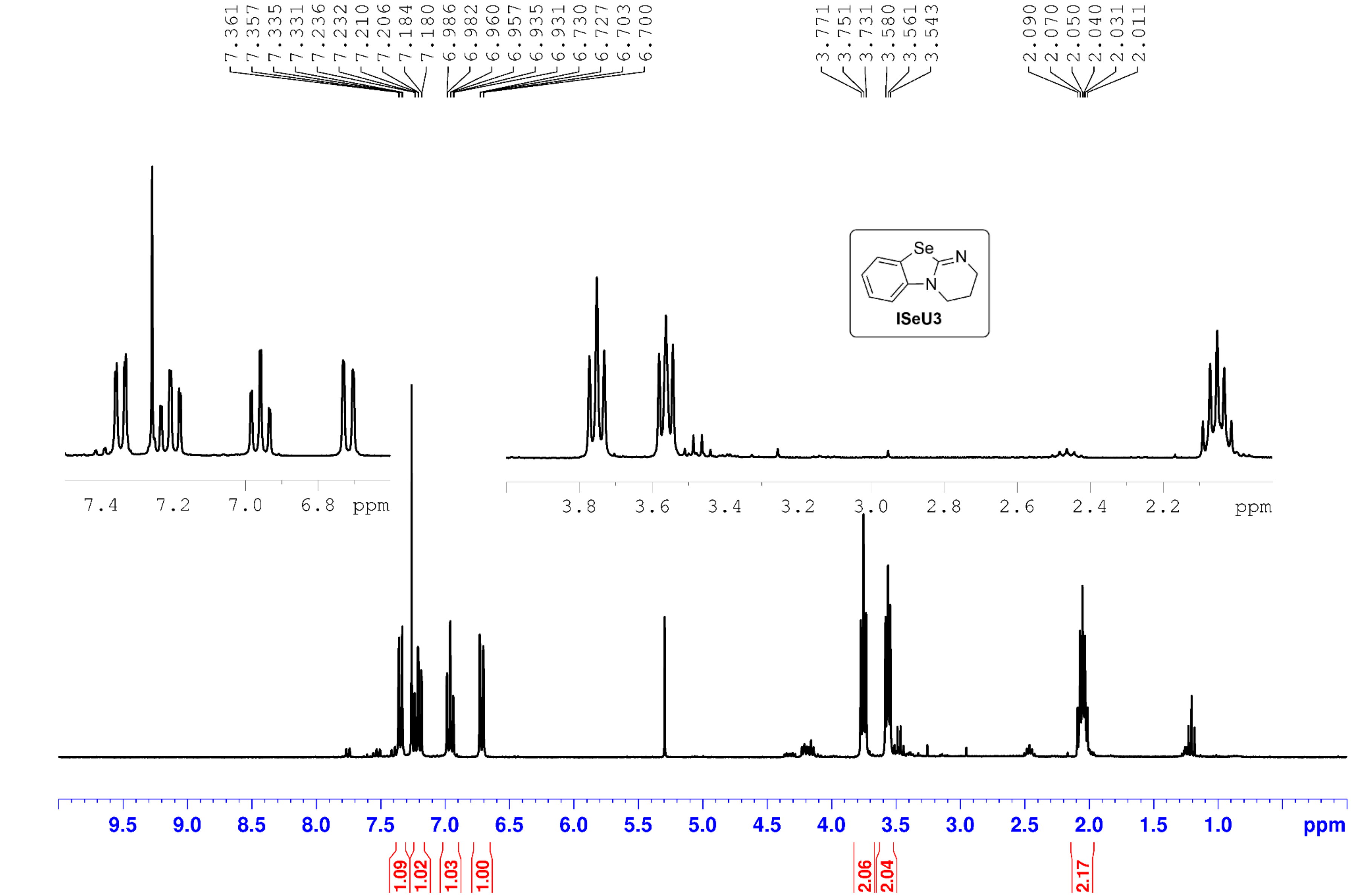

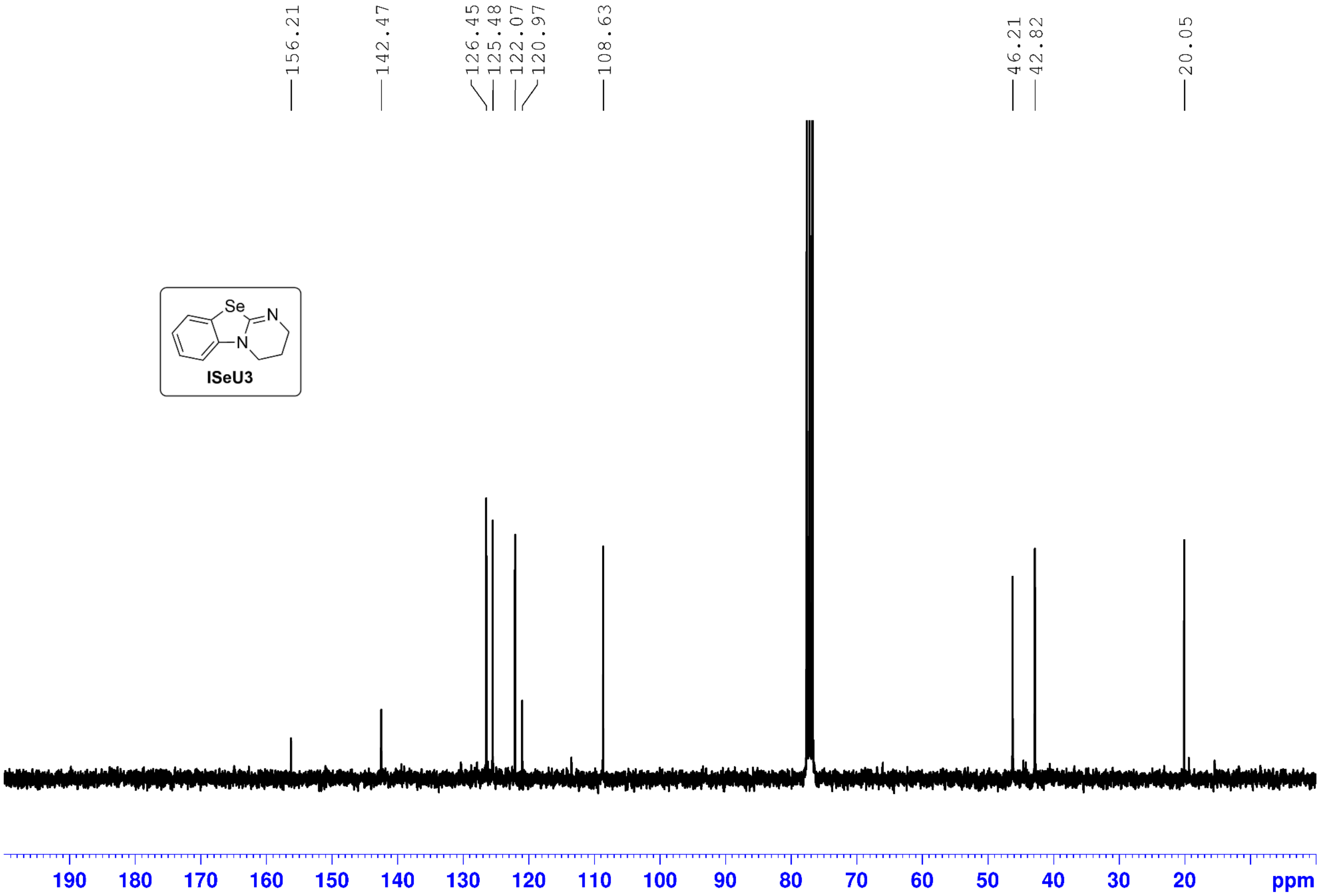
156.21
142.47
126.45
125.48
122.07
120.97
108.63
46.21
42.82
20.05
Se
N
N
ISeU3
190
180
170
160
150
140
130
120
110
100
90
80
70
60
50
40
30
20
ppm

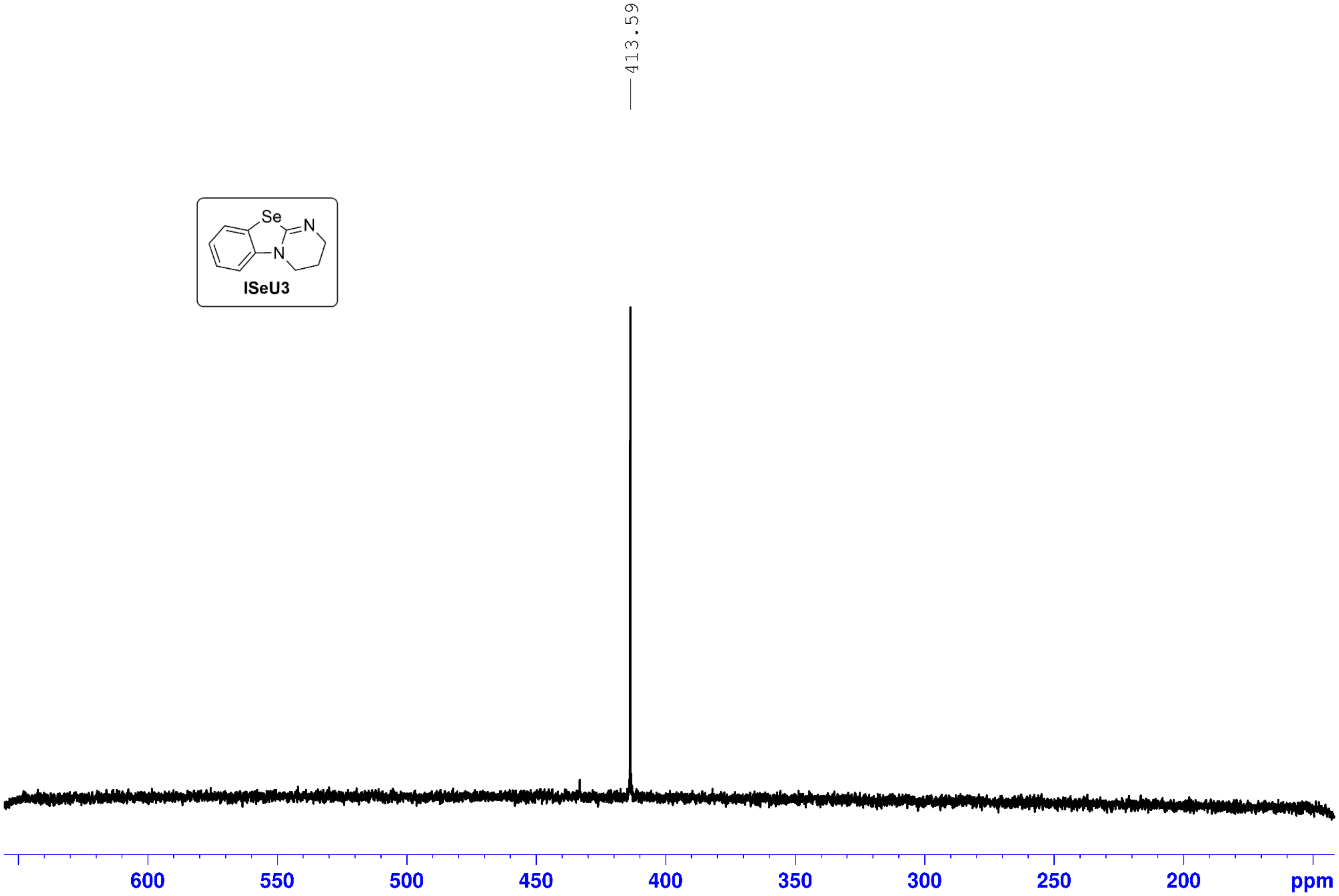

413.59
Se
N
N
ISeU3
600
550
500
450
400
350
300
250
200
ppm

4,5-Dihydro-1,3-selenazol-2-amine (10)

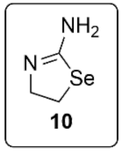

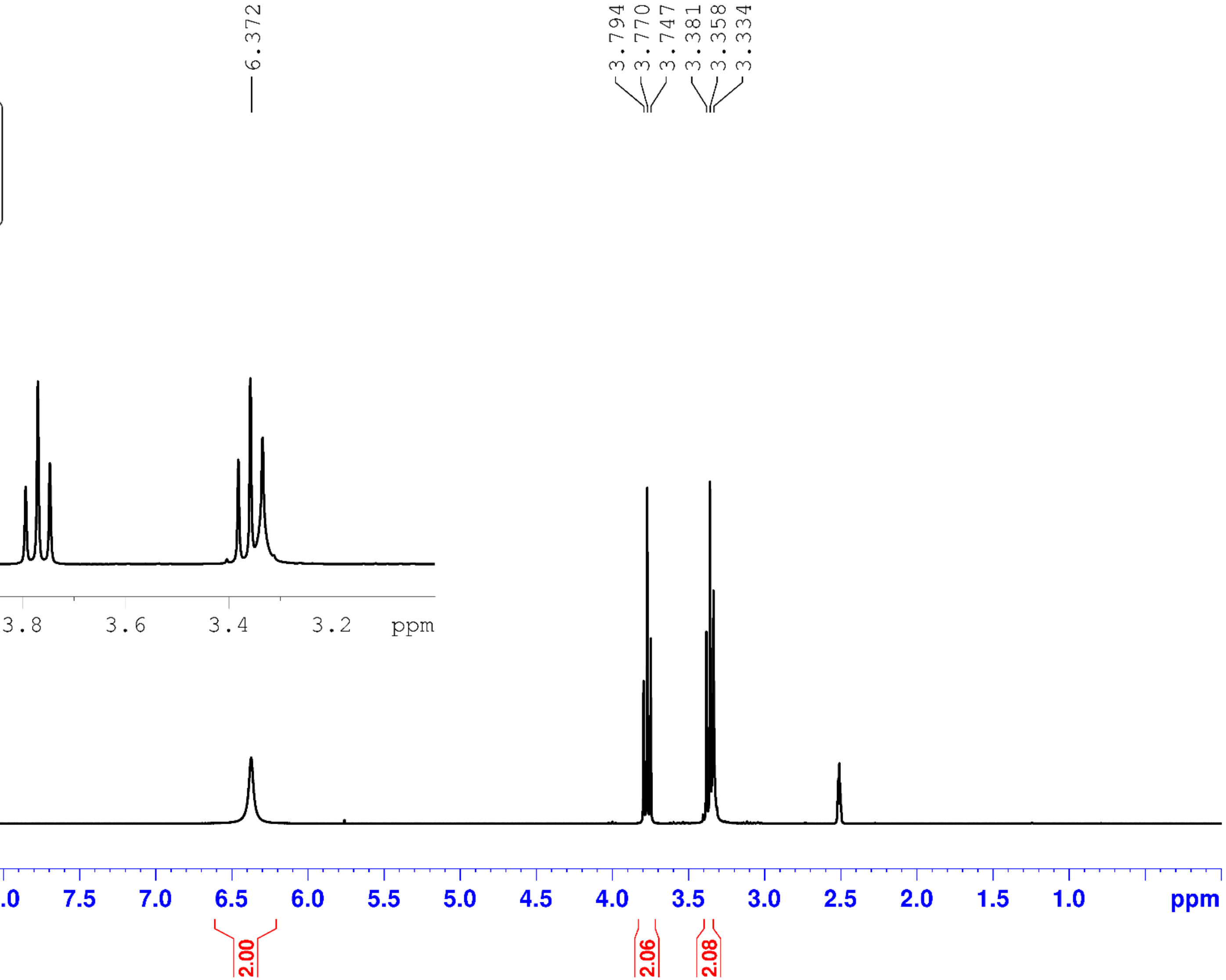

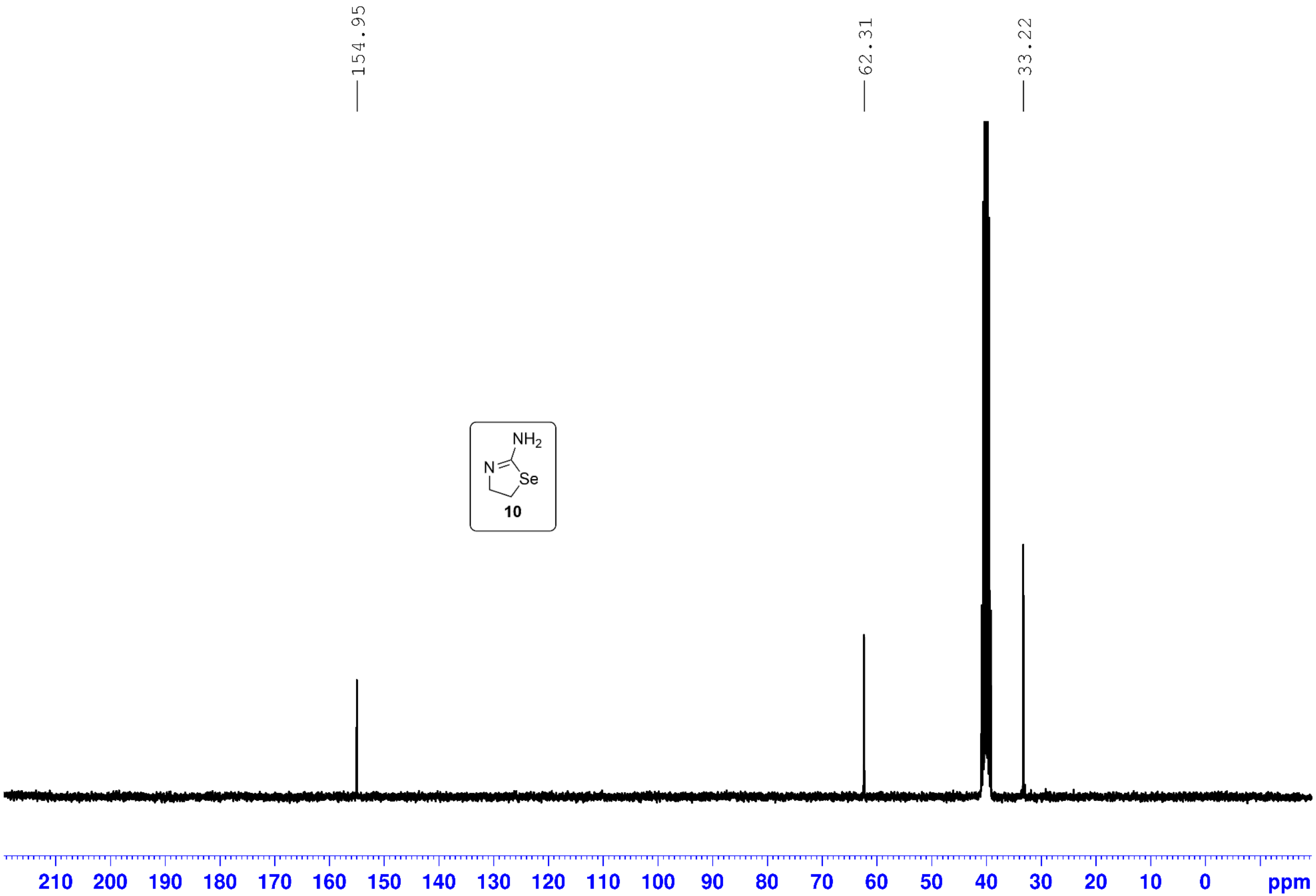

154.95
62.31
33.22
NH2
N
Se
10
210 200 190 180 170 160 150 140 130 120 110 100 90 80 70 60 50 40 30 20 10 0 ppm

3-(2-oxo-2-phenylethyl)-1,3-selenazolidin-2-iminium bromide (12)

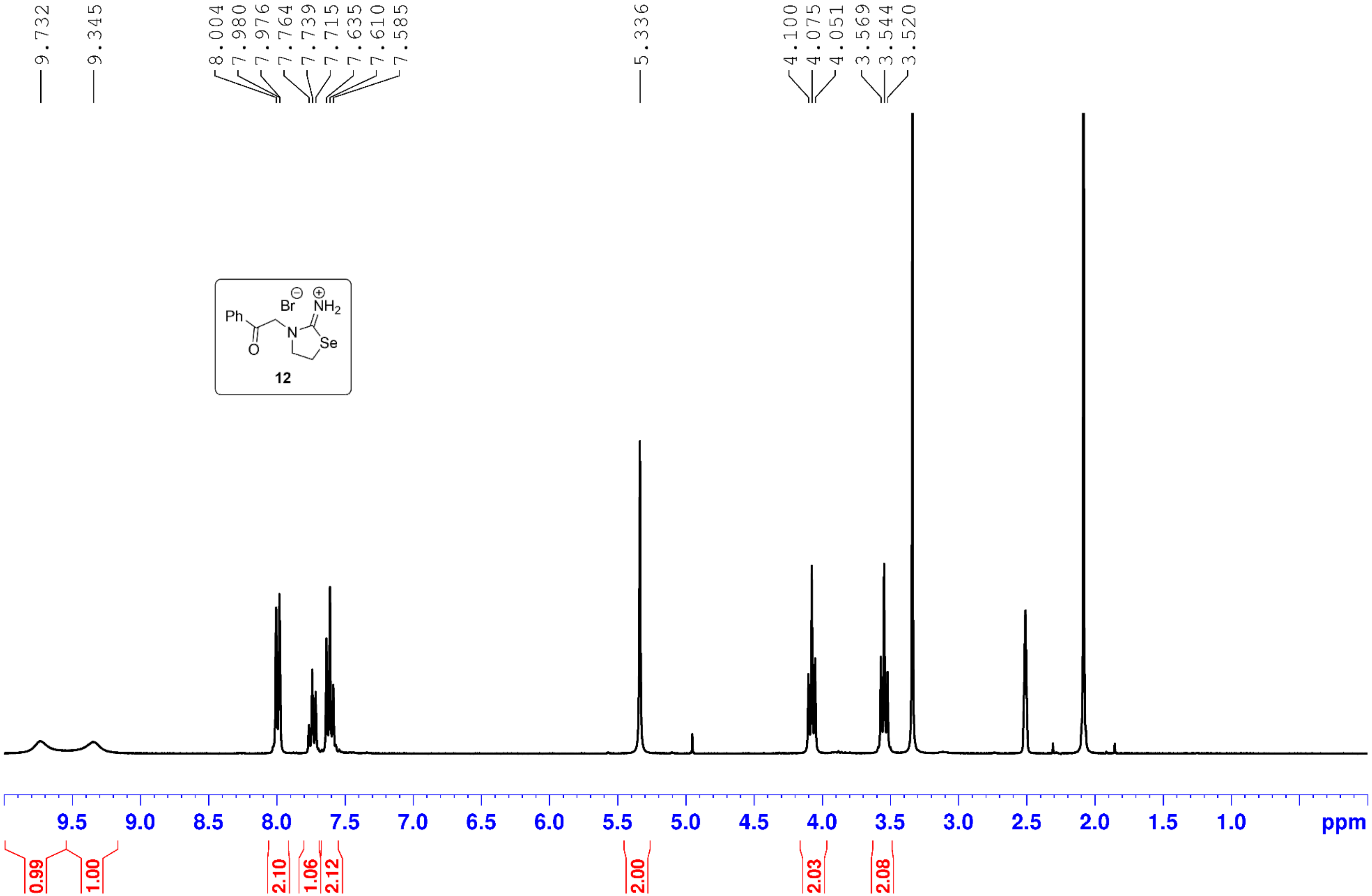

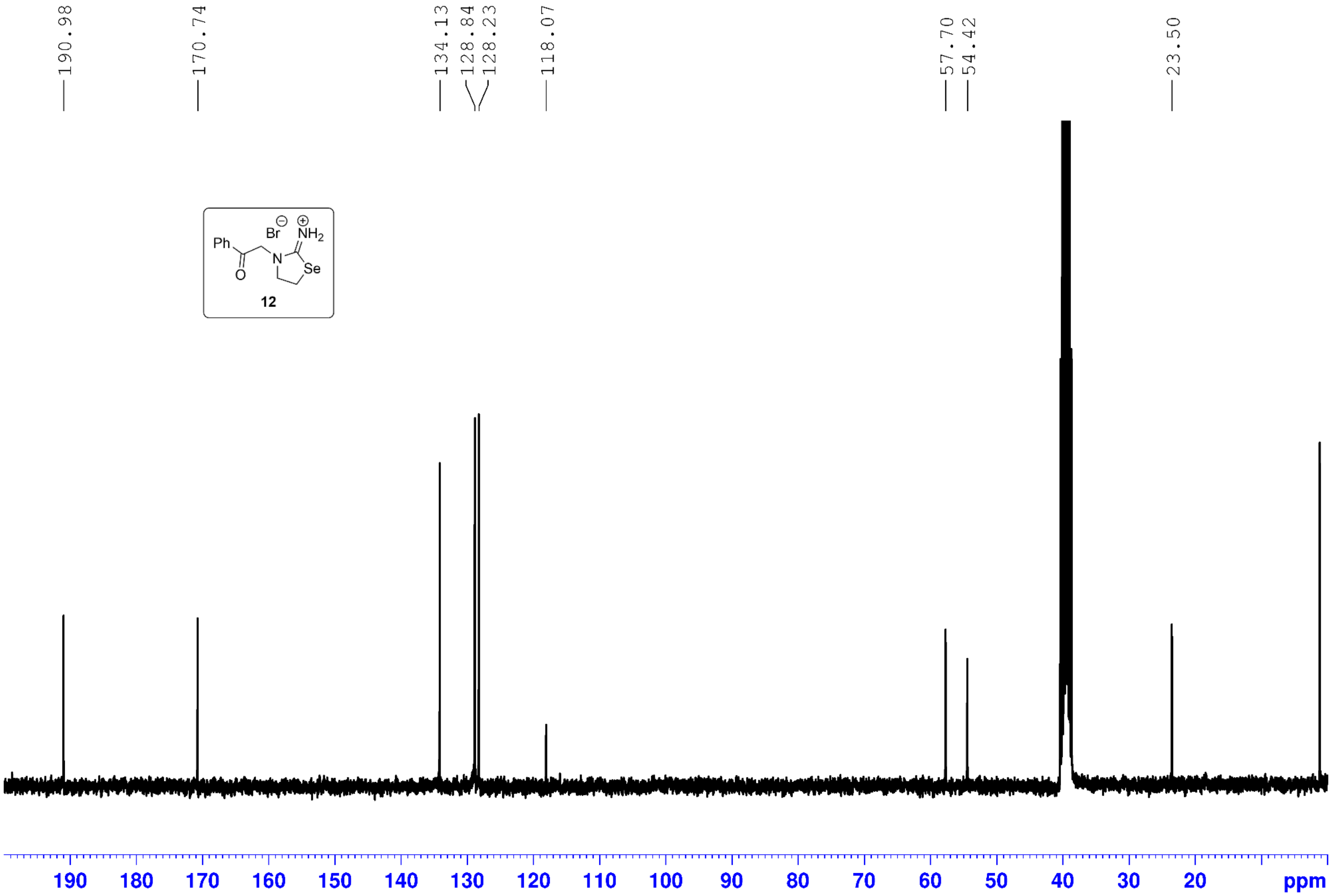
190.98
170.74
134.13
128.84
128.23
118.07
57.70
54.42
23.50
Ph
Br
NH2
N
Se
O
12
190
180
170
160
150
140
130
120
110
100
90
80
70
60
50
40
30
20
ppm

3-(2-Hydroxy-2-phenylethyl)-1,3-selenazolidin-2-iminium bromide (13)

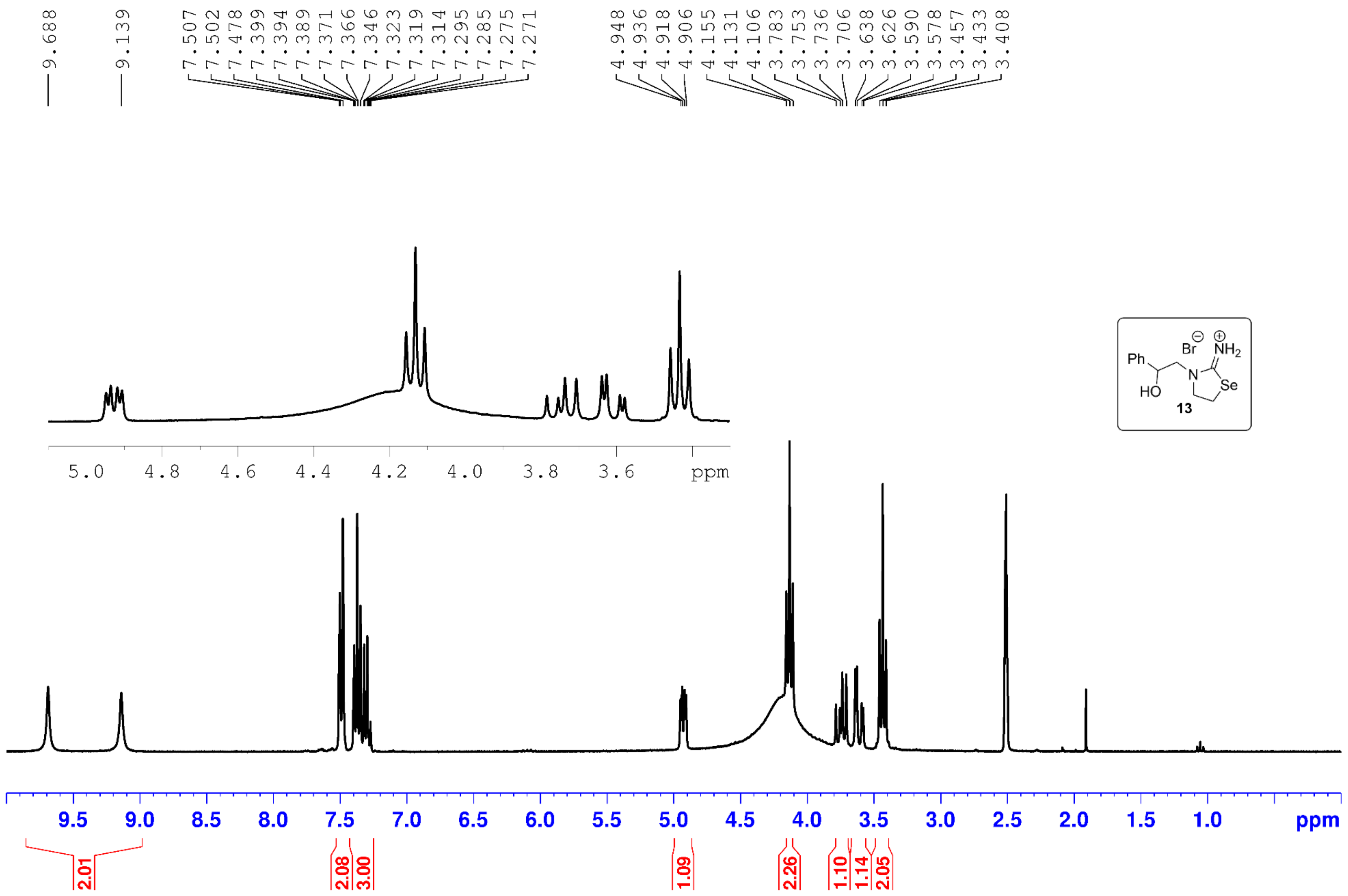

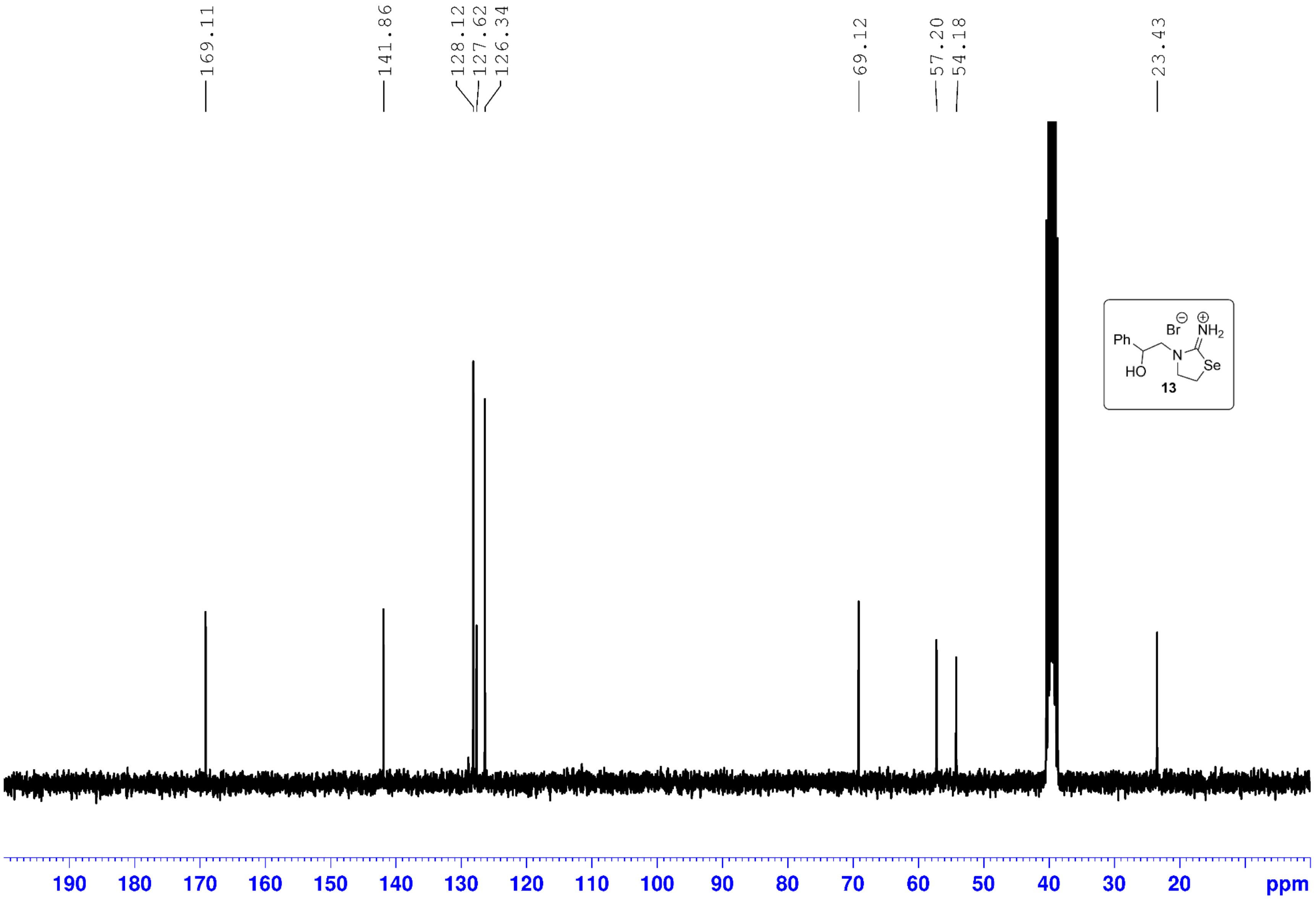
169.11
141.86
128.12
127.62
126.34
69.12
57.20
54.18
23.43
Ph
HO
Br
NH2
N
Se
13
190
180
170
160
150
140
130
120
110
100
90
80
70
60
50
40
30
20
ppm

SeTM (ISeU1)

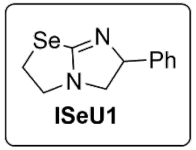
Se
N
N
Ph
ISeU1

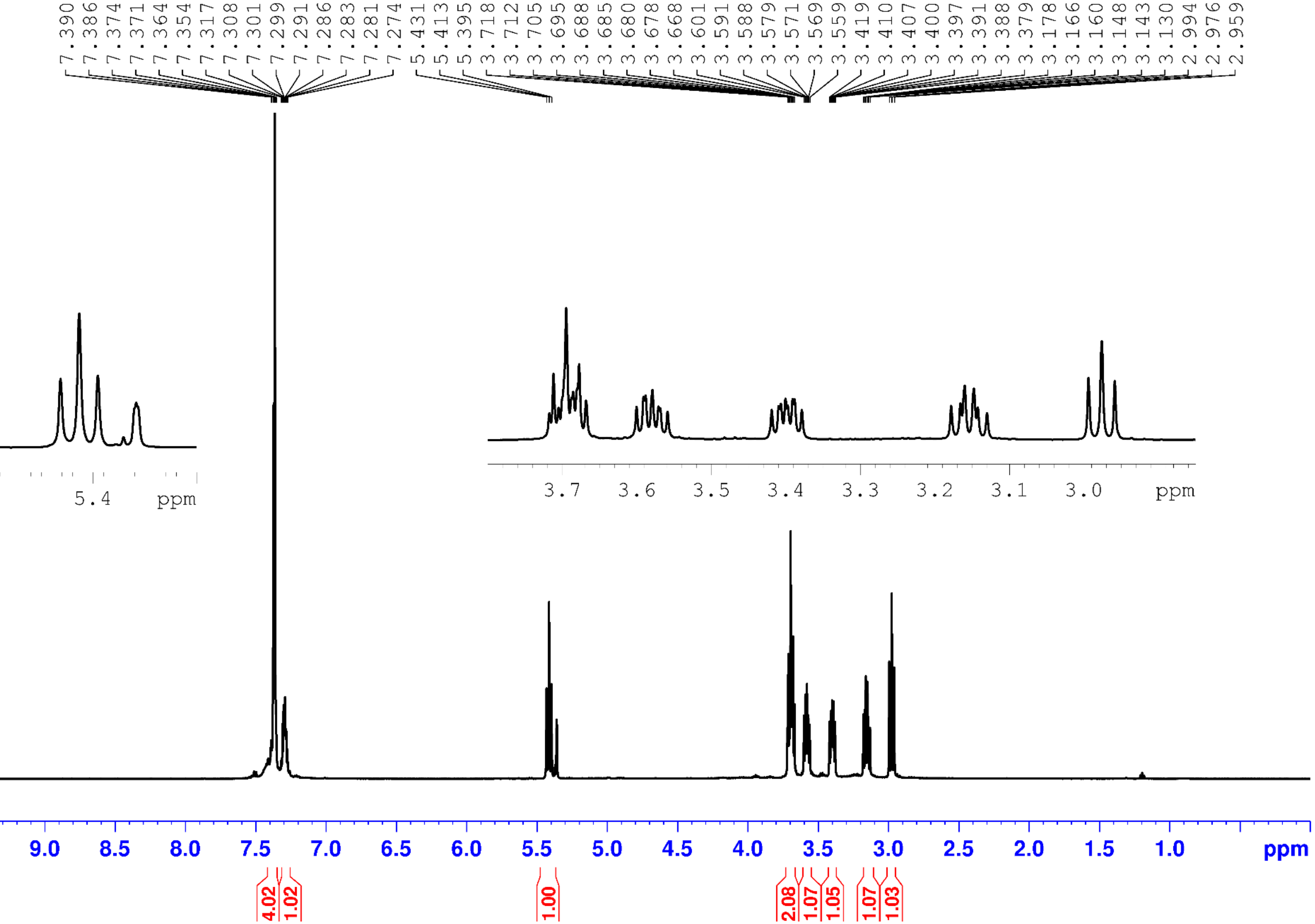
7.390
7.386
7.374
7.371
7.364
7.354
7.317
7.308
7.301
7.299
7.291
7.286
7.283
7.281
7.274
5.431
5.413
5.395
3.718
3.712
3.705
3.695
3.688
3.685
3.680
3.678
3.668
3.601
3.591
3.588
3.579
3.571
3.569
3.559
3.419
3.410
3.407
3.400
3.397
3.391
3.388
3.379
3.178
3.166
3.160
3.148
3.143
3.130
2.994
2.976
2.959
5.4
ppm
3.7
3.6
3.5
3.4
3.3
3.2
3.1
3.0
ppm
9.5
9.0
8.5
8.0
7.5
7.0
6.5
6.0
5.5
5.0
4.5
4.0
3.5
3.0
2.5
2.0
1.5
1.0
ppm
4.02
1.02
1.00
2.08
1.07
1.05
1.07
1.03

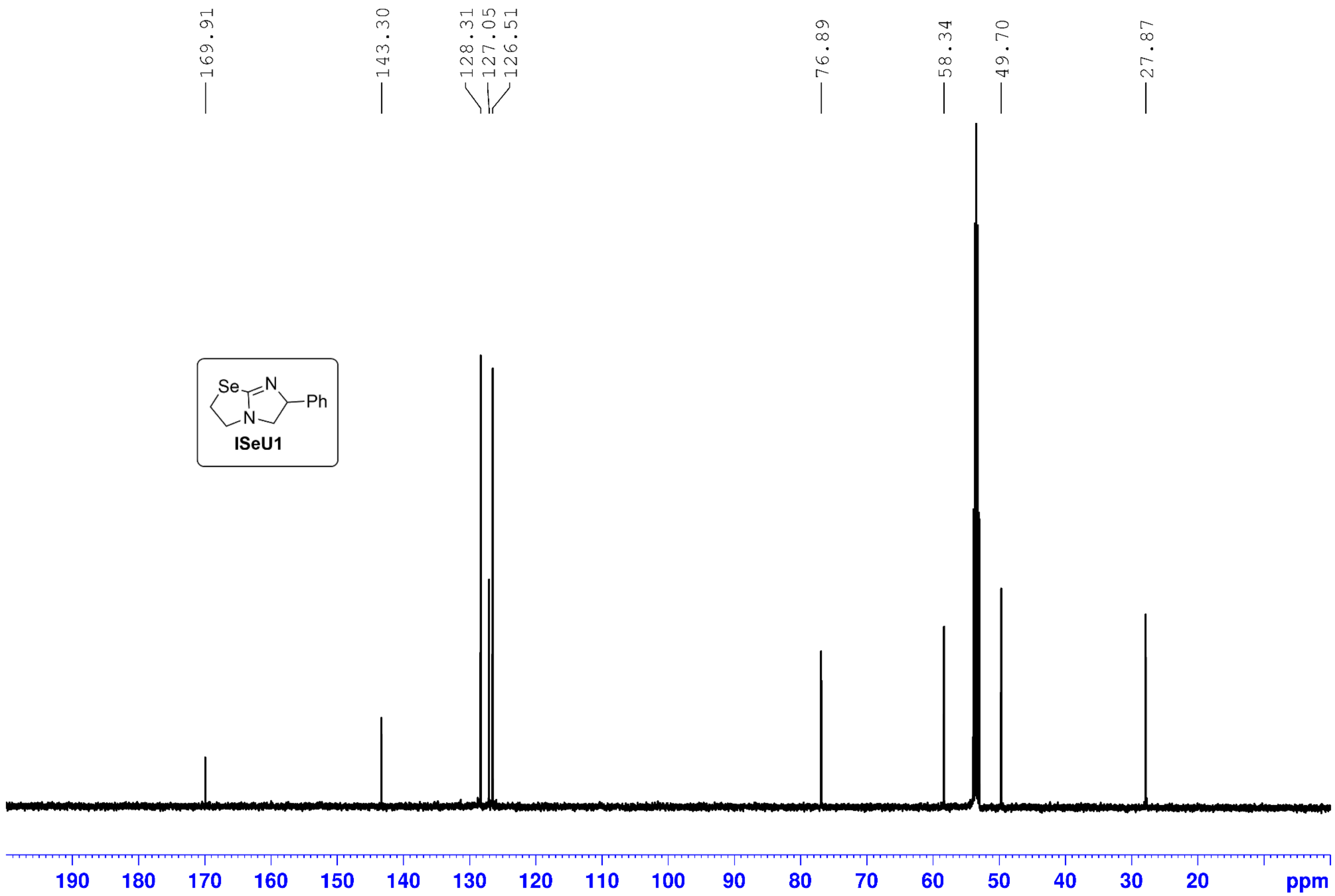
169.91
143.30
128.31
127.05
126.51
76.89
58.34
49.70
27.87
Se
N
N
Ph
ISeU1
190 180 170 160 150 140 130 120 110 100 90 80 70 60 50 40 30 20
ppm

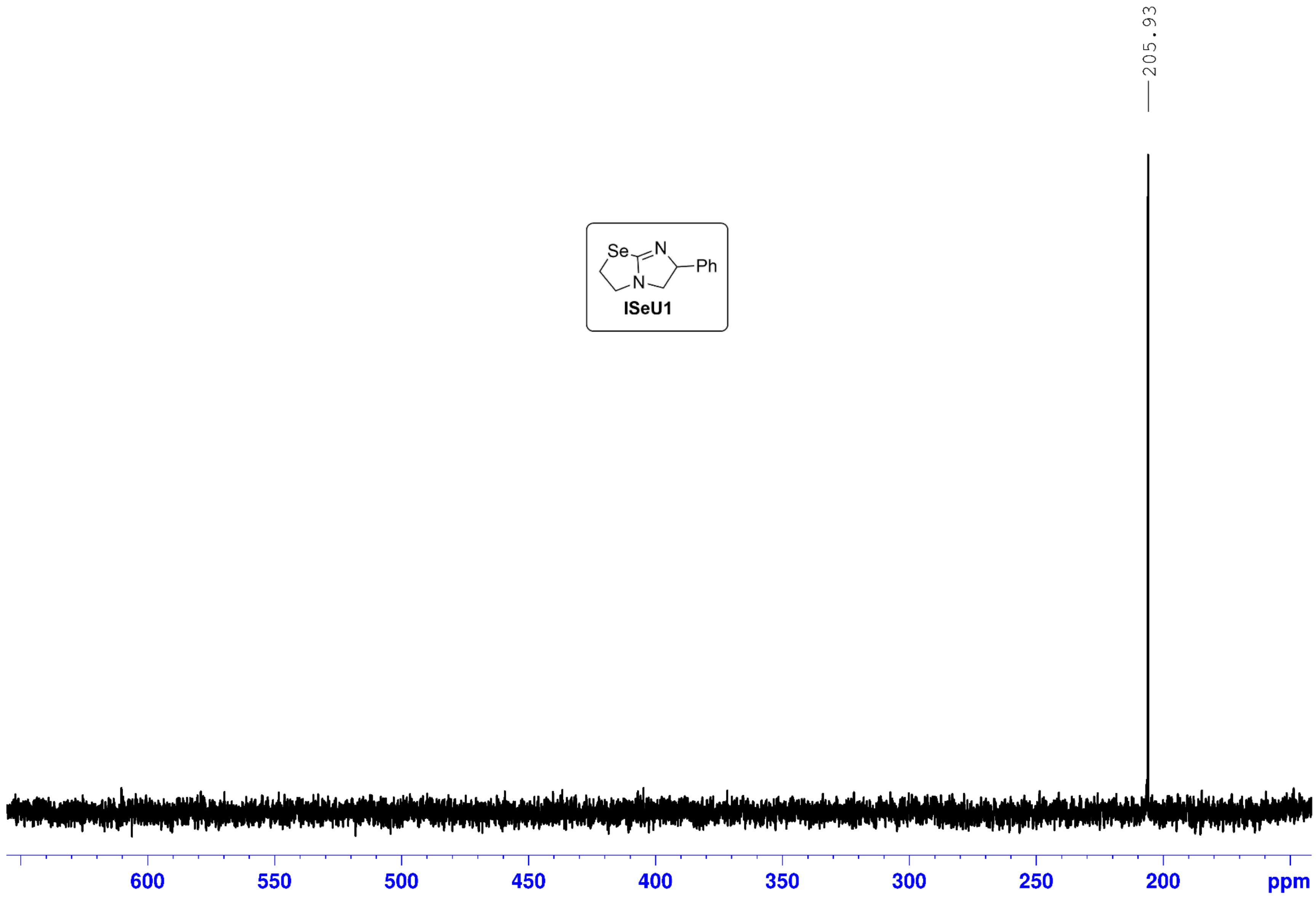
205.93
Se
N
N
Ph
ISeU1
600
550
500
450
400
350
300
250
200
ppm

Isothiocyanate (15a)

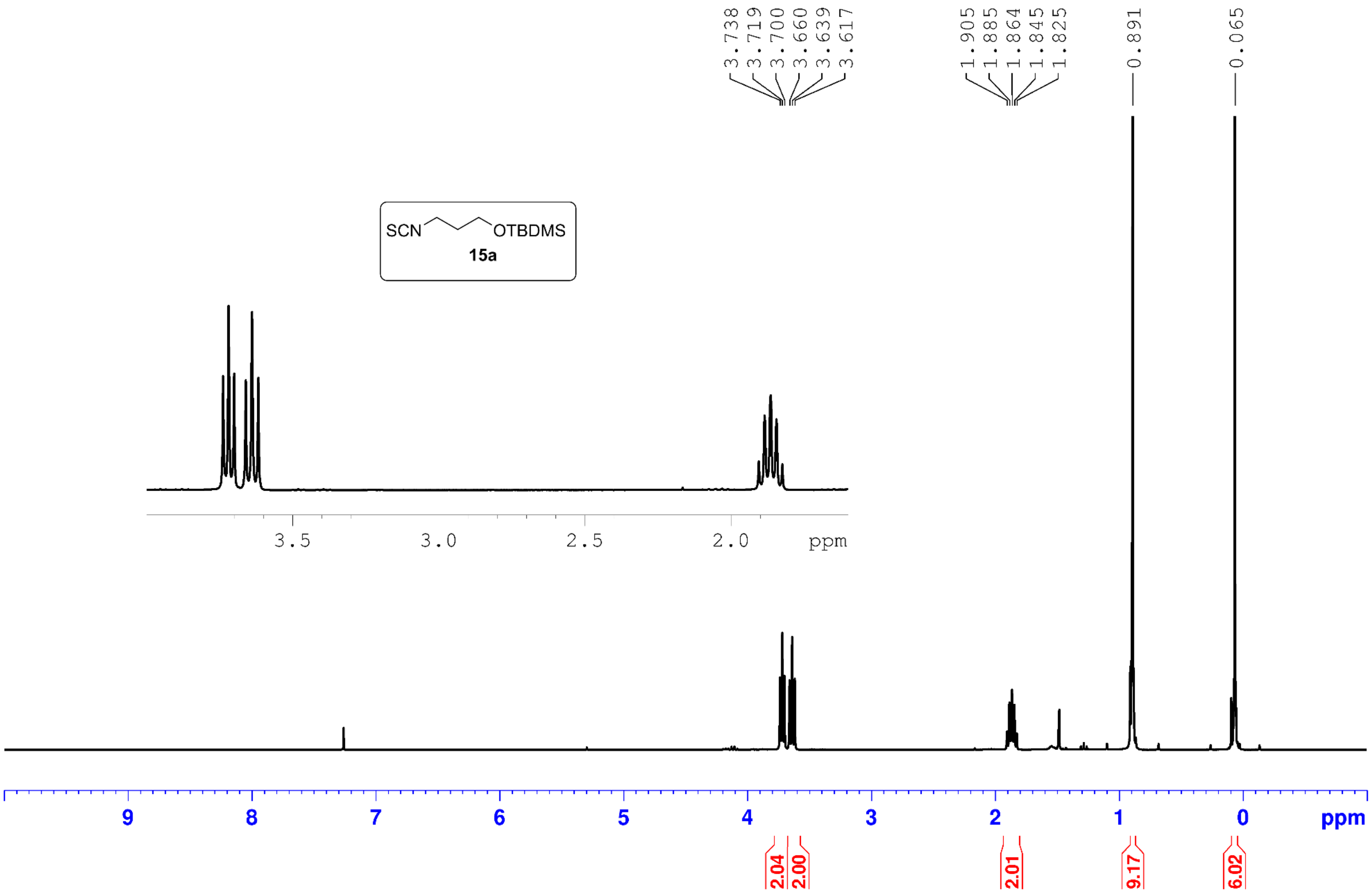

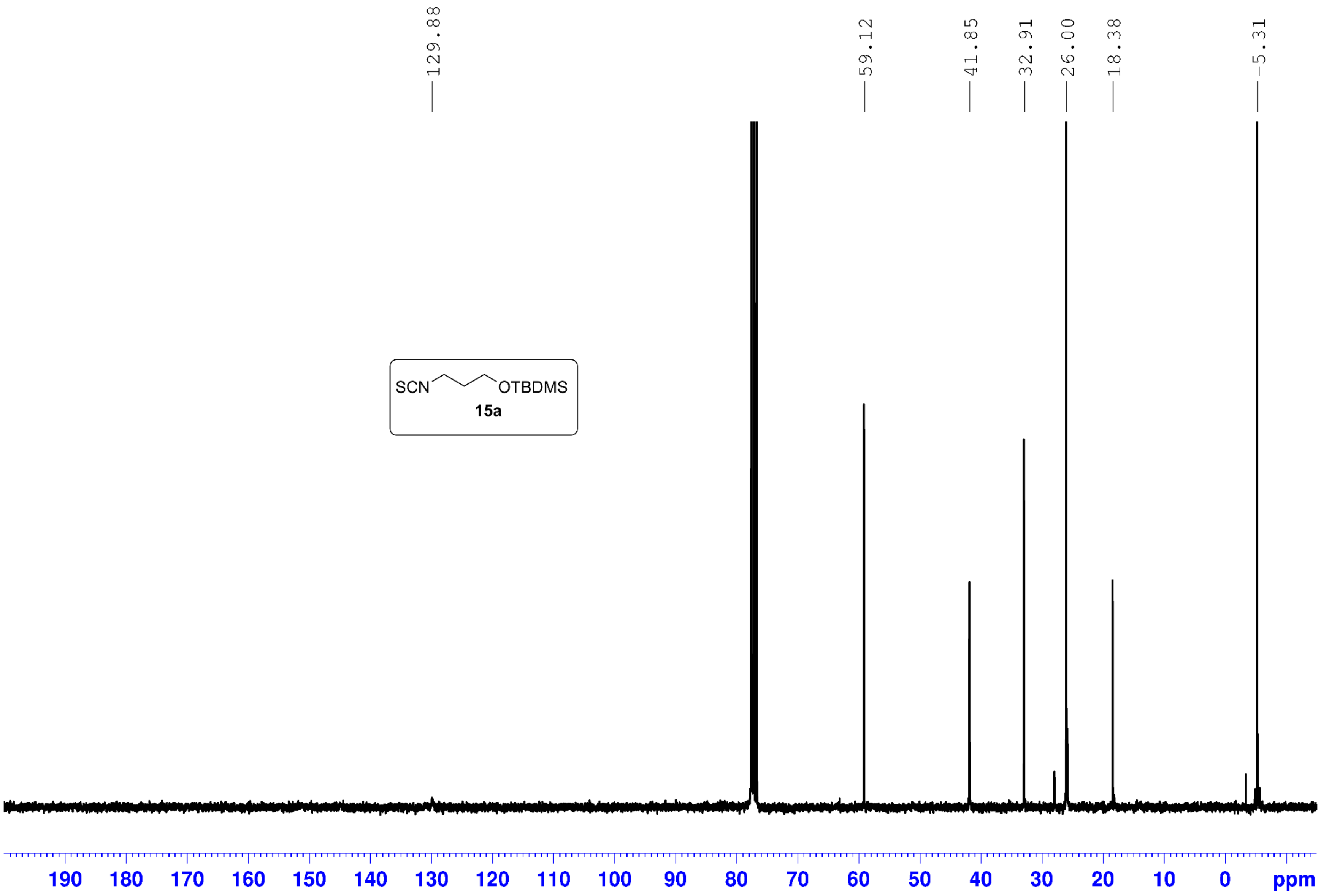
129.88
59.12
41.85
32.91
26.00
18.38
-5.31
SCN
OTBDMS
15a
190 180 170 160 150 140 130 120 110 100 90 80 70 60 50 40 30 20 10 0 ppm

Benzotellurazole 17a

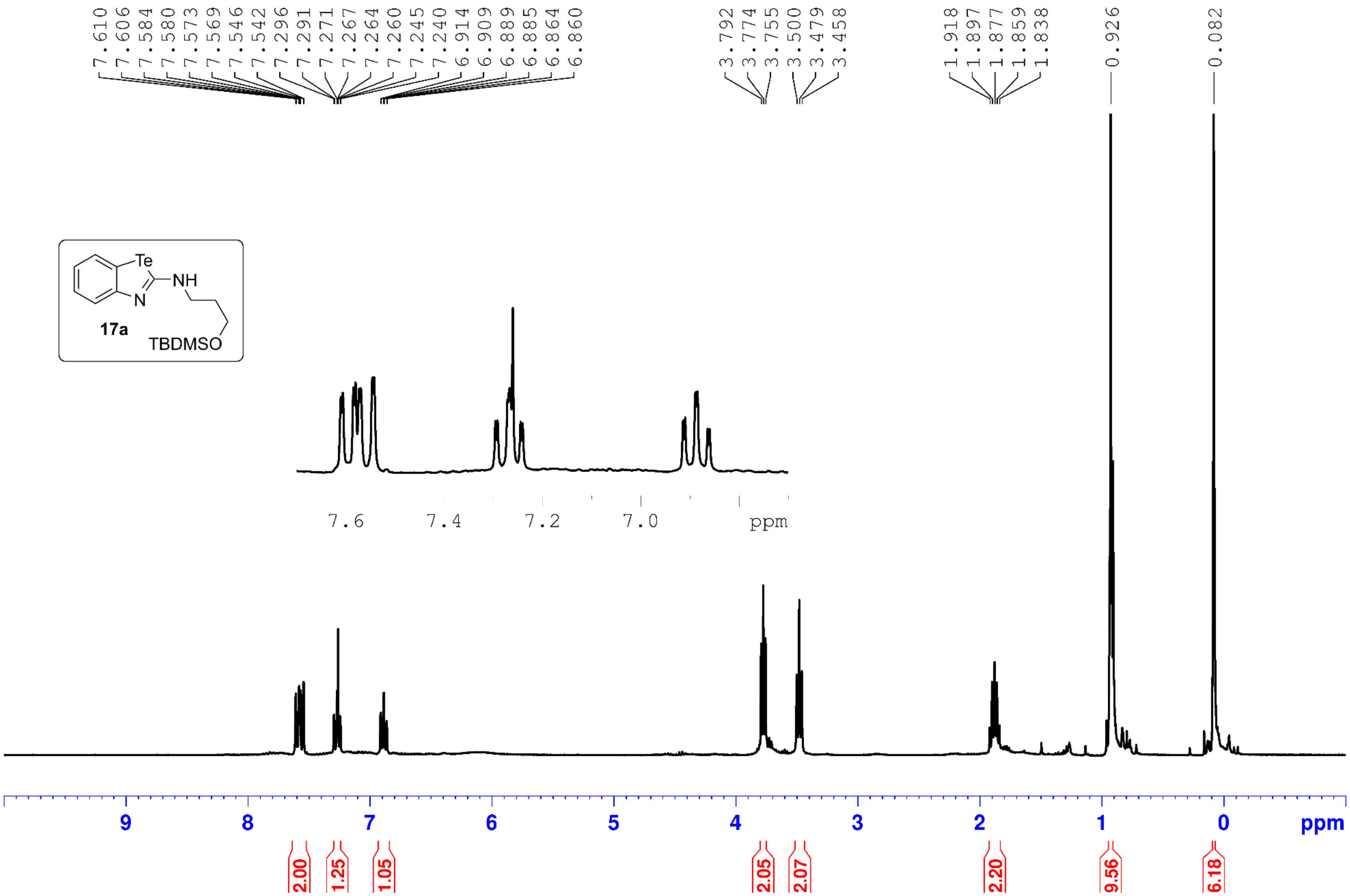

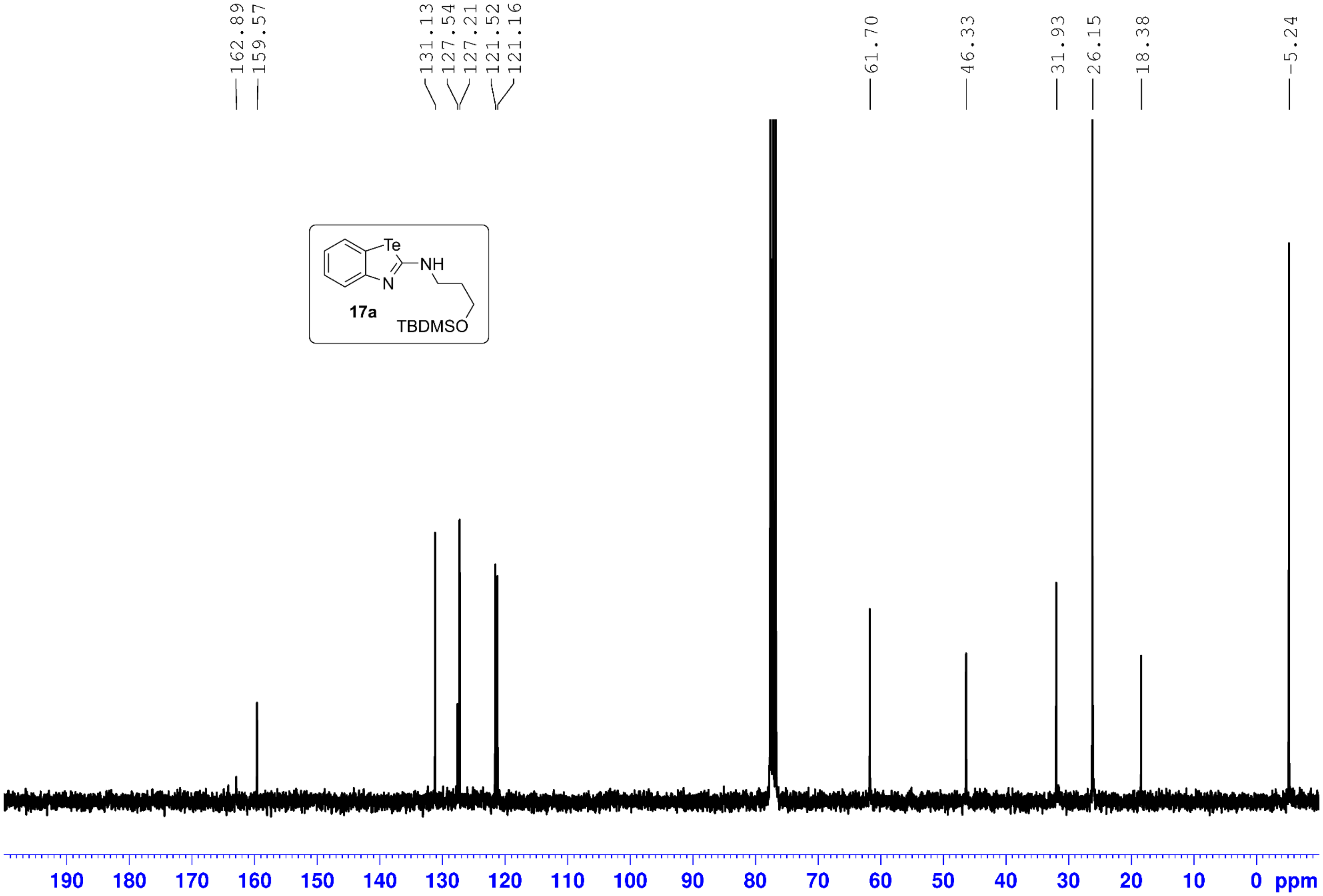
162.89
159.57
131.13
127.54
127.21
121.52
121.16
61.70
46.33
31.93
26.15
18.38
-5.24
Te
NH
N
17a
TBDMSO
190 180 170 160 150 140 130 120 110 100 90 80 70 60 50 40 30 20 10 0 ppm

Benzotellurazole 17b

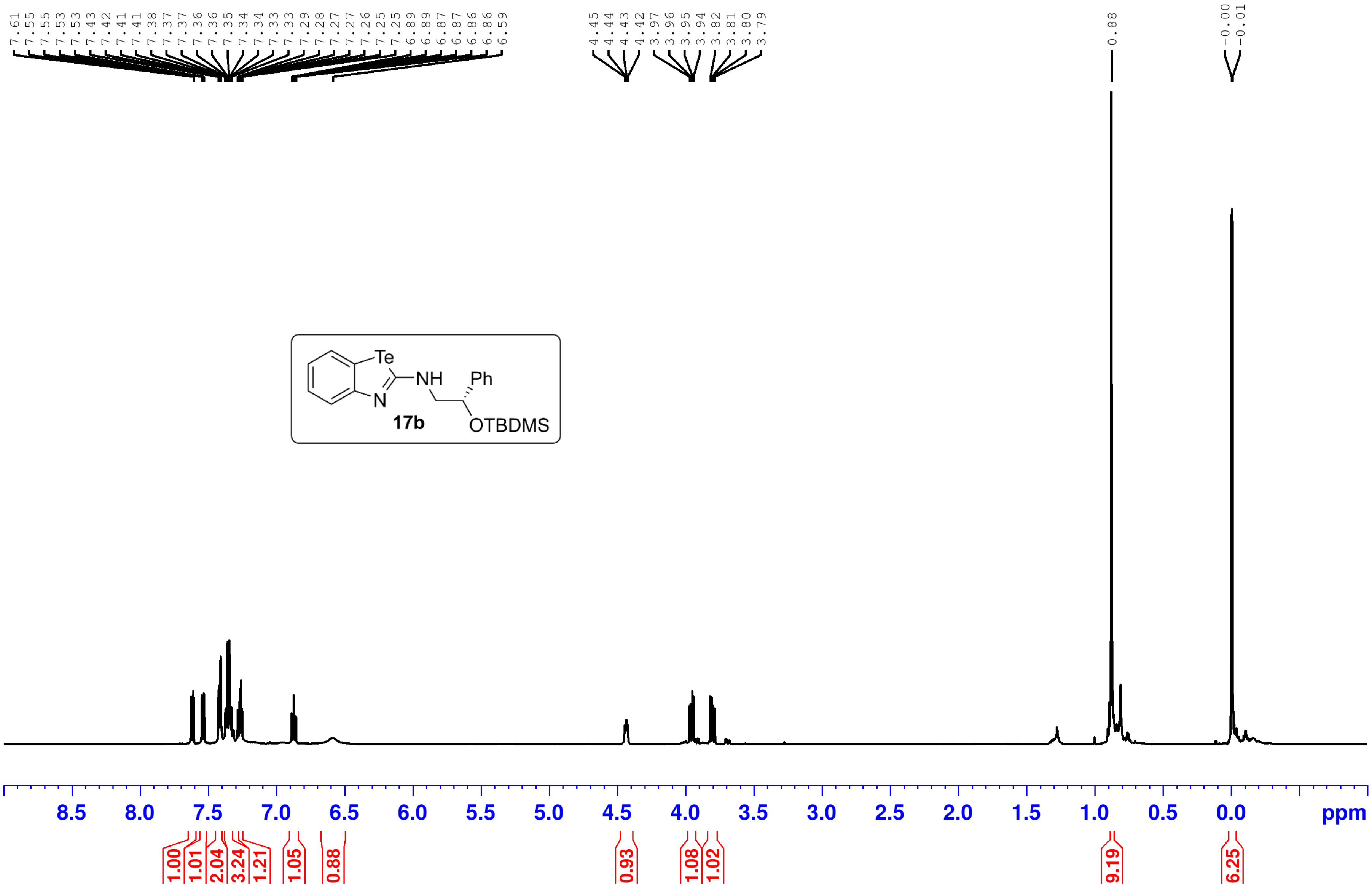

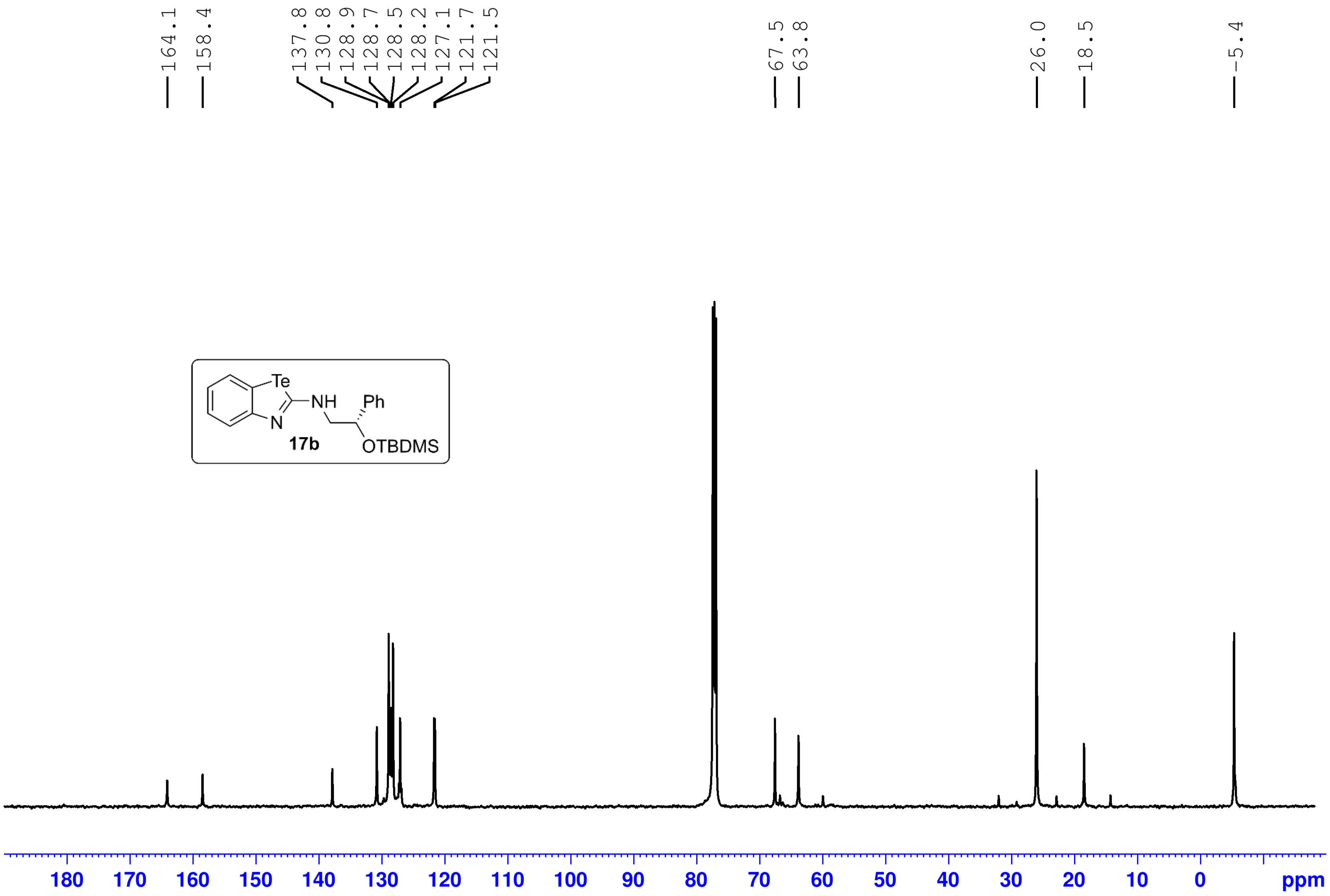
164.1
158.4
137.8
130.8
128.9
128.7
128.5
128.2
127.1
121.7
121.5
67.5
63.8
26.0
18.5
-5.4
Te
NH
Ph
N
17b
OTBDMS
180
170
160
150
140
130
120
110
100
90
80
70
60
50
40
30
20
10
0
ppm

Deprotected Benzotellurazole (17a')

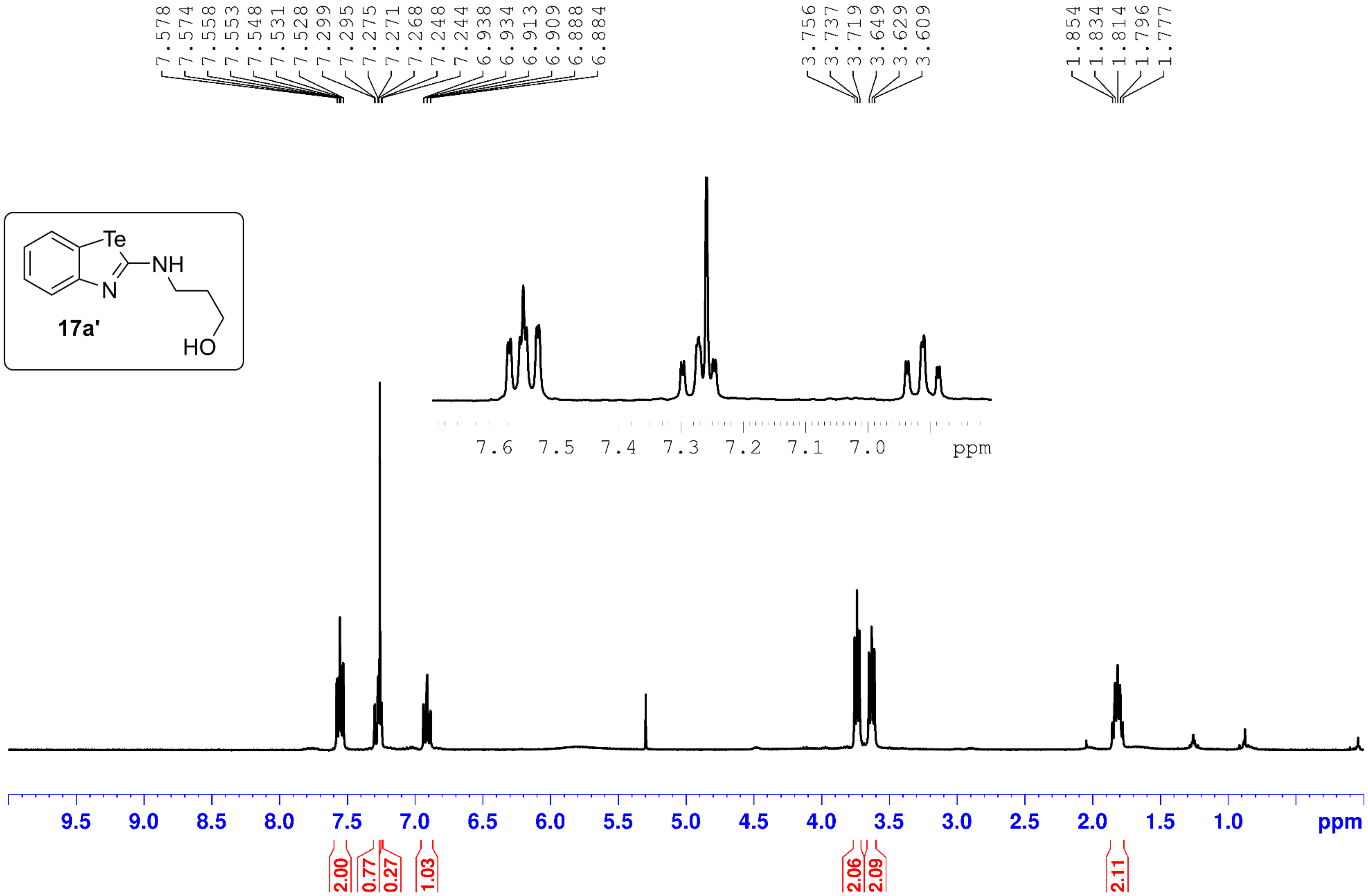

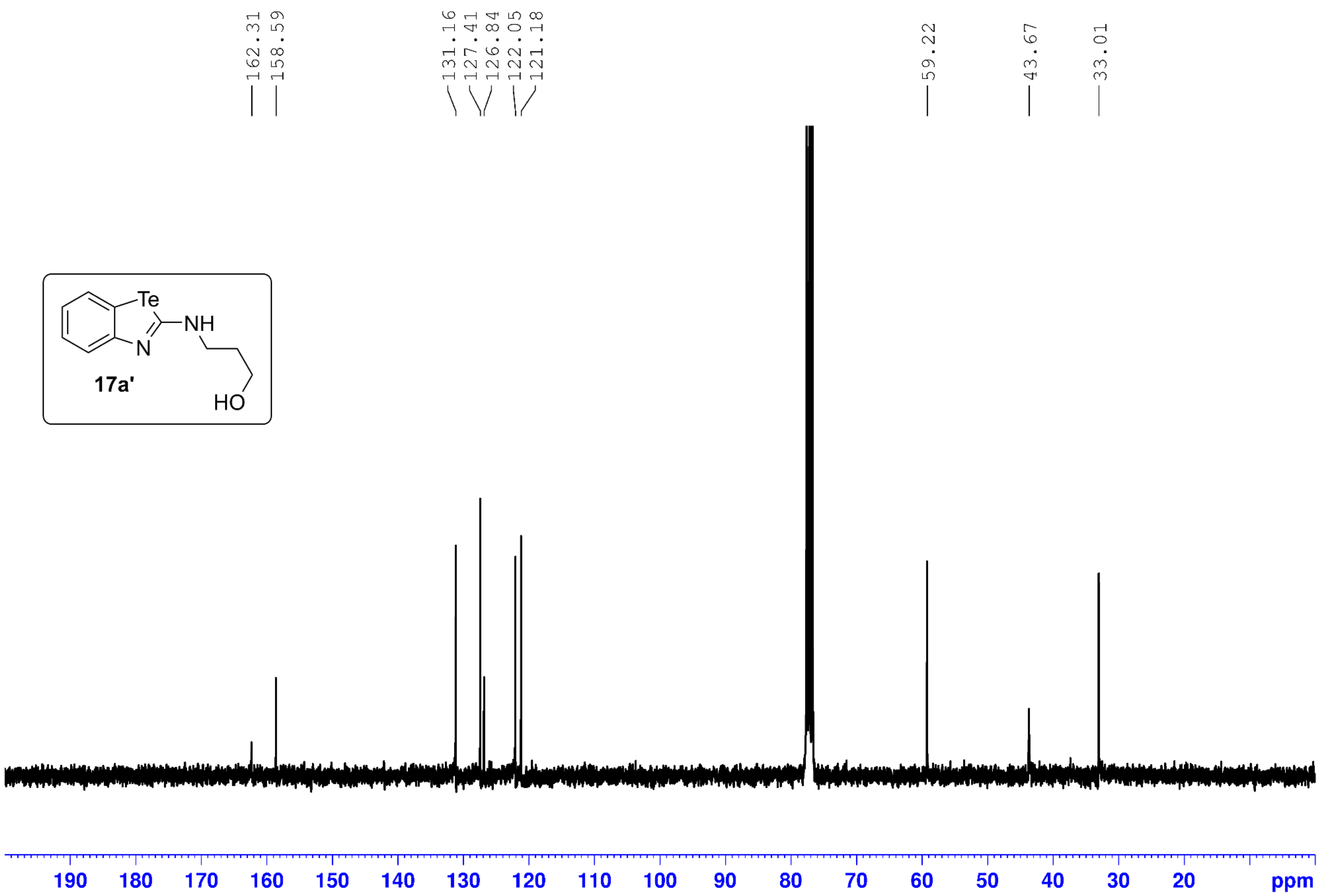

162.31
158.59
131.16
127.41
126.84
122.05
121.18
59.22
43.67
33.01
Te
NH
N
HO
17a'
190
180
170
160
150
140
130
120
110
100
90
80
70
60
50
40
30
20
ppm

Deprotected Benzotellurazole 17b'

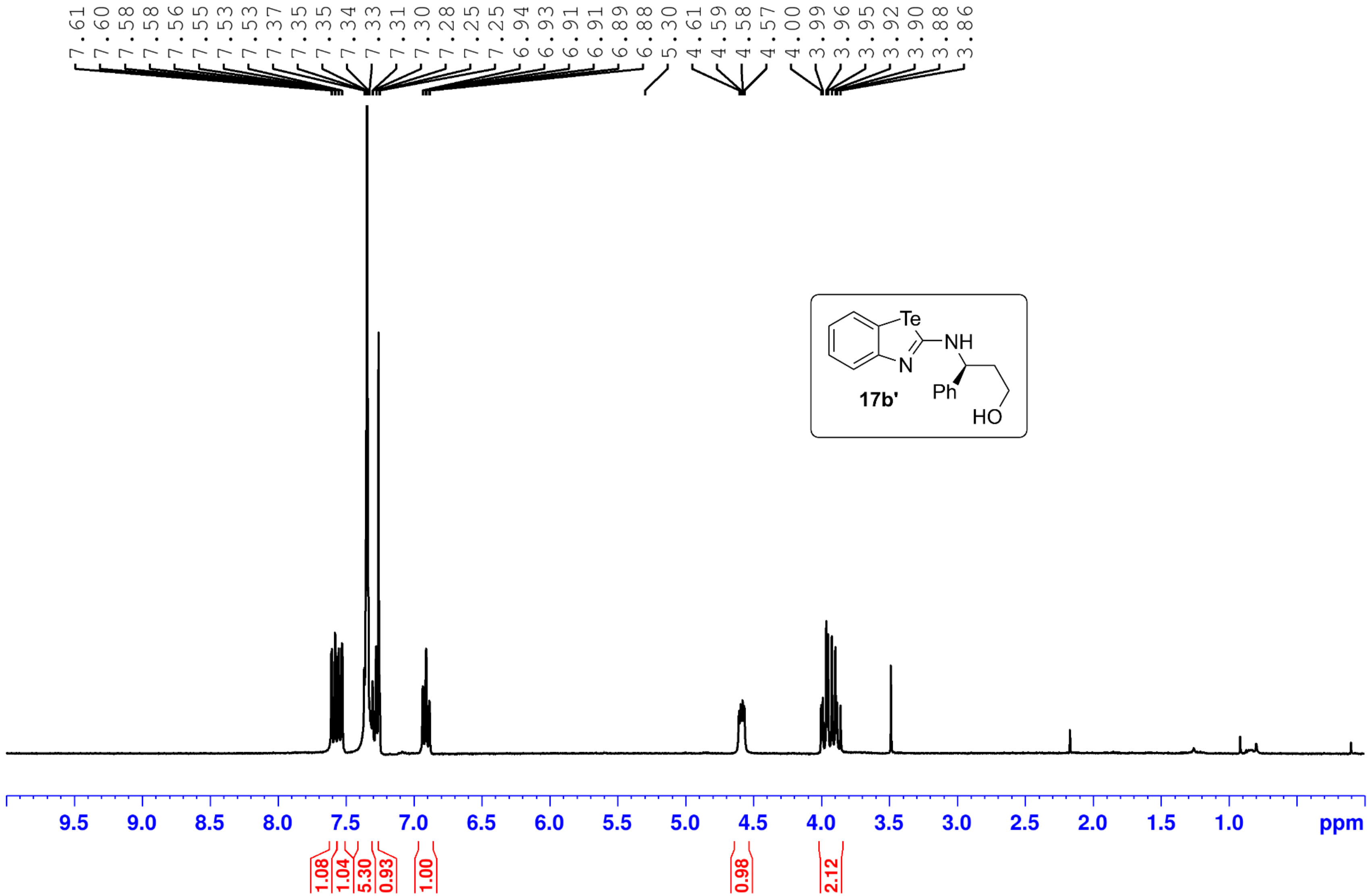

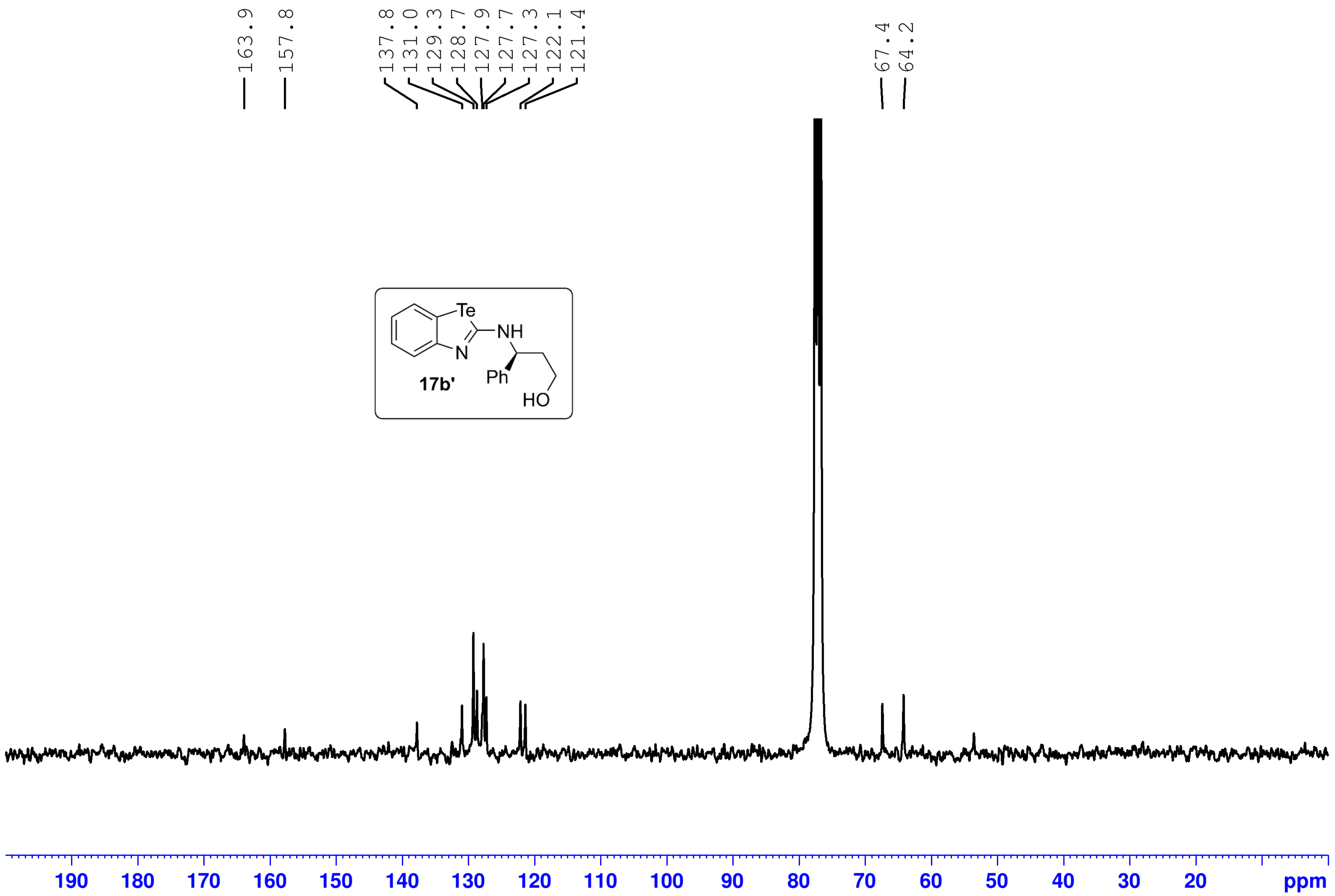
163.9
157.8
137.8
131.0
129.3
128.7
127.9
127.7
127.3
122.1
121.4
67.4
64.2
Te
NH
N
Ph
HO
17b'
190
180
170
160
150
140
130
120
110
100
90
80
70
60
50
40
30
20
ppm

TeDHPB (ITeU3)

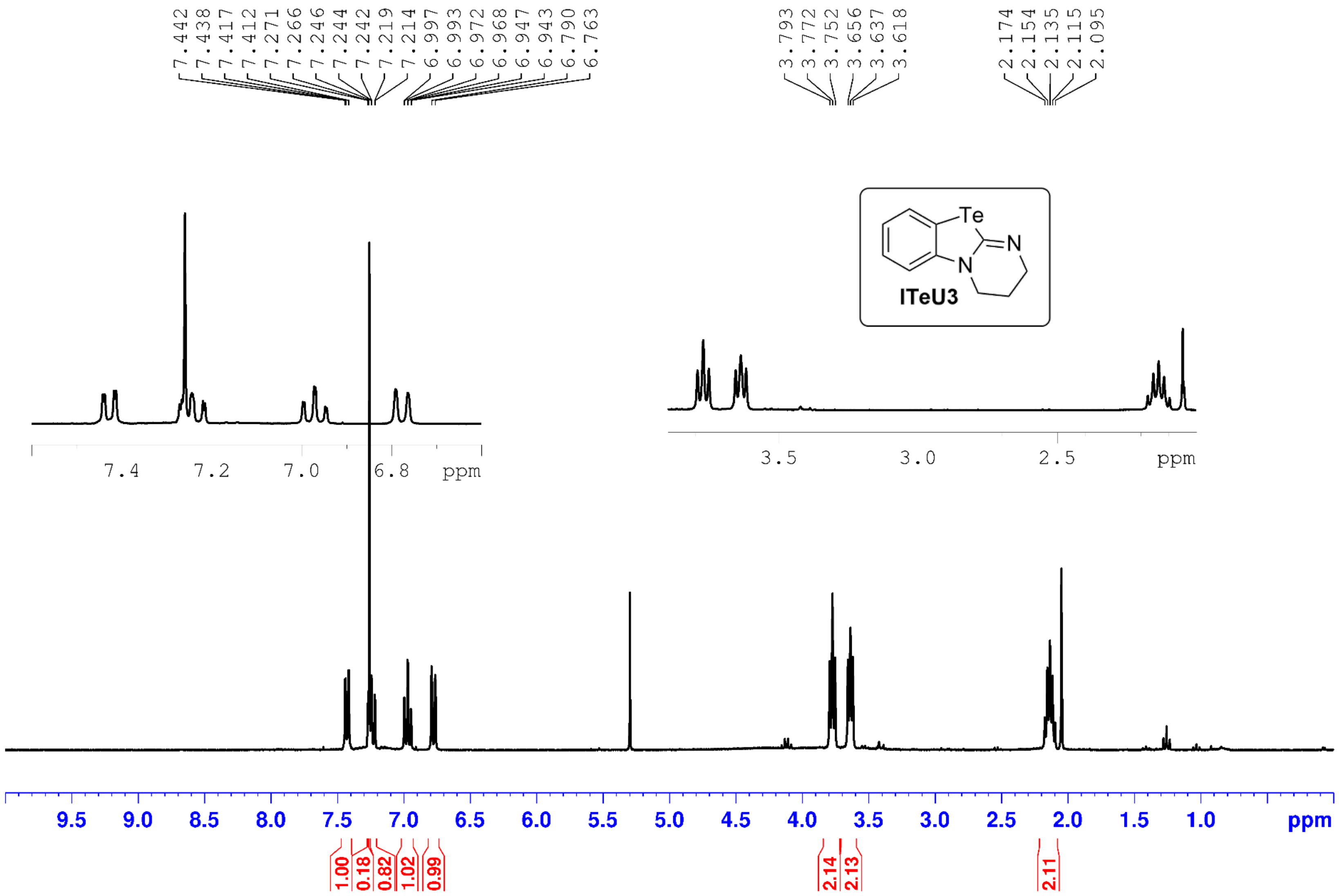

1H,13C CPD
D1: 30s
NS: 482

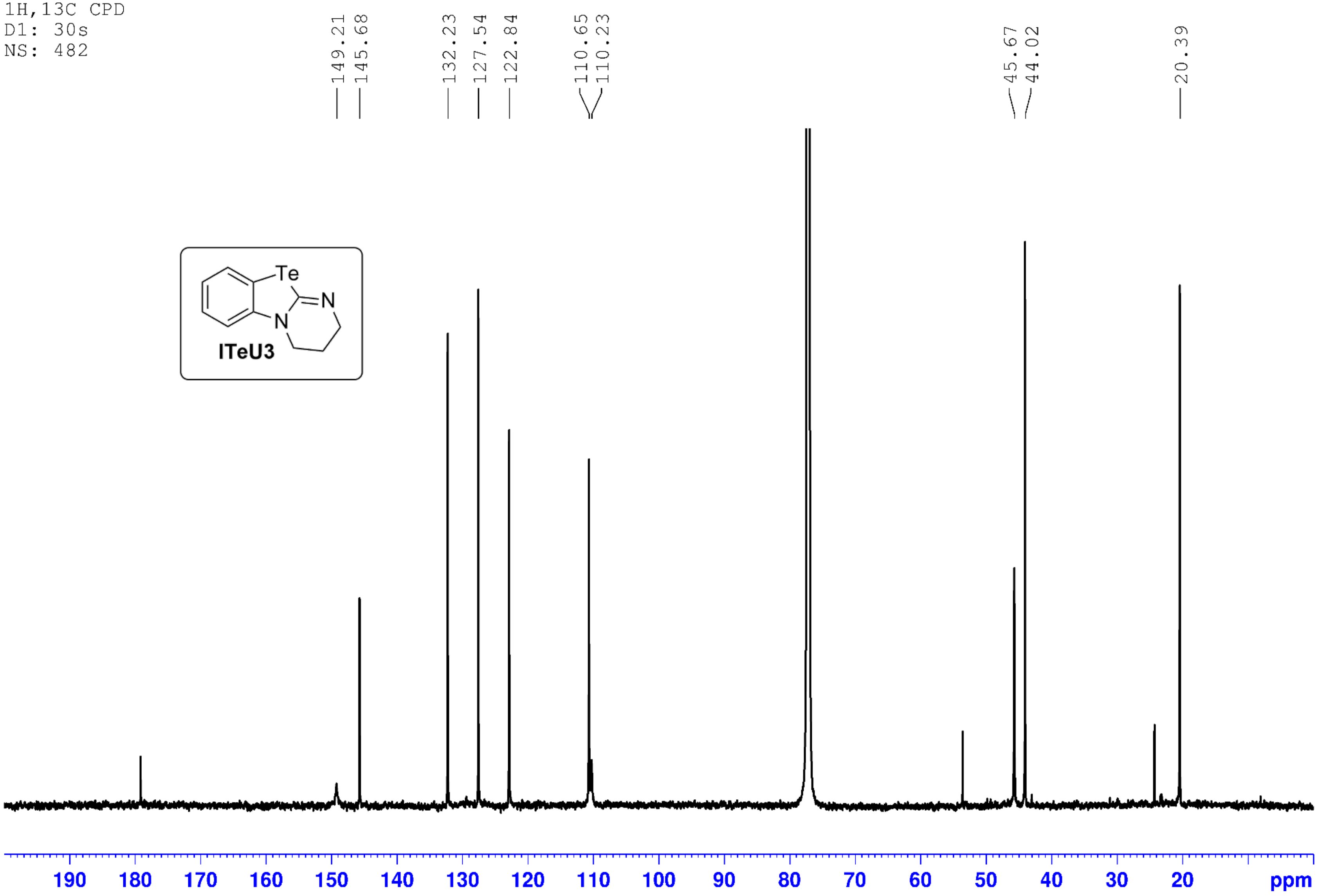

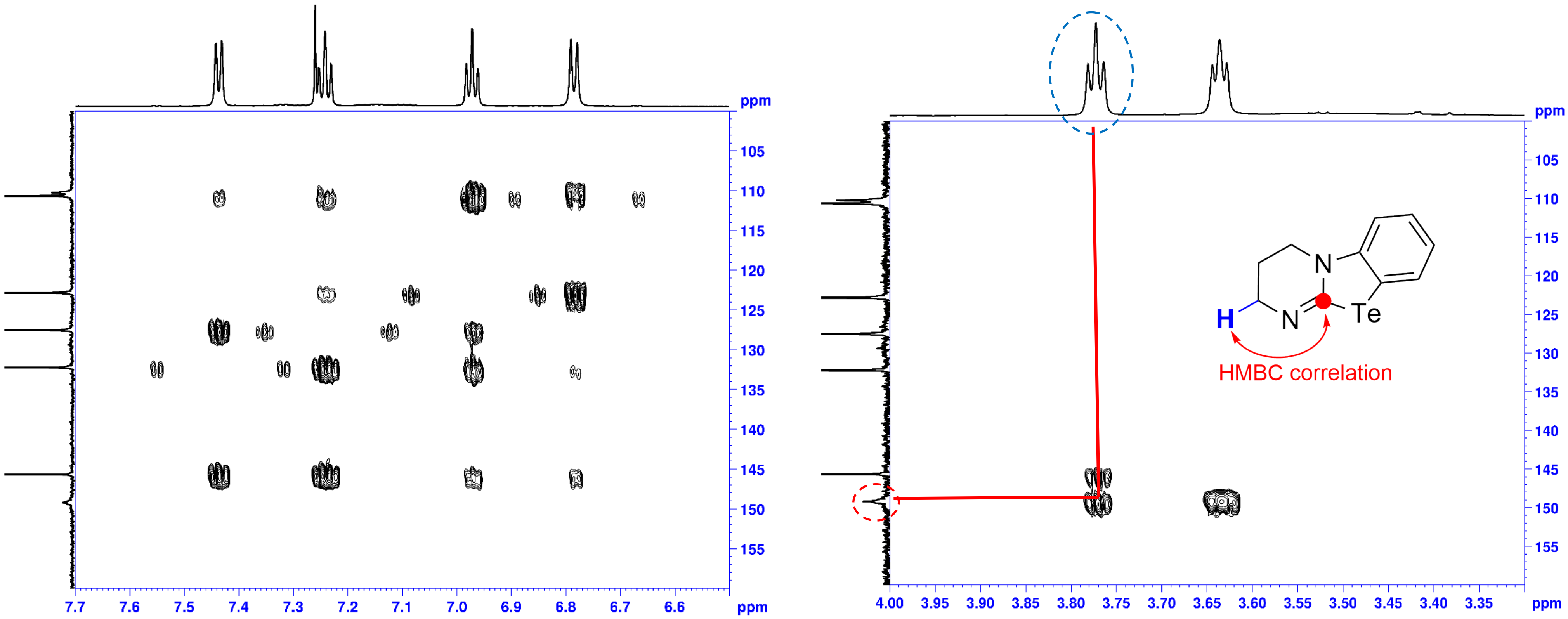

Figure S61: HMBC correlations of TeDHPB: Carbons adjacent to the Tellurium atom become more easily visible.

TeBTM (ITeU2)

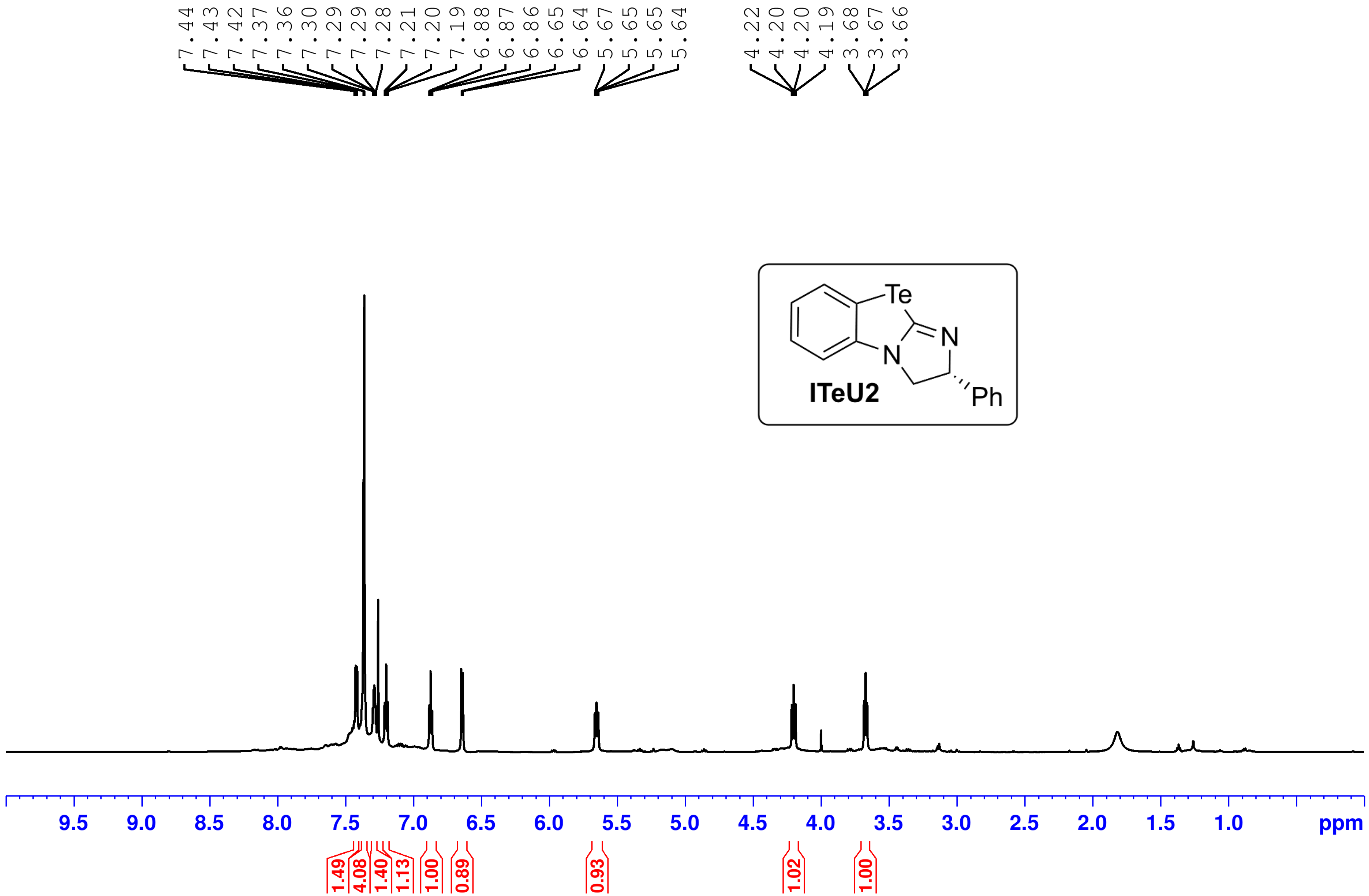
7.44
7.43
7.42
7.37
7.36
7.30
7.29
7.29
7.28
7.21
7.20
7.19
6.88
6.87
6.86
6.65
6.64
5.67
5.65
5.65
5.64
4.22
4.20
4.20
4.19
3.68
3.67
3.66
Te
N
N
Ph
ITeU2
9.5
9.0
8.5
8.0
7.5
7.0
6.5
6.0
5.5
5.0
4.5
4.0
3.5
3.0
2.5
2.0
1.5
1.0
ppm
1.49
4.08
1.40
1.13
1.00
0.89
0.93
1.02
1.00

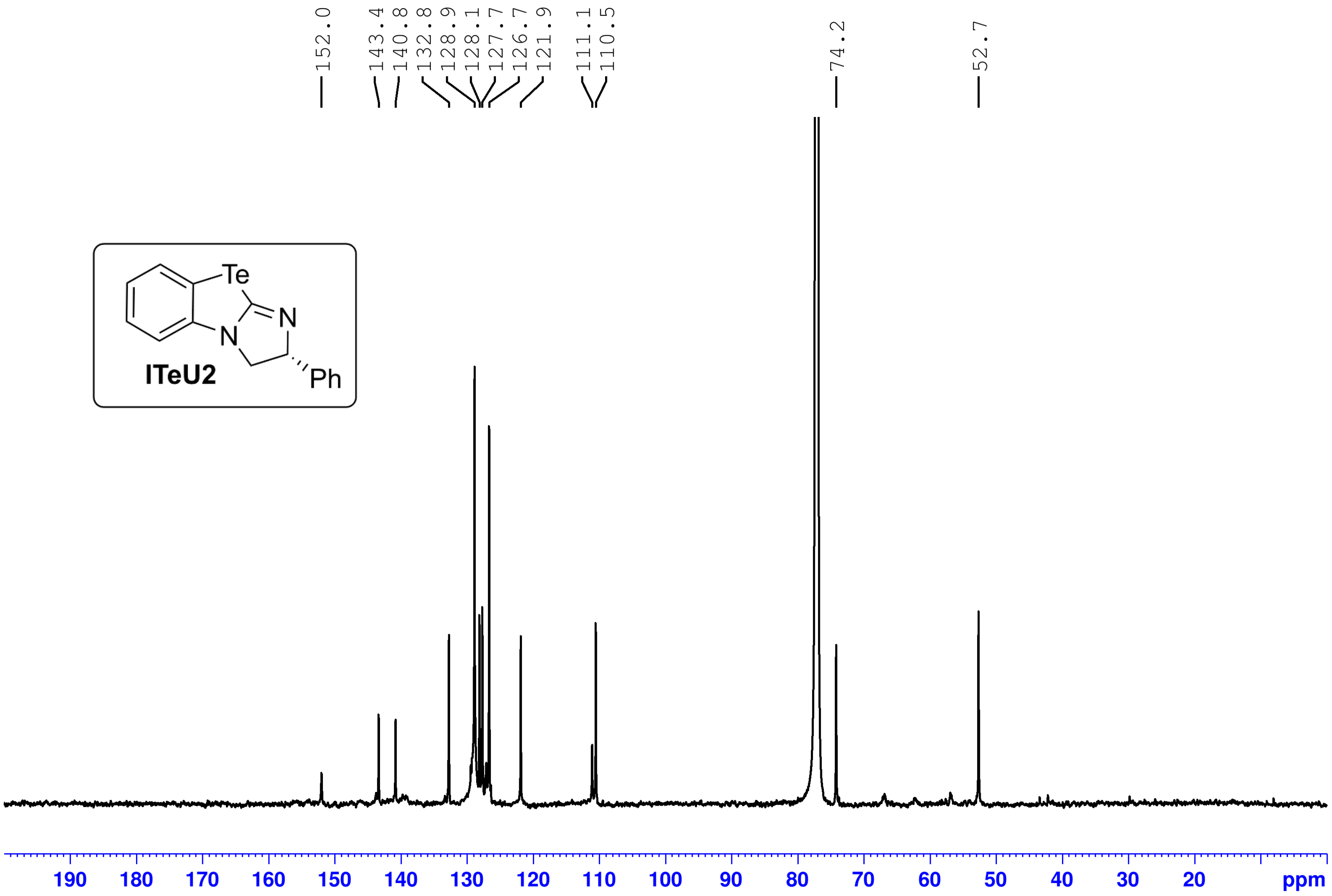
152.0
143.4
140.8
132.8
128.9
128.1
127.7
126.7
121.9
111.1
110.5
74.2
52.7
Te
N
N
Ph
ITeU2
190
180
170
160
150
140
130
120
110
100
90
80
70
60
50
40
30
20
ppm

SeTM-(dma)₂CH⁺ Adduct (19p)

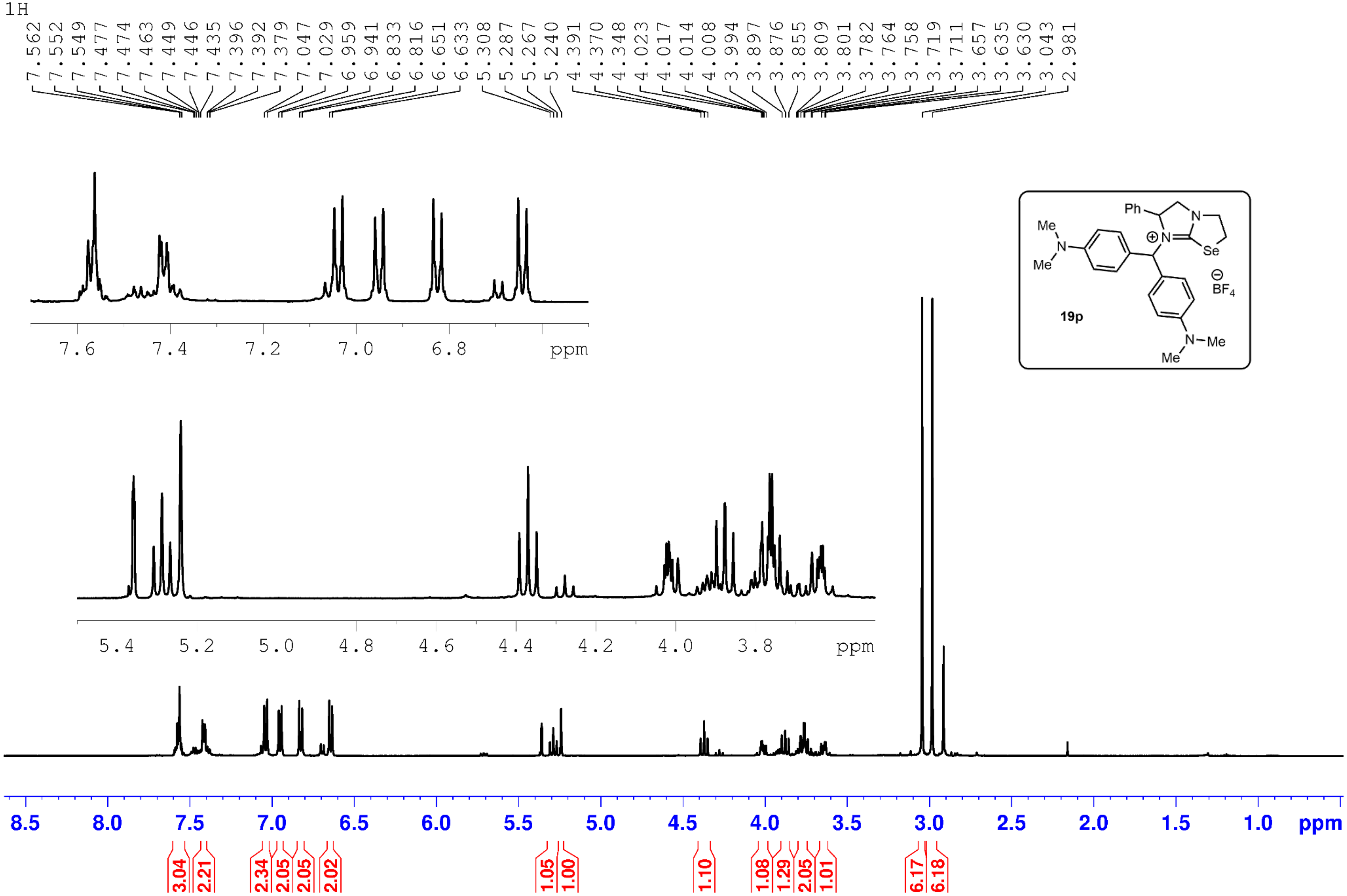

19p

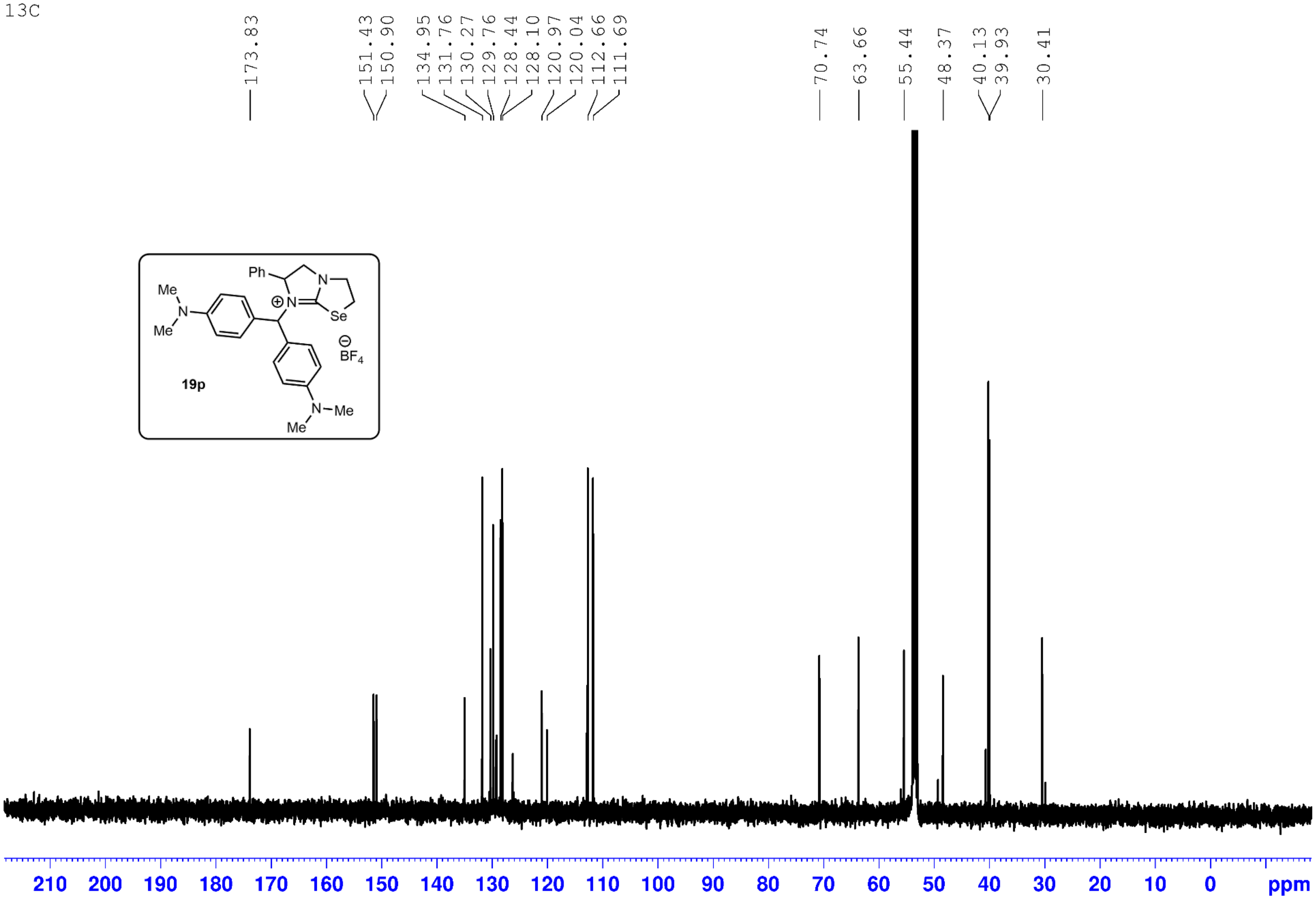
13C
173.83
151.43
150.90
134.95
131.76
130.27
129.76
128.44
128.10
120.97
120.04
112.66
111.69
70.74
63.66
55.44
48.37
40.13
39.93
30.41
Ph
Me
N
Me
N
Se
BF4
19p
N
Me
Me
210 200 190 180 170 160 150 140 130 120 110 100 90 80 70 60 50 40 30 20 10 0 ppm

77SeIG

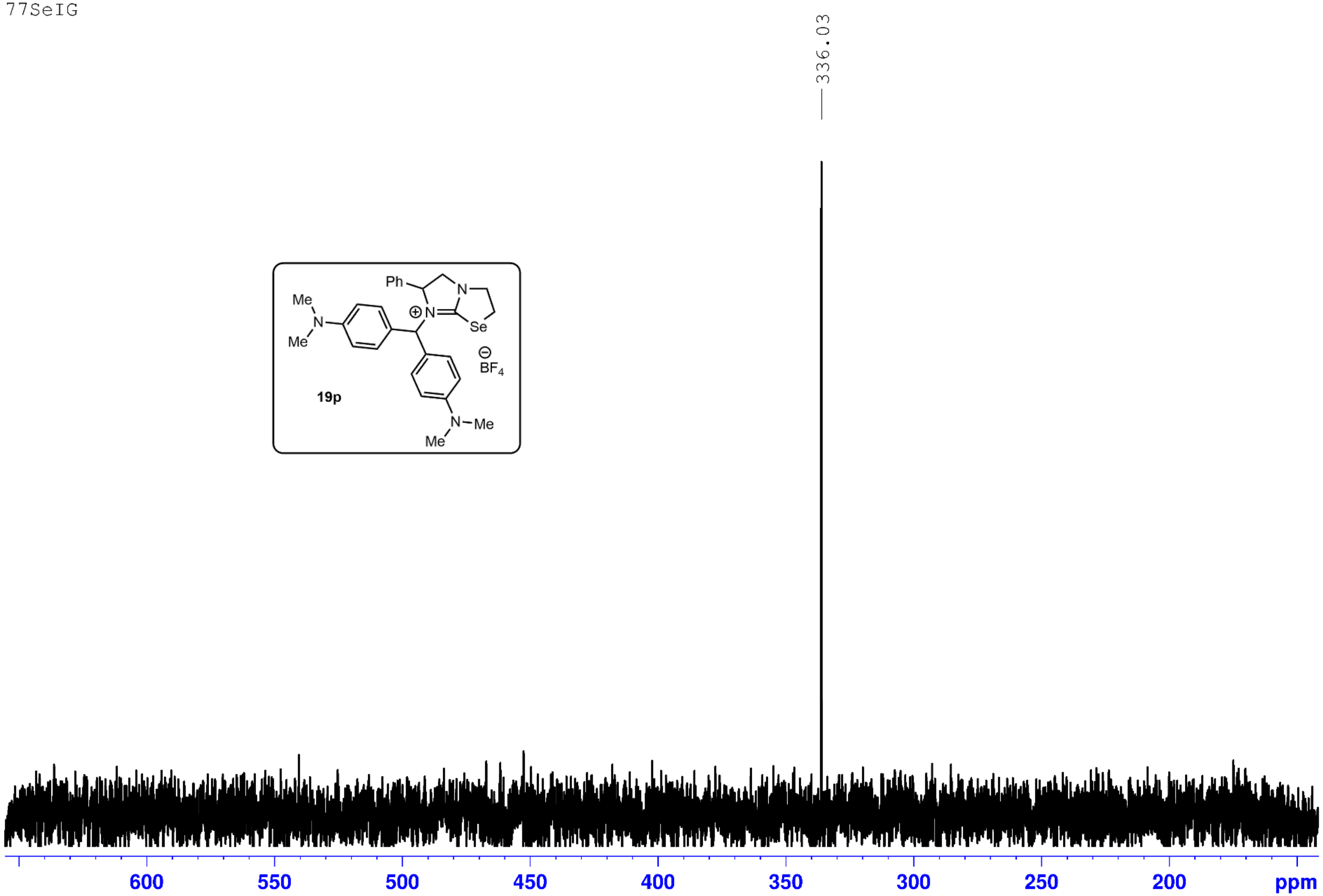
336.03
Ph
N
Se
Me
N
Me
N
BF4
19p
N
Me
Me
600
550
500
450
400
350
300
250
200
ppm

SeBTM-(dma)$_2$CH$^+$ Adduct (19q)

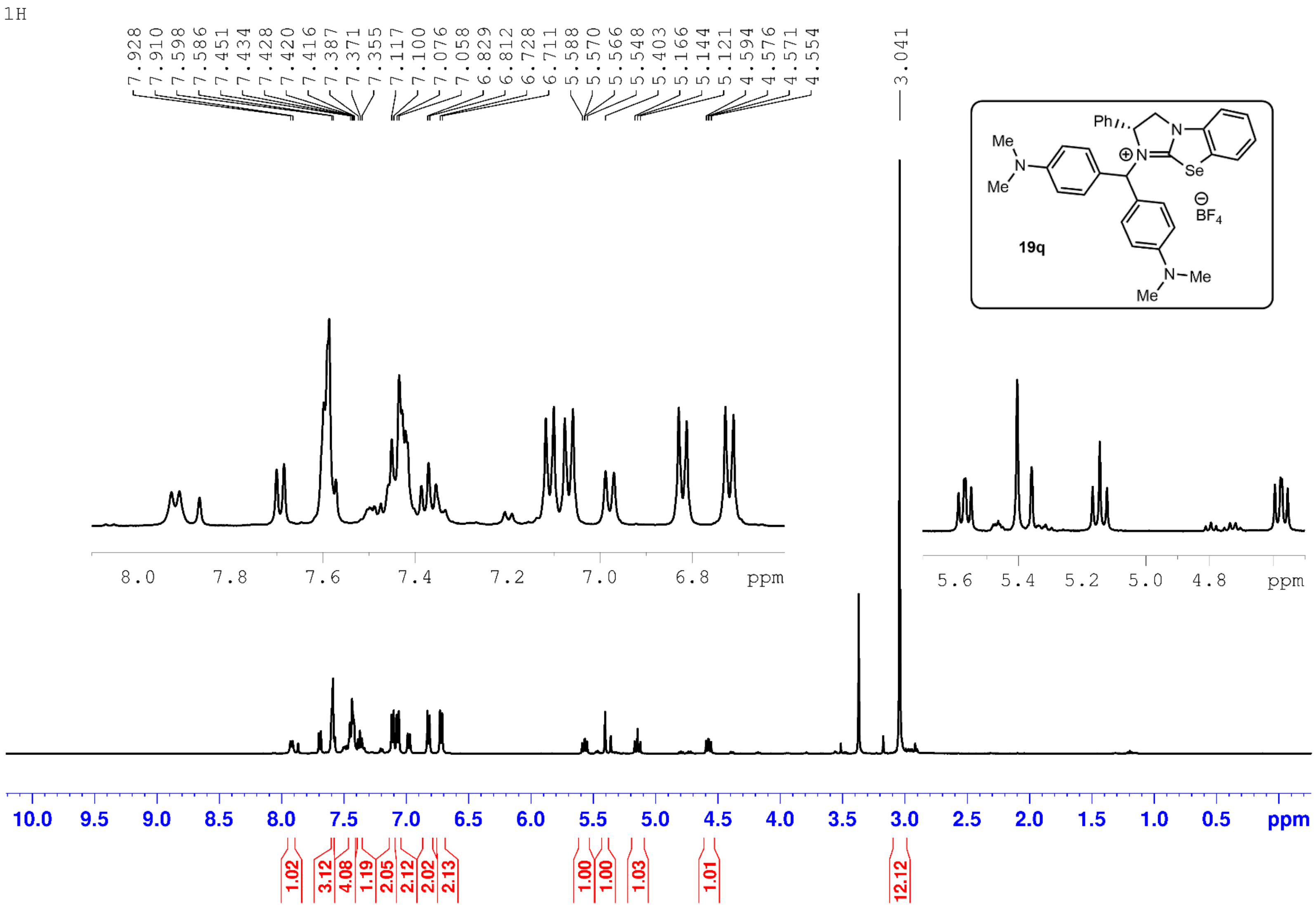

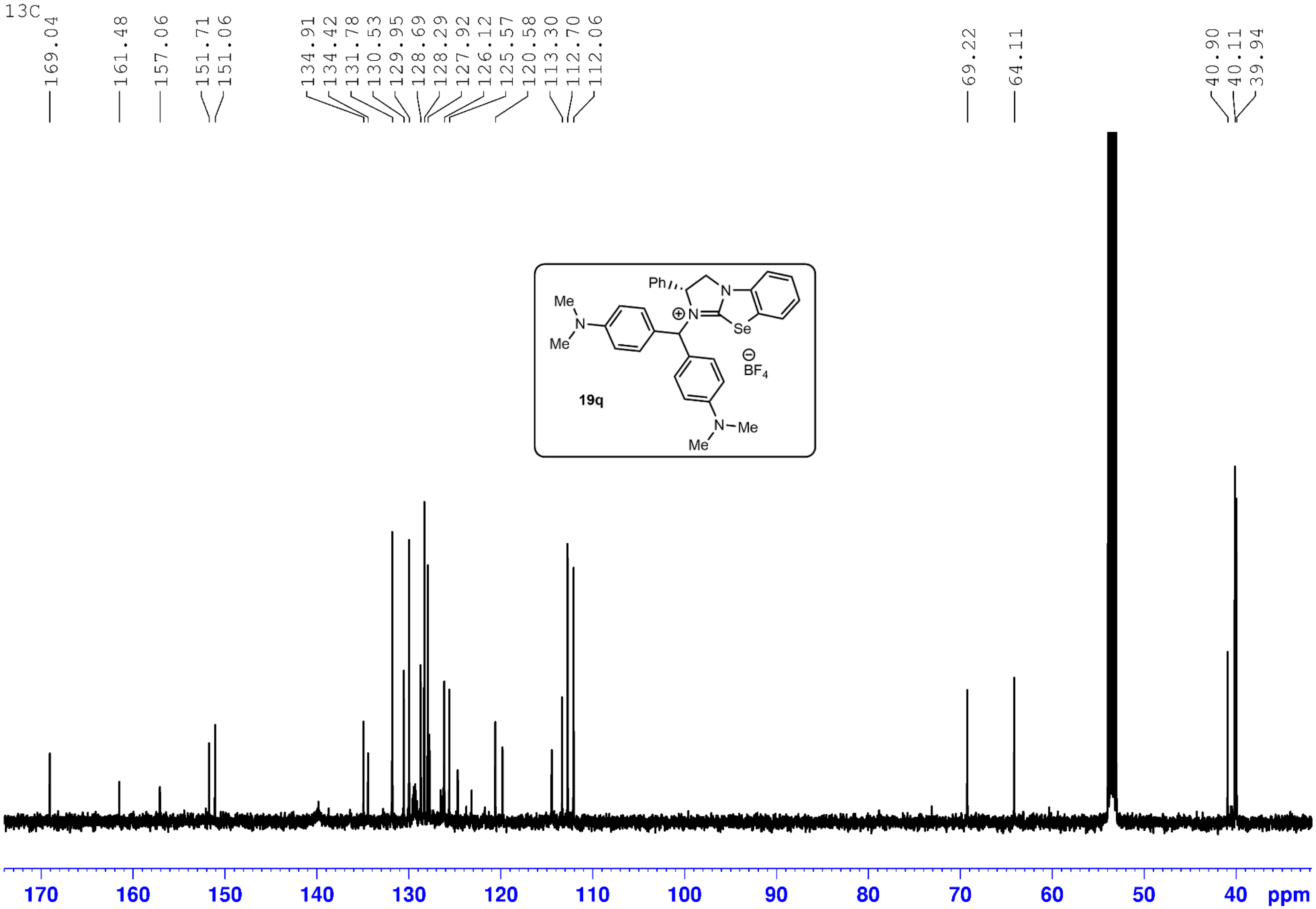
13C
169.04
161.48
157.06
151.71
151.06
134.91
134.42
131.78
130.53
129.95
128.69
128.29
127.92
126.12
125.57
120.58
113.30
112.70
112.06
69.22
64.11
40.90
40.11
39.94
Ph
N
Se
Me
Me
N
BF_4
19q
N
Me
Me
170
160
150
140
130
120
110
100
90
80
70
60
50
40
ppm

77SeIG

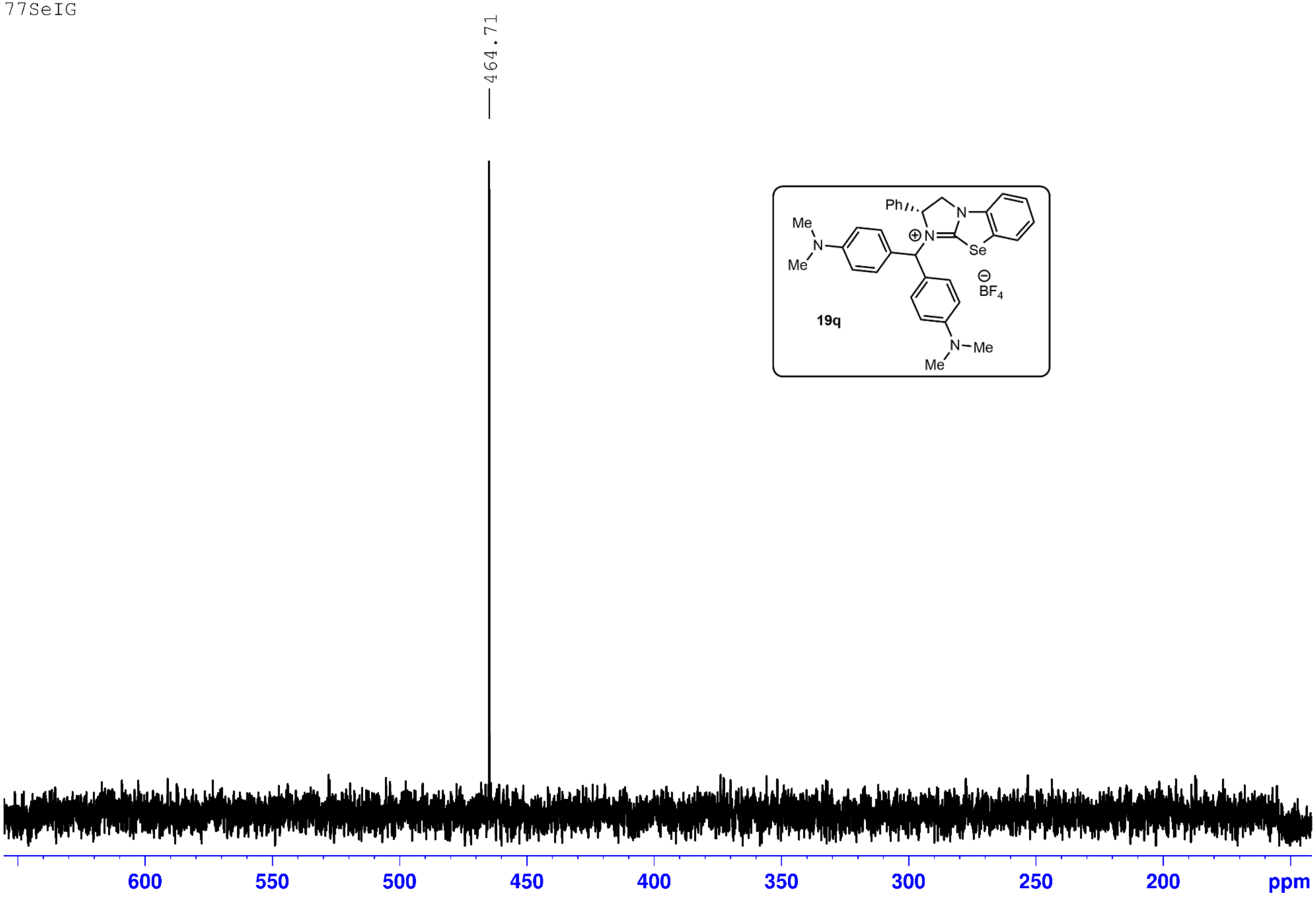
464.71
Ph
Me
N
Me
N
N
Se
BF4
19q
N
Me
Me
600
550
500
450
400
350
300
250
200
ppm

SeHyperBTM-(dma)$_2$CH$^+$ Adduct (19r)

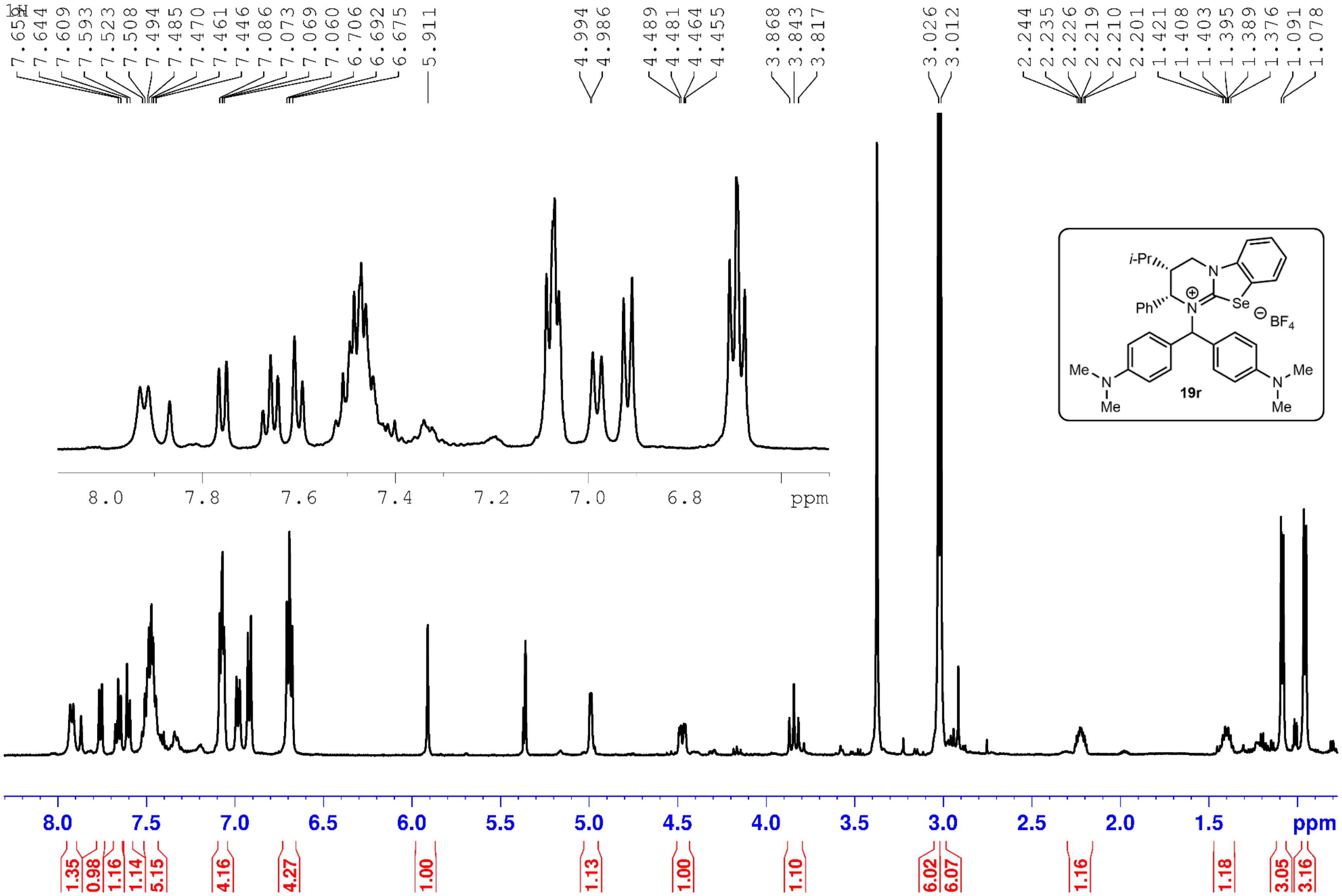

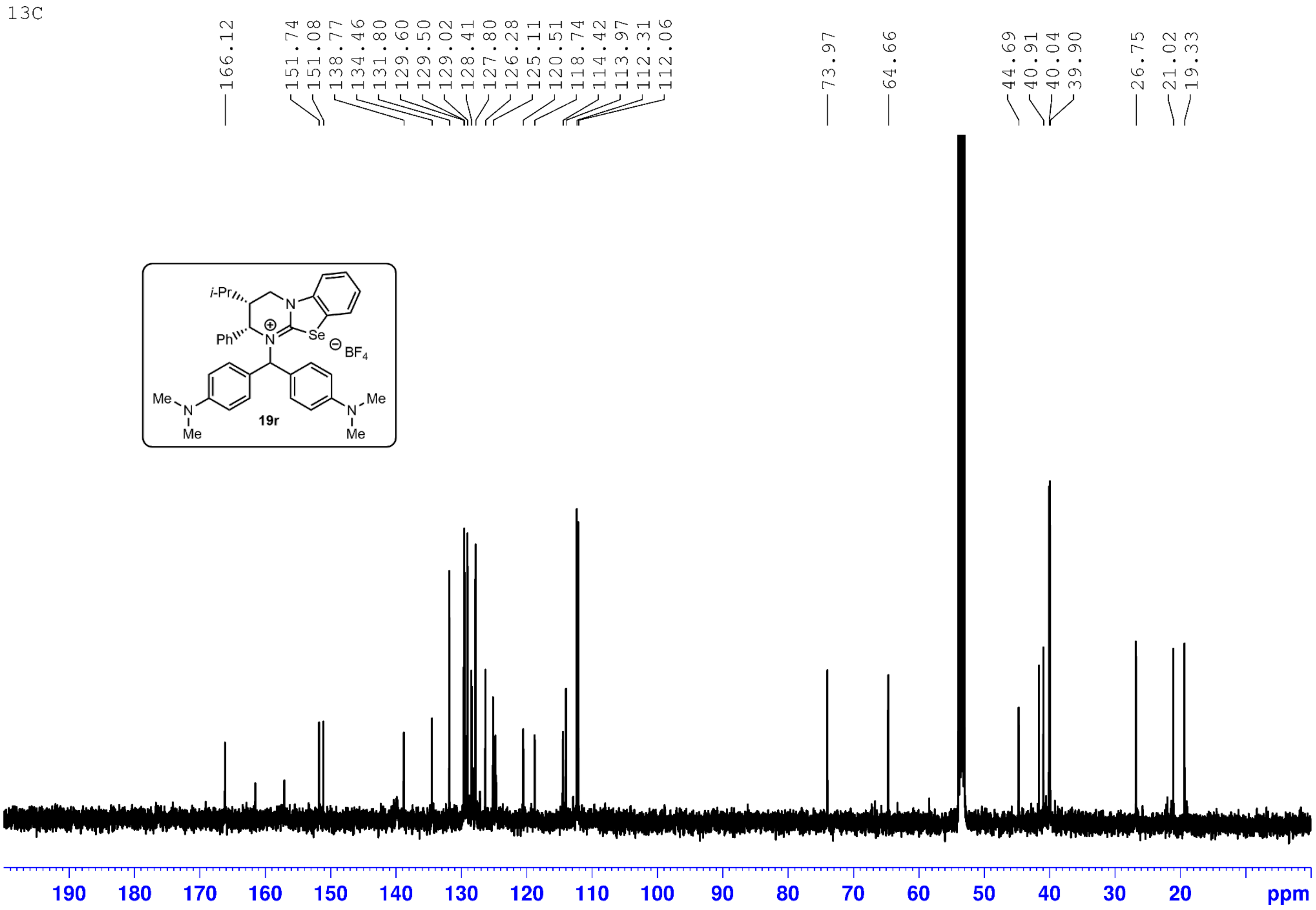
13C
166.12
151.74
151.08
138.77
134.46
131.80
129.60
129.50
129.02
128.41
127.80
126.28
125.11
120.51
118.74
114.42
113.97
112.31
112.06
73.97
64.66
44.69
40.91
40.04
39.90
26.75
21.02
19.33
i-Pr
Ph
N
Se
BF4
Me
19r
190
180
170
160
150
140
130
120
110
100
90
80
70
60
50
40
30
20
ppm

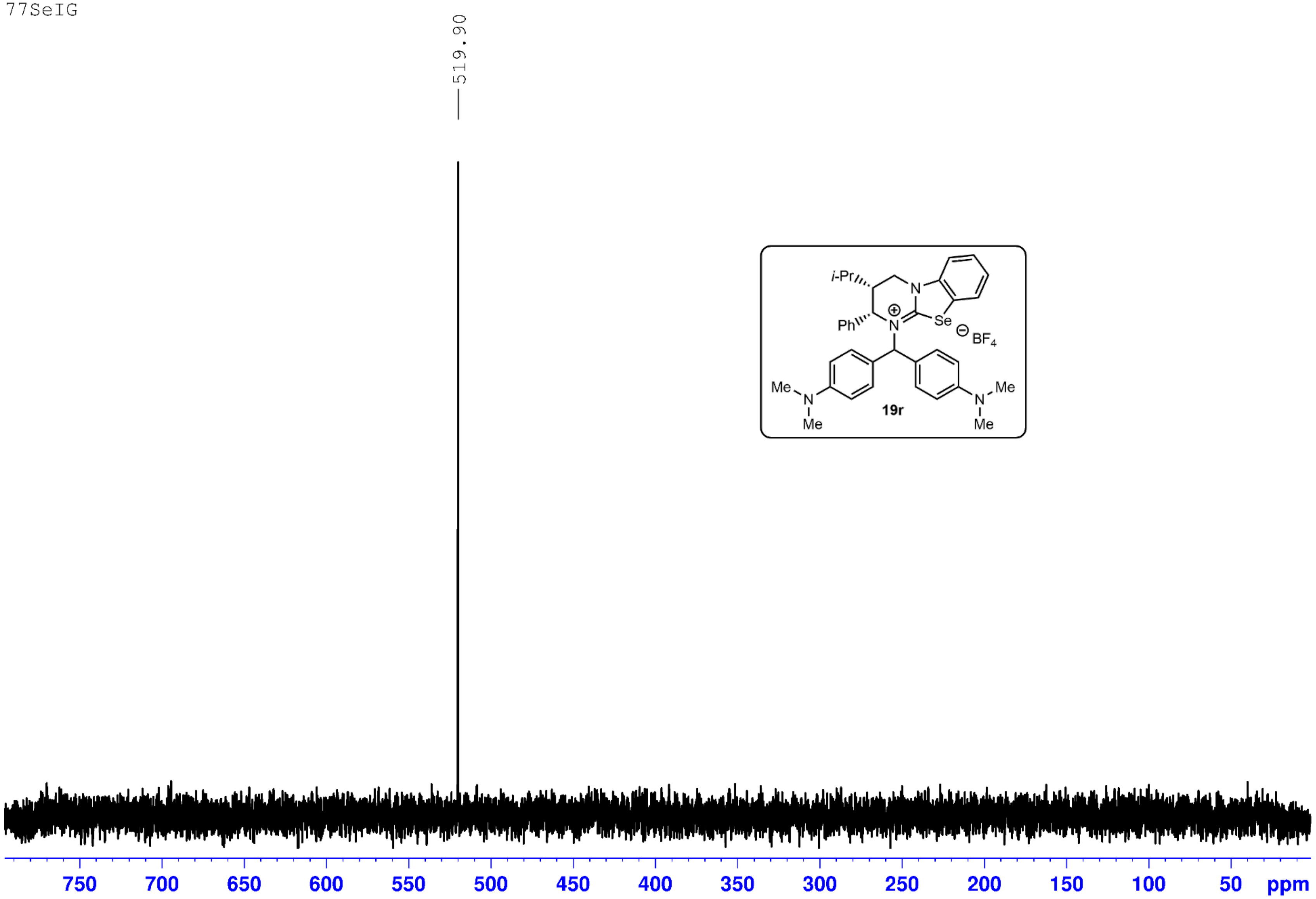
77SeIG
519.90
i-Pr
Ph
N
N
Se
BF4
Me
N
Me
19r
Me
N
Me
750 700 650 600 550 500 450 400 350 300 250 200 150 100 50 ppm

17. NMR Spectra: CSI Experiments for pKa determination in MeCN

IU3-ODHPB

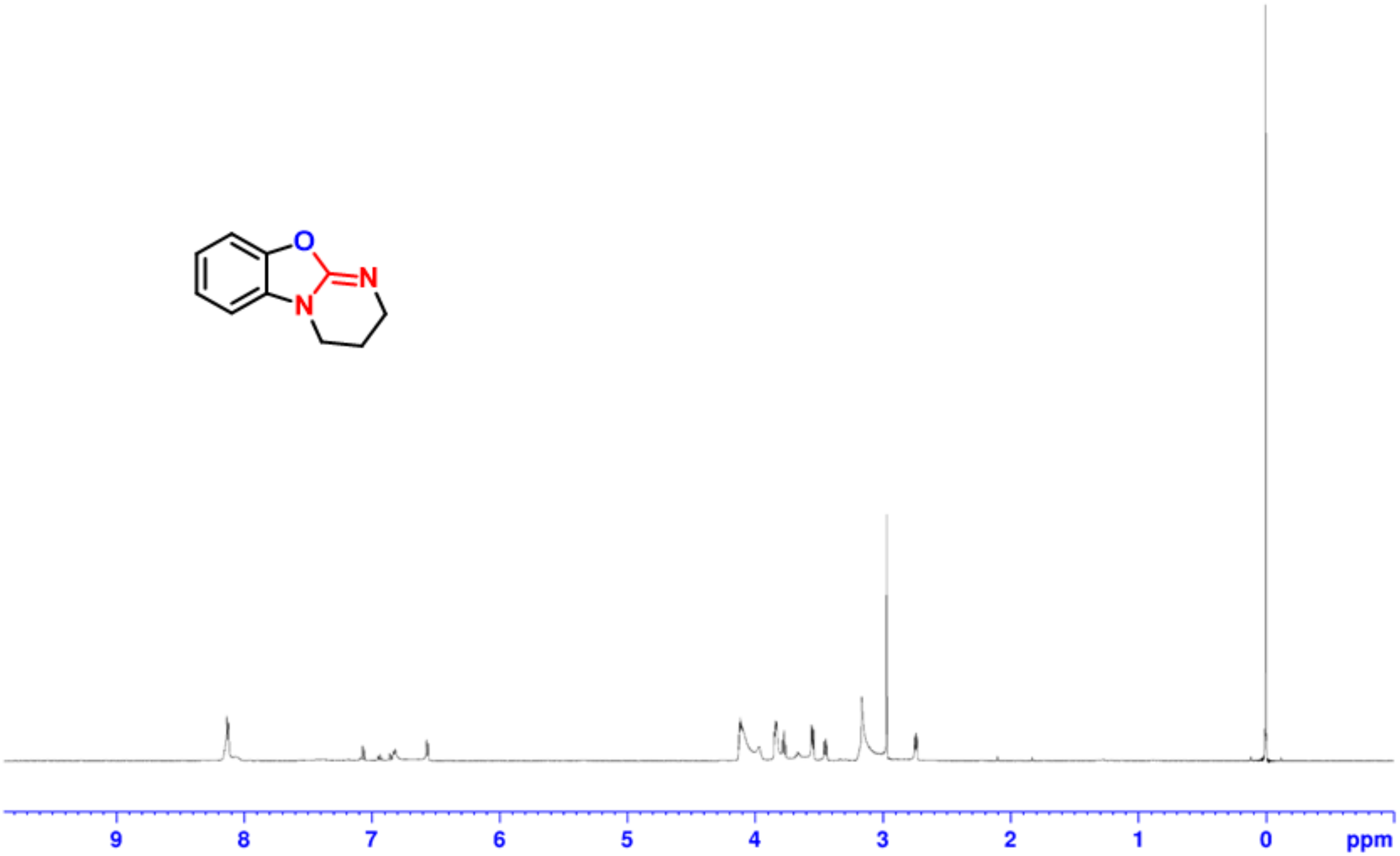

Figure S62: ^{1}H of CSI experiment over whole sample for IU3 - ODHPB in acetonitrile.

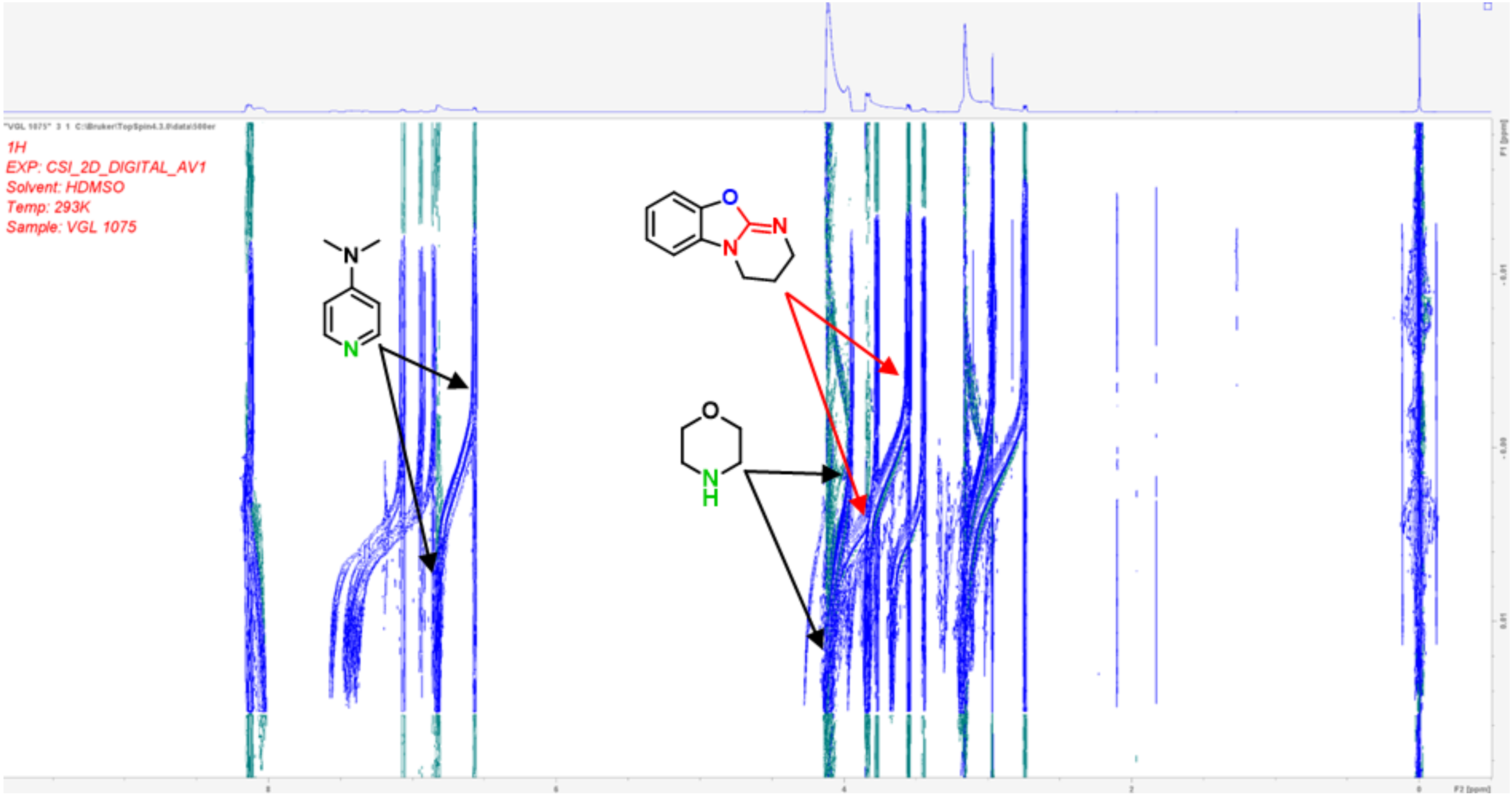

Figure S63: CSI experiment for IU3 - ODHPB in acetonitrile.

ISeU3 - SeDHPB

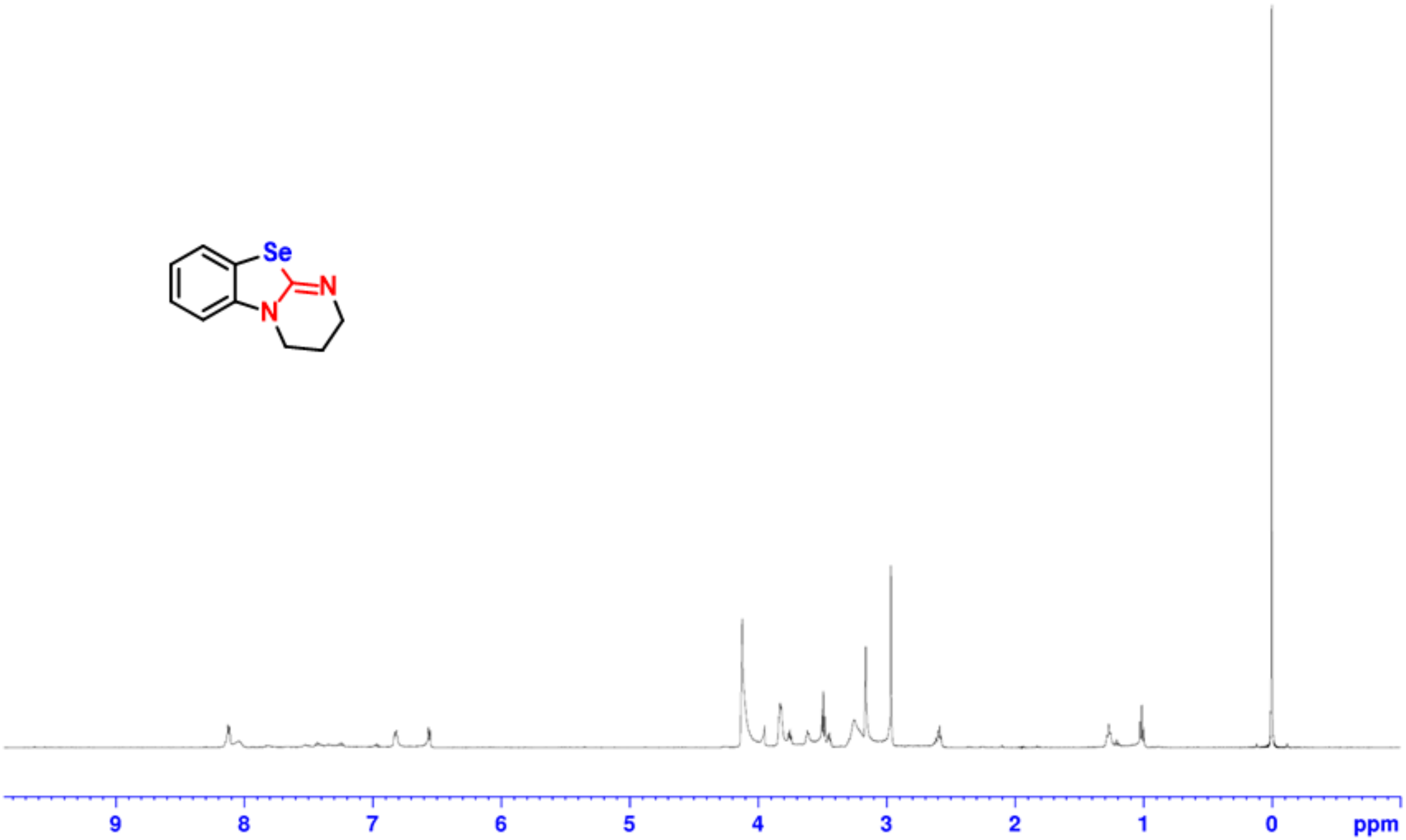

Figure S64: ^{1}H of CSI experiment over whole sample for ISeU3 - SeDHPB in acetonitrile.

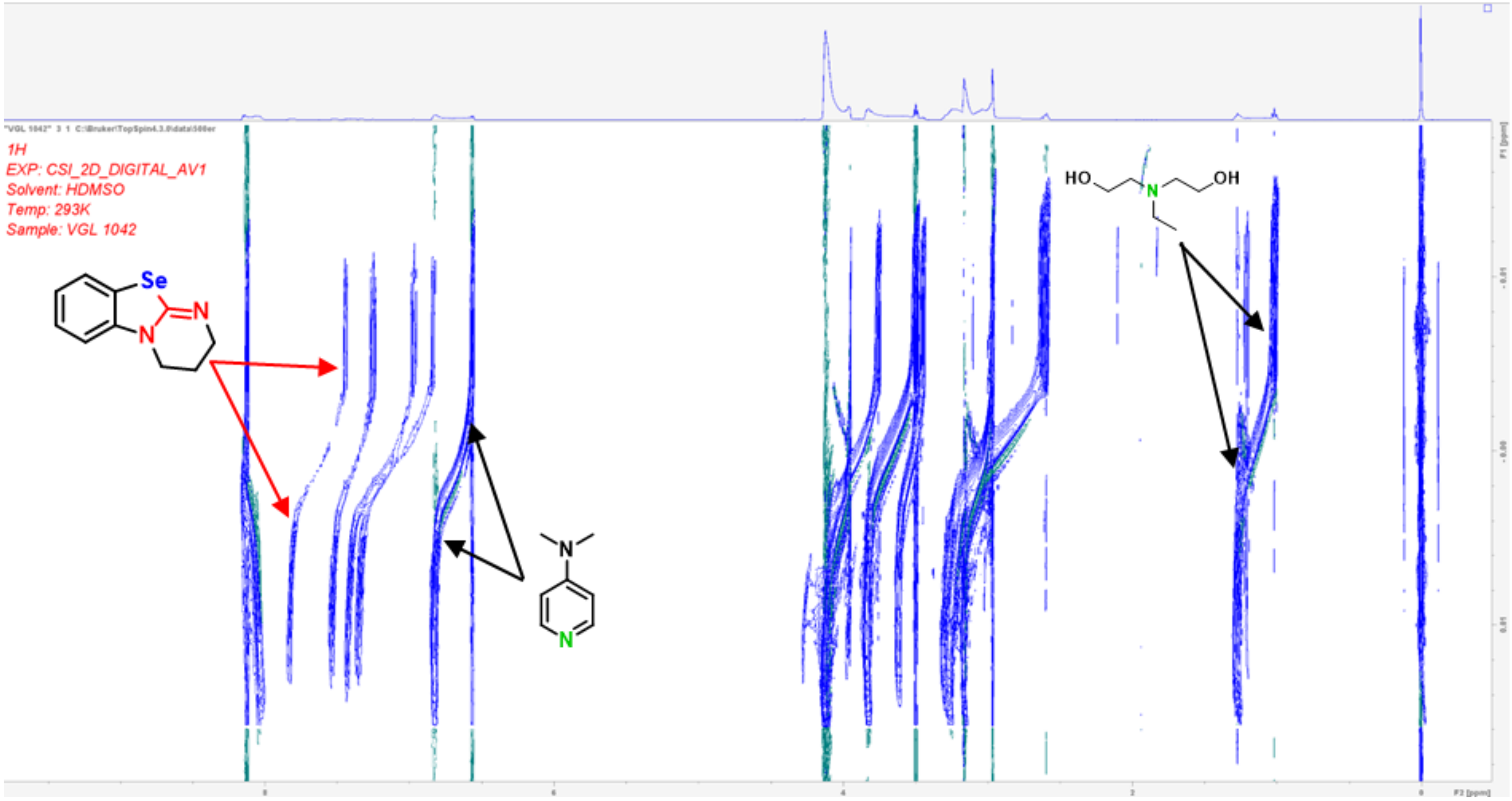

Figure S65: CSI experiment for ISeU3 - SeDHPB in acetonitrile.

ITeU3 - TeDHPB

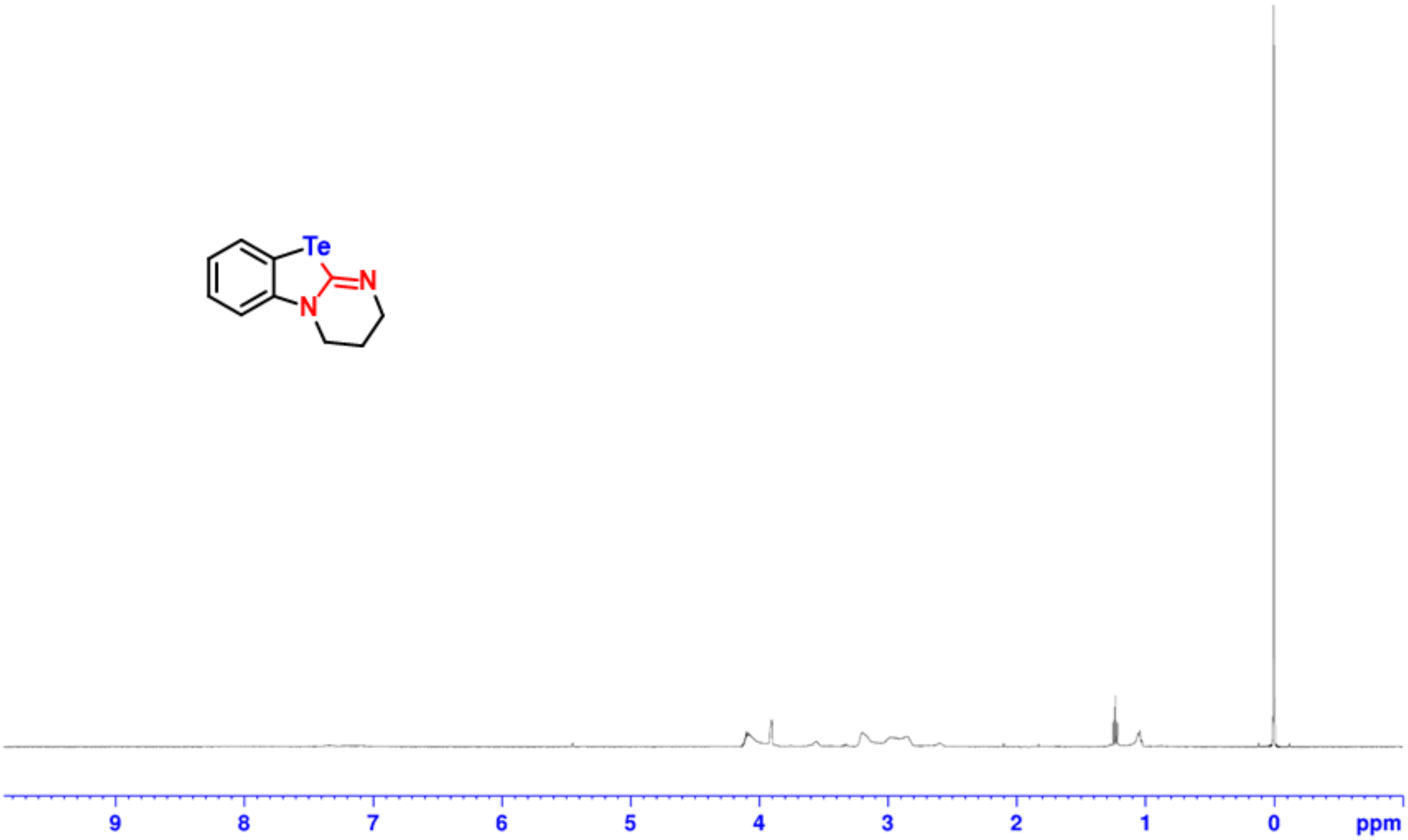

Figure S66: ^{1}H of CSI experiment over whole sample for ITeU3 - TeDHPB in acetonitrile.

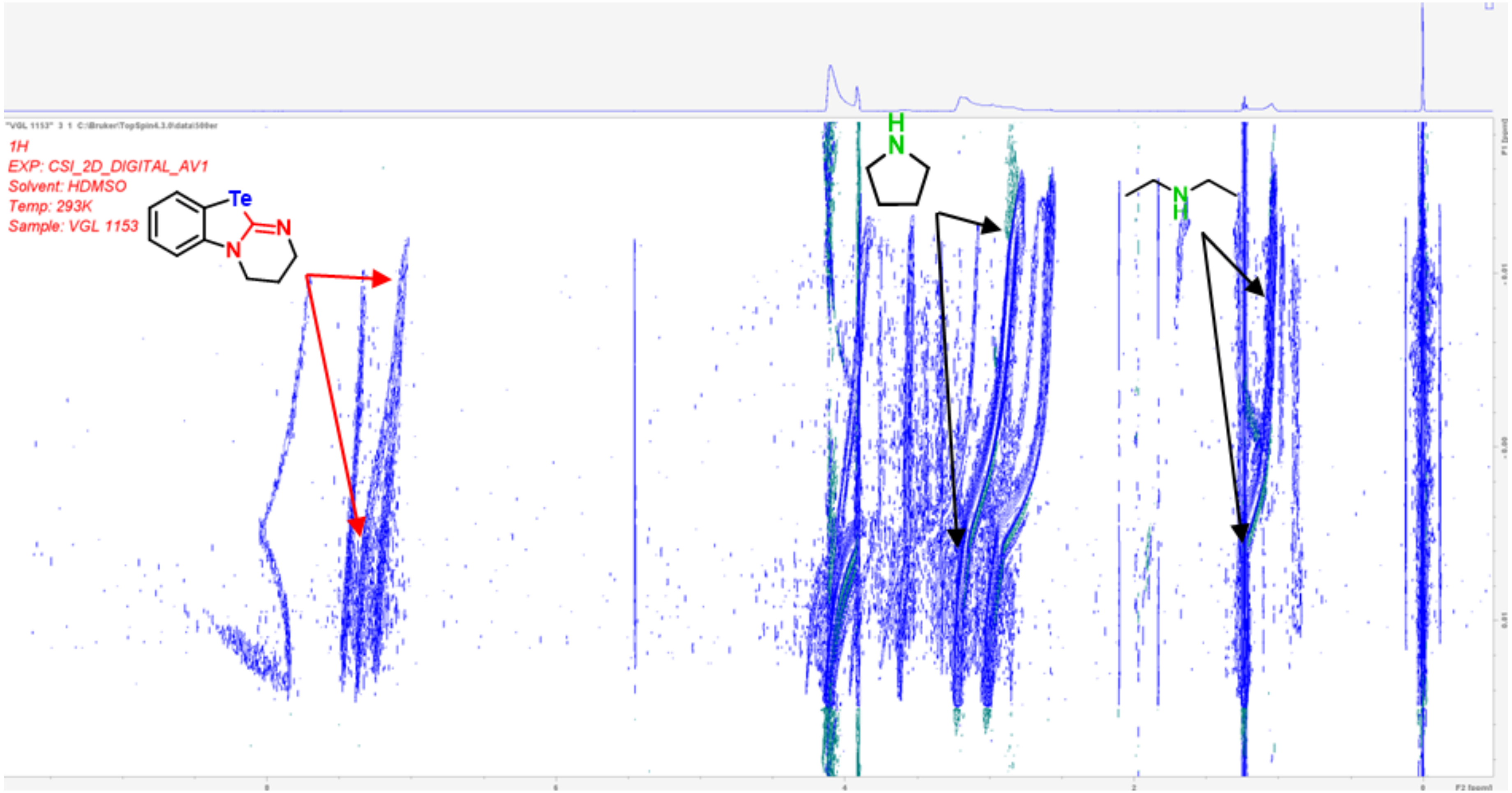

Figure S67: CSI experiment for ITeU3 - TeDHPB in acetonitrile.

ISeU2 - SeBTM

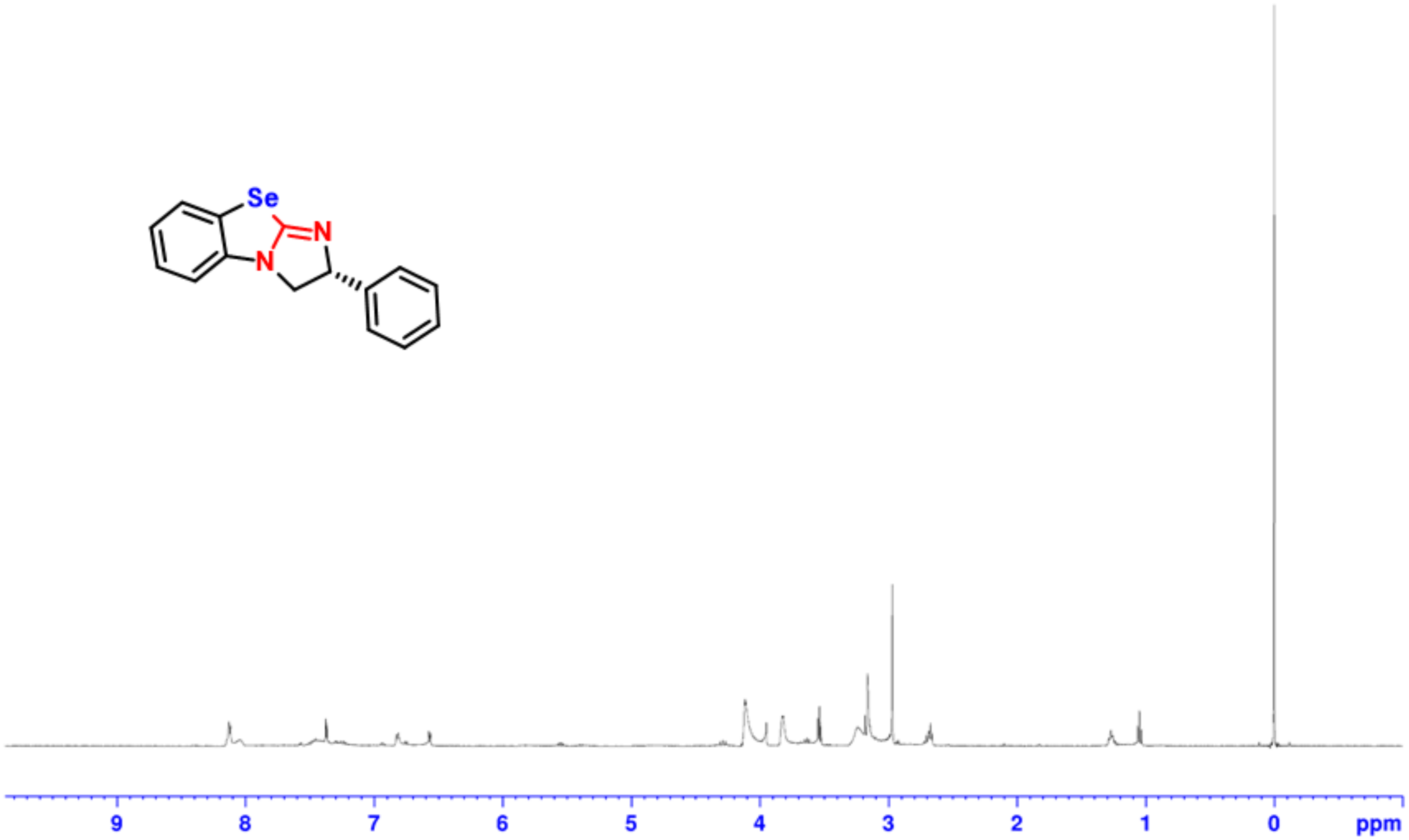

Figure S68: ^{1}H of CSI experiment over whole sample for ISeU2 - SeBTM in acetonitrile.

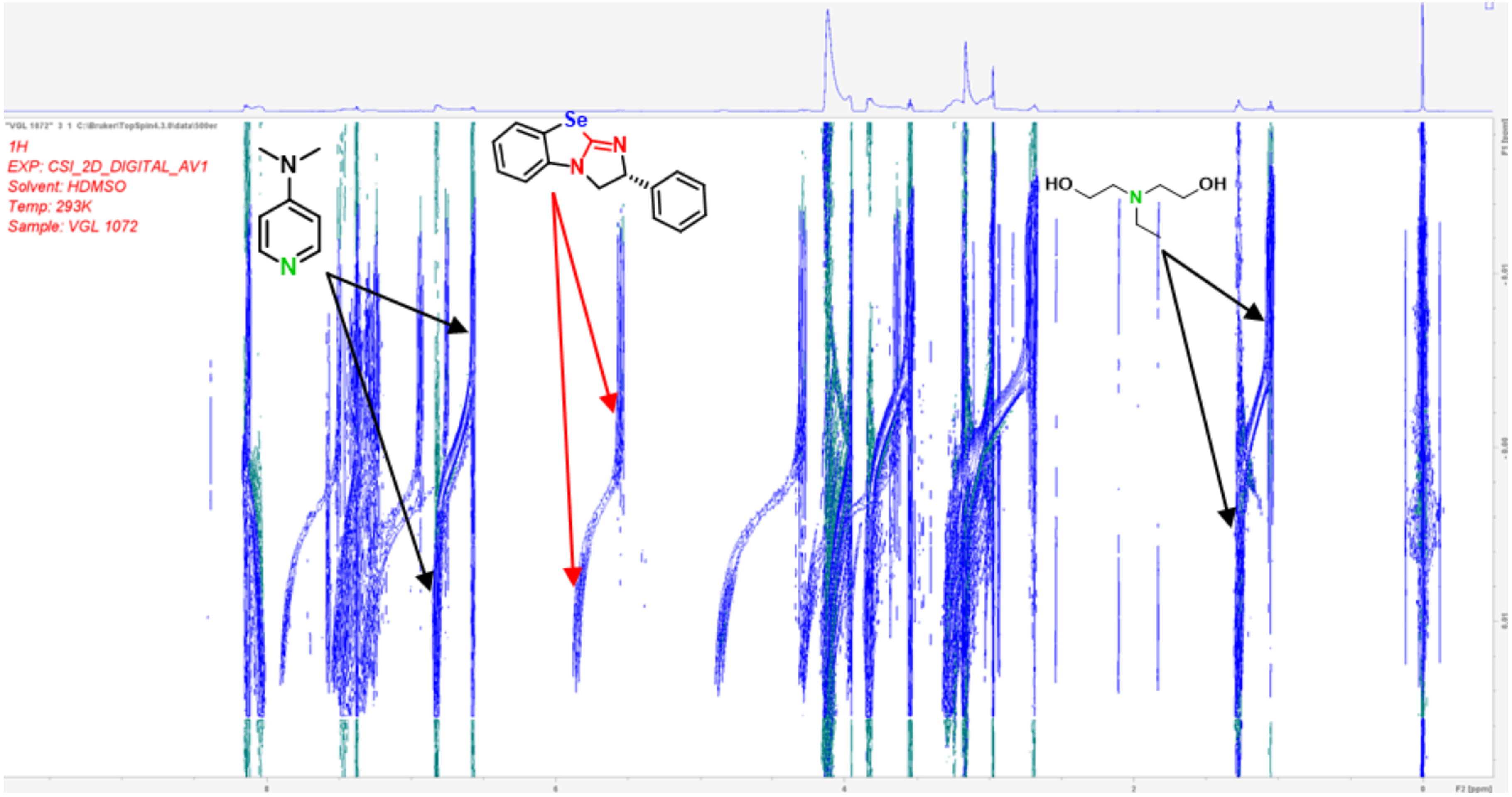

Figure S69: CSI experiment for ISeU2 - SeBTM in acetonitrile.

ITeU2 – TeBTM

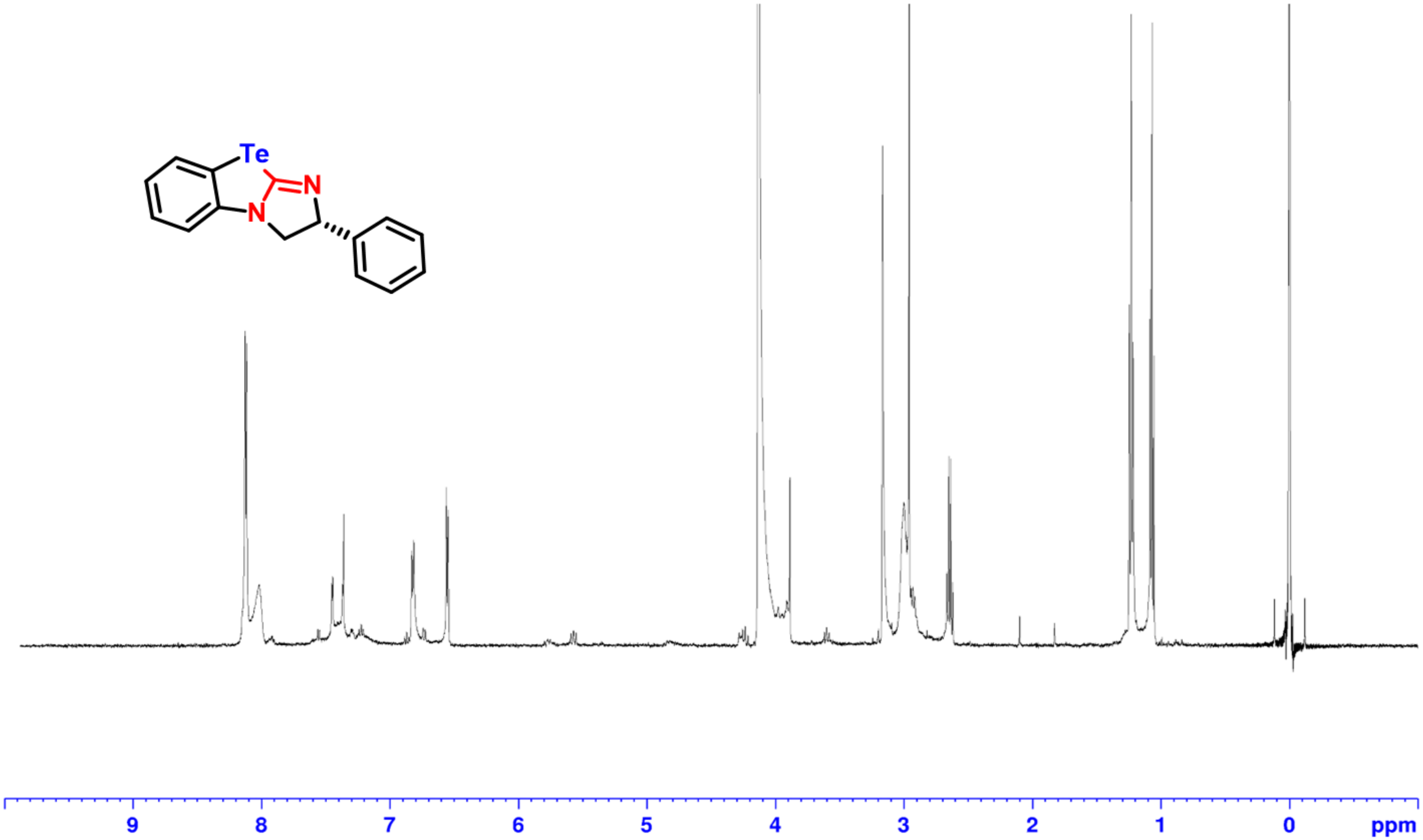

Figure S70: ^{1}H of CSI experiment over whole sample for ITeU2 - TeBTM in acetonitrile.

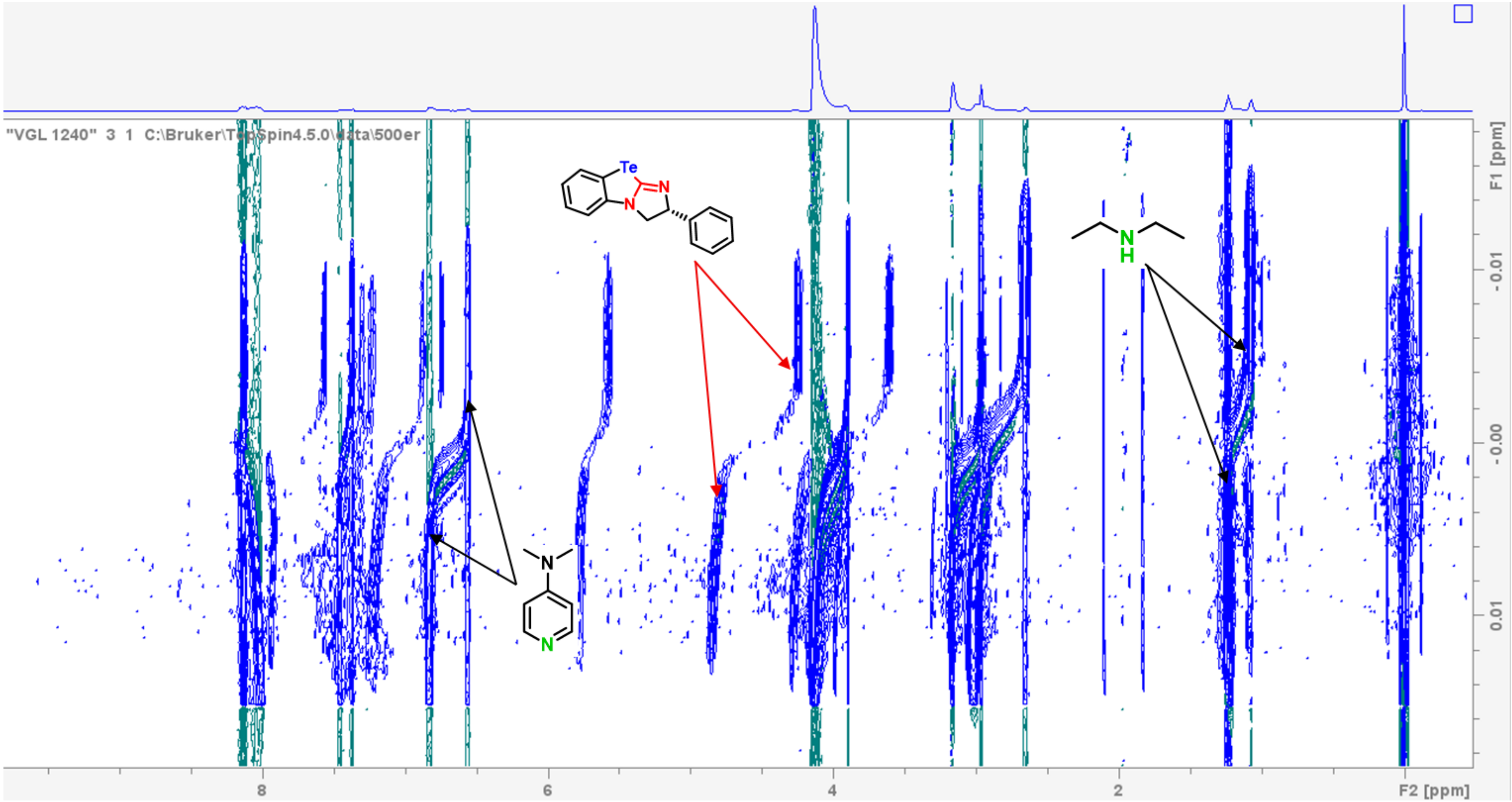

Figure S71: CSI experiment for ISeU2 - SeBTM in acetonitrile.

ISeU1 - SeTM

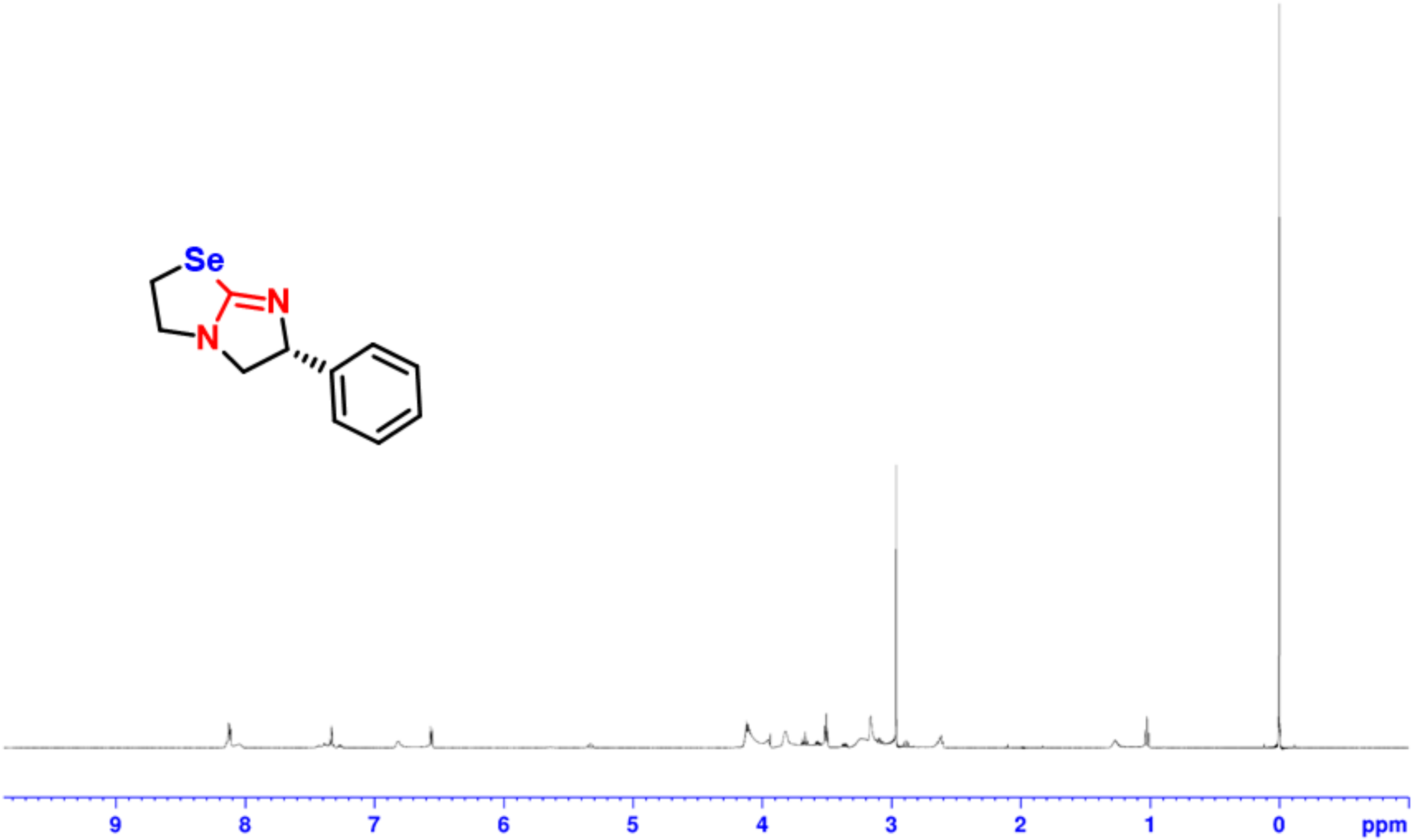

Figure S72: ^{1}H of CSI experiment over whole sample for ISeU1 - SeTM in acetonitrile.

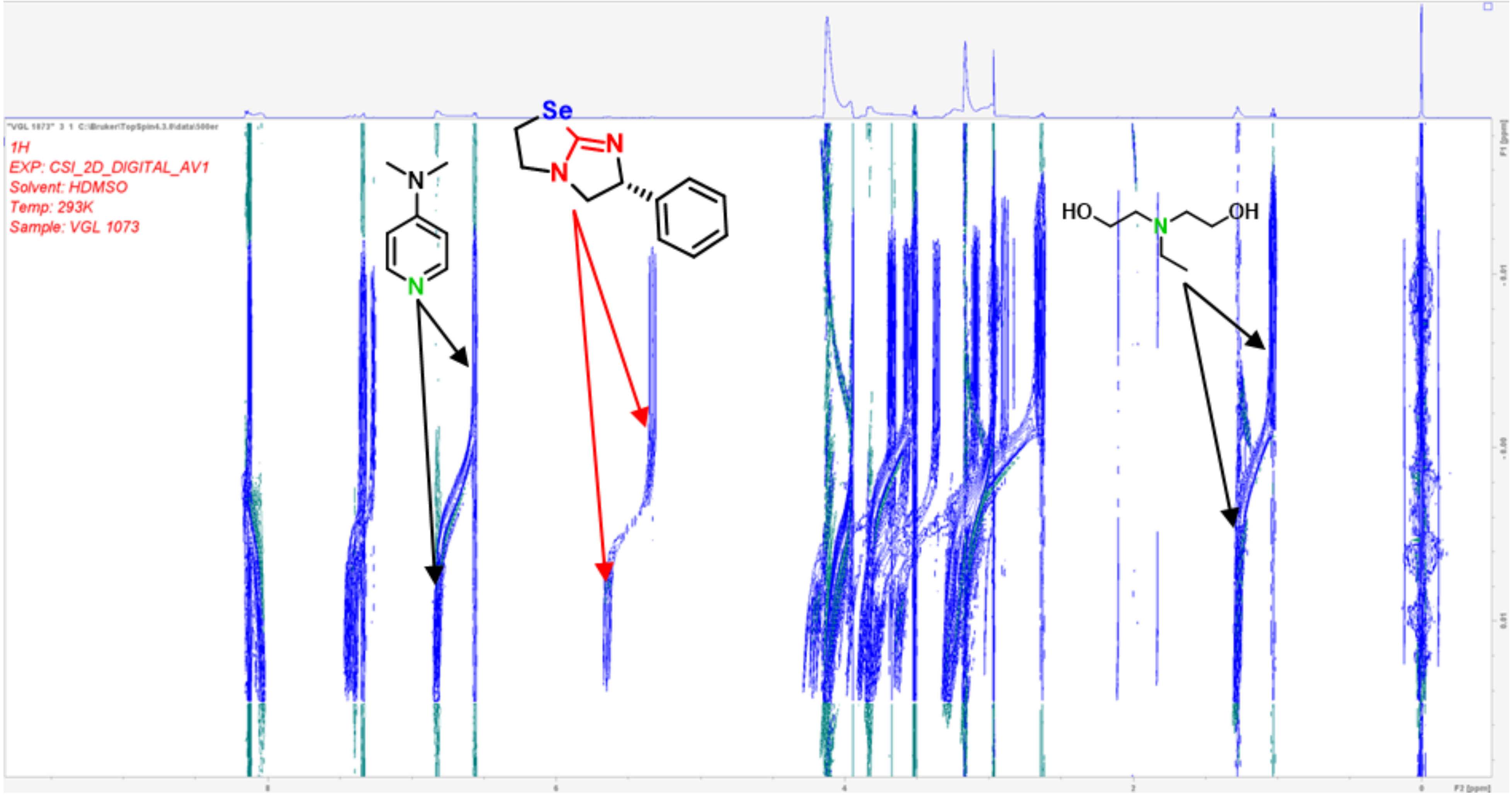

Figure S73: CSI experiment for ISeU1 - SeTM in acetonitrile.

IU5 - OHyperBTM

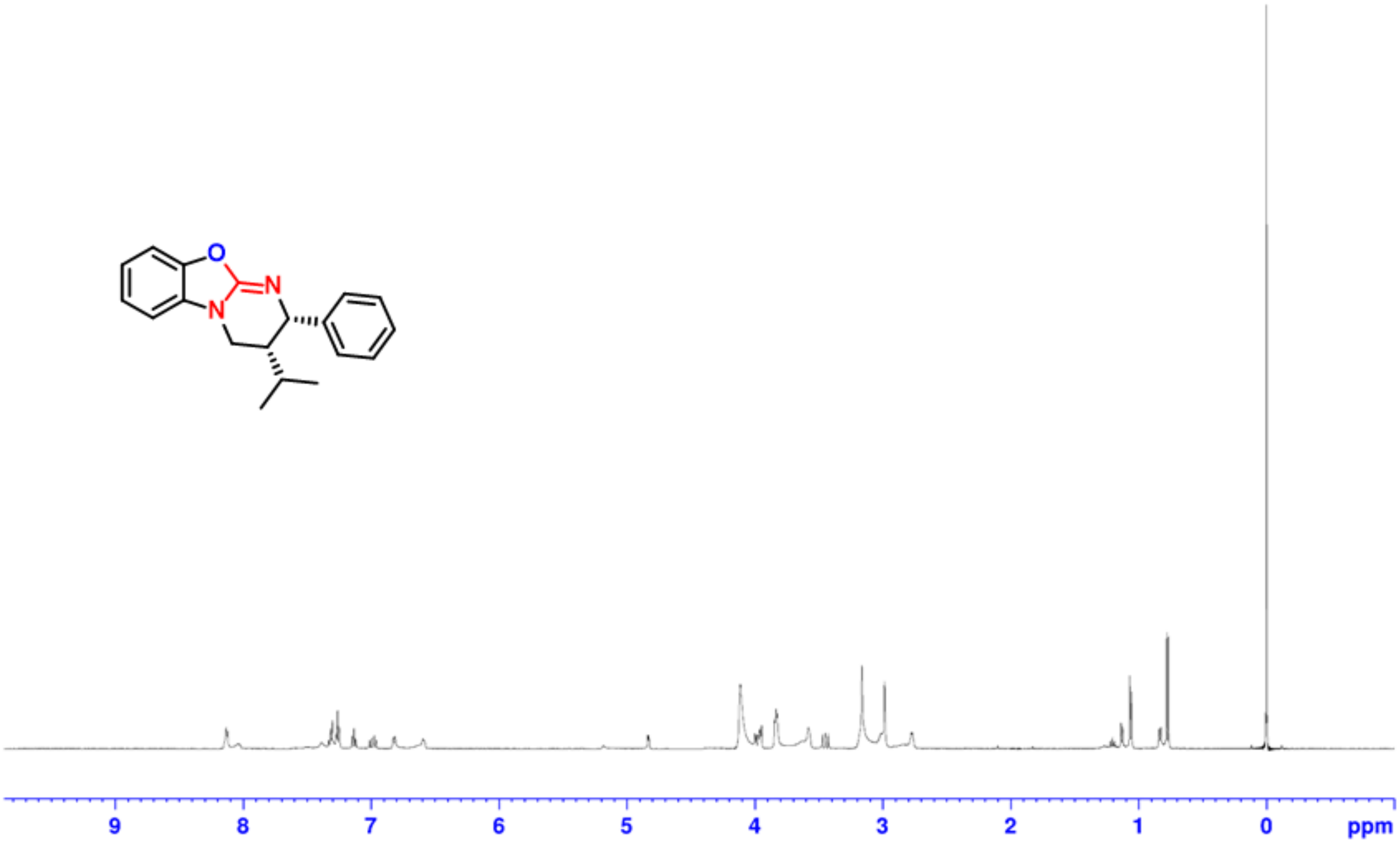

Figure S74: ^{1}H of CSI experiment over whole sample for IU5 - OHyperBTM in acetonitrile.

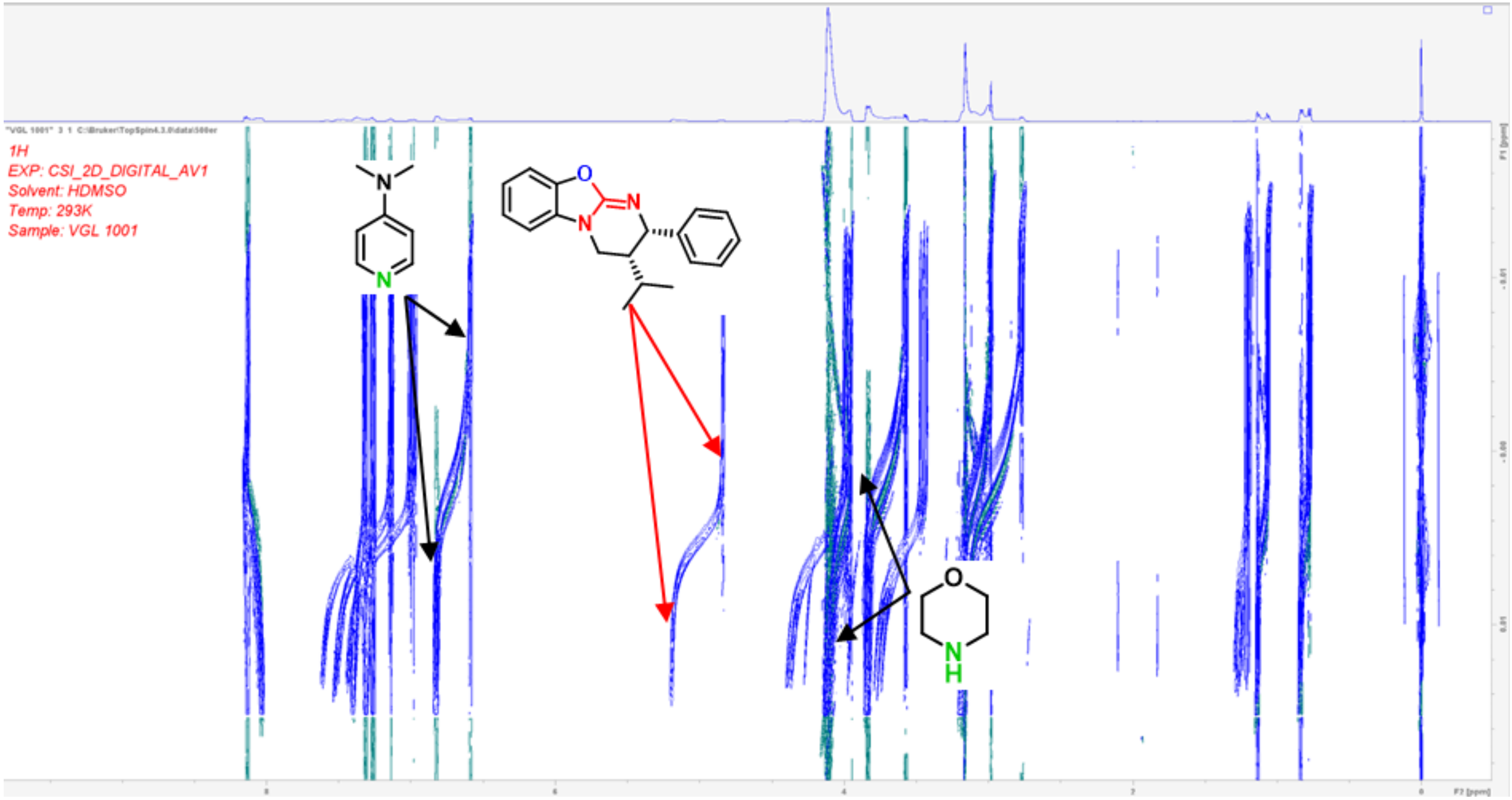

Figure S75: CSI experiment for IU5 - OHyperBTM in acetonitrile.

ISeU5 - SeHyperBTM

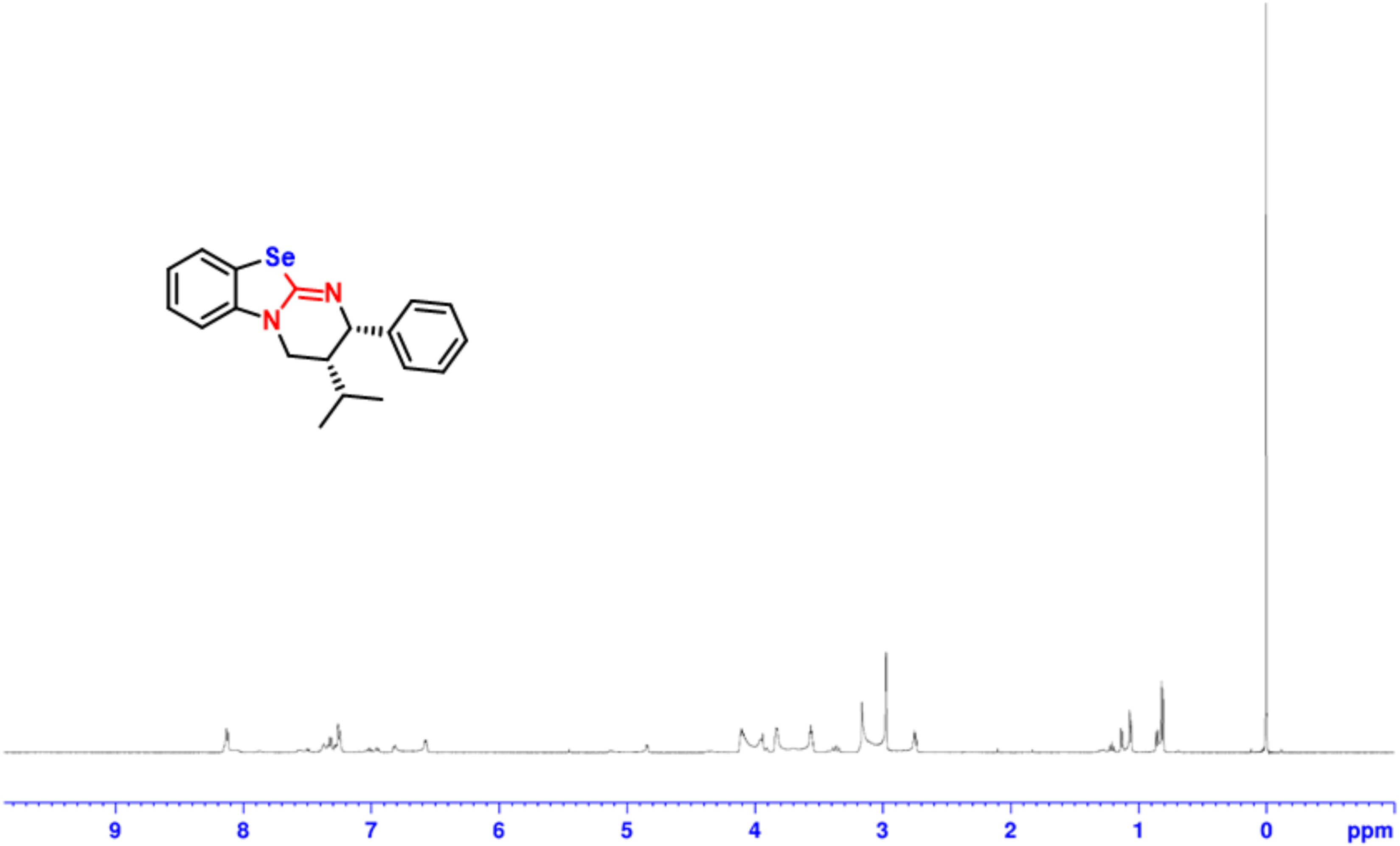

Figure S76: ^{1}H of CSI experiment over whole sample for ISeU5 - SeHyperBTM in acetonitrile.

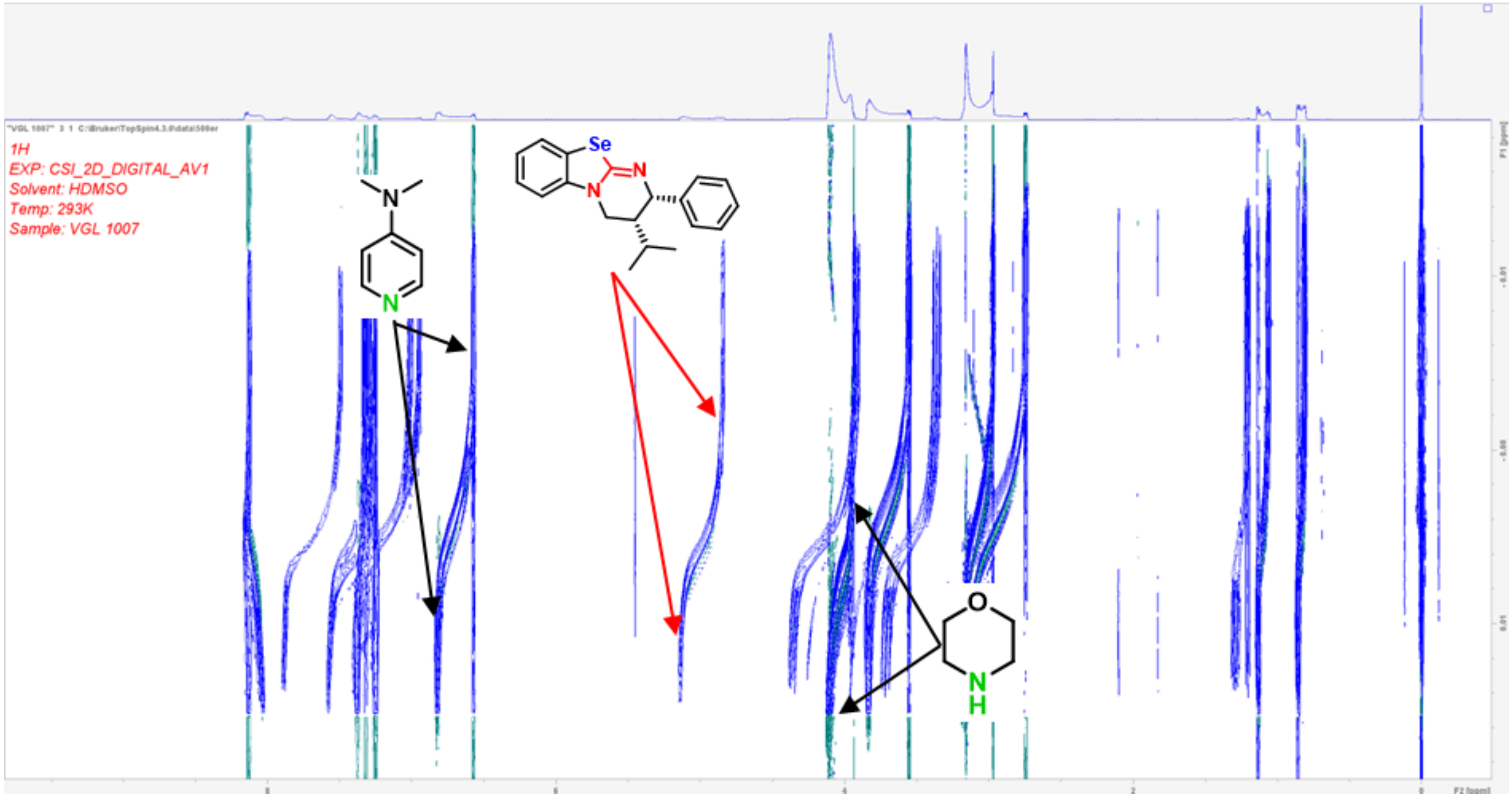

Figure S77: CSI experiment for ISeU5 - SeHyperBTM in acetonitrile.

18. NMR Spectra: CSI Experiments for pKa determination in DMSO

IU3 - ODHPB

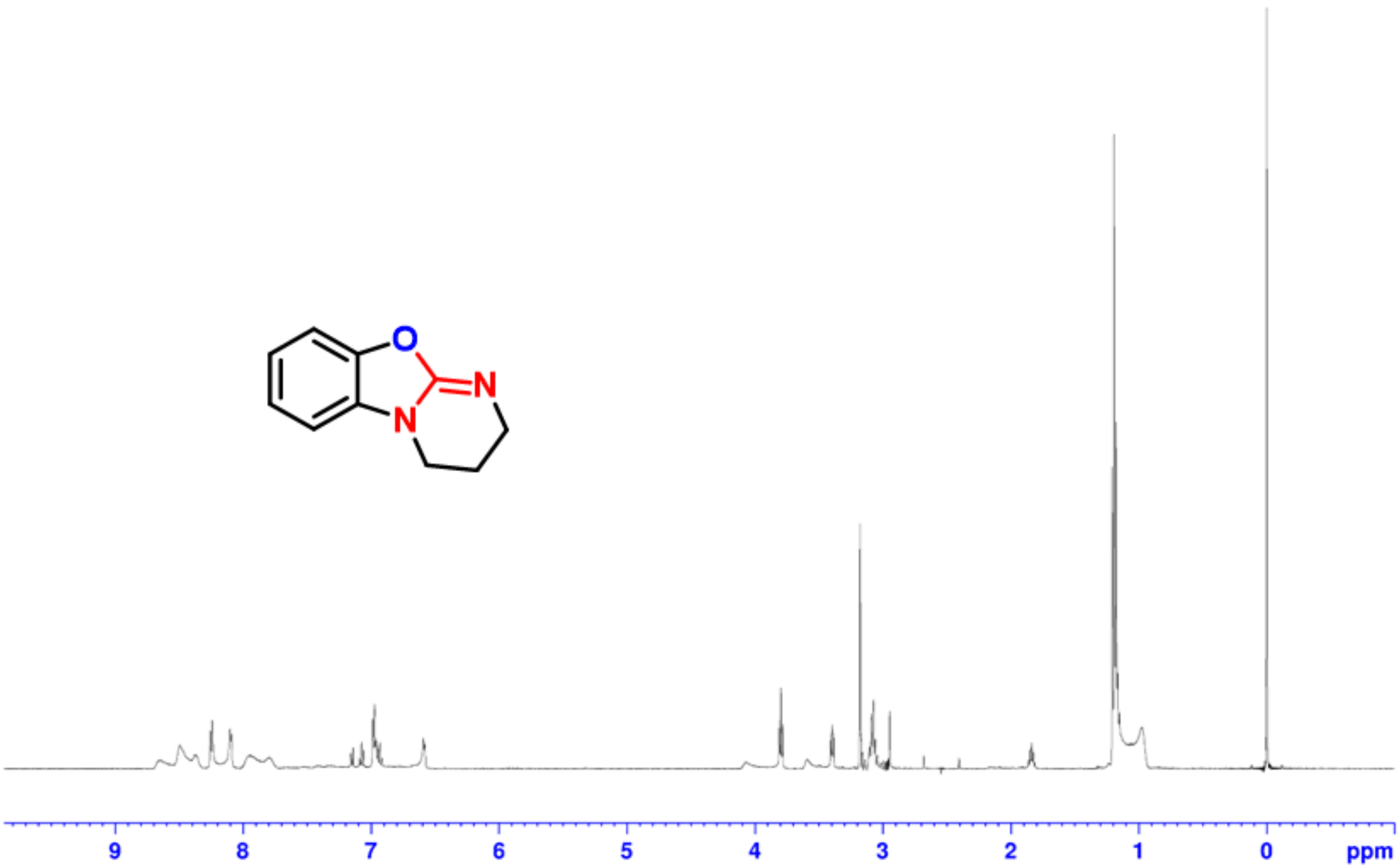

Figure S78: ^{1}H experiment over whole sample of CSI experiment of IU3 - ODHPB in DMSO.

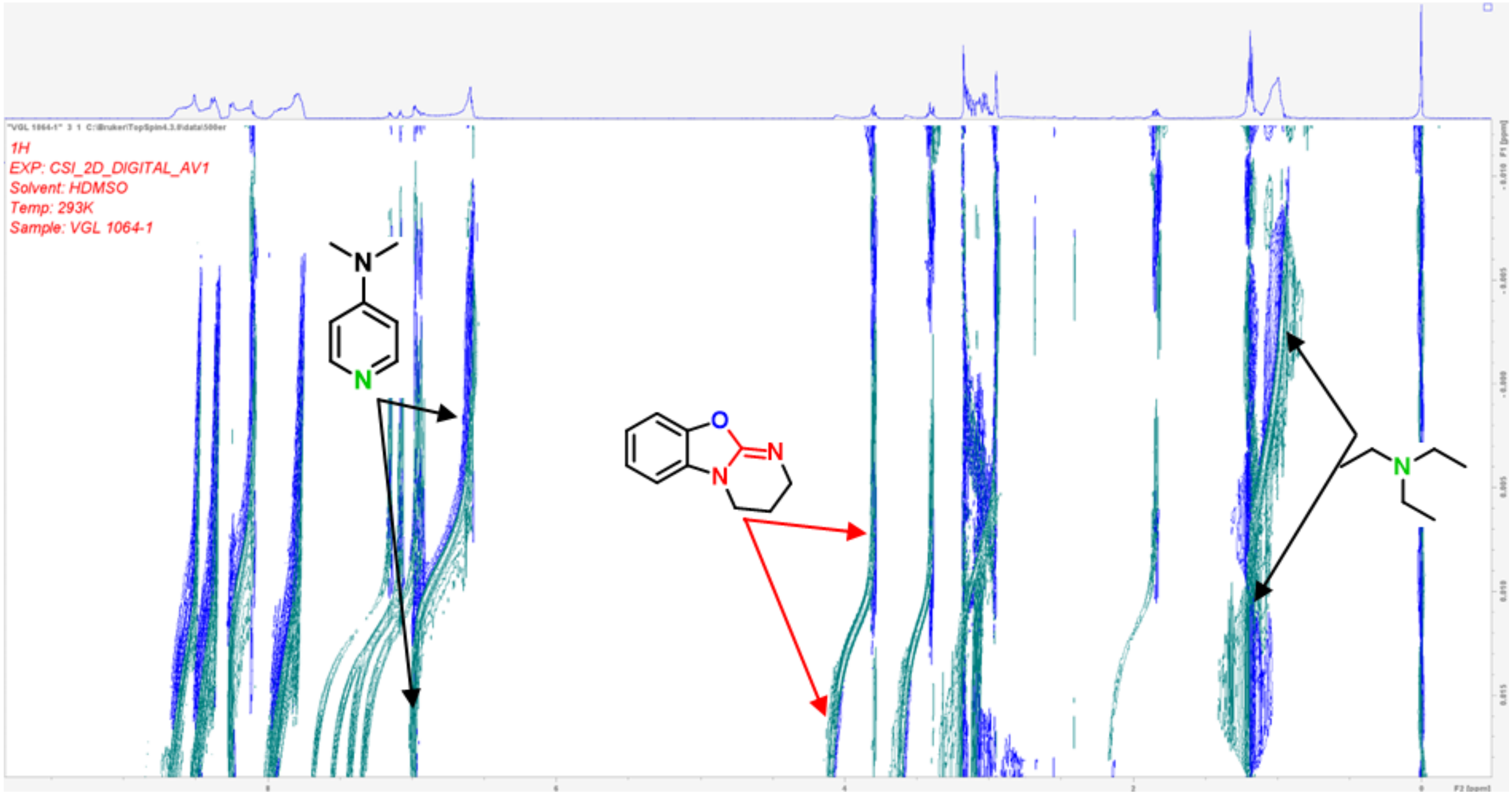

Figure S79: CSI experiment for IU3 - ODHPB in DMSO.

ISeU3 - SeDHPB

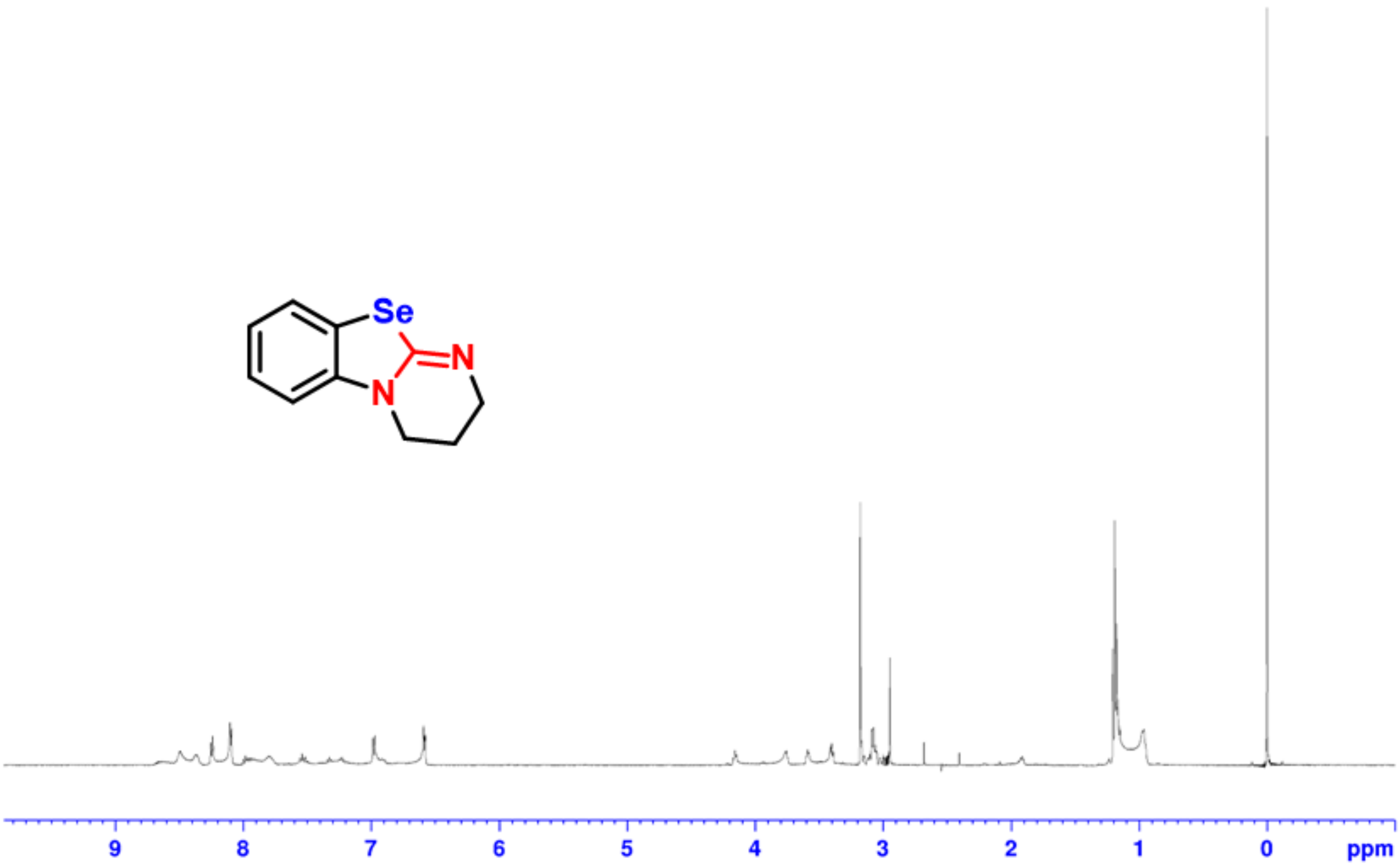

Figure S80: ^{1}H experiment over whole sample of CSI experiment of ISeU3 - SeDHPB in DMSO.

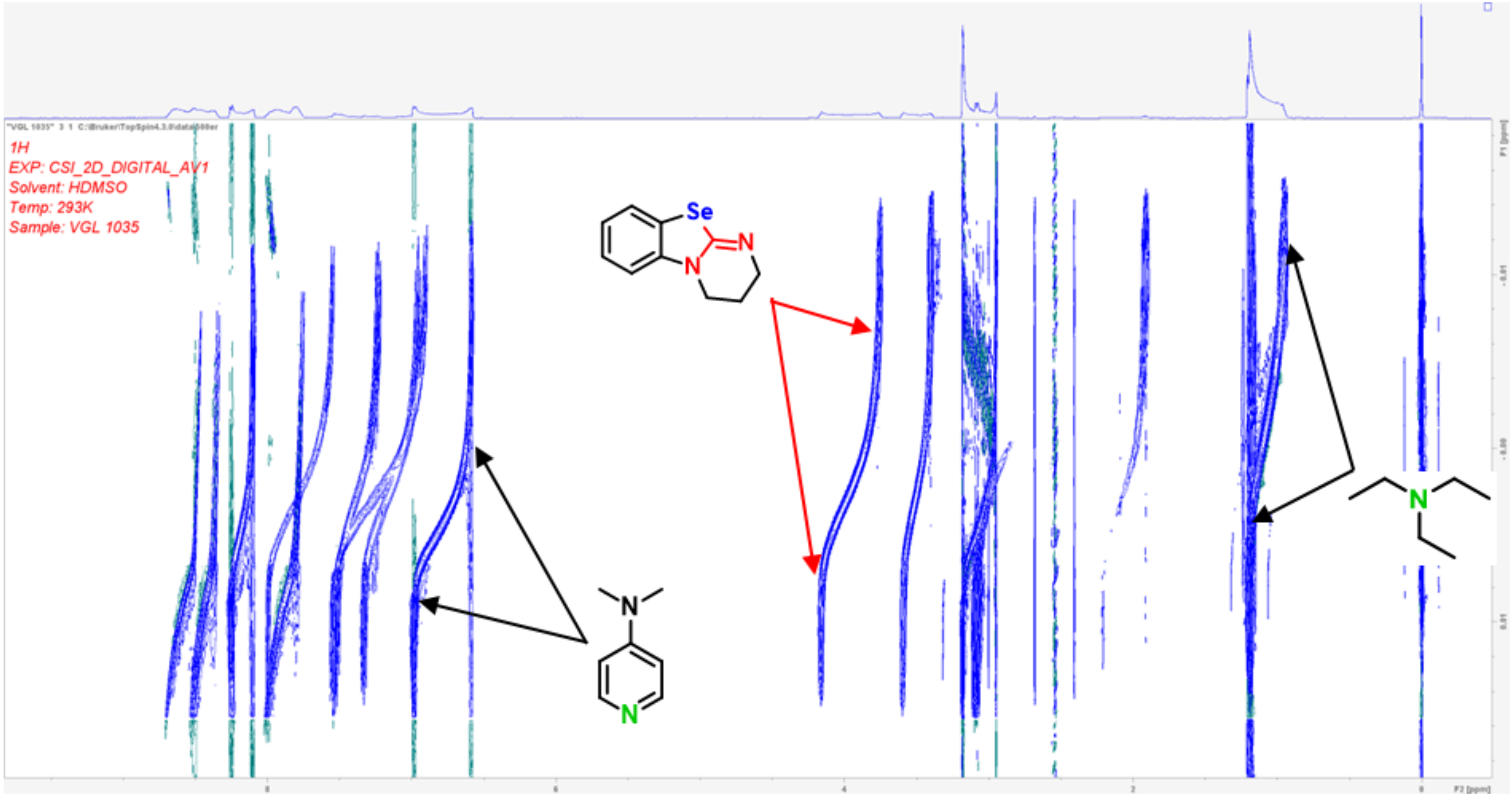

Figure S81: CSI experiment for ISeU3 - SeDHPB in DMSO.

ITeU3 - TeDHPB

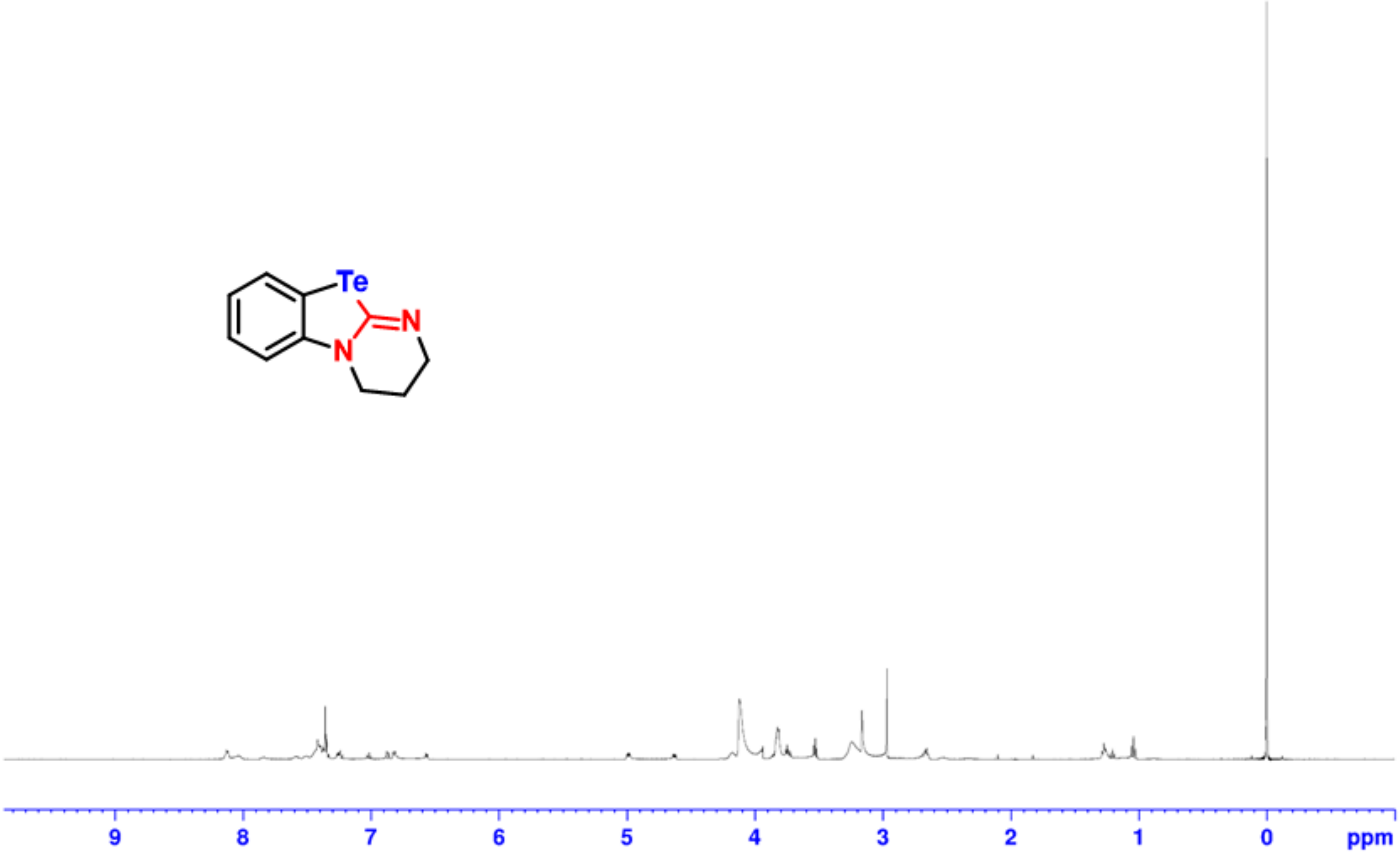

Figure S82: ^{1}H experiment over whole sample of CSI experiment of ITeU3 - TeDHPB in DMSO.

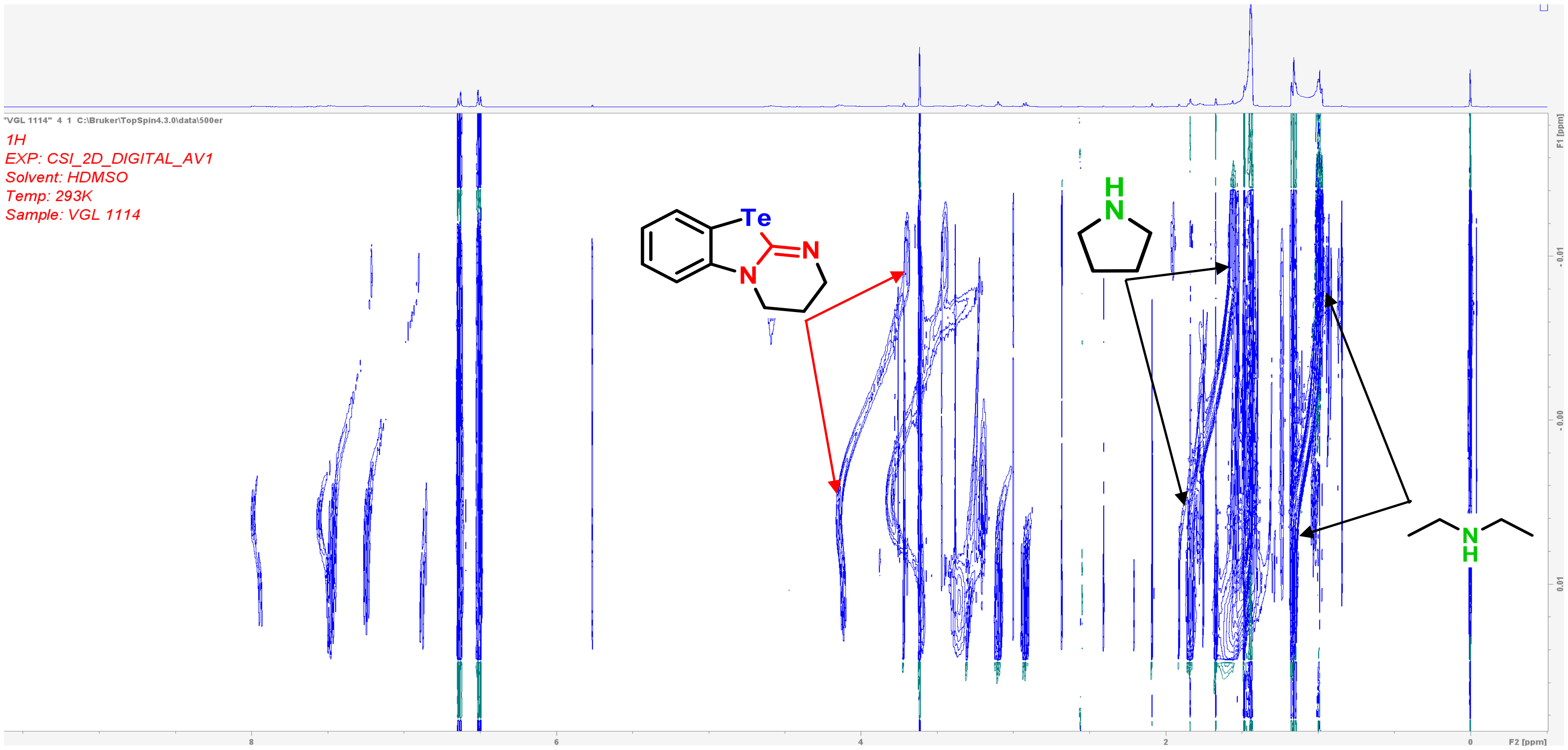

Figure S83: CSI experiment for ITeU3 - TeDHPB in DMSO.

ISeU2 - SeBTM

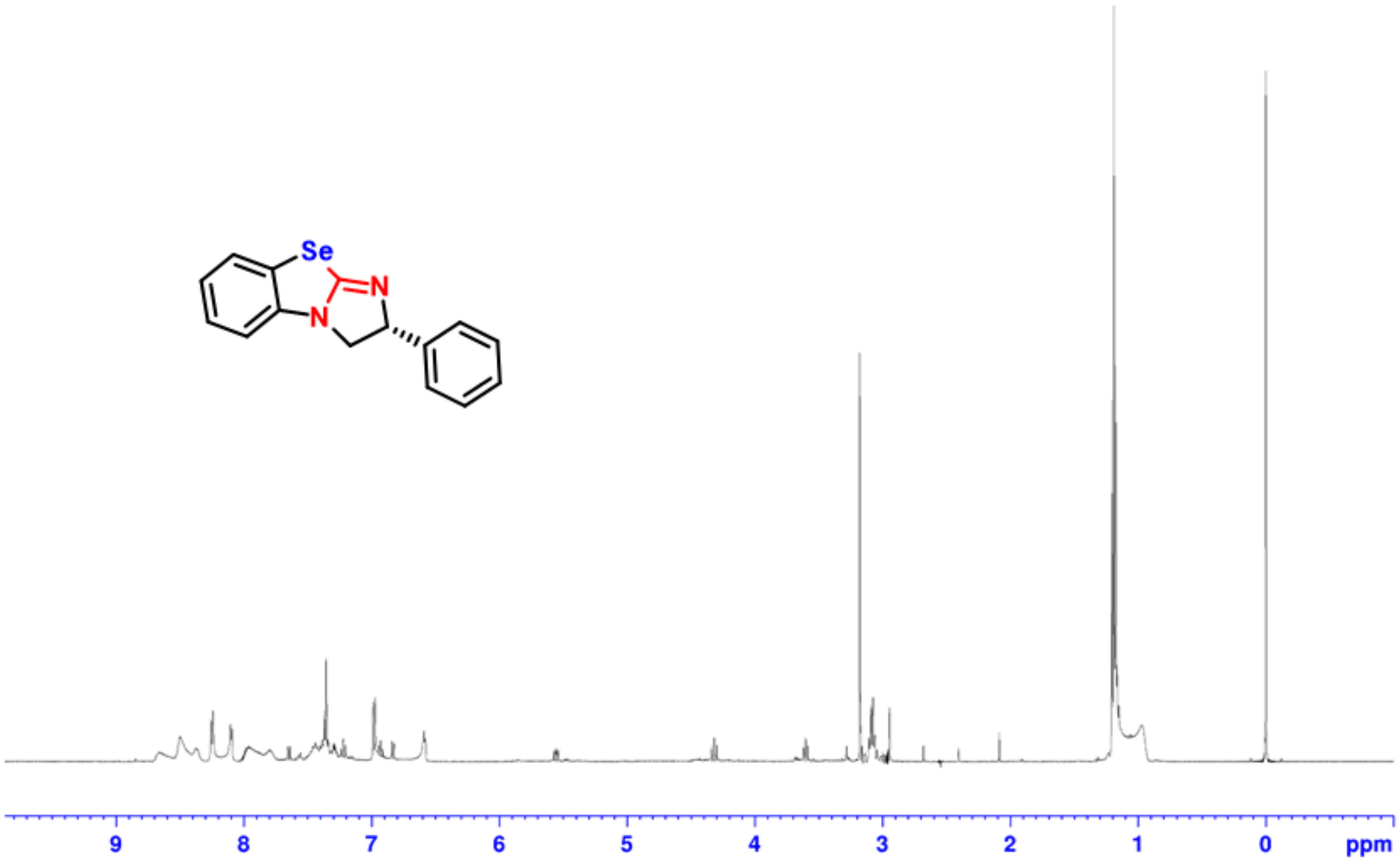

Figure S84: ^{1}H experiment over whole sample of CSI experiment of ISeU2 - SeBTM in DMSO.

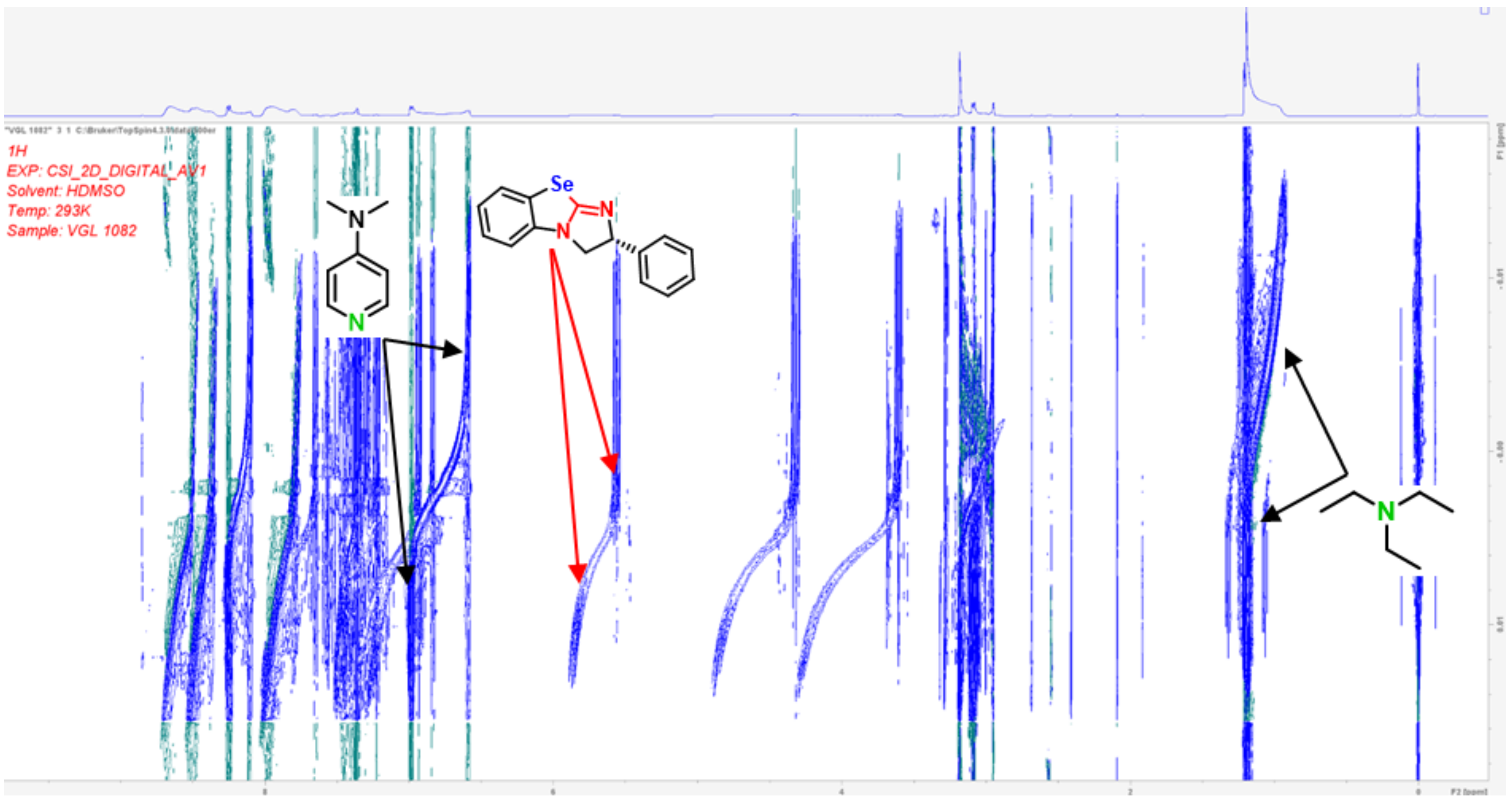

Figure S85: CSI experiment for ISeU2 - SeBTM in DMSO.

ITeU2 - TeBTM

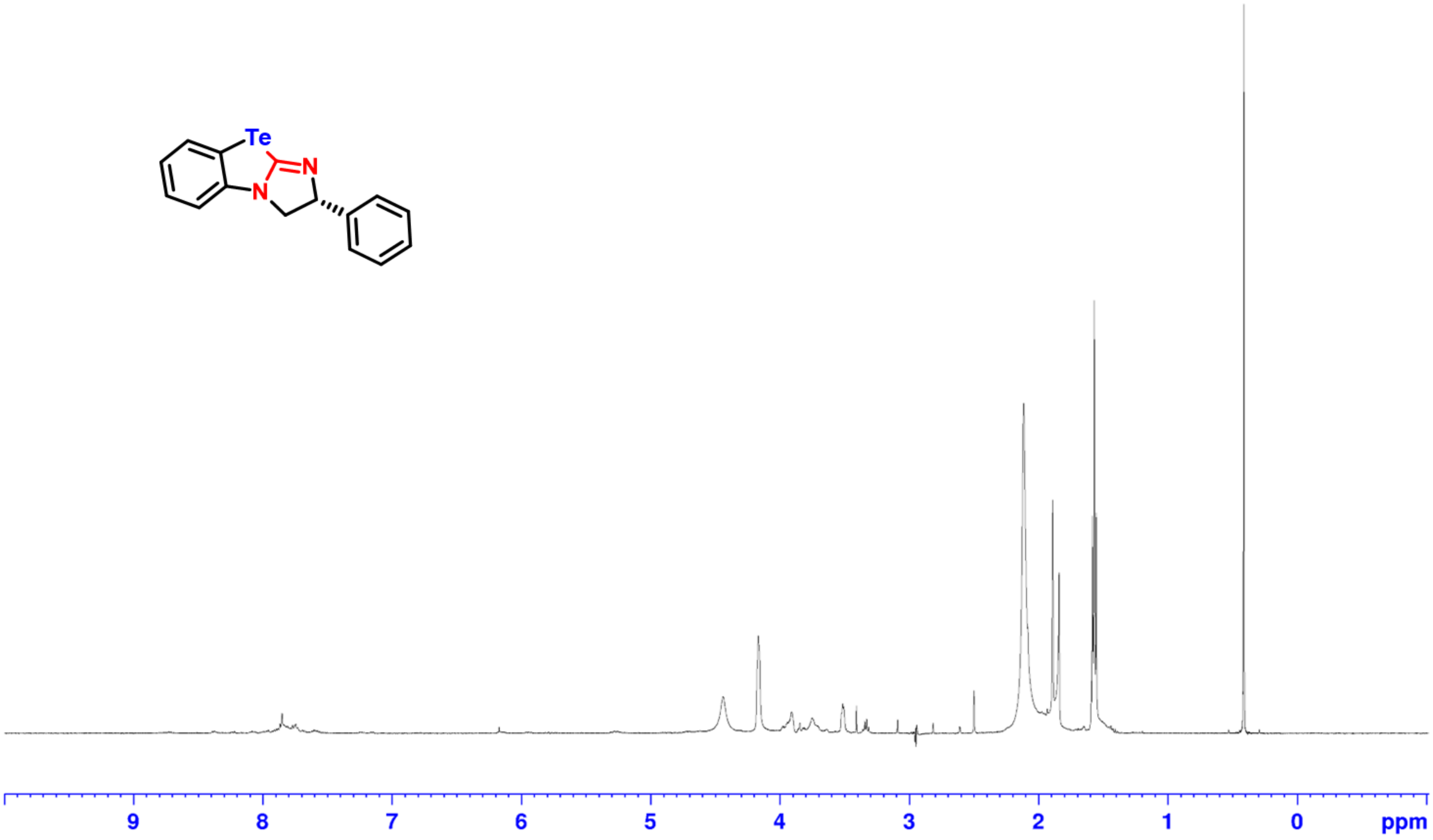

Figure S86: ^{1}H experiment over whole sample of CSI experiment of ITeU2 - TeBTM in DMSO.

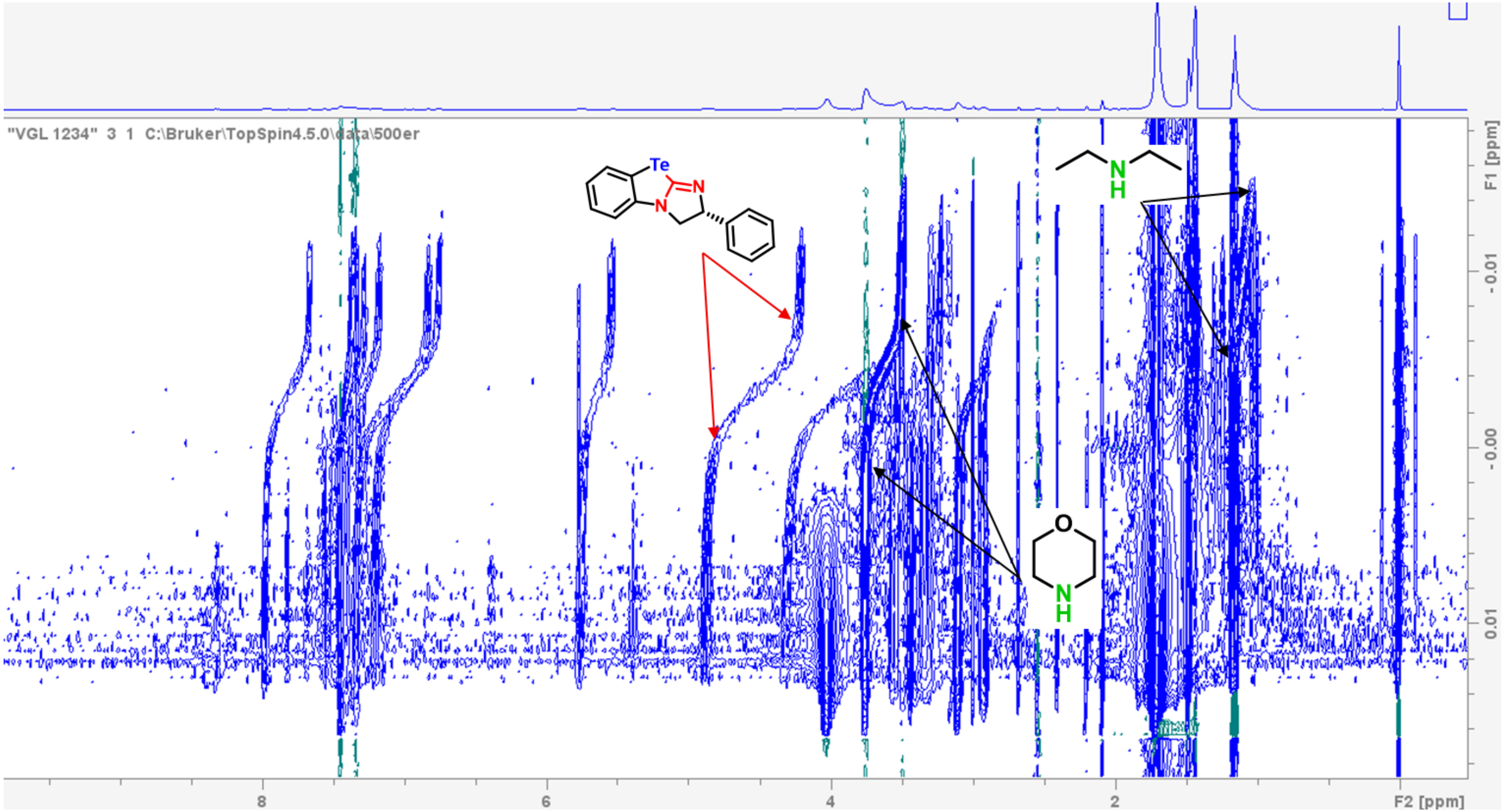

Figure S87: CSI experiment for ITeU2 - TeBTM in DMSO.

ISeU1 - SeTM

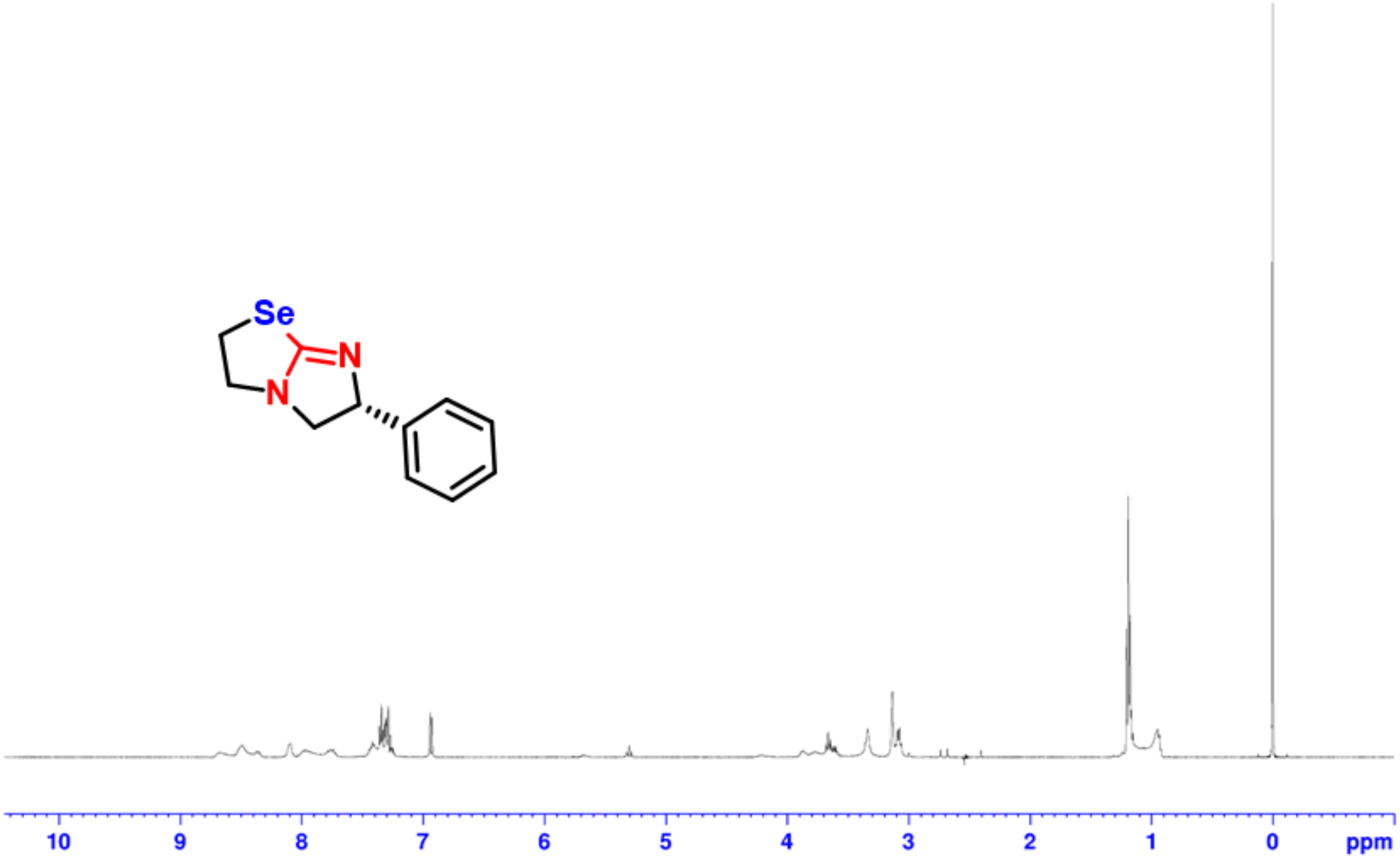

Figure S88: ^{1}H experiment over whole sample of CSI experiment of ISeU1 - SeTM in DMSO.

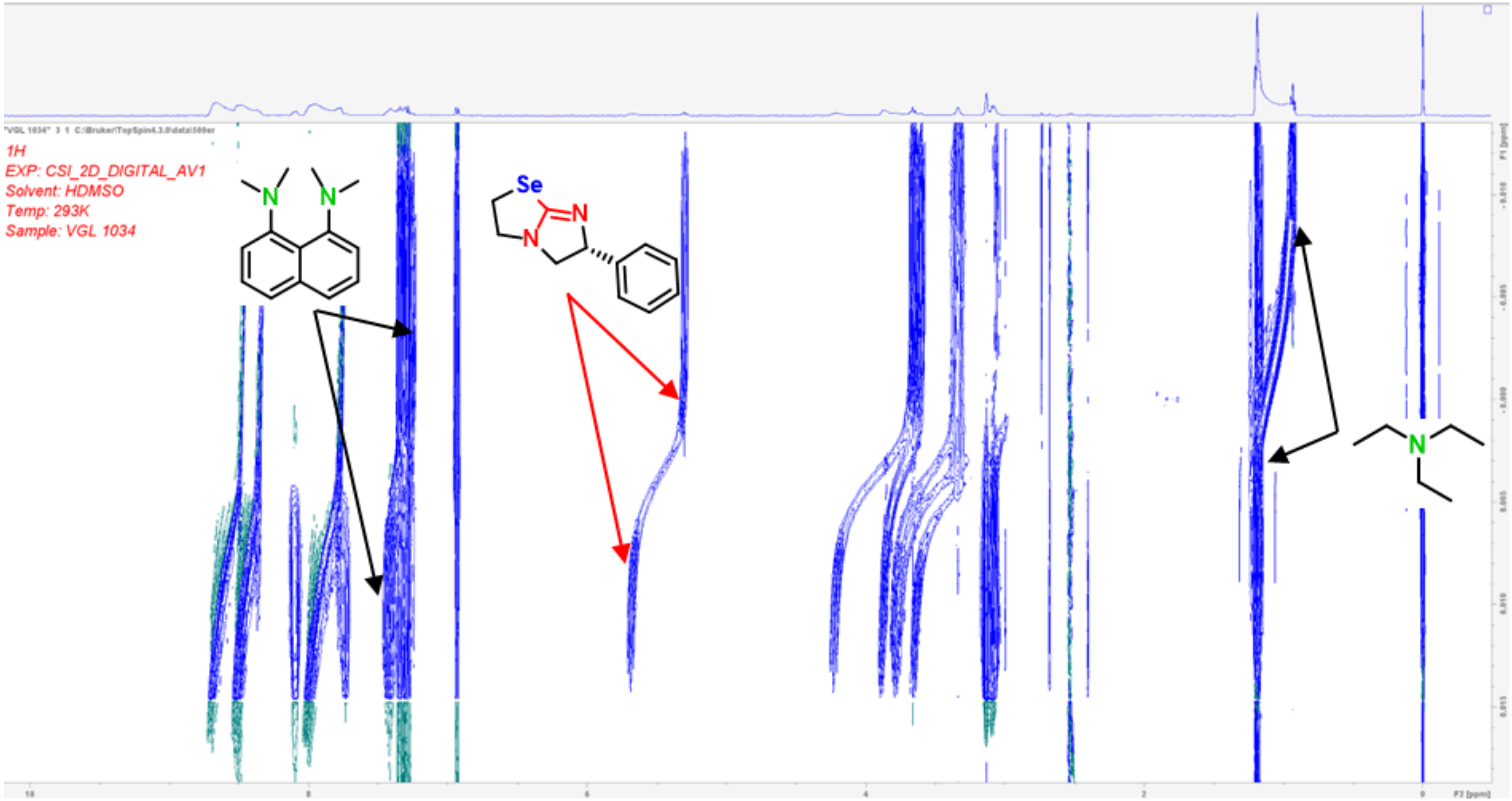

Figure S89: CSI experiment for ISeU1 - SeTM in DMSO.

IU5 - OHyperBTM

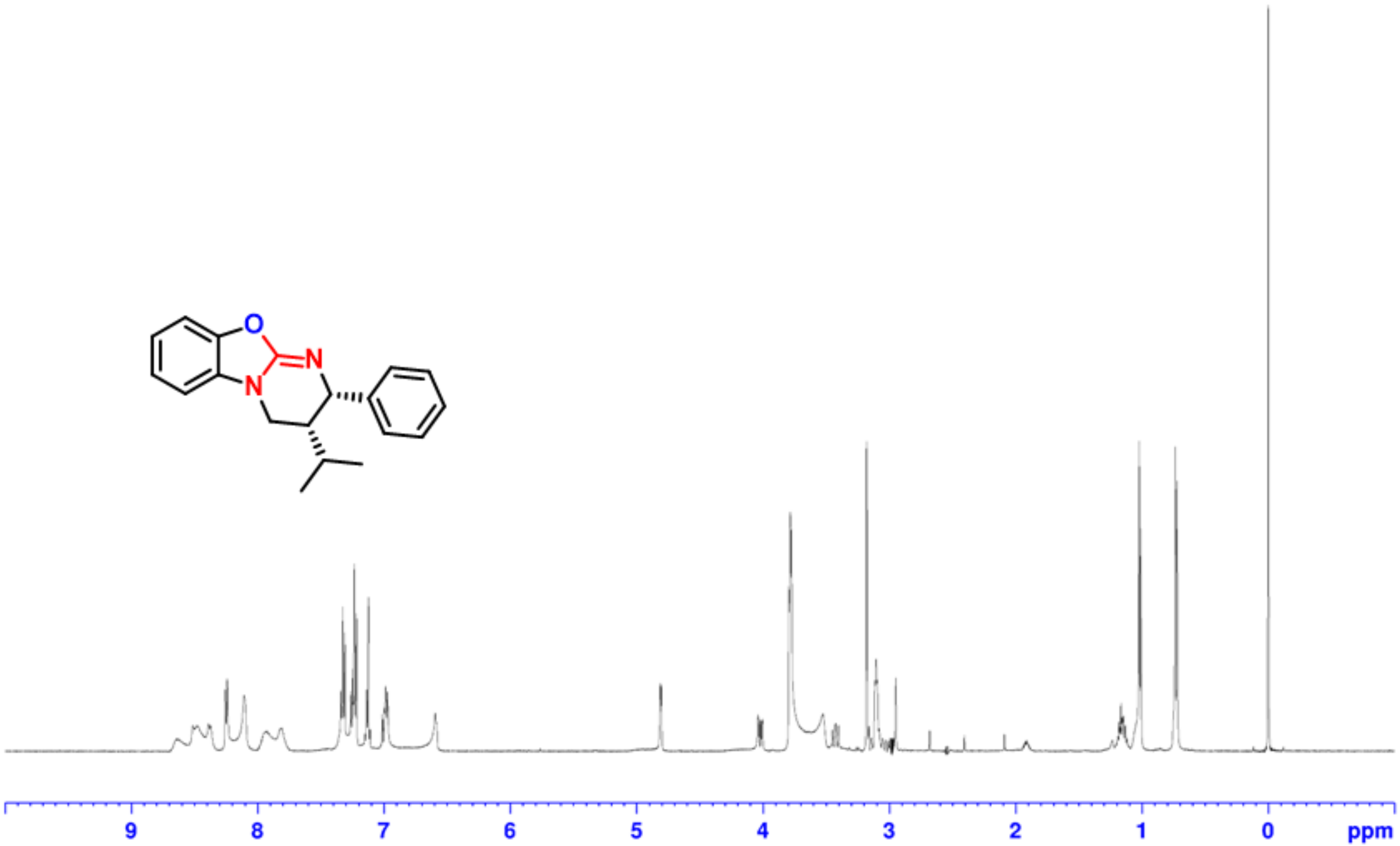

Figure S90: [1]H experiment over whole sample of CSI experiment of IU5 - OHyperBTM in DMSO.

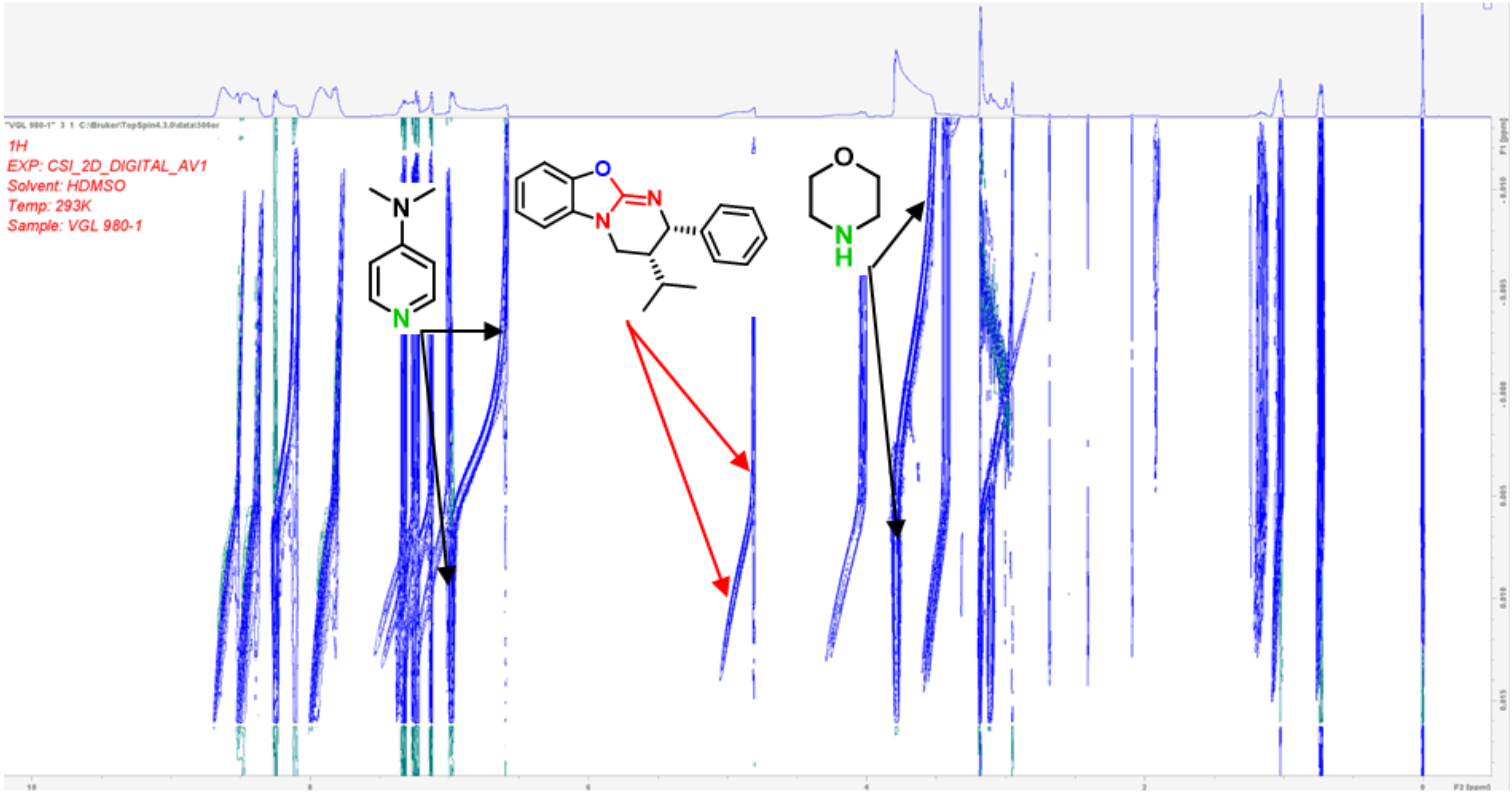

Figure S91: CSI experiment for IU5 - OHyperBTM in DMSO.

ISeU5 - SeHyperBTM

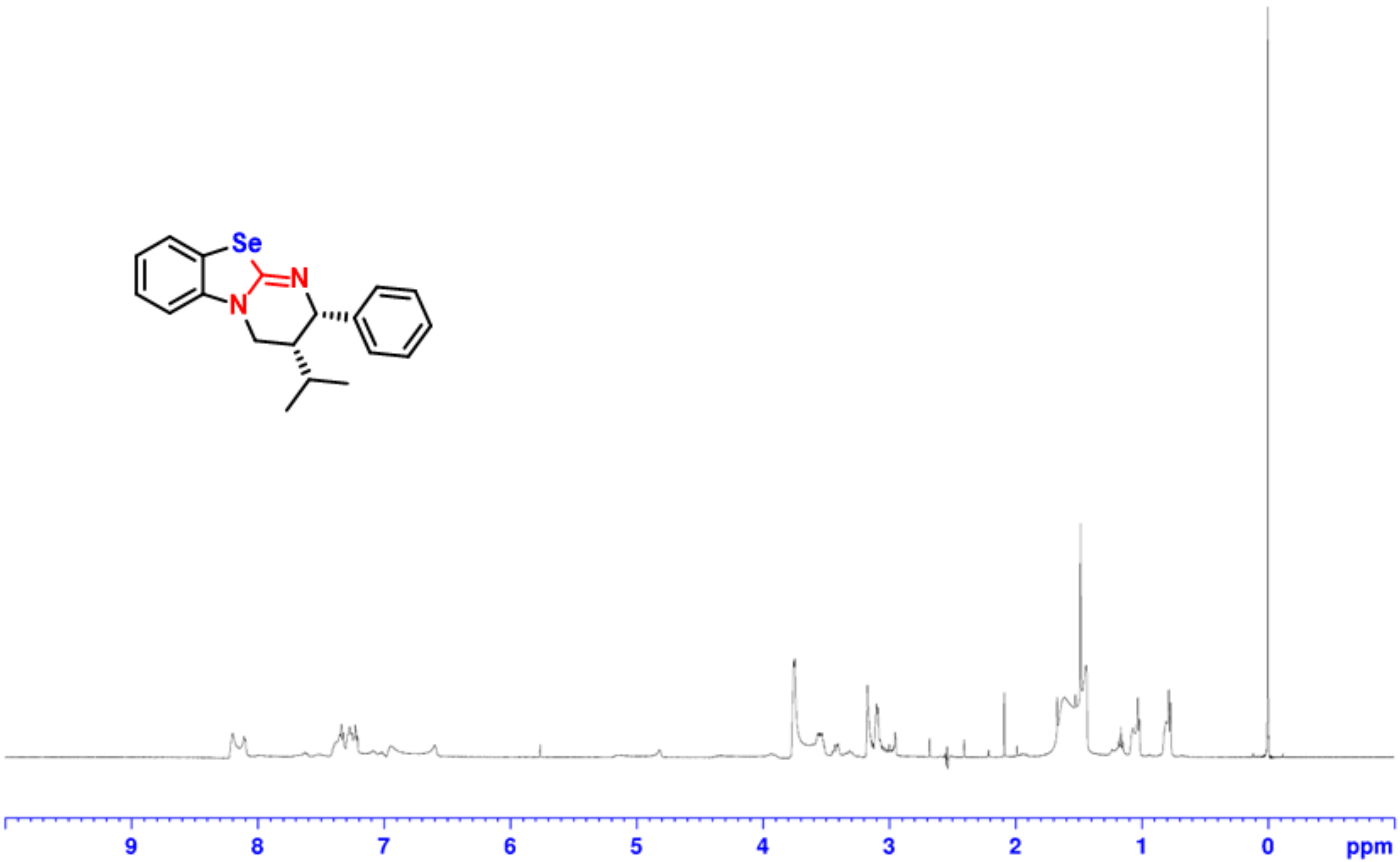

Figure S92: ^{1}H experiment over whole sample of CSI experiment of ISeU5 - SeHyperBTM in DMSO.

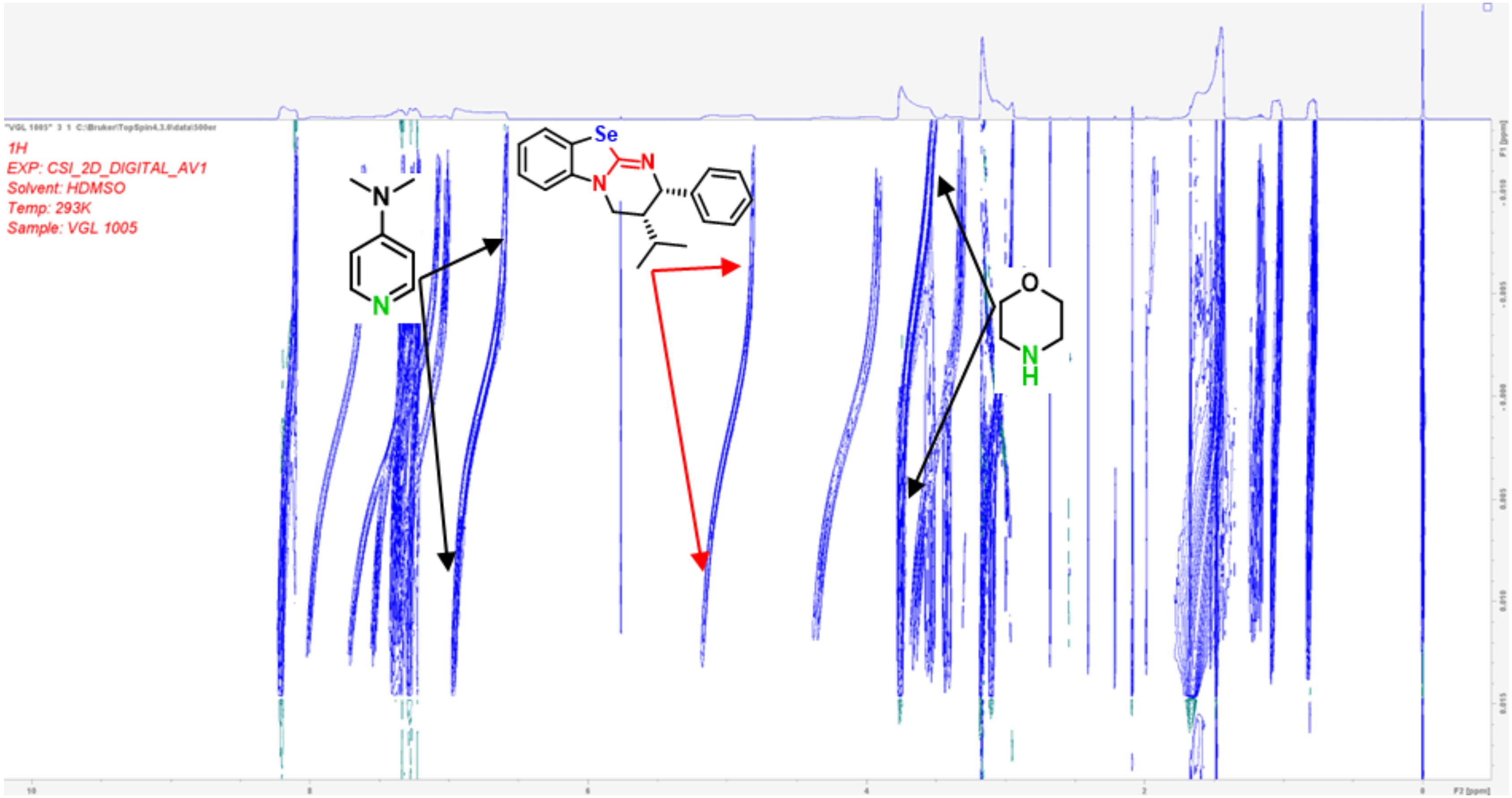

Figure S93: CSI experiment for ISeU5 - SeHyperBTM in DMSO.
